# Advances and mechanistic insight on the catalytic Mitsunobu reaction using recyclable azo reagents†
†Electronic supplementary information (ESI) available: Full experimental details and copies of analytical data. See DOI: 10.1039/c6sc00308g


**DOI:** 10.1039/c6sc00308g

**Published:** 2016-04-13

**Authors:** Daisuke Hirose, Martin Gazvoda, Janez Košmrlj, Tsuyoshi Taniguchi

**Affiliations:** a Graduate School of Natural Science and Technology , Kanazawa University , Kakuma-machi , Kanazawa 920-1192 , Japan; b Faculty of Chemistry and Chemical Technology , University of Ljubljana , Večna pot 113, SI-1000 , Ljubljana , Slovenia . Email: janez.kosmrlj@fkkt.uni-lj.si; c School of Pharmaceutical Sciences , Institute of Medical , Pharmaceutical and Health Sciences , Kanazawa University , Kakuma-machi , Kanazawa 920-1192 , Japan . Email: tsuyoshi@p.kanazawa-u.ac.jp

## Abstract

Ethyl 2-arylhydrazinecarboxylates can work as organocatalysts for Mitsunobu reactions because they provide ethyl 2-arylazocarboxylates through aerobic oxidation with a catalytic amount of iron phthalocyanine. First, ethyl 2-(3,4-dichlorophenyl)hydrazinecarboxylate has been identified as a potent catalyst, and the reactivity of the catalytic Mitsunobu reaction was improved through strict optimization of the reaction conditions. Investigation of the catalytic properties of ethyl 2-arylhydrazinecarboxylates and the corresponding azo forms led us to the discovery of a new catalyst, ethyl 2-(4-cyanophenyl)hydrazinecarboxylates, which expanded the scope of substrates. The mechanistic study of the Mitsunobu reaction with these new reagents strongly suggested the formation of betaine intermediates as in typical Mitsunobu reactions. The use of atmospheric oxygen as a sacrificial oxidative agent along with the iron catalyst is convenient and safe from the viewpoint of green chemistry. In addition, thermal analysis of the developed Mitsunobu reagents supports sufficient thermal stability compared with typical azo reagents such as diethyl azodicarboxylate (DEAD). The catalytic system realizes a substantial improvement of the Mitsunobu reaction and will be applicable to practical synthesis.

## Abstract 2

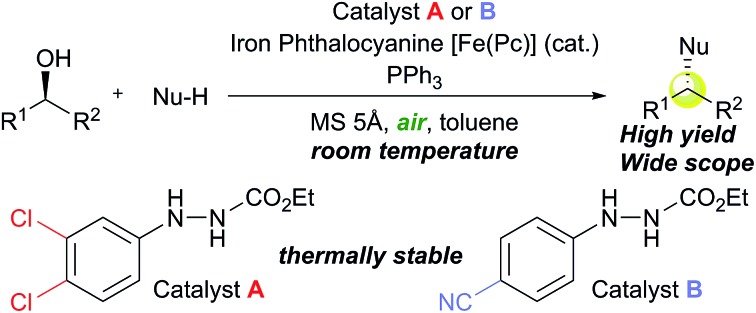
Catalytic Mitsunobu reactions have been substantially improved based on strict optimization, mechanistic studies and discovery of a new catalyst.

## Introduction

Many reactions have been utilized as important tools in synthetic organic chemistry. “Name reactions”, such as Wittig, Suzuki–Miyaura, and Mitsunobu, to name just a few, have an outstanding utility that has influenced broad fields of academia and industry.^1^ In view of economic and environmental concerns however, many of these synthetic methods suffer from serious limitations, diminishing their practical applicability. Therefore, substantial improvements of known synthetic protocols are currently an important subject in chemistry.

Indeed, the Mitsunobu reaction is a typical example including both a wide utility and serious drawbacks.^2^ The reaction is one of the oxidation–reduction condensations reported by Mitsunobu and co-workers in 1967.^3^ Since then, it has been widely used for the substitution of hydroxyl groups or inversion of the stereochemistry of secondary alcohols. Typically, diethyl azodicarboxylate (DEAD) and triphenylphosphine are employed as the oxidant and reducing agent in the Mitsunobu reaction, but production of a large amount of waste, *i.e.*, diethyl hydrazinedicarboxylate and triphenylphosphine oxide, is unavoidable. These byproducts often contaminate the desired product. In addition, DEAD is hazardous due to its toxicity and potential explosiveness. As a result, the use of the Mitsunobu reaction tends to be avoided in practical synthesis on plant scales.^4^

**Fig. 1:**
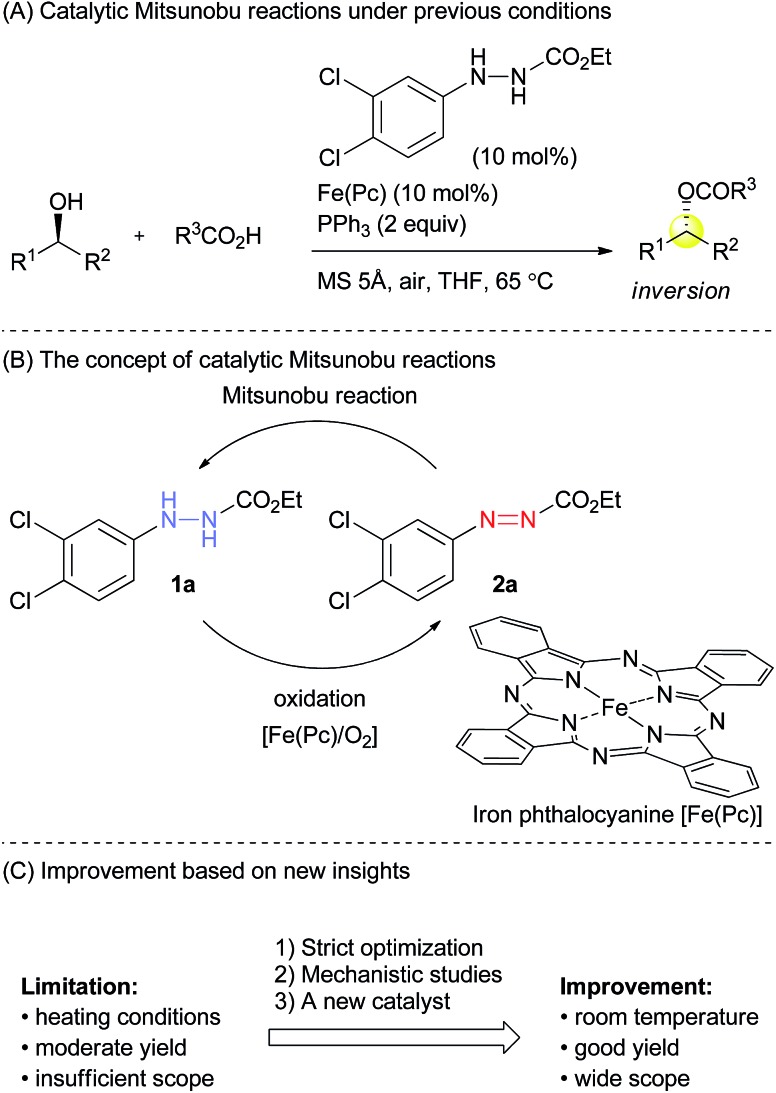
An outline of the catalytic Mitsunobu reaction.

Several modified methods have been developed to facilitate the removal of the waste generated by the Mitsunobu reaction.^5^ However, there has been no substantial approach to reducing the problematic waste in the Mitsunobu reaction until the report on the catalytic Mitsunobu reaction by Toy in 2006.^6^ Toy succeeded in reducing DEAD in the Mitsunobu reaction to a catalytic amount (10 mol%) by employing a sacrificial oxidative reagent, *i.e.*, iodobenzene diacetate. Recently, Mitsunobu-type reactions without azo reagents were reported.^7^ In 2013, we reported the second example of the catalytic Mitsunobu reaction with azo reagents that are recyclable through aerobic oxidation with iron phthalocyanine (Fig. 1A).^8^ Ethyl 2-(3,4-dichlorophenyl)hydrazinecarboxylate (**1a**) has been tentatively identified as the best catalyst. A catalytic concept of this reaction is beneficial from the viewpoint of green chemistry because atmospheric oxygen is economically and environmentally ideal as a sacrificial oxidant to generate a reactive azo form **2a** (Fig. 1B). However, the scope of substrates and product yields were still moderate, and the reaction required heating conditions to obtain the products in acceptable yields. Thus, the applicability of the method was still inferior to that of the original Mitsunobu reaction.

The effect of substituents on the aromatic ring of the hydrazine catalysts was drastic. Clearly, electronic properties of catalysts affected both the Mitsunobu reactivity of the azo form as well as the aerobic oxidation of the hydrazine form. At first glance, these seem incompatible because electron-withdrawing groups would promote the addition reaction of triphenylphosphine to the azo form but would suppress oxidation of the hydrazine form to the azo form. In the case of electron-donating groups there is the same dilemma, though the situation is interchanged. We presumed that the 3,4-dichlorophenyl group had an electronic property that made the two processes moderately compatible.

Quite recently, we have reported a detail of the aerobic oxidation process of 2-arylhydrazinecarboxylates with iron phthalocyanine, indicating two important observations.^9^ First, the oxidation process was promoted in apolar solvents such as toluene or dichloromethane, and second, electron-withdrawing substituents at the aryl group did not suppress the hydrazine-to-azo compound oxidation. Interestingly, halogen atoms at the *para*-position rather promoted the reaction. Thus, this study provided us important insights to improve the catalytic Mitsunobu reaction.

Providing the serious limitations indicated in Fig. 1C are avoided, the catalytic Mitsunobu reaction will gain a large potential in practical synthesis.^10^ In this paper, we describe new advances in our catalytic Mitsunobu reaction including substantial improvement of the reaction and insights into the reaction mechanism.

## Results and discussion

### Strict optimization of the reaction conditions

We previously found that the combination of ethyl 2-(3,4-dichlorophenyl)hydrazinecarboxylate (**1a**) and iron phthalocyanine [Fe(*Pc*)] formed an optimum catalytic system (both 10 mol%), and that addition of activated molecular sieves was required to induce the reaction (*vide infra*).^8^ We tentatively improved the yields of the products by using 3,5-dinitrobenzoic acid as a nucleophile when secondary alcohols were used as substrates.^8^ We employed a model reaction between (*S*)-ethyl lactate (**3**, 99 : 1 er) and 4-nitrobenzoic acid (**4**) using this catalytic system to strictly optimize the conditions. The reaction between **3** and **4** in heating THF (65 °C) gave ester product **5** in 50% yield and in 97% inversion (Table 1, entry 1).

**Table 1:** The effect of solvents^*a*^

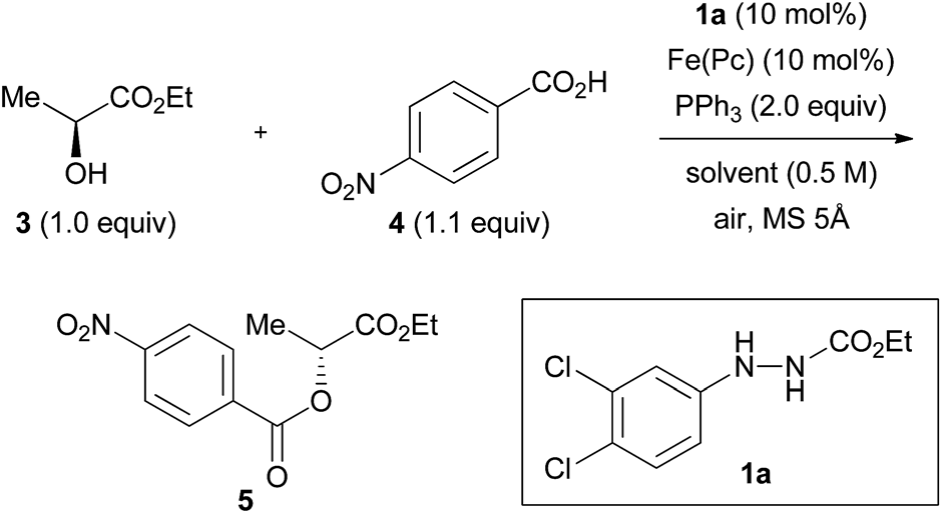					
Entry	Solvent	Temp. (°C)	Time (h)	Yield (%)	Er
1	THF	65	24	50	97 : 3
2	1,4-Dioxane	65	24	46	96 : 4
3	CPME	65	24	70	97 : 3
4	MTBE	55^*b*^	24	76	98 : 2
5	DME	65	24	40	58 : 42
6	MeCN	65	24	14	19 : 81
7	*n*-Hexane	65	24	69	99 : 1
8	Toluene	65	24	74	94 : 6
9	Toluene	110^*b*^	12	78	49 : 51
10	Toluene	rt	29	88	99 : 1
11	CPME	rt	36	75	99 : 1
12	CHCl_3_	62^*b*^	24	80	38 : 62
13	CH_2_Cl_2_	rt	48	75	12 : 88
14	PhCl	65	24	70	59 : 41
15	PhCF_3_	65	24	75	95 : 5

^*a*^Reaction conditions: **3** (1.0 mmol), **4** (1.1 mmol), catalyst **1a** (0.10 mmol), Fe(*Pc*) (0.10 mmol), PPh_3_ (2.0 mmol), solvent (2 mL), MS 5 Å (500 mg) under air atmosphere. MS 5 Å was activated by heating using a heat gun (*ca.* 450 °C) *in vacuo* (*ca.* 0.1 mmHg) for 5 min.

^*b*^Under reflux.

In the previous study, the effect of solvents was investigated at a very preliminary stage using unoptimized catalysts.^11^ We could not find a large effect of the solvents at that time, and thereby, the effects of solvents and temperature were re-investigated using the optimum catalytic system (Table 1).^12^ Ether solvents such as 1,4-dioxane, cyclopentyl methyl ether (CPME)^13^ and *tert*-butyl methyl ether (MTBE), except for dimethoxyethane (DME), provided product **5** in a high inversion ratio (entries 2–5), whereas acetonitrile gave a contrasting result (entry 6).^14^ Reactions in hydrocarbon solvents such as *n*-hexane and toluene at 65 °C afforded good results (entries 7 and 8). However, chlorinated solvents gave product **5** in a low inversion ratio, though the total product yield was good (entries 12–14). This drastic change in the results was attributed to the presence of chlorine atoms in the solvent, and is based on the fact that the reaction in α,α,α-trifluorotoluene^15^ provided similar results to those in toluene (entry 15). The enantiomeric ratio was sensitive to temperature in the reaction in toluene (entries 8–10). To our delight, the reaction in toluene at room temperature provided product **5** in an excellent yield (88%) and in a perfect inversion ratio. CPME also gave a relatively good result for the reaction at room temperature. The reactions were basically clean. In the case of low yields of the product, the starting materials remained unconsumed.

The effect of molecular sieves was drastic, and no reaction was induced in their absence (Table 2, entry 1).^16^ This is likely due to the high moisture sensitivity of the intermediate generated from the azo form of catalyst **1a** and triphenylphosphine. Molecular sieves would serve for removing residual moisture as well as water generated by the iron-catalyzed aerobic oxidation of the hydrazine catalyst. The use of at least 500 mg MS 5 Å (1.0 mmol scale), activated by heating with a heat gun (*ca.* 450 °C) under reduced pressure (*ca.* 0.1 mmHg), was desirable to obtain product **5** in a good yield (entries 2–5). MS 4 Å and MS 3 Å were ineffective in the present reaction (entries 6 and 7).

**Table 2:** The effect of desiccants^*a*^

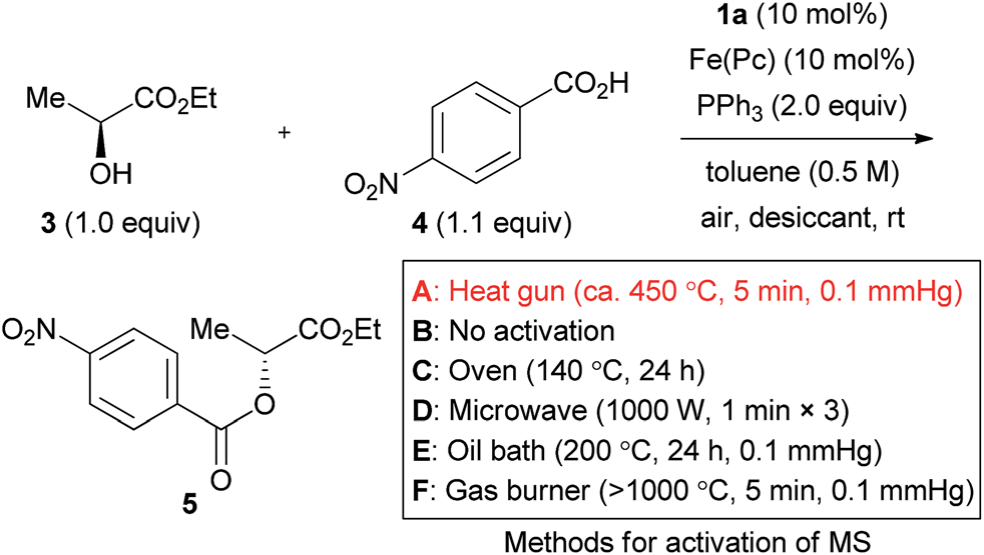				
Entry	Desiccant	Amount (mg mmol^–1^)	Yield (%)	Er
1	None	—	0	—
2	MS 5 Å (**A**)	100	32	99 : 1
3	MS 5 Å (**A**)	300	77	99 : 1
4	MS 5 Å (**A**)	500	88	99 : 1
5	MS 5 Å (**A**)	1000	95	99 : 1
6	MS 4 Å (**A**)	500	32	97 : 3
7	MS 3 Å (**A**)	500	26	98 : 2
8	MS 5 Å (**B**)	500	13	96 : 4
9	MS 5 Å (**C**)	500	16	91 : 9
10	MS 5 Å (**D**)	500	25	97 : 3
11	MS 5 Å (**E**)	500	67	98 : 2
12	MS 5 Å (**F**)	500	94	99 : 1
13	Na_2_SO_4_	500	0	—
14	CaSO_4_	500	0	—
15	MgSO_4_	500	0	—

^*a*^Reaction conditions: **3** (1.0 mmol), **4** (1.1 mmol), catalyst **1a** (0.10 mmol), Fe(*Pc*) (0.10 mmol), PPh_3_ (2.0 mmol), toluene (2 mL), desiccant (0–1000 mg) for 24–48 h at room temperature under air atmosphere. Methods for activation of molecular sieves: **A**: heated using a heat gun (*ca.* 450 °C) *in vacuo* (*ca.* 0.1 mmHg) for 5 min; **B**: not activated; **C**: heated in an oven (140 °C) for 24 h; **D**: heated using a microwave (1000 W for 1 min, three times); **E**: heated using an oil bath (200 °C) *in vacuo* (*ca.* 0.1 mmHg) for 24 h; **F**: heated using a gas burner (>1000 °C) *in vacuo* (*ca.* 0.1 mmHg) for 5 min.

Various “traditional methods” for the activation of molecular sieves are used in many laboratories. Representative activation methods were tested to assure a reliable experimental procedure. The use of MS 5 Å without activation gave the product in a very poor yield (entry 8). MS 5 Å heated for 24 h at 140 °C in an oven were also ineffective (entry 9). Although heating using a microwave is sometimes used for activation of molecular sieves, this method did not afford a good result in the present reaction (entry 10). When the reaction was tested with MS 5 Å activated through heating at 200 °C with an oil bath under reduced pressure (*ca.* 0.1 mmHg), the product yield was still insufficient (entry 11). Heating using a flame under reduced pressure would be a strict method for activation of molecular sieves, and this method provided product **5** in an excellent 94% yield (entry 12). As a result, and from the viewpoints of safety and convenience, we consider the activation with a heat gun as the method of choice. Incidentally, sulfate salts did not work as a desiccant in the reaction (entries 13–15).

The concentration of the reactants is likely to affect the product yield (Table 3, entries 1–4 and 7). The reaction was promoted and gave improved yields of product **5** in high concentrations (2.0 M or 4.0 M) (entries 4 and 7). When the amount of triphenylphosphine was decreased to 1.5 equivalent in the reaction in high concentration (2.0 M or 4.0 M), a good yield was maintained in this model reaction (entries 5 and 8). However, the use of a lower amount (1.1 equiv.) of triphenylphosphine diminished the yield of product **5** (entries 6 and 9). High concentration conditions would be beneficial to a practical synthesis because the solvent can be saved. The good result was reproducible in a scale-up experiment (10 mmol), though the reaction time was somewhat prolonged (entry 7, results in parentheses). Triphenylphosphine is sometimes replaced with trialkylphosphines because they often provide good results due to their high nucleophilicity.^17^ We tested a representative reaction with tri-*n*-butylphosphine, but the result was very poor (entry 8, results in parentheses). TLC analysis of the reaction mixture implied decomposition of the iron phthalocyanine presumably through strong coordination with the tri-*n*-butylphosphine. When most of the triphenylphosphine was consumed in the reaction, the Mitsunobu catalyst was detected as the azo form using TLC. The latter was easily recovered in 80–90% yield using silica gel chromatography due to its low polarity. The hydrazine form of the catalyst, if it remained in the reaction mixture, usually did not cause problems in the purification of the product. Finally, iron phthalocyanine could be easily removed using filtration of the reaction mixture through a pad of Celite® or filter paper. The impact of decreasing the amount of hydrazine catalyst **1a** seemed to be larger than that of decreasing the amount of iron phthalocyanine (entries 10–15). It is noteworthy that good results were maintained with as low as 1 mol% of iron phthalocyanine (entries 11 and 12) indicating that its amount can be flexibly changed depending on the substrates or situations of the reactions. No reaction was induced in the absence of the iron catalyst.^8^,^9^

**Table 3:** Effects of the amounts of reagents and concentrations^*a*^

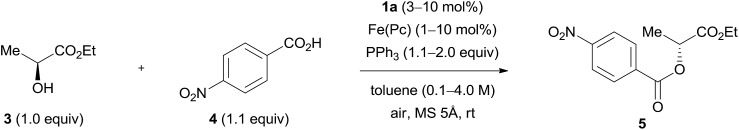							
Entry	**1a** (mmol%)	Fe(*Pc*) (mmol%)	PPh_3_ (equiv.)	Conc. (M)	Time (h)	Yield (%)	Er
1	10	10	2.0	0.1	52	80	98 : 2
2	10	10	2.0	0.5	29	88	99 : 1
3	10	10	2.0	1.0	18	91	98 : 2
4	10	10	2.0	2.0	14	97	98 : 2
5	10	10	1.5	2.0	14	91	99 : 1
6	10	10	1.1	2.0	12	68	99 : 1
7	10	10	2.0	4.0	12 (24)^*b*^	93 (88)^*b*^	99 : 1 (99 : 1)^*b*^
8	10	10	1.5	4.0	12 (48)^*c*^	92 (10)^*c*^	99 : 1 (46 : 54)^*c*^
9	10	10	1.1	4.0	12	76	99 : 1
10	10	5	1.5	4.0	21	84	99 : 1
11	10	1	1.5	4.0	18	81	99 : 1
12	10	1	2.0	4.0	36	89	99 : 1
13	5	10	1.5	4.0	38	78	99 : 1
14	5	5	1.5	4.0	24	76	99 : 1
15	3	3	1.5	4.0	39	68	99 : 1

^*a*^Reaction conditions: **3** (1.0 mmol), **4** (1.1 mmol), **1a** (0.10, 0.050 or 0.030 mmol), Fe(*Pc*) (0.10, 0.050, 0.030 and 0.010 mmol), PPh_3_ (2.0, 1.5 or 1.1 mmol), toluene (10, 2, 1, 0.5 or 0.25 mL), MS 5 Å (500 mg) at room temperature under air atmosphere unless otherwise noted. MS 5 Å was activated by heating using a heat gun (*ca.* 450 °C) *in vacuo* (*ca.* 0.1 mmHg) for 5 min.

^*b*^The reaction was performed on the 10 mmol scale.

^*c*^PBu_3_ was used instead of PPh_3_.

### Kinetic properties of the ethyl 2-arylazocarboxylates

**Fig. 2:**
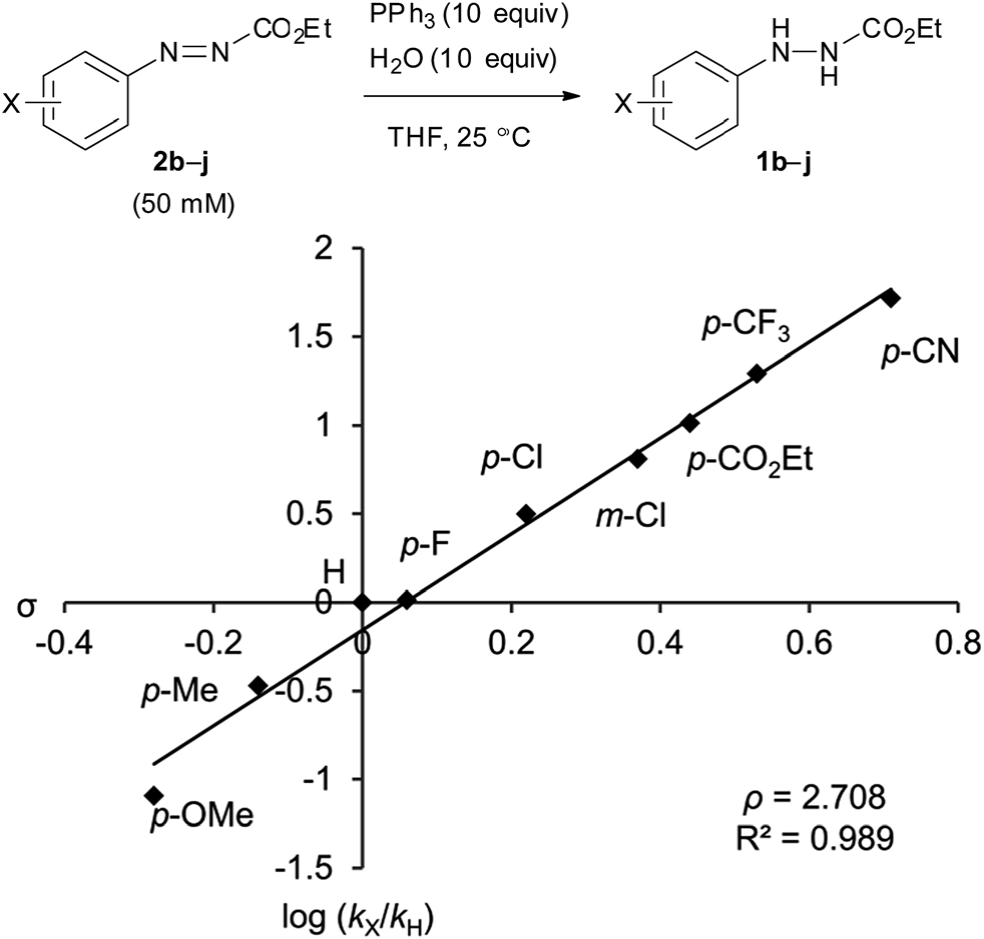
The Hammett plot of the reactions of ethyl 2-arylazocarboxylates with PPh_3_ in the presence of water. **b**: *p*-OMe, **c**: *p*-Me, **d**: H, **e**: *p*-F, **f**: *p*-Cl, **g**: *m*-Cl, **h**: *p*-CO_2_Et, **i**: *p*-CF_3_, **j**: *p*-CN.

The catalytic cycle between the hydrazines and azo compounds would affect the efficiency of the formation of an alkoxyphosphonium intermediate to provide the final product. We conducted kinetic experiments to investigate the substituent effect of azo compounds **2b–j** in the reaction with triphenylphosphine (Fig. 2). The analysis of a mixture of **2b–j** and triphenylphosphine (10 equiv.) in CDCl_3_ using ^1^H NMR spectroscopy revealed the presence of some starting azo compounds after 10 hours. In contrast, in an independent experiment, ^1^H NMR analysis showed that DEAD immediately disappeared under the same reaction conditions, indicating an irreversible process in this case.^18^ Obviously, the addition of triphenylphosphine to ethyl 2-arylazocarboxylates is reversible, and the formation of adducts is less favorable as compared to DEAD. Therefore, reaction rates were estimated from the model reaction of azo compounds **2b–j** (50 mM) with excessive amounts (10 equiv.) of triphenylphosphine and water in THF at 25 °C. The reactions were monitored by measuring the absorbance of the azo compounds **2b–j** at *λ* = 419–450 nm. Rate constants were calculated from plots of a pseudo-first-order dependence.

The Hammett plot for these reactions shows a linear fit with a relatively large positive slope value of *ρ* = +2.71 (Fig. 2). The value is close to that of the alkaline hydrolysis of benzoate esters (*ρ* = +2.51).^19^ The result reflects a dependence of the electronic density at the aromatic ring of azo compounds in the rate of the addition reaction of triphenylphosphine. Ethyl 2-(3,4-dichlorophenyl)azocarboxylate (**2a**) was also applied to the kinetic experiment, and its reaction rate (*k*_obs_ = 8.5 × 10^–2^ min^–1^) was approximately 13.7 times faster than that of ethyl 2-phenylazocarboxylate (**2d**, *k*_obs_ = 6.2 × 10^–3^ min^–1^). In addition, it is still 2.3 times faster compared to that of ethyl 2-(3-chlorophenyl)azocarboxylate (**2g**, *k*_obs_ = 3.75 × 10^–2^ min^–1^). This supports the high reactivity of **2a** in the catalytic Mitsunobu reaction.

When benzoic acid or 4-nitrobenzoic acid (each 10 equiv.) were added to the reaction system with **2d**, only a minor impact to the reaction rate was noted (**2d** with benzoic acid: *k*_obs_ = 7.1 × 10^–3^ min^–1^; **2d** with 4-nitrobenzoic acid: *k*_obs_ = 6.8 × 10^–3^ min^–1^). This observation supports that the model reaction reflects the reactivity of azo compounds toward triphenylphosphine and indicates that acids do not kinetically affect the reaction.

**Fig. 3:**
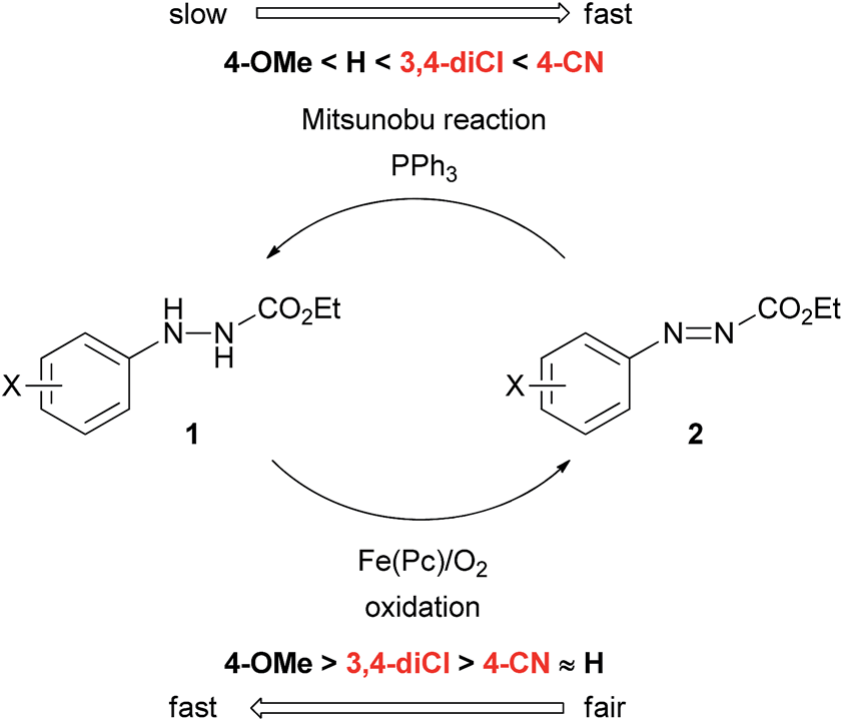
An outline of the substituent effect of Mitsunobu catalysts in the catalytic cycle.

**Fig. 4:**
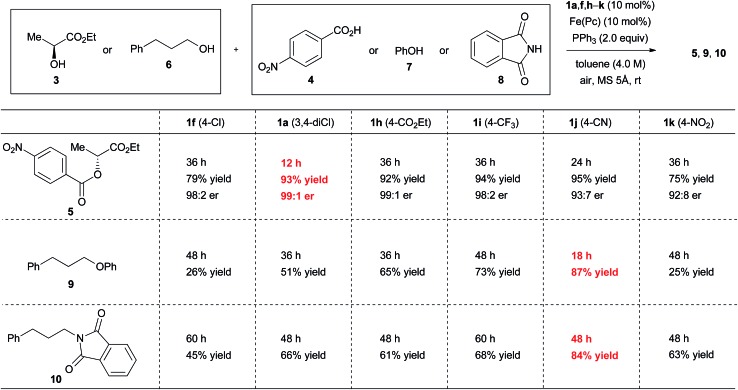
Catalytic activity of representative hydrazine catalysts. Reaction conditions: alcohol (1.0 mmol), nucleophile (1.1 mmol), catalyst **1a**, **f**, **h–k** (0.10 mmol), Fe(*Pc*) (0.10 mmol), PPh_3_ (2.0 mmol), toluene (0.25 mL), MS 5 Å (500 mg) at room temperature under air atmosphere unless otherwise noted. MS 5 Å was activated by heating using a heat gun (*ca.* 450 °C) *in vacuo* (*ca.* 0.1 mmHg) for 5 min. The reaction between **6** and **8** to give **10** was performed in 2 mL of toluene (0.5 M).

The kinetics of the catalytic aerobic oxidation of ethyl 2-arylhydrazinecarboxylates (**1**) with iron phthalocyanine basically show zero-order dependence, but the substituent effect is of irregular tendency probably due to the participation of radical species in the mechanism.^9^ The reaction rates of aerobic oxidation of ethyl 2-(4-chlorophenyl)hydrazinecarboxylate (**1f**) and ethyl 2-(4-bromophenyl)hydrazinecarboxylate to the corresponding azo compounds are approximately 1.5 times faster than that of ethyl 2-phenylhydrazinecarboxylate (**1d**).^9^ In the model reaction, in dichloromethane as a solvent, the aerobic oxidation of ethyl 2-(3,4-dichlorophenyl)hydrazinecarboxylate (**1a**) with iron phthalocyanine is completed within 2 hours. This is clearly faster than the oxidation (4 hours)^9^ of ethyl 2-phenylhydrazinecarboxylate (**1d**), though the kinetics of the reaction of **1a** do not show a clear zero-order dependence (Fig. S14 in the ESI†). Thus, the 4-chlorine atom on the aromatic ring of **1a** promotes oxidation to the corresponding azo form **2a** by stabilization of the intermediary radical species, whereas the 3-chlorine atom of azo compound **2a** contributes to an increased electrophilicity by its inductive effect. This is the reason why azo compound **2a** operates as a good catalyst in the catalytic Mitsunobu reaction. In short, two processes involving Mitsunobu activity and hydrazine re-oxidation are compatible through the 3,4-dichlorophenyl group (Fig. 3). The catalytic activity of ethyl 2-(4-chlorophenyl)hydrazinecarboxylate (**1f**) was insufficient under the optimal conditions compared to that of **1a** (Fig. 4).

Given the above considerations, ethyl 2-arylhydrazinecarboxylates with strong electron-withdrawing groups on the aromatic ring should be more effective catalysts as these groups should promote the Mitsunobu reaction without significantly suppressing the aerobic oxidation process. For instance, as monitored using NMR spectroscopy, the aerobic oxidation of ethyl 2-(4-cyanophenyl)hydrazinecarboxylate (**1j**) was completed within 5 hours, which was roughly the same reaction time as that of ethyl 2-phenylhydrazinecarboxylate (**1d**) (*ca.* 4 hours).^9^ On the other hand, higher electrophilicity of 2-(4-cyanophenyl)azocarboxylate (**2j**) over 3,4-dichlorophenyl derivative **2a** is consistent with the higher (3.8 times) reaction rates of **2j** (*k*_obs_ = 3.2 × 10^–1^ min^–1^) over **2a** (Fig. 3). This suggested that ethyl 2-(4-cyanophenyl)hydrazinecarboxylate (**1j**) might work as a good catalyst in the catalytic Mitsunobu reaction.

When ethyl 2-(4-cyanophenyl)hydrazinecarboxylate (**1j**) was used in the reaction between (*S*)-ethyl lactate (**3**) and 4-nitrobenzoic acid (**4**) under optimal conditions, product **5** was obtained in an excellent yield, although with a slightly decreased inversion ratio (Fig. 4). On the other hand, when phenol (**7**) or phthalimide (**8**) was used as the reaction partner of 3-phenylpropanol (**6**), both reactions using **1j** provided better results (87% and 84% yields) than the reactions with **1a** (51% and 66% yields). Although 2-(4-nitrophenyl)hydrazinecarboxylate (**1k**) should generate a strongly electrophilic azo compound,^20^ the results with this catalyst were disappointing. Gradual decomposition of **1k** or its azo form was observed in the reaction with triphenylphosphine using ^1^H NMR analysis, which appears to be the main reason for the poor results.^21^

The reaction rate of ethyl 2-[4-(ethoxycarbonyl)phenyl]azocarboxylate (**2h**, *k*_obs_ = 6.4 × 10^–2^ min^–1^) and ethyl 2-[4-(trifluoromethyl)phenyl]azocarboxylate (**2i**, *k*_obs_ = 1.2 × 10^–1^ min^–1^) with triphenylphosphine was roughly close to that of **2a**. Good yields of ester **5** were obtained in the reaction between (*S*)-ethyl lactate (**3**) and 4-nitrobenzoic acid (**4**) using the hydrazine forms **1h** and **1i** as a catalyst, but reaction times were prolonged (Fig. 4). When phenol (**7**) or phthalimide (**8**) were used as a nucleophile in the reaction with 3-phenylpropanol (**6**), catalysts **1h** and **1i** did not provide better results than catalyst **1j**, though catalyst **1i** showed somewhat improved results compared with catalyst **1a**. Thus, catalyst **1h** showed reactivity similar to that of **1a**, and the position of reactivity for catalyst **1i** is likely to lie between **1a** and **1j**. These trends are consistent with the results of the Hammett study. Incidentally, when model experiments of iron-catalyzed aerobic oxidation of **1h** and **1i** were conducted in dichloromethane, the reactions were completed at 4 h and 6 h, respectively (see the ESI†). The trend of the oxidation process is similar to that of other hydrazide derivatives.^9^

The above results imply that there is no perfect catalyst for the catalytic Mitsunobu reaction. Instead two catalysts can complement each other. In short, ethyl 2-(3,4-dichlorophenyl)hydrazinecarboxylate (**1a**) would be suitable for the reactions of carboxylic acids whereas 2-(4-cyanophenyl)hydrazinecarboxylate (**1j**) could serve for the reactions of other nucleophiles except for carboxylic acids.

### Scope of substrates using the optimized protocol

**Fig. 5:**
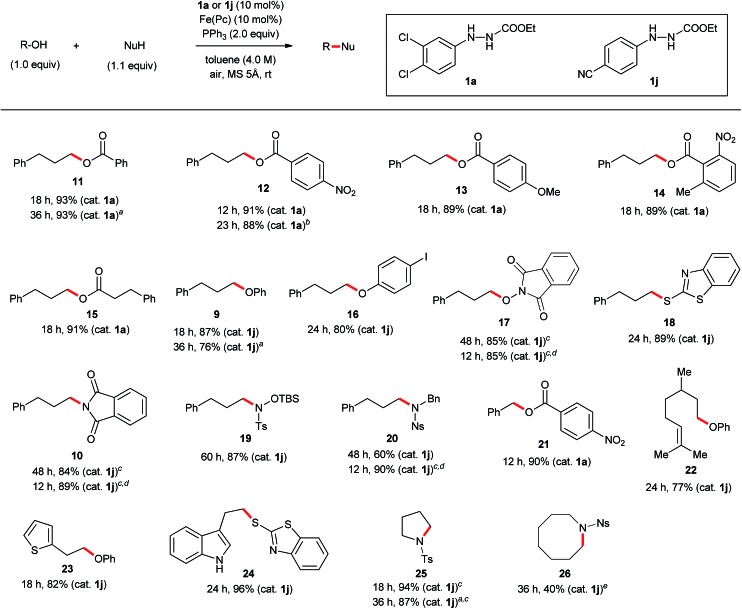
Reactions of primary alcohols with various nucleophiles. Reaction conditions: alcohol (1.0 mmol), nucleophile (1.1 mmol), catalyst **1a** or **1j** (0.10 mmol), Fe(*Pc*) (0.10 mmol), PPh_3_ (2.0 mmol), toluene (0.25 mL), MS 5 Å (500 mg) at room temperature under air atmosphere unless otherwise noted. MS 5 Å was activated by heating using a heat gun (*ca.* 450 °C) *in vacuo* (*ca.* 0.1 mmHg) for 5 min. ^*a*^1 mol% (0.010 mmol) of Fe(*Pc*) and 1.5 equiv. (1.5 mmol) of PPh_3_ were used. ^*b*^3 mol% (0.030 mmol) of catalyst **1a**, 3 mol% (0.030 mmol) of Fe(*Pc*), and 1.5 equiv. (1.5 mmol) of PPh_3_ were used. ^*c*^2 mL (0.5 M) of toluene was used. ^*d*^65 °C. ^*e*^20 mL (0.05 M) of toluene was used.

The discovery of new catalyst **1j** largely expanded the scope of the catalytic Mitsunobu reaction. Fig. 5 shows the results of catalytic Mitsunobu reactions applying catalyst **1a** or **1j** to various substrates. Typically, the reactions were performed under the optimal conditions that provided the best result (Table 3, entry 7), but more practical conditions (*e.g.*, Table 3, entry 11) were also applicable to several substrates. Reactions between 3-phenylpropanol and various carboxylic acids with catalyst **1a** provided the corresponding esters **11–15** in excellent yields. The reaction of the alcohol with phenols gave the corresponding ethers **9** and **16** in improved yields when catalyst **1j** was employed. An iodine atom was intact under the present conditions in the reaction of 4-iodophenol to give **16**. *N*-Hydroxyphthalimide also worked as a good nucleophile to give an *O*-alkylated product **17** in the presence of catalyst **1j**. Similarly, a sulfur nucleophile (2-mercaptobenzothiazole) underwent the Mitsunobu reaction with the alcohol to give the corresponding alkylated sulfide **18** in a good yield. Reactions of the alcohol with representative nitrogen nucleophiles were tested using catalyst **1j** and produced alkylated phthalimide **10**, and sulfonylamides **19** (ref. 22) and **20** (ref. 23) in good yields. Reactions with phthalimides and the nosylamide needed to be performed in 0.5 M solution due to the solubility issues. In such cases, heating the reaction mixture at 65 °C improved the results in reaction time and product yield. Alcohols sensitive to oxidative conditions were tested with several nucleophiles and were transformed into the corresponding Mitsunobu products **21–24** in good yields. It is noteworthy that a trisubstituted olefin, a thiophene and an indole were intact under the aerobic oxidation conditions. The catalytic Mitsunobu reaction using catalyst **1j** was applicable to intramolecular reactions of alkyl sulfonamides having a hydroxyl group to give the corresponding cyclic amines **25** and **26** (ref. 23*b*) in reasonable yields.

**Fig. 6:**
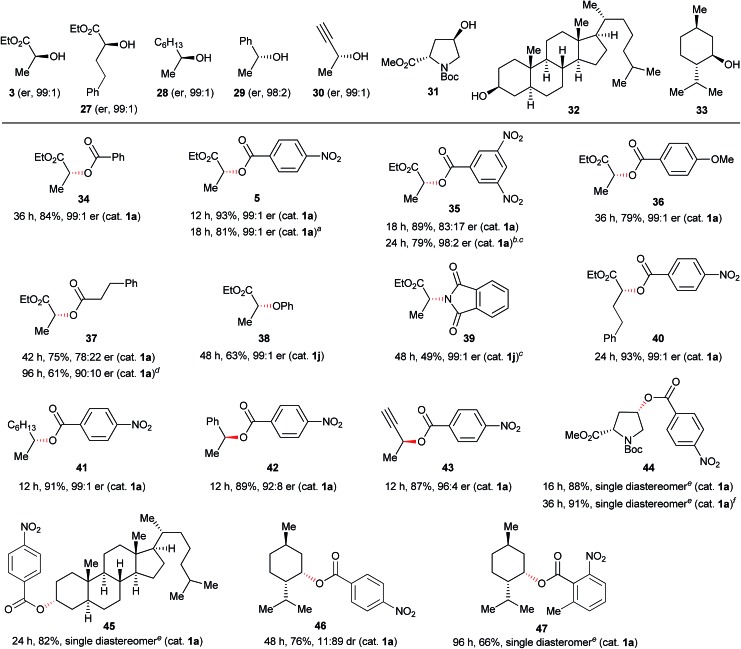
Reactions of secondary alcohols with various nucleophiles. Reaction conditions: alcohol (1.0 mmol), nucleophile (1.1 mmol), catalyst **1a** or **1j** (0.10 mmol), Fe(*Pc*) (0.10 mmol), PPh_3_ (2.0 mmol), toluene (0.25 mL), MS 5 Å (500 mg) at room temperature under air atmosphere unless otherwise noted. MS 5 Å was activated by heating using a heat gun (*ca.* 450 °C) *in vacuo* (*ca.* 0.1 mmHg) for 5 min. ^*a*^1 mol% (0.010 mmol) of Fe(*Pc*) and 1.5 equiv. (1.5 mmol) of PPh_3_ were used. ^*b*^THF was used as a solvent. ^*c*^2 mL (0.5 M) of solvent was used at 65 °C. ^*d*^0 °C. ^*e*^Determined using ^1^H NMR analysis of the crude product. ^*f*^3 mol% (0.030 mmol) of Fe(*Pc*) and 1.5 equiv. (1.5 mmol) of PPh_3_ were used.

Next, various combinations of secondary alcohols and nucleophiles were tested (Fig. 6). Reactions of (*S*)-ethyl lactate (**3**) with several aromatic carboxylic acids gave the corresponding esters **34–36** in good yields with almost full inversion of stereochemistry. The reaction of alcohol **3** with 3,5-dinitrobenzoic acid in toluene gave ester **35** in a moderate level of enantioenrichment (er, 83 : 17). The reaction of 3,5-dinitrobenzoic acid, under the previous conditions (in THF at 65 °C) provided **35** in a higher level of enantioenrichment.^8^ In the reaction of **3** with 3-phenylpropionic acid, the enantioenrichment of ester **37** was not good (er, 78 : 22), but the reaction at low temperature (0 °C) gave an improved result (er, 90 : 10). Other nucleophiles such as phenol and phthalimide were applicable to reactions of chiral secondary alcohol **3** to provide the corresponding Mitsunobu products **38** and **39**, though the product yields were somewhat moderate. Reactions of other representative secondary alcohols **27–32** with 4-nitrobenzoic acid (**4**) readily provided the corresponding inversion products **40–45** in good yields. There was a slight loss of the optical purity of ester **42**, which was also observed in the typical Mitsunobu reaction with DEAD.6*a* However, the case of (–)-menthol (**33**) was still a limitation in the catalytic Mitsunobu reaction even though a highly acidic carboxylic acid was employed.^24^ For instance, the reaction of **33** with 4-nitrobenzoic acid gave inversion product **46** as a minor isomer. Fortunately, we found out that inversion product **47** was produced exclusively when the 2-methyl-6-nitrobenzoic acid was used as a nucleophile. These contrasting results could be attributed to the catalytic system. The reaction with a catalytic amount of the azo reagent maintains a low concentration of an intermediary alkoxyphosphonium salt. There would be an equilibrium process between the alkoxyphosphonium intermediate and an acyloxyphosphonium intermediate.^25^ If a subsequent reaction of the alkoxyphosphonium intermediate with a carboxylic acid to give an inversion product is slow, a retention product would increase *via* the equilibrium process to give the acyloxyphosphonium intermediate because the concentration of a free carboxylic acid is sufficiently higher than that of the alkoxyphosphonium intermediate in the catalytic system. 2-Methyl-6-nitrobenzoic acid has a sufficient acidity but is sterically hindered. Therefore, conversion of the alkoxyphosphonium intermediate into the corresponding acyloxyphosphonium intermediate would be an unfavourable process due to a steric factor of the carboxylic acid.^26^

### Mechanistic studies of the reaction using NMR spectroscopic methodologies

**Fig. 7:**
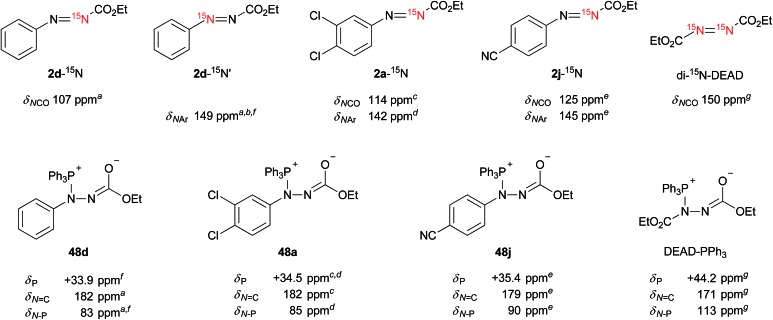
^15^N-labeled ethyl 2-arylazocarboxylates **2d**-^15^N, **2d**-^15^N′, **2a**-^15^N, **2j**-^15^N and di-^15^N-DEAD with ^15^N NMR chemical shifts (up), and ^31^P and ^15^N NMR data in CDCl_3_ of betaine intermediates produced through the reaction of the triphenylphosphine (10 equiv.) with azo compounds (bottom). The data were obtained from: ^*a*^**2d**-^15^N, ^*b*^**2d**-^15^N′, ^*c*^**2a**-^15^N, ^*d*^unlabeled **2a**, ^*e*^**2j**-^15^N, ^*f*^unlabeled **2d**, ^*g*^di-^15^N-DEAD.

Does the reaction of the ethyl 2-arylazocarboxylates with triphenylphosphine form Morrison–Brunn–Huisgen betaine intermediates^27^ like in the typical Mitsunobu reaction? Precedent mechanistic studies indicate the formation of betaine intermediates from azo reagents and phosphines. To obtain insights into the intermediates in the present reaction, we monitored the reactions of ethyl 2-arylazocarboxylates with triphenylphosphine using multinuclear (^1^H, ^13^C, ^31^P, ^15^N) 1D and 2D NMR spectroscopy. To assist an unambiguous structure elucidation and assignment of NMR parameters, two kinds of ^15^N-labeled ethyl 2-phenylazocarboxylates **2d**-^15^N and **2d**-^15^N′, ^15^N-labeled potent Mitsunobu reagents **2a**-^15^N, **2j**-^15^N and doubly ^15^N-labeled DEAD (di-^15^N-DEAD) were prepared and used in the study, along with some unlabeled analogues (Fig. 7).

The addition of triphenylphosphine (10 equiv.) into the solution of azo compounds in CDCl_3_ resulted in the appearance of low-field resonances in the ^31^P NMR spectra (**2d**: +33.9 ppm, **2a**: +34.5 ppm, **2j**: +35.4 ppm) that are supportive of the formation of betaine intermediates **48**. In light of the electron density of a nitrogen atom, these chemical shifts are roughly consistent with that of di-^15^N-DEAD (+44.2 ppm) and DEAD (+44.8 ppm).^27^

Although it is predicted that Michael-type addition of triphenylphosphine to ethyl 2-arylazocarboxylates (an attack to N2) takes place to form betaines,^28^ the formation of other intermediary structures should be considered. Unlike for the symmetric DEAD,^29^ the issue of the regiochemistry of the triphenylphosphine attack to ethyl 2-arylazocarboxylates is raised as a consequence of their non-symmetric nature and the potential electrophilicity of the azo benzene derivatives toward triphenylphosphine.^30^,^31^

^15^N NMR spectroscopy was sought as a probe for the *in situ* investigation of the regiochemistry. The formation of adducts formed between the triphenylphosphine and azo reagents was monitored using ^1^H, ^13^C, ^31^P, ^1^H–^1^H COSY, ^1^H–^13^C HSQC, ^1^H–^13^C HMBC, ^1^H–^31^P HMBC, ^1^H–^15^N HMBC experiments, as well as HRMS. The results are summarized in Fig. 7 (and Tables S3 and S4 in the ESI†). In ethyl 2-arylazocarboxylates (*e.g.***2a**, **d**, **j**), the *N*CO and *N*–Ar nitrogen atoms resonate in the regions of 107–125 ppm and 142–149 ppm, respectively. Upon the addition of triphenylphosphine, a large downfield shift of *N*CO to around 180 ppm, and a significant upfield shift of *N*Ar to approximately 83–90 ppm is observed for the betaine intermediates. The nitrogen atoms resonating in di-^15^N-DEAD at 150 ppm appear after the addition of triphenylphosphine at 113 ppm and 171 ppm.

**Scheme 1:**
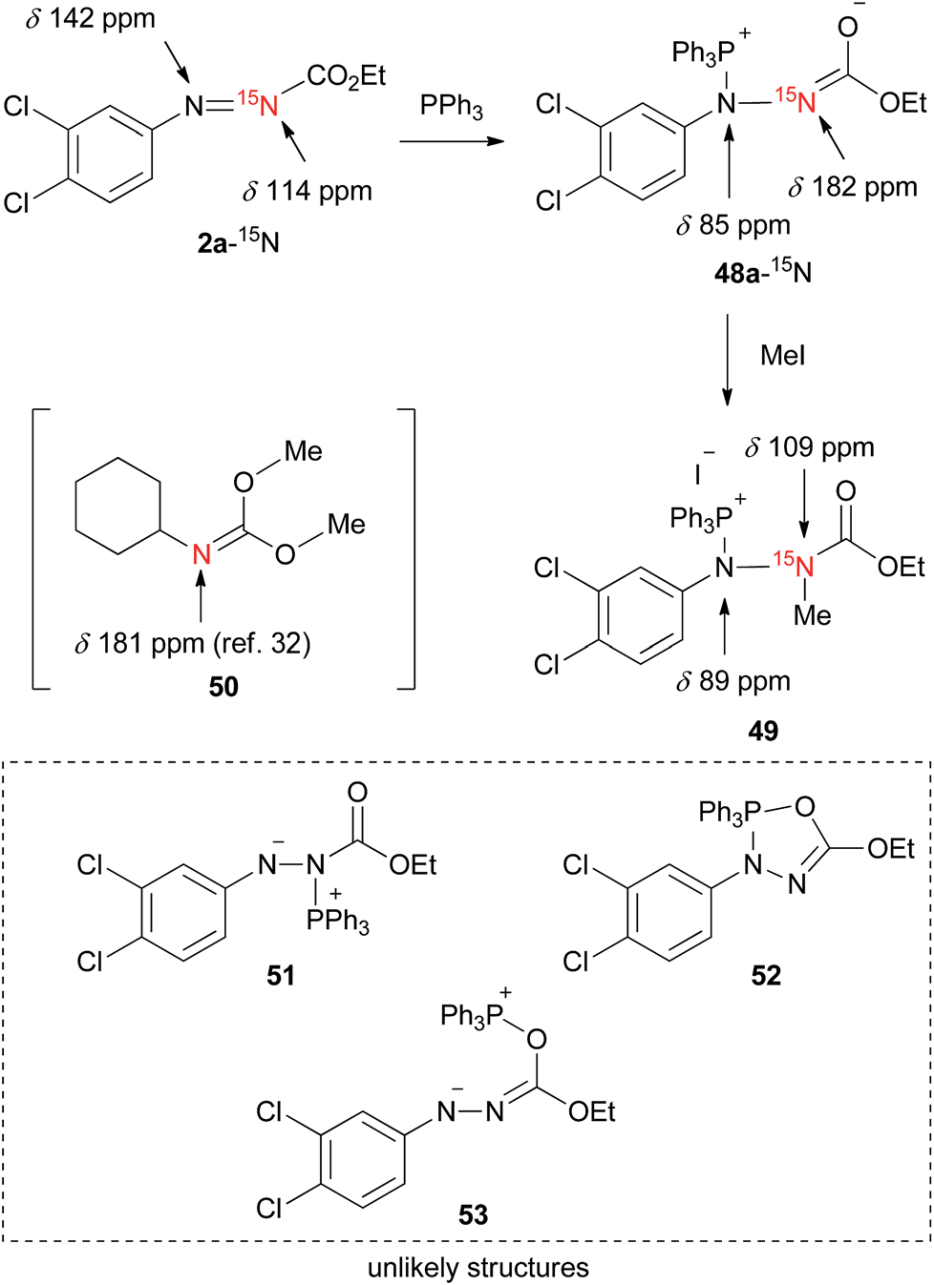
A trapping experiment of a betaine with iodomethane and the chemical shifts of the ^15^N NMR analysis.

Since, to the best of our knowledge, this is the first ^15^N NMR study of the intermediates formed in the Mitsunobu reaction, no direct comparison with the literature data is possible. Nevertheless, the downfield ^15^N resonances, which are common for all phosphine intermediates from Fig. 7, suggest carbonimidate structural fragments as they are consistent with the ^15^N NMR data of dimethyl cyclohexylcarbonimidate (**50** in Scheme 1, *δ*_N_ 181 ppm).^32^ Although this is reminiscent of a five-membered oxadiazophosphole ring structure (*e.g.*, *O*,*N*-phosphorane **52** in Scheme 1), the ^31^P NMR chemical shift of such an intermediate should possess a negative value.27*c* Perhaps, the *O*,*N*-phosphorane is formed as a transient intermediate,27*c* but formation of the betaine intermediate having a carbonimidate anion appears to be predominant in the reaction mixture.

To further support the structure of the intermediate we carried out a trapping experiment in which betaine **48a**, formed *in situ* from **2a**-^15^N and triphenylphosphine in CDCl_3_, was treated in an NMR tube with iodomethane. ^15^N NMR chemical shifts of the starting compounds and products are shown in Scheme 1. The reaction of **2a**-^15^N with triphenylphosphine followed by treatment with iodomethane readily afforded a methylated product holding a phosphine, as confirmed using ^1^H–^31^P HMBC. A correlation between the N–CH_3_ proton resonance with that of the C=O carbonyl in the ^1^H–^13^C HMBC spectrum, along with the absence of N–CH_3_ correlations with aromatic carbons, strongly suggested the formation of ^15^N-methylated phosphonium salt **49**. An upfield ^15^N NMR shift from 182 ppm (in **48a**-^15^N) to 109 ppm upon methylation additionally supports the structure of **49**. By repeating the trapping experiment with ^15^N-unlabeled **2a** in a preparative way, the corresponding phosphonium salt decomposed during chromatographic purification on silica gel into ethyl 2-(3,4-dichlorophenyl)-1-methylhydrazine-1-carboxylate (see the ESI†). Although the intermediates generated from the dialkyl azodicarboxylates and triphenylphosphine are generally presented in a form of a resonance structure with a negatively charged nitrogen atom and a C=O double bond, our NMR data suggest that the alternative with the sp^2^ hybridized nitrogen atom more accurately represents the true structure of the betaine (Fig. 7). This is also in agreement with oxygen being more electronegative than nitrogen.

Overall, the NMR experimental results support the formation of P–N betaines such as **48** in the Mitsunobu reaction using our reagents and indicate that other structures such as regioisomer **51** and P–O betaine **53** are unlikely. Formation of *O*,*N*-phosphorane **52** could not be ruled out but was not detected in our NMR analysis.

By treating butan-1-ol (10 equiv.) with triphenylphosphine (10 equiv.) and azo reagent **2a** (1 equiv.) in solvents like THF-*d*_8_, CD_3_CN, CDCl_3_, or toluene-*d*_8_, a ^31^P NMR resonance corresponding to di-*n*-butoxytriphenylphosphorane (**54**) appeared in the spectra between –56.0 ppm and –55.2 ppm (Table 4), which is consistent with the data for DEAD (–55.0 ppm in THF-*d*_8_).27*a* On the other hand, unlike for THF-*d*_8_, CD_3_CN and CDCl_3_, the resonance of betaine **48a** in toluene-*d*_8_ could not be detected. This suggests that an equilibrium toward betaine **48a** from **2a** is unfavorable but the reactivity of **48a** toward an alcohol is sufficiently high in toluene. Thus, the fate of the betaine generated from the ethyl 2-arylazocarboxylates and triphenylphosphine appears to be very similar to that from the typical Mitsunobu reaction using DEAD.

**Table 4:** Detection of a phosphorane intermediate **54** from butan-1-ol and betaine **48a** using ^31^P NMR analysis in different solvents

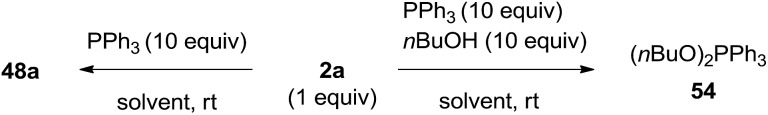			
Entry	Solvent	*δ*_P_ (ppm)	
		**54**	**48a**
1	THF-*d*_8_	–56.0	+21.1
2	CDCl_3_	–55.3	+34.5
3	CD_3_CN	–55.2	+33.7
4	Toluene-*d*_8_	–55.8	ND^*a*^

^*a*^Not detected.

### Thermal stability of the developed Mitsunobu reagents

When typical azo reagents such as DEAD are used, sufficient care is often required from the viewpoint of their thermal instability. Ethyl 2-(3,4-dichlorophenyl)hydrazinecarboxylate (**1a**), ethyl 2-(4-cyanophenyl)hydrazinecarboxylate (**1j**) and their azo forms **2a** and **2j** are stable crystalline solids under ambient conditions, and no decomposition of these compounds was observed after two months. Incidentally, when di(2-methoxyethyl) azodicarboxylate (DMEAD), that is a crystalline solid, was exposed to ambient conditions for two months, a partial but clear decomposition was observed using ^1^H NMR analysis. It is known, from differential scanning calorimetry (DSC), that DEAD, diisopropyl azodicarboxylate (DIAD) and di(2-methoxyethyl) azodicarboxylate (DMEAD) show a large exothermic peak at 210–250 °C, indicating exponential decomposition of these compounds.^33^

We investigated the thermal properties of ethyl 2-(3,4-dichlorophenyl)azocarboxylate (**2a**) and ethyl 2-(4-cyanophenyl)azocarboxylate (**2j**) using thermogravimetry-differential thermal analysis (TG-DTA). Interestingly, it indicated the absence of exothermic peaks, whereas endothermic peaks were observed at 191.3 °C (3,4-dichlorophenyl derivative **2a**, mp: 52.1 °C) and 225.7 °C (4-cyanophenyl derivative **2j**, mp: 55.4 °C) with a loss of weight of the samples. These peaks likely show boiling points of the azo compounds that are accompanied by some evaporation. A possibility of endothermic decomposition is unlikely because decomposition of azo compounds is generally exothermic. To eliminate the possibility of the endothermic decomposition, we representatively tested by heating **2a** in the solution-phase. A solution of **2a** in benzene-*d*_6_ was kept for 10 min at 200 °C in an autoclave and then analyzed using ^1^H NMR spectroscopy, which indicated no decomposition (see the ESI†). Similarly, TG-DTA of ethyl 2-(3,4-dichlorophenyl)hydrazinecarboxylate (**1a**, mp: 114.0 °C) and ethyl 2-(4-cyanophenyl)hydrazinecarboxylate (**1j**, mp: 138.1 °C) showed endothermic peaks with a loss of weight of the samples at 250.3 °C and 267.4 °C, though partial decomposition seems to occur around this temperature in the case of **1j**. Thus, we did not observe clear exponential decomposition of our azo and hydrazine compounds under the ambient pressure unlike in typical Mitsunobu reagents, though we did not test the thermal stability of these compounds at higher temperatures in a pressured vessel.^34^ Overall, the experimental results support that our Mitsunobu catalysts can be safely stored and used without special precautions.

## Conclusions

Ethyl 2-arylazocarboxylates can operate in the Mitsunobu reaction like typical Mitsunobu reagents such as diethyl azodicarboxylate (DEAD). The former, however, are recyclable using aerobic re-oxidation of the resultant ethyl 2-arylhydrazinecarboxylate with cheap and nontoxic iron phthalocyanine. This outstanding ability enables catalytic Mitsunobu reactions by using these reagents as organocatalysts. Our systematic study reveals that Mitsunobu activity of azo forms of these catalysts is compatible with an oxidation process of hydrazine forms. Two effective catalysts have been identified. Ethyl 2-(3,4-dichlorophenyl)hydrazinecarboxylate (**1a**) is suitable for catalytic Mitsunobu reactions with carboxylic acids, working best for the inversion of stereochemistry of secondary alcohols. Ethyl 2-(4-cyanophenyl)hydrazinecarboxylate (**1j**) provides excellent results in reactions with nucleophiles other than carboxylic acids, serving for the transformation of the hydroxyl groups of alcohols to other functional groups. Thus, the catalytic Mitsunobu reaction has been complemented by two potent reagents and strict optimization of the reaction conditions. The present catalytic protocol is comparable to the original Mitsunobu reaction in both, reactivity and scope. It is also noteworthy that these reagents are stable solids, and their thermal behavior is different from the typical Mitsunobu reagents. Our study has illustrated that serious limitations of the Mitsunobu reaction are avoidable using new reagents and improved procedures. We expect that the improved method will promote the use of the Mitsunobu reaction in practical synthesis.

## Supplementary Material

Supplementary information
