# Supplementary material for: Advances and mechanistic insight on the catalytic Mitsunobu reaction using recyclable azo reagents
Source: Chem Sci. 2016 Apr 13;7(8):5148–59. doi: 10.1039/c6sc00308g (PMC6020523; doi:10.1039/c6sc00308g)
Supplement: Supplementary file 1 [file SC-007-C6SC00308G-s001.pdf]

Electronic Supplementary Material (ESI) for Chemical Science.
This journal is © The Royal Society of Chemistry 2016

-SUPPLEMENTARY INFORMATION-

Advances and mechanistic insight on the catalytic Mitsunobu reaction using recyclable azo reagents

Daisuke Hirose,[a] Martin Gazvoda,[b] Janez Košmrlj*[b] and Tsuyoshi Taniguchi*[c]

[a]*Graduate School of Natural Science and Technology, Kanazawa University, Kakuma-machi, Kanazawa 920-1192, Japan*

[b]*Faculty of Chemistry and Chemical Technology, University of Ljubljana, Večna pot 113, SI-1000, Ljubljana, Slovenia*

[c]*School of Pharmaceutical Sciences, Institute of Medical, Pharmaceutical and Health Sciences, Kanazawa University, Kakuma-machi, Kanazawa 920-1192, Japan*

Table of Contents

General remarks....................S2

Experimental details
1. Synthetic procedures and analytical data for products....................S4
2. Kinetic studies for developed Mitsunobu reagents....................S19
3. NMR experiments of intermediates in the Mitsunobu reactions with ethyl 2-arylazocarboxylates....................S35
4. Thermal analysis of azo and hydrazine compounds....................S45
5. Photos of the typical experiment of the catalytic Mitsunobu reaction on 10 mmol scale....................S48

References....................S49

Copies of NMR spectra and HPLC data....................S50

General remarks

All reactions were performed in oven-dried glassware. All reagents purchased commercially were used without further purification unless otherwise noted. Iron phthalocyanine was purchased from Tokyo Chemical Industry Co., Ltd. Dehydrated THF, toluene and CH_2Cl_2 were purchased from Kanto Chemical Co., Inc. Other solvents were dried with activated molecular sieves. Molecular sieves were activated by heating with a heat gun (ISHIZAKI ELECTRIC MFG PJ-208A, ca. 450 °C) in vacuo (ca. 0.1 mmHg) for 5 min unless otherwise noted. Thin-layer chromatography (TLC) analysis was performed by illumination with a UV lamp (254 nm) or staining with PMA and heating. Silica gel column chromatography was carried out on silica gel 60N (Kanto Chemical Co., Inc., spherical, neutral, 40−50 μm). The chromatographic separation of enantiomers was performed using a JASCO PU-2080 Plus liquid chromatography equipped with Multi UV-Vis (JASCO MD-910). Melting points were recorded on Yanako melting point apparatus or on a Kofler micro hot stage and were uncorrected.

^{1}H and ^{13}C NMR spectra were recorded with JEOL JNM ECS400 (400 MHz and 100 MHz), JEOL JNM ECS500 (500 MHz and 125 MHz) and JEOL JNM ECA600 (600 MHz and 150 MHz) spectrometers at 293−295 K in Japan. ^{31}P NMR spectra were recorded with a JEOL JNM ECA600 (243 MHz) spectrometer at 298 K in Japan. Some ^{1}H, ^{31}P, ^{13}C and ^{15}N NMR spectra were recorded with a Bruker Avance III 500 MHz NMR (500 MHz, 202 MHz, 126 MHz and 51 MHz) instrument at 296 K in Slovenia. Proton spectra were referenced to TMS as an internal standard. Carbon chemical shifts were determined relative to the ^{13}C signal of $CDCl_3$ (77.0 ppm). ^{31}P NMR spectra were referenced to external 85% phosphoric acid (δ = 0 ppm). ^{15}N chemical shifts were extracted from ^{1}H–^{15}N *gs*-HMBC spectra (with 20 Hz digital resolution in the indirect dimension and the parameters adjusted for a long-range ^{1}H–^{15}N coupling constant of 5 Hz), determined with respect to external nitromethane and corrected to external ammonia by addition of 380.5 ppm. Assignments of some proton, carbon, phosphorous, and nitrogen resonances were performed by 2D NMR techniques (^{1}H−^{1}H *gs*-COSY, ^{1}H−^{13}C *gs*-HSQC, ^{1}H−^{13}C *gs*-HMBC, ^{1}H−^{31}P *gs*-HMBC, ^{1}H−^{15}N *gs*-HMBC). Coupling constants (J) are given in Hz. Multiplicities are indicated as follows: s (singlet), d (doublet), t (triplet), q (quartet), m (multiplet) or br (broadened).

IR spectra were recorded with a JASCO Fourier Transform IR-460 spectrometer or with a Perkin-Elmer Spectrum 100 (equipped with a Specac Golden Gate Diamond ATR as a solid sample support). Mass spectra were recorded on JEOL JMS-T100TD (direct analysis in real time, DART or electrospray ionization, ESI). A time-of-flight (TOF) mass spectrometer equipped with a double orthogonal electrospray source at atmospheric pressure ionization (ESI) coupled to an HPLC instrument was used for recording HRMS spectra. Optical rotations were measured on a JASCO P-1030 polarimeter. Absorption spectra were measured in a 0.1 mm quartz cell on a JASCO V-570 spectrophotometer. Thermogravimetry-Differential thermal analysis (TG-DTA) was performed on

EXSTAR TG/DTA-7300 (heating rate: 10.0 °C/min) under a nitrogen atmosphere. Differential scanning calorimetric (DSC) analysis was performed on Perkin Elmer Jade DSC (heating rate of 10.0 °C/min) under a nitrogen atmosphere.

Experimental details

1. Synthetic procedure and analytical data for products

Ethyl 2-arylhydrazinecarboxylates (**1**) and ethyl 2-arylazocarboxylates (**2**) except for ethyl 2-[(4-ethoxycarbonyl)phenyl]hydrazinecarboxylate (**1h**) and 2-[(4-ethoxycarbonyl)phenyl]azocarboxylate (**2h**) were known compounds and prepared according to previous reports.[1,2]

*Procedure for preparation of ethyl 2-[4-(ethoxycarbonyl)phenyl]hydrazinecarboxylate (**1h**):*

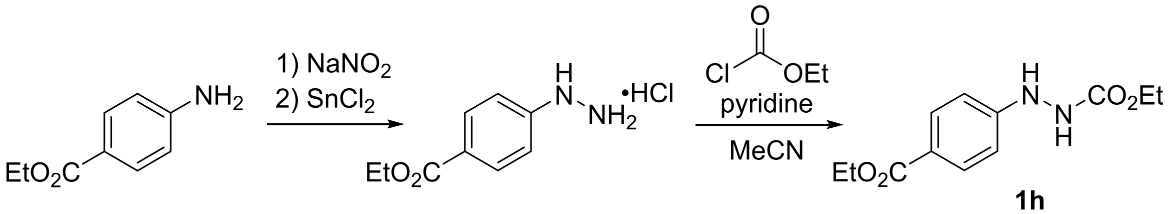

1h

A suspension of ethyl 4-aminobenzoate (1.65 g, 10 mmol) in concentrated HCl aq. (10 mL) was cooled to 0 °C. A solution of $NaNO_2$ (759 mg, 11 mmol) in water (10 mL) was added dropwise to the suspension. After the mixture was stirred for 10 min at 0 °C, a solution of $SnCl_2$ (5.88 g, 31 mmol) in concentrated HCl aq. (30 mL) was slowly added to the mixture. The mixture was stirred for 30 min at room temperature, and the formed precipitate was collected by filtration. The precipitate was washed with concentrated HCl aq. and dried under vacuum. To a solution of the obtained crude product and pyridine (3.96 g, 50 mmol) in CH_3CN (10 mL) was added dropwise ethyl chloroformate (1.19 g, 11 mmol) at 0 °C. The mixture was stirred for 30 min at 0 °C and allowed to warm to room temperature, and further stirred for 13 h. The reaction mixture was poured into water and extracted with ethyl acetate. The combined organic layers were washed with brine and dried with Na_2SO_4. After the solvent was evaporated, the crude product was washed by hexane and dried under vacuum to afford ethyl 2-[4-(ethoxycarbonyl)phenyl]hydrazinecarboxylate (**1h**: 1.45 g, 5.74 mmol, 57% yield) as a white solid. Recrystallization (hexane/EtOAc) of this product gave highly pure form as colorless needles.

Mp 81.5−82.0 °C. ^{1}H NMR (400 MHz, $CDCl_3$) δ 7.94 (2H, app. d, J = 9.2 Hz), 6.81 (2H, d, J = 8.7 Hz), 6.58 (1H, br-s), 6.04 (1H, br-s), 4.33 (2H, q, J = 7.2 Hz), 4.21 (2H, q, J = 7.0 Hz), 1.37 (3H, t, J = 7.1 Hz), 1.28 (3H, br-s); ^{13}C NMR (101 MHz, $CDCl_3$) δ 166.5, 156.8, 151.8, 131.3, 122.7, 111.8, 62.2, 60.5, 14.5, 14.4; IR (KBr, cm^{-1}) υ 2982, 1694, 1608, 1539, 1282, 1171, 1108; HRMS (DART+) (*m*/*z*): calcd for $C_{12}H_{17}N_2O_2$ ([M+H]$^+$): 253.1188, found: 253.1197.

*Procedure for preparation of ethyl 2-[4-(ethoxycarbonyl)phenyl]azocarboxylate (**2h**):*

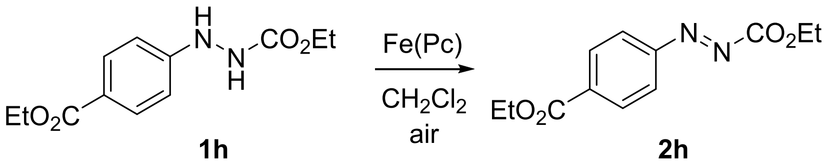

According to the reported procedure,[2] a mixture of ethyl 2-[4-(ethoxycarbonyl)phenyl]hydrazinecarboxylate (**1h**) (1.01 g, 4.0 mmol) and iron phthalocyanine (227 mg, 0.40 mmol) in CH_2Cl_2 (4.0 mL) was stirred for 20 h at room temperature under air. After the solvent was removed under reduced pressure, the residue was purified by flash chromatography (silica gel, hexane/EtOAc=5:1) to give ethyl 2-[4-(ethoxycarbonyl)phenyl]azocarboxylate (**2h**: 1.00 g, 4.0 mmol, 100% yield) as a red oil.

^{1}H NMR (400 MHz, $CDCl_3$) δ 8.21 (2H, app. d, J = 6.9 Hz), 7.97 (2H, app. d, J = 6.9 Hz), 4.54 (2H, q, J = 7.2 Hz), 4.42 (2H, q, J = 7.2 Hz), 1.48 (3H, t, J = 7.1 Hz), 1.43 (3H, t, J = 7.3 Hz); ^{13}C NMR (101 MHz, $CDCl_3$) δ 165.5, 161.9, 153.7, 134.6, 130.6, 123.4, 64.7, 61.5, 14.3, 14.1; IR (neat, cm^{-1}) υ 2985, 1760, 1722, 1277, 1246, 1188, 1106; HRMS (DART+) (*m*/*z*): calcd for $C_{12}H_{15}N_2O_2$ ($[M+H]^+$): 251.1032, found: 251.1025.

General procedure for catalytic Mitsunobu reactions:

A mixture of alcohol (1.0 mmol), nucleophile (1.1 mmol), triphenylphosphine (525 mg, 2.0 mmol), a hydrazine catalyst [ethyl 2-(3,4-dichlorophenyl)hydrazinecarboxylate (**1a**): 24.9 mg, 0.1 mmol or ethyl 2-(4-cyanophenyl)hydrazinecarboxylate (**1j**): 20.5 mg, 0.1 mmol], iron phthalocyanine (56.8 mg, 0.1 mmol) and powder of activated molecular sieves 5Å (500 mg) in toluene (250 μL) was irradiated for ca. 1 minute in an ultrasound bath and vigorously stirred under air atmosphere (balloon) at room temperature until disappearance of alcohol or triphenylphosphine. After the reaction mixture was filtrated through a filter paper or a pad of Celite®, the solvent was removed under reduced pressure. The crude material was purified by silica gel chromatography (*n*-hexane/EtOAc) to give the corresponding product.

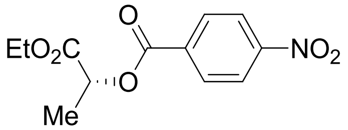

(*R*)-1-Ethoxycarbonylethyl 4-nitrobenzoate (5).[1] (–)-(*S*)-Ethyl lactate (**3**) (118 mg, 1.0 mmol, 99:1 er) and 4-nitrobenzoic acid (**3**) (184 mg, 1.1 mmol) were used as substrates. 12 h. 93% yield (249 mg, 0.93 mmol). 99:1 er [Fe(Pc) (1 mol%), PPh_3 (1.5 equiv): 18 h. 81% yield (215 mg, 0.81 mmol), 99:1 er]. White solid. Elute: Hex/AcOEt = 6:1. ^{1}H NMR (500 MHz, $CDCl_3$) δ 8.31 (2H, app. d, J = 9.5 Hz), 8.27 (2H, app. d, J = 9.5 Hz), 5.36 (1H, q, J = 7.0 Hz), 4.26 (2H, q, J = 7.0 Hz), 1.67 (3H, d, J = 7.0 Hz), 1.30 (3H, t, J = 7.0 Hz); ^{13}C NMR (125 MHz, $CDCl_3$) δ 170.2, 164.0, 150.7, 134.8, 130.9, 123.5, 69.9, 61.6, 16.9, 14.0; The enantiomeric ratio was determined by HPLC analysis

using a chiral column. Chiral HPLC: Daicel-Chiralpak AD-H 46 × 150 mm, multi UV-Vis detector (230 nm), room temperature eluent: (*n*-hexane/*i*-PrOH) 5:1, flow rate: 0.5 mL/min, retention time (min) 14.0 (*R* isomer), 17.5 (*S* isomer). $[\alpha]_D^{25} = -18.5$ (*c* 1.00, $CHCl_3$) [lit.[1] (*S*)-1-ethoxycarbonylethyl 4-nitrobenzoate (99:1 er), $[\alpha]_D^{30} = +13.6$ (*c* 1.00, $CHCl_3$)].

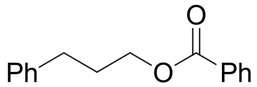

3-Phenylpropyl benzoate (11).[1] 3-Phenylpropanol (**6**) (136 mg, 1.0 mmol) and benzoic acid (134 mg, 1.1 mmol) were used as substrates. 18 h. 93% yield (222 mg, 0.93 mmol) [Fe(Pc) (1 mol%), PPh_3 (1.5 equiv): 36 h. 93% yield (223 mg, 0.93 mmol)]. Colorless oil. Elute: Hex/AcOEt = 30:1. ^{1}H NMR (600 MHz, $CDCl_3$) δ 8.04 (2H, d, *J* = 7.2 Hz), 7.55 (1H, app. t, *J* = 7.6 Hz), 7.43 (2H, app. t, *J* = 7.7 Hz), 7.29 (2H, app. t, *J* = 7.6 Hz), 7.22−7.18 (3H, m), 4.34 (2H, t, *J* = 6.6 Hz), 2.78 (2H, t, *J* = 7.6 Hz), 2.10 (2H, tt, *J* = 7.6, 6.6 Hz); ^{13}C NMR (150 MHz, $CDCl_3$) δ 166.5, 141.1, 132.8, 130.3, 129.5, 128.43, 128.39, 128.30, 126.0, 64.2, 32.2, 30.2.

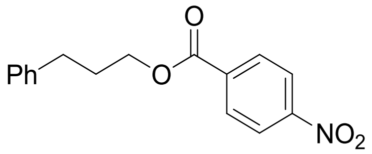

3-Phenylpropyl 4-nitrobenzoate (12).[1] 3-Phenylpropanol (**6**) (136 mg, 1.0 mmol) and 4-nitrobenzoic acid (**4**) (184 mg, 1.1 mmol) were used as substrates. 12 h. 91% yield (257 mg, 0.91 mmol) [**1a** (3 mol%), Fe(Pc) (3 mol%), PPh_3 (1.5 equiv): 23 h. 88% yield (250 mg, 0.88 mmol)]. Pale yellow oil. Elute: Hex/AcOEt = 20:1. ^{1}H NMR (600 MHz, $CDCl_3$) δ 8.28 (2H, app. d, *J* = 8.9 Hz), 8.16 (2H, app. d, *J* = 8.9 Hz), 7.30 (2H, app. t, *J* = 7.6 Hz), 7.22−7.20 (3H, m), 4.40 (2H, t, *J* = 6.6 Hz), 2.80 (2H, t, *J* = 7.2 Hz), 2.15 (2H, tt, *J* = 7.2, 6.6 Hz); ^{13}C NMR (150 MHz, $CDCl_3$) δ 164.6, 150.5, 140.9, 135.6, 130.6, 128.5, 128.4, 126.1, 123.5, 65.3, 32.3, 30.0.

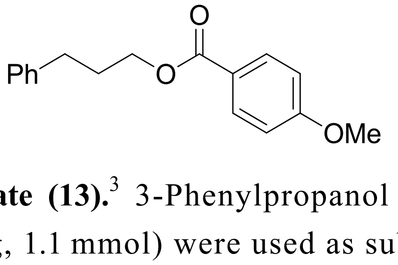

3-Phenylpropyl-4-methoxybenzoate (13).[3] 3-Phenylpropanol (**6**) (136 mg, 1.0 mmol) and 4-methoxybenzoic acid (167 mg, 1.1 mmol) were used as substrates. 18 h. 89% yield (238 mg, 0.89 mmol). Colorless oil. Elute: Hex/AcOEt = 20:1. ^{1}H NMR (600 MHz, $CDCl_3$) δ 7.99 (2H, app. d, *J* = 8.9 Hz), 7.29 (2H, app. t, *J* = 7.6 Hz), 7.22−7.18 (3H, m), 6.92 (2H, app. d, *J* = 8.9 Hz), 4.31 (2H, t, *J* = 6.6 Hz), 3.85 (3H, s), 2.78 (2H, t, *J* = 7.6 Hz), 2.09 (2H, tt, *J* = 7.6, 6.6 Hz); ^{13}C NMR (150 MHz, $CDCl_3$) δ 166.3, 163.3, 141.2, 131.5, 128.4, 126.0, 122.8, 113.6, 63.9, 55.4, 32.3, 30.3.

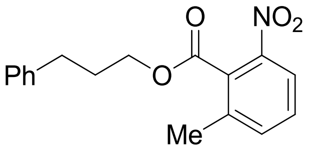

3-Phenylpropyl-2-methyl-6-nitrobenzoate (14). 3-Phenylpropanol (**6**) (136 mg, 1.0 mmol) and 2-methyl-6-nitrobenzoic acid (199 mg, 1.1 mmol) were used as substrates. 18 h. 89% yield (265 mg, 0.89 mmol). Pale yellow oil. Elute: Hex/AcOEt=10:1. ^{1}H NMR (600 MHz, $CDCl_3$) δ 7.99 (1H, d, J = 8.2 Hz), 7.54 (1H, d, J = 7.6 Hz), 7.47 (1H, t, J = 7.9 Hz), 7.29 (2H, app. t, J = 7.6 Hz), 7.22−7.19 (3H, m), 4.40 (2H, t, J = 6.6 Hz), 2.73 (2H, t, J = 7.8 Hz), 2.43 (3H, s), 2.08 (2H, tt, J = 7.8, 6.6 Hz); ^{13}C NMR (150 MHz, $CDCl_3$) δ 166.5, 146.2, 141.0, 137.5, 135.9, 129.66, 129.57, 128.4, 126.0, 121.8, 65.6, 32.1, 30.0, 19.1; IR (neat, cm^{-1}) υ 2928, 1734, 1539; HRMS (DART+) (*m*/*z*): $[M+NH_4]^+$ calcd for $C_{17}H_{21}N_2O_4$: 317.1501, found: 317.1504.

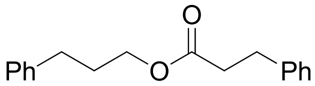

3-Phenylpropyl-3-phenylpropanoate (15).[3] 3-Phenylpropanol (**6**) (136 mg, 1.0 mmol) and 3-phenylpropionic acid (165 mg, 1.1 mmol) were used as substrates. 18 h. 91% yield (244 mg, 0.91 mmol). Colorless oil. Elute: Hex/AcOEt = 30:1. ^{1}H NMR (600 MHz, $CDCl_3$) δ 7.30−7.27 (4H, m), 7.22−7.18 (4H, m), 7.14 (2H, app. d, J = 7.6 Hz), 4.08 (2H, t, J = 6.6 Hz), 2.95 (2H, t, J = 7.7 Hz), 2.631 (2H, t, J = 7.2 Hz), 2.627 (2H, t, J = 7.8 Hz), 1.92 (2H, tt, J = 7.2, 6.6 Hz); ^{13}C NMR (150 MHz, $CDCl_3$) δ 172.9, 141.1, 140.5, 128.46, 128.38, 128.35, 128.24, 128.21, 125.9, 63.8, 35.8, 32.1, 30.9, 30.1.

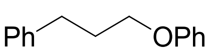

(3-Phenylpropoxy)benzene (9).[1] 3-Phenylpropanol (**6**) (136 mg, 1.0 mmol) and phenol (**7**) (104 mg, 1.1 mmol) were used as substrates. 18 h. 87% yield (184 mg, 0.87 mmol) [Fe(Pc) (1 mol%), PPh_3 (1.5 equiv): 36 h. 76% yield (161 mg, 0.76 mmol)]. Colorless oil. Elute: Hex/AcOEt = 20:1. ^{1}H NMR (600 MHz, $CDCl_3$) δ 7.30−7.25 (4H, m), 7.22−7.19 (3H, m), 6.93 (1H, app. t, J = 7.2 Hz), 6.90−6.89 (2H, m), 3.95 (2H, t, J = 6.6 Hz), 2.80 (2H, t, J = 7.7 Hz), 2.10 (2H, tt, J = 7.7, 6.6 Hz); ^{13}C NMR (150 MHz, $CDCl_3$) δ 159.0, 141.5, 129.4, 128.5, 128.4, 125.9, 120.5, 114.5, 66.7, 32.1, 30.8.

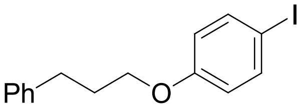

1-Iodo-4-(3-phenylpropoxy)benzene (16).[4] 3-Phenylpropanol (**6**) (136 mg, 1.0 mmol) and 4-iodophenol (242 mg, 1.1 mmol) were used as substrates. 24 h. 80% yield (270 mg, 0.80 mmol). Colorless oil. Elute: Hex/AcOEt = 20:1. ^{1}H NMR (600 MHz, $CDCl_3$) δ 7.53 (2H, app. d, J = 9.7, Hz), 7.28 (2H, app. t, J = 7.6 Hz), 7.20−7.18 (3H, m), 6.65 (2H, app. d, J = 9.6 Hz), 3.90 (2H, t,

J = 6.6 Hz), 2.79 (2H, t, J = 7.6 Hz), 2.08 (2H, tt, J = 7.6, 6.6 Hz); ^{13}C NMR (150 MHz, $CDCl_3$) δ 158.8, 141.3, 138.1, 128.5, 128.4, 126.0, 116.9, 82.5, 66.9, 32.0, 30.6.

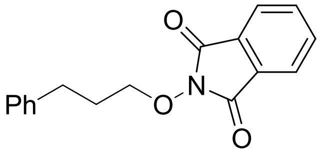

2-(3-Phenylpropoxy)isoindoline-1,3-dione (17).[1] 3-Phenylpropanol (**6**) (136 mg, 1.0 mmol) and *N*-hydroxyphthalimide (179 mg, 1.1 mmol) were used as substrates. 48 h. 85% yield (237 mg, 0.85 mmol) [0.5 M at 65 °C: 12 h. 85% yield (238 mg, 0.85 mmol)]. Pale yellow solid. Elute: Hex/AcOEt = 6:1. ^{1}H NMR (600 MHz, $CDCl_3$) δ 7.83−7.82 (m, 2H), 7.74−7.73 (m, 2H), 7.31−7.26 (4H, m), 7.19 (1H, app. t, J = 7.0 Hz), 4.22 (2H, t, J = 6.6 Hz), 2.87 (2H, t, J = 7.8 Hz), 2.10 (2H, tt, J = 7.8, 6.6 Hz); ^{13}C NMR (150 MHz, $CDCl_3$) δ 163.6, 141.1, 134.4, 128.9, 128.6, 128.4, 126.0, 123.4, 77.5, 31.7, 29.9.

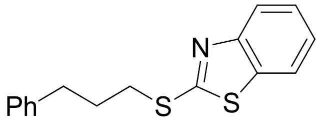

2-((3-Phenylpropyl)thio)benzo[*d*]thiazole (18). 3-Phenylpropanol (**6**) (136 mg, 1.0 mmol) and 2-mercaptobenzothiazole (184 mg, 1.1 mmol) were used as substrates. 24 h. 89% yield (253 mg, 0.89 mmol). Colorless oil. Elute: Hex/AcOEt=20:1. ^{1}H NMR (600 MHz, $CDCl_3$) δ 7.85 (1H, app. d, J = 7.9 Hz), 7.73 (1H, app. d, J = 7.9 Hz), 7.39 (1H, app. t, J = 7.7 Hz), 7.30−7.26 (3H, m), 7.25−7.18 (3H, m), 3.33 (2H, t, J = 7.2 Hz), 2.80 (2H, t, J = 7.2 Hz), 2.15 (2H, quint, J = 7.2 Hz); ^{13}C NMR (150 MHz, $CDCl_3$) δ 166.9, 153.3, 140.8, 135.1, 128.46, 128.42, 126.1, 125.9, 124.1, 121.4, 120.9, 34.6, 32.8, 30.7; IR (neat, cm^{-1}) υ 2928, 1456, 1423; HRMS (DART+) (*m*/*z*): $[M+H]^+$ calcd for $C_{16}H_{16}NS_2$: 286.0724, found: 286.0720.

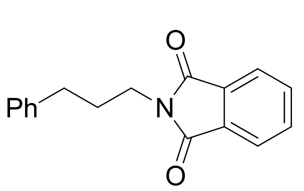

2-(3-Phenylpropyl)isoindole-1,3-dione (10).[1] 3-Phenylpropanol (**6**) (136 mg, 1.0 mmol) and phthalimide (**8**) (162 mg, 1.1 mmol) were used as substrates. 12 h (0.5 M at 65 °C). 89% yield (234 mg, 0.89 mmol) [General procedure: 48 h. 84% yield (221 mg, 0.84 mmol)]. Pale yellow oil, Elute: Hex/AcOEt = 10:1. ^{1}H NMR (500 MHz, $CDCl_3$) δ 7.82−7.81 (m, 2H), 7.70−7.68 (m, 2H), 7.24 (2H, app. t, J = 7.4 Hz), 7.19 (2H, app. d, J = 6.9 Hz), 7.14 (1H, app. t, J = 7.2 Hz), 3.74 (2H, t, J = 7.5 Hz), 2.68 (2H, t, J = 8.0 Hz), 2.03 (2H, tt, J = 8.0, 7.5 Hz); ^{13}C NMR (125 MHz, $CDCl_3$) δ 168.3, 141.0, 133.8, 132.0, 128.3, 128.2, 125.9, 123.1, 37.7, 33.1, 29.8.

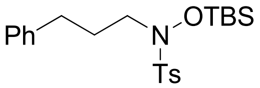

3-Phenylpropyl-*O*-(*tert*-butyldimethylsilyl)-*N*-tosylhydroxylamine (19).[5] 3-Phenylpropanol (**6**) (136 mg, 1.0 mmol) and *O*-(*tert*-butyldimethylsilyl)-*N*-tosylhydroxylamine (332 mg, 1.1 mmol) were used as substrates. 60 h. 87% yield (364 mg, 0.87 mmol). Colorless oil. Elute: Hex/AcOEt = 20:1. ^{1}H NMR (600 MHz, $CDCl_3$) *δ* 7.69 (2H, app. d, *J* = 8.2 Hz), 7.32 (2H, app. d, *J* = 8.2 Hz), 7.27 (2H, app. t, *J* = 7.6 Hz), 7.18 (1H, app. t, *J* = 7.9 Hz), 7.14−7.13 (2H, m), 2.94 (2H, t, *J* = 7.0 Hz), 2.62 (2H, t, *J* = 7.8 Hz), 2.44 (3H, s), 1.89 (2H, tt, *J* = 7.8, 7.2 Hz), 0.91 (9H, s), 0.28 (6H, s); ^{13}C NMR (150 MHz, $CDCl_3$) *δ* 144.5, 141.1, 129.9, 129.8, 129.2, 128.39, 128.37, 126.0, 55.4, 33.1, 28.5, 26.0, 21.6, 18.2, −4.3.

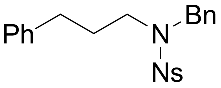

***N*-Benzyl-4-nitro-*N*-(3-phenylpropyl)benzenesulfonamide (20).**[1] 3-Phenylpropanol (**6**) (136 mg, 1.0 mmol) and *N*-benzyl-2-nitrobenzenesulfonylamide (321 mg, 1.1 mmol) were used as substrates. 12 h (0.5 M at 65 ˚C). 90% yield (367 mg, 0.90 mmol) [General procedure: 48 h. 60% yield (245 mg, 0.60 mmol)]. Pale yellow oil. Elute: Hex/AcOEt = 5:1. ^{1}H NMR (600 MHz, $CDCl_3$) *δ* 7.91 (d, *J* = 7.9 Hz, 1H), 7.68−7.58 (m, 3H), 7.30−7.15 (m, 8H), 6.98 (d, *J* = 7.6 Hz, 2H), 4.51 (s, 2H), 3.25 (t, *J* = 7.6 Hz, 2H), 2.43 (t, *J* = 7.8 Hz, 2H), 1.70 (tt, *J* = 7.8, 7.6 Hz, 2H); ^{13}C NMR (150 MHz, $CDCl_3$) *δ* 147.9, 140.9, 135.6, 133.7, 133.4, 131.6, 130.9, 128.7, 128.4, 128.3, 128.2, 127.9, 126.0, 124.2, 51.3, 46.7, 32.6, 29.0. [Note: One peak (46.7 ppm) has been inadvertently omitted from the list of the ^{13}C NMR data in the previous report[1]]. HRMS (DART+) (*m*/*z*): $[M+H]^+$ calcd for $C_{22}H_{23}N_2O_4S$: 411.1379, found: 411.1369. [Note: Since the HRMS data was incorrect in the previous report (the value based on $[M{-}Ns]^+$ was inadvertently described),[1] reanalysis of HRMS was conducted]

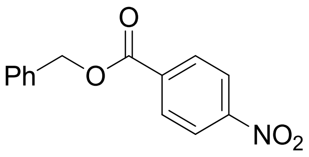

Benzyl-4-nitrobenzoate (21).[3] Benzyl alcohol (108 mg, 1.0 mmol) and 4-nitrobenzoic acid (**4**) (184 mg, 1.1 mmol) were used as substrates. 12 h. 90% yield (230 mg, 0.90 mmol). Pale yellow oil. Elute: Hex/AcOEt = 10:1. ^{1}H NMR (600 MHz, $CDCl_3$) *δ* 8.28 (2H, app. d, *J* = 9.0 Hz), 8.24 (2H, app. d, *J* = 9.0 Hz), 7.46 (2H, d, *J* = 7.2 Hz), 7.43−7.38 (3H, m), 5.41 (2H, s); ^{13}C NMR (150 MHz, $CDCl_3$) *δ* 164.5, 150.6, 135.48, 135.22, 130.8, 128.73, 128.65, 128.44, 123.5, 67.6.

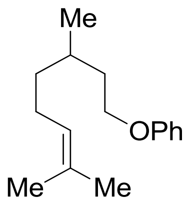

((3,7-Dimethyloct-6-en-1-yl)oxy)benzene (22). (±)-Citronellol (156 mg, 1.0 mmol) and phenol (**7**) (104 mg, 1.1 mmol) were used as substrates. 24 h. 77% yield (177 mg, 0.77 mmol). Colorless oil. Elute: Hex/AcOEt = 15:1. ^{1}H NMR (600 MHz, $CDCl_3$) δ 7.28−7.25 (2H, m), 6.94−6.89 (3H, m), 5.11 (1H, app. t, J = 7.2 Hz), 4.02−3.95 (2H, m), 2.07−1.95 (2H, m), 1.87−1.81 (1H, m), 1.71−1.68 (1H, m), 1.69 (3H, s), 1.62−1.58 (1H, m), 1.61 (3H, s), 1.41−1.38 (1H, m), 1.25−1.19 (1H, m), 0.95 (3H, d, J = 6.5 Hz); ^{13}C NMR (150 MHz, $CDCl_3$) δ 159.1, 131.3, 129.4, 124.7, 120.4, 114.5, 66.1, 37.1, 36.1, 29.5, 25.7, 25.4, 19.5, 17.7; IR (neat, cm^{-1}) υ 2923, 1600, 1498. 1243; HRMS (DART+) (*m*/*z*): [M+H]$^{+}$ calcd for $C_{16}H_{25}O$: 233.1905, found: 233.1894.

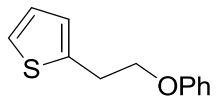

2-(2-Phenoxyethyl)thiophene (23). 3-Thiopheneethanol (128 mg, 1.0 mmol) and phenol (**7**) (104 mg, 1.1 mmol) were used as substrates. 18 h. 82% yield (167 mg, 0.82 mmol). colorless oil. Elute: Hex/AcOEt = 30:1. ^{1}H NMR (600 MHz, $CDCl_3$) δ 7.29−7.26 (2H, m), 7.15 (1H, dd, J = 5.2, 1.4 Hz), 6.96−6.91 (5H, m), 4.18 (2H, t, J = 6.7 Hz), 3.30 (2H, app. t, J = 6.9 Hz); ^{13}C NMR (150 MHz, $CDCl_3$) δ 158.6, 140.4, 129.5, 126.8, 125.5, 123.9, 120.9, 114.6, 68.3, 30.0; IR (neat, cm^{-1}) υ 2924, 1596, 1497, 1243; HRMS (DART+) (*m*/*z*): [M+H]$^{+}$ calcd for $C_{12}H_{13}OS$: 205.0687, found: 205.0689.

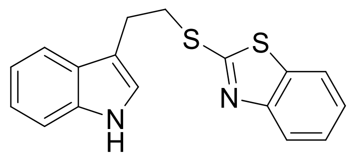

2-((2-(1H-Indol-3-yl)ethyl)thio)benzo[d]thiazole (24).[6] 3-Indoleethanol (161 mg, 1.0 mmol) and 2-mercaptobenzothiazole (184 mg, 1.1 mmol) were used as substrates. 24 h. 96% yield (296 mg, 0.96 mmol). White solid. Elute: Hex/AcOEt = 10:1. ^{1}H NMR (600 MHz, $CDCl_3$) δ 8.07 (1H, br s), 7.90 (1H, d, J = 8.6 Hz), 7.75 (2H, d, J = 7.6 Hz), 7.41 (1H, app. t, J = 7.7 Hz), 7.35 (1H, d, J = 7.9 Hz), 7.29 (1H, app. t, J = 7.6 Hz), 7.21 (1H, app. t, J = 7.6, Hz), 7.17−7.15 (1H, m), 7.07 (1H, d, J = 2.4 Hz), 3.66 (2H, t, J = 7.8 Hz), 3.30 (2H, t, J = 7.8 Hz); ^{13}C NMR (150 MHz, $CDCl_3$) δ 167.1, 153.3, 136.3, 135.2, 127.1, 126.0, 124.1, 122.2, 122.1, 121.4, 120.9, 119.5, 118.9, 114.1, 111.2, 34.2, 25.5.

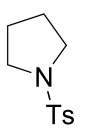

***N*-(*p*-Toluenesulfonyl)pyrrolidine (25).**[7] *N*-(4-Hydroxybutyl)-toluenesulfonamide[8] (243 mg, 1.0 mmol) was used as substrates in 0.5 M. 18 h. 94% yield (211 mg, 0.94 mmol) [Fe(Pc) (1 mol%), PPh_3 (1.5 equiv): 36 h. 87% yield (196 mg, 0.87 mmol)]. White solid. Elute: Hex/AcOEt = 5:1. ^{1}H NMR (600 MHz, $CDCl_3$) δ 7.72 (2H, app. d, J = 8.2 Hz), 7.32 (2H, app. d, J = 8.2 Hz), 3.24−3.22 (4H, m), 2.43 (3H, s), 1.76−1.74 (4H, m); ^{13}C NMR (150 MHz, $CDCl_3$) δ 143.3, 133.9, 129.6, 127.5, 47.9, 25.2, 21.5.

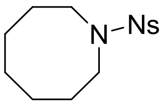

1-(2-Nitrobenzenesulfonyl)azocane (26).[9] *N*-(7-Hydroxyheptyl)-2-nitrobenzenesulfonamide[9] (316 mg, 1.0 mmol) was used as substrates in 0.05 M. 36 h. 40% yield (118 mg, 0.40 mmol). White solid. Elute: Hex/AcOEt = 5:1. ^{1}H NMR (500 MHz, $CDCl_3$) δ 7.93−7.92 (1H, m), 7.68−7.66 (2H, m), 7.59−7.58 (1H, m), 3.33 (4H, t, J = 6.0 Hz), 1.79−1.77 (4H, m), 1.67−1.65 (6H, m); ^{13}C NMR (125 MHz, $CDCl_3$) δ 148.3, 133.2, 132.5, 131.3, 130.5, 123.9, 49.2, 27.7, 26.5, 24.7.

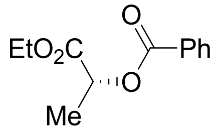

(*R*)-1-Ethoxycarbonylethyl benzoate (34).[10] (–)-(*S*)-Ethyl lactate (**3**) (118 mg, 1.0 mmol) and benzoic acid (134 mg, 1.1 mmol) were used as substrates. 36 h. 84% yield (186 mg, 0.84 mmol) 99:1 er. Pale yellow solid. Elute: Hex/AcOEt=20:1. ^{1}H NMR (600 MHz, $CDCl_3$) δ 8.09 (2H, app. d, J = 8.2 Hz), 7.58 (1H, app. t, J = 7.4 Hz), 7.45 (2H, app. t, J = 7.7 Hz), 5.31 (1H, q, J = 7.0 Hz), 4.23 (2H, q, J = 7.0 Hz), 1.63 (3H, d, J = 7.0 Hz), 1.28 (3H, t, J = 7.0 Hz); ^{13}C NMR (150 MHz, $CDCl_3$) δ 170.8, 165.9, 133.2, 129.8, 129.5, 128.3, 69.1, 61.3, 17.0, 14.1; The enantiomeric ratio was determined by HPLC analysis using a chiral column. Chiral HPLC: Daicel-Chiralpak OJ-H 46 × 150 mm, multi UV-Vis detector (230 nm), room temperature eluent: (*n*-hexane /*i*-PrOH) 99.5:0.5, flow rate: 1.0 mL/min, retention time (min) 22.8 (*R* isomer), 25.1 (*S* isomer). $[\alpha]_D^{25}$ = –12.6 (*c* 1.00, $CHCl_3$) [lit.[11] (*R*)-1-Ethoxycarbonylethyl benzoate; $[\alpha]_D^{25}$ = –13.8 (*c* 1.00, $CHCl_3$)], [Authentic sample (retention): (*S*)-1-Ethoxycarbonylethyl benzoate (99:1 er), $[\alpha]_D^{25}$ = +16.1 (*c* 1.00, $CHCl_3$)].

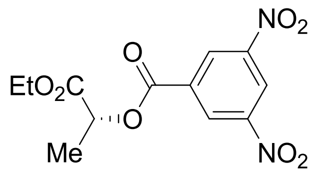

(*R*)-1-Ethoxycarbonylethyl 3,5-dinitrobenzoate (35).[1] (–)-(*S*)-Ethyl lactate (**3**) (118 mg, 1.0 mmol) and 3,5-dinitrobenzoic acid (233 mg, 1.1 mmol) were used as substrates. 18 h. 89% yield (278 mg, 0.89 mmol) 87:13 er. Pale yellow solid. Elute: Hex/AcOEt = 6:1. ^{1}H NMR (500 MHz, $CDCl_3$) δ 9.26 (1H, t, J = 2.0 Hz), 9.21 (2H, d, J = 2.3 Hz), 5.42 (1H, q, J = 7.0 Hz), 4.27 (2H, q, J = 7.2 Hz), 1.73 (3H, d, J = 7.5 Hz), 1.32 (3H, t, J = 7.2 Hz); ^{13}C NMR (125 MHz, $CDCl_3$) δ 169.7, 162.0, 148.6, 133.2, 129.6, 122.7, 70.8, 61.9, 16.9, 14.1; The enantiomeric ratio was determined by HPLC analysis using a chiral column. Chiral HPLC: Daicel-Chiralpak OJ-H 46 × 150 mm, multi UV-Vis detector (210 nm), room temperature eluent: (*n*-hexane /*i*-PrOH) 1:5, flow rate: 0.25 mL/min, retention time (min) 51.0 (*S* isomer), 61.1 (*R* isomer). $[\alpha]_D^{25}$ = –3.5 (*c* 1.00, $CHCl_3$) [lit.[1] (*S*)-1-ethoxycarbonylethyl 3,5-dinitrobenzoate (>99:1 er), $[\alpha]_D^{22}$ = +8.8 (*c* 1.00, $CHCl_3$)].
For data of the reaction at 65 °C in THF (0.5 M), see the Supporting Information in ref. 1.

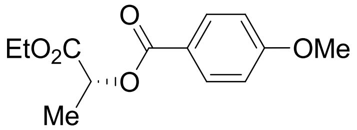

(*R*)-1-Ethoxycarbonylethyl 4-methoxybenzoate (36). (–)-(*S*)-Ethyl lactate (**3**) (118 mg, 1.0 mmol) and 4-methoxybenzoic acid (167 mg, 1.1 mmol) were used as substrates. 36 h. 79% yield (197 mg, 0.79 mmol) 99:1 er. Colorless oil. Elute: Hex/AcOEt = 10:1. ^{1}H NMR (600 MHz, $CDCl_3$) δ 8.04 (2H, app. d, J = 8.9 Hz), 6.93 (2H, app. d, J = 8.9 Hz), 5.28 (1H, q, J = 6.6 Hz), 4.23 (2H, q, J = 7.2 Hz), 3.86 (3H, s), 1.61 (3H, d, J = 6.6 Hz), 1.28 (3H, t, J = 7.2 Hz); ^{13}C NMR (150 MHz, $CDCl_3$) δ 171.0, 165.6, 163.6, 131.9, 121.8, 113.6, 68.9, 61.3, 55.4, 17.1, 14.1; IR ($CHCl_3$, cm^{-1}) υ 2987, 1717, 1608, 1513, 1457, 1259; HRMS (DART+) (*m*/*z*): $[M+H]^+$ calcd for $C_{13}H_{17}O_5$: 253.1076, found: 253.1076. The enantiomeric ratio was determined by HPLC analysis using a chiral column. Chiral HPLC: Daicel-Chiralpak OD-H 46 × 150 mm, multi UV-Vis detector (250 nm), room temperature eluent: (*n*-hexane /*i*-PrOH) 99.5:0.5, flow rate: 1.0 mL/min, retention time (min) 30.2 (*S* isomer), 34.0 (*R* isomer). $[\alpha]_D^{25}$ = –29.4 (*c* 1.00, $CHCl_3$) [Authentic sample (retention): (*S*)-1-Ethoxycarbonylethyl 4-methoxybenzoate (99:1 er), $[\alpha]_D^{25}$ = +32.6 (*c* 1.00, $CHCl_3$)].

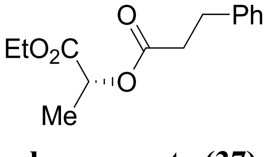

(*R*)-1-Ethoxy-1-oxopropan-2-yl 3-phenylpropanoate (37). (–)-(*S*)-Ethyl lactate (**3**) (118 mg, 1.0 mmol) and 3-phenylpropionic acid (165 mg, 1.1 mmol) were used as substrates, and the reaction was performed at 0 °C. 96 h. 61% yield (185 mg, 0.75 mmol) 90:10 er [General

procedure: 42 h. 75% yield (185 mg, 0.75 mmol), 78:22 er]. Colorless oil. Elute: Hex/AcOEt = 10:1. ^{1}H NMR (600 MHz, $CDCl_3$) δ 7.29 (2H, app. t, J = 7.4 Hz), 7.22−7.19 (3H, m), 5.07 (1H, q, J = 7.2 Hz), 4.19 (2H, q, J = 7.2 Hz), 2.98 (2H, t, J = 7.4 Hz), 2.77−2.67 (2H, m), 1.47 (3H, d, J = 7.2 Hz), 1.26 (3H, t, J = 7.2 Hz).; ^{13}C NMR (150 MHz, $CDCl_3$) δ 172.2, 170.8, 140.3, 128.4, 128.3, 126.2, 68.6, 61.3, 35.5, 30.7, 16.9, 14.0; IR (neat, cm^{-1}) υ 2987, 1734, 1455, 1374; HRMS (DART+) (*m*/*z*): $[M+H]^+$ calcd for $C_{14}H_{19}O_4$: 251.1283, found: 251.1284. The enantiomeric ratio was determined by HPLC analysis using a chiral column. Chiral HPLC: Daicel-Chiralpak OD-H 46 × 150 mm, multi UV-Vis detector (210 nm), room temperature eluent: (*n*-hexane/*i*-PrOH) 99.5:0.5, flow rate: 1.0 mL/min, retention time (min) 32.8 (*S* isomer), 41.1 (*R* isomer). $[\alpha]_D^{25}$ = +26.6 (*c* 1.00, $CHCl_3$) [Authentic sample (retention): (*S*)-1-Ethoxy-1-oxopropan-2-yl 3-phenylpropanoate (99:1 er), $[\alpha]_D^{25}$ = –29.5 (*c* 1.00, $CHCl_3$)].

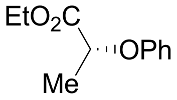

Ethyl (*R*)-2-phenoxypropanoate (38).[12] (–)-(*S*)-Ethyl lactate (**3**) (118 mg, 1.0 mmol) and phenol (**7**) (104 mg, 1.1 mmol) were used as substrates. 48 h. 63% yield (121 mg, 0.63 mmol) 99:1 er. Colorless oil. Elute: Hex/AcOEt = 8:1. ^{1}H NMR (600 MHz, $CDCl_3$) δ 7.28−7.25 (2H, m), 6.97 (1H, app. t, J = 7.4 Hz), 6.88 (2H, app. d, J = 7.9 Hz), 4.74 (1H, q, J = 7.2 Hz), 4.22 (2H, q, J = 7.2 Hz), 1.62 (3H, d, J = 6.6 Hz), 1.24 (3H, t, J = 7.2 Hz); ^{13}C NMR (150 MHz, $CDCl_3$) δ 172.2, 157.5, 129.5, 121.5, 115.0, 72.5, 61.2, 18.5, 14.1; The enantiomeric ratio was determined by HPLC analysis using a chiral column. Chiral HPLC: Daicel-Chiralpak OD-H 46 × 150 mm, multi UV-Vis detector (275 nm), room temperature eluent: (*n*-hexane/*i*-PrOH) 90:10, flow rate: 1.0 mL/min, retention time (min) 4.7 (*S* isomer), 7.3 (*R* isomer). $[\alpha]_D^{25}$ = +50.9 (*c* 1.00, MeOH) [lit.[12] ethyl (*R*)-2-phenoxypropanoate, $[\alpha]_D^{18}$ = +47.2 (*c* 0.50, MeOH)].

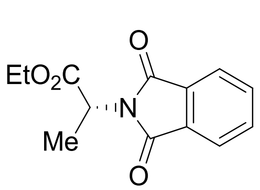

Ethyl (*R*)-2-(1,3-dioxoisoindolin-2-yl)propanoate (39).[13] (–)-(*S*)-Ethyl lactate (**3**) (118 mg, 1.0 mmol) and phthalimide (**8**) (162 mg, 1.1 mmol) were used as substrates in 0.5 M. 48 h. 49% yield (121 mg, 0.49 mmol) 99:1 er. white solid. Elute: Hex/AcOEt=10:1. ^{1}H NMR (500 MHz, $CDCl_3$) δ 7.87 (2H, app. dd, J = 5.0, 3.5 Hz), 7.75 (2H, app. dd, J = 5.0, 3.5 Hz), 4.97 (1H, q, J = 7.5 Hz), 4.26−4.17 (2H, m), 1.70 (3H, d, J = 6.5 Hz), 1.24 (3H, t, J = 7.5 Hz); ^{13}C NMR (125 MHz, $CDCl_3$) δ 169.7, 167.4, 134.1, 131.9, 123.5, 61.8, 47.5, 15.2, 14.1; The enantiomeric ratio was determined by HPLC analysis using a chiral column. Chiral HPLC: Daicel-Chiralpak OD-H 46 × 150

mm, multi UV-Vis detector (210 nm), room temperature eluent: (*n*-hexane/*i*-PrOH) 99.5:0.5, flow rate: 0.5 mL/min, retention time (min) 38.6 (*S* isomer), 40.9 (*R* isomer). $[\alpha]_D^{25}$ = +18.4 (*c* 1.00, MeOH) [lit.[13] ethyl (*R*)-2-(1,3-dioxoisoindolin-2-yl)propanoate, $[\alpha]_D^{25}$ = +18.2 (*c* 1.00, MeOH)].

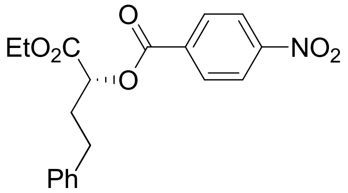

(*S*)-1-Ethoxy-1-oxo-4-phenylbutan-2-yl 4-nitrobenzoate (40). Ethyl (–)-(*R*)-2-hydroxy-4-phenylbutyrate (**27**) (208 mg, 1.0 mmol, 99:1 er) and 4-nitrobenzoic acid (**4**) (184 mg, 1.1 mmol) were used as substrates. 24 h. 93% yield (332 mg, 0.93 mmol) 99:1 er. Pale yellow oil. Elute: Hex/AcOEt = 15:1. ^{1}H NMR (600 MHz, $CDCl_3$) δ 8.29 (2H, app. d, *J* = 8.9 Hz), 8.17 (2H, app. d, *J* = 8.9 Hz), 7.31−7.29 (2H, m), 7.23−7.20 (3H, m), 5.28 (1H, t, *J* = 6.6 Hz), 4.24 (2H, q, *J* = 7.2 Hz), 2.85 (2H, t, *J* = 7.8 Hz), 2.36 (2H, dt, *J* = 7.8, 6.6 Hz), 1.29 (3H, t, *J* = 7.2 Hz); ^{13}C NMR (150 MHz, $CDCl_3$) δ 169.5, 164.1, 150.7, 140.2, 134.8, 131.0, 128.6, 128.4, 126.3, 123.5, 73.1, 61.7, 32.5, 31.5, 14.1; IR (neat, cm^{-1}) υ 2983, 1733, 1530, 1276; HRMS (DART+) (*m*/*z*): calcd for $[M+NH_4]^+$ $C_{15}H_{21}N_2O_6$: 375.1556, found: 375.1563; The enantiomeric ratio was determined by HPLC analysis using a chiral column. Chiral HPLC: Daicel-Chiralpak OJ-H 46 × 150 mm, multi UV-Vis detector (210 nm), room temperature eluent: (*n*-hexane/*i*-PrOH) 5:1, flow rate: 1.0 mL/min, retention time (min) 52.4 (*S* isomer), 25.7 (*R* isomer). $[\alpha]_D^{25}$ = –16.5 (*c* 1.00, $CHCl_3$) [Authentic sample (retention): (*R*)-1-ethoxy-1-oxo-4-phenylbutan-2-yl 4-nitrobenzoate (99:1 er), $[\alpha]_D^{25}$ = +19.6 (*c* 1.00, $CHCl_3$)].

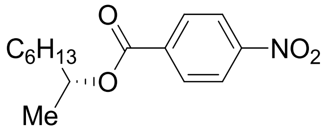

(*R*)-2-Octyl 4-nitrobenzoate (41).[14] (+)-(*S*)-2-Octanol (**28**) (130 mg, 1.0 mmol, 99:1 er) and 4-nitrobenzoic acid (**4**) (184 mg, 1.1 mmol) were used as substrates. 12 h. 91% yield (252 mg, 0.91 mmol) 99:1 er. Pale yellow oil. Elute: Hex/AcOEt = 30:1. ^{1}H NMR (600 MHz, $CDCl_3$) δ 8.29 (2H, app. d, *J* = 8.9 Hz), 8.21 (2H, app. d, *J* = 8.9 Hz), 5.20−5.18 (1H, m), 1.79−1.74 (1H, m), 1.67−1.60 (1H, m), 1.42−1.26 (8H, m), 1.38 (3H, d, *J* = 7.2 Hz), 0.88 (3H, t, *J* = 7.2 Hz); ^{13}C NMR (150 MHz, $CDCl_3$) δ 164.3, 150.4, 136.3, 130.6, 123.4, 73.1, 35.9, 31.7, 29.1, 25.4, 22.5, 20.0, 14.0; The enantiomeric ratio was determined by HPLC analysis using a chiral column. Chiral HPLC: Daicel-Chiralpak OJ-H 46 × 150 mm, multi UV-Vis detector (230 nm), room temperature eluent: (*n*-hexane/*i*-PrOH) 99.5:0.5, flow rate: 0.5 mL/min, retention time (min) 15.7 (*S* isomer), 19.9 (*R* isomer). $[\alpha]_D^{25}$ = –33.8 (*c* 1.00, $CHCl_3$) [lit.[15] (*R*)-2-Octyl 4-nitrobenzoate, $[\alpha]_D^{23}$ = –34.2 (*c* 2.21, $CHCl_3$); Authentic sample (retention): (*S*)-2-Octyl 4-nitrobenzoate (99:1 er), $[\alpha]_D^{25}$ = +39.6 (*c* 1.00, $CHCl_3$)].

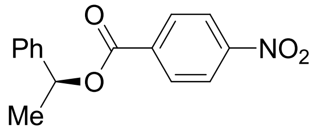

(*S*)-1-Phenylethyl 4-nitrobenzoate (42).[16] (+)-(*R*)-1-Phenyl-1-ethanol (**29**) (122 mg, 1.0 mmol, 98:2 er) and 4-nitrobenzoic acid (**4**) (184 mg, 1.1 mmol) were used as substrates. 12 h. 89% yield (241 mg, 0.89 mmol) 92:8 er. Colorless oil. Elute: Hex/AcOEt = 30:1. ^{1}H NMR (600 MHz, $CDCl_3$) δ 8.28 (2H, app. d, J = 8.9 Hz), 8.23 (2H, app. d, J = 8.9 Hz), 7.45−7.44 (2H, m), 7.39 (2H, app. t, J = 7.6 Hz), 7.34 (1H, app. t, J = 7.2 Hz), 6.16 (1H, q, J = 6.5 Hz), 1.71 (3H, d, J = 6.5 Hz); ^{13}C NMR (150 MHz, $CDCl_3$) δ 163.9, 150.5, 140.9, 135.8, 130.7, 128.7, 128.2, 126.1, 123.5, 74.2, 22.2; The enantiomeric ratio was determined by HPLC analysis using a chiral column. Chiral HPLC: Daicel-Chiralpak OJ-H 46 × 150 mm, multi UV-Vis detector (210 nm), room temperature eluent: (*n*-hexane/*i*-PrOH) 1:1 flow rate: 0.5 mL/min, retention time (min) 22.3 (*R* isomer), 26.5 (*S* isomer). $[\alpha]_D^{25}$ = +43.9 (*c* 1.00, $CHCl_3$) [Authentic sample (retention): (*R*)-1-Phenylethyl 4-nitrobenzoate (98:2 er), $[\alpha]_D^{25}$ = –49.0 (*c* 1.00, $CHCl_3$); lit.[15] (*R*) -1-Phenylethyl 4-nitrobenzoate, $[\alpha]_D^{23}$ = –50.5 (*c* 1.29, $CHCl_3$)].

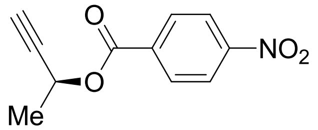

(*S*)-3-Butynyl 4-nitrobenzoate (43).[17] (+)-(*R*)-3-Butyn-2-ol (**30**) (70.1 mg, 1.0 mmol, 99:1 er) and 4-nitrobenzoic acid (**4**) (184 mg, 1.1 mmol) were used as substrates. 12 h. 87% yield (189 mg, 0.87 mmol) 96:4 er. White solid. Elute: Hex/AcOEt = 30:1. ^{1}H NMR (600 MHz, $CDCl_3$) δ 8.30 (2H, app. d, J = 9.3 Hz), 8.24 (2H, app. d, J = 9.3 Hz), 5.71 (1H, qd, J = 6.6, 1.8 Hz), 2.54 (1H, d, J = 1.8 Hz), 1.68 (3H, d, J = 6.6 Hz); ^{13}C NMR (150 MHz, $CDCl_3$) δ 163.6, 150.7, 135.1, 130.9, 123.6, 81.4, 73.7, 61.7, 21.2; The enantiomeric ratio was determined by HPLC analysis using a chiral column. Chiral HPLC: Daicel-Chiralpak OJ-H 46 × 150 mm, multi UV-Vis detector (230 nm), room temperature eluent: (*n*-hexane/*i*-PrOH) 5:1, flow rate: 1.0 mL/min, retention time (min) 12.4 (*R* isomer), 25.4 (*S* isomer). $[\alpha]_D^{25}$ = –9.2 (*c* 1.00, $CHCl_3$) [Authentic sample (retention): (*R*)-3-Butynyl 4-nitrobenzoate (99:1 er); $[\alpha]_D^{25}$ = +14.4 (*c* 1.00, $CHCl_3$)].

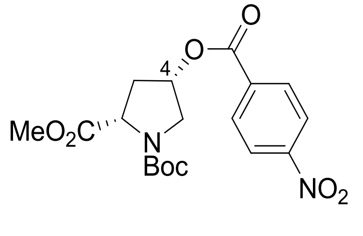

1-(*tert*-butyl) 2-methyl (2*S*,4*S*)-4-((4-nitrobenzoyl)oxy)pyrrolidine-1,2-dicarboxylate (44).[18] (2*S*,4*R*)-4-Hydroxypyrrolidine-1,2-dicarboxylic acid 1-*tert*-butyl 2-methyl ester (**31**) (245 mg, 1.0

mmol) and 4-nitrobenzoic acid (**4**) (184 mg, 1.1 mmol) were used as substrates. 16 h. 88% yield (346 mg, 0.88 mmol, as a single diastereomer in NMR analysis). [Fe(Pc) (3 mol%), PPh_3 (1.5 equiv): 36 h. 91% yield (359 mg, 0.91 mmol), as a single diastereomer in NMR analysis].White solid. Elute: Hex/AcOEt=3:1. Rotamers were observed in NMR spectra. ^{1}H NMR (600 MHz, $CDCl_3$) δ 8.29 (2H, app. d, J = 8.6 Hz), 8.16 (2H, app. d, J = 8.6 Hz), 5.58 (1H, app. d, J = 4.1 Hz), 4.62 and 4.56 (total 1H, both d, both J = 8.4 Hz, based on rotamers), 3.85−3.82 (2H, m), 3.71 and 3.70 (total 3H, both s, based on rotamers), 2.64−2.53 (1H, m), 2.48 and 2.45 (total 1H, both s, based on rotamers), 1.49 and 1.45 (total 9H, both s, based on rotamers); ^{13}C NMR (150 MHz, $CDCl_3$) δ 172.3, 172.1, 164.0, 163.8, 154.0, 153.6, 150.7, 135.0, 130.8, 123.5, 80.6, 80.5, 74.5, 73.4, 57.7, 57.4, 52.5, 52.3, 52.2, 52.1, 36.5, 35.6, 28.3, 28.2.

[The authentic sample of 1-(*tert*-butyl)-2-methyl-(2*S*,4*R*)-4-((4-nitrobenzoyl)oxy)pyrrolidine-1,2-dicarboxylate **(*epi*-44)** was prepared by condensation between (2*S*,4*R*)-4-hydroxypyrrolidine-1,2-dicarboxylic acid 1-*tert*-butyl 2-methyl ester (**31**) and 4-nitrobenzoic acid (**4**) with EDC•HCl. ^{1}H NMR (600 MHz, $CDCl_3$) δ 8.30 (2H, app. d, J = 9.0 Hz), 8.19 (2H, app. d, J = 9.0 Hz), 5.58−5.57 (1H, m), 4.54 and 4.45 (total 1H, both t, both J = 7.8 Hz, based on rotamers), 3.88, 3.86 and 3.71 (total 2H, dd, J = 6.6, 4.2 Hz, d, J = 3.0 Hz and d, J = 12.6 Hz, based on rotamers), 3.79 and 3.78 (total 3H, both s, based on rotamers), 2.60−2.53 (1H, m), 2.38−2.34 (1H, m), 1.47 and 1.44 (total 9H, both s, based on rotamers). No peak on C3 (δ 2.38−2.34 (1H, m)) of 1-(*tert*-butyl)-2-methyl-(2*S*,4*R*)-4-((4-nitrobenzoyl)oxy)pyrrolidine-1,2-dicarboxylate was detected in ^{1}H NMR of the crude product including **44**]

.

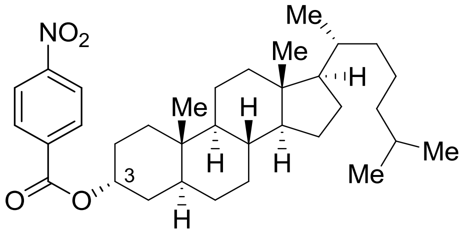

(3*S*)-5α-Cholestan-3-yl 4-nitrobenzoate (45).[14] Dihydrocholesterol (**32**) (389 mg, 1.0 mmol) and 4-nitrobenzoic acid (**4**) (184 mg, 1.1 mmol) were used as substrates. 24 h. 82% yield (439 mg, 0.82 mmol, as a single diastereomer). Pale yellow solid. Elute: Hex/AcOEt = 25:1. ^{1}H NMR (600 MHz, $CDCl_3$) δ 8.31 (2H, app. d, J = 8.9 Hz), 8.22 (2H, app. d, J = 8.9 Hz), 5.32−5.31 (1H, m), 1.99 (1H, dt, J = 12.5, 3.2 Hz), 1.88 (1H, br d, J = 12.6 Hz), 1.86−1.80 (2H, m), 1.70−1.50 (8H, m), 1.40−0.92 (18H, m), 0.91 (3H, d, J = 6.6 Hz), 0.87 (3H, d, J = 6.6 Hz), 0.86 (3H, d, J = 7.2 Hz), 0.85 (3H, s), 0.78 (1H, dt, J = 16.2, 6.3 Hz), 0.67 (3H, s); ^{13}C NMR (150 MHz, $CDCl_3$) δ 164.0, 150.4, 136.6, 130.6, 123.5, 72.2, 56.5, 56.3, 54.4, 42.6, 40.5, 40.0, 39.5, 36.2, 35.9, 35.8, 35.4, 33.2, 32.9, 31.9, 28.4, 28.2, 28.0, 26.2, 24.1, 23.8, 22.8, 22.5, 20.8, 18.7, 12.1, 11.4.

[The authentic sample of (3*R*)-5α-cholestan-3-yl 4-nitrobenzoate **(*epi*-45)** was prepared by condensation of dihydrocholesterol (**32**) and 4-nitrobenzoic acid (**4**) with EDC•HCl. ^{1}H NMR

(600 MHz, $CDCl_3$) δ 8.27 (2H, app. d, J = 8.4 Hz), 8.21 (2H, app. d, J = 8.4 Hz), 5.02−4.94 (1H, m), 1.99−1.97 (2H, m), 1.82−1.79 (2H, m), 1.73−1.66 (3H, m), 1.57−1.49 (3H, m), 1.37−1.23 (9H, m), 1.16−0.98 (9H, m), 0.91 (3H, d, J = 6.6 Hz), 0.89−0.86 (9H, m), 0.69−0.68 (1H, m), 0.66 (3H, s). No peak on C3 (δ 5.02−4.94 (1H, m)) of (3*R*)-5α-cholestan-3-yl 4-nitrobenzoate was detected in ^1H NMR of the crude product including **45**]

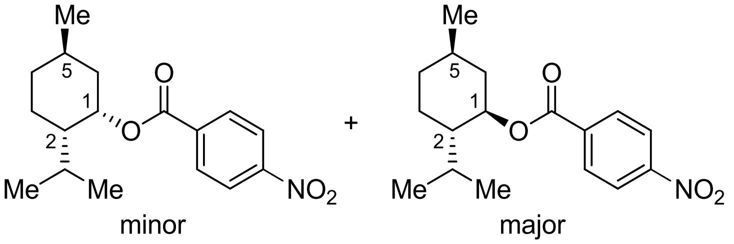

(1*S*,2*S*,5*R*)-Menthyl 4-nitrobenzoate (46).[14] (–)-Menthol (**33**) (156 mg, 1.0 mmol) and 4-nitrobenzoic acid (**4**) (184 mg, 1.1 mmol) were used as substrates. 48 h. 76% yield (230 mg, 0.76 mmol, as an inseparable mixture of two diastereomers, 11:89 dr in NMR analysis). White solid. Elute: Hex/AcOEt = 30:1. ^1H NMR (600 MHz, $CDCl_3$, for major isomer) δ 8.29 (2H, app. d, J = 8.9 Hz), 8.21 (2H, app. d, J = 8.9 Hz), 4.98 (1H, td, J = 10.8, 4.2 Hz), 2.15−2.12 (1H, m), 1.95−1.90 (1H, m), 1.77−1.73 (2H, m), 1.62−1.55 (2H, m), 1.19−1.11 (2H, m), 0.95 (3H, d, J = 6.6 Hz), 0.93 (3H, d, J = 7.2 Hz), 0.80 (3H, d, J = 6.6 Hz); ^1H NMR (600 MHz, $CDCl_3$, for partial peaks of minor isomer) δ 5.50 (1H, app. d, J = 2.4 Hz); ^{13}C NMR (150 MHz, $CDCl_3$) δ 164.2 (major), 164.0 (minor), 150.4 (major), 136.3 (minor), 136.2 (major), 130.62 (major), 130.58 (minor), 123.53 (minor), 123.46 (major), 76.1 (major), 73.1 (minor), 47.1 (major), 46.9 (minor), 40.8 (major), 39.1 (minor), 34.7 (minor), 34.2 (major), 31.4 (major), 29.4 (minor), 26.8 (minor), 26.5 (major), 25.3 (minor), 23.5 (major), 22.1 (minor), 22.0 (major), 20.9 (minor), 20.74 (minor), 20.70 (major), 16.4 (major); The diastereomeric ratio was determined from integration values of peaks of C1 [4.98 (1H, td, J = 10.8, 4.2 Hz, for major isomer), 5.50 (1H, app. d, J = 2.4 Hz, for minor isomer)].

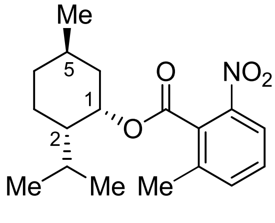

(1*S*,2*S*,5*R*)-Menthyl 2-methyl-6-nitrobenzoate (47). (–)-Menthol (**33**) (156 mg, 1.0 mmol) and 2-methyl-6-nitrobenzoic acid (199 mg, 1.1 mmol) were used as substrates. 96 h. 66% yield (195 mg, 0.66 mmol, as a single diastereomer in NMR analysis). Colorless crystals, mp 66.5−67.0 °C (*n*-hexane/EtOAc). Elute: Hex/AcOEt = 15:1. ^1H NMR (600 MHz, $CDCl_3$) δ 7.98 (1H, d, J = 7.8 Hz), 7.53 (1H, d, J = 7.2 Hz), 7.45 (1H, t, J = 7.8 Hz), 5.567 (1H, app. d, J = 2.1 Hz), 2.43 (3H, s), 2.37−2.32 (1H, m), 1.75−1.69 (2H, m), 1.62−1.57 (1H, m), 1.51−1.45 (1H, m), 1.22 (1H, qd, J =

12.6, 3.6 Hz), 1.14 (1H, ddd, J = 15.0, 13.2, 2.4 Hz), 1.08−1.03 (1H, m), 1.01 (3H, d, J = 6.6 Hz), 0.96−0.90 (1H, m), 0.91 (3H, d, J = 6.6 Hz), 0.88 (3H, d, J = 6.6 Hz); ^{13}C NMR (150 MHz, $CDCl_3$) δ 166.1, 146.0, 137.2, 136.0, 130.3, 129.3, 121.8, 73.7, 47.1, 38.4, 34.6, 28.7, 26.6, 24.9, 22.1, 20.99, 20.95, 19.1; IR ($CHCl_3$, cm^{-1}) υ 2954, 1736, 1536, 1456, 1347, 1270; HRMS (DART+) (*m*/*z*): $[M+NH_4]^+$ calcd for $C_{18}H_{29}N_2O_4$: 337.2127, found: 337.2137.

[The authentic sample of (1*R*,2*S*,5*R*)-menthyl 2-methyl-6-nitrobenzoate **(*epi*-47)** was preparation by acylation of (–)-menthol (**33**) and 2-methyl-6-nitrobenzoic anhydride. ^{1}H NMR (600 MHz, $CDCl_3$) δ 8.00 (1H, d, J = 8.4 Hz), 7.53 (1H, d, J = 7.8 Hz), 7.45 (1H, t, J = 7.8 Hz), 4.99 (1H, dt, J = 12.8, 4.2 Hz), 2.43 (3H, s), 2.41−2.37 (1H, m), 2.02−2.00 (1H, m), 1.73 (2H, app. d, J = 12.8 Hz), 1.59−1.56(1H, m), 1.44 (1H, app. t, J = 6.6 Hz), 1.15−1.05 (2H, m), 0.97 (3H, d, J = 6.0 Hz), 0.93−0.91 (1H, m), 0.89 (3H, d, J = 6.6 Hz), 0.86 (3H, d, J = 6.6 Hz). No peak on C1 (δ 4.99 (1H, td, J = 12.8, 4.2 Hz)) of (1*R*,2*S*,5*R*)-menthyl 2-methyl-6-nitrobenzoate was detected in ^{1}H NMR of the crude product including **47**]

2. Kinetic studies for developed Mitsunobu reagents

Procedure for kinetic studies of the reaction of ethyl 2-arylazocarboxylates with triphenylphosphine and water:

A solution of ethyl 2-arylazocarboxylate (50 mM), triphenylphosphine (500 mM) and water (500 mM) in THF (1.0 mL) was prepared in a 10 mL round-bottom flask at 25 ˚C under a nitrogen atmosphere, and a small amount (ca. 0.5 mL) of a sample was taken from the reaction mixture, and absorption spectra of the sample continuously were measured in a 0.1 mm quartz cell. The first measurement was defined as 0 min for convenience (ca. 2 minutes from addition of reagents). Concentrations of the azo compound at each time were estimated from the absorption intensity based on calibration curves.

Table S1. The kinetic data of substituted ethyl 2-arylazocarboxylates

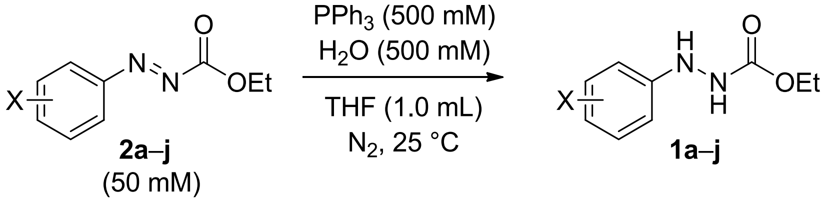

Entry	X	σ	k_{obs} (min^{-1})	log(k_X/k_H)
1	*p*-OMe (**b**)	–0.28	0.0005	–1.09342
2	*p*-Me (**c**)	–0.14	0.0021	–0.47017
3	*p*-H (**d**)	0	0.0062	0
4	*p*-F (**e**)	0.06	0.0064	0.013788
5	*p*-Cl (**f**)	0.22	0.0195	0.497643
6	*m*-Cl (**g**)	0.37	0.0375	0.809668
7	*p*-CO_2Et (**h**)	0.44	0.0639	1.013109
8	*p*-CF_3 (**i**)	0.53	0.1218	1.293256
9	*p*-CN (**j**)	0.71	0.3224	1.716003
10	3,4-diCl (**a**)	–	0.0851	1.137538

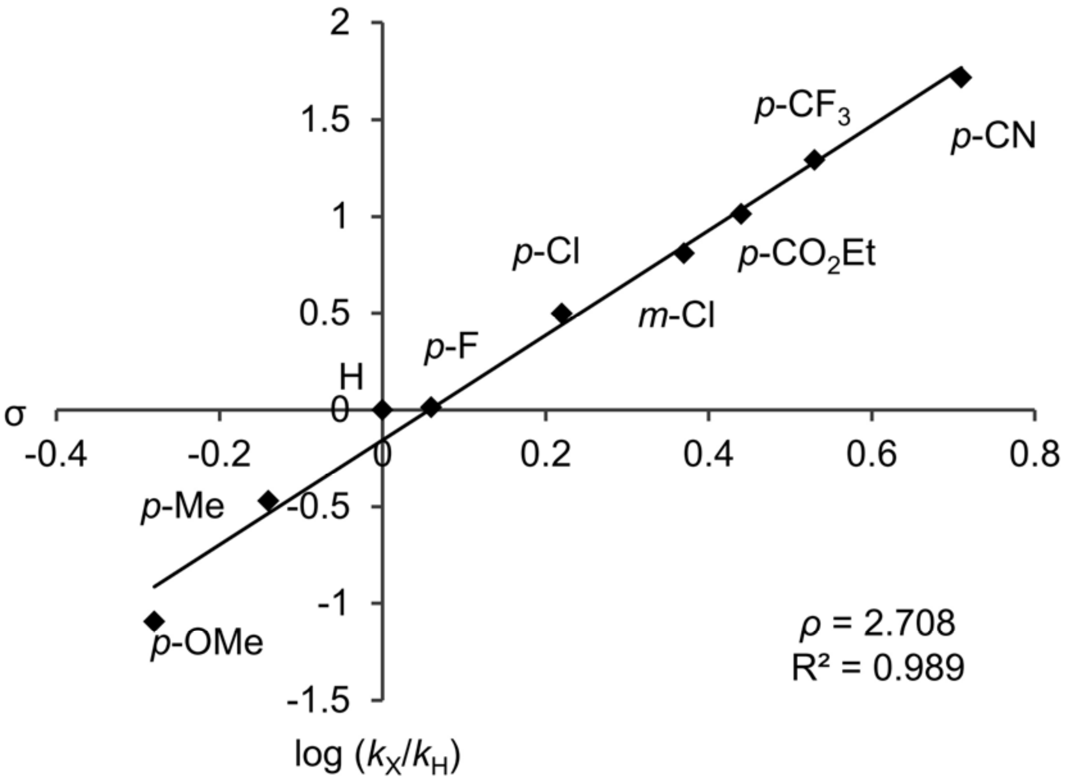

Figure S1. The Hammett plot of *p*-substituted ethyl arylazocarboxylates

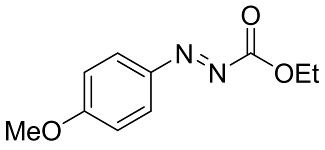

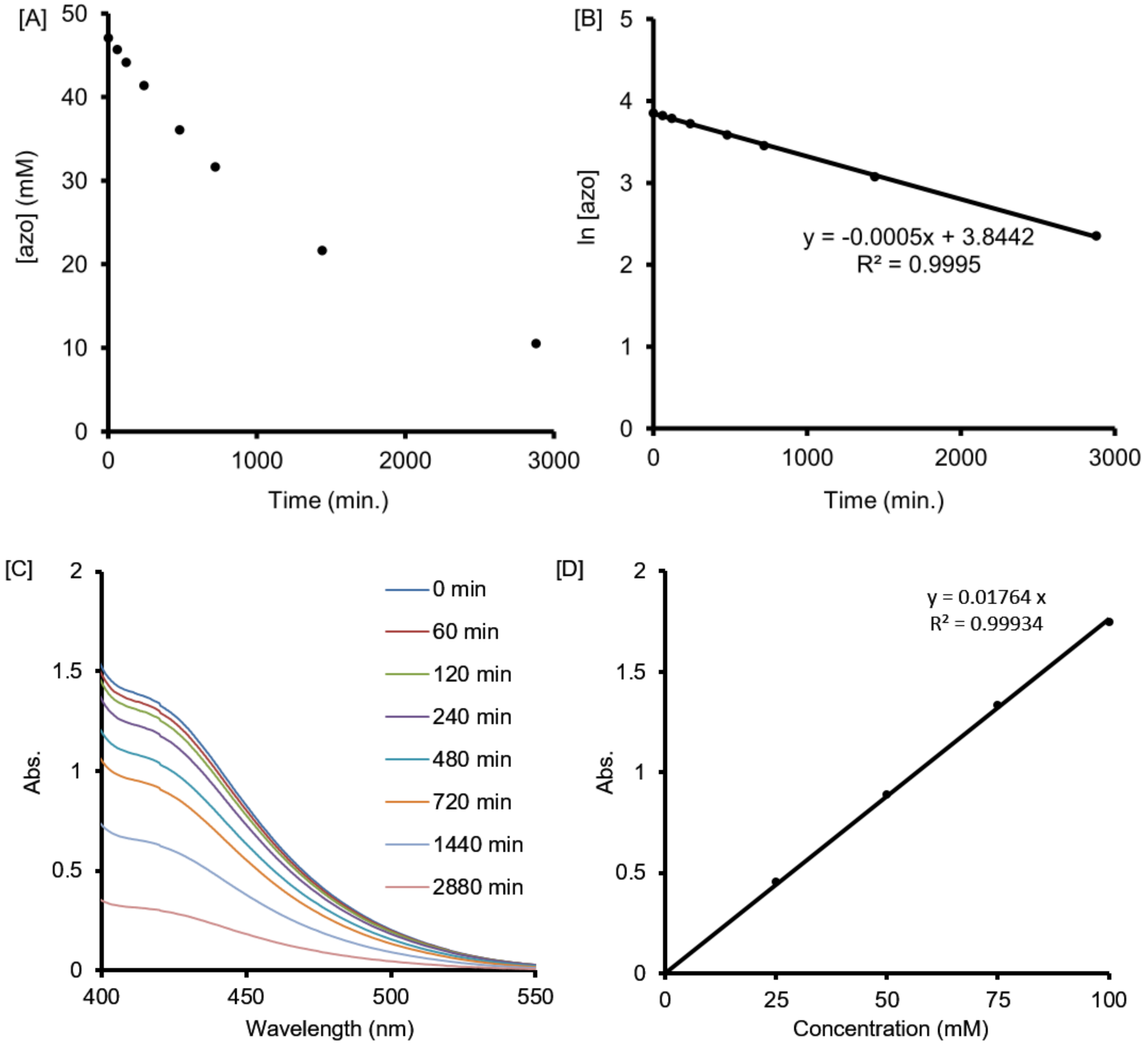

Time (min)	0	60	120	240	480	720	1440	2880
Abs (450nm)	0.83027	0.80586	0.77874	0.72994	0.63628	0.55819	0.38182	0.18547
Conc (mM)	47.06746	45.68367	44.14626	41.37982	36.07029	31.64342	21.64512	10.51417

Figure S2. [A] Plot of the concentration of ethyl 2-(4-methoxyphenyl)azocarboxylate (**2b**) against time. [B] Semi-log plot of [A]. [C] Visible light absorption spectra. [D] Calibration curve.

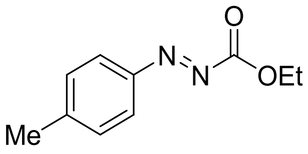

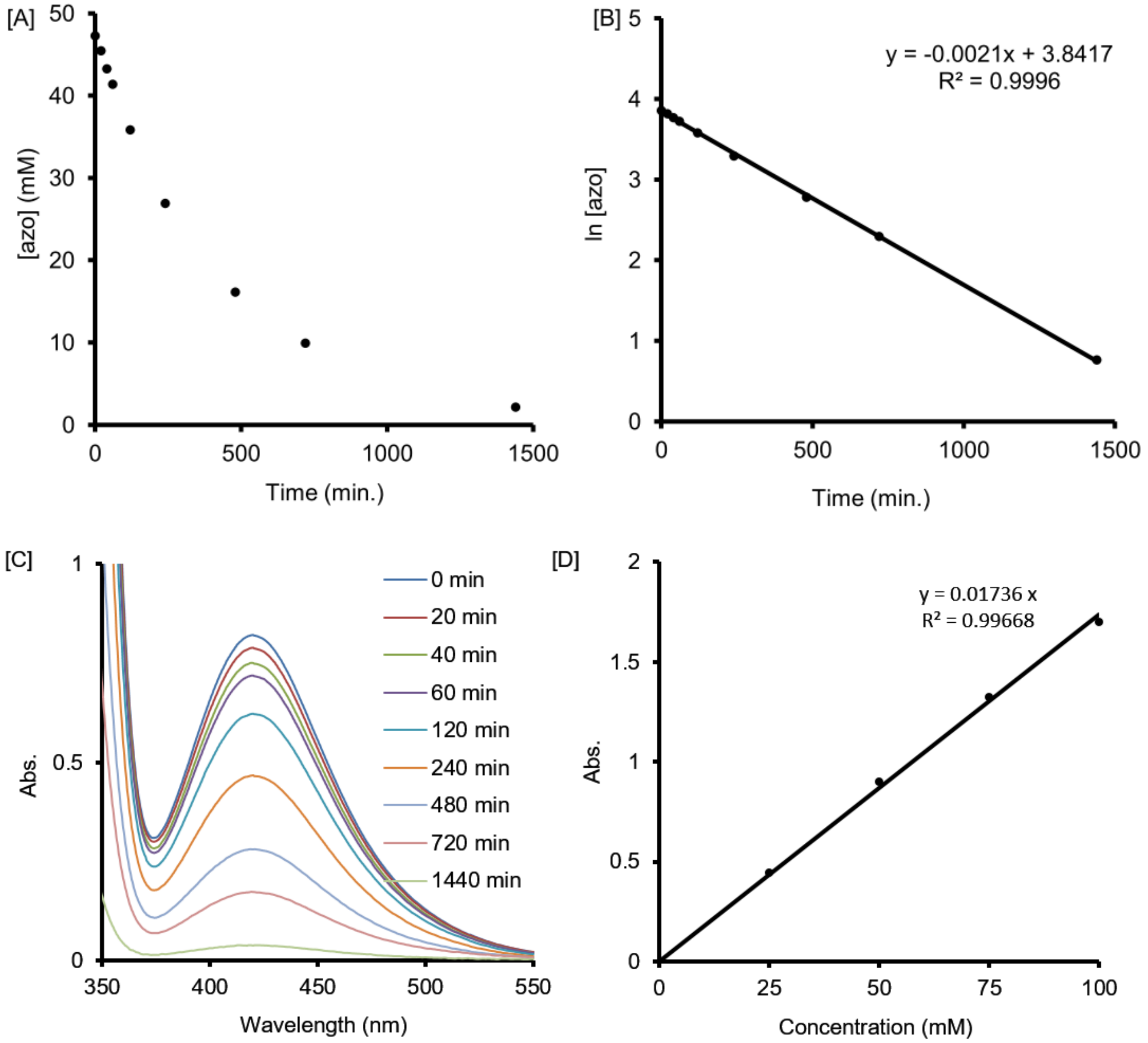

Time (min)	0	20	40	60	120	240	480	720	1440
Abs (420nm)	0.82056	0.78905	0.75101	0.71838	0.62215	0.46707	0.27989	0.17218	0.03721
Conc (mM)	47.26728	45.45219	43.26094	41.38134	35.83813	26.90495	16.1227	9.918203	2.143433

Figure S3. [A] Plot of the concentration of ethyl 2-(4-methylphenyl)azocarboxylate (**2c**) against time. [B] Semi-log plot of [A]. [C] Visible light absorption spectra. [D] Calibration curve.

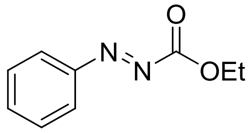

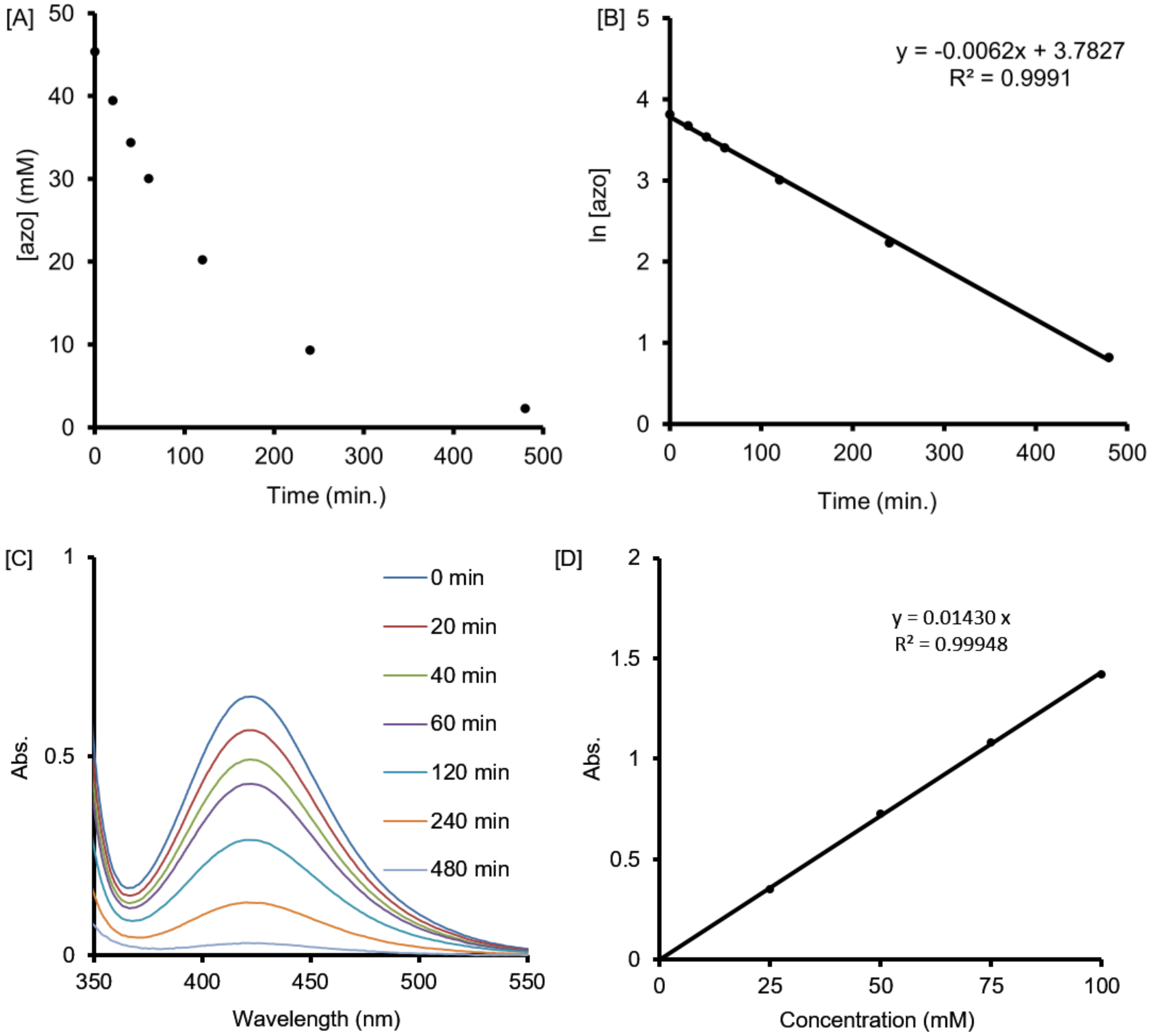

Time (min)	0	20	40	60	120	240	480
Abs (421nm)	0.64862	0.56421	0.49164	0.42943	0.28917	0.13318	0.03248
Conc (mM)	45.35804	39.45524	34.38042	30.03007	20.22168	9.313287	2.271329

Figure S4. [A] Plot of the concentration of ethyl 2-phenylazocarboxylate (**2d**) against time. [B] Semi-log plot of [A]. [C] Visible light absorption spectra. [D] Calibration curve.

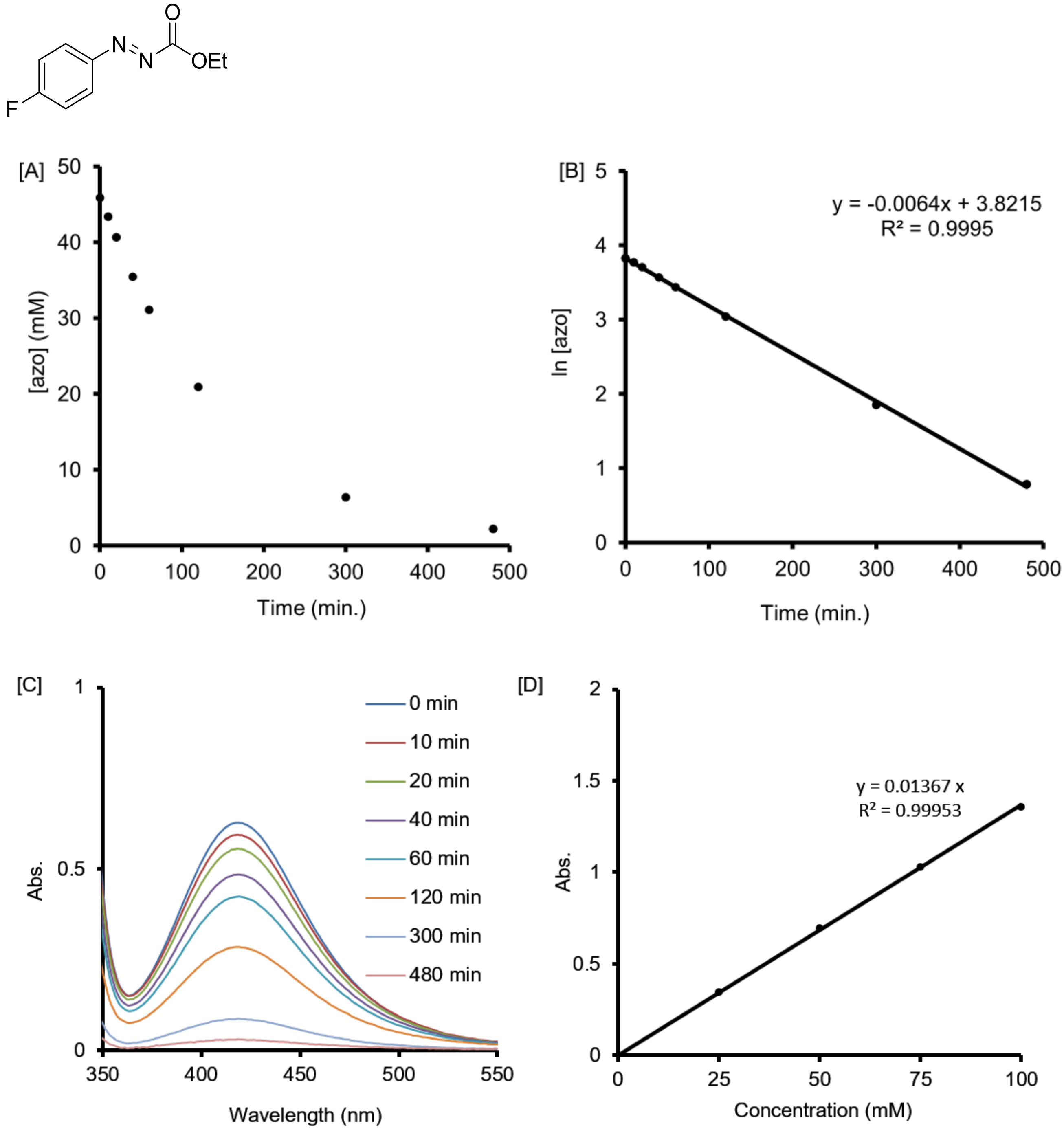

Time (min)	0	10	20	40	60	120	300	480
Abs (419nm)	0.62695	0.59284	0.55568	0.48446	0.42485	0.28585	0.08697	0.02991
Conc (mM)	45.8632	43.36796	40.6496	35.43965	31.07901	20.91075	6.362107	2.188003

Figure S5. [A] Plot of the concentration of ethyl 2-(4-fluorophenyl)azocarboxylate (**2e**) against time. [B] Semi-log plot of [A]. [C] Visible light absorption spectra. [D] Calibration curve.

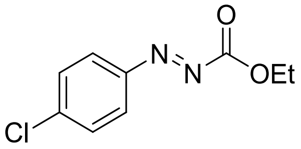

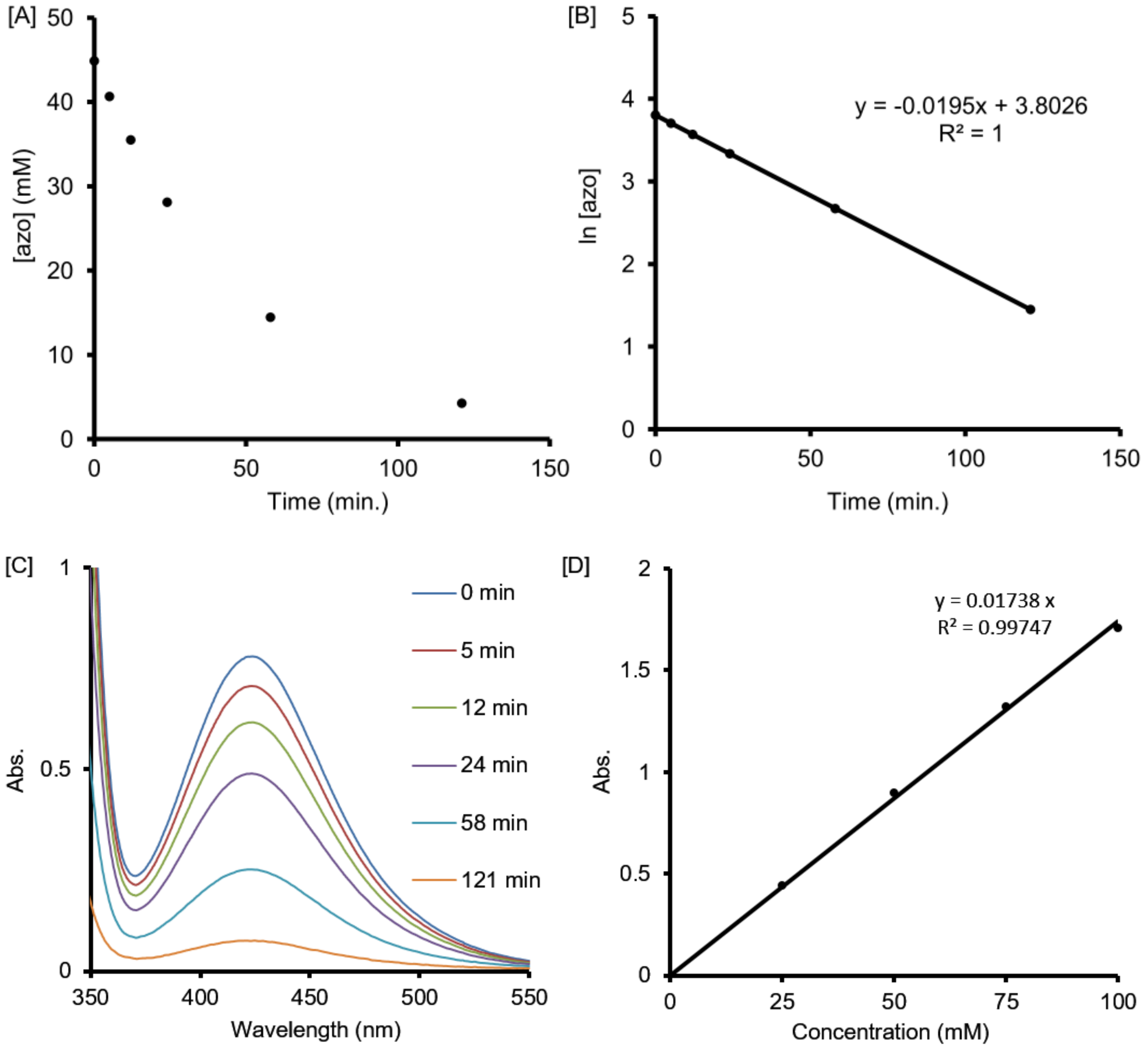

Time (min)	0	5	12	24	58	121
Abs (423nm)	0.77987	0.70652	0.61698	0.48847	0.25126	0.07405
Conc (mM)	44.87169	40.65132	35.49942	28.10529	14.45685	4.260644

Figure S6. [A] Plot of the concentration of ethyl 2-(4-chlorophenyl)azocarboxylate (**2f**) against time. [B] Semi-log plot of [A]. [C] Visible light absorption spectra. [D] Calibration curve.

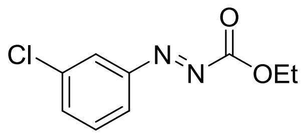

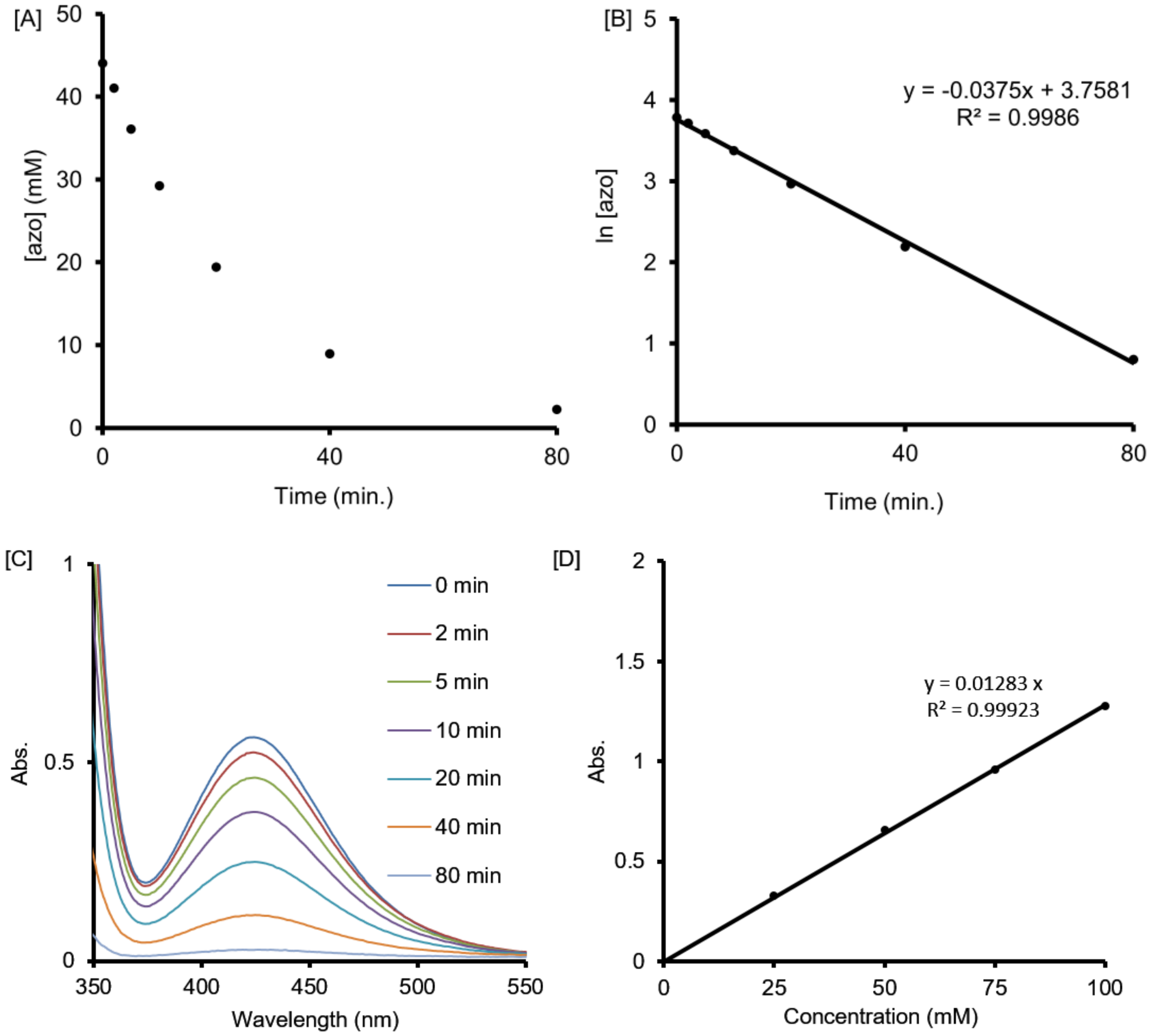

Time (min)	0	2	5	10	20	40	80
Abs (424nm)	0.56505	0.52644	0.46294	0.37509	0.24915	0.11483	0.0286
Conc (mM)	44.04131	41.03196	36.08262	29.23539	19.41933	8.950117	2.22915

Figure S7. [A] Plot of the concentration of ethyl 2-(3-chlorophenyl)azocarboxylate (**2g**) against time. [B] Semi-log plot of [A]. [C] Visible light absorption spectra. [D] Calibration curve.

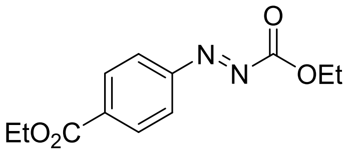

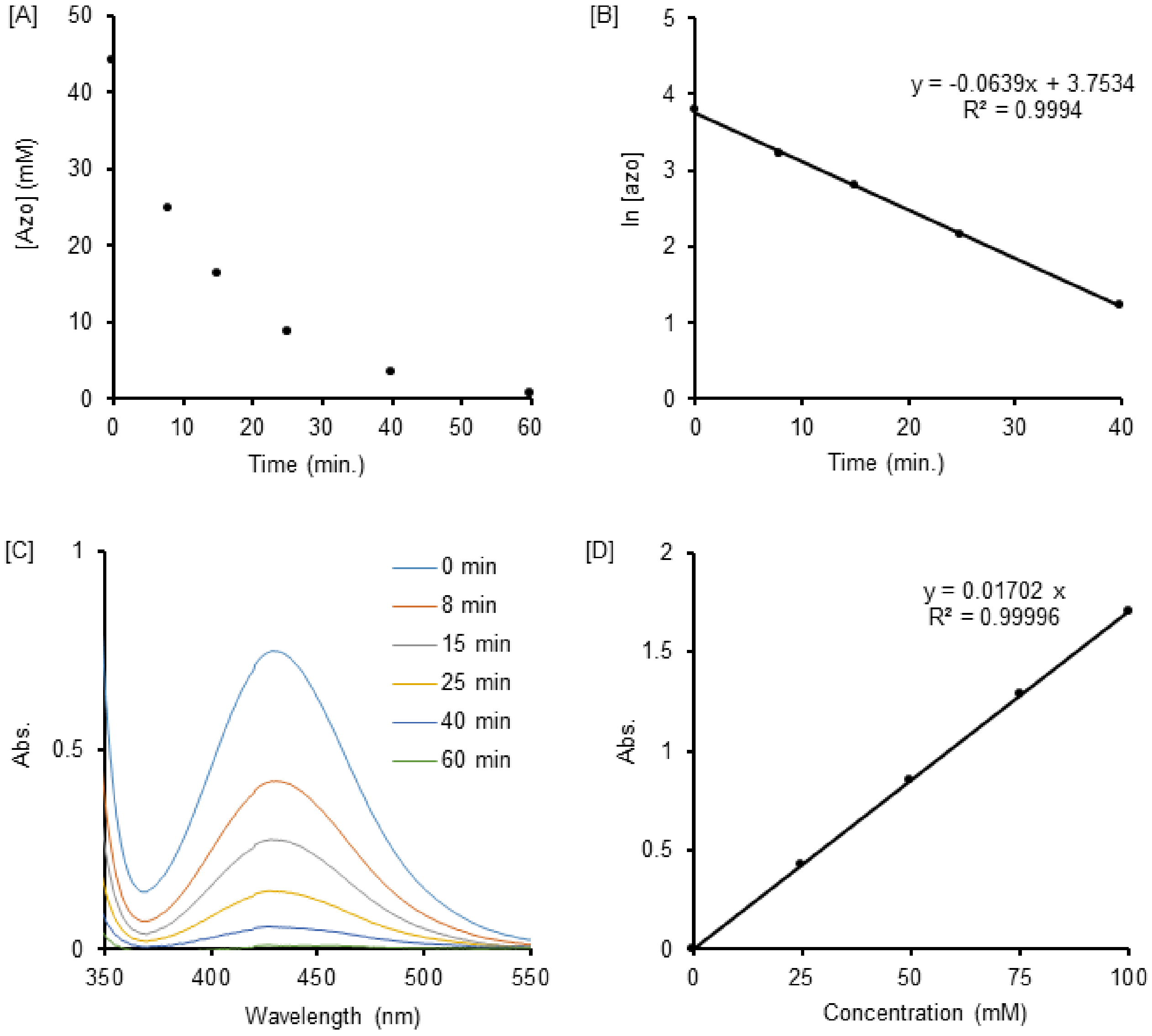

Time (min)	0	8	15	25	40	60
abs (429 nm)	0.74995	0.42258	0.27573	0.1463	0.05711	0.01066
Conc (mM)	44.06287	24.82844	16.20035	8.59577	3.355464	0.626322

Figure S8. [A] Plot of the concentration of ethyl 2-[4-(ethoxycarbonyl)phenyl]azocarboxylate (**2h**) against time. [B] Semi-log plot of [A]. [C] Visible light absorption spectra. [D] Calibration curve.

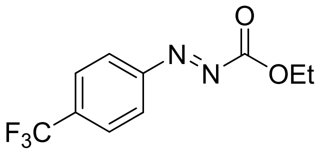

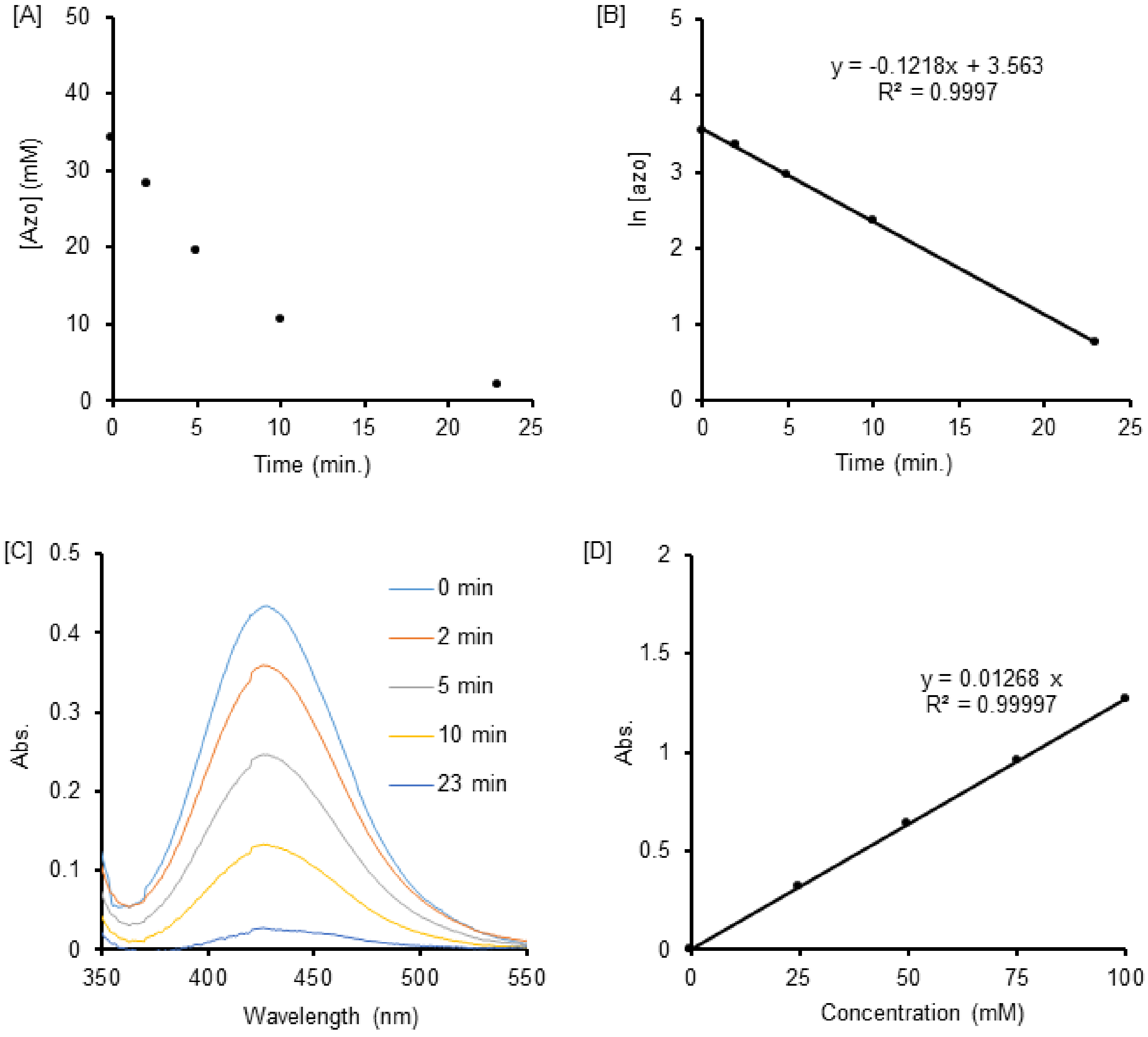

Time (min)	0	2	5	10	23
abs (427 nm)	0.43355	0.35835	0.24598	0.13274	0.02699
Conc (mM)	34.19164	28.26104	19.39905	10.46845	2.128549

Figure S9. [A] Plot of the concentration of ethyl 2-[4-(trifluoromethyl)phenyl]azocarboxylate (**2i**) against time. [B] Semi-log plot of [A]. [C] Visible light absorption spectra. [D] Calibration curve.

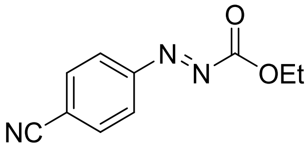

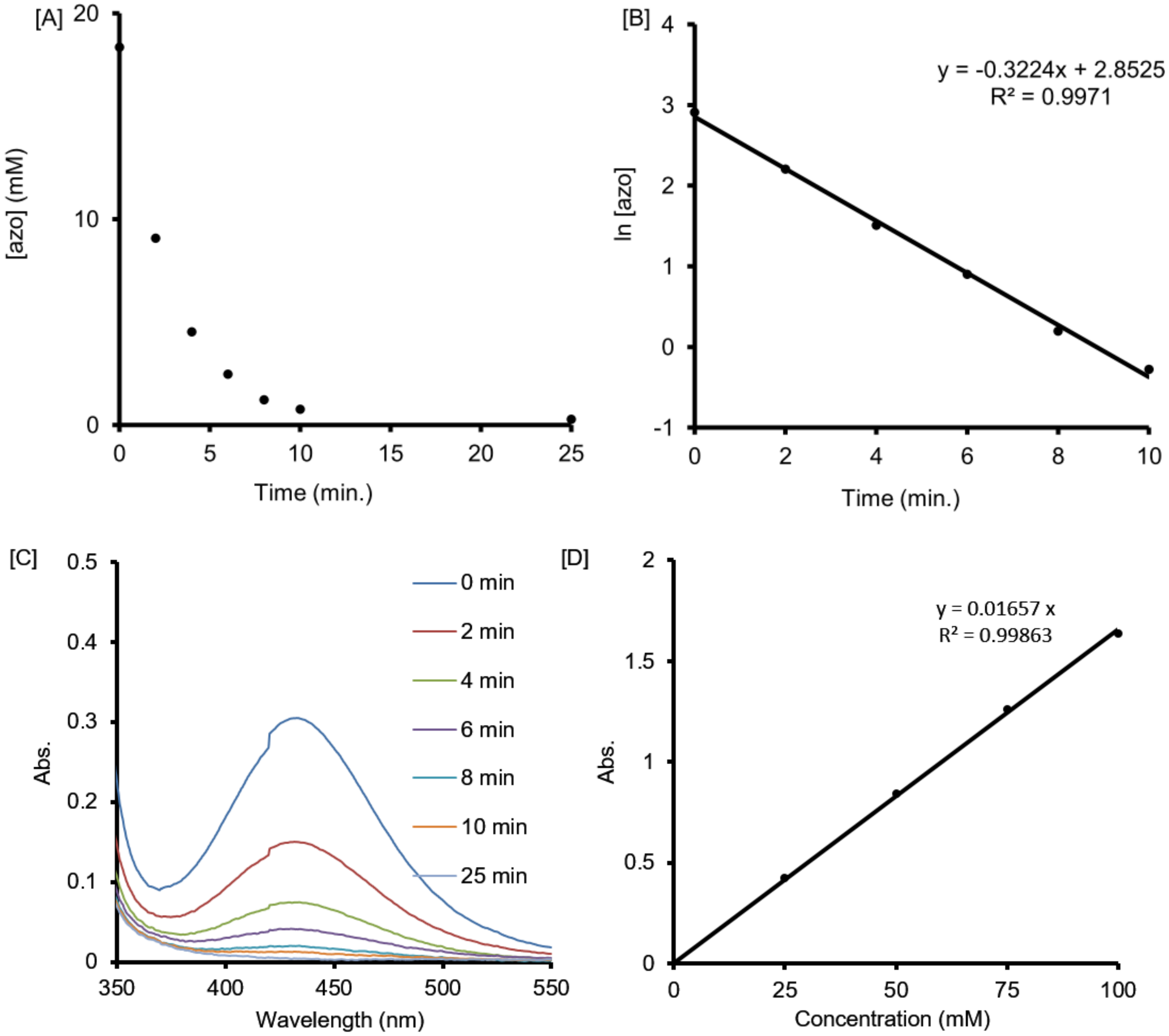

Time (min)	0	2	4	6	8	10	25
Abs (434nm)	0.30405	0.15027	0.07488	0.04079	0.02017	0.01255	0.00448
Conc (mM)	18.34943	9.068799	4.51901	2.461678	1.21726	0.757393	0.270368

Figure S10. [A] Plot of the concentration of ethyl 2-(4-cyanophenyl)azocarboxylate (**2j**) against time. [B] Semi-log plot of [A]. [C] Visible light absorption spectra. [D] Calibration curve.

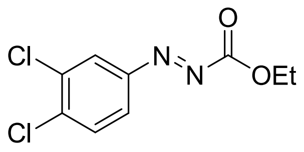

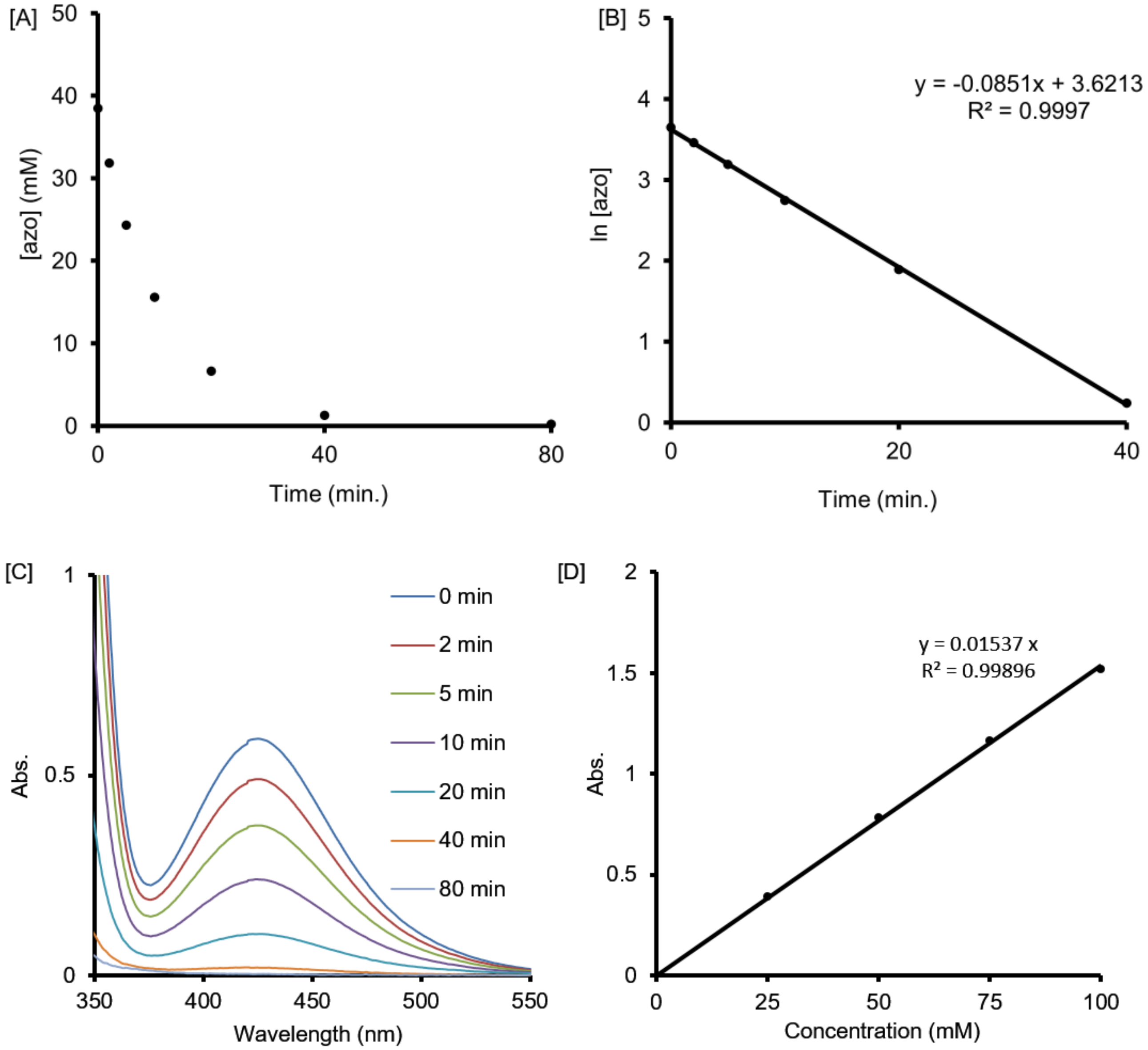

Time (min)	0	2	5	10	20	40	80
Abs (425nm)	0.59126	0.48934	0.37366	0.2394	0.10182	0.01953	0.00308
Conc (mM)	38.46845	31.83735	24.311	15.5758	6.624593	1.270657	0.20039

Figure S11. [A] Plot of the concentration of ethyl 2-(3,4-dichlorophenyl)azocarboxylate (**2a**) against time. [B] Semi-log plot of [A]. [C] Visible light absorption spectra. [D] Calibration curve.

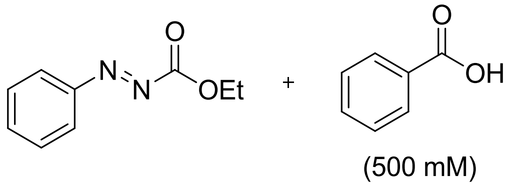

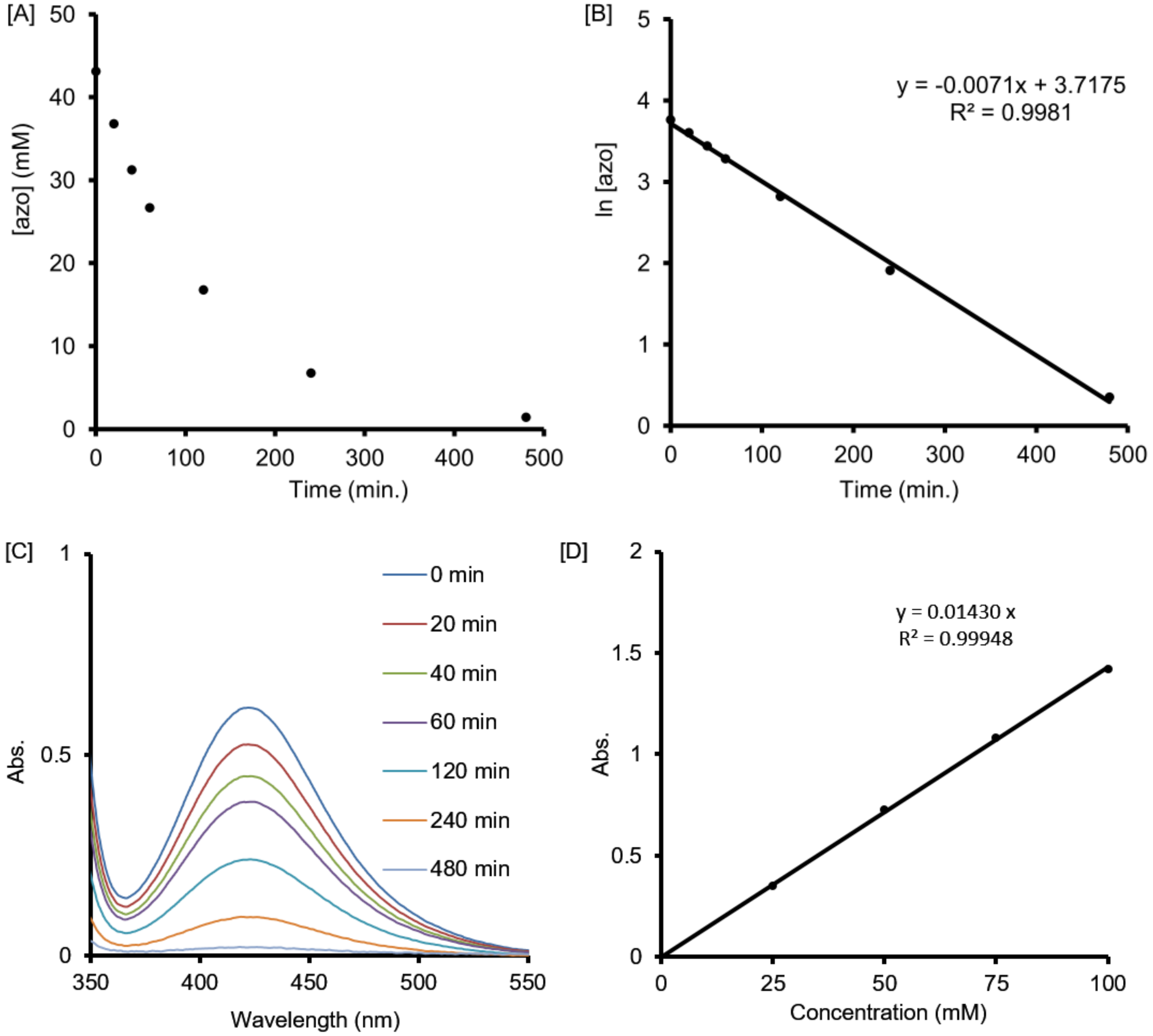

Time (min)	0	20	40	60	120	240	480
Abs (421nm)	0.61657	0.5262	0.44672	0.38147	0.23986	0.09656	0.02032
Conc (mM)	43.11678	36.7972	31.23916	26.67622	16.77343	6.752448	1.420979

Figure S12. [A] Plot of the concentration of ethyl 2-phenylazocarboxylate (**2d**) against time in the presence of benzoic acid. [B] Semi-log plot of [A]. [C] Visible light absorption spectra. [D] Calibration curve.

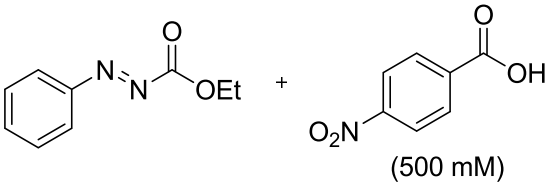

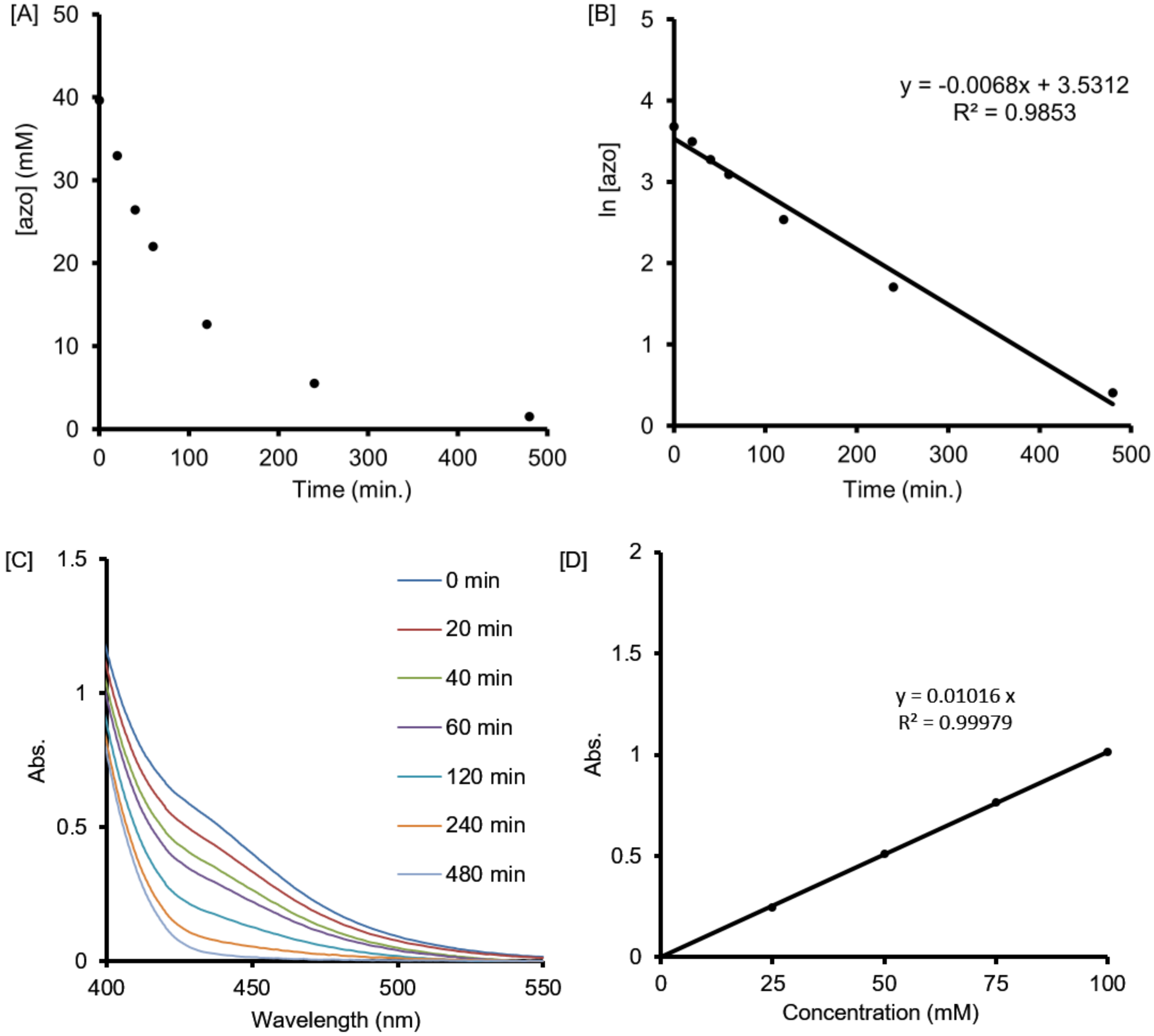

Time (min)	0	20	40	60	120	240	480
Abs (450nm)	0.4026	0.33481	0.26844	0.22353	0.12827	0.05588	0.01521
Conc (mM)	39.62598	32.95374	26.42126	22.00098	12.625	5.5	1.497047

Figure S13. [A] Plot of the concentration of ethyl 2-phenylazocarboxylate (**2d**) against time in the presence of 4-nitrobenzoic acid. [B] Semi-log plot of [A]. [C] Visible light absorption spectra. [D] Calibration curve.

*Monitoring aerobic oxidation of ethyl 2-(3,4-dichlorophenyl)hydrazinecarboxylate (**1a**):*

A solution of ethyl 2-(3,4-dichlorophenyl)hydrazinecarboxylate (**1a**) (1.0 mmol) and dimethyl sulfone (94.1 mg, 1.0 mmol, internal standard) in $CDCl_3$ (5.0 mL) was prepared in a 10 mL round-bottom flask. Iron phthalocyanine (28.4 mg, 50 μmol) was added to the stirred solution at 22 °C under air atmosphere (defined as 0 min). A small amount (ca. 0.1 mL) of a sample was taken from the reaction mixture every 10 min while mixture was stirred, the sample was rapidly filtered through Celite®. An obtained clear solution was diluted with $CDCl_3$ and the solution was analyzed by ^{1}H NMR. The amount of the azo product was estimated from the integration value (full relaxation was confirmed).

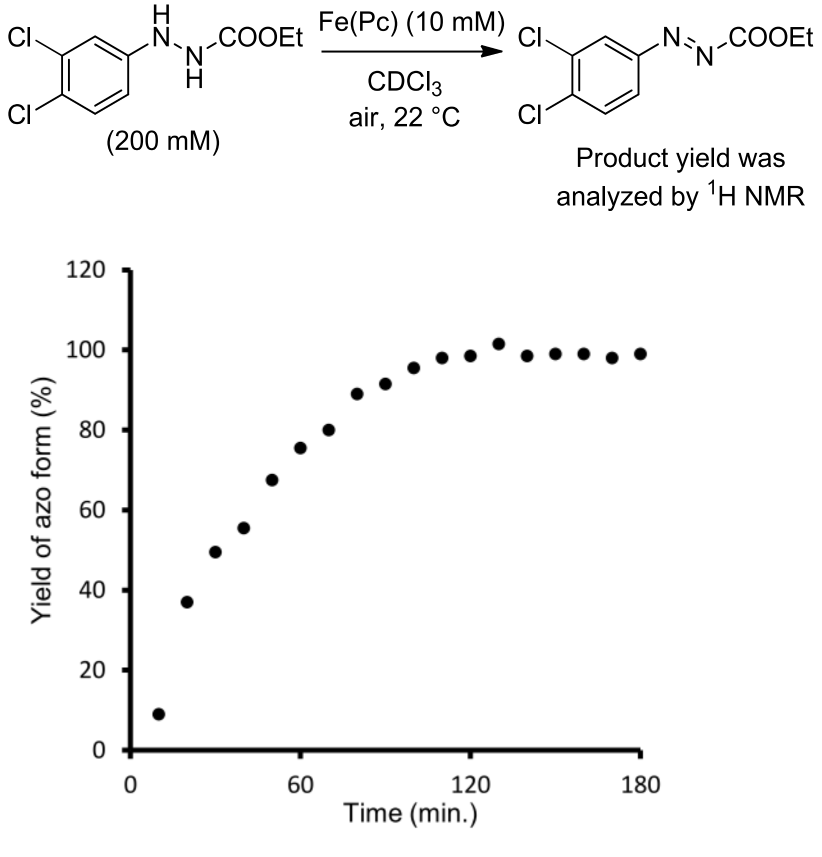

Figure S14. The kinetic plot of the reaction of aerobic oxidation of ethyl 2-(3,4-dichlorophenyl)hydrazinecarboxylate (**1a**) (200 mM) with Fe(Pc) (10 mM) in $CDCl_3$ at 22 °C.

*Aerobic oxidation of 2-[4-(ethoxycarbonyl)phenyl]hydrazinecarboxylate (**1h**) and 2-[4-(trifluoromethyl)phenyl]hydrazinecarboxylate (**1i**) on 0.2 mmol scale:*

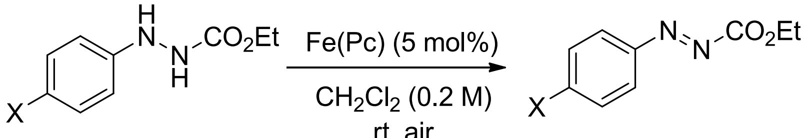

1h (X=CO_2Et)
1i (X=CF_3)
(0.2 mmol)

2h (X=CO_2Et): 4 h, 94%
2i (X=CF_3): 6 h, 96%

In the previous study,[2] iron-catalyzed aerobic oxidation reactions of 2-[4-(ethoxycarbonyl)phenyl]hydrazinecarboxylate (**1h**) and 2-[4-(trifluoromethyl)phenyl]hydrazinecarboxylate (**1i**) were not tested. Therefore, preparative reactions of **1h** and **1i** with iron phthalocyanine were performed under some conditions (0.2 mmol scale in CH_2Cl_2).

According to the reported procedure,[2] **1h** (50.5 mg, 0.20 mmol) and **1i** (46.9 mg, 0.20 mmol) was treated with iron phthalocyanine (5.7 mg, 0.01 mmol) in CH_2Cl_2 (1 mL) under air, respectively. The reaction time was carefully monitored by TLC analysis (**1h**: 4 h; **1i**: 6 h). After the solvent was removed under reduced pressure, the residue was purified by flash chromatography (silica gel, hexane/EtOAc = 5:1) to give 2-[4-(ethoxycarbonyl)phenyl]azocarboxylate (**2h**, 47.1 mg, 94% yield, red oil) and 2-[4-(trifluoromethyl)phenyl]azocarboxylate (**2i**, 47.3 mg, 96% yield, red oil), respectivity.

Thus, these results imply that a trend in reactivity of **1h** and **1i** is roughly similar to that of ethyl 2-phenylhydrazinecarboxylate (**1d**) or ethyl 2-(4-cyanophenyl)hydrazinecarboxylate (**1j**).[2]

3. NMR experiments of intermediates in the Mitsunobu reactions with ethyl 2-arylazocarboxylates

*Procedure for preparation of ethyl 2-phenylazocarboxylate-1-^{15}N (**2d**-^{15}N) and ethyl 2-phenylazocarboxylate-2-^{15}N (**2d**-^{15}N'):*

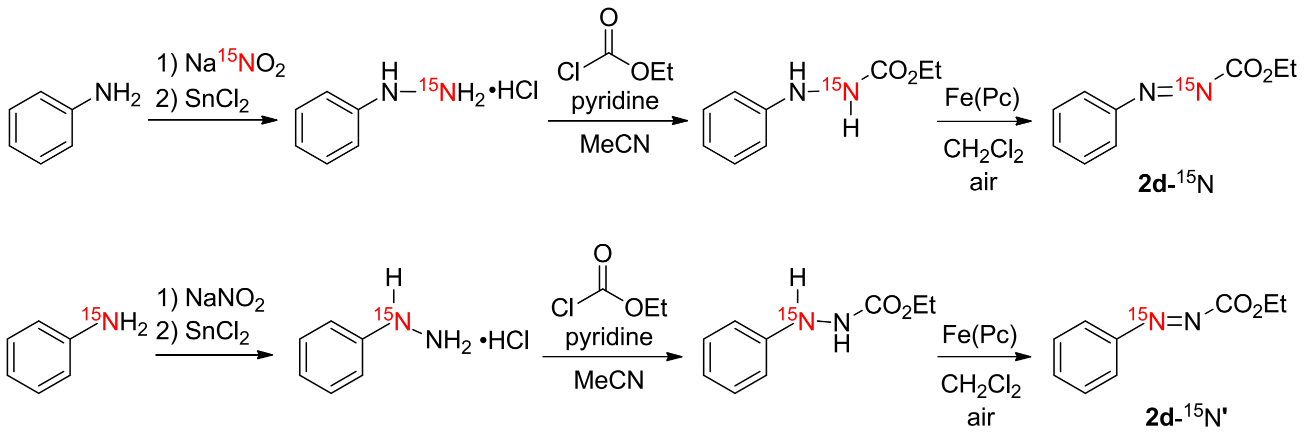

A suspension of aniline (186 mg, 2.0 mmol) or aniline-^{15}N (189 mg, 2.0 mmol, 98.8% ^{15}N incorporation) in concentrated HCl (2.0 mL) was cooled to 0 °C. A solution of Na^{15}NO$_2$ (154 mg, 2.2 mmol, 99.2% ^{15}N incorporation) or NaNO$_2$ (152 mg, 2.2 mmol) in water (2.2 mL) was added dropwise to the suspension. After the mixture was stirred for 30 min at 0 °C, a solution of SnCl$_2$ (1.18 g, 6.2 mmol) in concentrated HCl aq. (6.2 mL) was slowly added to the mixture. The mixture was stirred for 1 h at 0 °C, and the formed precipitate was collected by filtration. The precipitate was washed with diluted HCl aq. and dried under vacuum. To a solution of the obtained crude products and pyridine (791 mg, 10 mmol) in CH$_3$CN (10 mL) was added dropwise ethylchloroformate (260 mg, 2.4 mmol) at 0 °C. The mixture was stirred for 10 min at 0 °C and for 4 h at room temperature. The reaction mixture was poured into water and extracted with ethyl acetate. The combined organic layers were washed with brine, and dried with Na$_2$SO$_4$. After the solvent was evaporated, the crude product was washed by hexane, and dryed under vacuum. A mixture of the crude products and iron phthalocyanine (57 mg, 0.1 mmol) in CH$_2$Cl$_2$ (10 mL) was stirred for 15 h at room temperature under air. After the solvent was removed under reduced pressure, the residue was purified by flash chromatography (silica gel, hexane/EtOAc, 5:1) to give ethyl 2-phenylazocarboxylate-1-*^{15}N* (**2d**-^{15}N) (98.6 mg, 0.55 mmol, 28% yield from aniline) or ethyl 2-phenylazocarboxylate-2-*^{15}N* (**2d**-^{15}N') (79 mg, 0.44 mmol, 23% yield from aniline-^{15}N) as a red oil.

2d-^{15}N: ^{1}H NMR (500 MHz, CDCl$_3$): δ 7.96–7.91 (m, 2H, H-2 and H-6), 7.61–7.56 (m, 1H, H-4), 7.56–7.50 (m, 2H, H-3 and H-5), 4.52 (q, J = 7.1 Hz, 2H, CH$_2$), 1.47 (t, J = 7.1 Hz, 3H, CH$_3$); ^{13}C NMR (126 MHz, CDCl$_3$): δ 162.2 (CO), 151.6 (d, J = 4.9 Hz, C-1), 133.8 (C-4), 129.3 (C-3 and C-5), 123.7 (d, J =3.8 Hz, C-2 and C-6), 64.4 (CH$_2$), 14.1 (CH$_3$); ^{15}N NMR (51 MHz, CDCl$_3$) δ 149 (ArN), 107.2 (^{15}N-CO); IR (neat, cm^{-1}) υ 3064, 2983, 1751, 1588, 1489, 1473, 1449, 1234; HRMS (ESI+) (*m*/*z*): [M+H]$^+$ calcd for C$_9$H$_{11}$N(^{15}N)O$_2$: 180.0785; found: 180.0787.

2d-^{15}N’: ^{1}H NMR (500 MHz, $CDCl_3$): δ 7.96–7.92 (m, 2H, H-2 and H-6), 7.62–7.57 (m, 1H, H-4), 7.56–7.50 (m, 2H, H-3 and H-5), 4.53 (q, J = 7.1 Hz, 2H, CH_2), 1.48 (t, J = 7.1 Hz, 3H, CH_3); ^{13}C NMR (126 MHz, $CDCl_3$): δ 162.2 (d, J = 6.3 Hz, CO), 151.6 (d, J = 2.8 Hz, C-1), 133.8 (C-4), 129.3 (d, J = 1.8 Hz, C-3 and C-5), 123.7 (d, J =4.3 Hz, C-2 and C-6), 64.5 (CH_2), 14.1 (CH_3); ^{15}N NMR (51 MHz, $CDCl_3$): δ 149 (Ar-^{15}N); IR (neat, cm^{-1}) υ 3063, 2983, 2939, 1750, 1587, 1490, 1471, 1459, 1233; HRMS (ESI+) (*m*/*z*): $[M+H]^+$ calcd for $C_9H_{11}N(^{15}N)O_2$: 180.0785; found: 180.0785.

*Procedure for preparation of ethyl 2-(3,4-dichlorophenyl)azocarboxylate-1-^{15}N (**2a**-^{15}N) and ethyl 2-(4-cyanophenyl)azocarboxylate-1-^{15}N (**2j**-^{15}N):*

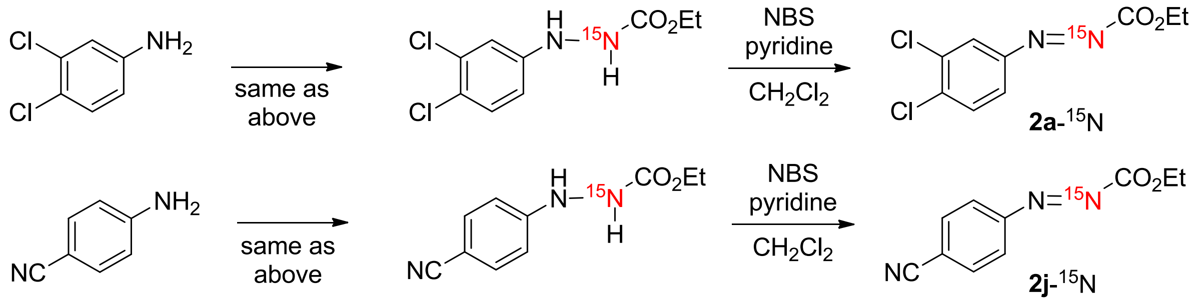

According to the procedure for preparation of **2d**-^{15}N, ethyl 2-(3,4-dichlorophenyl)hydrazinecarboxylate-1-*^{15}N* and ethyl 2-(4-cyanophenyl)hydrazine-1-*^{15}N* were prepared from 3,4-dichloroaniline and 4-cyanoaniline. To a solution of an appropriate hydrazide (0.5 mmol) and pyridine (101 μL, 99 mg, 1.25 mmol) in dichloromethane (4 mL), *N*-bromosuccinimide (98 mg, 0.55 mmol) was added portion-wise at room temperature. After stirring for 30 min at room temperature, the reaction mixture was diluted with dichloromethane (15 mL), and the organic layer was successively washed with 1 M solution of hydrochloric acid (10 mL), 1.5% solution of sodium thiosulfate (5 mL), a saturated solution of sodium bicarbonate (10 mL) and brine (10 mL), and dried over anhydrous sodium sulfate. After the solvent was evaporated, the crude product was purified by flash chromatography (silica gel, hexanes/EtOAc, 6:1) to give the corresponding azo compound.

2a-^{15}N: 92% yield (114 mg, 0.46 mmol). Red solid, mp 49–50 °C.
^{1}H NMR (500 MHz, $CDCl_3$): δ 8.02 (d, J = 2.2 Hz, 1H, H-2), 7.81 (dd, J_1 = 8.6 Hz, J_2 = 2.2 Hz, 1H, H-6), 7.63 (d, J = 8.6 Hz, 1H, H-5), 4.53 (q, J = 7.1 Hz, 2H, CH_2), 1.47 (t, J = 7.1 Hz, 3H, CH_3); ^{13}C NMR (126 MHz, $CDCl_3$): δ 161.6 (CO), 150.3 (d, J = 5.3 Hz, C-1), 138.1 (C-4), 134.0 (C-3), 131.2 (C-5), 124.6 (d, J = 4.1 Hz, C-2), 123.6 (d, J =3.9 Hz, C-6), 64.8 (CH_2), 14.1 (CH_3); ^{15}N NMR (51 MHz, $CDCl_3$): δ 114 (^{15}N-CO); IR (neat, cm^{-1}) υ 3351, 3094, 2983, 1755, 1586, 1520, 1471, 1383, 1234; HRMS (ESI+) (*m*/*z*): $[M+H]^+$ calcd for $C_9H_9Cl_2N(^{15}N)O_2$: 248.0006; found: 248.0006.

2j-^{15}N: 95% yield (97.0 mg, 0.475 mmol). Red solid, mp 45–46 °C.
^{1}H NMR (500 MHz, $CDCl_3$): δ 8.00 (d, J = 8.5 Hz, 2H, H-2 and H-6), 7.85 (d, J = 8.5 Hz, 2H, H-3

and H-5), 4.55 (q, J = 7.2 Hz, 2H, CH_2), 1.48 (t, J = 7.2 Hz, 3H, CH_3); ^{13}C NMR (126 MHz, $CDCl_3$): δ 161.6 (CO), 153.1 (d, J = 5.3 Hz, C-1), 133.4 (C-3 and C-5), 124.0 (d, J =4.1 Hz, C-2 and C-6), 117.8 (CN), 116.7 (C-4), 65.0 (CH_2), 14.1 (CH_3); ^{15}N NMR (51 MHz, $CDCl_3$): δ 145 (Ar-N), 125 (^{15}N-CO); IR (neat, cm^{-1}) υ 3096, 2993, 2230, 1751, 1599, 1496, 1468, 1237; HRMS (ESI+) (*m*/*z*): $[M+H]^+$ calcd for $C_{10}H_{10}N_2(^{15}N)O_2$: 205.0738; found: 205.0738.

Procedure for preparation of diethyl azodicarboxylate-1,2-$^{15}N_2$ (di-^{15}N-DEAD):

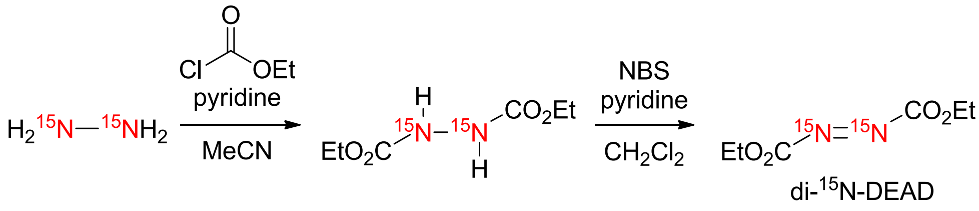

To a solution of hydrazine-$^{15}N_2$ sulfate salt (130 mg, 1.0 mmol) and pyridine (316 mg, 4.0 mmol) in acetonitrile (4 mL), ethyl chloroformate (238 mg, 2.2 mmol) was added dropwise at 0 °C. The reaction mixture was stirred for 15 minutes at 0 °C and then for 1 h at room temperature. The reaction mixture was diluted with dichloromethane (25 mL), and the organic layer was successively washed with 1 M solution of hydrochloric acid (1M, 10 mL), a saturated solution of sodium bicarbonate (10 mL) and brine (10 mL), and dried over anhydrous sodium sulfate. Solvent evaporation afforded pure diethyl hydrazinecaroboxylate-1,2-$^{15}N_2$ (128 mg, 0.72 mmol, 72%) as white crystals, which was used in next step without further purification. According to the procedure for preparation of **2a**-^{15}N and **2j**-^{15}N, diethyl hydrazinecaroboxylate-1,2-$^{15}N_2$ was oxidized by treatment of NBS and pyridine in dichloromethane to give diethyl azodicarboxylate-1,2-$^{15}N_2$ (78 mg, 0.445 mmol, 89%) as a yellow oil.

Diethyl hydrazine-1,2-dicarboxylate-1,2-$^{15}N_2$:
mp 131–133 °C.
^1H NMR (500 MHz, $CDCl_3$): δ 6.44 (d, J = 100.9 Hz, ^{15}NH), 4.21 (q, J = 7.1 Hz, 2H, CH_2), 1.28 (t, J = 7.1 Hz, 3H, CH_3); ^{13}C NMR (126 MHz, $CDCl_3$): δ 156.8 (br s, CO), 62.3 (CH_2), 14.4 (CH_3); ^{15}N NMR (51 MHz, $CDCl_3$): δ 97 (^{15}NH-CO); IR (neat, cm^{-1}) υ 3228, 2990, 2917, 1745, 1693, 1516, 1481, 1233; HRMS (ESI+) (*m*/*z*): $[M+H]^+$ calcd for $C_6H_{13}(^{15}N)_2O_4$: 179.0811; found: 179.0810.

di-^{15}N-DEAD:
^1H NMR (500 MHz, $CDCl_3$): δ 4.51 (q, J = 7.1 Hz, 2H, CH_2), 1.45 (t, J = 7.1 Hz, 3H, CH_3); ^{13}C NMR (126 MHz, $CDCl_3$): δ 160.3 (dd, J = 2.3, 2.3 Hz, CO), 65.5 (CH_2), 14.0 (CH_3); ^{15}N NMR (51 MHz, $CDCl_3$): δ 150 (^{15}N-CO); IR (neat, cm^{-1}) υ 2987, 1768, 1471, 1368; HRMS (ESI+) (*m*/*z*): $[M+3H]^+$ calcd for $C_6H_{13}(^{15}N)_2O_4$: 179.0811; found: 179.0809.
General procedure for preparation and analysis of betaine intermediates **48**:
A solution of an azo compound (0.04 mmol) in $CDCl_3$ (0.4 mL) was added to an NMR tube under

argon atmosphere, followed by the addition of a solution of triphenylphospine (0.4 mmol) in $CDCl_3$ (0.4 mL). NMR spectra were recorded after 10 minutes.

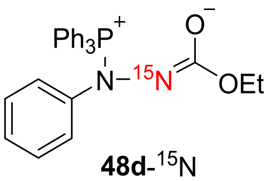

48d-^{15}N

^{1}H NMR (500 MHz, $CDCl_3$): δ 7.91–7.84 (m, 6H, H-2' and H-6'), 7.63–7.57 (m, 3H, H-4'), 7.51–7.40 (m, 6H, H-3' and H-5'), 7.14–7.09 (m, 2H, H-2 and H-6), 7.03–6.97 (m, 2H, H-3 and H-5), 6.91–6.86 (m, 1H, H-4), 3.82 (q, J = 7.0 Hz, 2H, -CH_2CH$_3$), 1.04 (t, J = 7.0 Hz, 3H, CH$_3$); ^{13}C NMR (126 MHz, $CDCl_3$): δ168.1 (d, J = 6.4 Hz, CO) , 144.1 (d, J = 12.1 Hz, C-1), 134.6 (d, J = 10.3 Hz, C-2' and C-6'), 133.3 (d, J = 1.9 Hz, C-4'), 128.9 (d, J = 13.1 Hz, C-3' and C-5'), 128.1 (C-3 and C-5), 124.8 (C-2 and C-6), 124.0 (C-4), 121.7 (d, J = 103.0 Hz, C-1'), 59.1 (CH$_2$), 15.2 (CH$_3$); ^{15}N NMR (51 MHz, $CDCl_3$): δ 182 (^{15}N-CO), 83 (Ar-N); ^{31}P NMR (202 MHz, $CDCl_3$): δ +33.3 (d, J = 5.2 Hz); HRMS (ESI+) (m/z): [M+H]$^+$ calcd for $C_{27}H_{26}N(^{15}N)O_2P$: 442.1697; found: 442.1694.

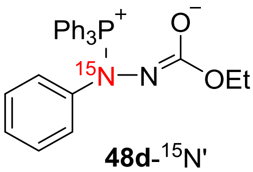

48d-^{15}N'

^{1}H NMR (500 MHz, $CDCl_3$): δ 7.91–7.84 (m, 6H, H-2' and H-6'), 7.61–7.56 (m, 3H, H-4'), 7.51–7.41 (m, 6H, H-3' and H-5'), 7.15–7.10 (m, 2H, H-2 and H-6), 7.03 –6.97 (m, 2H, H-3 and H-5), 6.91–6.86 (m, 1H, H-4), 3.86–3.80 (m, 2H, -CH$_2$), 1.06–1.01 (m, 3H, CH$_3$); ^{13}C NMR (126 MHz, $CDCl_3$): δ 168.1 (d, J = 6.1 Hz, CO), 144.0 (d, J = 12.4 Hz, C-1), 134.6 (d, J = 10.3 Hz, C-2' and C-6'), 133.3 (d, J = 2.3 Hz, C-4'), 128.9 (d, J = 12.9 Hz, C-3' and C-5'), 128.2 (d, J = 7.0 Hz, C-3 and C-5), 124.7 (C-2 and C-6), 124.0 (C-4), 59.1 (CH$_2$), 15.2 (CH$_3$);^{15}N NMR (51 MHz, $CDCl_3$): δ 84 (Ar-^{15}N); ^{31}P NMR (202 MHz, $CDCl_3$): δ +33.4 (d, J = 6.2 Hz); HRMS (ESI+) (m/z): [M+H]$^+$ calcd for $C_{27}H_{26}N(^{15}N)O_2P$: 442.1697; found: 442.1691.

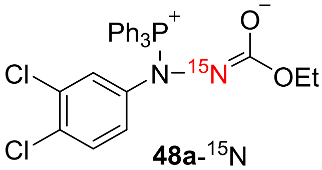

48a-^{15}N

^{1}H NMR (500 MHz, $CDCl_3$): δ 7.91–7.83 (m, 6H, H-2' and H-6'), 7.67–7.62 (m, 3H, H-4'), 7.54–7.49 (m, 6H, H-3' and H-5'), 7.21–7.17 (m, 1H, H-2), 7.02 (d, J = 8.7 Hz, 1H, H-5), 6.86–6.82 (m, 1H, H-6), 3.82 (q, J = 7.0 Hz, 2H, CH$_2$), 1.03 (t, J = 7.0 Hz, 3H, CH$_3$); ^{13}C NMR (126 MHz, $CDCl_3$): δ 168.0 (dd, J_1 = 5.5 Hz, J_2 = 1.8 Hz, CO), 144.1 (d, J = 12.8 Hz, C-1), 134.6 (d, J = 10.5 Hz, C-2' and C-6'), 134.1 (d, J = 2.5 Hz, C-4'), 131.9 (C-3), 129.4 (C-5), 129.1 (d, J = 12.9 Hz, C-3'

and C-5’), 127.1 (C-4), 125.6 (d, J = 3.1 Hz, C-2), 123.1 (d, J = 2.5 Hz, C-6), 122.4 (d, J = 103.3 Hz, C-1’), 59.3 (CH_2), 15.2 (CH_3); ^{15}N NMR (51 MHz, $CDCl_3$): δ 182 (^{15}N-CO); ^{31}P NMR (202 MHz, $CDCl_3$): δ +34.5 (d, J = 5.0 Hz); HRMS (ESI+) (m/z): $[M+H]^+$ calcd for $C_{27}H_{24}Cl_2N(^{15}N)O_2P$: 510.0917; found: 510.0915.

Table S2. Chemical shifts of ^{31}P NMR of betaine **48a** in different solvents

entry	solvent	^{31}P NMR (202 MHz) δ (ppm)
1	THF-d_8	+21.1
2	$CDCl_3$	+34.5
3	CD_3CN	+33.7
4	toluene-d_8	Not detected

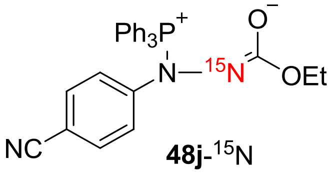

48j-^{15}N

^{1}H NMR (500 MHz, $CDCl_3$): δ 7.92–7.83 (m, 6H, H-2’ and H-6’), 7.71–7.65 (m, 3H, H-4’), 7.56–7.49 (m, 6H, H-3’ and H-5’), 7.27 (d, J = 8.5 Hz, 2H, H-3 and H-5), 7.00 (d, J = 8.5 Hz, 2H, H-2 and H-6), 3.71 (q, J = 7.0 Hz, 2H, -CH_2CH_3), 0.96 (t, J = 7.0 Hz, 3H, CH_3); ^{13}C NMR (126 MHz, $CDCl_3$): δ 168.4 (CO), 149.4 (d, J = 15.7 Hz), 134.7 (d, J = 10.6 Hz, C-2’ and C-6’), 134.2 (d, J = 2.6 Hz, C-4’), 132.0 (C-3 and C-5), 129.2 (d, J = 13.2 Hz, C-3’ and C-5’), 121.7 (d, J = 103.1 Hz, C-1’), 120.6 (d, J = 3.2 Hz, C-2 and C-6), 119.2 (CN), 104.7 (C-4), 59.3 (CH_2), 15.1 (CH_3); ^{15}N NMR (51 MHz, $CDCl_3$): δ 179 (^{15}N-CO), 90 (Ar-N); ^{31}P NMR (202 MHz, $CDCl_3$): δ +35.4 (s); HRMS (ESI+) (m/z): $[M+H]^+$ calcd for $C_{28}H_{25}N_2(^{15}N)O_2P$: 467.1649; found: 467.1642.

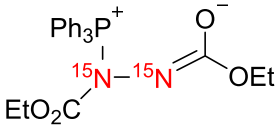

^{1}H NMR (500 MHz, $CDCl_3$): δ 7.97–7.90 (m, 6H, H-2’ and H-6’), 7.71–7.65 (m, 3H, H-4’), 7.58–7.52 (m, 6H, H-3’ and H-5’), 4.16 (q, J = 7.0 Hz, 2H, Ph_3P-N-COOCH_2), 3.74 (q, J = 7.0 Hz, 2H, N=COOCH_2), 1.20 (t, J = 7.0 Hz, 3H, Ph_3P-N-COOCH_2CH_3), 0.94 (t, J = 7.0 Hz, 3H, N=COOCH_2CH_3); ^{13}C NMR (126 MHz, $CDCl_3$): δ 168.2 (N=COOCH_2CH_3), 158.2 (t, J = 23.0 Hz, Ph_3P-N-COOCH_2CH_3), 134.6 (d, J = 10.8 Hz, C-2’ and C-6’), 133.8 (d, J = 2.4 Hz, C-4’), 128.8 (d, J = 13.2 Hz, C-3’ and C-5’), 121.7 (d, J = 103.5 Hz, C-1’), 63.5 (Ph_3P-N-COOCH_2CH_3), 59.5 (N=COOCH_2CH_3), 15.1 (N=COOCH_2CH_3), 14.1 (Ph_3P-N-COOCH_2CH_3); ^{15}N NMR (51 MHz, $CDCl_3$): δ 171 (^{15}N=CO), 113 (Ph_3P-^{15}N); ^{31}P NMR (202 MHz, $CDCl_3$): δ +44.2 (s); HRMS (ESI+) (m/z): $[M+H]^+$ calcd for $C_{24}H_{26}(^{15}N)_2O_4P$: 439.1565; found: 439.1564.

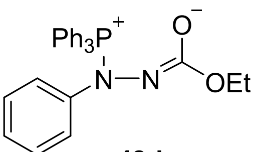

48d

^{1}H NMR (500 MHz, $CDCl_3$): δ 7.92–7.86 (m, 6H, H-2' and H-6'), 7.61–7.56 (m, 3H, H-4'), 7.49–7.41 (m, 6H, H-3' and H-5'), 7.14–7.11 (m, 2H, H-2 and H-6), 7.03–6.98 (m, 2H, H-3 and H-5), 6.91–6.86 (m, 1H, H-4), 3.84 (q, J = 7.1 Hz, 2H, -CH_2), 1.04 (t, J_1 = 7.1 Hz, 3H, CH_3); ^{13}C NMR (126 MHz, $CDCl_3$): δ 168.0 (br s, CO), 144.0 (d, J = 12.4 Hz, C-1), 134.6 (d, J = 10.4 Hz, C-2' and C-6'), 132.9 (C-4'), 128.9 (d, J = 13.0 Hz, C-3' and C-5'), 128.1 (C-3 and C-5), 124.8 (d, J = 3.0 Hz, C-2 and C-6), 124.0 (C-4), 59.2 (CH_2), 15.2 (CH_3), (C-1' overlaid); ^{15}N NMR (51 MHz, $CDCl_3$): δ 83 (Ar-N); ^{31}P NMR (202 MHz, $CDCl_3$): δ +33.9 (s); HRMS (ESI+) (*m/z*): $[M+H]^+$ calcd for $C_{27}H_{26}N_2O_2P$: 441.1718; found: 441.1717.

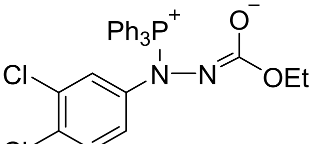

48a

^{1}H NMR (500 MHz, $CDCl_3$): δ 7.91–7.84 (m, 6H, H-2' and H-6'), 7.67–7.62 (m, 3H, H-4'), 7.54–7.48 (m, 6H, H-3' and H-5'), 7.22–7.18 (m, 1H, H-2), 7.02 (d, J = 8.8 Hz, 1H, H-5), 6.86–6.82 (m, 1H, H-6), 3.82 (q, J = 7.1 Hz, 2H, -CH_2), 1.03 (t, J_1 = 7.1 Hz, 3H, CH_3); ^{13}C NMR (126 MHz, $CDCl_3$): δ 168.0 (d, J_1 = 5.5 Hz), 144.1 (d, J = 13.1 Hz, C-1), 134.6 (d, J = 10.6 Hz, C-2' and C-6'), 134.1 (d, J = 2.4 Hz, C-4'), 131.9 (C-3), 129.4 (C-5), 129.2 (d, J = 13.0 Hz, C-3' and C-5'), 127.1 (C-4), 125.6 (d, J = 3.2 Hz, C-2), 123.1 (d, J = 2.7 Hz, C-6), 122.4 (d, J = 103.3 Hz, C-1'), 59.3 (CH_2), 15.2 (CH_3); ^{15}N NMR (51 MHz, $CDCl_3$): δ 85 (Ar-N); ^{31}P NMR (202 MHz, $CDCl_3$): δ +34.5 (s); HRMS (ESI+) (*m/z*): $[M+H]^+$ calcd for $C_{27}H_{24}Cl_2N_2O_2P$: 509.0947; found: 509.0943.

Procedure for methylation of the betaine with MeI:

a) A preparative method

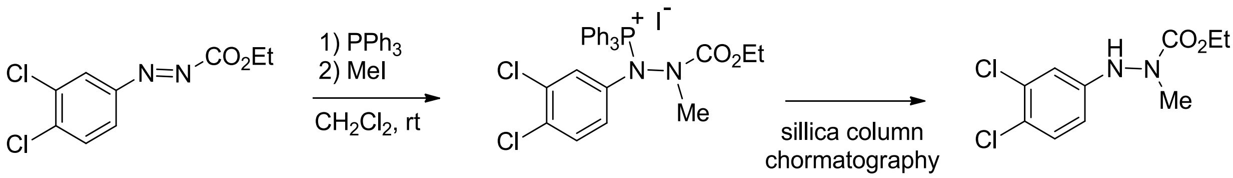

To a solution of ethyl 2-(3,4-dichlorophenyl)diazene-1-carboxylate (**2a**, 247 mg, 1.0 mmol) in dry dichloromethane (distilled over CaH_2), triphenylphosphine (1.05 g, 4.0 mmol) was added under argon atmosphere. The reaction mixture was stirred at room temperature for 5 minutes, and then methyl iodide (710 mg, 5.0 mmol) was added via syringe. After 30 minutes of stirring at room

temperature, the solvent was evaporated under reduced pressure (clean and quantitative conversion of starting diazene). The excess of PPh_3 reacts into phosphonium salt $^{+}PPh_3Me\ ^{-}I$.

Methylated adduct: ^{1}H NMR (500 MHz, $CDCl_3$): δ 7.98–7.89 (m, 6H, H-2' and H-6'), 7.85–7.60 (H-4', H-3' and H-5' resonances overlaid with resonances for $^{+}PPh_3Me\ ^{-}I$), 7.27 (H-5, overlaid), 6.92 (d, J = 2.7 Hz, H-2), 6.71 (dd, J_1 = 8.9, 2.7 Hz, 1H, H-6), 4.04–3.90 (m, -C*H₂*CH₃), 3.38 (br s, 3H, N-CH_3), 1.10–1.02 (m, 3H, -CH_2CH_3); HRMS (ESI+) (m/z): $[M–I]^+$ calcd for $C_{28}H_{26}Cl_2N_2O_2P$: 523.1103; found: 523.1099.

The crude product (methylated adduct) was subjected to a purification on silica gel column chromatography (dichloromethane/methanol/ammonium hydroxide solution 80/10/1) to obtain ethyl 2-(3,4-dichlorophenyl)-1-methylhydrazine-1-carboxylate as a brown oil. IR (neat, cm^{-1}) υ 3213, 3053, 1704, 1594, 1473, 1433, 1373, 1175; ^{1}H NMR (500 MHz, $CDCl_3$): δ 7.27 (d, J = 8.7 Hz, H-5), 6.81 (d, J = 2.4 Hz, H-2), 6.57 (dd, J = 8.7, 2.4 Hz, 1H, H-6), 6.01 (br s, 1H, NH), 4.17 (q, J = 7.0 Hz, CH_2), 3.23 (s, 3H, -N-CH_3), 1.24 (br s, 3H, -CH_2CH_3); ^{13}C NMR (126 MHz, $CDCl_3$): δ 157.0 (CO), 146.8 (C-1), 133.1 (C-3), 130.8 (C-5), 123.6 (C-4), 114.3 (C-2), 112.4 (C-6), 62.5 (CH_2), 37.8 (N-CH_3), 14.6 (CH_3); HRMS (ESI+) (m/z): $[M+H]^+$ calcd for $C_{10}H_{13}Cl_2N_2O_2$: 263.0349; found: 263.0345.

b) The reaction in NMR tube

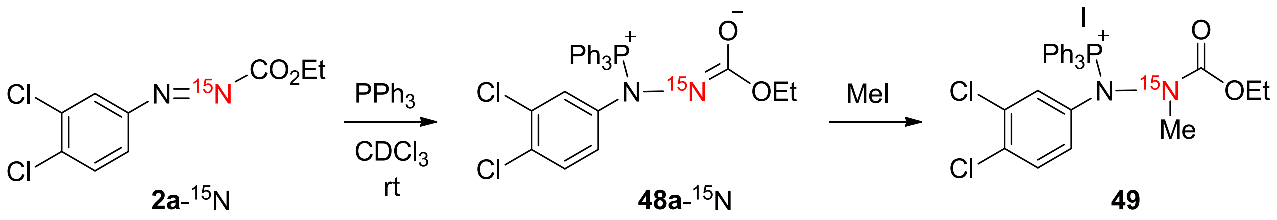

A similar protocol as described above (preparative method) was used for the NMR experiment with **2a**-^{15}N in $CDCl_3$ in an NMR tube.

49: ^{1}H NMR (500 MHz, $CDCl_3$): δ 7.90–7.40 (N-$^{+}P(C_6H_5)_3$ resonances overlaid with resonances for $^{+}PPh_3Me\ ^{-}I$), 7.18 (H-5, overlaid), 6.82 (d, J = 2.7 Hz, 1H, H-2), 6.64 (dd, J = 8.8, 2.7 Hz, 1H, H-6), 3.94–3.78 (m, 2H, -CH_2CH_3), 3.27 (br s, 3H, N-CH_3), 1.02–0.90 (m, 3H, CH_3); ^{13}C NMR (126 MHz, $CDCl_3$): δ 155.2 (br s, CO), 137.8 (d, J = 13.7 Hz, C-1), 119.6 (C-2), 118.1 (C-6), 63.4 (CH_2), 38.3 (N-CH_3), 13.6 (CH_3), (C-3, C-4, C-5, N-$^{+}P(C_6H_5)_3$ resonances overlaid); ^{15}N NMR (51 MHz, $CDCl_3$): δ 109 (^{15}N-CO), 89 (Ar-N); ^{31}P NMR (202 MHz, $CDCl_3$): δ +51.1 (s); HRMS (ESI+) (m/z): $[M–I]^+$ calcd for $C_{28}H_{26}Cl_2N(^{15}N)O_2P$: 524.1074; found: 524.1070.

Table S3: NMR data of ethyl 2-arylazocarboxylates (2)

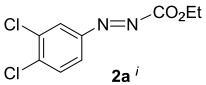
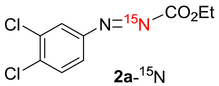
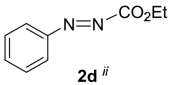
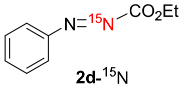
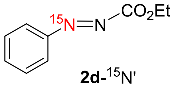
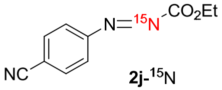

Atom	**2a** [i]	**2a**-^{15}N	**2d** [ii]	**2d**-^{15}N	**2d**-^{15}N'	**2j**-^{15}N
*C*H$_3$	1.48 (t, J = 7.2 Hz, 3H)	1.47 (t, J = 7.1 Hz, 3H)	1.47 (t, J = 7.1 Hz, 3H)	1.47 (t, J = 7.1 Hz, 3H)	1.48 (t, J = 7.1 Hz, 3H)	1.48 (t, J = 7.2 Hz, 3H)
*C*H$_2$	4.53 (q, J = 7.2 Hz, 2H)	4.53 (q, J = 7.1 Hz, 2H)	4.52 (q, J = 7.1 Hz, 2H)	4.52 (q, J = 7.1 Hz, 2H)	4.53 (q, J = 7.1 Hz, 2H)	4.55 (q, J = 7.2 Hz, 2H)
H2	8.02 (d, $J_{H2–H6}$ = 2.3 Hz, 1H)	8.02 (d, $J_{H2–H6}$ = 2.2 Hz, 1H)	7.92–7.96 (m, 1H)	7.91–7.96 (m, 1H)	7.92–7.96 (m, 1H)	8.00 (d, $J_{H2–H3}$ = 8.5 Hz, 1H)
H3	–	–	7.51–7.56 (m, 1H)	7.50–7.56 (m, 1H)	7.50–7.56 (m, 1H)	7.85 (d, $J_{H2–H3}$ = 8.5 Hz, 1H)
H4	–	–	7.57–7.61 (m, 1H)	7.56–7.61 (m, 1H)	7.57–7.62 (m, 1H)	–
H5	7.64 (d, $J_{H5–H6}$ = 8.5 Hz, 1H)	7.63 (d, $J_{H5–H6}$ = 8.6 Hz, 1H)	7.51–7.56 (m, 1H)	7.50–7.56 (m, 1H)	7.50–7.56 (m, 1H)	7.85 (d, $J_{H5–H6}$ = 8.5 Hz, 1H)
H6	7.81 (dd, $J_{H5–H6}$ = 8.5 Hz, $J_{H2–H6}$ = 2.3 Hz, 1H)	7.81 (dd, $J_{H5–H6}$ = 8.6 Hz, $J_{H2–H6}$ = 2.2 Hz, 1H)	7.92–7.96 (m, 1H)	7.91–7.96 (m, 1H)	7.92–7.96 (m, 1H)	8.00 (d, $J_{H5–H6}$ = 8.5 Hz, 1H)
*C*H$_3$	14.2 (s)	14.1 (s)	14.1 (s)	14.1 (s)	14.1 (s)	14.1 (s)
*C*H$_2$	64.8 (s)	64.8 (s)	64.5 (s)	64.4 (s)	64.5 (s)	65.0 (s)
CO	161.7 (s)	161.6 (s)	162.2 (s)	162.2 (s)	162.2 (d, $J_{CO\text{-}ArN}$ = 6.3 Hz)	161.6 (s)
C1	150.3 (s)	150.3 (d, $J_{C1\text{-}NCO}$ = 5.3 Hz)	151.6 (s)	151.6 (d, $J_{C1\text{-}NCO}$ = 4.9 Hz)	151.6 (d, $J_{C1\text{-}ArN}$ = 2.8 Hz)	153.1 (d, $J_{C1\text{-}NCO}$ = 5.3 Hz)
C2	124.6 (s)	124.6 (d, $J_{C2\text{-}NCO}$ = 4.1 Hz)	123.7 (s)	123.7 (d, $J_{C2\text{-}NCO}$ = 3.8 Hz)	123.7 (d, $J_{C2\text{-}ArN}$ = 4.3 Hz)	124.0 (d, $J_{C2\text{-}NCO}$ = 4.1 Hz)
C3	134.0 (s)	134.0 (s)	129.3 (s)	129.3 (s)	129.3 (d, $J_{C3\text{-}ArN}$ = 1.8 Hz)	133.4 (s)
C4	138.1 (s)	138.1 (s)	133.8 (s)	133.8 (s)	133.8	116.7 (s)
C5	131.2 (s)	131.2 (s)	129.3 (s)	129.3 (s)	129.3 (d, $J_{C3\text{-}ArN}$ = 1.8 Hz)	133.4 (s)
C6	123.6 (s)	123.6 (d, $J_{C6\text{-}NCO}$ = 3.9 Hz)	123.7 (s)	123.7 (d, $J_{C6\text{-}NCO}$ = 3.8 Hz)	123.7 (d, $J_{C6\text{-}ArN}$ = 4.3 Hz)	124.0 (d, $J_{C6\text{-}NCO}$ = 4.1 Hz)
CN	–	–	–	–	–	117.8
ArN	142	ND	149	149	149	145
NCO	ND	114	ND	107	ND	125

[i]Prepared by the literature procedure.[2a] [ii]Prepared by the literature procedure.[2b]

Table S4: NMR data of betaine intermediates 48

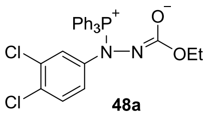
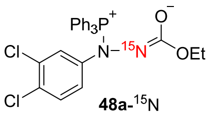
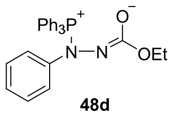
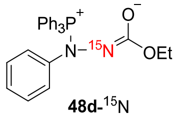
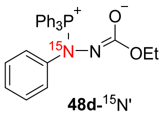
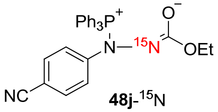

Atom	**48a**	**48a-^{15}N**	**48d**	**48d-^{15}N**	**48d-^{15}N'**	**48j-^{15}N**
CH_3	1.03 (t, J = 7.1 Hz, 3H)	1.03 (t, J = 7.0 Hz, 3H)	1.04 (t, J = 7.1 Hz, 3H)	1.04 (t, J = 7.0 Hz, 3H)	1.01–1.06 (m, 3H)	0.96 (t, J = 7.0 Hz, 3H)
CH_2	3.82 (q, J = 7.1 Hz, 2H)	3.82 (q, J = 7.0 Hz, 2H)	3.84 (q, J = 7.0 Hz)	3.82 (q, J = 7.0 Hz)	3.80–3.86 (m, 3H)	3.71 (q, J = 7.0 Hz, 2H)
H2	7.18–7.22 (m, 1H)	7.17–7.21 (m, 1H)	7.11–7.14 (m, 1H)	7.09–7.14 (m, 1H)	7.10–7.15 (m, 1H)	7.00 (d, $J_{H2–H3}$ = 8.5 Hz, 1H)
H3	–	–	6.98–7.03 (m, 1H)	6.97–7.03 (m, 1H)	6.97–7.03 (m, 1H)	7.27 (d, $J_{H2–H3}$ = 8.5 Hz, 1H)
H4	–	–	6.86–6.91 (m, 1H)	6.86–6.91 (m, 1H)	6.86–6.91 (m, 1H)	–
H5	7.02 (d, $J_{H5–H6}$ = 8.8 Hz, 1H)	7.02 (d, $J_{H5–H6}$ = 8.7 Hz, 1H)	6.98–7.03 (m, 1H)	6.97–7.03 (m, 1H)	6.97–7.03 (m, 1H)	7.27 (d, $J_{H5–H6}$ = 8.5 Hz, 1H)
H6	6.82–6.86 (m, 1H)	6.82–6.86 (m, 1H)	7.11–7.14 (m, 1H)	7.09–7.14 (m, 1H)	7.10–7.15 (m, 1H)	7.00 (d, $J_{H5–H6}$ = 8.5 Hz, 1H)
H2',H6'	7.84–7.91 (m, 6H)	7.83–7.91 (m, 6H)	7.86–7.92 (m, 6H)	7.84–7.91 (m, 6H)	7.84–7.91 (m, 6H)	7.83–7.92 (m, 6H)
H3',H5'	7.48–7.54(m, 6H)	7.49–7.54 (m, 6H)	7.41–7.49 (m, 6H)	7.40–7.51 (m, 6H)	7.41–7.51 (m, 6H)	7.49–7.56 (m, 6H)
H4'	7.62–7.67 (m, 3H)	7.62–7.67 (m, 3H)	7.56–7.61 (m, 3H)	7.57–7.63 (m, 3H)	7.56–7.61 (m, 3H)	7.65–7.71 (m, 3H)
CH_3	15.2 (s)	15.2 (s)	15.2 (s)	15.2 (s)	15.2 (s)	15.1 (s)
CH_2	59.3 (s)	59.3 (s)	59.2 (s)	59.1 (s)	59.1 (s)	59.3 (s)
CO	168.0 (d, J_{CO-P} = 5.5 Hz)	168.0 (dd, J = 5.5, 1.8 Hz)	168.0 (br s)	168.1 (d, J_{CO-P} = 6.4 Hz)	168.1 (d, J_{CO-P} = 6.1 Hz)	168.4 (br s)
C1	144.1 (d, J_{C1-P} = 13.1 Hz)	144.1 (d, J_{C1-P} = 12.8 Hz)	144.0 (d, J_{C1-P} = 12.4 Hz)	144.1 (d, J_{C1-P} = 12.1 Hz)	144.0 (dd, J_{C1-P} = 12.4 Hz, J_{C1-N} = 12.4 Hz)	149.4 (d, J_{C1-P} = 15.7 Hz)
C2	125.6 (d, J = 3.2 Hz)	125.6 (d, J = 3.1 Hz)	124.8 (d, J = 3.0 Hz)	124.8 (s)	124.7 (br s)	120.6 (d, J = 3.2 Hz)
C3	131.9 (s)	131.9 (s)	128.1 (s)	128.1 (s)	128.2 (d, J_{C3-N} = 7.0 Hz)	132.0 (s)
C4	127.1 (s)	127.1 (s)	124.0 (s)	124.0 (s)	124.0 (s)	104.7 (s)
C5	129.4 (s)	129.4 (s)	128.1 (s)	128.1 (s)	128.2 (d, J_{C5-N} = 7.0 Hz)	132.0 (s)
C6	123.1 (d, J = 2.7 Hz)	123.1 (d, J = 2.5 Hz)	124.8 (d, J = 3.0 Hz)	124.8 (s)	124.7 (br s)	120.6 (d, J = 3.2 Hz)
C1'	122.4 (d, J_{P-C} = 103.3 Hz)	122.4 (d, J_{P-C} = 103.3 Hz)	ND	121.7 (d, J_{P-C} = 103.0 Hz)	ND	121.7 (d, J_{P-C} = 103.1 Hz)
C2',C6'	134.6 (d, J_{P-C} = 10.6 Hz)	134.6 (d, J_{P-C} = 10.5 Hz)	134.6 (d, J = 10.4 Hz)	134.6 (d, J_{P-C} = 10.3 Hz)	134.6 (d, J_{P-C} = 10.3 Hz)	134.7 (d, J_{P-C} = 10.6 Hz)
C3',C5'	129.2 (d, J_{P-C} = 13.0 Hz)	129.1 (d, J_{P-C} = 12.9 Hz)	128.9 (d, J = 13.0 Hz)	128.9 (d, J_{P-C} = 13.1 Hz)	128.9 (d, J_{P-C} = 12.9 Hz)	129.2 (d, J_{P-C} = 13.2 Hz)
C4'	134.1 (d, J_{P-C} = 2.4 Hz)	134.1 (d, J_{P-C} = 2.5 Hz)	132.9 (br s)	133.3 (d, J_{P-C} = 1.9 Hz)	133.3 (d, J_{P-C} = 2.3 Hz)	134.2 (d, J_{P-C} = 2.6 Hz)
CN	–	–	–	–	–	119.2
P	34.5 (s)	34.5 (d, J_{P-NCO} = 5.0 Hz)	33.9 (s)	33.3 (d, J_{P-NCO} = 5.2 Hz)	33.4 (d, J_{P-NCO} = 6.2 Hz)	35.4 (s)
ArN	85	ND	83	83	84	90
NCO	ND	182	ND	182	ND	179

*Detection of di-n-butoxytriphenylphosphorane (**54**) by ^{31}P NMR analysis in several solvents:*

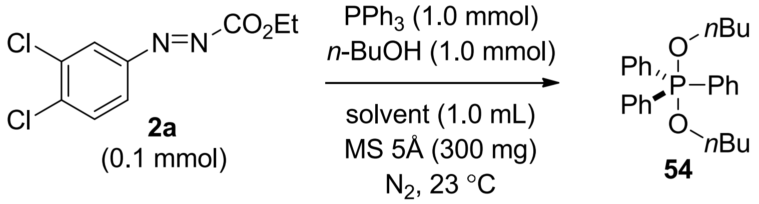

A solution of ethyl 2-(3,4-dichlorophenyl)azocarboxylate (**2a**) (24.7 mg, 0.1 mmol), *n*-BuOH (74.1 mg, 1.0 mmol) and powder of activated molecular sieves 5Å (300 mg) in THF-d_8 (1.0 mL) was stirred in a 10 mL round-bottom flask for 30 min. Triphenylphosphine (262 mg, 1.0 mmol) was added to the stirred solution at 23 °C under N_2 atmosphere. A small amount (ca. 0.5 mL) of a sample was taken from the reaction mixture, and rapidly filtered through a cotton plug. The obtained clear solution was analyzed by ^{31}P NMR.

Table S5. Chemical shifts of di-*n*-butoxytriphenylphosphorane in ^{31}P NMR spectrum

entry	solvent	^{31}P NMR (243 MHz) δ (ppm)
1	THF-d_8	–56.0 (–55.0)[19]
2	$CDCl_3$	–55.3
3	CD_3CN	–55.2
4	toluene-d_8	–55.8

4. Thermal analysis of azo and hydrazine compounds

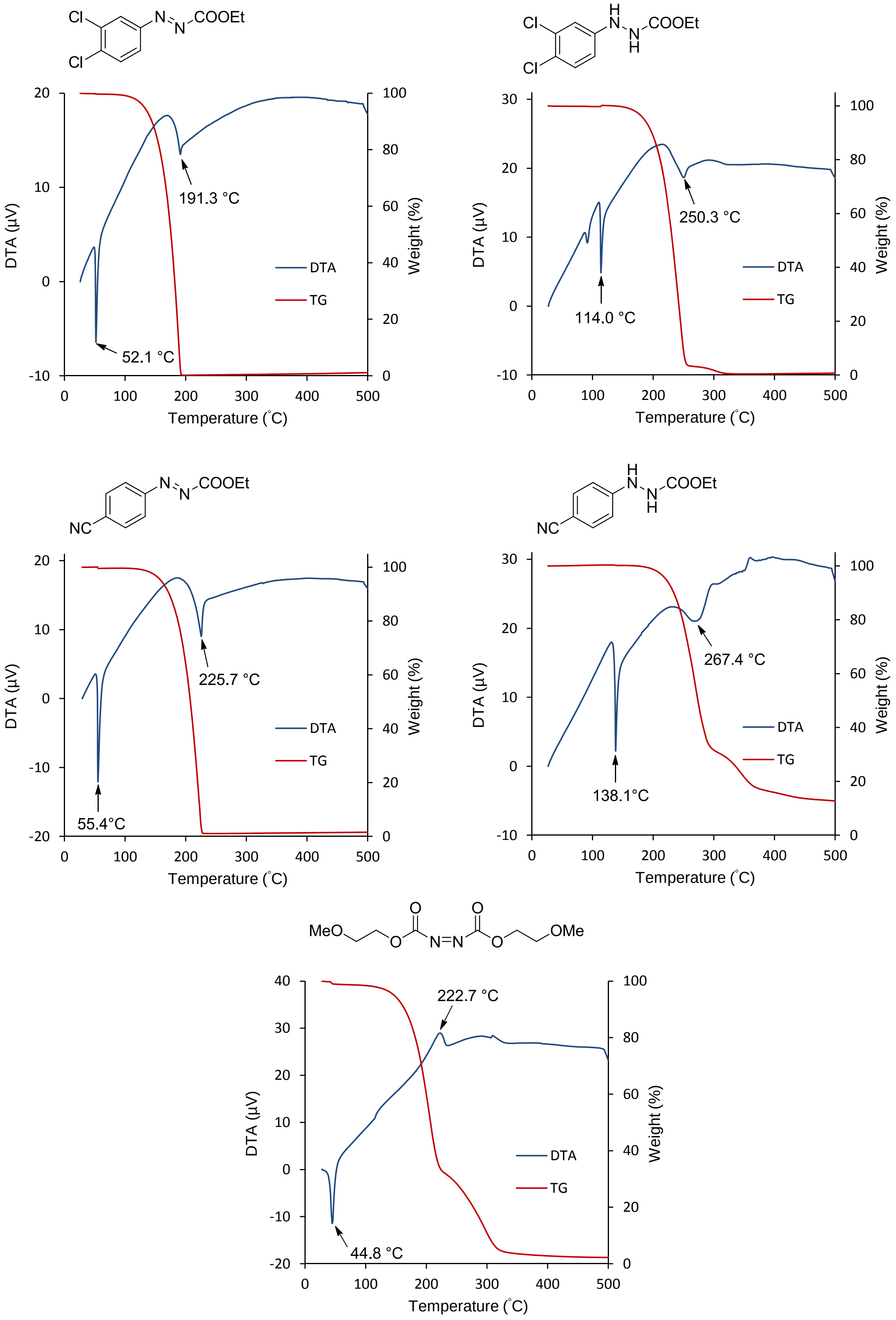

Figure S15. TG-DTA data of azo and hydrazine compounds.

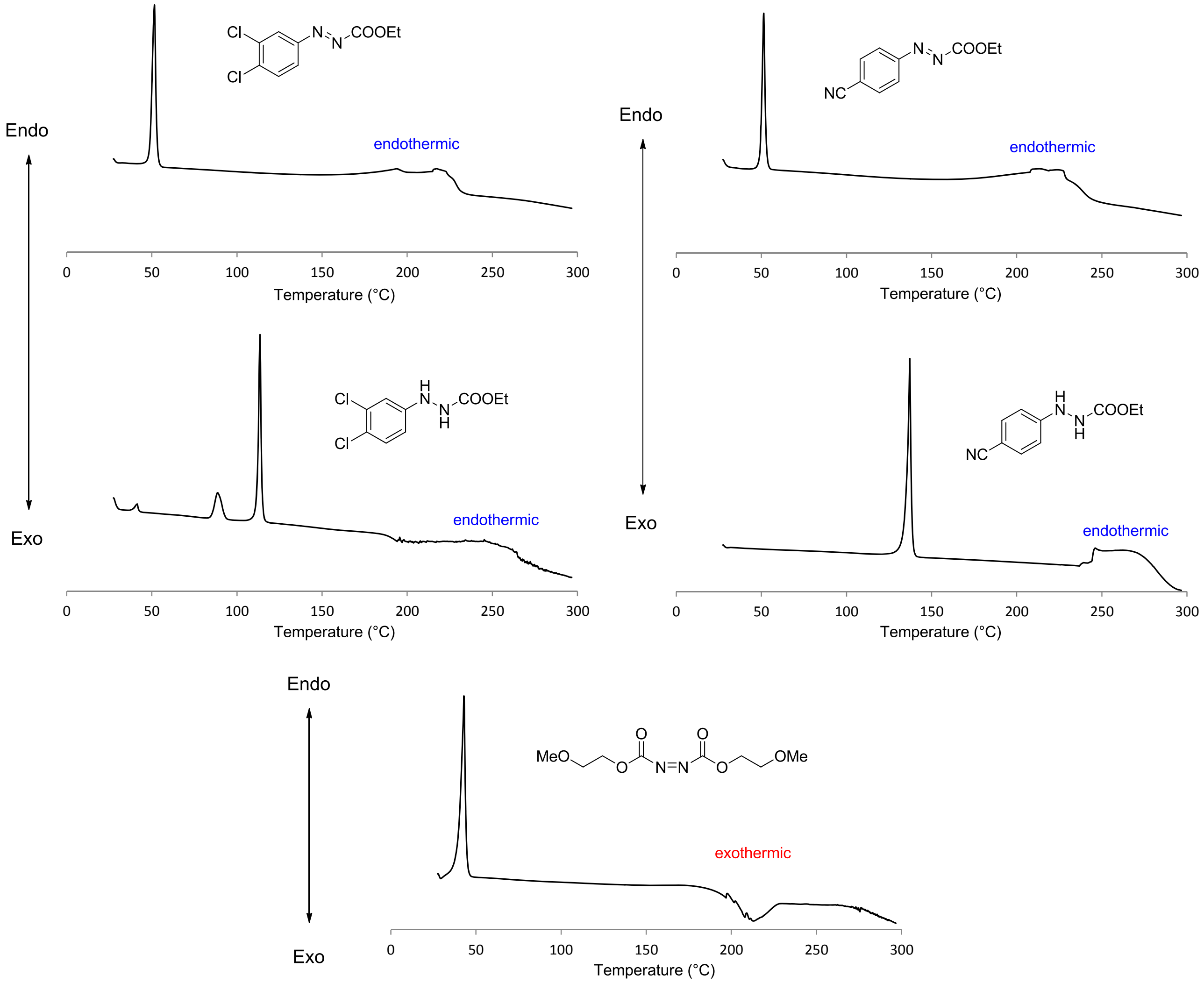

Figure S16. Preliminary DSC data of azo and hydrazine compounds.

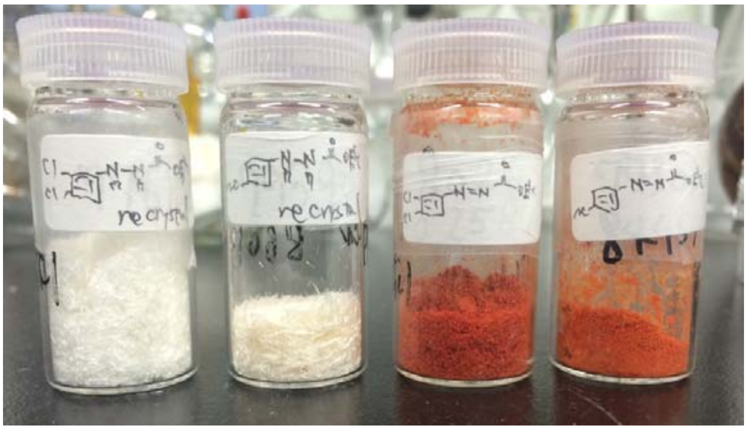

Figure S17. Photos of Mitsunobu reagents (from left to right,**1a**, **1j**, **2a**, **2j**). No decomposition of these reagents was observed after two months under ambient conditions unlike DMEAD (See the NMR spectra data).

*Procedure for thermal analysis of **2a** in a solution-phase:*

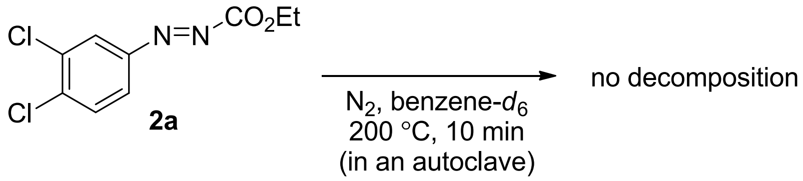

A solution of ethyl 2-(3,4-dichlorophenyl)azocarboxylate (**2a**) (9.4 mg, 0.038 mmol) in benzene-d_6 (1 mL) was placed into an autoclave under an nitrogen atmosphere. Heating the autoclave was begun with a pre-heating oil bath (180 ˚C). The temperature was raised to 200 ˚C over 15 min, and the autoclave was further heated for 10 min at the same temperature. After the autoclave was cooled to room temperature and opened, the solution was analyzed by ^{1}H NMR (400 MHz) analysis. An amount of **2a** was estimated by integration values in the presence of 1,3,5-trioxane as an internal standard (full relaxation was confirmed). Neither loss of **2a** nor detection of any impurities was observed.

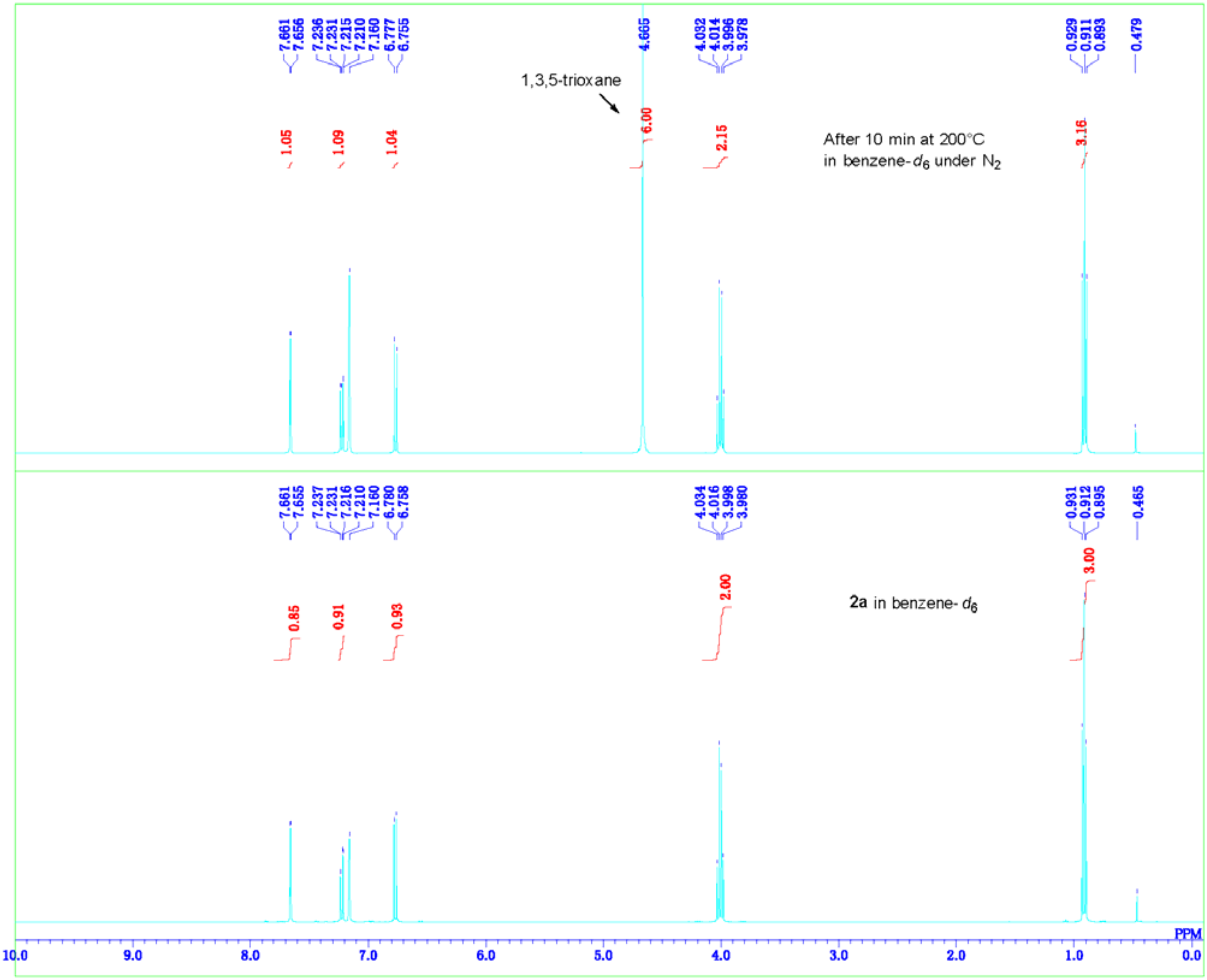

Figure S18. ^{1}H NMR spectra of **2a** after heating at 200 °C for 10 min (top), and before heating (bottom).

5. Photos of the typical experiment of the catalytic Mitsunobu reaction on 10 mmol scale (Table 3, entry 7)

(a) Activation of MS 5Å by a heat gun in vacuo

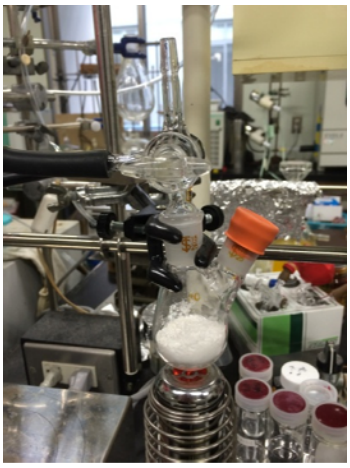

(b) The reaction mixture

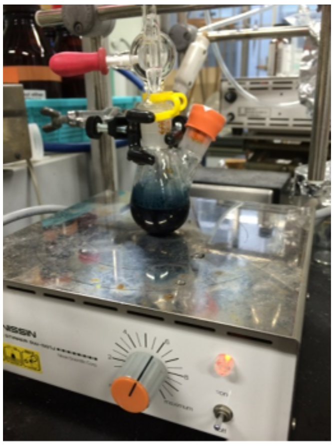

(c) The reaction mixture after filtration with Celite®

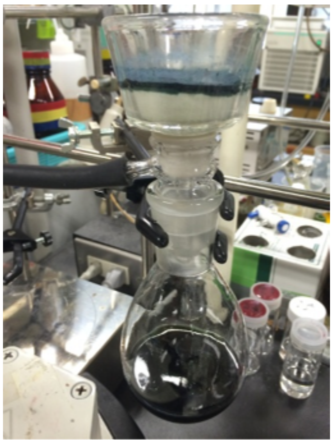

(d) Silica gel column chromatography (yellow elution is azo compound)

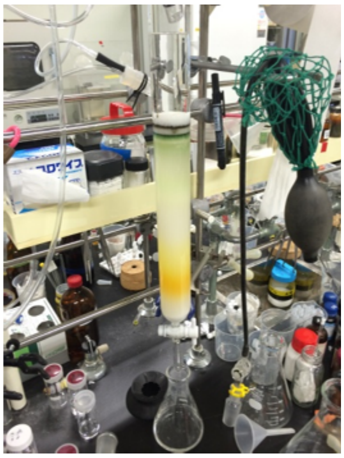

(e) After Silica gel column chromatography (left elute: **2a**, right elute: **5**)

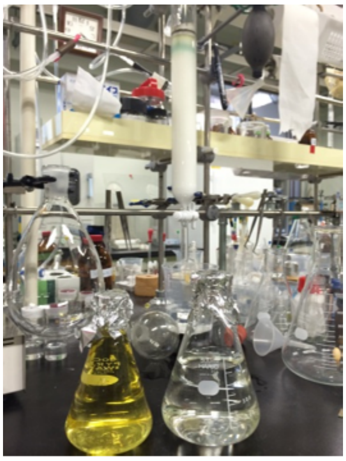

5: 88% yield (2.35 g, 8.8 mmol, 99:1 er)
2a: 81% recovery (200 mg, 0.81 mmol)

(f) TLC at 0, 24, 62, 72 h

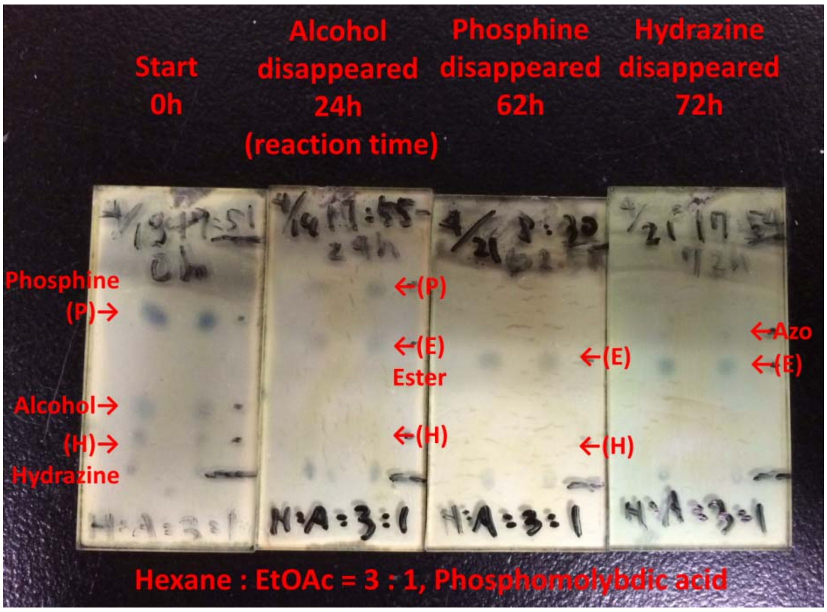

References

(1) D. Hirose, T. Taniguchi and H. Ishibashi, *Angew. Chem., Int. Ed.*, 2013, **52**, 4613–4617.

(2) (a) T. Hashimoto, D. Hirose and T. Taniguchi, *Adv. Synth. Catal.*, 2015, **357**, 3346–3352; (b) D. Urankar, M. Steinbücher, J. Kosjek and J. Košmrlj, *Tetrahedron*, 2010, **66**, 2602–2613.

(3) T. Taniguchi, D. Hirose and H. Ishibashi, *ACS Catal.*, 2011, **1**, 1469–1474.

(4) P. I. Jolly, S. Zhou, D. W. Thomson, J. Garnier, J. A. Parkinson, T. Tuttle and J. A. Murphy, *Chem. Sci.*, 2012, **3**, 1675–1679.

(5) K. Kitahara, T. Toma, J. Shimokawa and T. Fukuyama, *Org. Lett.*, 2008, **10**, 2259.

(6) K. Kuroda, Y. Maruyama, Y. Hayashi and T. Mukaiyama, *Bull. Chem. Soc. Jpn.*, 2009, **82**, 381–392.

(7) Y. Kato, D. H. Yen, Y. Fukudome, T. Hata and H. Urabe, *Org. Lett.*, 2010, **12**, 4137–4139.

(8) A. Poloukhtine, V. Rassadin, A. Kuzmin, V. V. Popik, *J. Org. Chem.*, 2010, **75**, 5953−5962.

(9) T. Kan, A. Fujiwara, H. Kobayashi and T. Fukuyama, *Tetrahedron*, 2002, **58**, 6267−6276.

(10) J. McNulty, A. Capretta, V. Laritchev, J. Dyck and A. J. Robertson, *Angew. Chem., Int. Ed.*, 2003, **42**, 4051−4054.

(11) A. G. M. Barrett, D. C. Braddock, R. A. James, N. Koike and P. A. Procopiou, *J. Org. Chem.*, 1998, **63**, 6273−6280.

(12) C. Chen, S. F. Zhu, B. Liu, L. X. Wang and Q. L. Zhou, *J. Am. Chem. Soc.*, 2007, **129**, 12616−12617.

(13) L. D. Arnold, H. I. Assil and J. C. Vederas, *J. Am. Chem. Soc.*, 1989, **111**, 3973−3976.

(14) J. C. Poupon, A. A. Boezio and A. B. Charette, *Angew. Chem., Int. Ed.*, 2006, **45**, 1415−1420.

(15) X. Tang, C. Chapman, M. Whiting and R. Denton, *Chem Commun.*, 2014, **50**, 7340−7343.

(16) T. Y. S. But and P. H. Toy, *J. Am. Chem. Soc.*, 2006, **128**, 9636−9637.

(17) T. Y. S. But, J. Lu and P. H. Toy, *Synlett*, 2010, 1115−1117.

(18) A. Zhang and A. D. Schluter, *Chem. Asian J.*, 2007, **2**, 1540−1548.

(19) E. Grochowski, B. D. Hilton, R. J. Kupper and C. J. Michejda, *J. Am. Chem. Soc.*, 1982, **104**, 6876−6877.

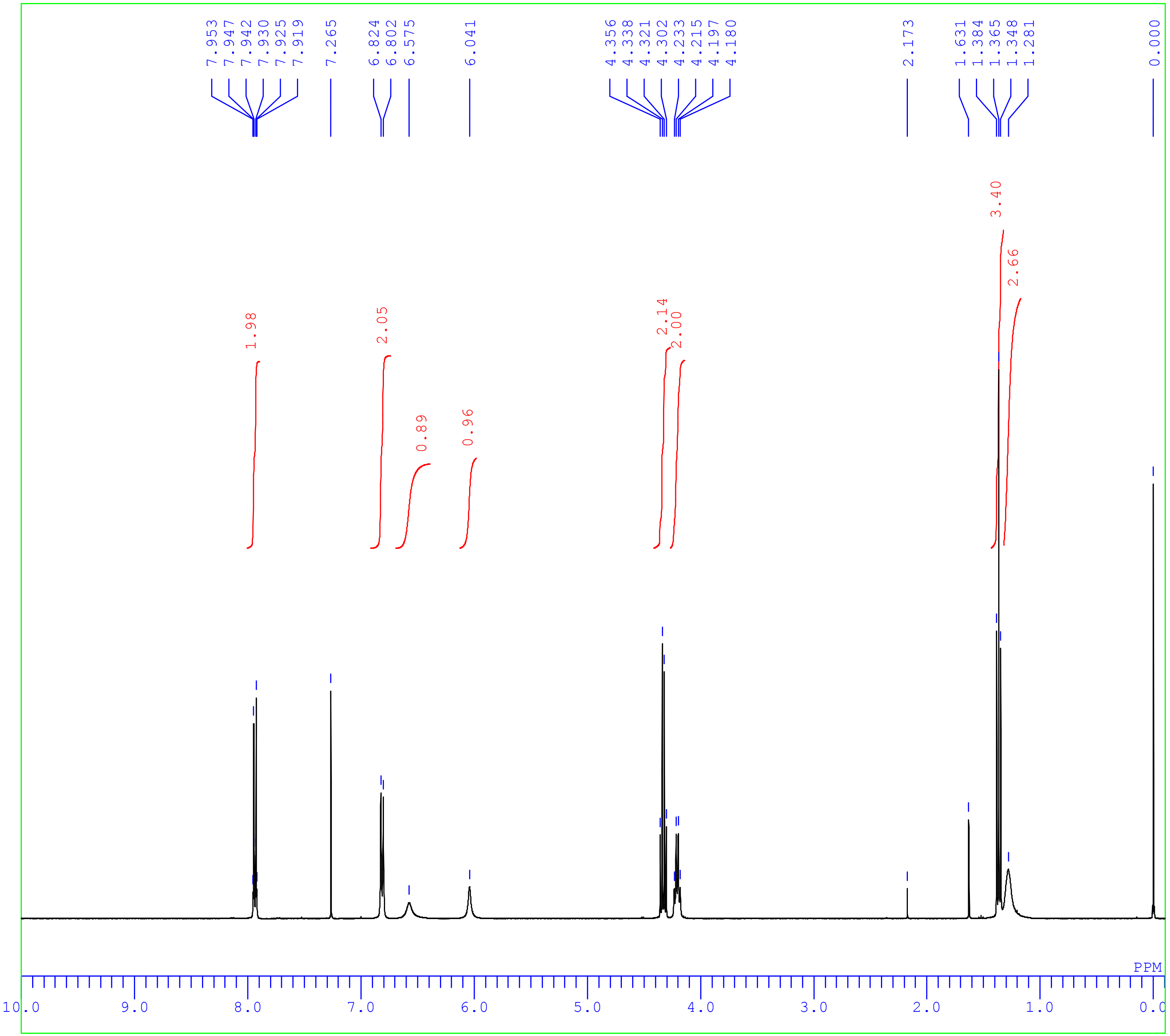

DFILE	d1892-1H-data2-1.als
COMNT	160330
DATIM	2016-03-30 18:26:45
OBNUC	1H
EXMOD	single_pulse.ex2
OBFRQ	399.78 MHz
OBSET	4.19 KHz
OBFIN	7.29 Hz
POINT	13107
FREQU	6002.31 Hz
SCANS	32
ACQTM	2.1837 sec
PD	2.0000 sec
PW1	4.70 usec
IRNUC	1H
CTEMP	20.4 c
SLVNT	CDCL3
EXREF	0.00 ppm
BF	0.12 Hz
RGAIN	40

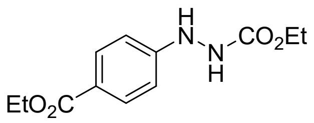

1h

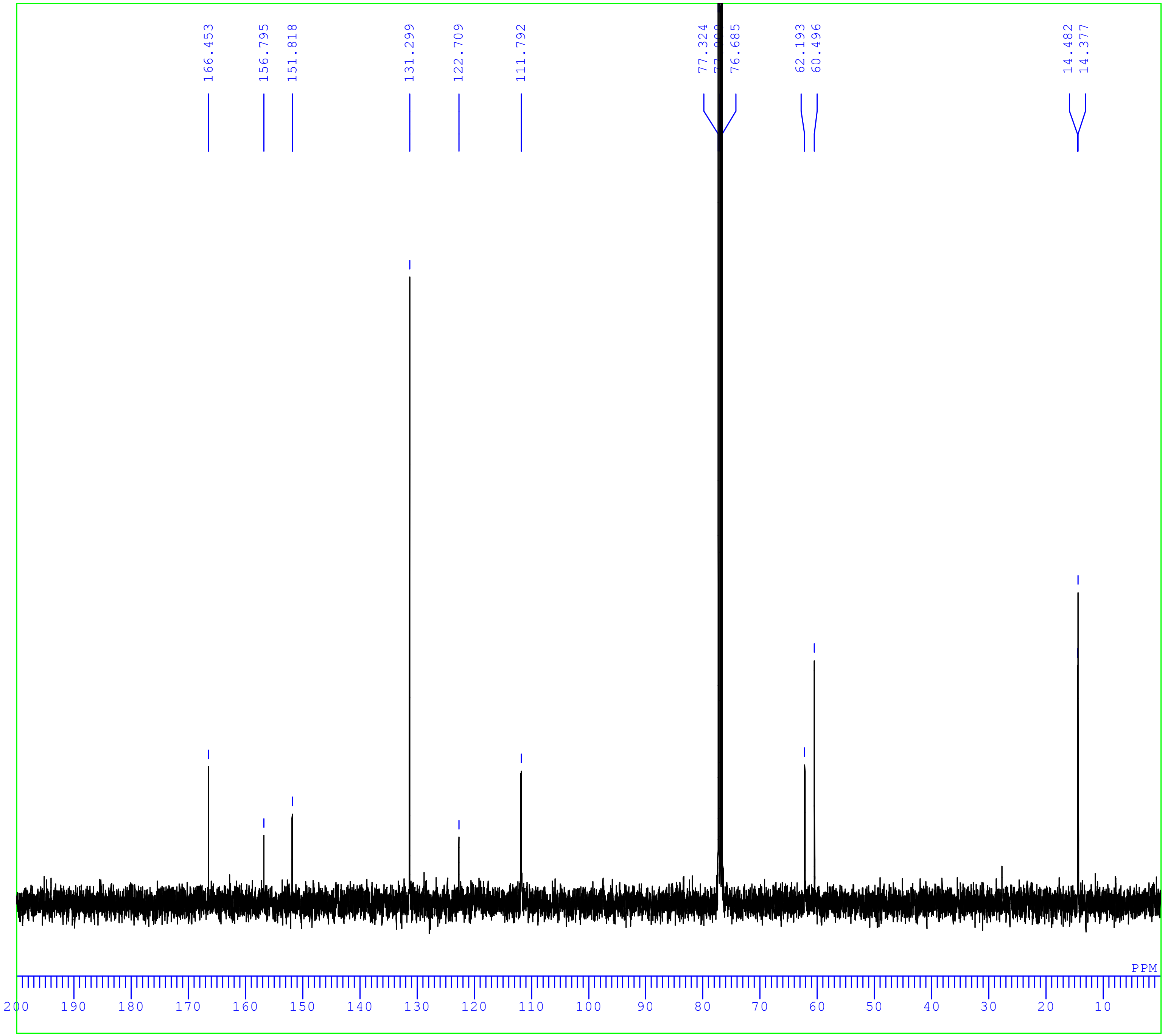

DFILE d1892-13C-data2-1.als
COMNT 160330
DATIM 2016-03-30 18:37:18
OBNUC 13C
EXMOD single_pulse_dec
OBFRQ 100.53 MHz
OBSET 5.35 KHz
OBFIN 5.86 Hz
POINT 26214
FREQU 25125.24 Hz
SCANS 256
ACQTM 1.0433 sec
PD 1.2000 sec
PW1 2.87 usec
IRNUC 1H
CTEMP 20.7 c
SLVNT CDCL3
EXREF 77.00 ppm
BF 1.20 Hz
RGAIN 60

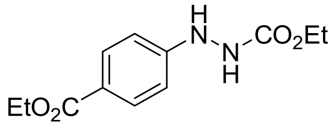

1h

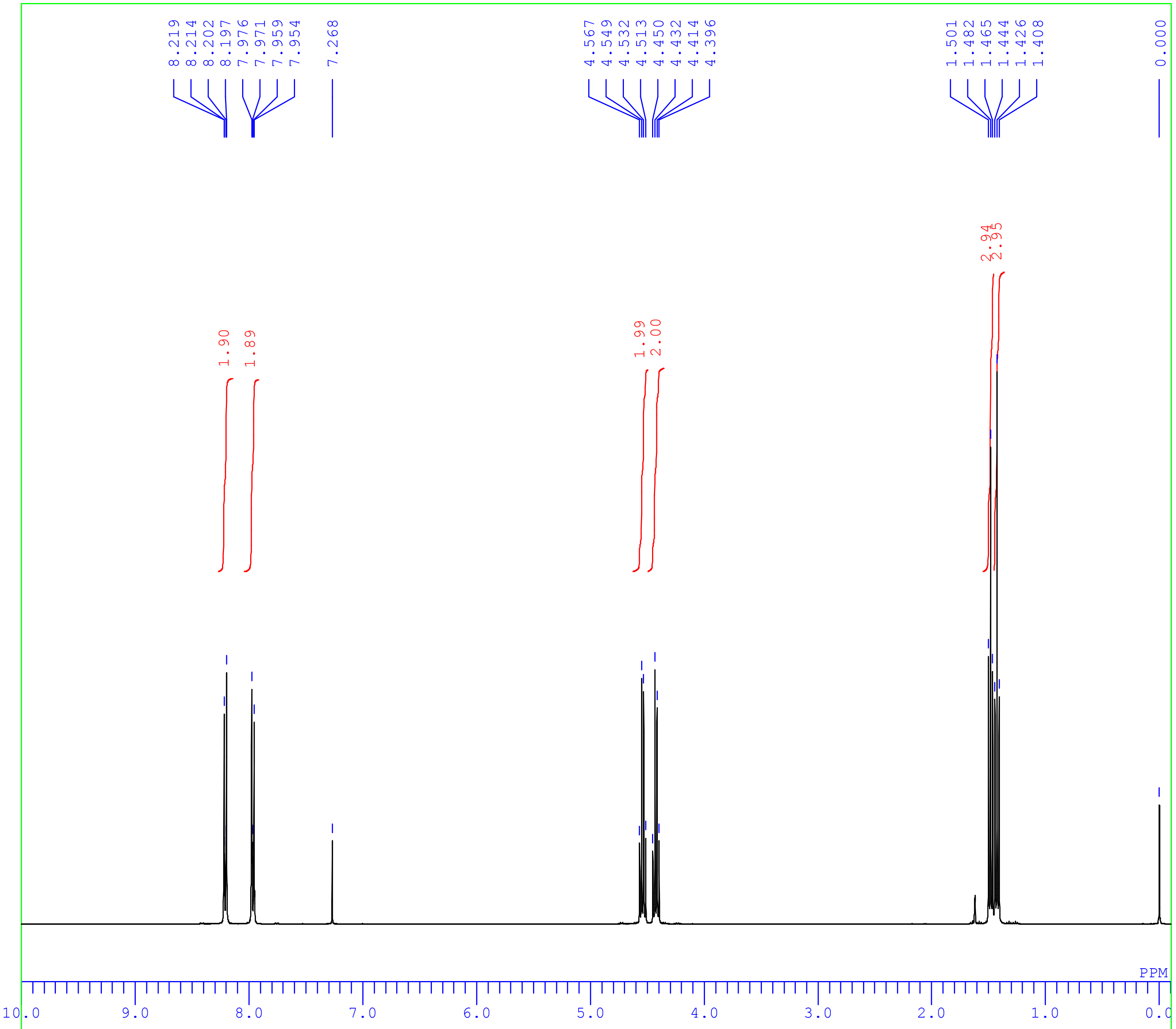

DFILE	d1898-1H-data2-1.jdf
COMNT	160330
DATIM	2016-03-30 18:45:35
OBNUC	1H
EXMOD	single_pulse.ex2
OBFRQ	399.78 MHz
OBSET	4.19 KHz
OBFIN	7.29 Hz
POINT	16384
FREQU	7503.00 Hz
SCANS	32
ACQTM	2.1837 sec
PD	2.0000 sec
PW1	4.70 usec
IRNUC	1H
CTEMP	20.3 c
SLVNT	CDCL3
EXREF	0.00 ppm
BF	0.12 Hz
RGAIN	38

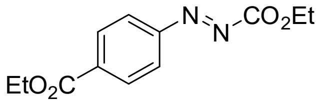

2h

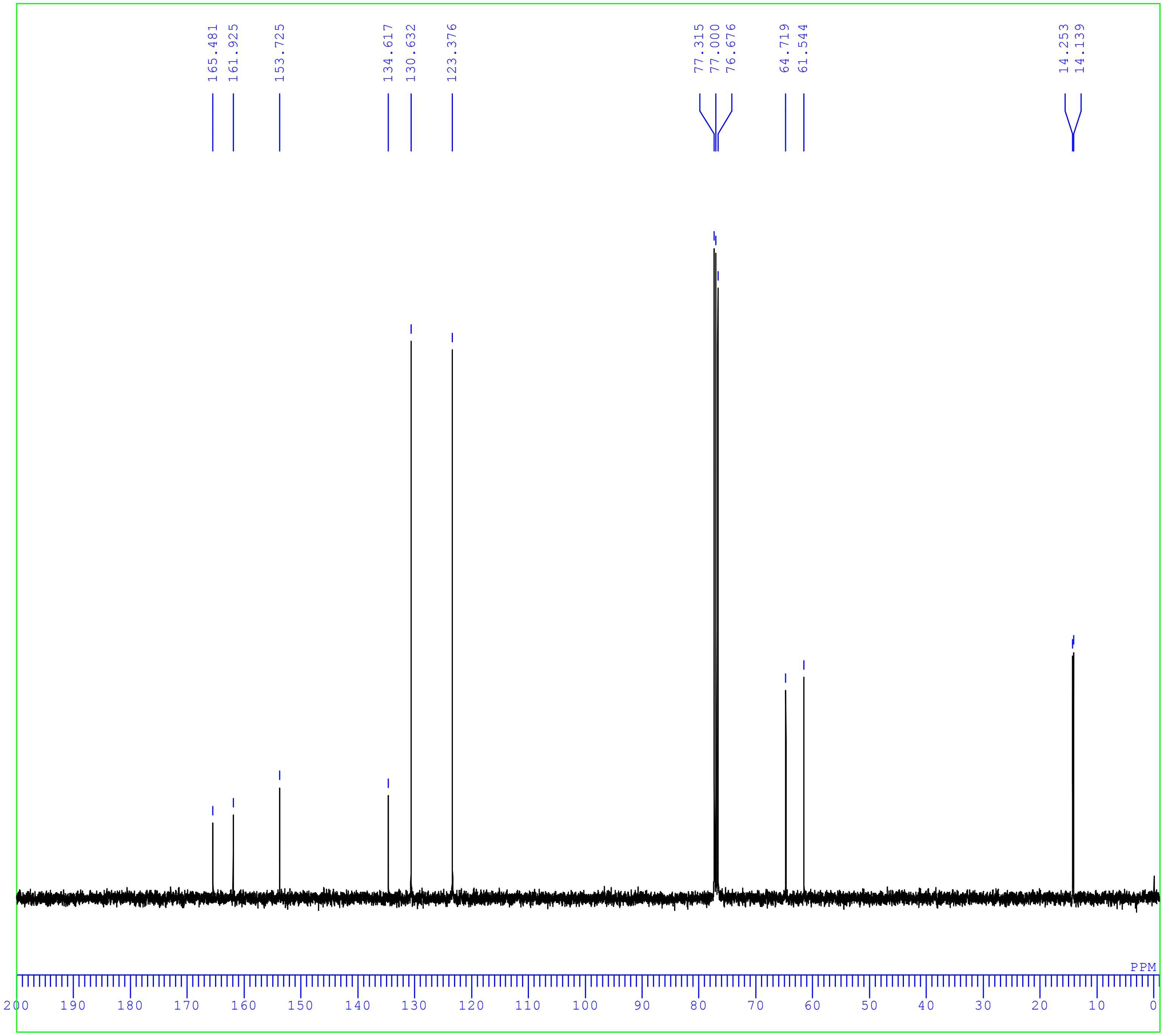

DFILE	d1898-13C-data2-1.als
COMNT	160330
DATIM	2016-03-30 18:55:46
OBNUC	13C
EXMOD	single_pulse_dec
OBFRQ	100.53 MHz
OBSET	5.35 KHz
OBFIN	5.86 Hz
POINT	26214
FREQU	25125.24 Hz
SCANS	256
ACQTM	1.0433 sec
PD	1.2000 sec
PW1	2.87 usec
IRNUC	1H
CTEMP	20.6 c
SLVNT	CDCL3
EXREF	77.00 ppm
BF	1.20 Hz
RGAIN	60

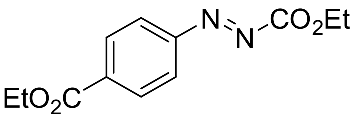

2h

150701

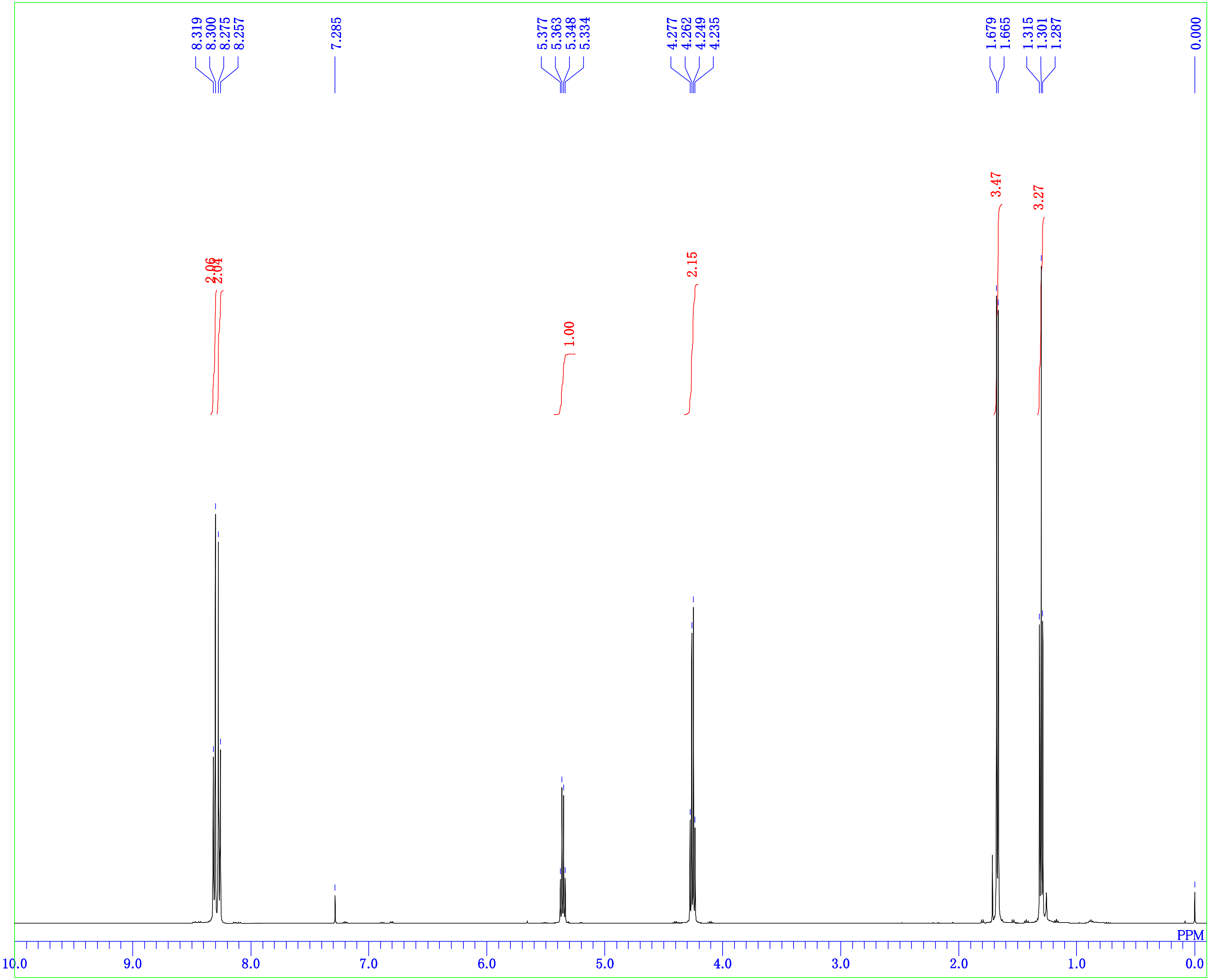

DFILE	d1453-gra-1h-1.als
COMNT	150701
DATIM	2015-07-01 20:53:07
OBNUC	1H
EXMOD	single_pulse.ex2
OBFRQ	500.16 MHz
OBSET	2.41 KHz
OBFIN	6.01 Hz
POINT	13107
FREQU	7507.39 Hz
SCANS	32
ACQTM	1.7459 sec
PD	2.0000 sec
PW1	5.80 usec
IRNUC	1H
CTEMP	20.5 c
SLVNT	CDCL3
EXREF	0.00 ppm
BF	0.12 Hz
RGAIN	32

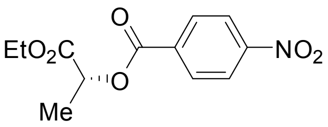

5

150701

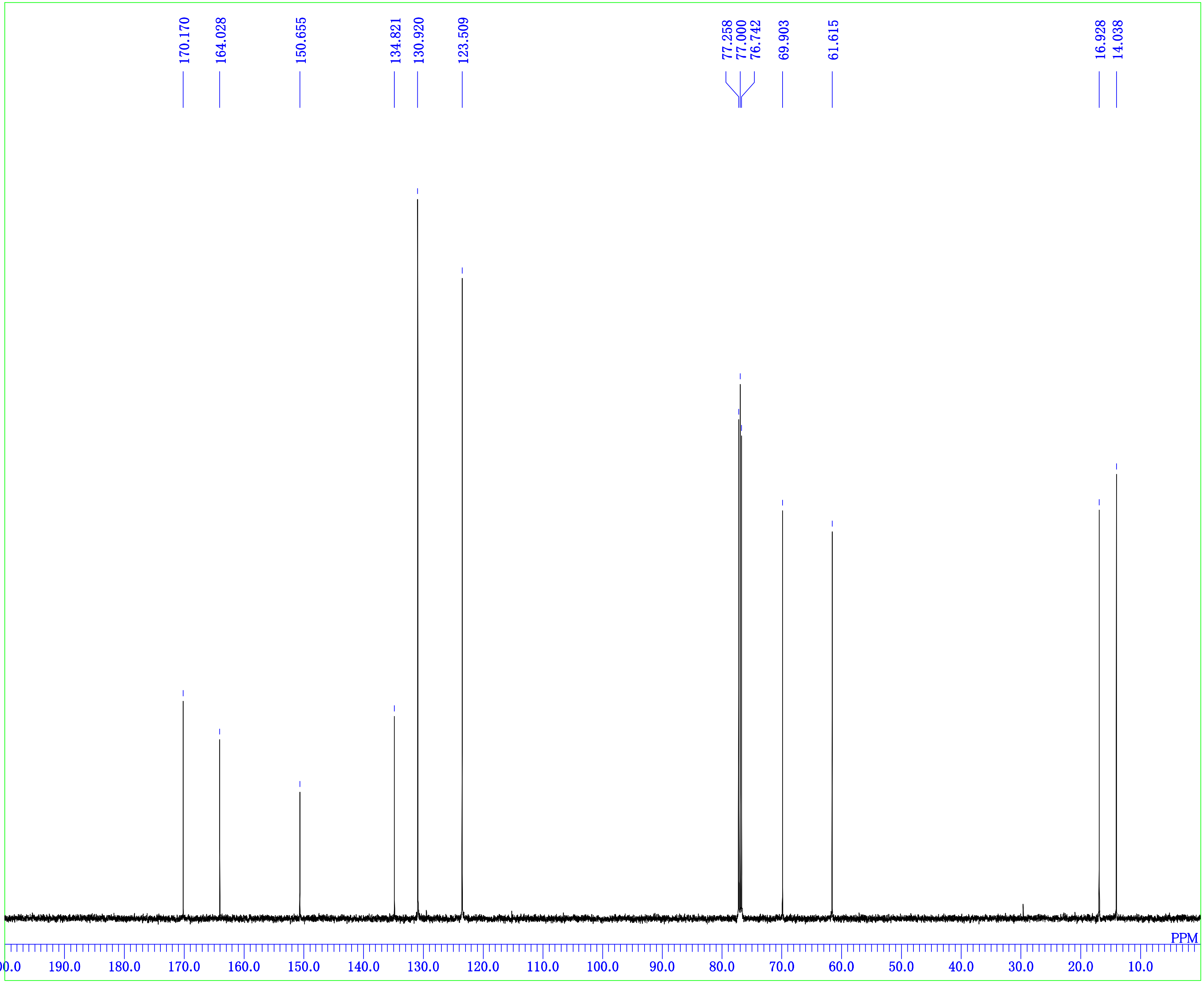

DFILE	d1453-gra-13c-1.als
COMNT	150701
DATIM	2015-07-01 21:05:54
OBNUC	13C
EXMOD	single_pulse_dec
OBFRQ	125.77 MHz
OBSET	7.87 KHz
OBFIN	4.21 Hz
POINT	26214
FREQU	31446.06 Hz
SCANS	256
ACQTM	0.8336 sec
PD	2.0000 sec
PW1	3.00 usec
IRNUC	1H
CTEMP	20.6 c
SLVNT	CDCL3
EXREF	77.00 ppm
BF	1.20 Hz
RGAIN	58

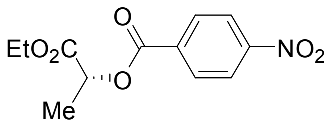

5

150701

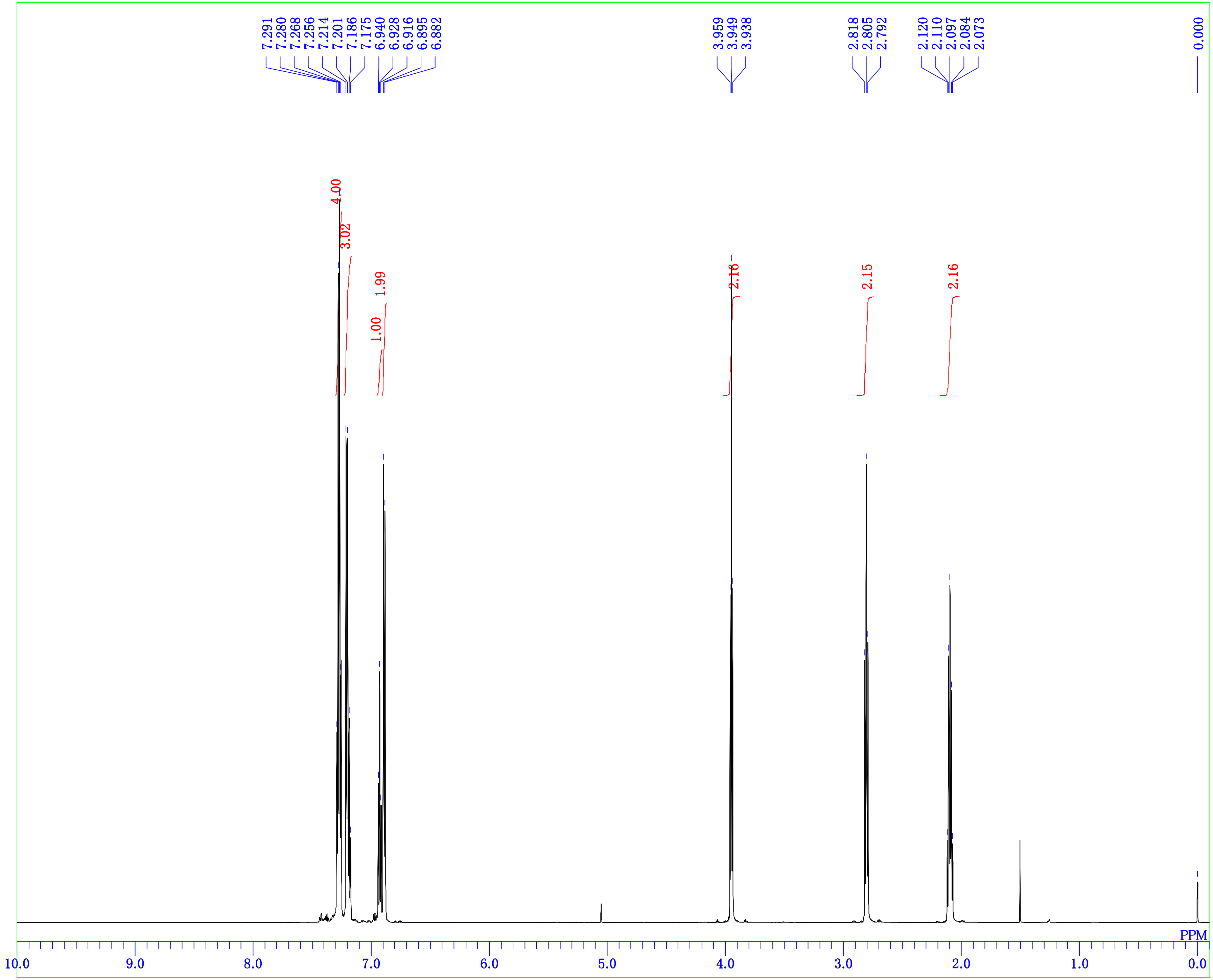

DFILE	d1506-gra-1h-1.als
COMNT	150701
DATIM	2015-03-06 16:41:45
OBNUC	1H
EXMOD	single_pulse.ex2
OBFRQ	600.17 MHz
OBSET	5.30 KHz
OBFIN	5.47 Hz
POINT	26214
FREQU	9008.87 Hz
SCANS	32
ACQTM	2.9098 sec
PD	2.0000 sec
PW1	5.85 usec
IRNUC	1H
CTEMP	20.6 c
SLVNT	CDCL3
EXREF	0.00 ppm
BF	0.12 Hz
RGAIN	30

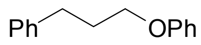

9

150701

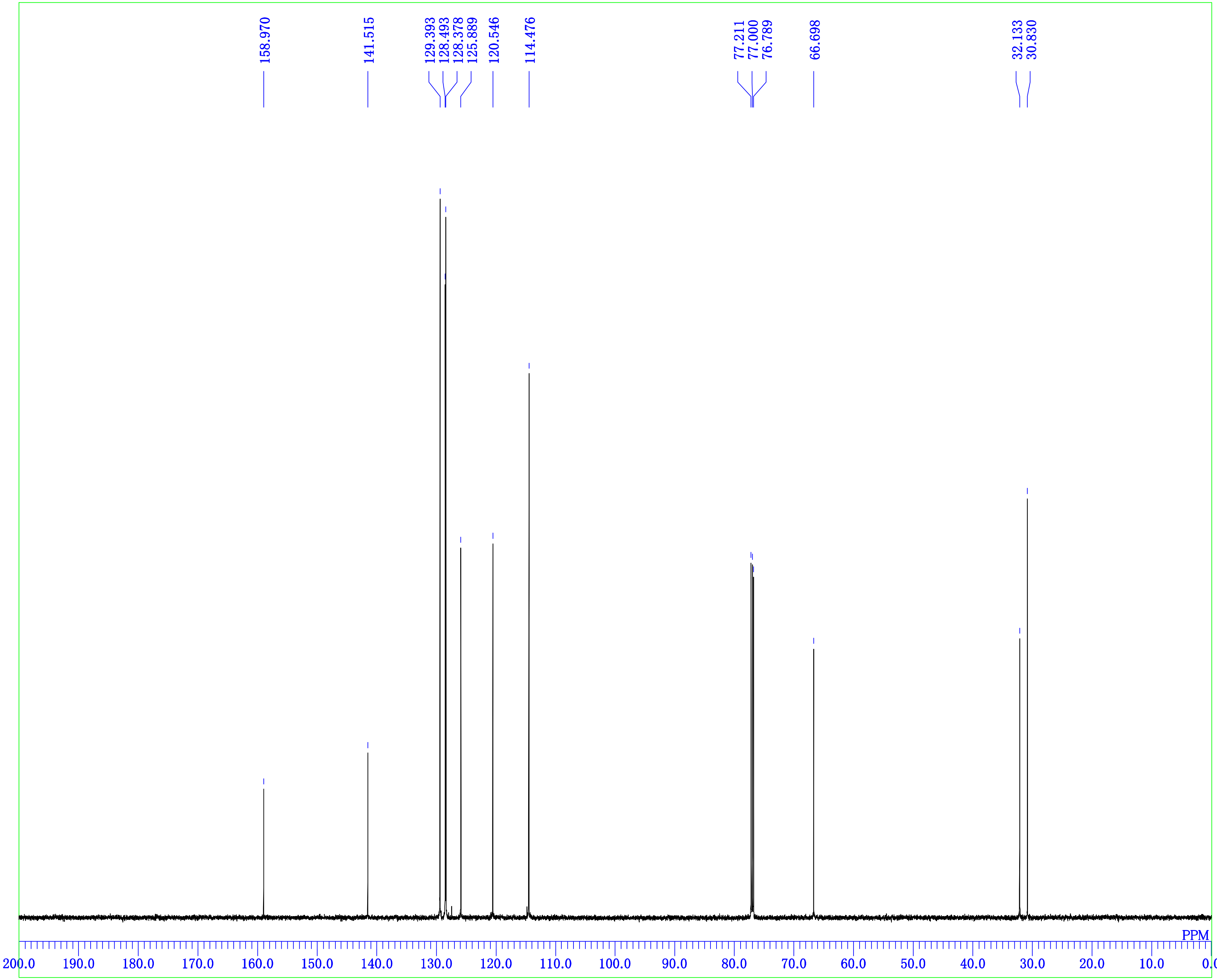

DFILE	d1506-gra-13c-1.als
COMNT	150701
DATIM	2015-03-06 16:50:20
OBNUC	13C
EXMOD	single_pulse_dec
OBFRQ	150.92 MHz
OBSET	8.52 KHz
OBFIN	1.74 Hz
POINT	26214
FREQU	37878.21 Hz
SCANS	256
ACQTM	0.6921 sec
PD	1.2000 sec
PW1	2.97 usec
IRNUC	1H
CTEMP	21.1 c
SLVNT	CDCL3
EXREF	77.00 ppm
BF	1.20 Hz
RGAIN	56

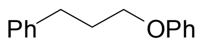

9

150704

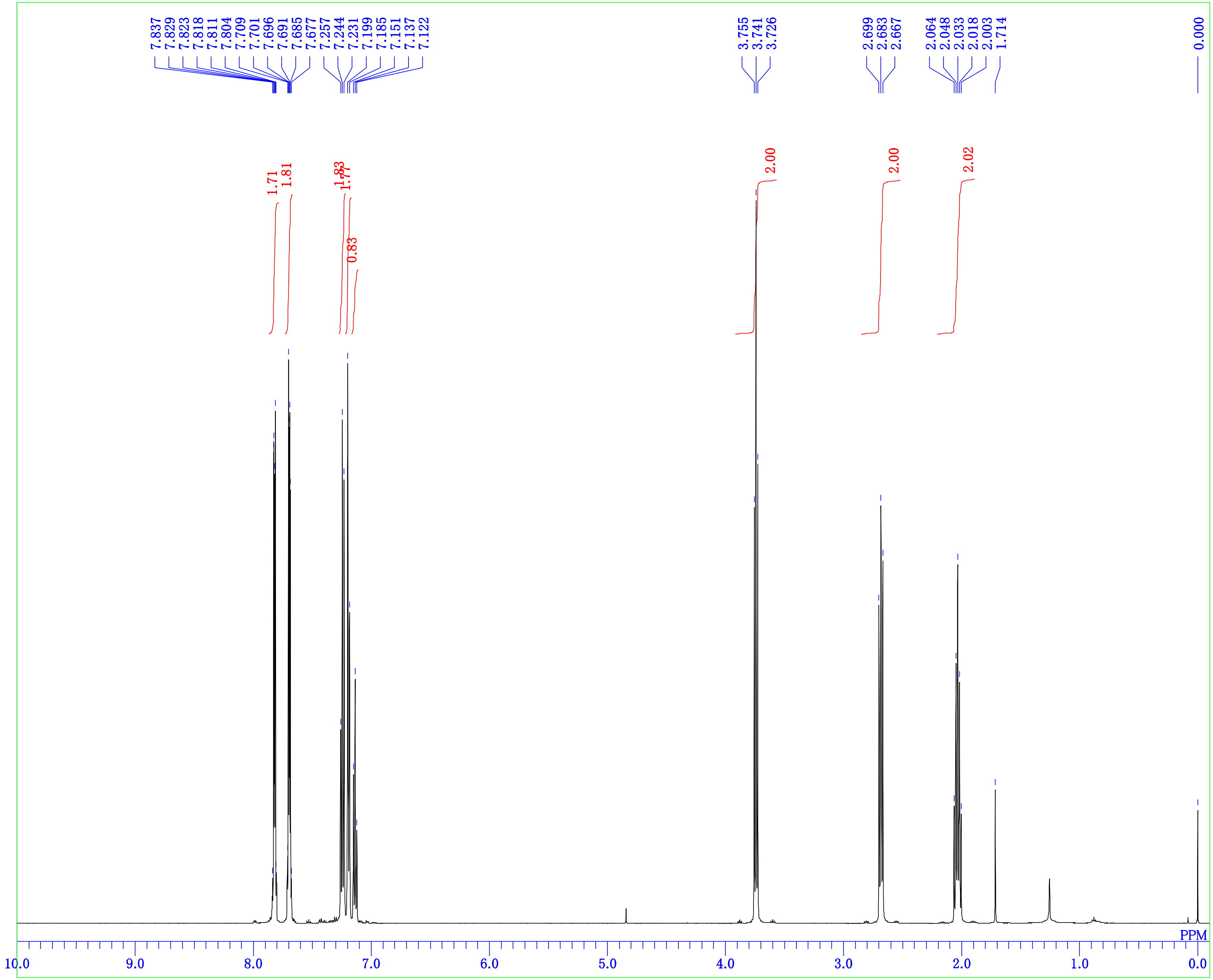

DFILE	d1500-gra-1h-1.als
COMNT	150704
DATIM	2015-07-04 17:11:18
OBNUC	1H
EXMOD	single_pulse.ex2
OBFRQ	500.16 MHz
OBSET	2.41 KHz
OBFIN	6.01 Hz
POINT	13107
FREQU	7507.39 Hz
SCANS	32
ACQTM	1.7459 sec
PD	2.0000 sec
PW1	5.80 usec
IRNUC	1H
CTEMP	20.0 c
SLVNT	CDCL3
EXREF	0.00 ppm
BF	0.12 Hz
RGAIN	34

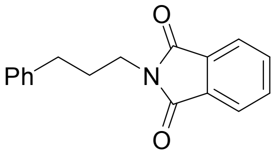

10

150704

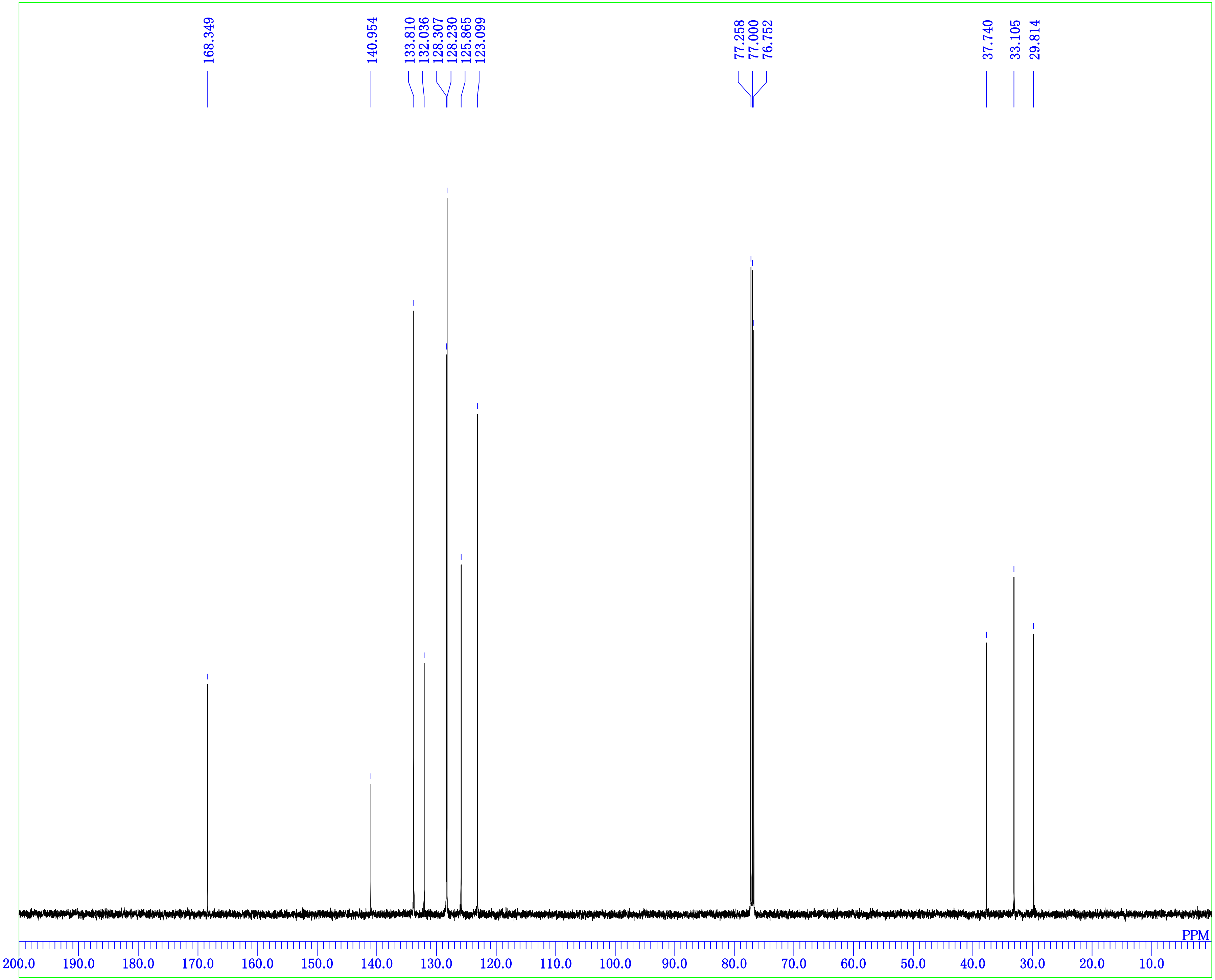

DFILE d1500-gra-13c-1.als
COMNT 150704
DATIM 2015-07-04 17:24:07
OBNUC 13C
EXMOD single_pulse_dec
OBFRQ 125.77 MHz
OBSET 7.87 KHz
OBFIN 4.21 Hz
POINT 26214
FREQU 31446.06 Hz
SCANS 256
ACQTM 0.8336 sec
PD 2.0000 sec
PW1 3.00 usec
IRNUC 1H
CTEMP 20.5 c
SLVNT CDCL3
EXREF 77.00 ppm
BF 1.20 Hz
RGAIN 60

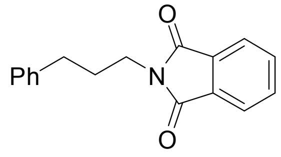

10

150701

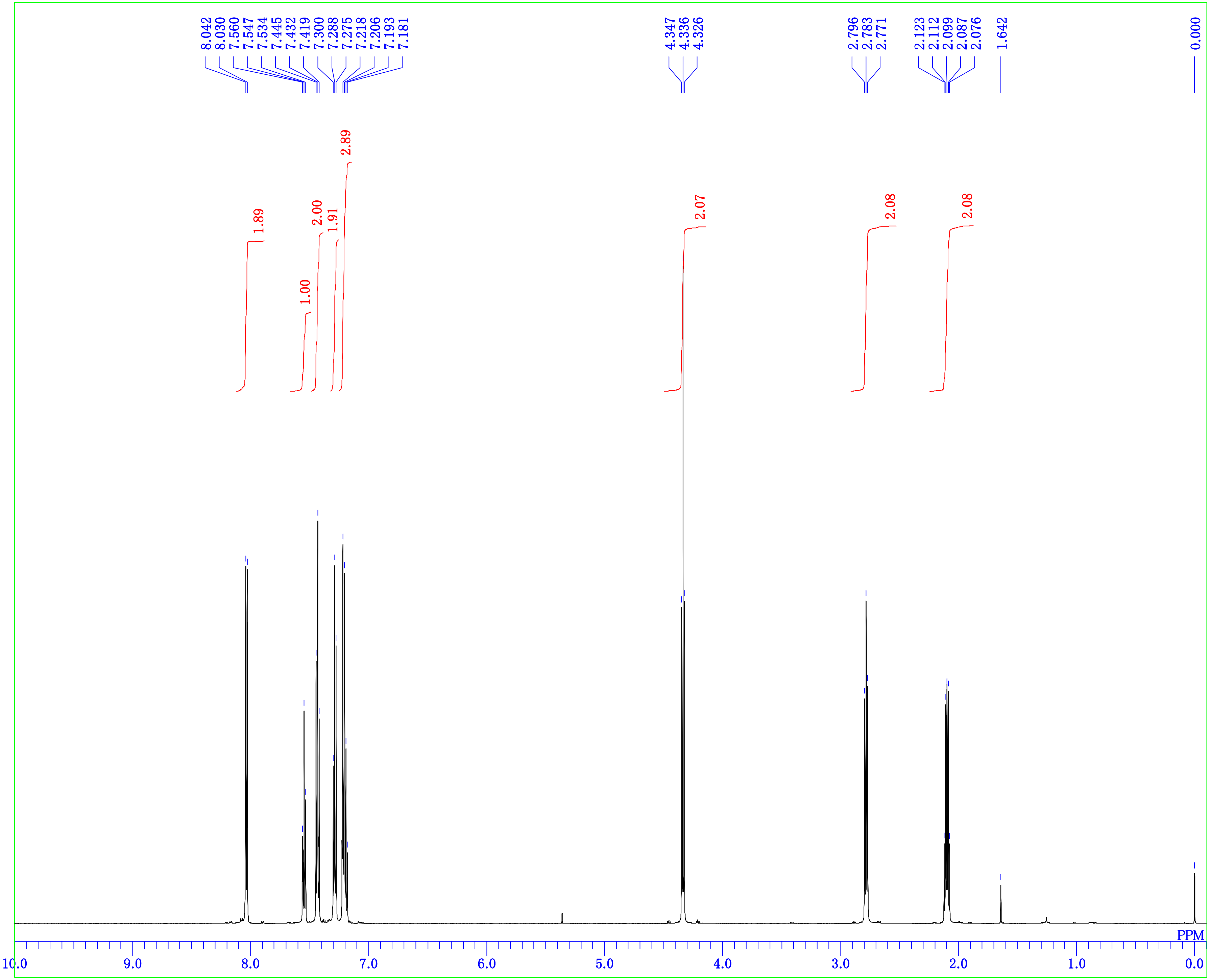

DFILE	d1505-gra-1h-1.als
COMNT	150701
DATIM	2015-03-06 16:23:16
OBNUC	1H
EXMOD	single_pulse.ex2
OBFRQ	600.17 MHz
OBSET	5.30 KHz
OBFIN	5.47 Hz
POINT	26214
FREQU	9008.87 Hz
SCANS	32
ACQTM	2.9098 sec
PD	2.0000 sec
PW1	5.85 usec
IRNUC	1H
CTEMP	20.4 c
SLVNT	CDCL3
EXREF	0.00 ppm
BF	0.12 Hz
RGAIN	30

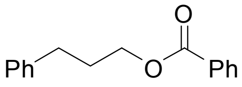

11

150701

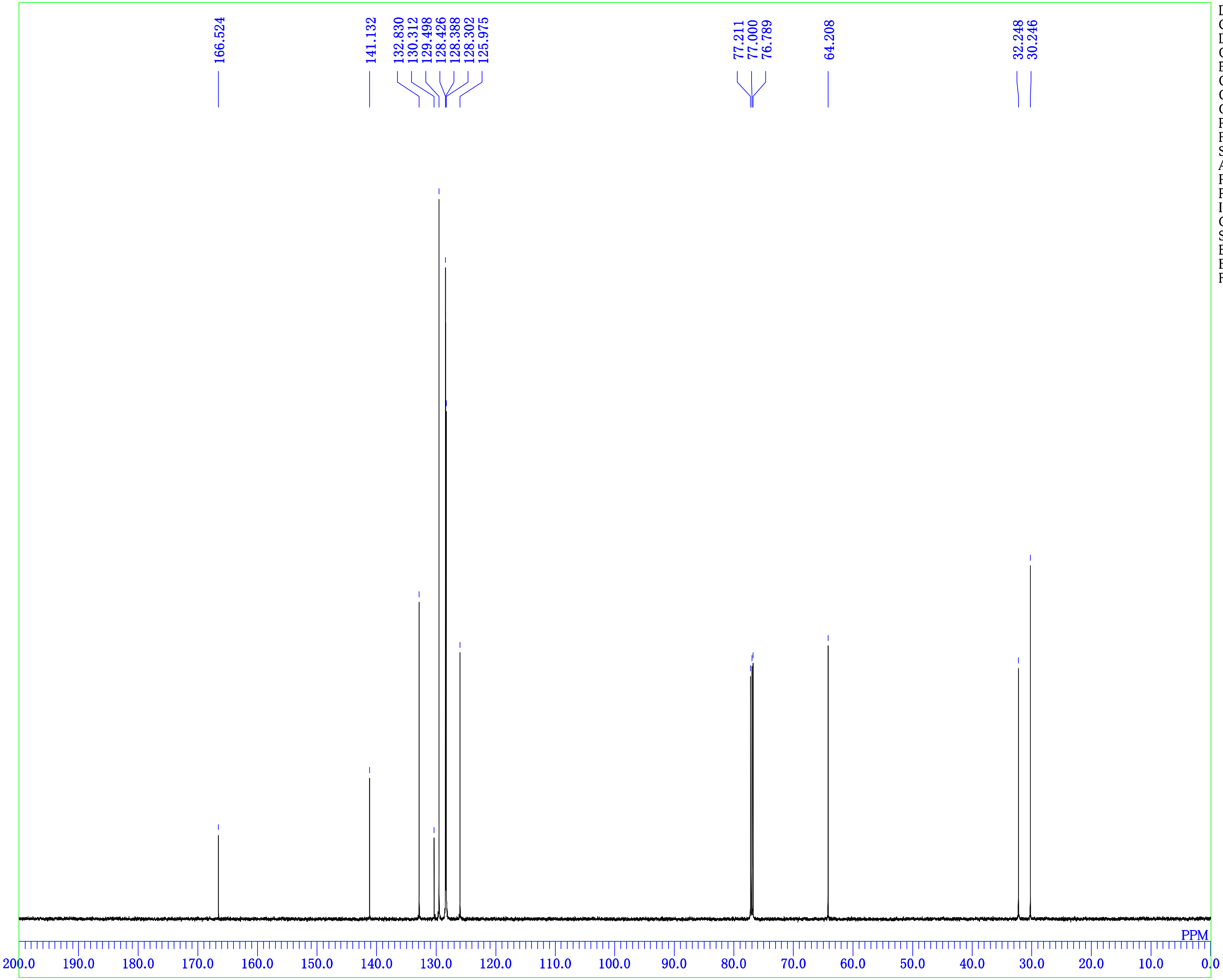

DFILE	d1505-gra-13c-1.als
COMNT	150701
DATIM	2015-03-06 16:32:54
OBNUC	13C
EXMOD	single_pulse_dec
OBFRQ	150.92 MHz
OBSET	8.52 KHz
OBFIN	1.74 Hz
POINT	26214
FREQU	37878.21 Hz
SCANS	256
ACQTM	0.6921 sec
PD	1.2000 sec
PW1	2.97 usec
IRNUC	1H
CTEMP	21.0 c
SLVNT	CDCL3
EXREF	77.00 ppm
BF	1.20 Hz
RGAIN	56

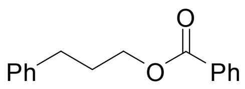

11

150701

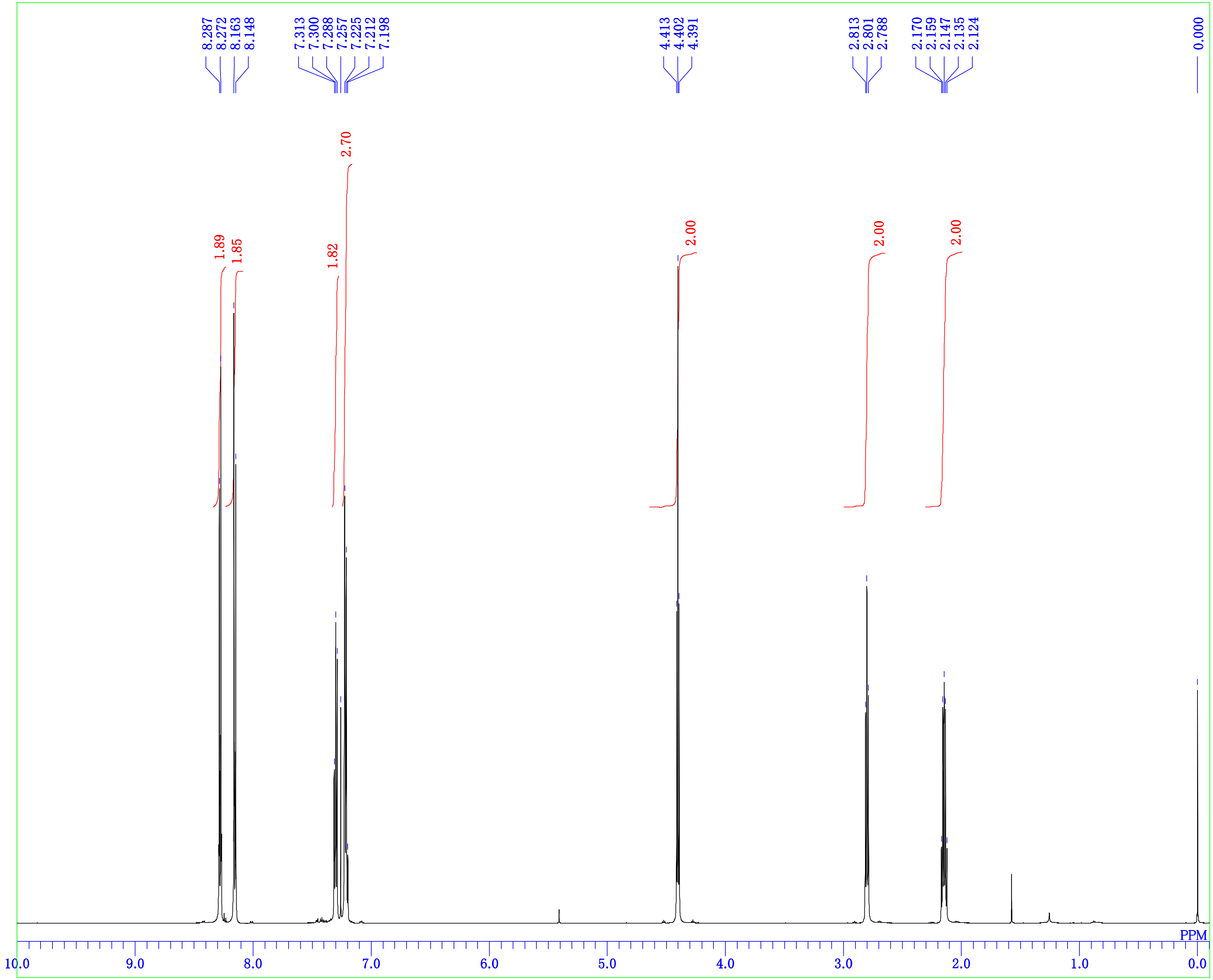

DFILE	d1523-gra-1h-1.als
COMNT	150701
DATIM	2015-03-24 21:10:26
OBNUC	1H
EXMOD	single_pulse.ex2
OBFRQ	600.17 MHz
OBSET	5.30 KHz
OBFIN	5.47 Hz
POINT	26214
FREQU	9008.87 Hz
SCANS	32
ACQTM	2.9098 sec
PD	2.0000 sec
PW1	7.30 usec
IRNUC	1H
CTEMP	20.2 c
SLVNT	CDCL3
EXREF	0.00 ppm
BF	0.12 Hz
RGAIN	40

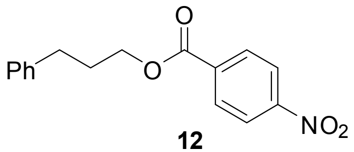

12

150701

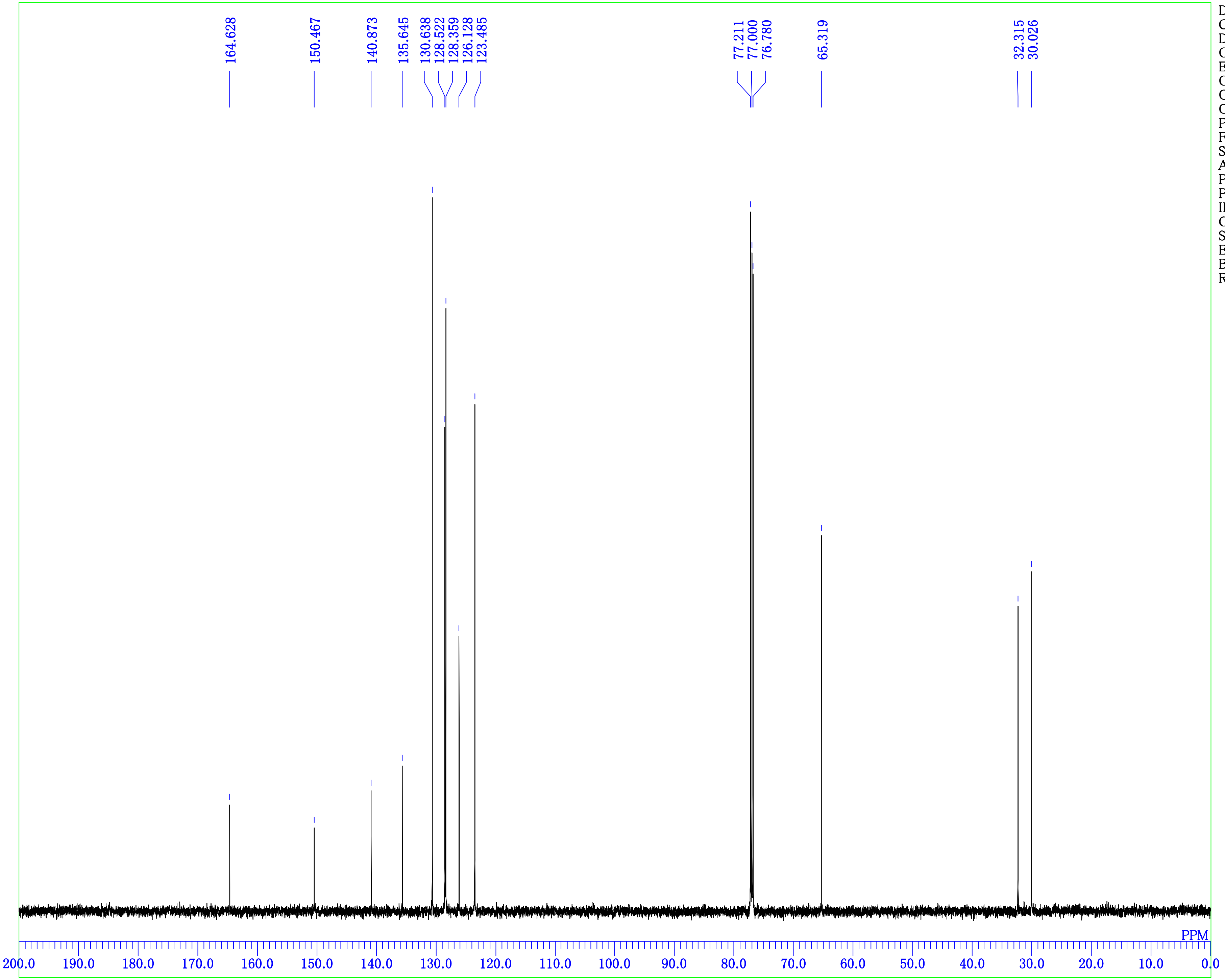

DFILE	d1523-gra-13c-1.als
COMNT	150701
DATIM	2015-03-24 21:19:07
OBNUC	13C
EXMOD	single_pulse_dec
OBFRQ	150.92 MHz
OBSET	8.52 KHz
OBFIN	1.74 Hz
POINT	26214
FREQU	37878.21 Hz
SCANS	256
ACQTM	0.6921 sec
PD	1.2000 sec
PW1	3.13 usec
IRNUC	1H
CTEMP	20.6 c
SLVNT	CDCL3
EXREF	77.00 ppm
BF	1.20 Hz
RGAIN	56

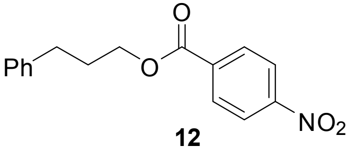

12

150501

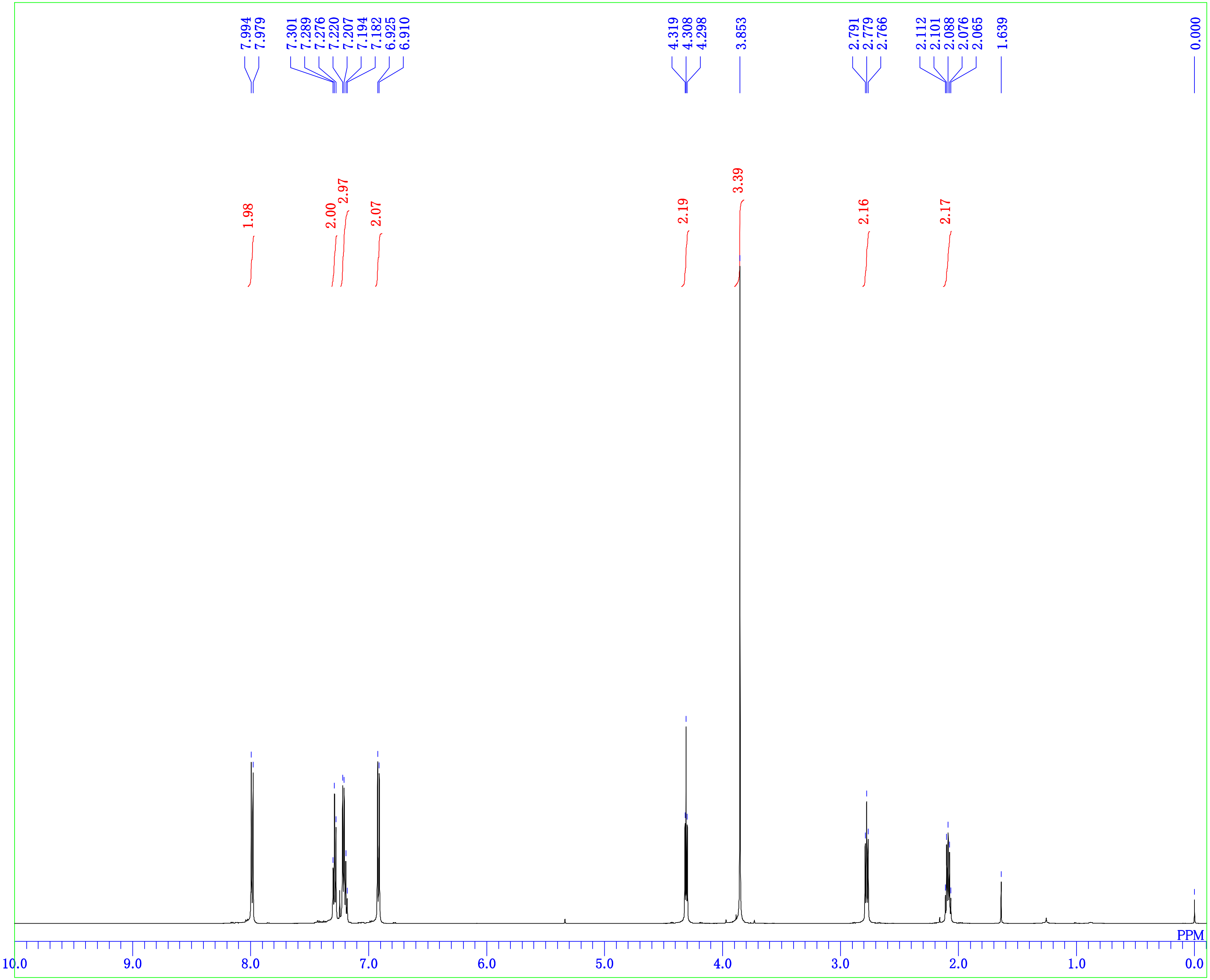

DFILE	d1568-gra-1h-1.als
COMNT	150501
DATIM	2015-05-01 09:47:35
OBNUC	1H
EXMOD	single_pulse.ex2
OBFRQ	600.17 MHz
OBSET	5.30 KHz
OBFIN	5.47 Hz
POINT	26214
FREQU	9008.87 Hz
SCANS	32
ACQTM	2.9098 sec
PD	2.0000 sec
PW1	7.30 usec
IRNUC	1H
CTEMP	21.8 c
SLVNT	CDCL3
EXREF	0.00 ppm
BF	0.12 Hz
RGAIN	32

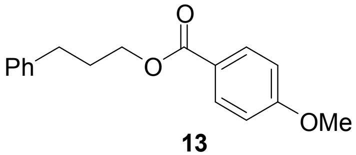

13

150501

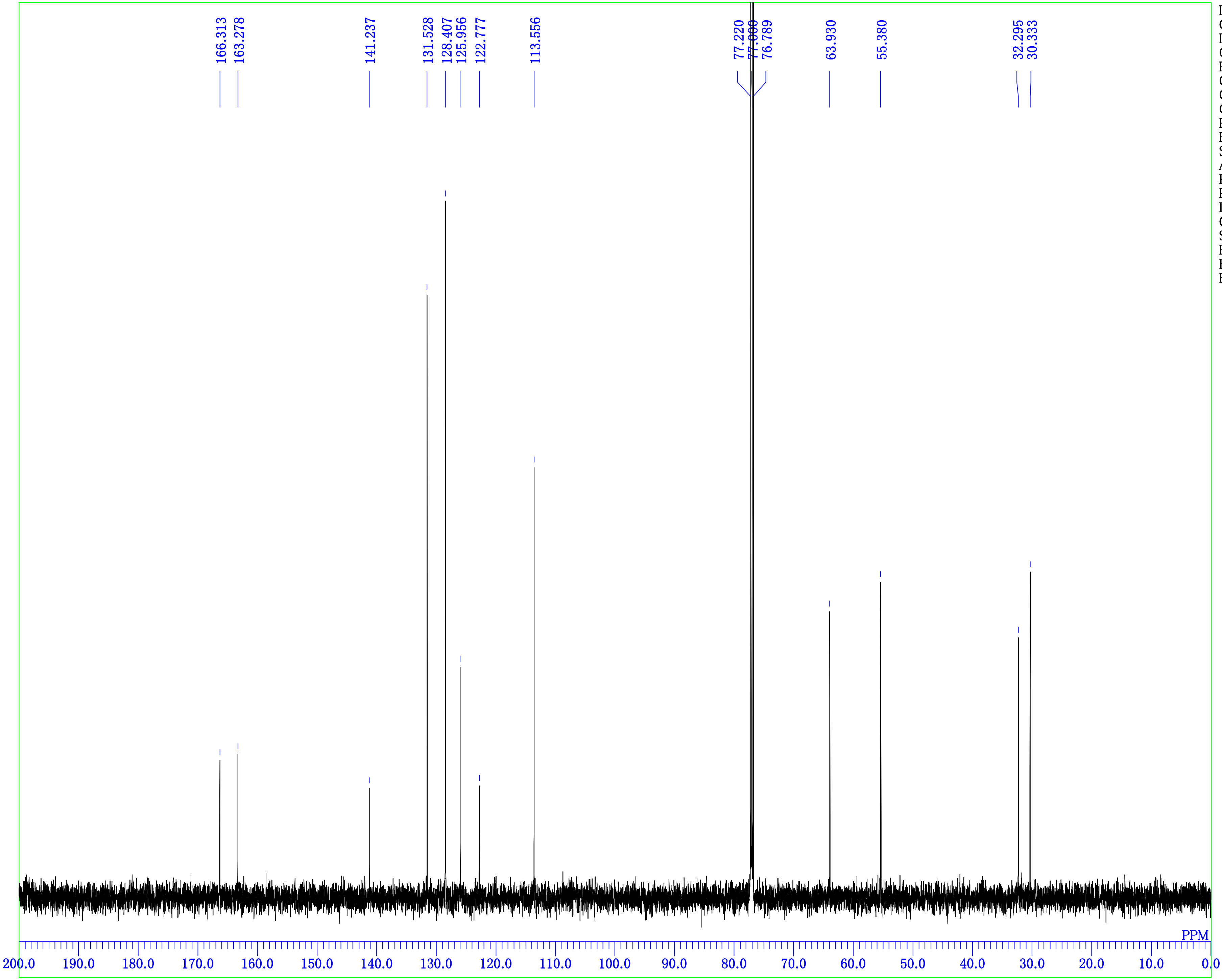

DFILE	d1568-gra-13c-1.als
COMNT	150501
DATIM	2015-05-01 09:56:06
OBNUC	13C
EXMOD	single_pulse_dec
OBFRQ	150.92 MHz
OBSET	8.52 KHz
OBFIN	1.74 Hz
POINT	26214
FREQU	37878.21 Hz
SCANS	256
ACQTM	0.6921 sec
PD	1.2000 sec
PW1	3.13 usec
IRNUC	1H
CTEMP	22.4 c
SLVNT	CDCL3
EXREF	77.00 ppm
BF	1.20 Hz
RGAIN	60

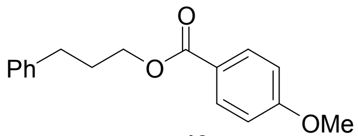

13

150701

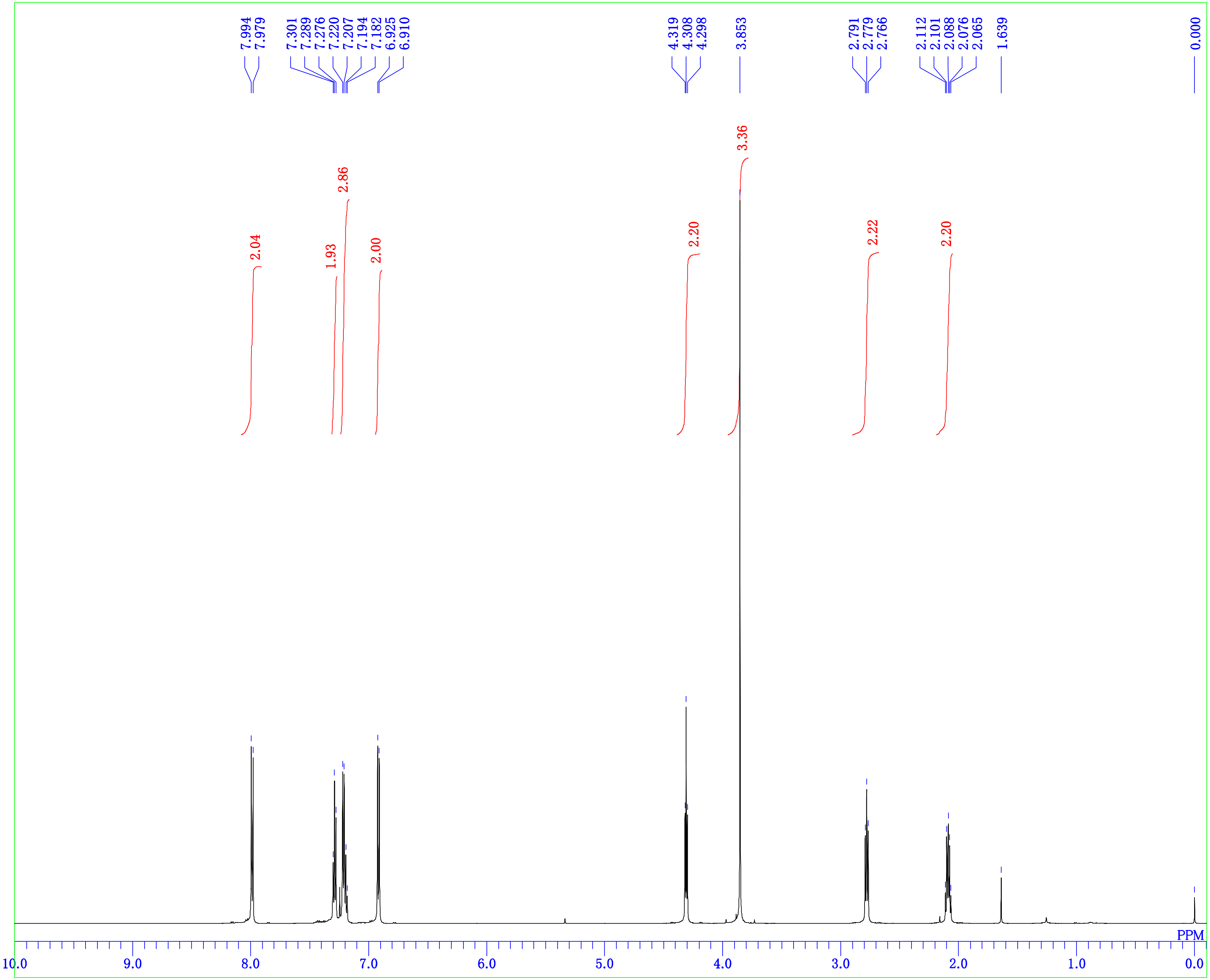

DFILE	d1568-gra-1h-1.als
COMNT	150701
DATIM	2015-05-01 09:47:35
OBNUC	1H
EXMOD	single_pulse.ex2
OBFRQ	600.17 MHz
OBSET	5.30 KHz
OBFIN	5.47 Hz
POINT	26214
FREQU	9008.87 Hz
SCANS	32
ACQTM	2.9098 sec
PD	2.0000 sec
PW1	7.30 usec
IRNUC	1H
CTEMP	21.8 c
SLVNT	CDCL3
EXREF	0.00 ppm
BF	0.12 Hz
RGAIN	32

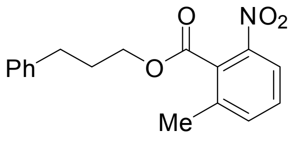

14

150701

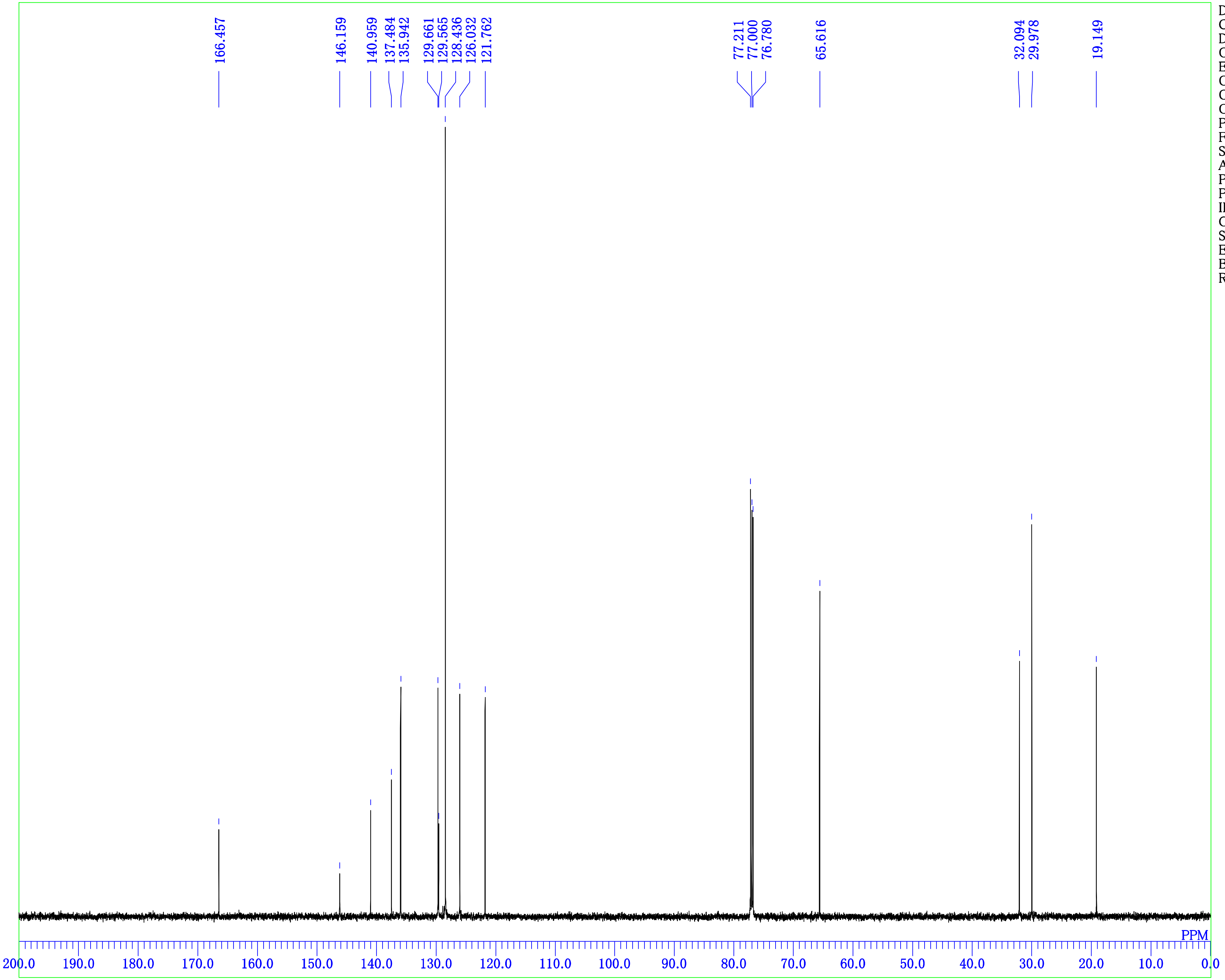

DFILE	d1575-gra-13c-1.als
COMNT	150701
DATIM	2015-05-19 18:19:49
OBNUC	13C
EXMOD	single_pulse_dec
OBFRQ	150.92 MHz
OBSET	8.52 KHz
OBFIN	1.74 Hz
POINT	26214
FREQU	37878.21 Hz
SCANS	256
ACQTM	0.6921 sec
PD	1.2000 sec
PW1	3.13 usec
IRNUC	1H
CTEMP	21.9 c
SLVNT	CDCL3
EXREF	77.00 ppm
BF	1.20 Hz
RGAIN	56

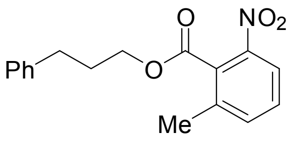

14

150701

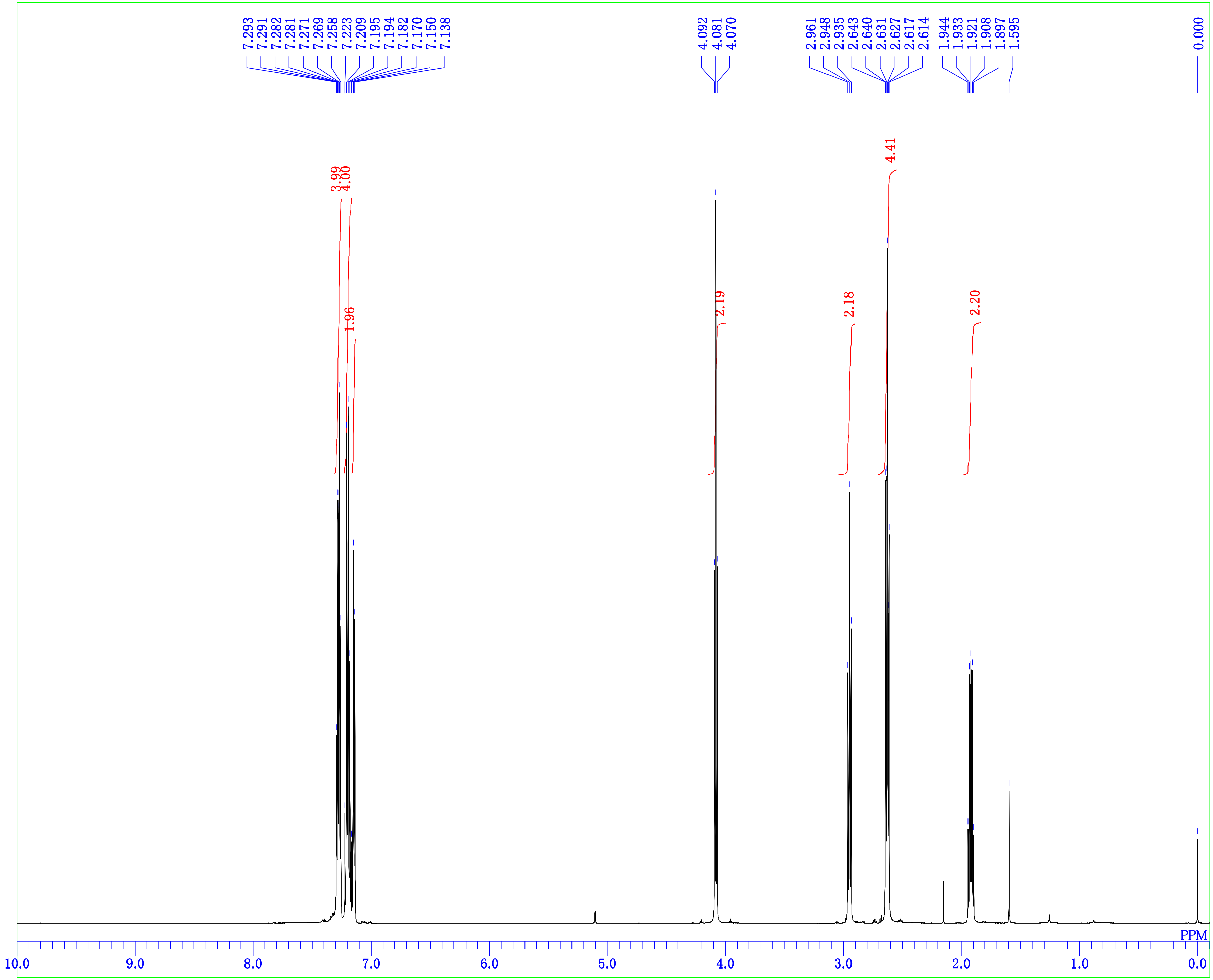

DFILE d1569-gra-1h-1.als
COMNT 150701
DATIM 2015-05-01 10:03:52
OBNUC 1H
EXMOD single_pulse.ex2
OBFRQ 600.17 MHz
OBSET 5.30 KHz
OBFIN 5.47 Hz
POINT 26214
FREQU 9008.87 Hz
SCANS 32
ACQTM 2.9098 sec
PD 2.0000 sec
PW1 7.30 usec
IRNUC 1H
CTEMP 21.6 c
SLVNT CDCL3
EXREF 0.00 ppm
BF 0.12 Hz
RGAIN 30

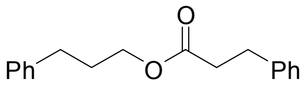

15

150701

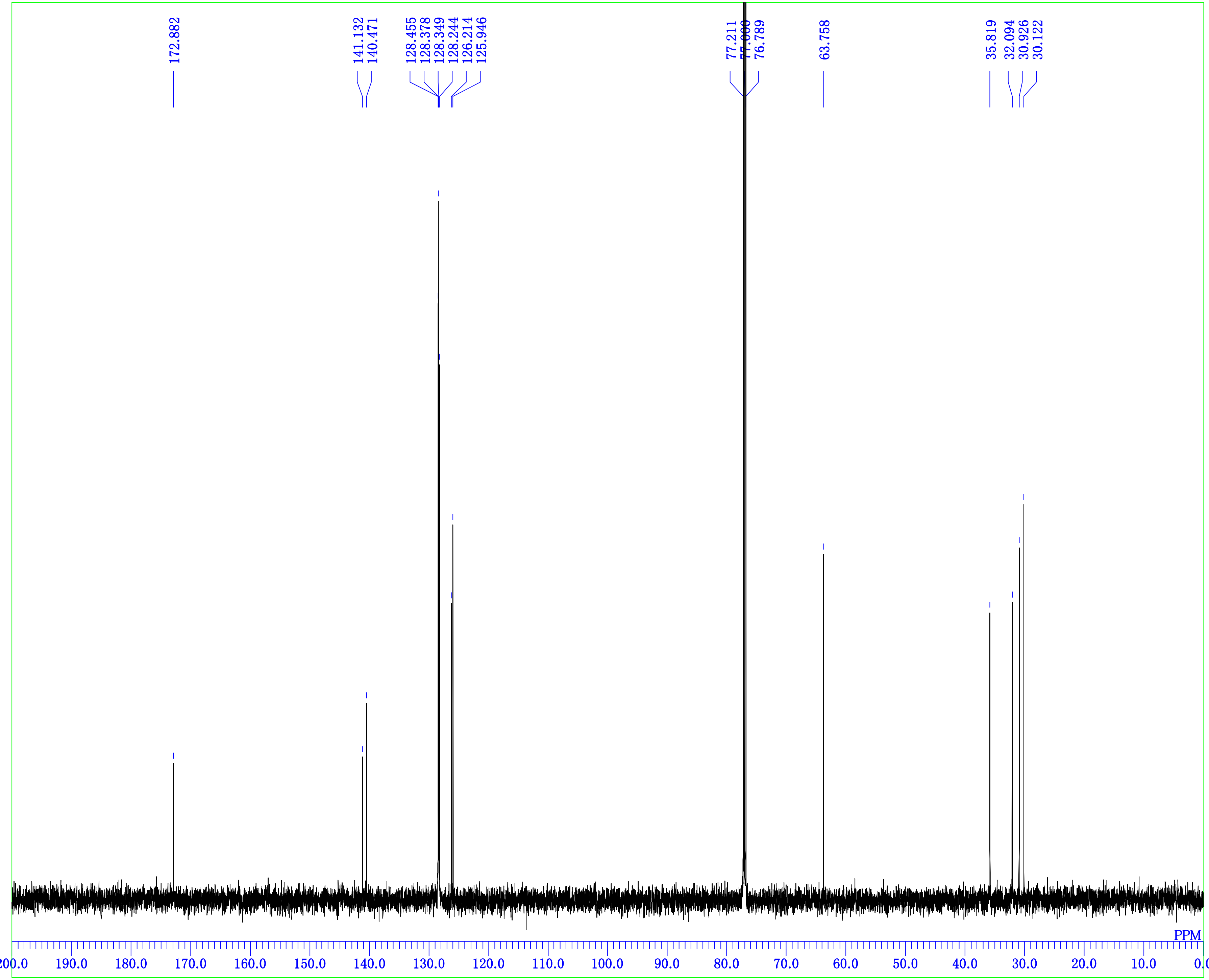

DFILE	d1569-gra-13c-1.als
COMNT	150701
DATIM	2015-05-01 10:12:25
OBNUC	13C
EXMOD	single_pulse_dec
OBFRQ	150.92 MHz
OBSET	8.52 KHz
OBFIN	1.74 Hz
POINT	26214
FREQU	37878.21 Hz
SCANS	256
ACQTM	0.6921 sec
PD	1.2000 sec
PW1	3.13 usec
IRNUC	1H
CTEMP	22.6 c
SLVNT	CDCL3
EXREF	77.00 ppm
BF	1.20 Hz
RGAIN	60

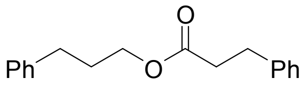

15

150701

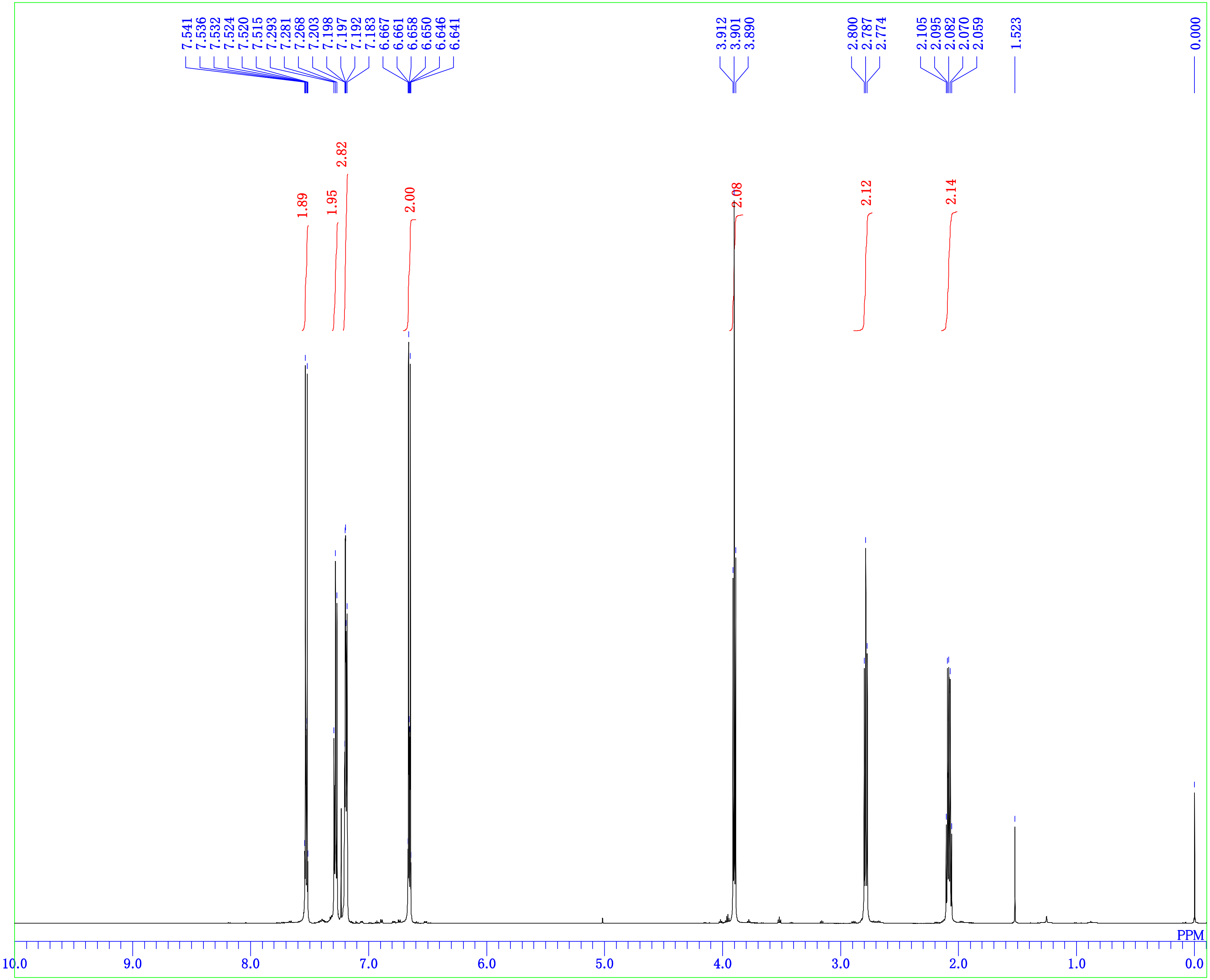

DFILE d1529-gra-1h-1.als
COMNT 150701
DATIM 2015-04-21 16:22:13
OBNUC 1H
EXMOD single_pulse.ex2
OBFRQ 600.17 MHz
OBSET 5.30 KHz
OBFIN 5.47 Hz
POINT 26214
FREQU 9008.87 Hz
SCANS 32
ACQTM 2.9098 sec
PD 2.0000 sec
PW1 7.30 usec
IRNUC 1H
CTEMP 21.1 c
SLVNT CDCL3
EXREF 0.00 ppm
BF 0.12 Hz
RGAIN 34

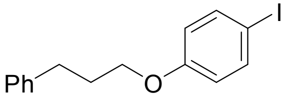

16

150701

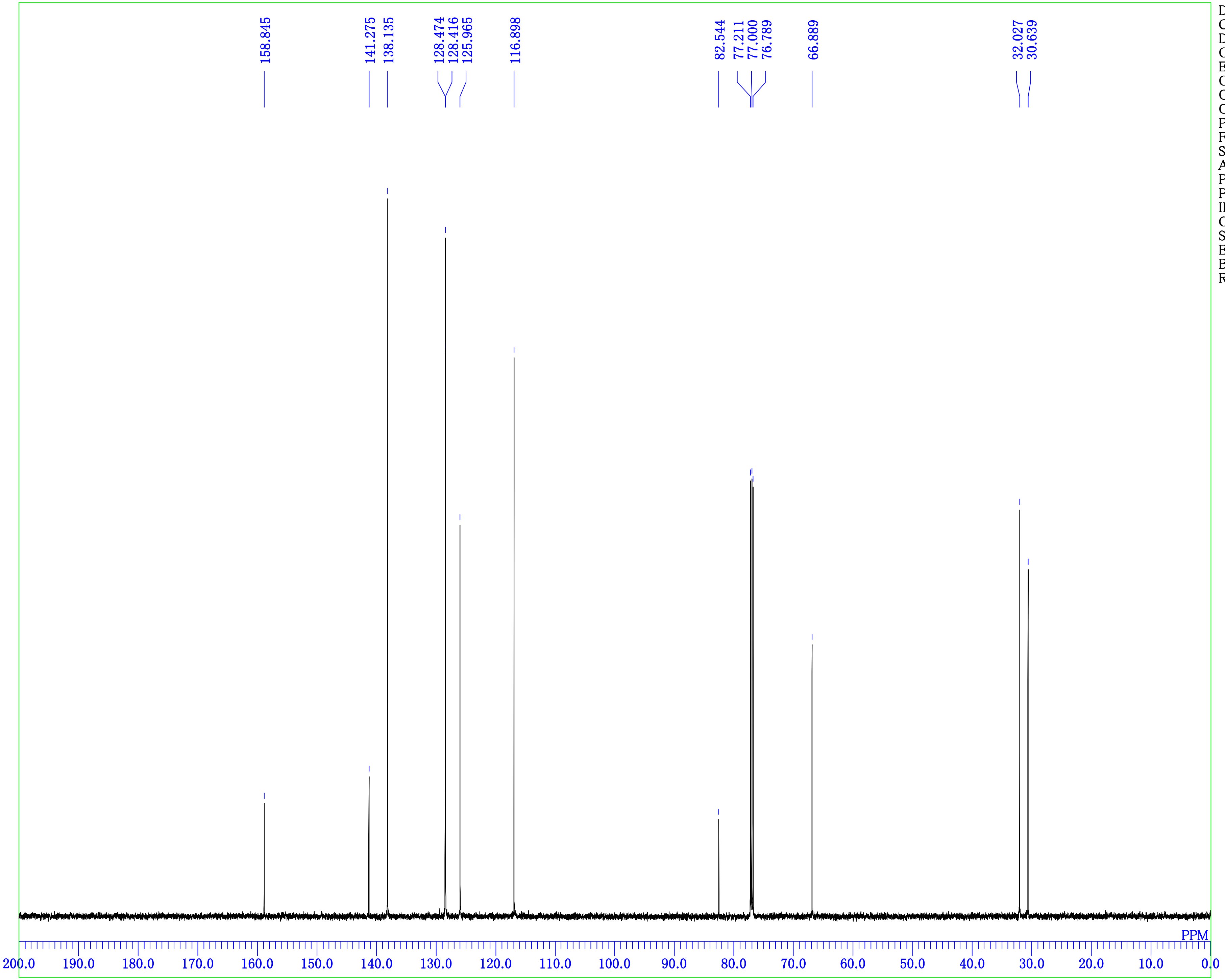

DFILE	d1529-gra-13c-1.als
COMNT	150701
DATIM	2015-04-21 16:33:50
OBNUC	13C
EXMOD	single_pulse_dec
OBFRQ	150.92 MHz
OBSET	8.52 KHz
OBFIN	1.74 Hz
POINT	26214
FREQU	37878.21 Hz
SCANS	256
ACQTM	0.6921 sec
PD	1.2000 sec
PW1	3.13 usec
IRNUC	1H
CTEMP	21.7 c
SLVNT	CDCL3
EXREF	77.00 ppm
BF	1.20 Hz
RGAIN	54

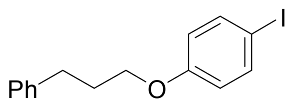

16

151113

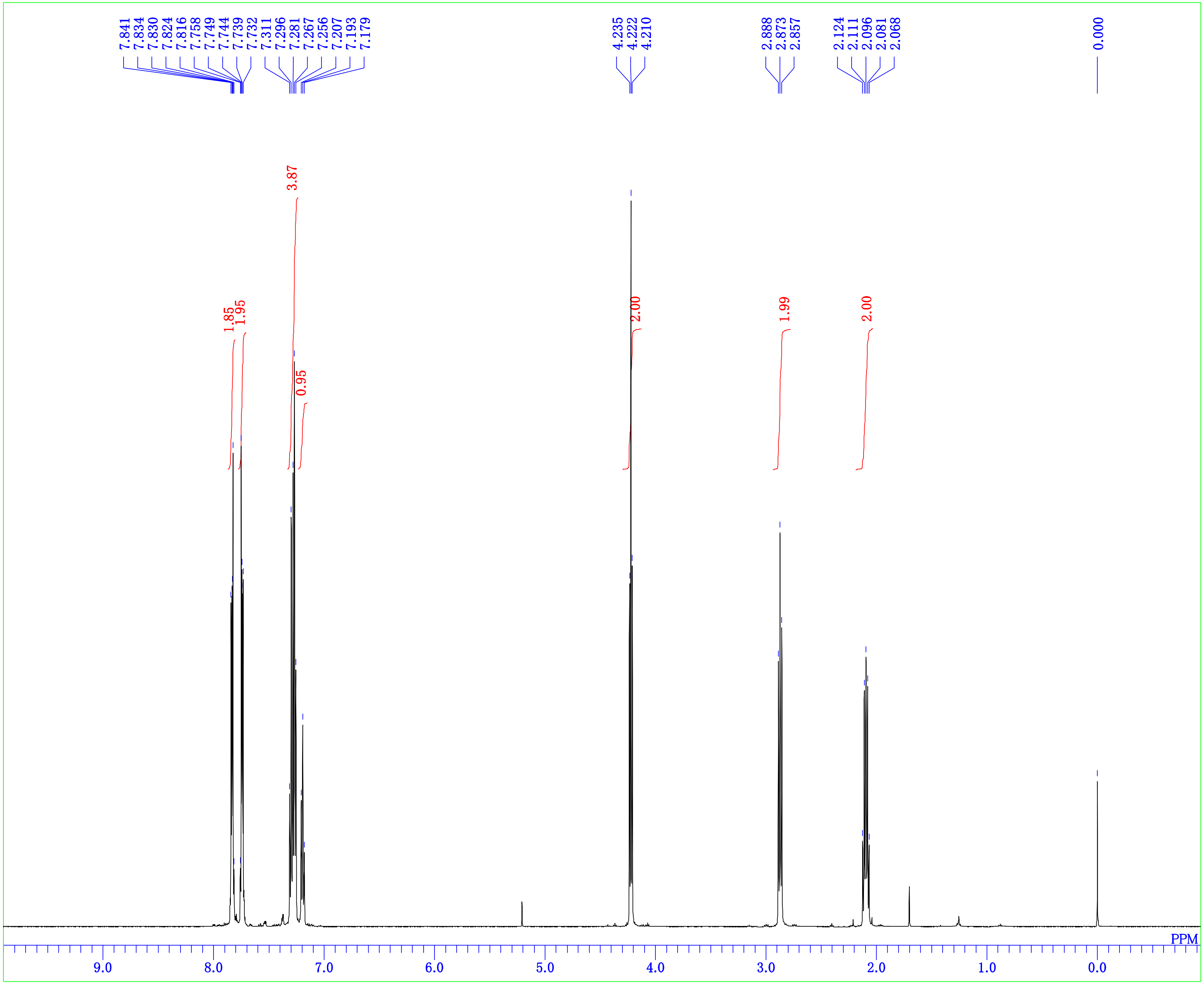

DFILE	d1769-gra-1h-1.jdf
COMNT	151113
DATIM	2015-11-13 13:00:17
OBNUC	1H
EXMOD	single_pulse.ex2
OBFRQ	500.16 MHz
OBSET	2.41 KHz
OBFIN	6.01 Hz
POINT	16384
FREQU	9384.38 Hz
SCANS	32
ACQTM	1.7459 sec
PD	2.0000 sec
PW1	5.80 usec
IRNUC	1H
CTEMP	18.6 c
SLVNT	CDCL3
EXREF	0.00 ppm
BF	0.12 Hz
RGAIN	40

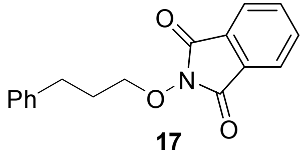

17

151113

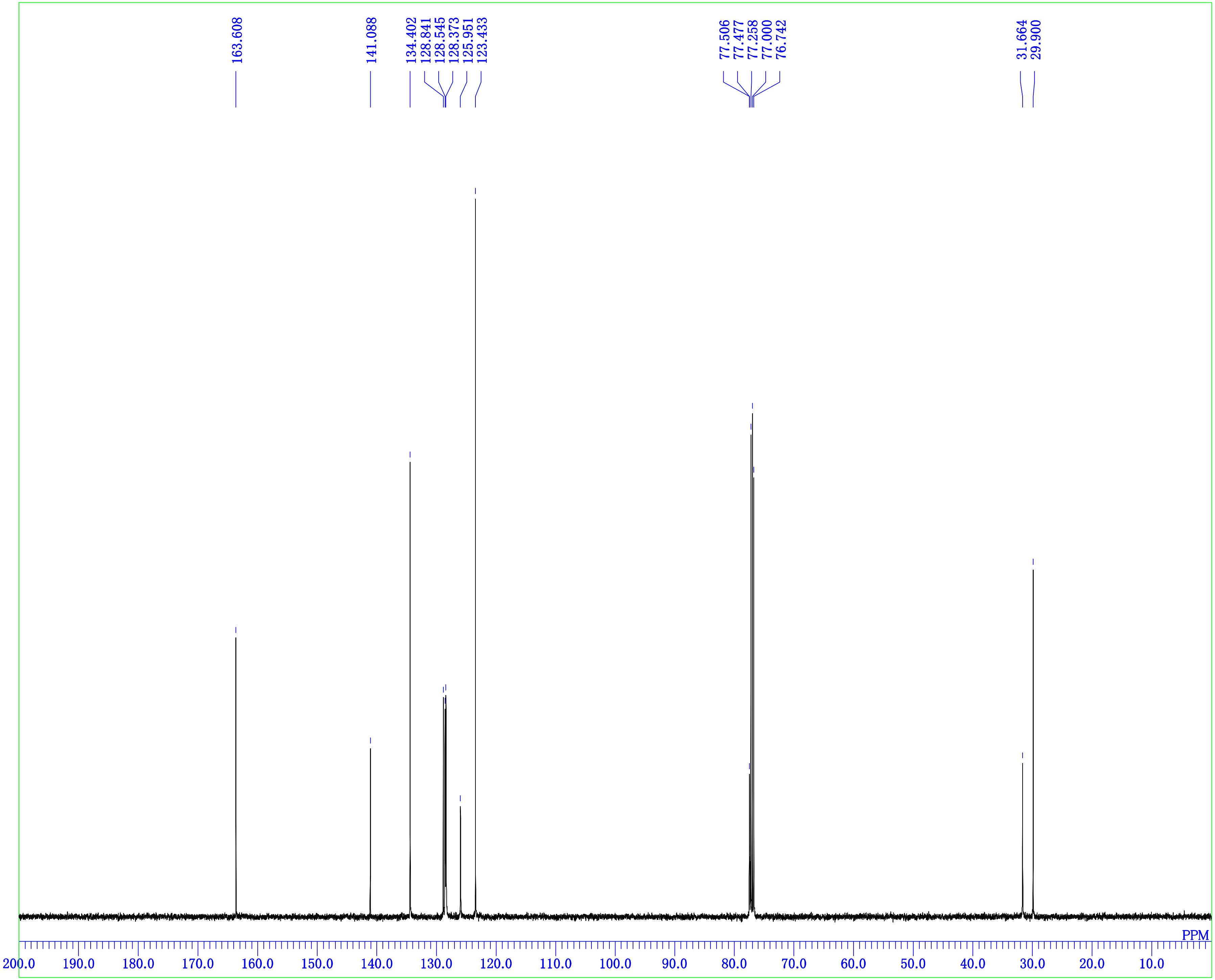

DFILE	d1769-gra-13c-1.als
COMNT	151113
DATIM	2015-11-13 13:13:10
OBNUC	13C
EXMOD	single_pulse_dec
OBFRQ	125.77 MHz
OBSET	7.87 KHz
OBFIN	4.21 Hz
POINT	26214
FREQU	31446.06 Hz
SCANS	256
ACQTM	0.8336 sec
PD	2.0000 sec
PW1	3.00 usec
IRNUC	1H
CTEMP	18.8 c
SLVNT	CDCL3
EXREF	77.00 ppm
BF	1.20 Hz
RGAIN	54

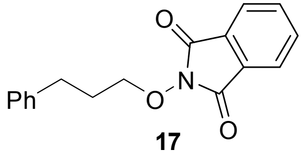

17

150406

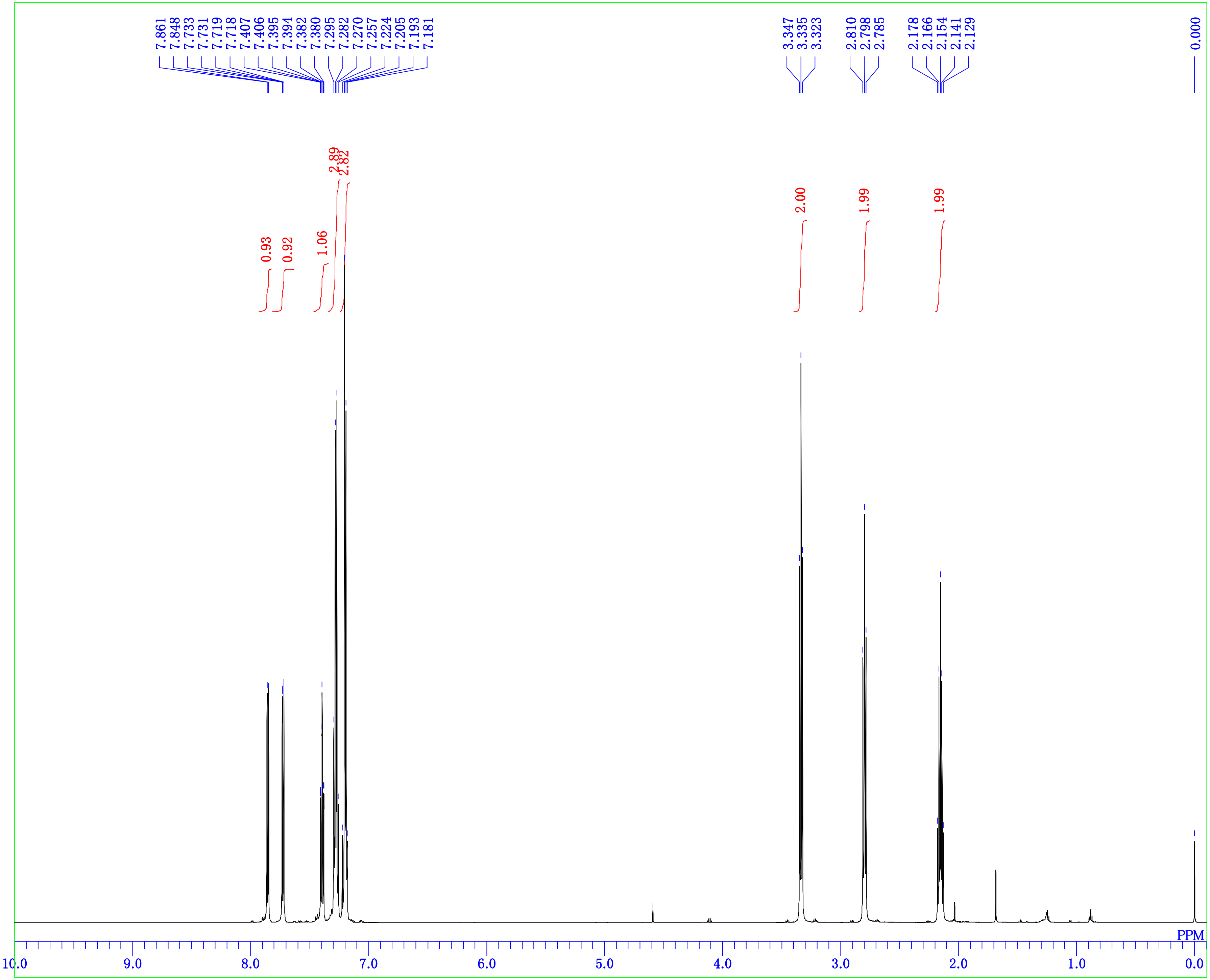

DFILE	d1547-gra-1h-1.als
COMNT	150406
DATIM	2015-04-06 15:29:56
OBNUC	1H
EXMOD	single_pulse.ex2
OBFRQ	600.17 MHz
OBSET	5.30 KHz
OBFIN	5.47 Hz
POINT	26214
FREQU	9008.87 Hz
SCANS	32
ACQTM	2.9098 sec
PD	2.0000 sec
PW1	7.30 usec
IRNUC	1H
CTEMP	20.9 c
SLVNT	CDCL3
EXREF	0.00 ppm
BF	0.12 Hz
RGAIN	30

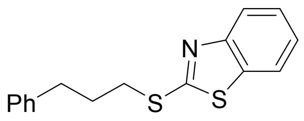

18

150406

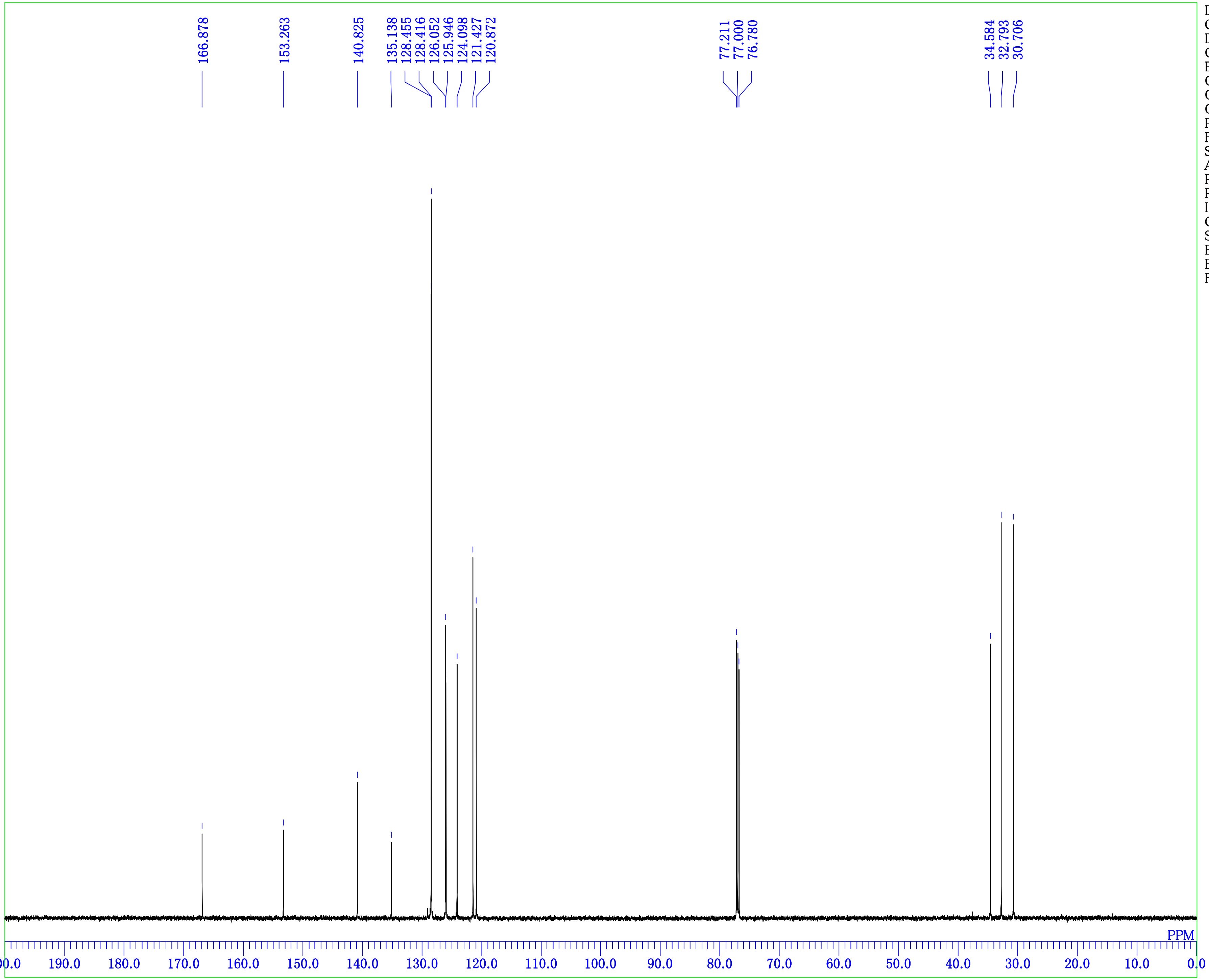

DFILE	d1547-gra-13c-1.als
COMNT	150406
DATIM	2015-04-06 15:39:21
OBNUC	13C
EXMOD	single_pulse_dec
OBFRQ	150.92 MHz
OBSET	8.52 KHz
OBFIN	1.74 Hz
POINT	26214
FREQU	37878.21 Hz
SCANS	256
ACQTM	0.6921 sec
PD	1.2000 sec
PW1	3.13 usec
IRNUC	1H
CTEMP	21.7 c
SLVNT	CDCL3
EXREF	77.00 ppm
BF	1.20 Hz
RGAIN	56

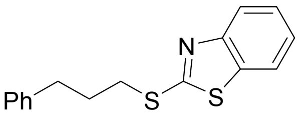

18

150704

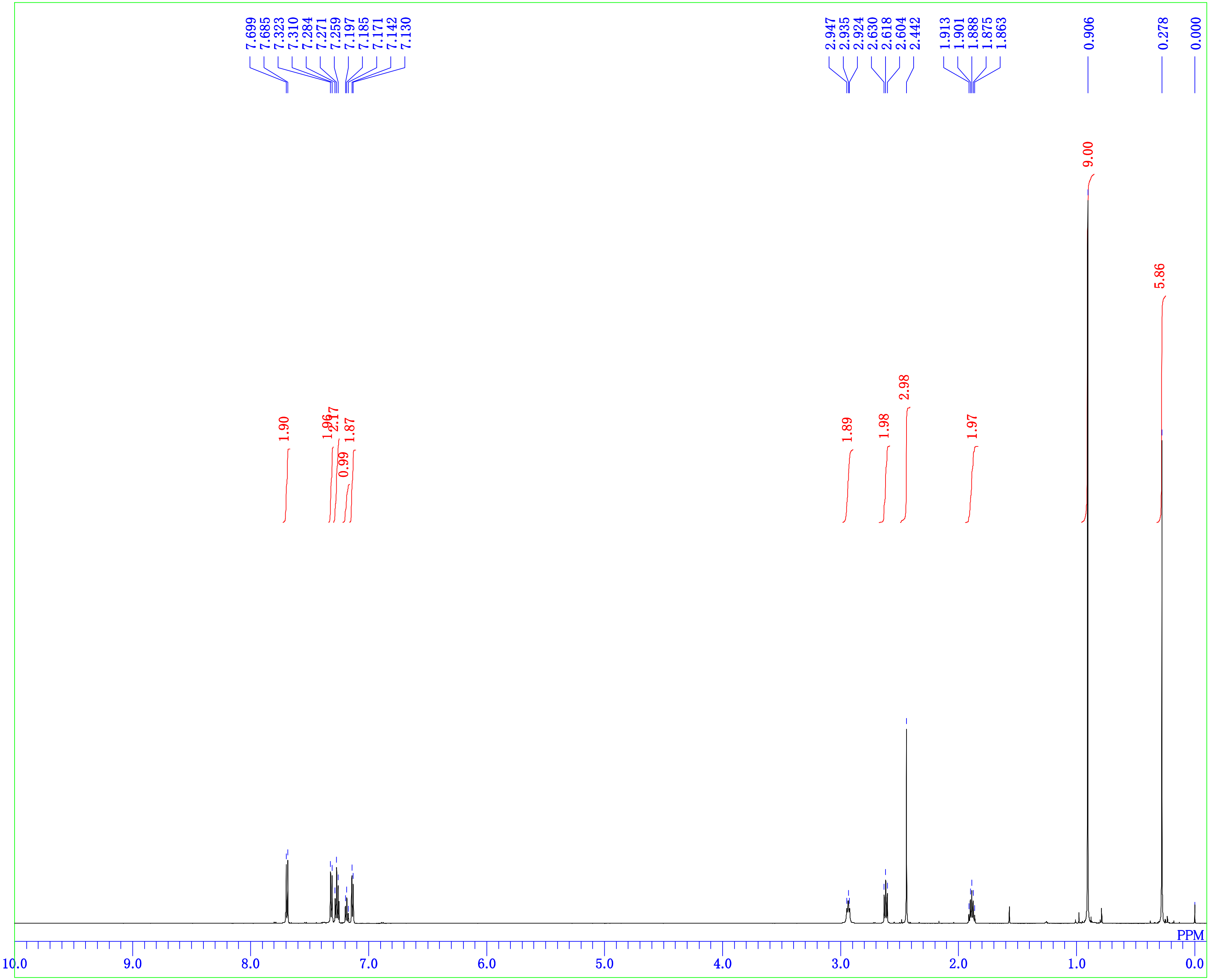

DFILE	otbsnhts-1h-1.als
COMNT	150704
DATIM	2015-02-23 16:57:20
OBNUC	1H
EXMOD	single_pulse.ex2
OBFRQ	600.17 MHz
OBSET	5.30 KHz
OBFIN	5.47 Hz
POINT	26214
FREQU	9008.87 Hz
SCANS	16
ACQTM	2.9098 sec
PD	2.0000 sec
PW1	5.85 usec
IRNUC	1H
CTEMP	20.4 c
SLVNT	CDCL3
EXREF	0.00 ppm
BF	0.12 Hz
RGAIN	30

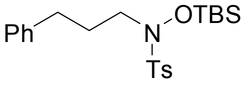

19

150704

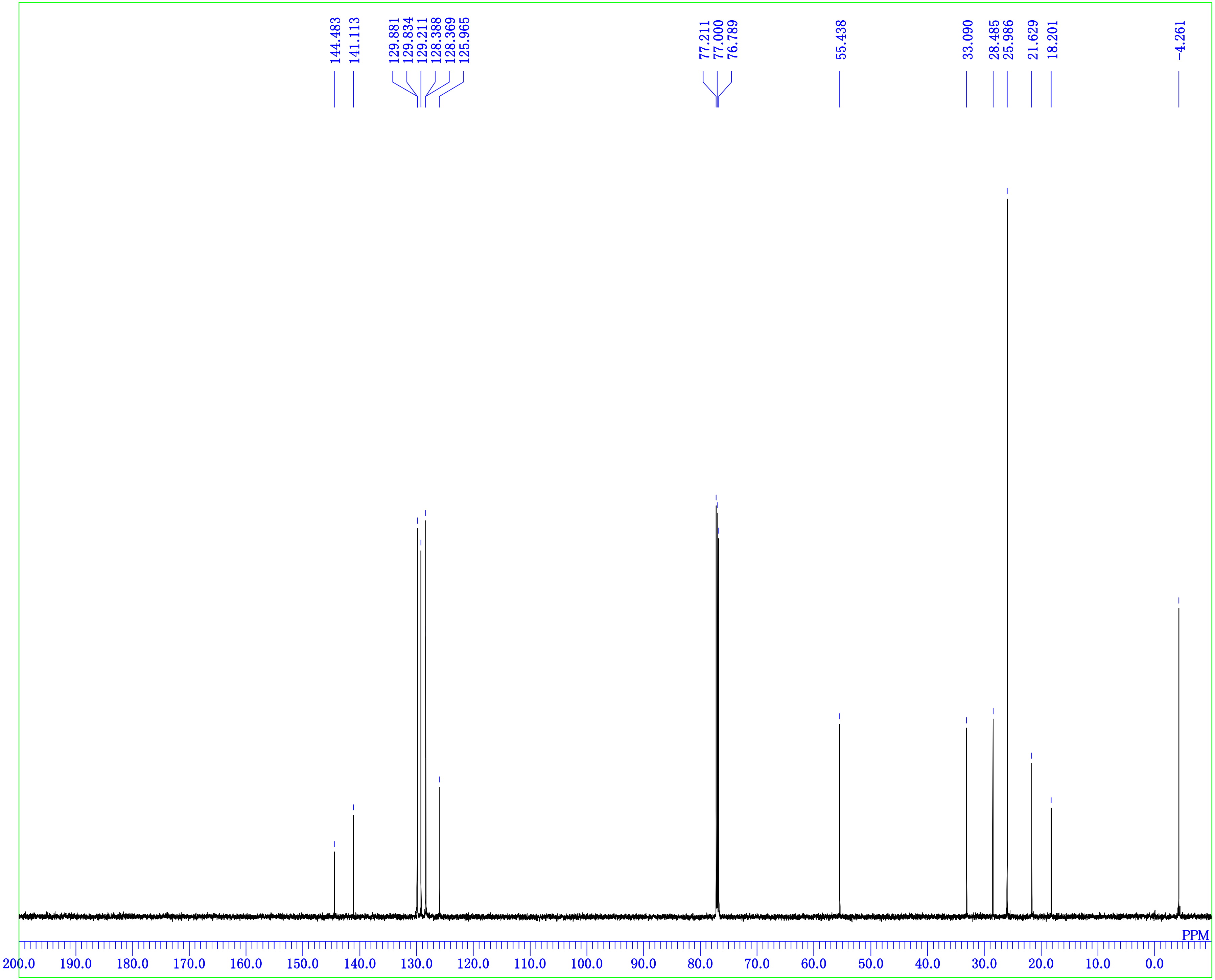

DFILE	otbsnhts-13C-1.als
COMNT	150704
DATIM	2015-02-23 17:09:09
OBNUC	13C
EXMOD	single_pulse_dec
OBFRQ	150.92 MHz
OBSET	8.52 KHz
OBFIN	1.74 Hz
POINT	26214
FREQU	37878.21 Hz
SCANS	256
ACQTM	0.6921 sec
PD	1.2000 sec
PW1	2.97 usec
IRNUC	1H
CTEMP	21.2 c
SLVNT	CDCL3
EXREF	77.00 ppm
BF	1.20 Hz
RGAIN	56

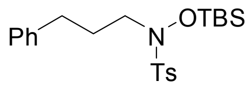

19

151211

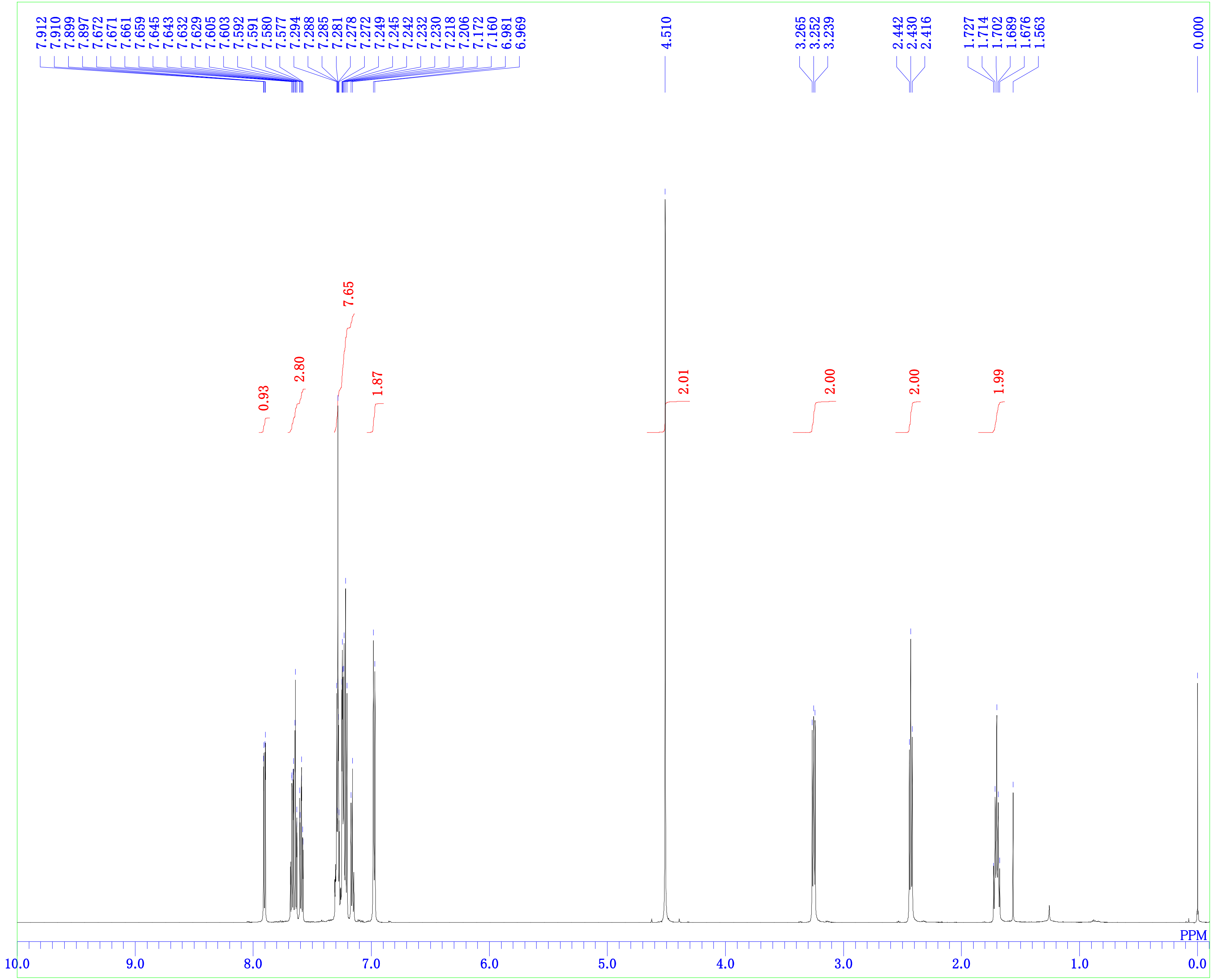

DFILE	d1792-a-1h-1.jdf
COMNT	151211
DATIM	2015-12-11 13:43:54
OBNUC	1H
EXMOD	single_pulse.ex2
OBFRQ	600.17 MHz
OBSET	5.30 KHz
OBFIN	5.47 Hz
POINT	32768
FREQU	11261.26 Hz
SCANS	32
ACQTM	2.9098 sec
PD	2.0000 sec
PW1	7.30 usec
IRNUC	1H
CTEMP	20.3 c
SLVNT	CDCL3
EXREF	0.00 ppm
BF	0.12 Hz
RGAIN	36

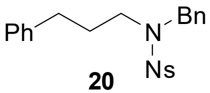

20

151211

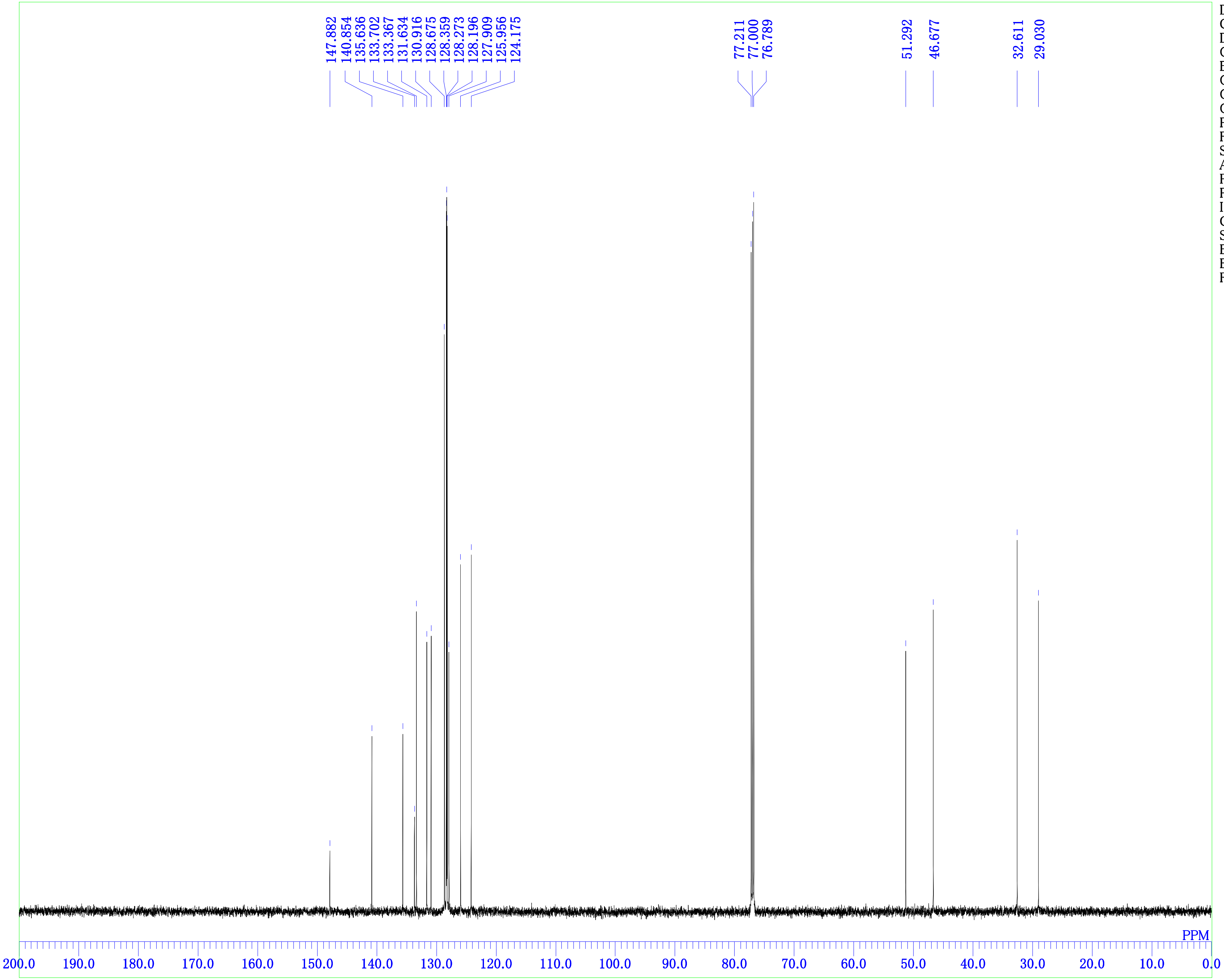

DFILE	d1792-a-13c-1.jdf
COMNT	151211
DATIM	2015-12-11 13:54:33
OBNUC	13C
EXMOD	single_pulse_dec
OBFRQ	150.92 MHz
OBSET	8.52 KHz
OBFIN	1.74 Hz
POINT	32768
FREQU	47348.49 Hz
SCANS	256
ACQTM	0.6921 sec
PD	1.2000 sec
PW1	3.17 usec
IRNUC	1H
CTEMP	21.0 c
SLVNT	CDCL3
EXREF	77.00 ppm
BF	1.20 Hz
RGAIN	54

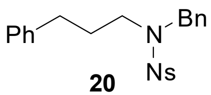

20

150704

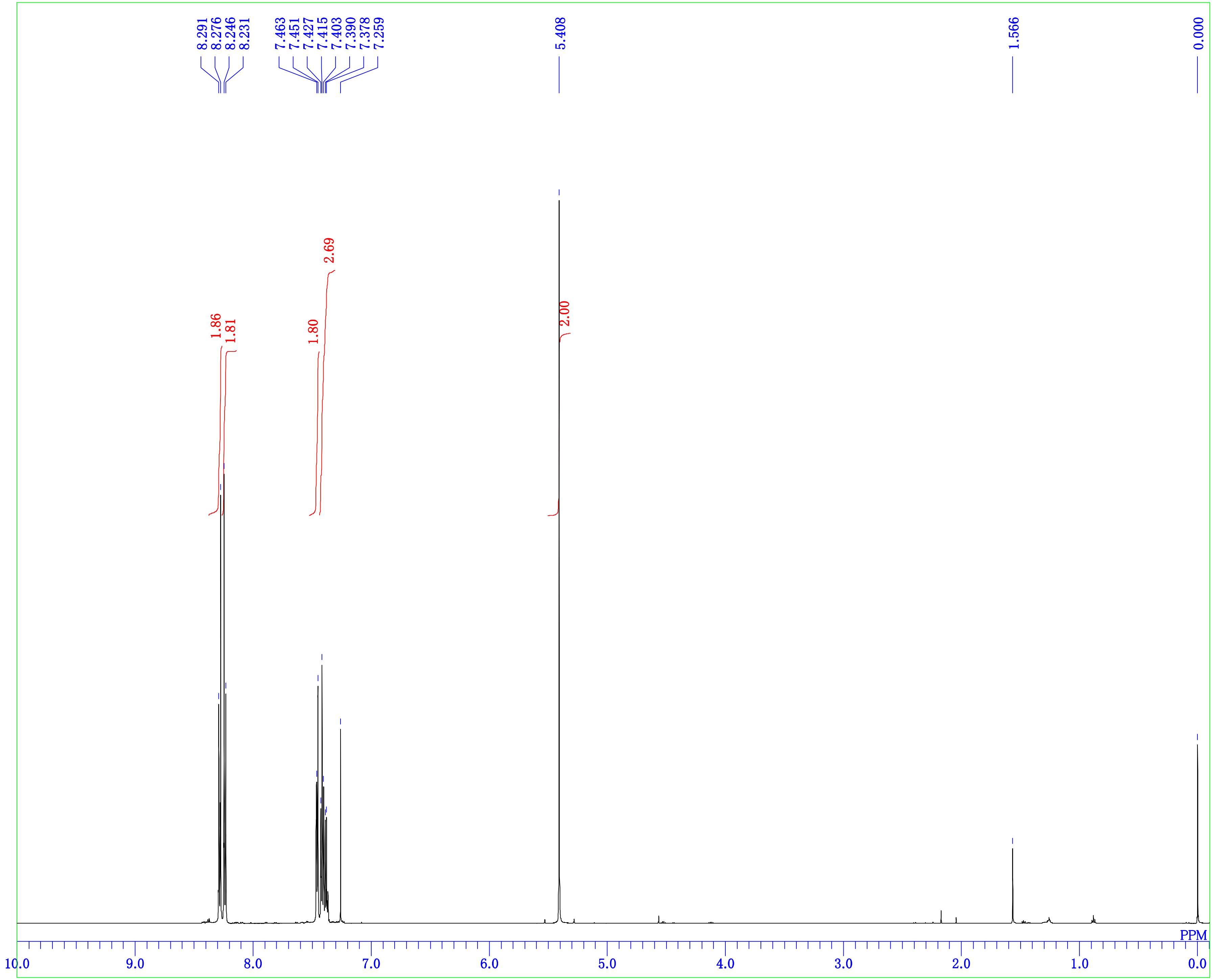

DFILE	d1542-gra-1h-1.als
COMNT	150704
DATIM	2015-04-02 14:45:45
OBNUC	1H
EXMOD	single_pulse.ex2
OBFRQ	600.17 MHz
OBSET	5.30 KHz
OBFIN	5.47 Hz
POINT	26214
FREQU	9008.87 Hz
SCANS	32
ACQTM	2.9098 sec
PD	2.0000 sec
PW1	7.30 usec
IRNUC	1H
CTEMP	20.9 c
SLVNT	CDCL3
EXREF	0.00 ppm
BF	0.12 Hz
RGAIN	44

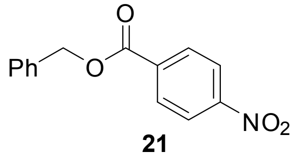

21

150704

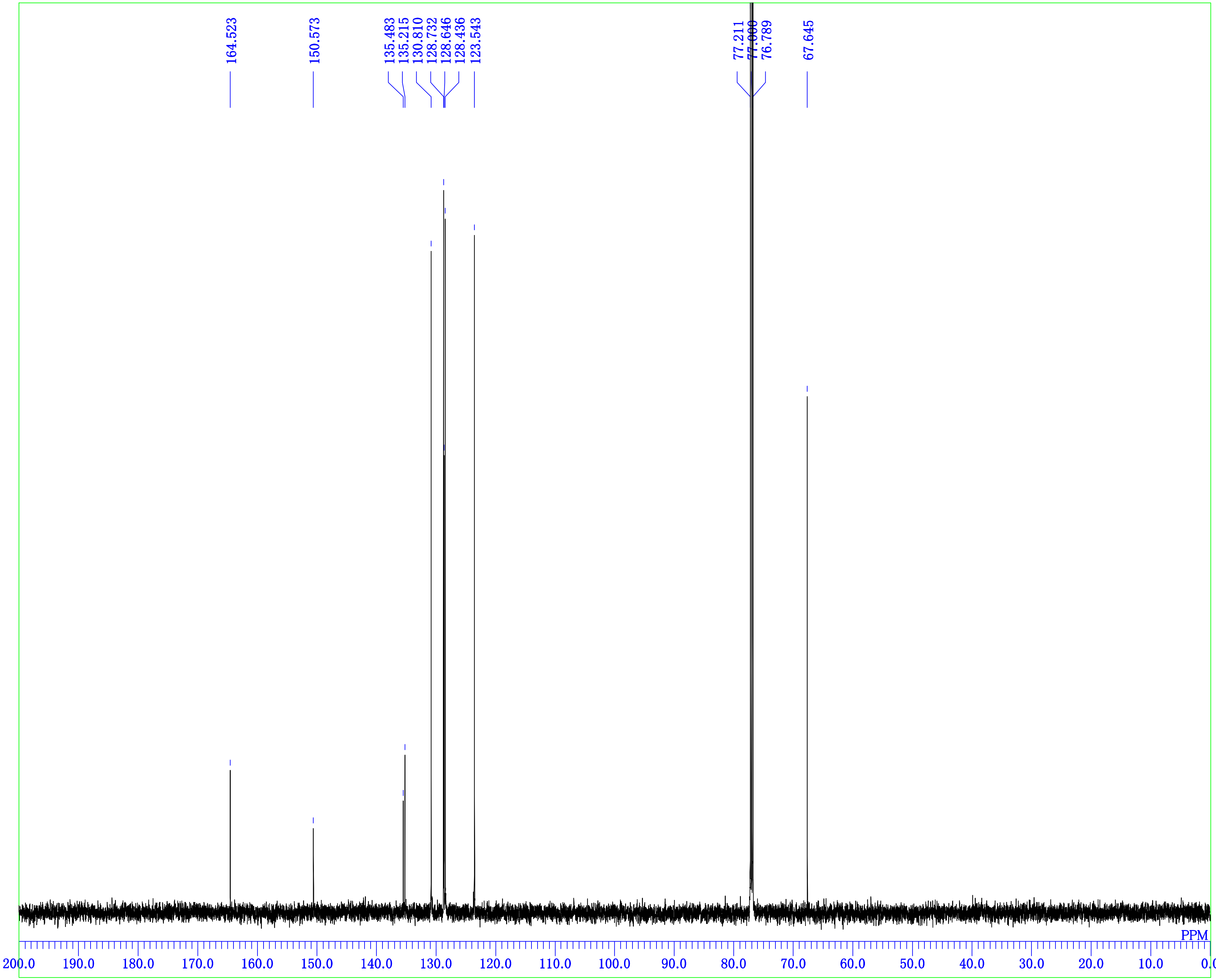

DFILE	d1542-gra-13c-1.als
COMNT	150704
DATIM	2015-04-02 14:54:25
OBNUC	13C
EXMOD	single_pulse_dec
OBFRQ	150.92 MHz
OBSET	8.52 KHz
OBFIN	1.74 Hz
POINT	26214
FREQU	37878.21 Hz
SCANS	256
ACQTM	0.6921 sec
PD	1.2000 sec
PW1	3.13 usec
IRNUC	1H
CTEMP	21.8 c
SLVNT	CDCL3
EXREF	77.00 ppm
BF	1.20 Hz
RGAIN	56

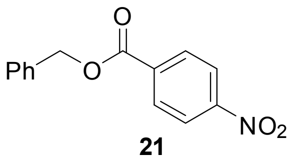

21

150704

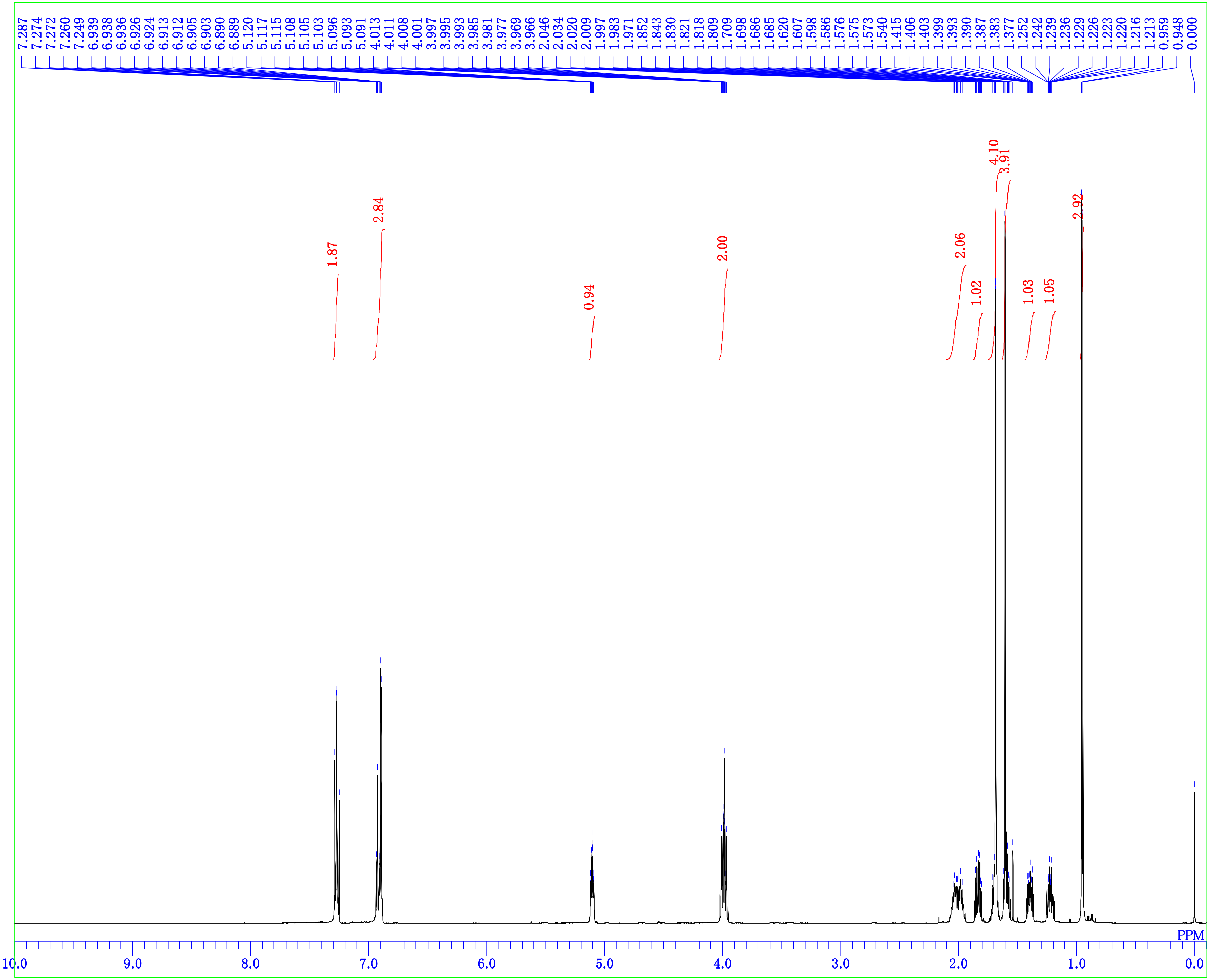

DFILE	d1535-gra-1h-1.als
COMNT	150704
DATIM	2015-03-24 21:33:37
OBNUC	1H
EXMOD	single_pulse.ex2
OBFRQ	600.17 MHz
OBSET	5.30 KHz
OBFIN	5.47 Hz
POINT	26214
FREQU	9008.87 Hz
SCANS	32
ACQTM	2.9098 sec
PD	2.0000 sec
PW1	7.30 usec
IRNUC	1H
CTEMP	20.2 c
SLVNT	CDCL3
EXREF	0.00 ppm
BF	0.12 Hz
RGAIN	36

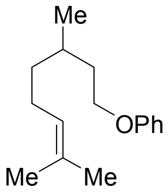

22

150704

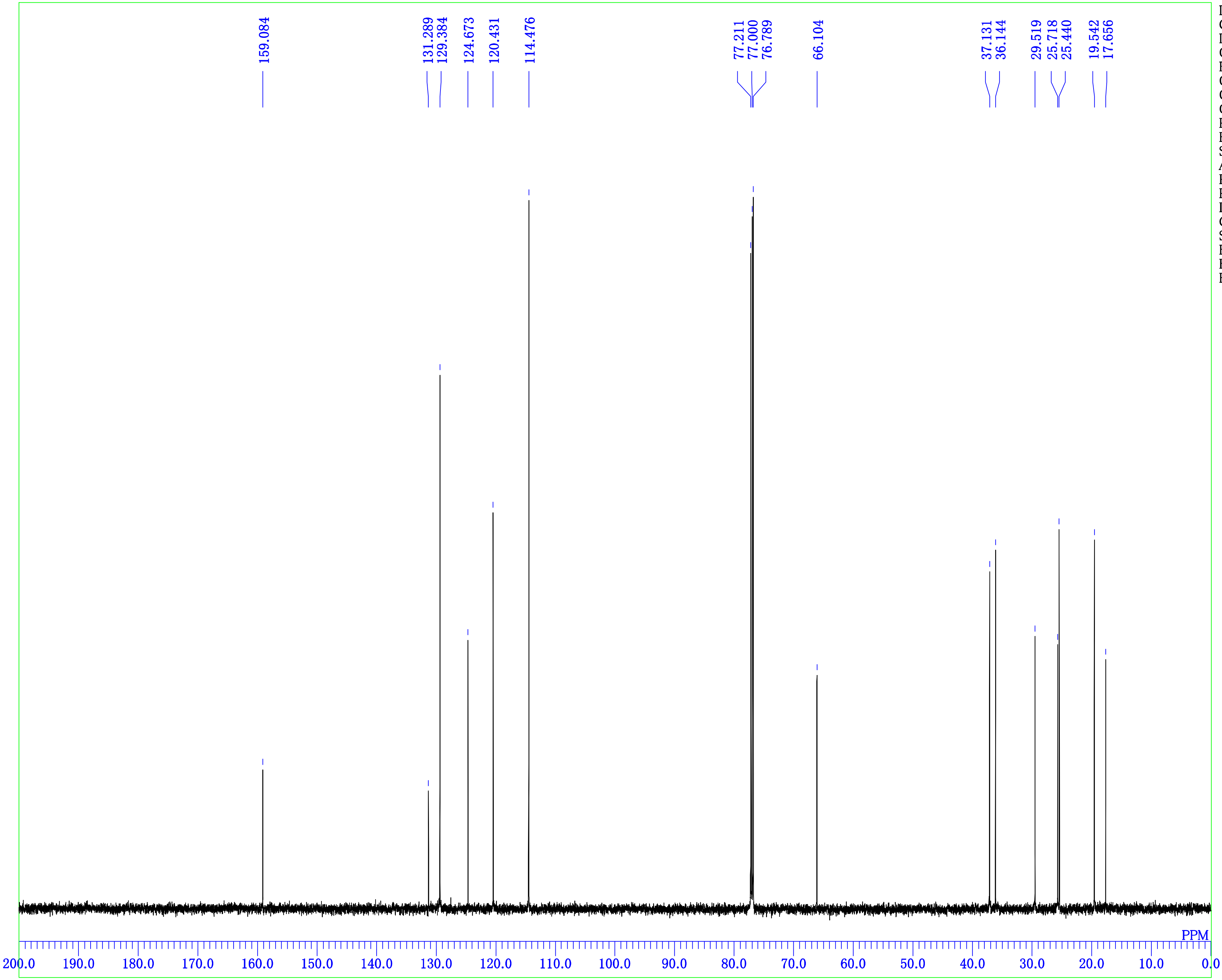

DFILE	d1535-gra-13c-1.als
COMNT	150704
DATIM	2015-03-24 21:42:15
OBNUC	13C
EXMOD	single_pulse_dec
OBFRQ	150.92 MHz
OBSET	8.52 KHz
OBFIN	1.74 Hz
POINT	26214
FREQU	37878.21 Hz
SCANS	256
ACQTM	0.6921 sec
PD	1.2000 sec
PW1	3.13 usec
IRNUC	1H
CTEMP	20.9 c
SLVNT	CDCL3
EXREF	77.00 ppm
BF	1.20 Hz
RGAIN	56

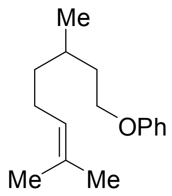

22

150704

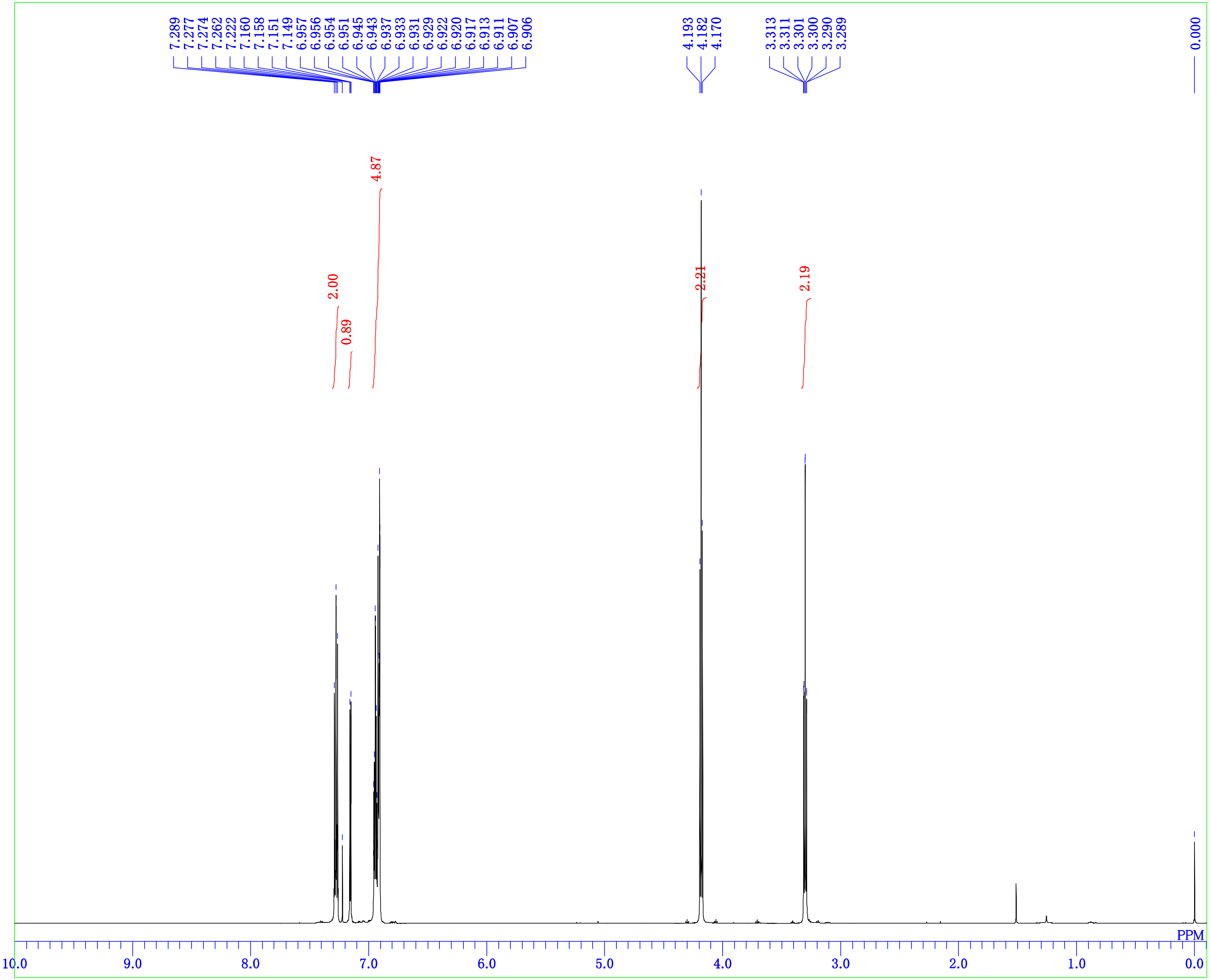

DFILE	d1526-gra-1h-thio-1.als
COMNT	150704
DATIM	2015-03-20 10:34:22
OBNUC	1H
EXMOD	single_pulse.ex2
OBFRQ	600.17 MHz
OBSET	5.30 KHz
OBFIN	5.47 Hz
POINT	26214
FREQU	9008.87 Hz
SCANS	32
ACQTM	2.9098 sec
PD	2.0000 sec
PW1	7.30 usec
IRNUC	1H
CTEMP	20.9 c
SLVNT	CDCL3
EXREF	0.00 ppm
BF	0.12 Hz
RGAIN	34

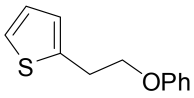

23

150704

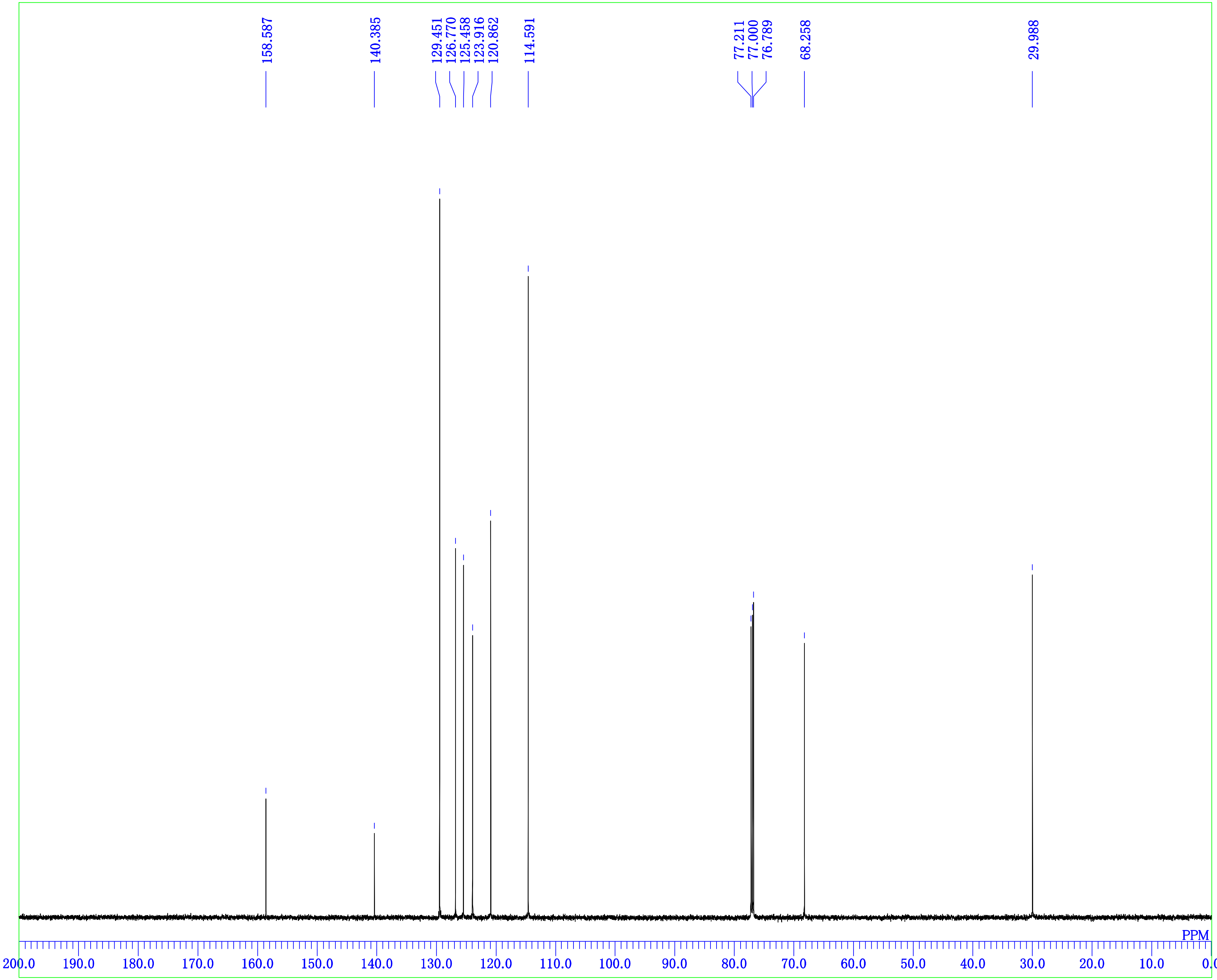

DFILE	d1526-gra-13c-thio-1.als
COMNT	150704
DATIM	2015-03-20 10:42:58
OBNUC	13C
EXMOD	single_pulse_dec
OBFRQ	150.92 MHz
OBSET	8.52 KHz
OBFIN	1.74 Hz
POINT	26214
FREQU	37878.21 Hz
SCANS	256
ACQTM	0.6921 sec
PD	1.2000 sec
PW1	3.13 usec
IRNUC	1H
CTEMP	21.8 c
SLVNT	CDCL3
EXREF	77.00 ppm
BF	1.20 Hz
RGAIN	56

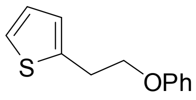

23

150704

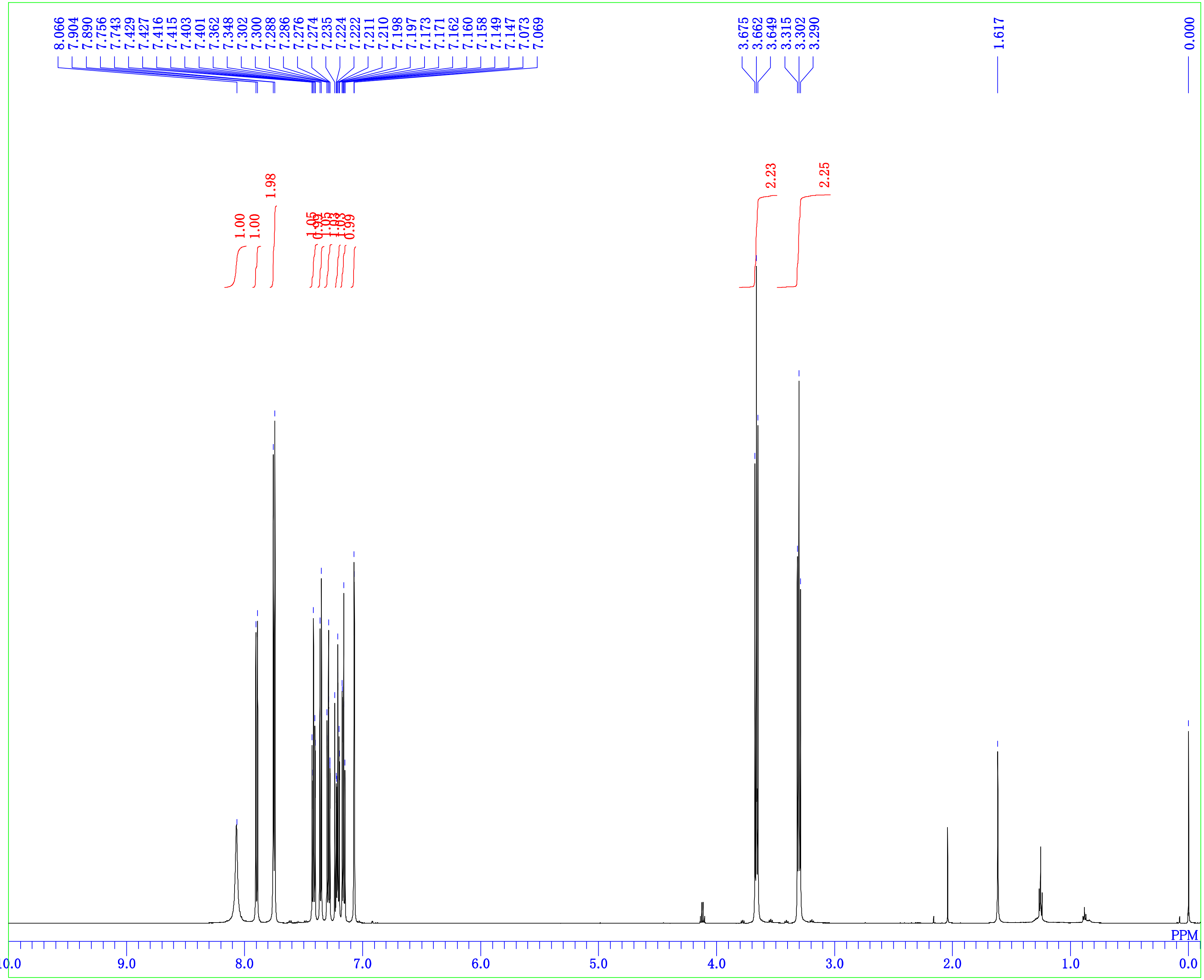

DFILE	d1589-gra-1h-1.als
COMNT	150704
DATIM	2015-05-24 13:04:38
OBNUC	1H
EXMOD	single_pulse.ex2
OBFRQ	600.17 MHz
OBSET	5.30 KHz
OBFIN	5.47 Hz
POINT	26214
FREQU	9008.87 Hz
SCANS	32
ACQTM	2.9098 sec
PD	2.0000 sec
PW1	7.30 usec
IRNUC	1H
CTEMP	22.6 c
SLVNT	CDCL3
EXREF	0.00 ppm
BF	0.12 Hz
RGAIN	40

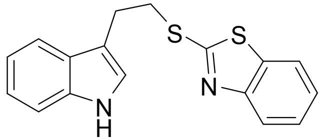

24

150704

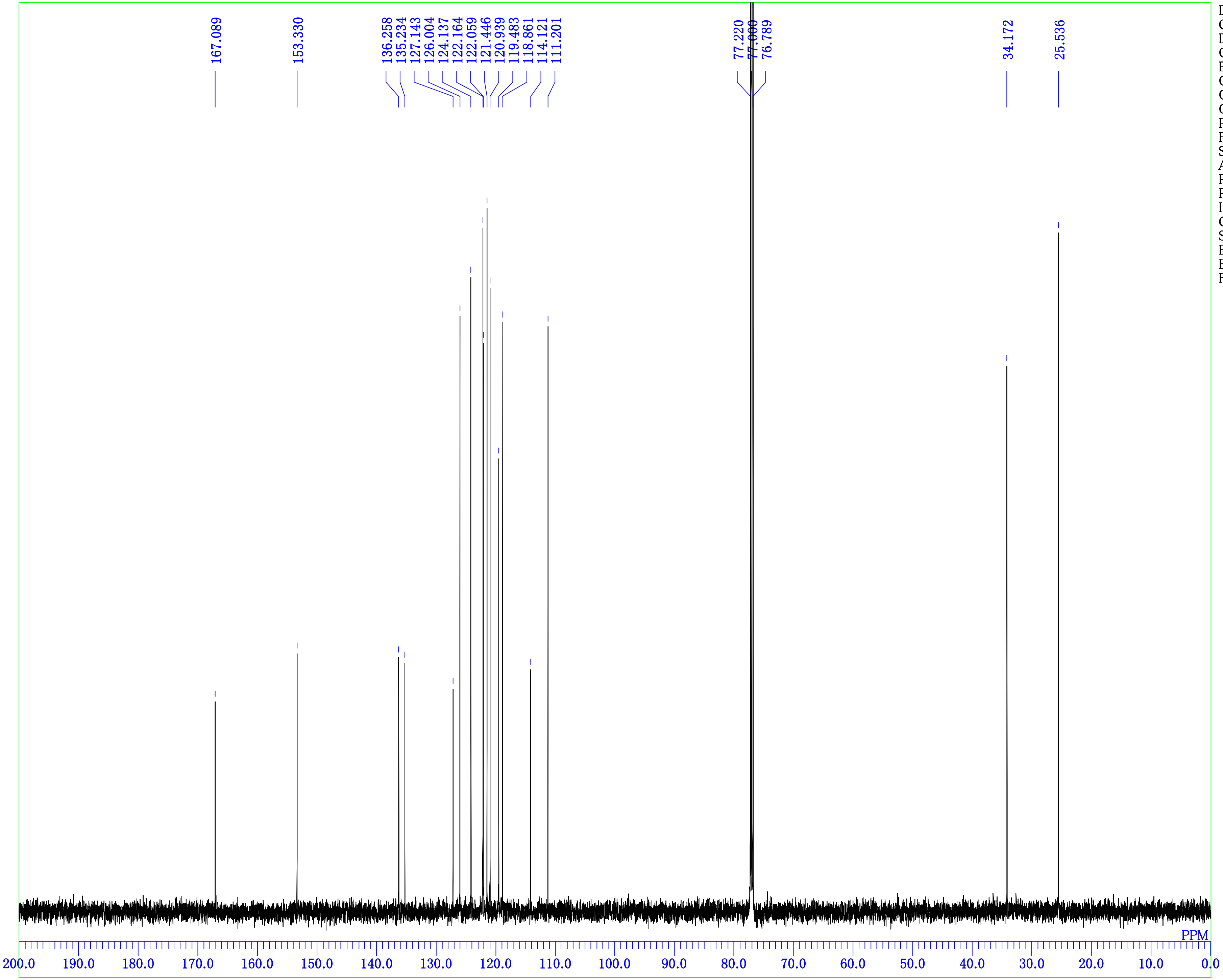

DFILE	d1589-gra-13c-1.als
COMNT	150704
DATIM	2015-05-24 13:14:46
OBNUC	13C
EXMOD	single_pulse_dec
OBFRQ	150.92 MHz
OBSET	8.52 KHz
OBFIN	1.74 Hz
POINT	26214
FREQU	37878.21 Hz
SCANS	256
ACQTM	0.6921 sec
PD	1.2000 sec
PW1	3.13 usec
IRNUC	1H
CTEMP	23.4 c
SLVNT	CDCL3
EXREF	77.00 ppm
BF	1.20 Hz
RGAIN	56

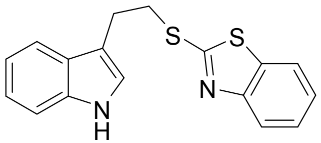

24

150519

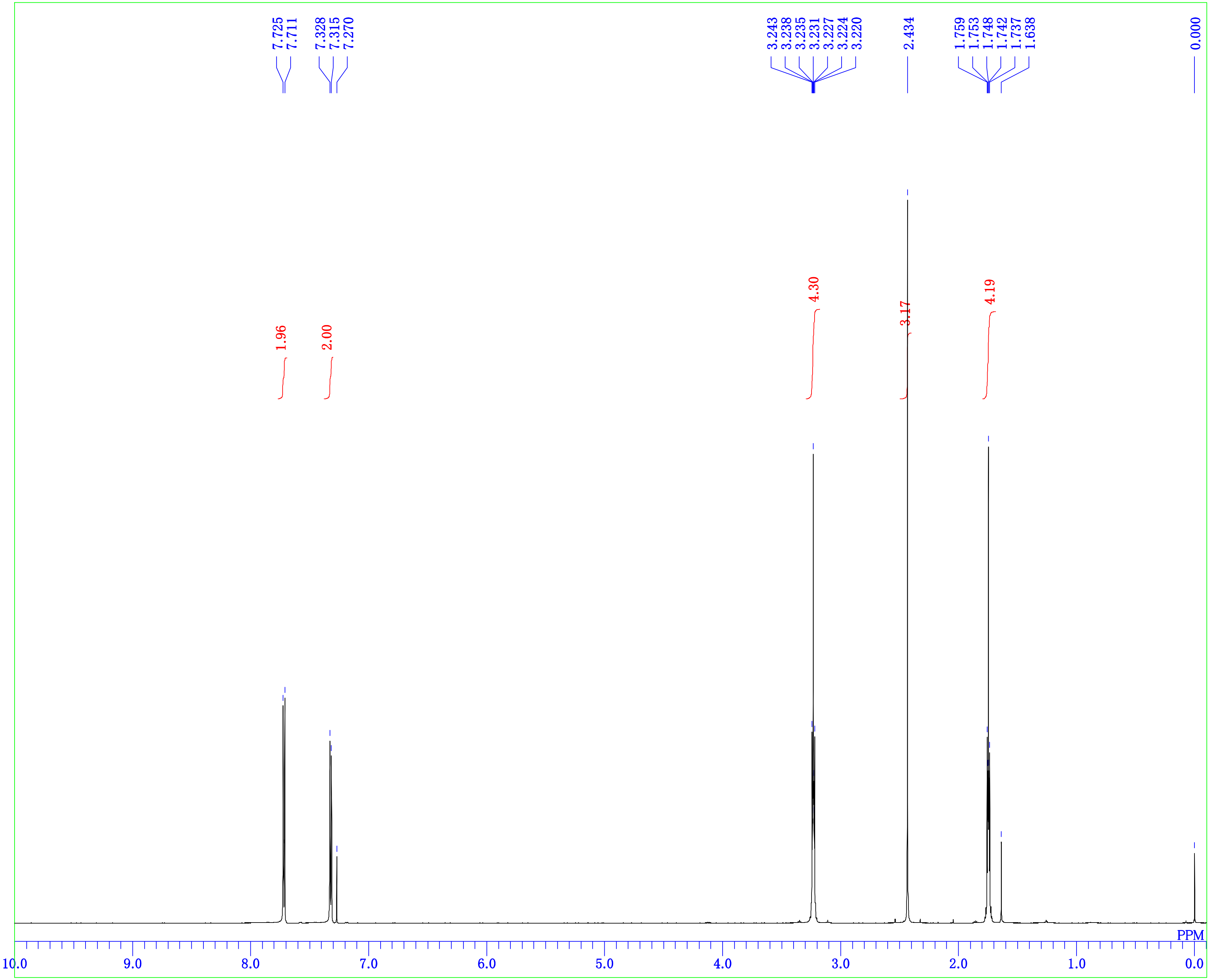

DFILE	d1583-gra-1h-1.als
COMNT	150519
DATIM	2015-05-19 18:45:02
OBNUC	1H
EXMOD	single_pulse.ex2
OBFRQ	600.17 MHz
OBSET	5.30 KHz
OBFIN	5.47 Hz
POINT	26214
FREQU	9008.87 Hz
SCANS	32
ACQTM	2.9098 sec
PD	2.0000 sec
PW1	7.30 usec
IRNUC	1H
CTEMP	21.4 c
SLVNT	CDCL3
EXREF	0.00 ppm
BF	0.12 Hz
RGAIN	38

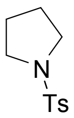

25

150519

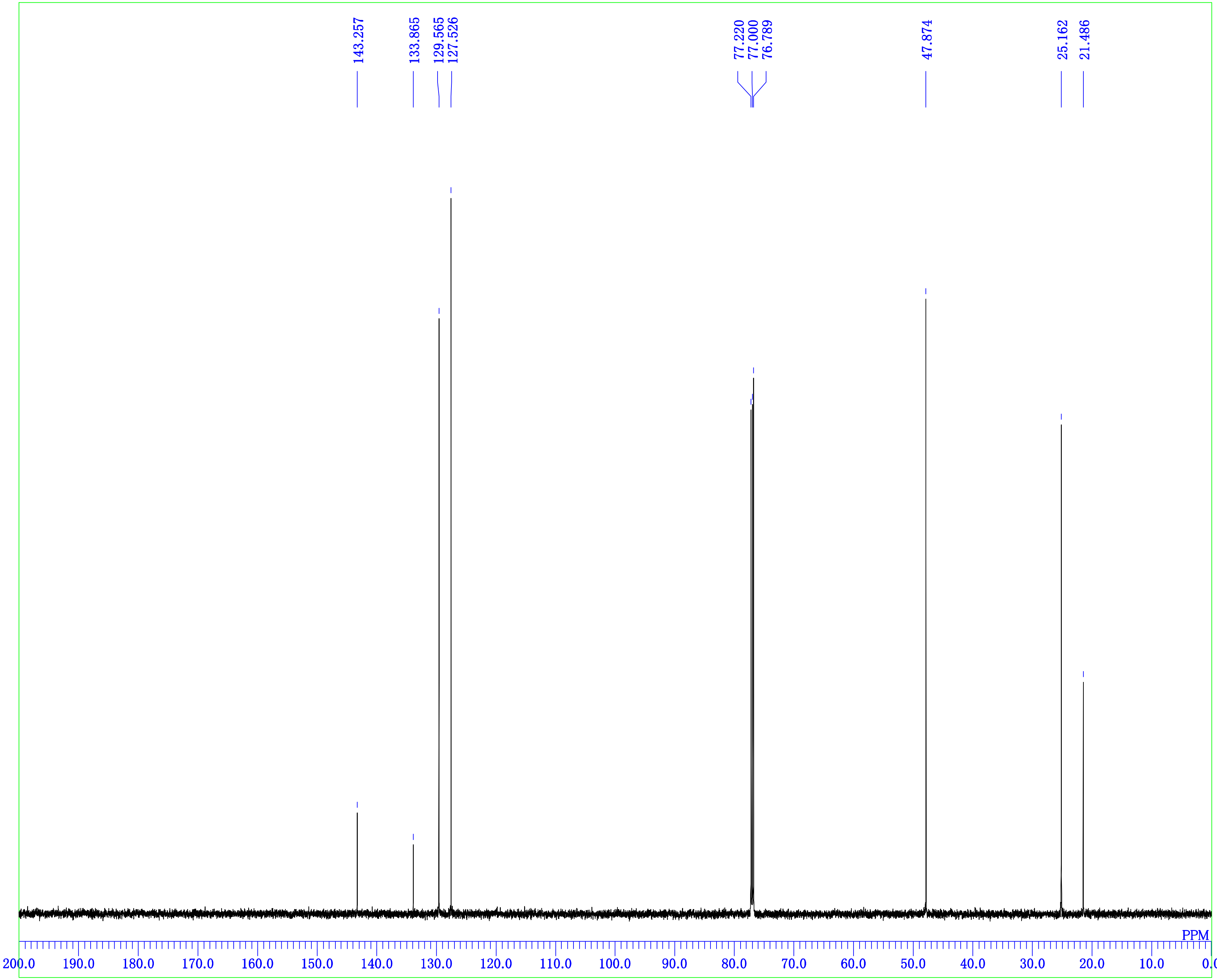

DFILE	d1583-gra-13c-1.als
COMNT	150519
DATIM	2015-05-19 18:53:36
OBNUC	13C
EXMOD	single_pulse_dec
OBFRQ	150.92 MHz
OBSET	8.52 KHz
OBFIN	1.74 Hz
POINT	26214
FREQU	37878.21 Hz
SCANS	256
ACQTM	0.6921 sec
PD	1.2000 sec
PW1	3.13 usec
IRNUC	1H
CTEMP	21.9 c
SLVNT	CDCL3
EXREF	77.00 ppm
BF	1.20 Hz
RGAIN	54

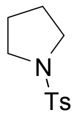

25

150727

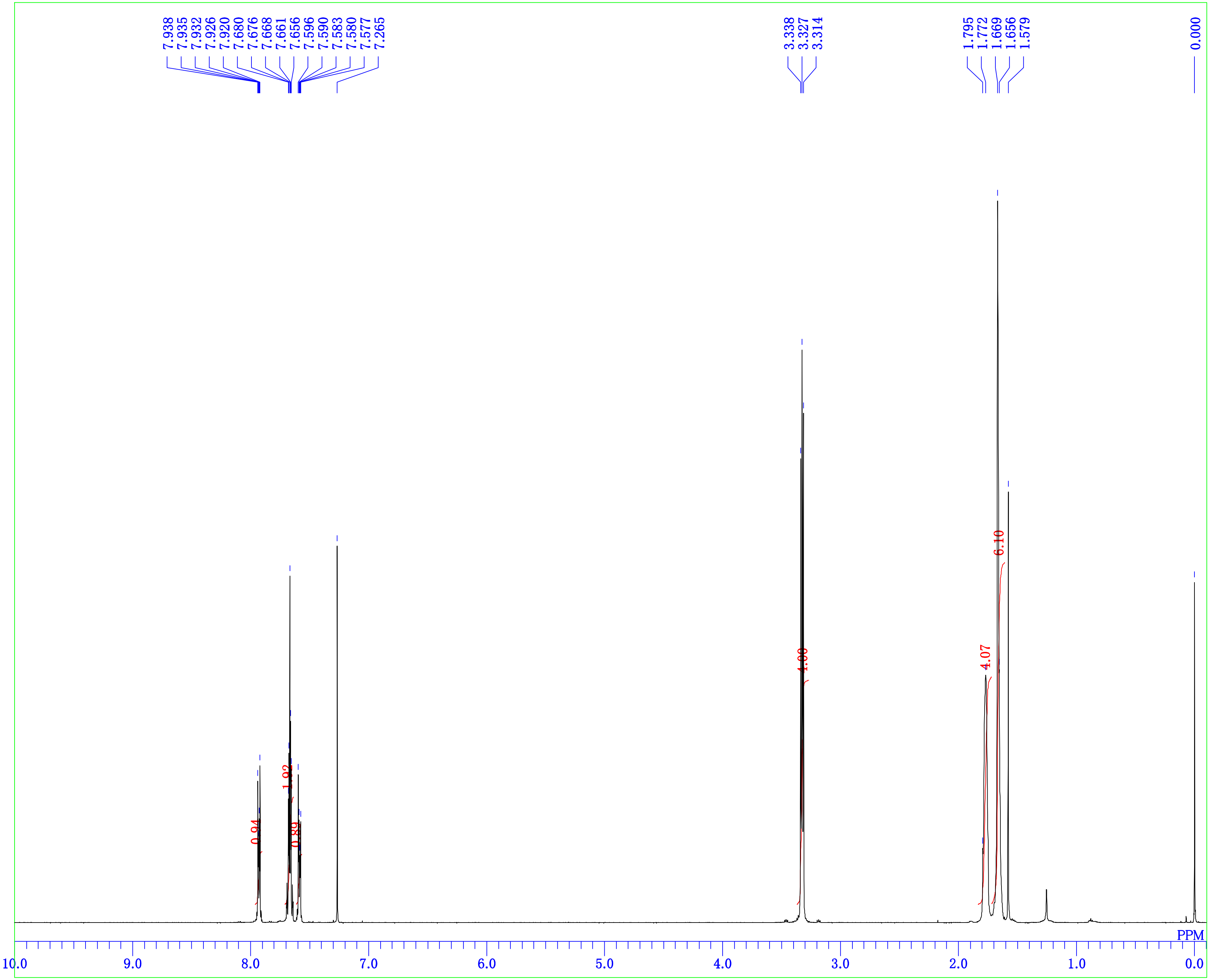

DFILE	d1671-gra-1h-1.als
COMNT	150727
DATIM	2015-07-27 08:50:01
OBNUC	1H
EXMOD	single_pulse.ex2
OBFRQ	500.16 MHz
OBSET	2.41 KHz
OBFIN	6.01 Hz
POINT	13107
FREQU	7507.39 Hz
SCANS	32
ACQTM	1.7459 sec
PD	2.0000 sec
PW1	5.80 usec
IRNUC	1H
CTEMP	20.1 c
SLVNT	CDCL3
EXREF	0.00 ppm
BF	0.12 Hz
RGAIN	52

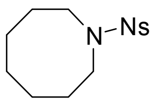

26

150727

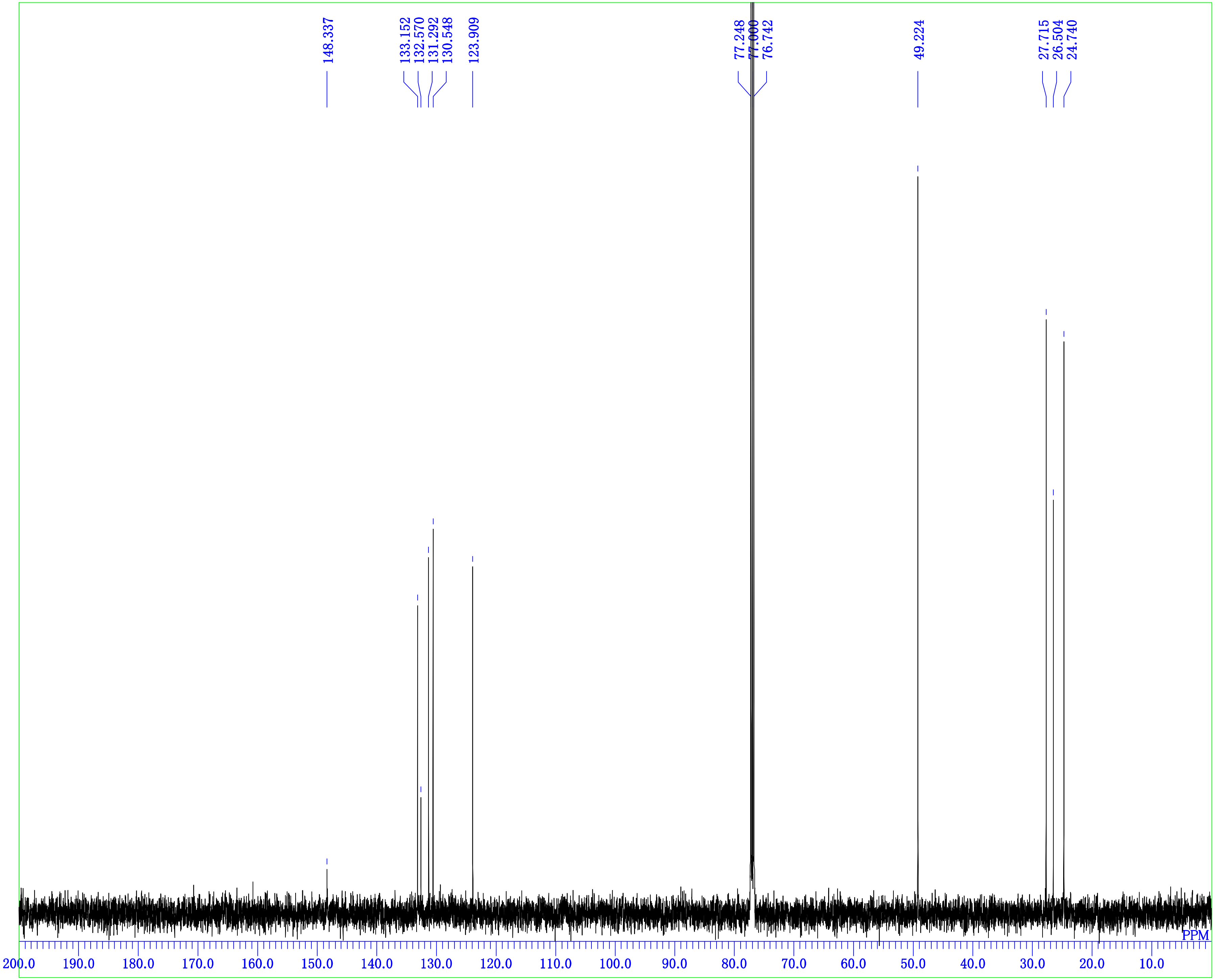

DFILE	d1671-gra-13c-1.als
COMNT	150727
DATIM	2015-07-27 09:04:03
OBNUC	13C
EXMOD	single_pulse_dec
OBFRQ	125.77 MHz
OBSET	7.87 KHz
OBFIN	4.21 Hz
POINT	26214
FREQU	31446.06 Hz
SCANS	256
ACQTM	0.8336 sec
PD	2.0000 sec
PW1	3.00 usec
IRNUC	1H
CTEMP	21.3 c
SLVNT	CDCL3
EXREF	77.00 ppm
BF	1.20 Hz
RGAIN	56

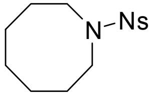

26

150306

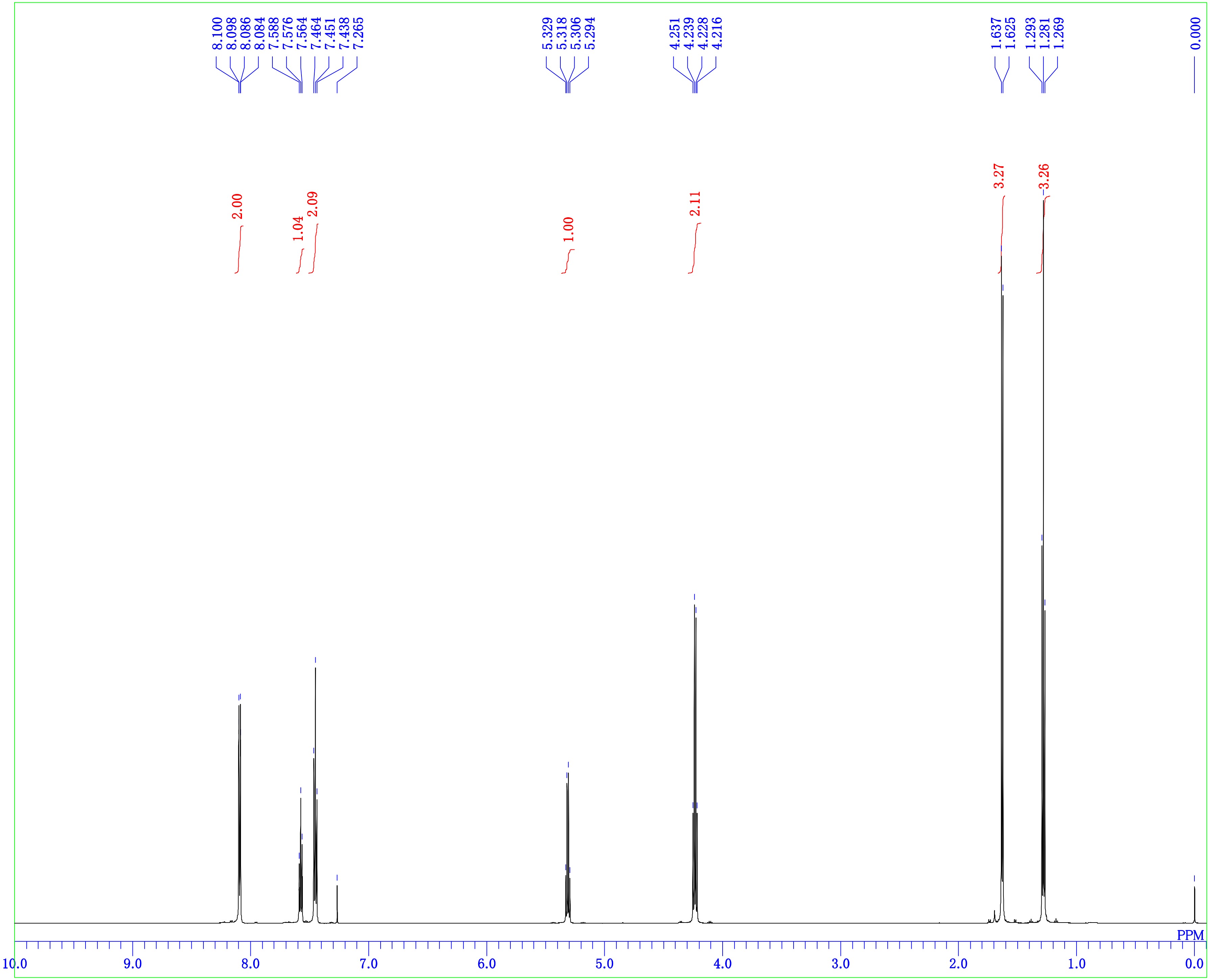

DFILE	d1502-gra-1h-1.als
COMNT	150306
DATIM	2015-03-06 16:06:43
OBNUC	1H
EXMOD	single_pulse.ex2
OBFRQ	600.17 MHz
OBSET	5.30 KHz
OBFIN	5.47 Hz
POINT	26214
FREQU	9008.87 Hz
SCANS	32
ACQTM	2.9098 sec
PD	2.0000 sec
PW1	5.85 usec
IRNUC	1H
CTEMP	20.2 c
SLVNT	CDCL3
EXREF	0.00 ppm
BF	0.12 Hz
RGAIN	30

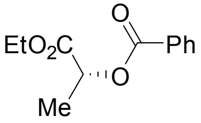

34

150306

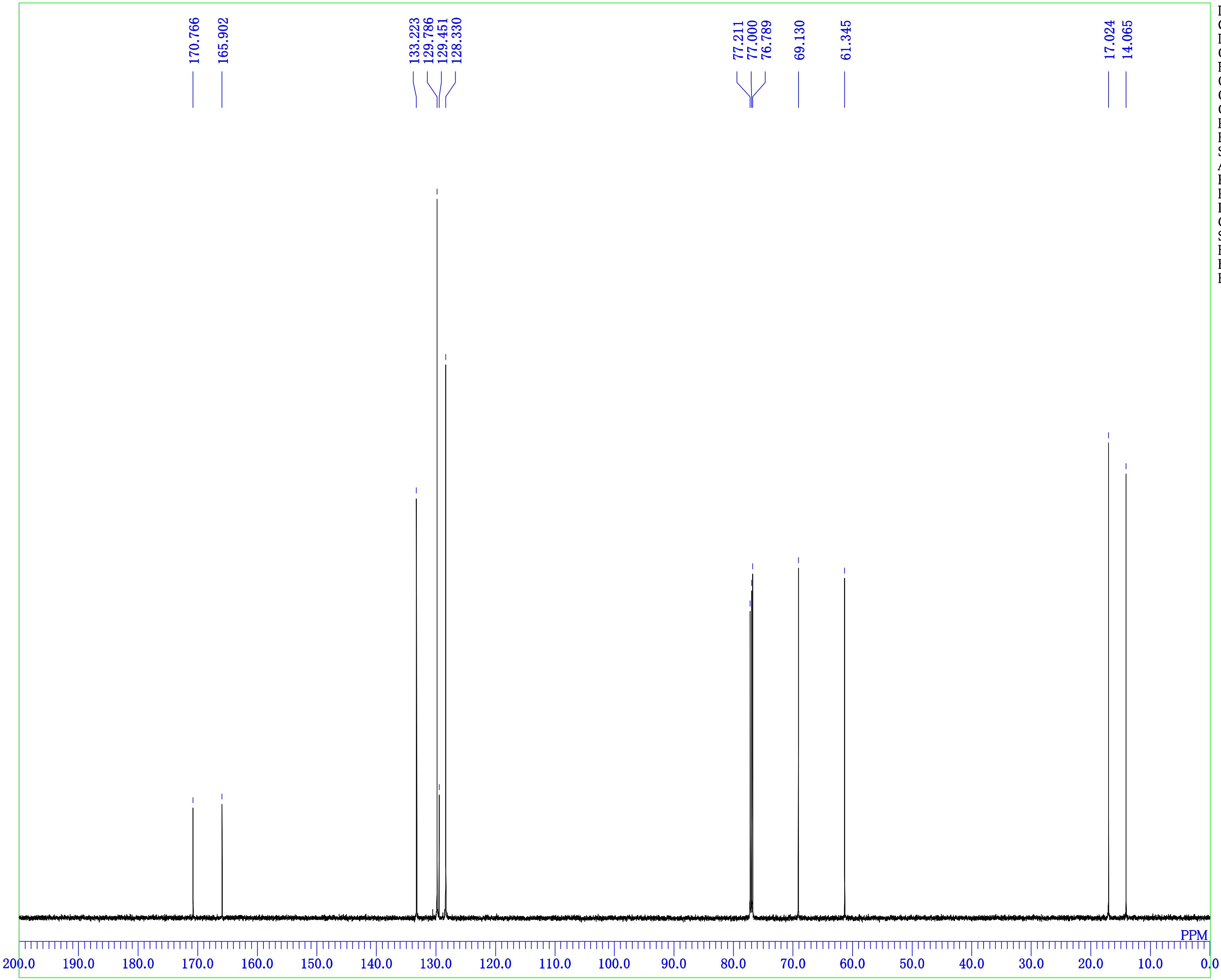

DFILE	d1502-gra-13c-1.als
COMNT	150306
DATIM	2015-03-06 16:15:25
OBNUC	13C
EXMOD	single_pulse_dec
OBFRQ	150.92 MHz
OBSET	8.52 KHz
OBFIN	1.74 Hz
POINT	26214
FREQU	37878.21 Hz
SCANS	256
ACQTM	0.6921 sec
PD	1.2000 sec
PW1	2.97 usec
IRNUC	1H
CTEMP	20.8 c
SLVNT	CDCL3
EXREF	77.00 ppm
BF	1.20 Hz
RGAIN	56

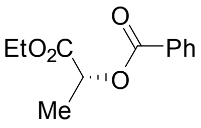

34

150621

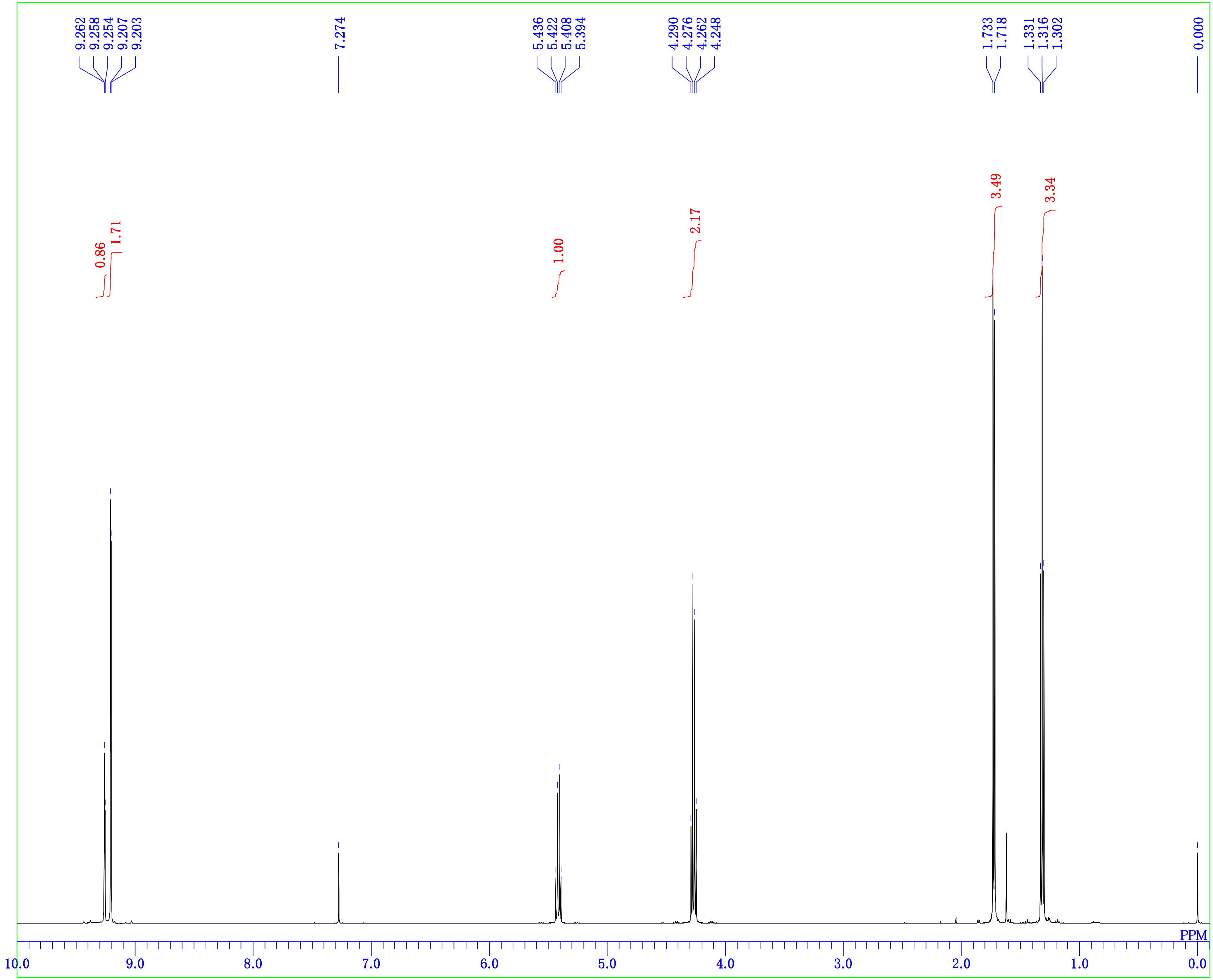

DFILE	d1609-gra-1h-1.als
COMNT	150621
DATIM	2015-06-21 17:14:42
OBNUC	1H
EXMOD	single_pulse.ex2
OBFRQ	500.16 MHz
OBSET	2.41 KHz
OBFIN	6.01 Hz
POINT	13107
FREQU	7507.39 Hz
SCANS	32
ACQTM	1.7459 sec
PD	2.0000 sec
PW1	5.80 usec
IRNUC	1H
CTEMP	20.5 c
SLVNT	CDCL3
EXREF	0.00 ppm
BF	0.12 Hz
RGAIN	40

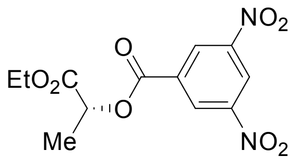

35

150621

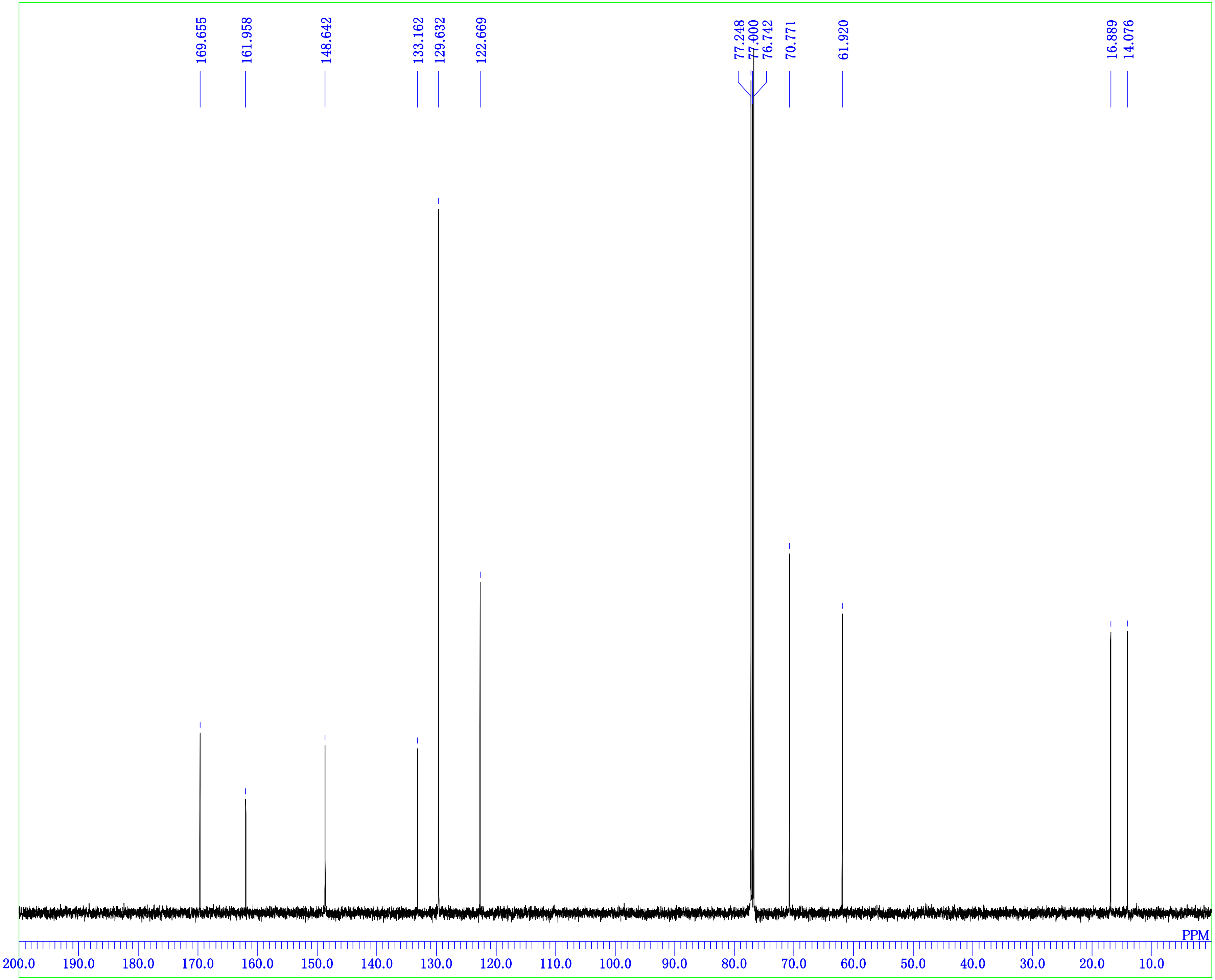

DFILE	d1609-gra-13c-1.als
COMNT	150621
DATIM	2015-06-21 17:27:43
OBNUC	13C
EXMOD	single_pulse_dec
OBFRQ	125.77 MHz
OBSET	7.87 KHz
OBFIN	4.21 Hz
POINT	26214
FREQU	31446.06 Hz
SCANS	256
ACQTM	0.8336 sec
PD	2.0000 sec
PW1	3.00 usec
IRNUC	1H
CTEMP	21.3 c
SLVNT	CDCL3
EXREF	77.00 ppm
BF	1.20 Hz
RGAIN	58

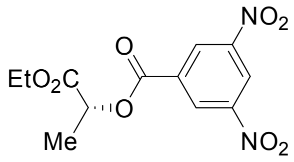

35

150406

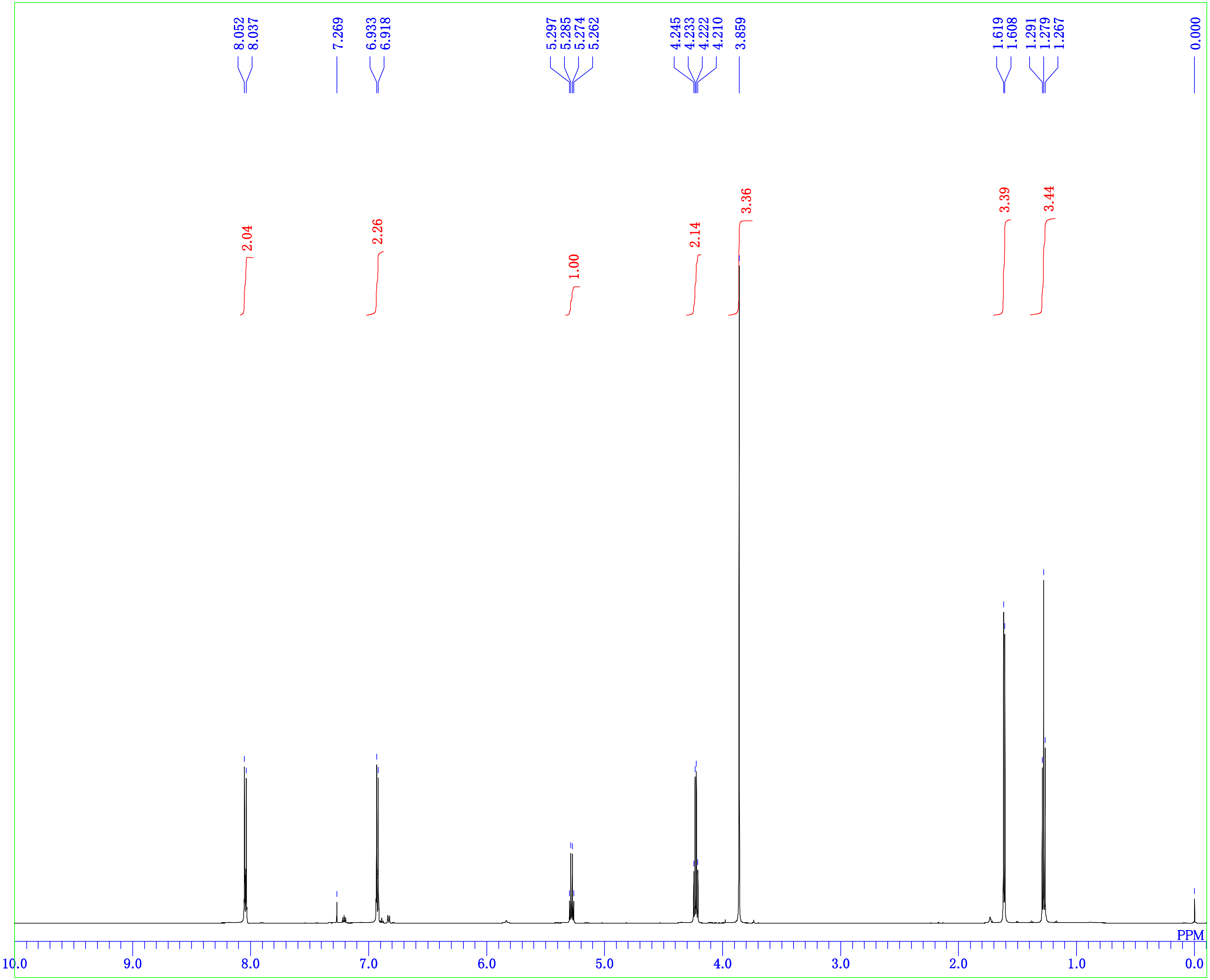

DFILE	d1546-gra-1h-1.als
COMNT	150406
DATIM	2015-04-06 15:11:47
OBNUC	1H
EXMOD	single_pulse.ex2
OBFRQ	600.17 MHz
OBSET	5.30 KHz
OBFIN	5.47 Hz
POINT	26214
FREQU	9008.87 Hz
SCANS	32
ACQTM	2.9098 sec
PD	2.0000 sec
PW1	7.30 usec
IRNUC	1H
CTEMP	20.9 c
SLVNT	CDCL3
EXREF	0.00 ppm
BF	0.12 Hz
RGAIN	30

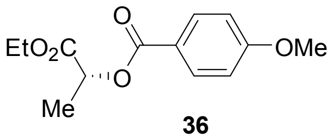

36

150406

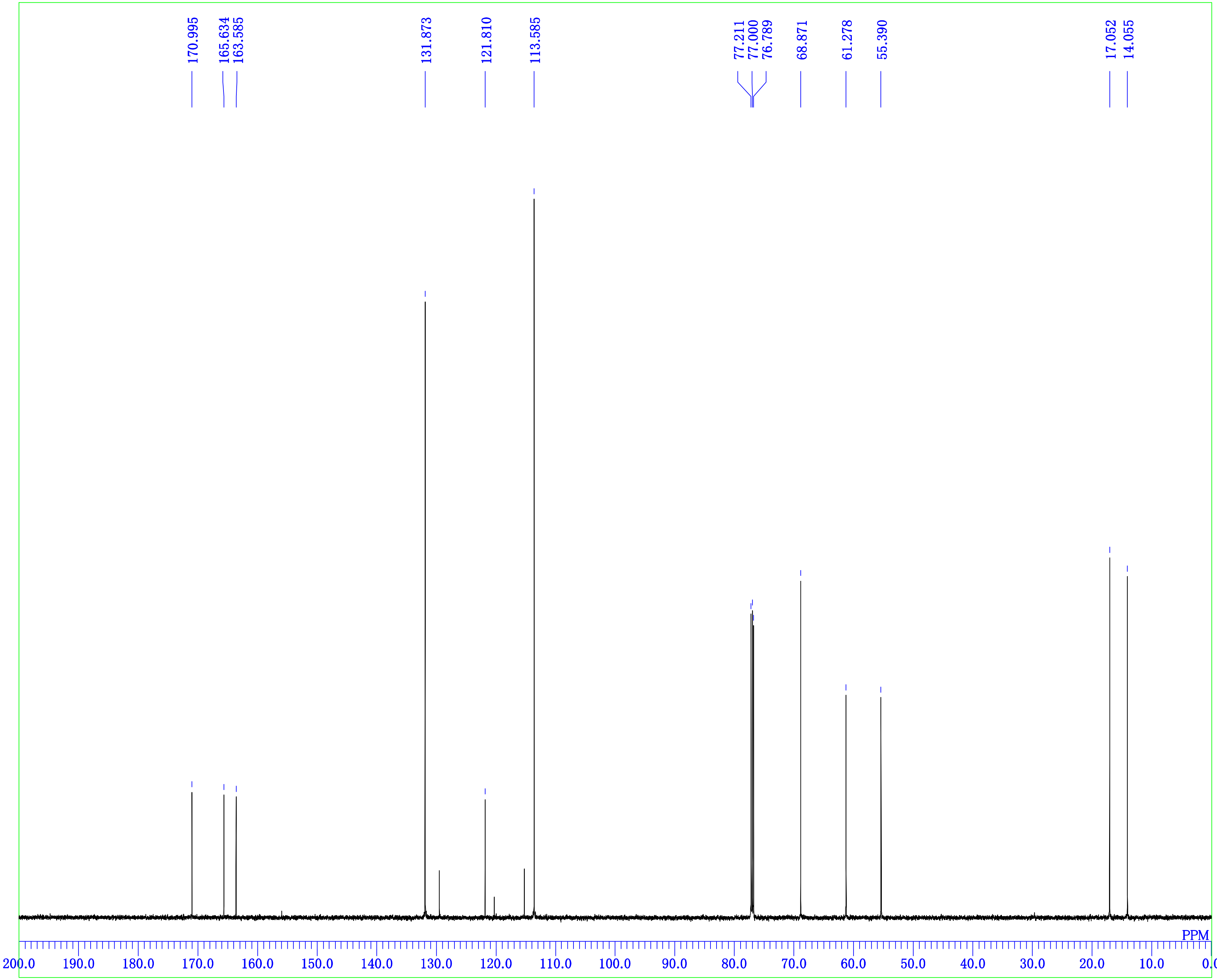

DFILE	d1546-gra-13c-1.als
COMNT	150406
DATIM	2015-04-06 15:20:27
OBNUC	13C
EXMOD	single_pulse_dec
OBFRQ	150.92 MHz
OBSET	8.52 KHz
OBFIN	1.74 Hz
POINT	26214
FREQU	37878.21 Hz
SCANS	256
ACQTM	0.6921 sec
PD	1.2000 sec
PW1	3.13 usec
IRNUC	1H
CTEMP	21.4 c
SLVNT	CDCL3
EXREF	77.00 ppm
BF	1.20 Hz
RGAIN	56

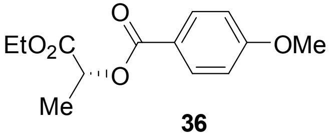

36

150418

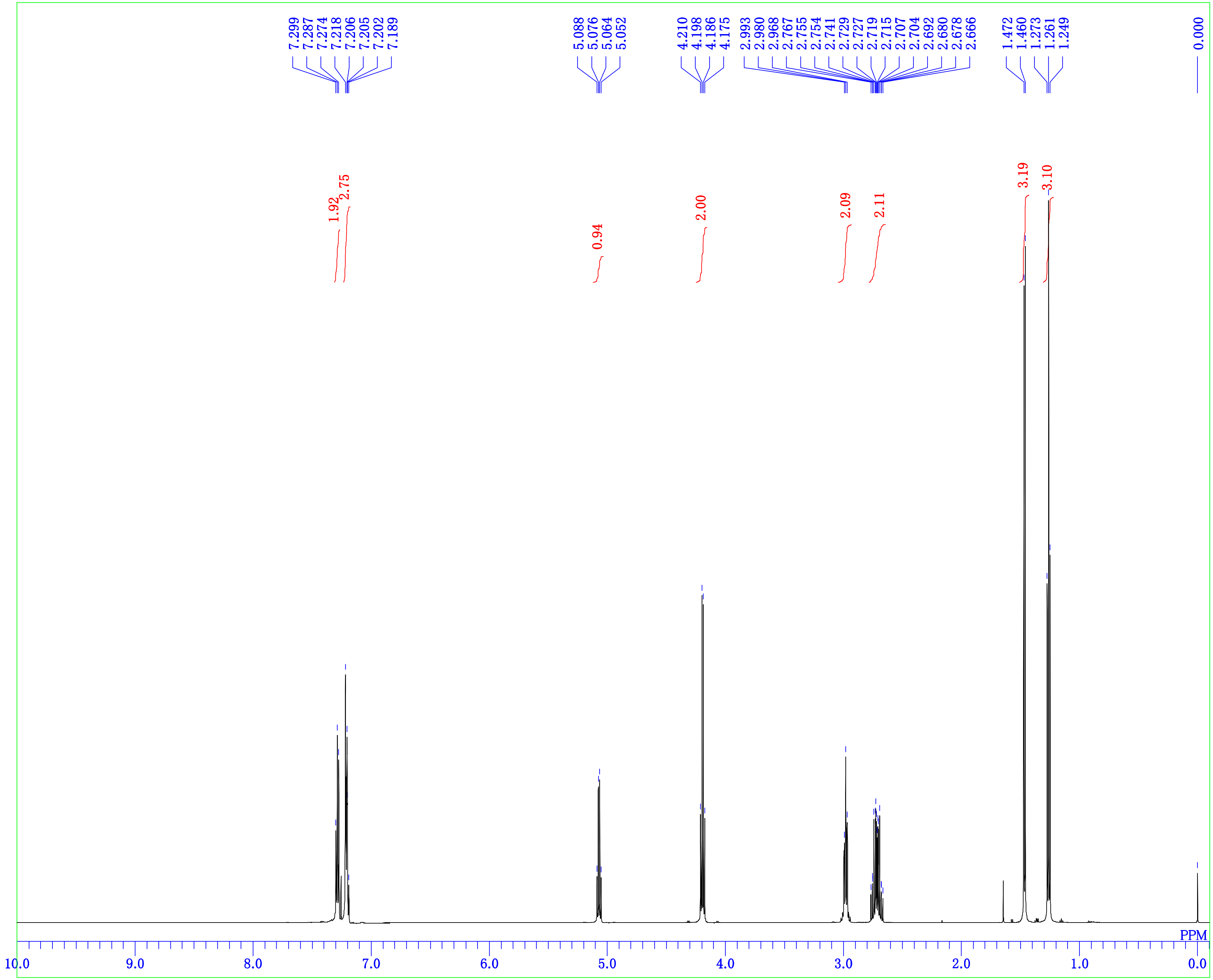

DFILE	d1533-gra-1h-1.als
COMNT	150418
DATIM	2015-04-18 15:47:07
OBNUC	1H
EXMOD	single_pulse.ex2
OBFRQ	600.17 MHz
OBSET	5.30 KHz
OBFIN	5.47 Hz
POINT	26214
FREQU	9008.87 Hz
SCANS	32
ACQTM	2.9098 sec
PD	2.0000 sec
PW1	7.30 usec
IRNUC	1H
CTEMP	21.0 c
SLVNT	CDCL3
EXREF	0.00 ppm
BF	0.12 Hz
RGAIN	30

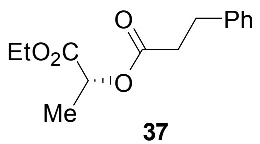

37

150418

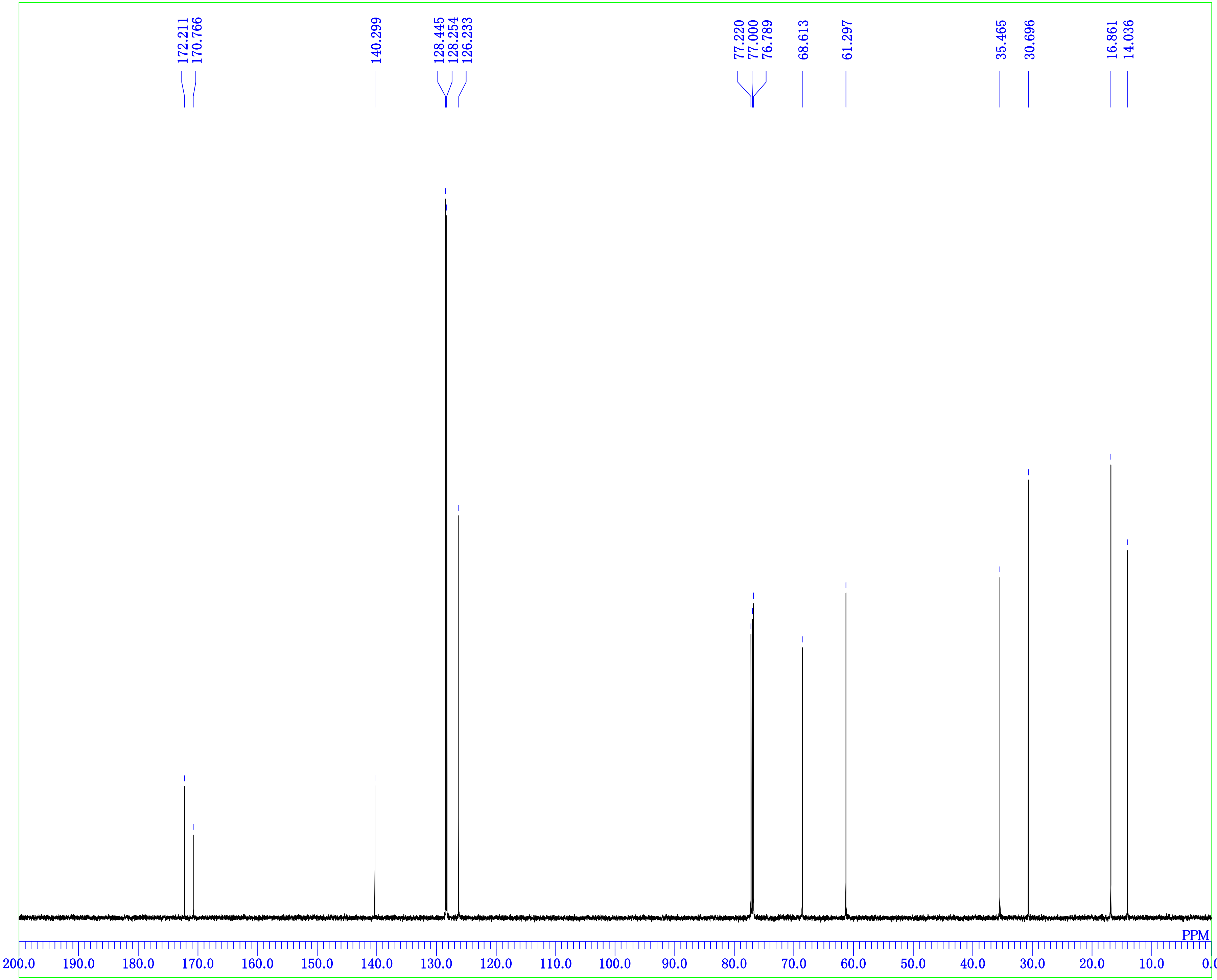

DFILE	d1533-gra-13c-1.als
COMNT	150418
DATIM	2015-04-18 15:56:05
OBNUC	13C
EXMOD	single_pulse_dec
OBFRQ	150.92 MHz
OBSET	8.52 KHz
OBFIN	1.74 Hz
POINT	26214
FREQU	37878.21 Hz
SCANS	256
ACQTM	0.6921 sec
PD	1.2000 sec
PW1	3.13 usec
IRNUC	1H
CTEMP	21.6 c
SLVNT	CDCL3
EXREF	77.00 ppm
BF	1.20 Hz
RGAIN	54

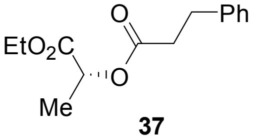

37

150324

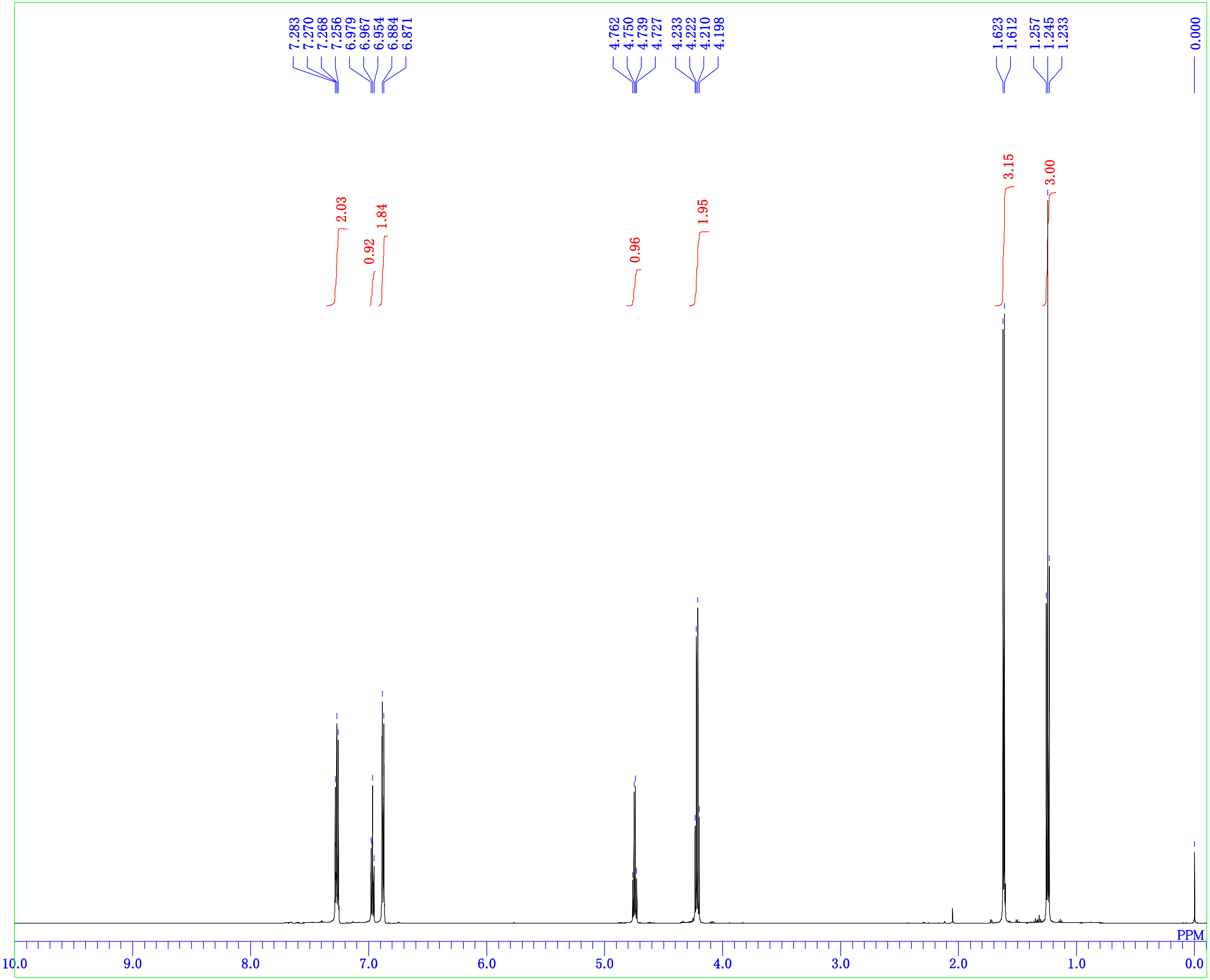

DFILE	d1515-gra-1h-1.als
COMNT	150324
DATIM	2015-03-24 20:53:03
OBNUC	1H
EXMOD	single_pulse.ex2
OBFRQ	600.17 MHz
OBSET	5.30 KHz
OBFIN	5.47 Hz
POINT	26214
FREQU	9008.87 Hz
SCANS	32
ACQTM	2.9098 sec
PD	2.0000 sec
PW1	7.30 usec
IRNUC	1H
CTEMP	20.3 c
SLVNT	CDCL3
EXREF	0.00 ppm
BF	0.12 Hz
RGAIN	38

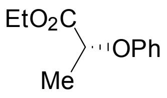

38

150324

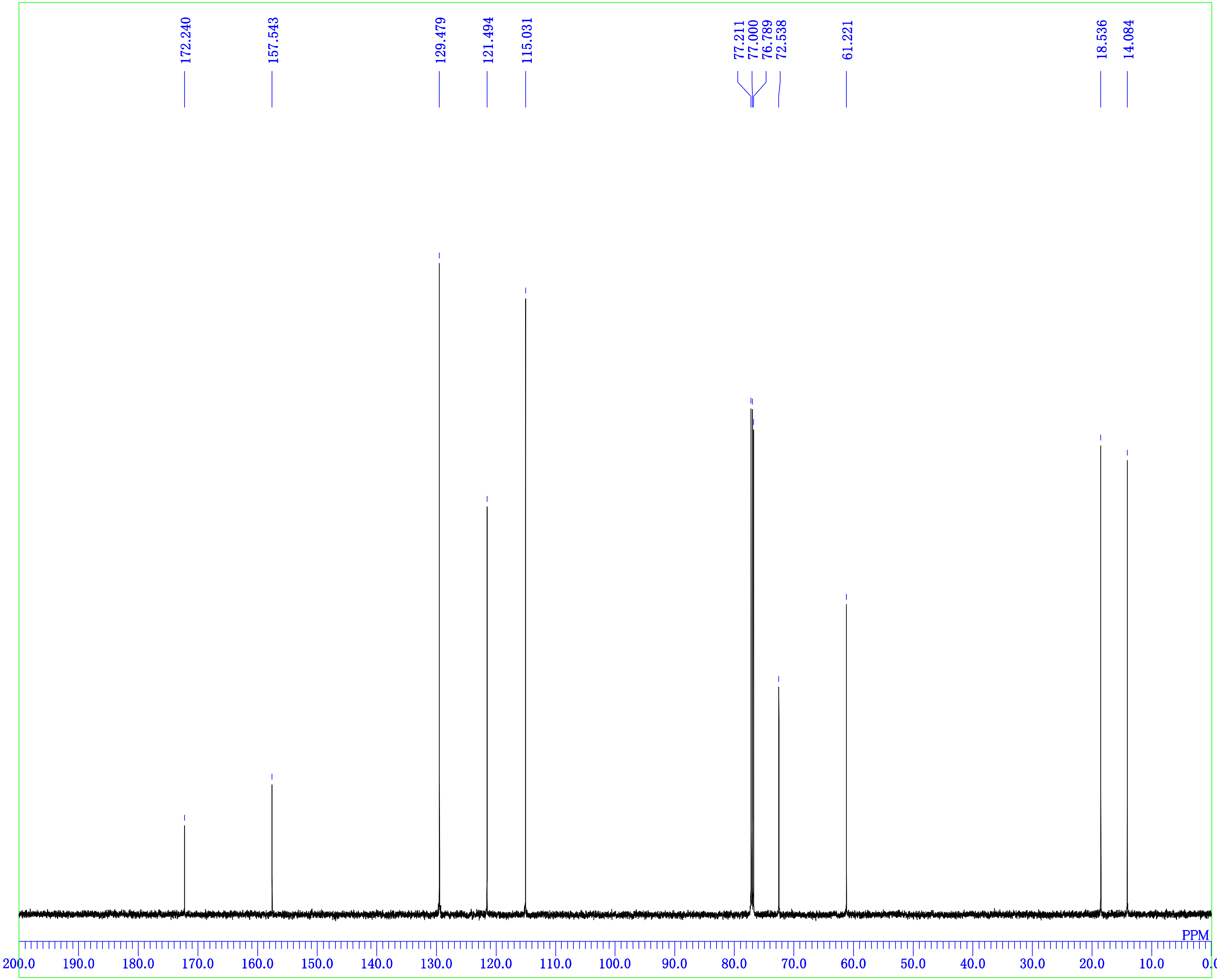

DFILE	d1515-gra-13c-1.als
COMNT	150324
DATIM	2015-03-24 21:01:43
OBNUC	13C
EXMOD	single_pulse_dec
OBFRQ	150.92 MHz
OBSET	8.52 KHz
OBFIN	1.74 Hz
POINT	26214
FREQU	37878.21 Hz
SCANS	256
ACQTM	0.6921 sec
PD	1.2000 sec
PW1	3.13 usec
IRNUC	1H
CTEMP	20.9 c
SLVNT	CDCL3
EXREF	77.00 ppm
BF	1.20 Hz
RGAIN	56

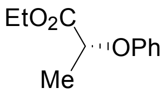

38

150701

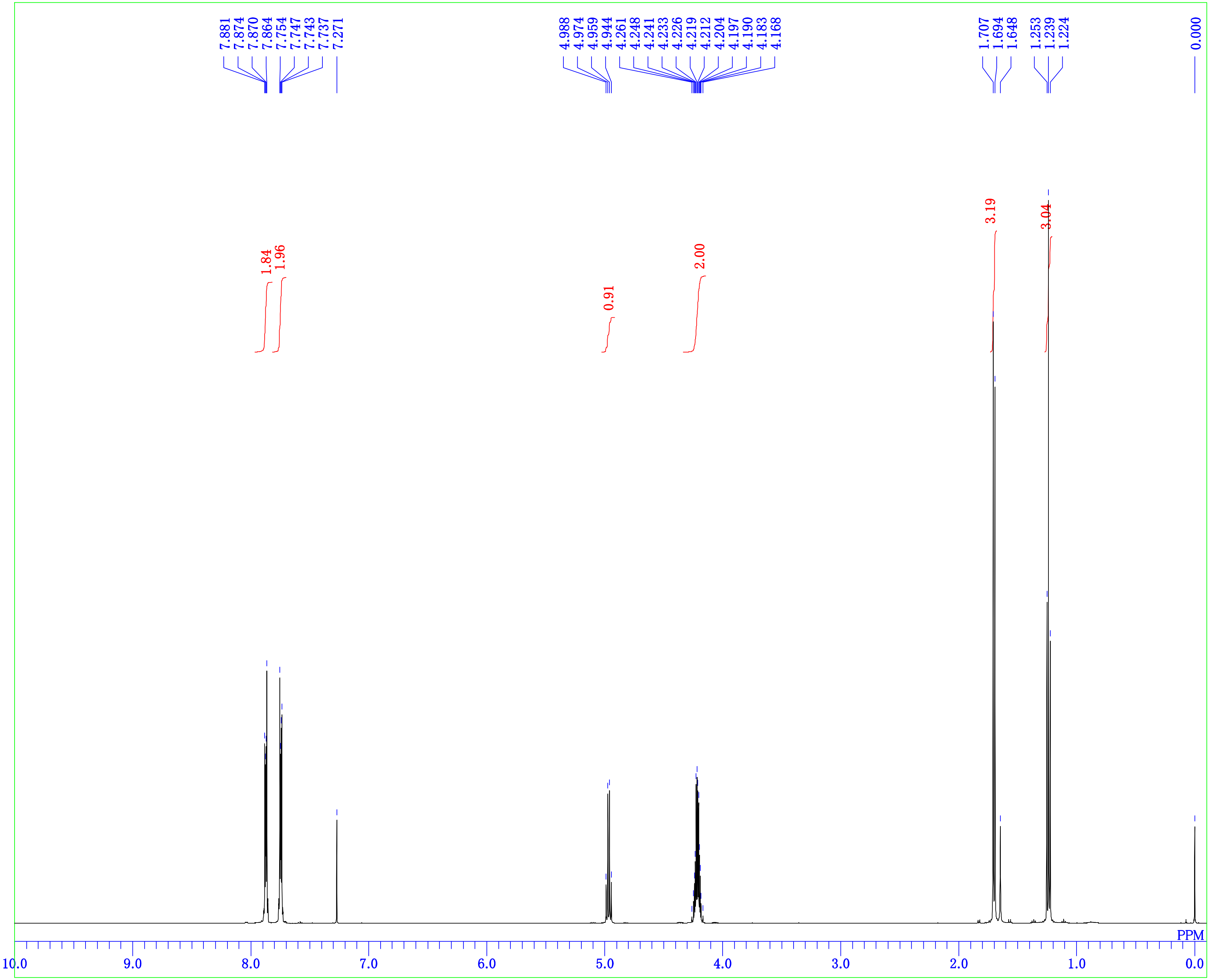

DFILE	d1503-gra-1h-1.als
COMNT	150701
DATIM	2015-07-01 21:12:31
OBNUC	1H
EXMOD	single_pulse.ex2
OBFRQ	500.16 MHz
OBSET	2.41 KHz
OBFIN	6.01 Hz
POINT	13107
FREQU	7507.39 Hz
SCANS	32
ACQTM	1.7459 sec
PD	2.0000 sec
PW1	5.80 usec
IRNUC	1H
CTEMP	20.2 c
SLVNT	CDCL3
EXREF	0.00 ppm
BF	0.12 Hz
RGAIN	42

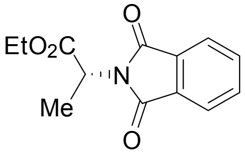

39

150701

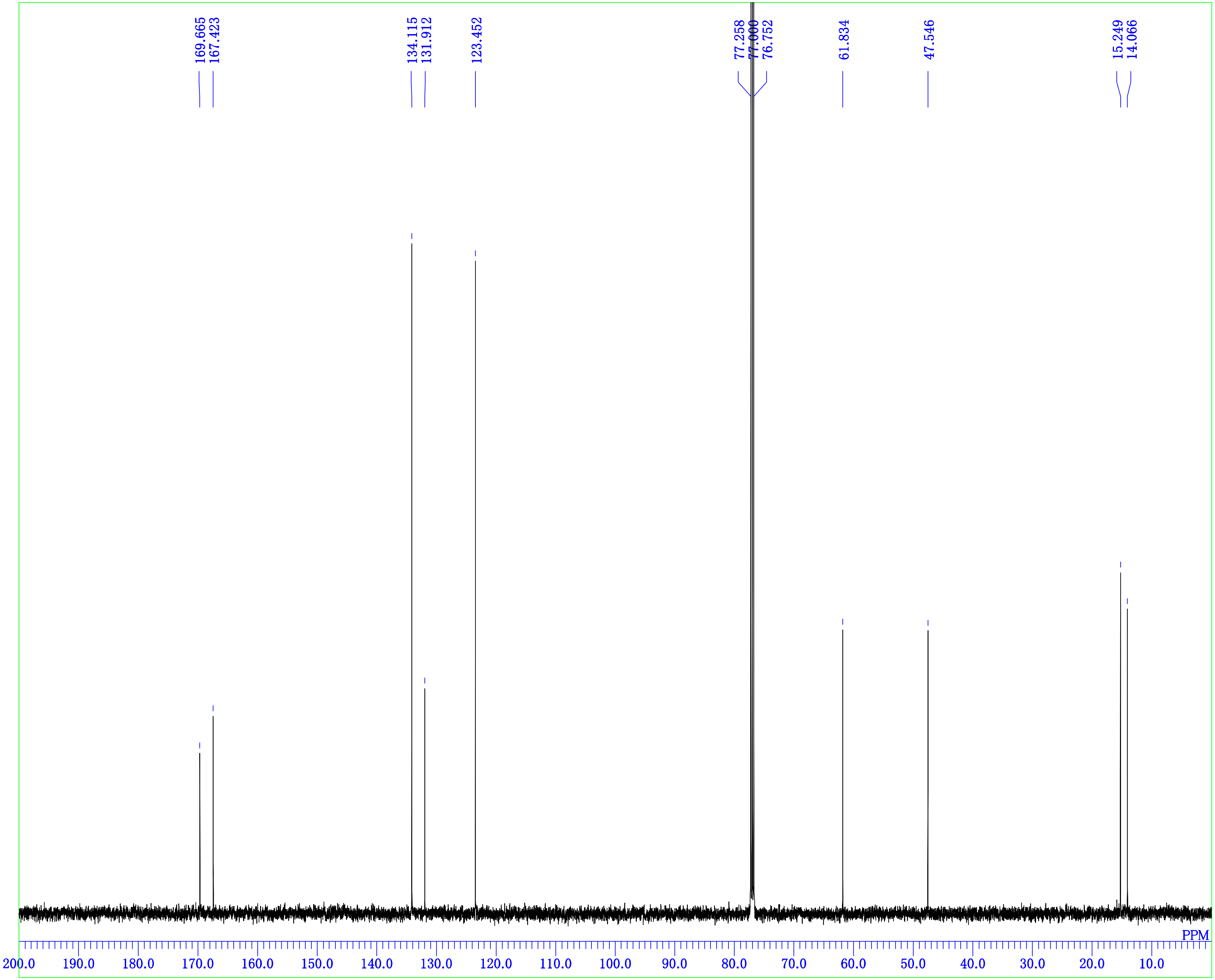

DFILE	d1503-gra-13c-1.als
COMNT	150701
DATIM	2015-07-01 21:25:17
OBNUC	13C
EXMOD	single_pulse_dec
OBFRQ	125.77 MHz
OBSET	7.87 KHz
OBFIN	4.21 Hz
POINT	26214
FREQU	31446.06 Hz
SCANS	256
ACQTM	0.8336 sec
PD	2.0000 sec
PW1	3.00 usec
IRNUC	1H
CTEMP	20.7 c
SLVNT	CDCL3
EXREF	77.00 ppm
BF	1.20 Hz
RGAIN	56

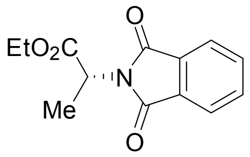

39

150601

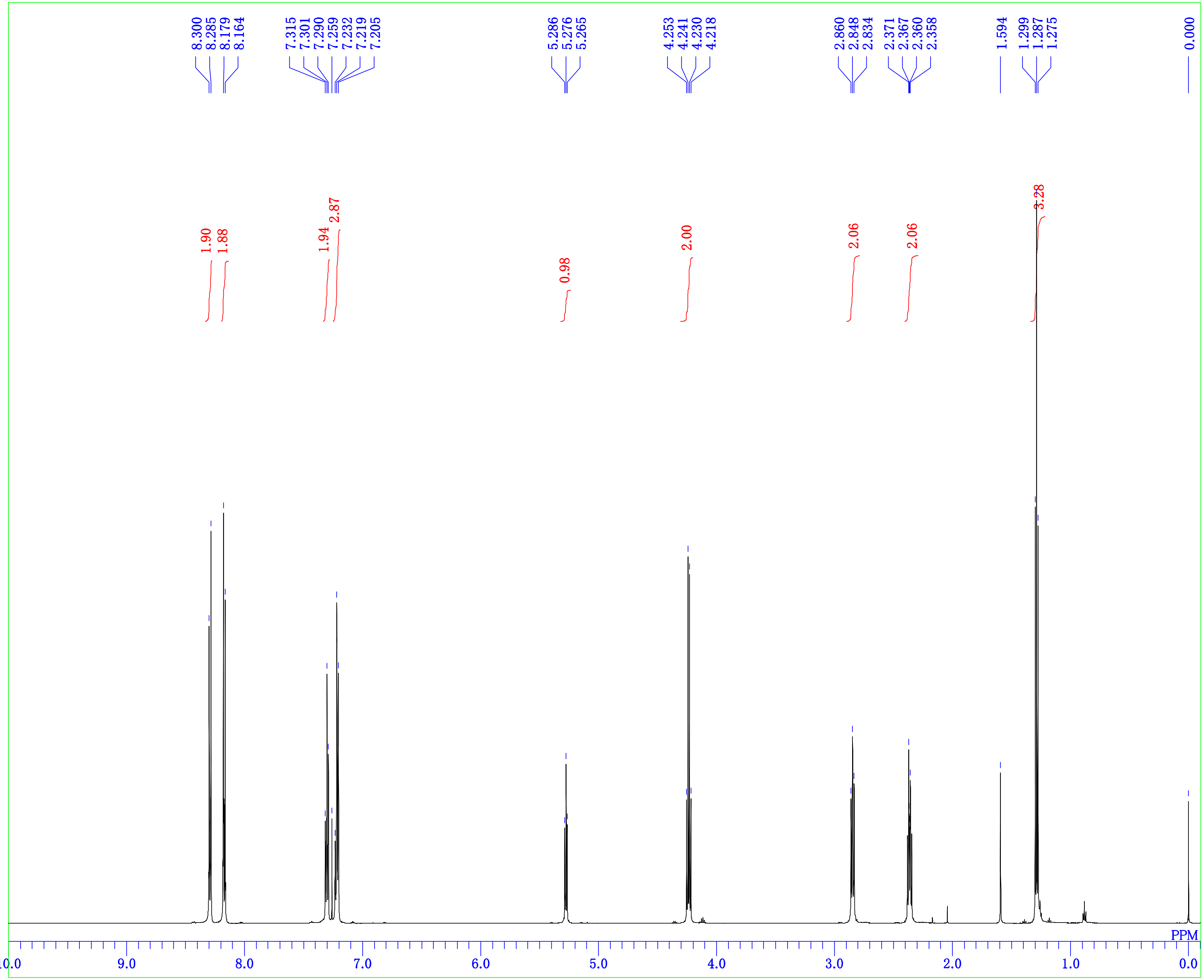

DFILE	d1608-gra-1h-1.als
COMNT	150601
DATIM	2015-06-01 11:09:43
OBNUC	1H
EXMOD	single_pulse.ex2
OBFRQ	600.17 MHz
OBSET	5.30 KHz
OBFIN	5.47 Hz
POINT	26214
FREQU	9008.87 Hz
SCANS	32
ACQTM	2.9098 sec
PD	2.0000 sec
PW1	7.30 usec
IRNUC	1H
CTEMP	22.5 c
SLVNT	CDCL3
EXREF	0.00 ppm
BF	0.12 Hz
RGAIN	38

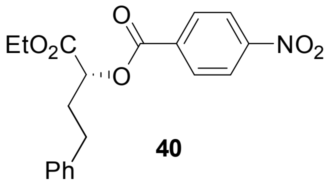

40

150601

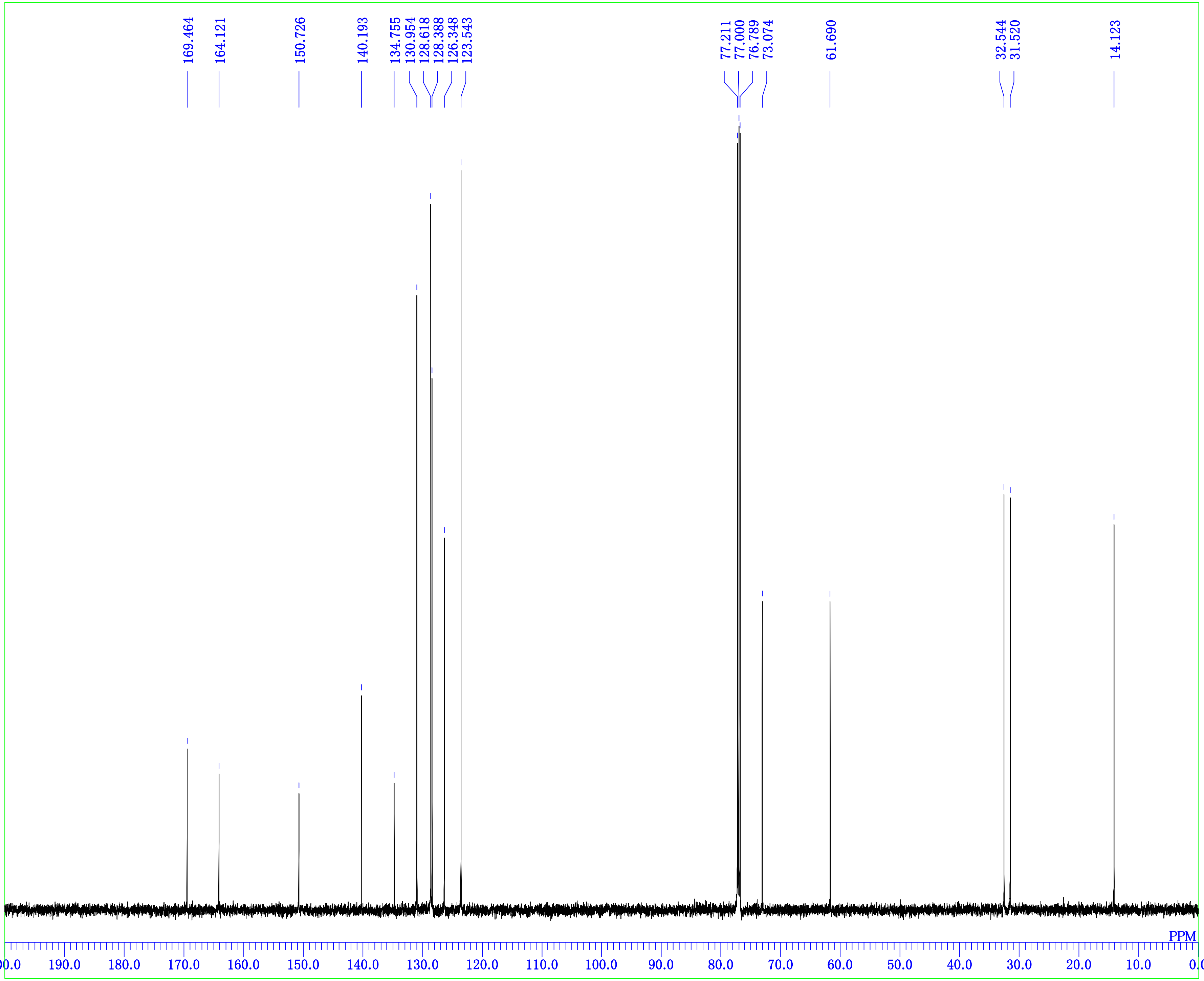

DFILE	d1608-gra-13c-1.als
COMNT	150601
DATIM	2015-06-01 11:18:24
OBNUC	13C
EXMOD	single_pulse_dec
OBFRQ	150.92 MHz
OBSET	8.52 KHz
OBFIN	1.74 Hz
POINT	26214
FREQU	37878.21 Hz
SCANS	256
ACQTM	0.6921 sec
PD	1.2000 sec
PW1	3.13 usec
IRNUC	1H
CTEMP	23.1 c
SLVNT	CDCL3
EXREF	77.00 ppm
BF	1.20 Hz
RGAIN	56

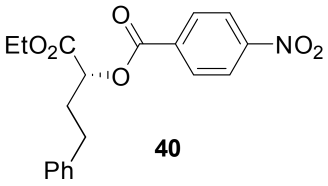

40

150304

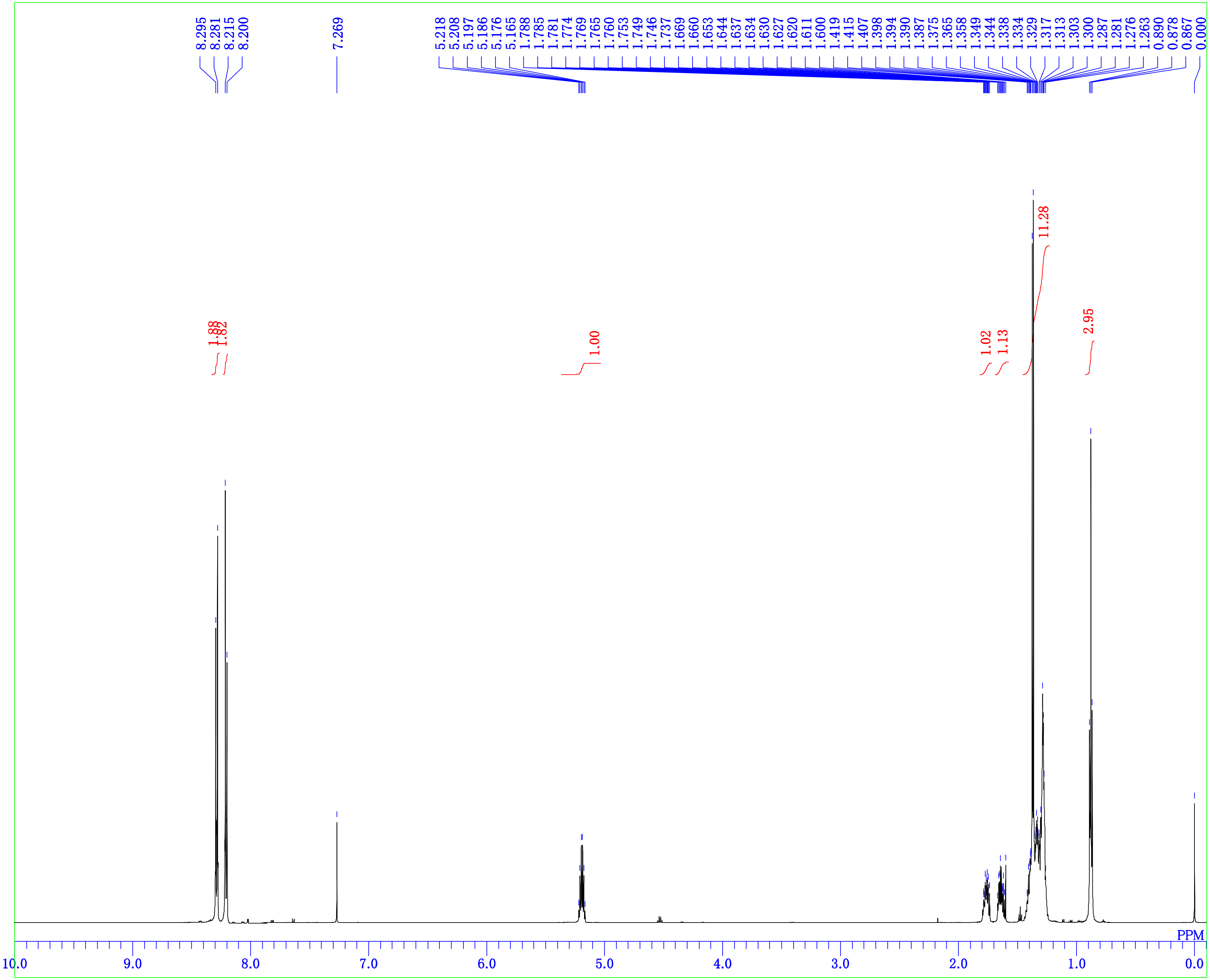

DFILE d1512-gra-1h-1.als
COMNT 150304
DATIM 2015-03-04 20:28:49
OBNUC 1H
EXMOD single_pulse.ex2
OBFRQ 600.17 MHz
OBSET 5.30 KHz
OBFIN 5.47 Hz
POINT 26214
FREQU 9008.87 Hz
SCANS 32
ACQTM 2.9098 sec
PD 2.0000 sec
PW1 5.85 usec
IRNUC 1H
CTEMP 20.3 c
SLVNT CDCL3
EXREF 0.00 ppm
BF 0.12 Hz
RGAIN 36

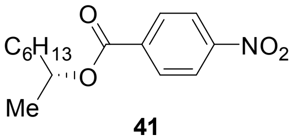

41

150304

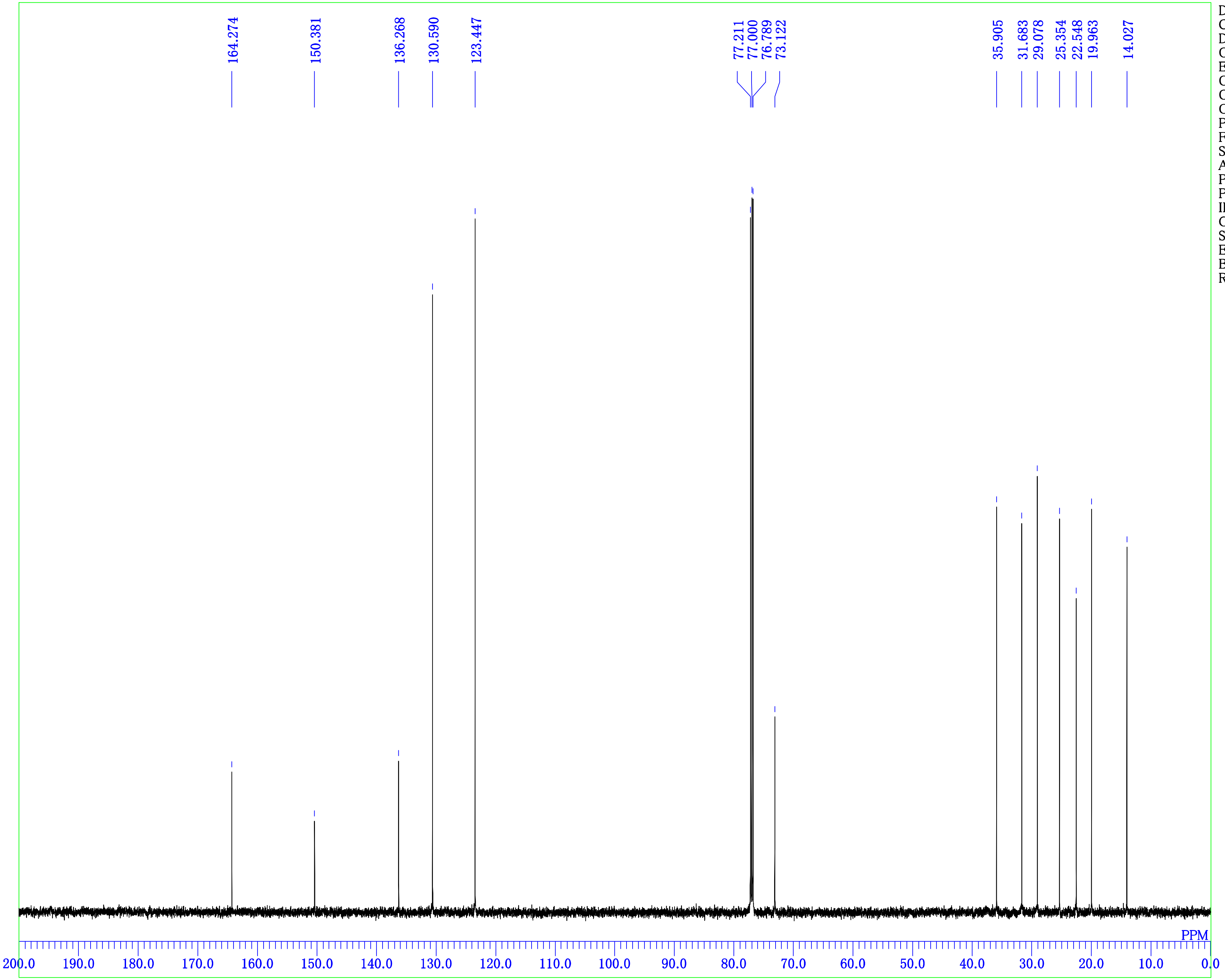

DFILE	d1512-gra-13c-1.als
COMNT	150304
DATIM	2015-03-04 20:38:37
OBNUC	13C
EXMOD	single_pulse_dec
OBFRQ	150.92 MHz
OBSET	8.52 KHz
OBFIN	1.74 Hz
POINT	26214
FREQU	37878.21 Hz
SCANS	256
ACQTM	0.6921 sec
PD	1.2000 sec
PW1	2.97 usec
IRNUC	1H
CTEMP	20.6 c
SLVNT	CDCL3
EXREF	77.00 ppm
BF	1.20 Hz
RGAIN	56

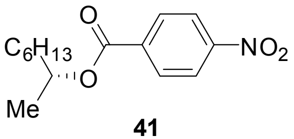

41

150302

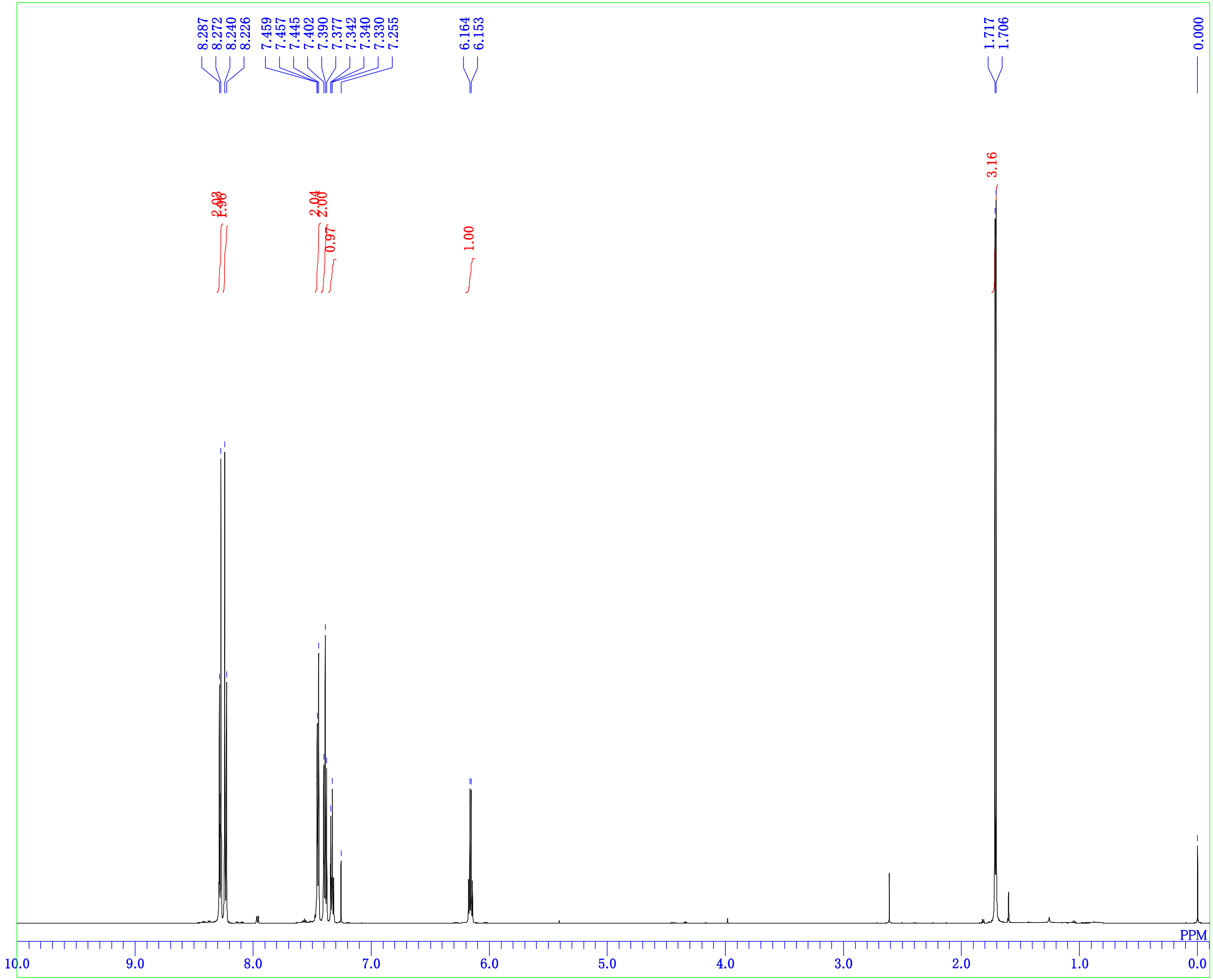

DFILE d1510-gra-1h-1.als
COMNT 150302
DATIM 2015-03-02 16:34:50
OBNUC 1H
EXMOD single_pulse.ex2
OBFRQ 600.17 MHz
OBSET 5.30 KHz
OBFIN 5.47 Hz
POINT 26214
FREQU 9008.87 Hz
SCANS 32
ACQTM 2.9098 sec
PD 2.0000 sec
PW1 5.85 usec
IRNUC 1H
CTEMP 20.7 c
SLVNT CDCL3
EXREF 0.00 ppm
BF 0.12 Hz
RGAIN 36

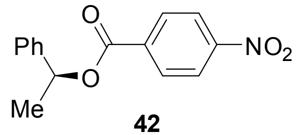

42

150302

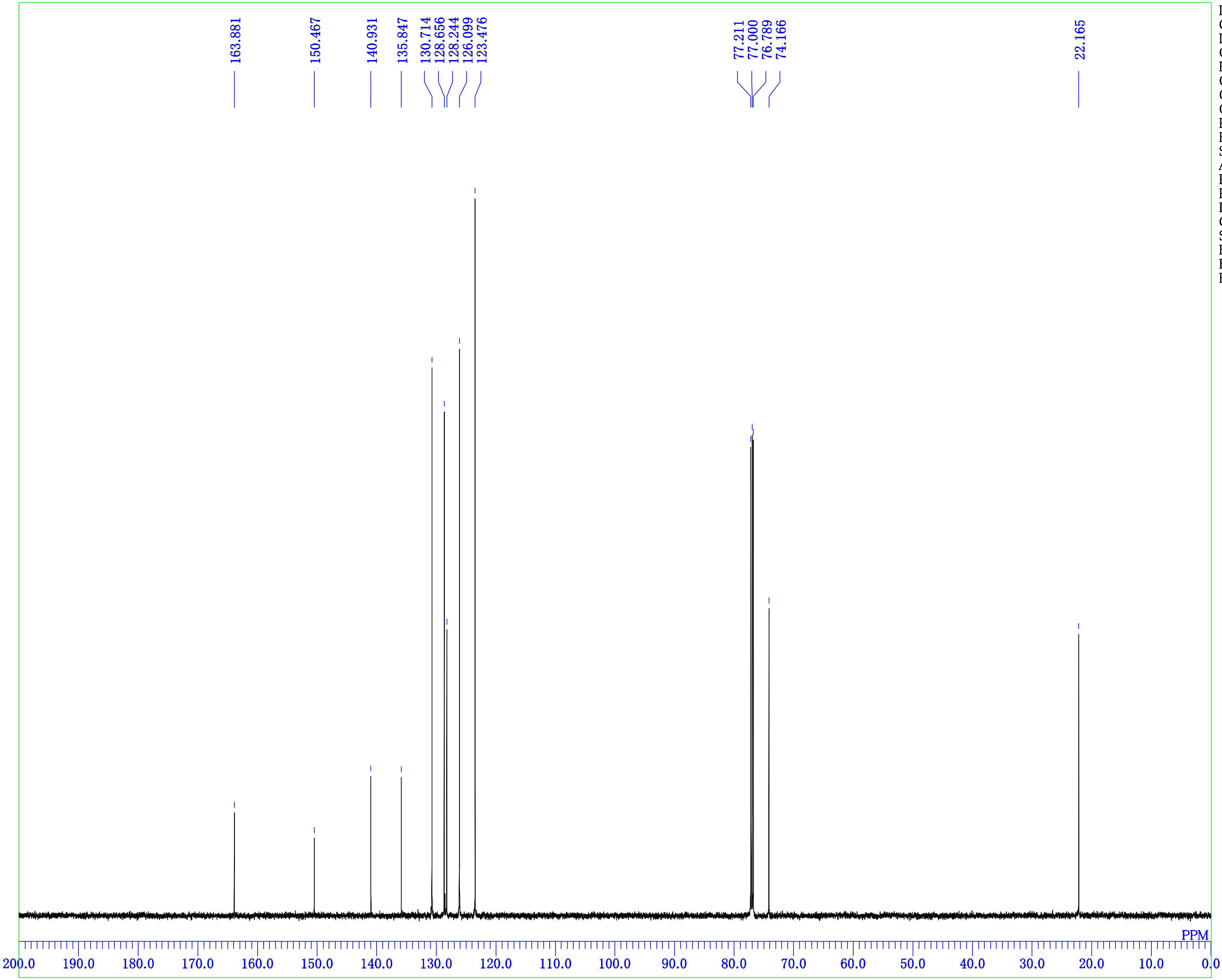

DFILE	d1510-gra-13c-1.als
COMNT	150302
DATIM	2015-03-02 16:43:25
OBNUC	13C
EXMOD	single_pulse_dec
OBFRQ	150.92 MHz
OBSET	8.52 KHz
OBFIN	1.74 Hz
POINT	26214
FREQU	37878.21 Hz
SCANS	256
ACQTM	0.6921 sec
PD	1.2000 sec
PW1	2.97 usec
IRNUC	1H
CTEMP	21.3 c
SLVNT	CDCL3
EXREF	77.00 ppm
BF	1.20 Hz
RGAIN	58

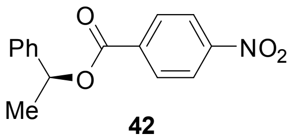

42

150421

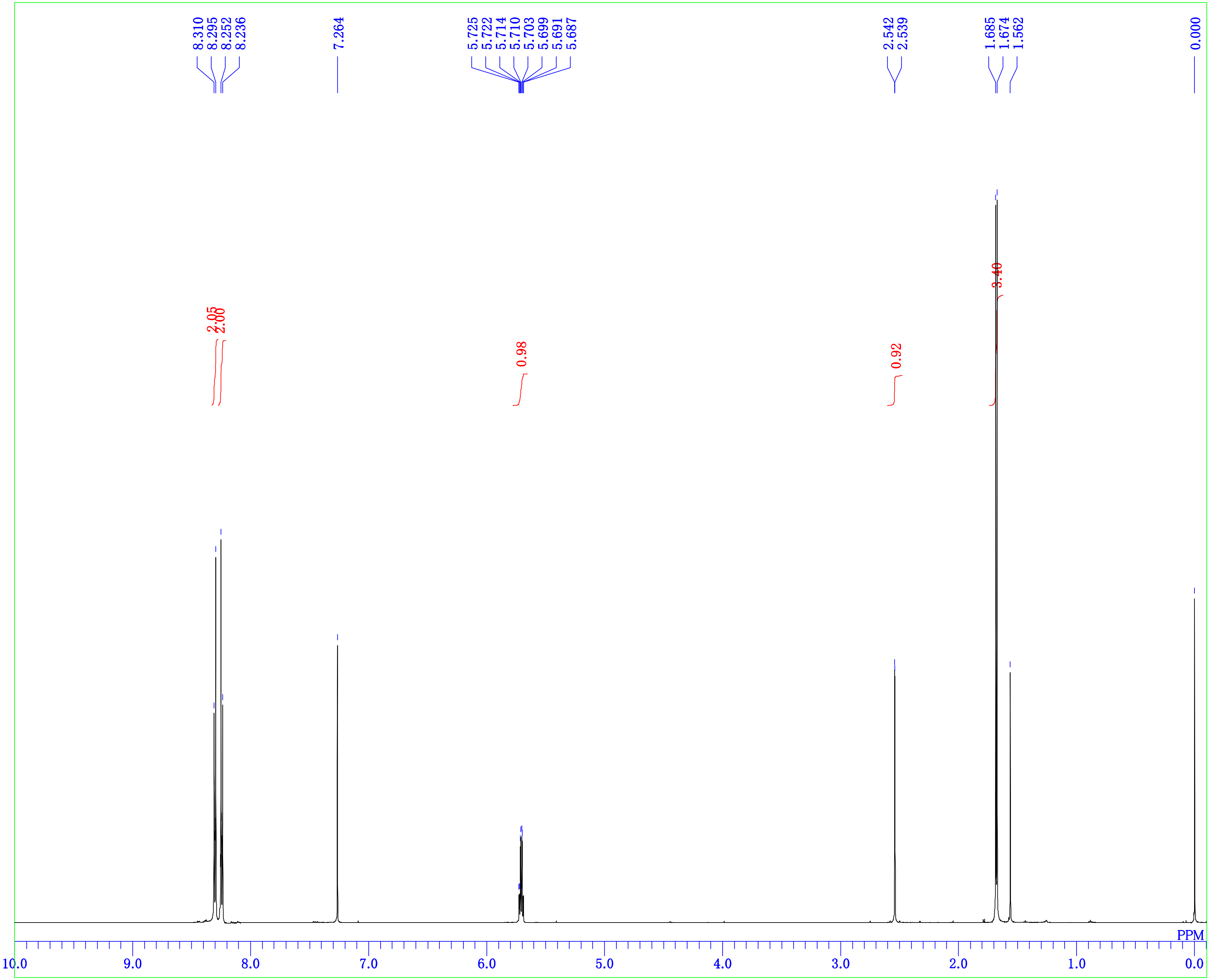

DFILE	d1530-gra-1h-1.als
COMNT	150421
DATIM	2015-04-21 16:42:57
OBNUC	1H
EXMOD	single_pulse.ex2
OBFRQ	600.17 MHz
OBSET	5.30 KHz
OBFIN	5.47 Hz
POINT	26214
FREQU	9008.87 Hz
SCANS	32
ACQTM	2.9098 sec
PD	2.0000 sec
PW1	7.30 usec
IRNUC	1H
CTEMP	21.2 c
SLVNT	CDCL3
EXREF	0.00 ppm
BF	0.12 Hz
RGAIN	50

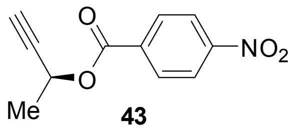

43

150421

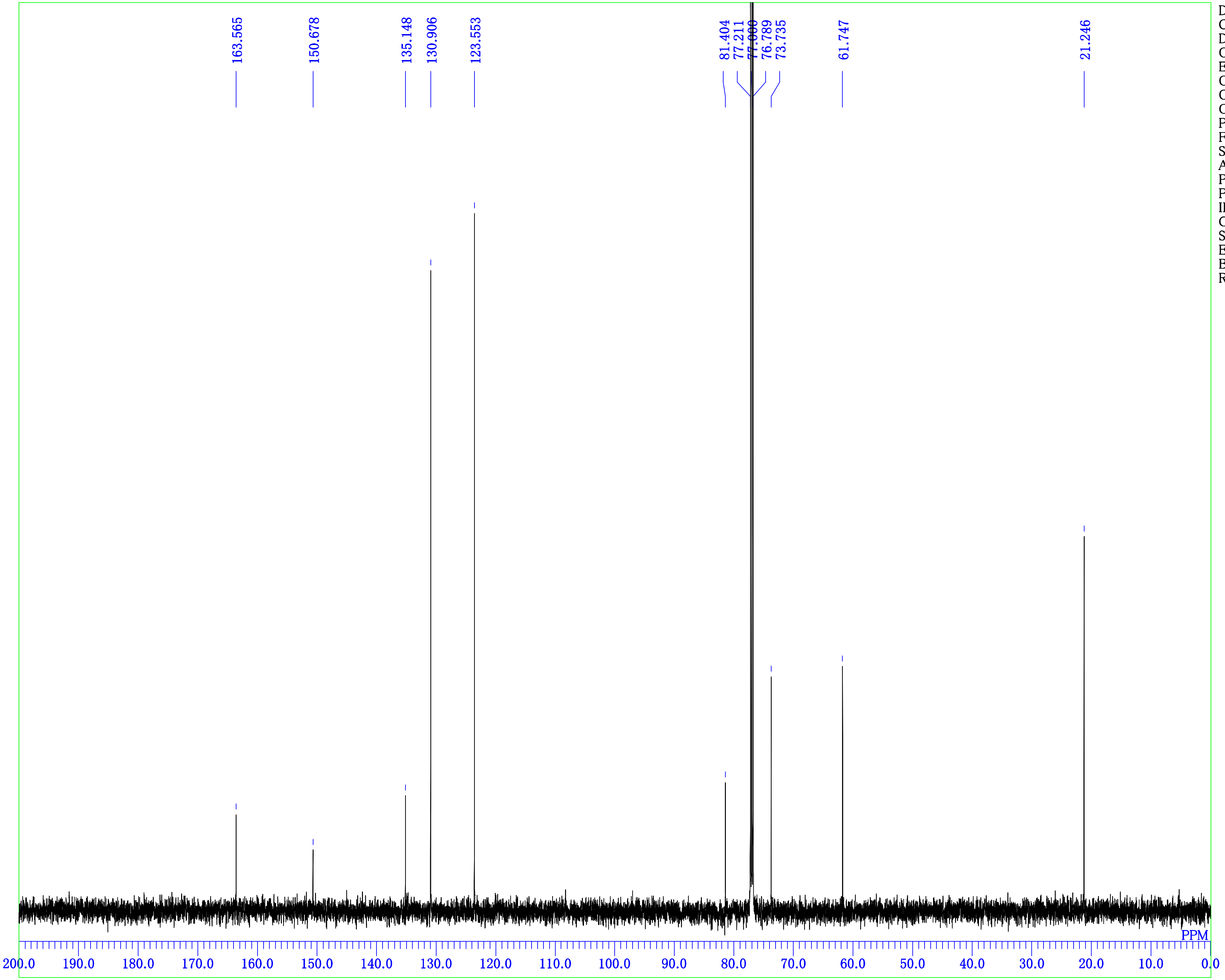

DFILE	d1530-gra-13c-1.als
COMNT	150421
DATIM	2015-04-21 16:54:40
OBNUC	13C
EXMOD	single_pulse_dec
OBFRQ	150.92 MHz
OBSET	8.52 KHz
OBFIN	1.74 Hz
POINT	26214
FREQU	37878.21 Hz
SCANS	256
ACQTM	0.6921 sec
PD	1.2000 sec
PW1	3.13 usec
IRNUC	1H
CTEMP	21.7 c
SLVNT	CDCL3
EXREF	77.00 ppm
BF	1.20 Hz
RGAIN	54

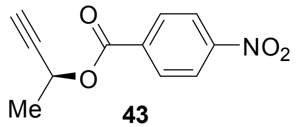

43

150429

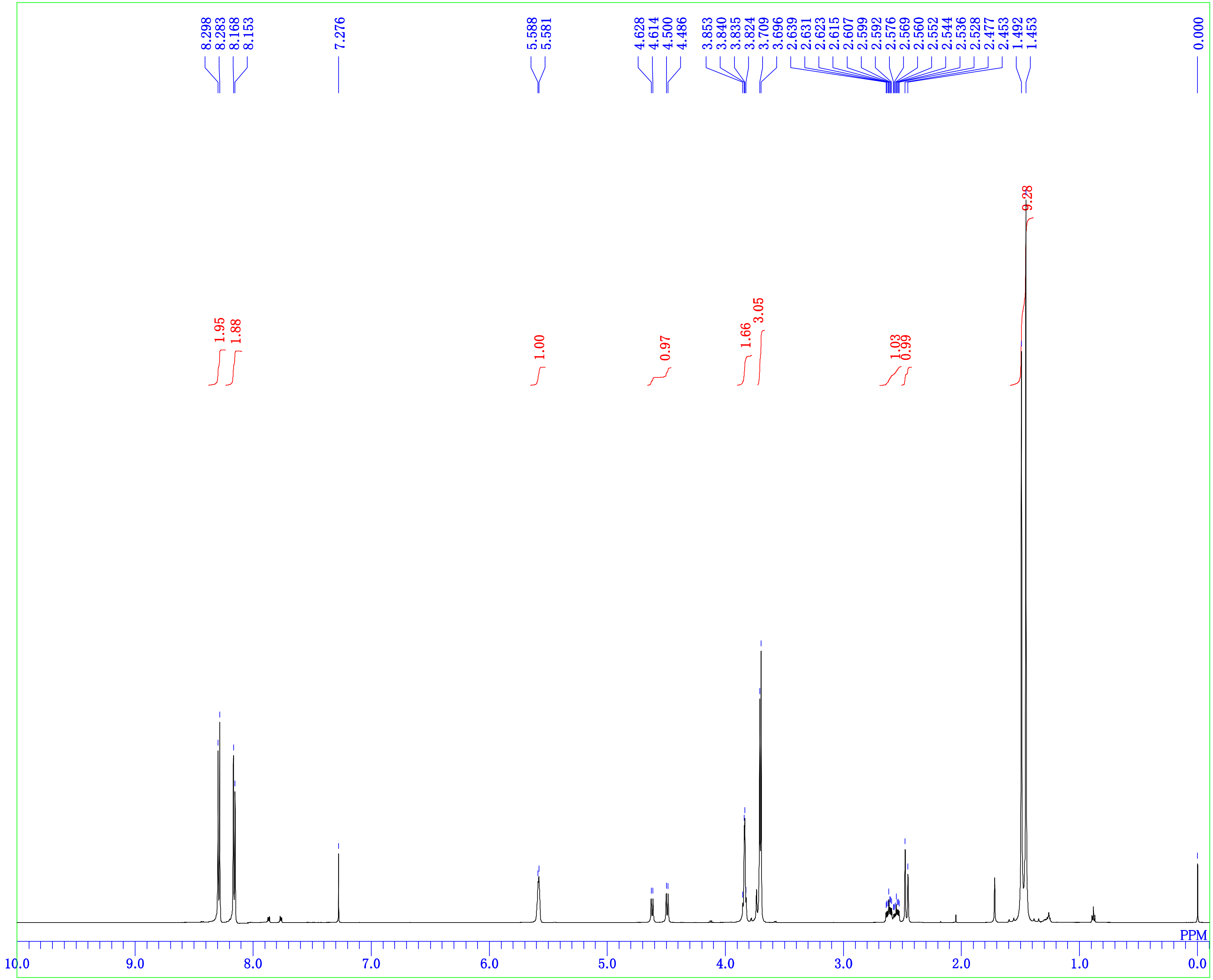

DFILE	d1561-gra-1h-1.als
COMNT	150429
DATIM	2015-04-29 18:12:44
OBNUC	1H
EXMOD	single_pulse.ex2
OBFRQ	600.17 MHz
OBSET	5.30 KHz
OBFIN	5.47 Hz
POINT	26214
FREQU	9008.87 Hz
SCANS	32
ACQTM	2.9098 sec
PD	2.0000 sec
PW1	7.30 usec
IRNUC	1H
CTEMP	21.8 c
SLVNT	CDCL3
EXREF	0.00 ppm
BF	0.12 Hz
RGAIN	38

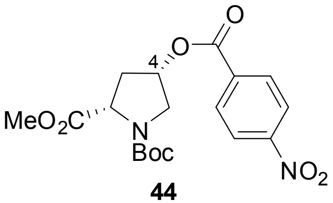

44

150429

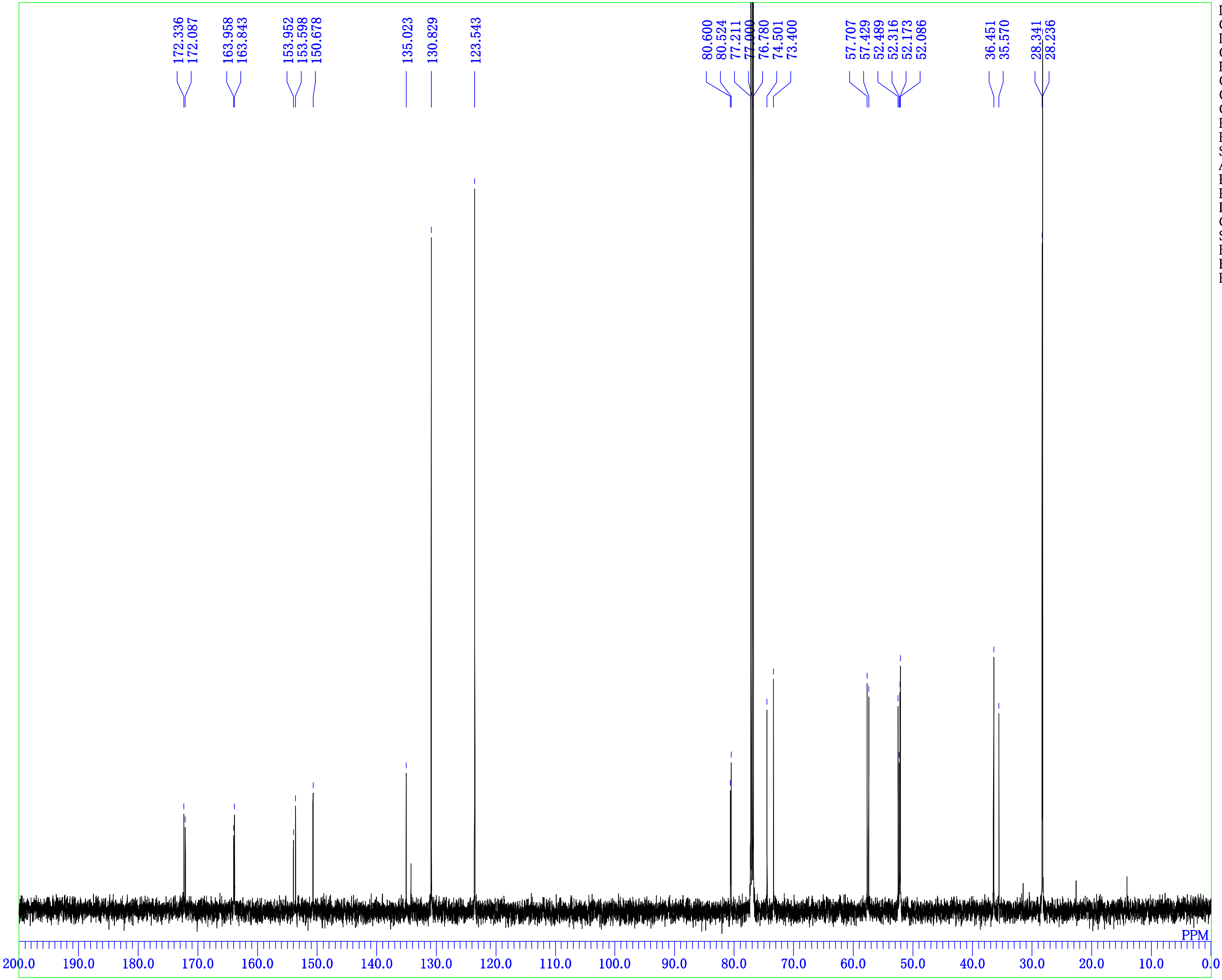

DFILE	d1561-gra-13c-1.als
COMNT	150429
DATIM	2015-04-29 18:22:09
OBNUC	13C
EXMOD	single_pulse_dec
OBFRQ	150.92 MHz
OBSET	8.52 KHz
OBFIN	1.74 Hz
POINT	26214
FREQU	37878.21 Hz
SCANS	256
ACQTM	0.6921 sec
PD	1.2000 sec
PW1	3.13 usec
IRNUC	1H
CTEMP	22.4 c
SLVNT	CDCL3
EXREF	77.00 ppm
BF	1.20 Hz
RGAIN	56

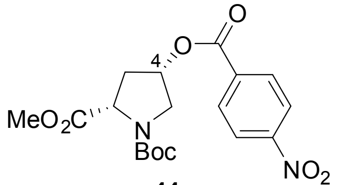

44

150418

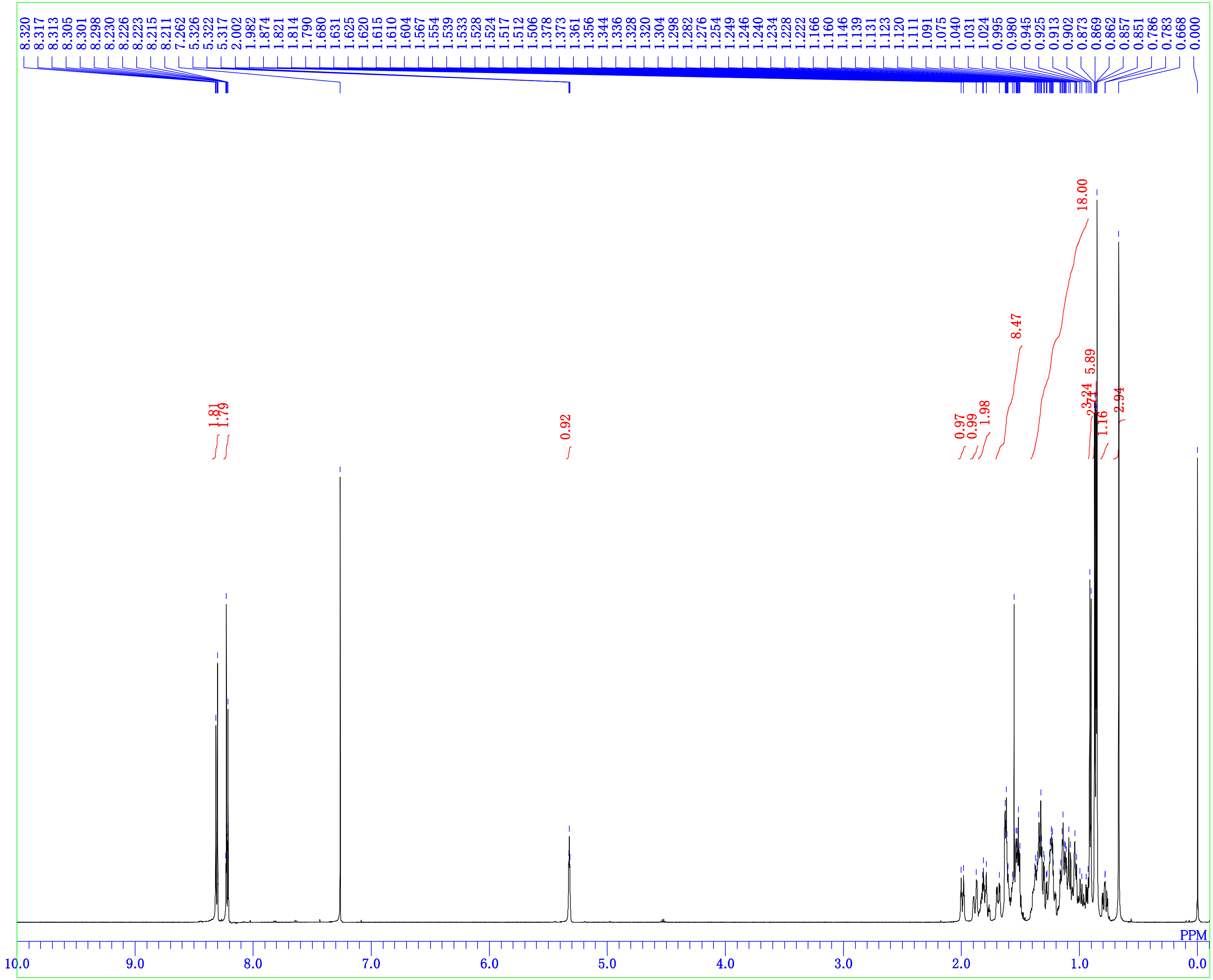

DFILE d1534-gra-1h-1.als
COMNT 150418
DATIM 2015-04-18 16:04:57
OBNUC 1H
EXMOD single_pulse.ex2
OBFRQ 600.17 MHz
OBSET 5.30 KHz
OBFIN 5.47 Hz
POINT 26214
FREQU 9008.87 Hz
SCANS 32
ACQTM 2.9098 sec
PD 2.0000 sec
PW1 7.30 usec
IRNUC 1H
CTEMP 21.1 c
SLVNT CDCL3
EXREF 0.00 ppm
BF 0.12 Hz
RGAIN 40

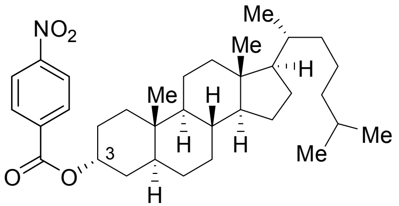

45

150418

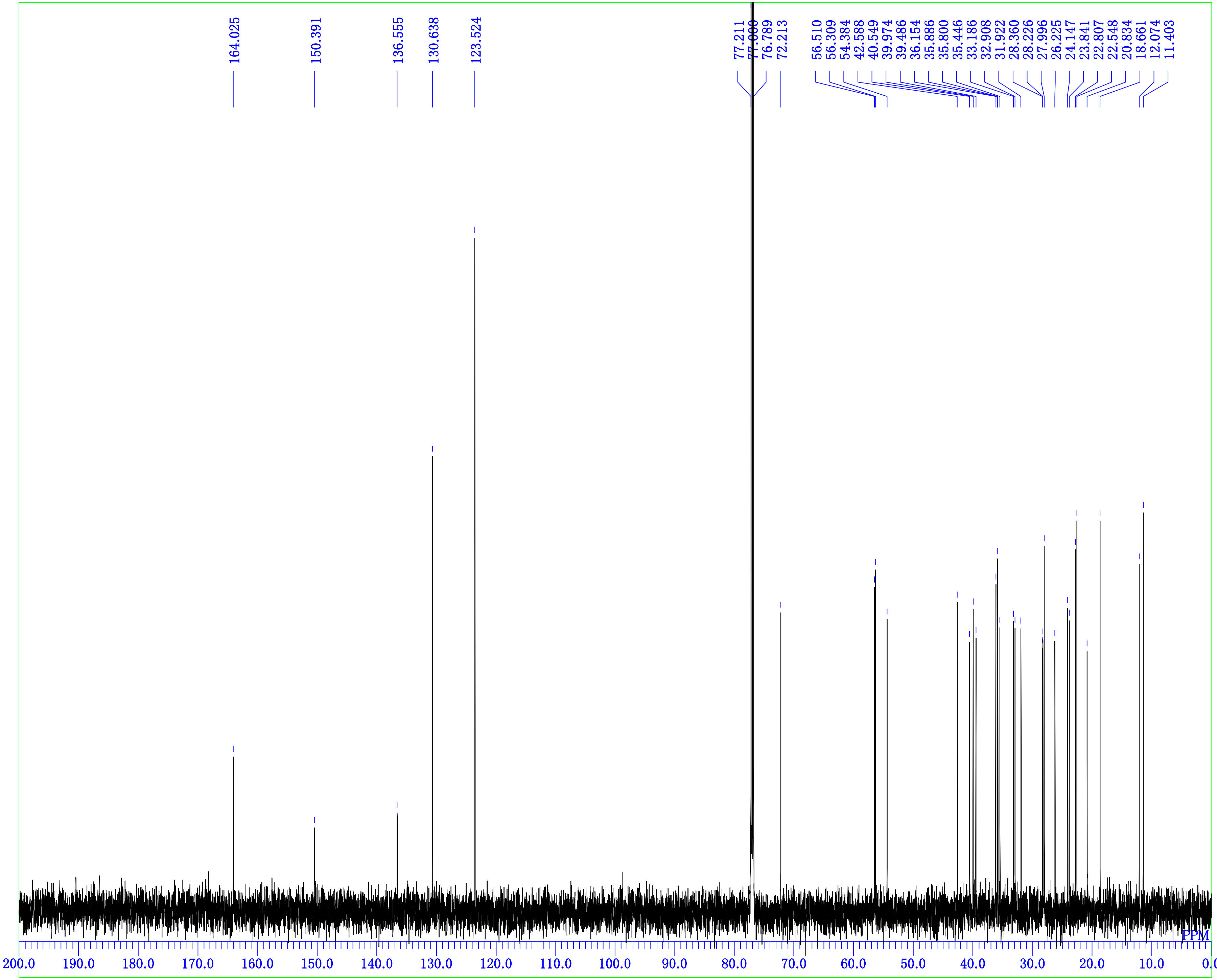

DFILE	d1534-gra-13c-1.als
COMNT	150418
DATIM	2015-04-18 16:13:31
OBNUC	13C
EXMOD	single_pulse_dec
OBFRQ	150.92 MHz
OBSET	8.52 KHz
OBFIN	1.74 Hz
POINT	26214
FREQU	37878.21 Hz
SCANS	256
ACQTM	0.6921 sec
PD	1.2000 sec
PW1	3.13 usec
IRNUC	1H
CTEMP	21.7 c
SLVNT	CDCL3
EXREF	77.00 ppm
BF	1.20 Hz
RGAIN	54

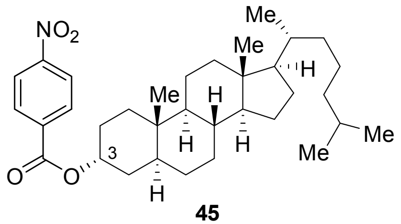

45

150304

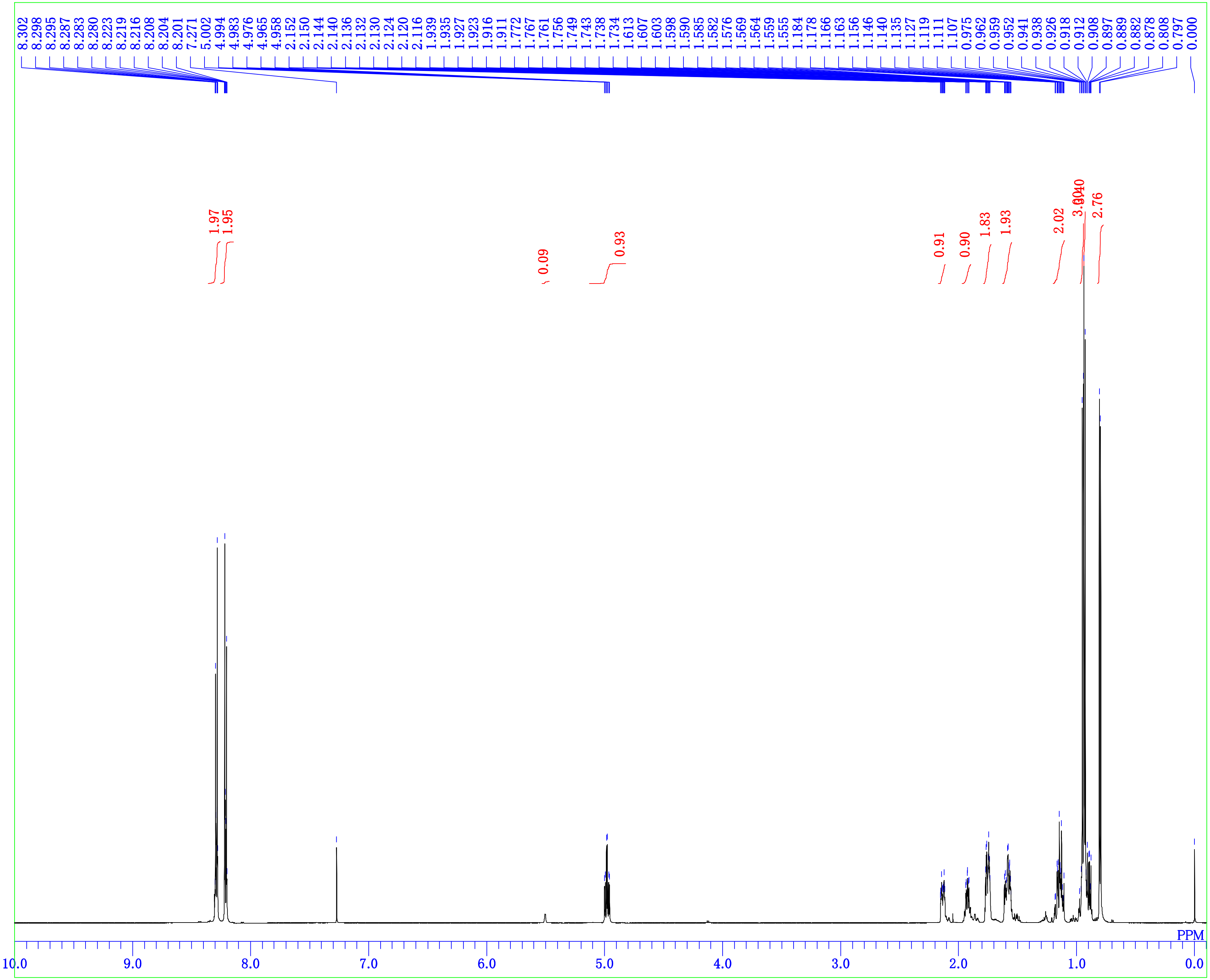

DFILE	d1511-gra-1h-1.als
COMNT	150304
DATIM	2015-03-04 20:47:01
OBNUC	1H
EXMOD	single_pulse.ex2
OBFRQ	600.17 MHz
OBSET	5.30 KHz
OBFIN	5.47 Hz
POINT	26214
FREQU	9008.87 Hz
SCANS	32
ACQTM	2.9098 sec
PD	2.0000 sec
PW1	5.85 usec
IRNUC	1H
CTEMP	20.1 c
SLVNT	CDCL3
EXREF	0.00 ppm
BF	0.12 Hz
RGAIN	32

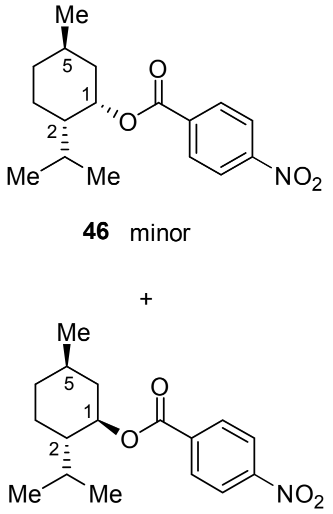

150304

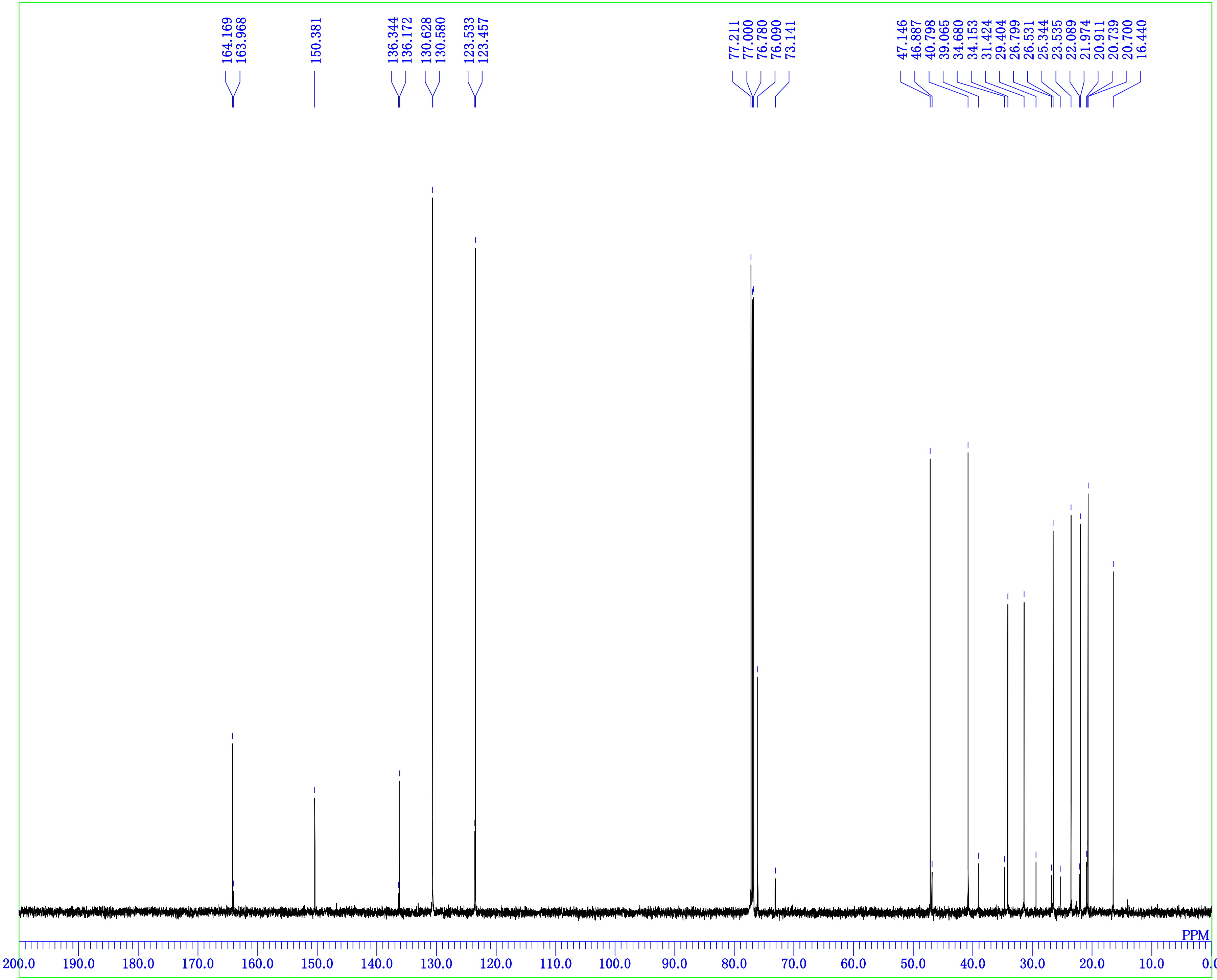

DFILE	d1511-gra-13c-1.als
COMNT	150304
DATIM	2015-03-04 20:55:56
OBNUC	13C
EXMOD	single_pulse_dec
OBFRQ	150.92 MHz
OBSET	8.52 KHz
OBFIN	1.74 Hz
POINT	26214
FREQU	37878.21 Hz
SCANS	256
ACQTM	0.6921 sec
PD	1.2000 sec
PW1	2.97 usec
IRNUC	1H
CTEMP	20.5 c
SLVNT	CDCL3
EXREF	77.00 ppm
BF	1.20 Hz
RGAIN	56

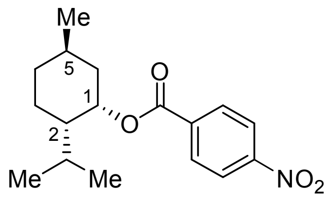

46 minor

+

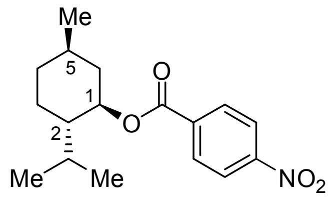

major

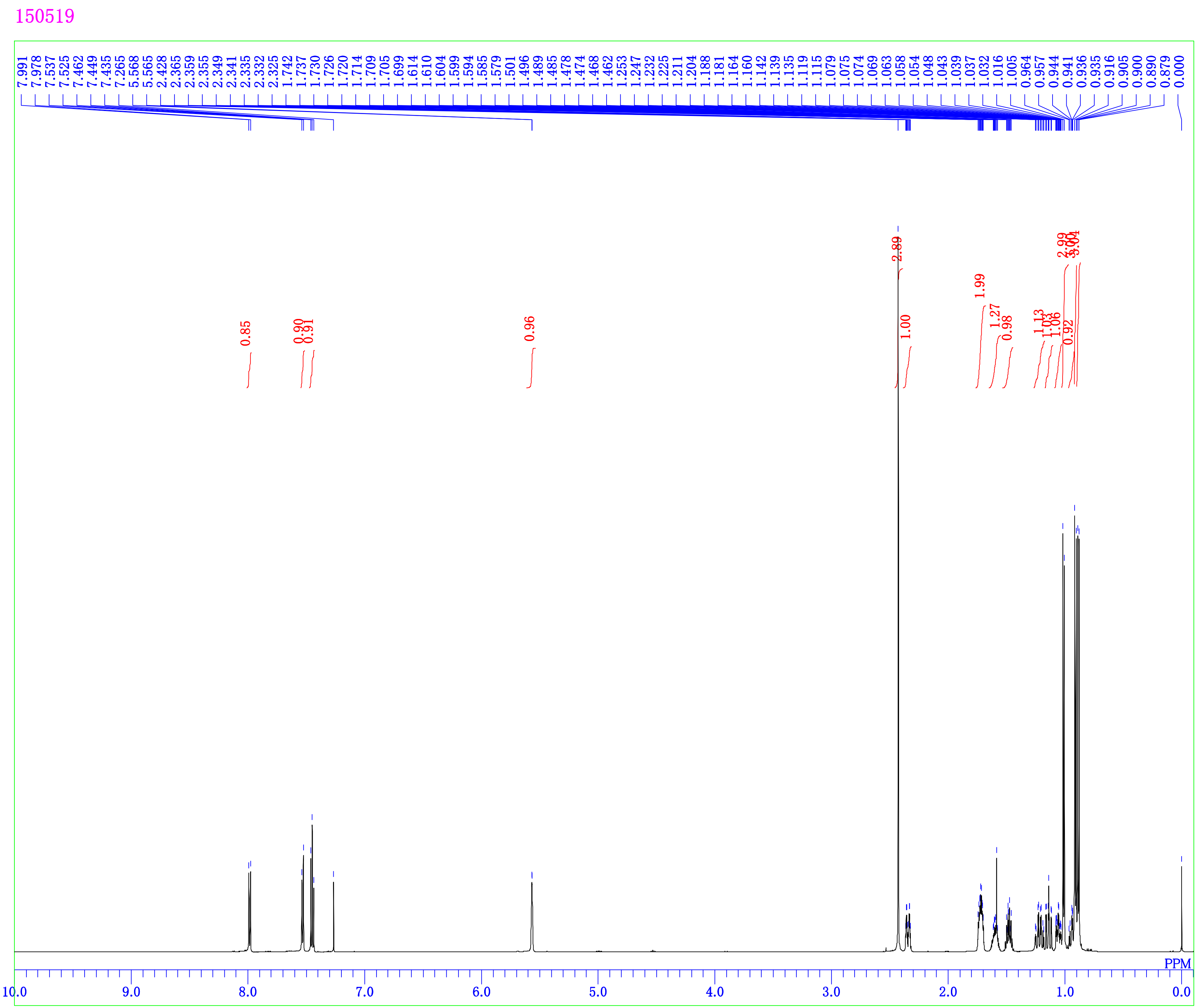

DFILE	d1580-gra-1h-1.als
COMNT	150519
DATIM	2015-05-19 18:27:43
OBNUC	1H
EXMOD	single_pulse.ex2
OBFRQ	600.17 MHz
OBSET	5.30 KHz
OBFIN	5.47 Hz
POINT	26214
FREQU	9008.87 Hz
SCANS	32
ACQTM	2.9098 sec
PD	2.0000 sec
PW1	7.30 usec
IRNUC	1H
CTEMP	21.4 c
SLVNT	CDCL3
EXREF	0.00 ppm
BF	0.12 Hz
RGAIN	34

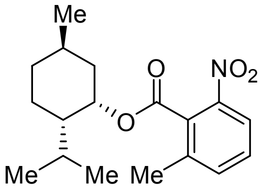

47

150519

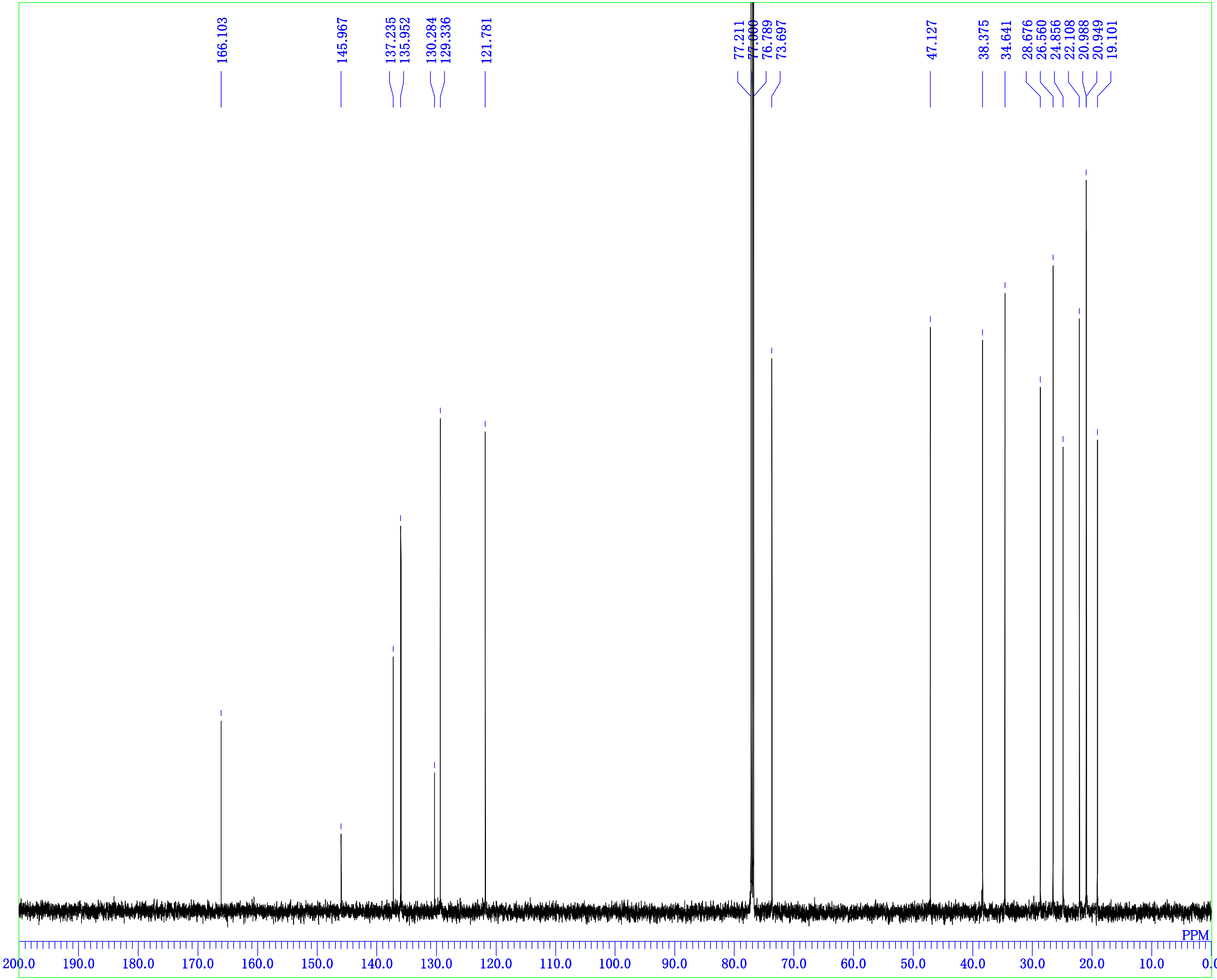

DFILE	d1580-gra-13c-1.als
COMNT	150519
DATIM	2015-05-19 18:36:26
OBNUC	13C
EXMOD	single_pulse_dec
OBFRQ	150.92 MHz
OBSET	8.52 KHz
OBFIN	1.74 Hz
POINT	26214
FREQU	37878.21 Hz
SCANS	256
ACQTM	0.6921 sec
PD	1.2000 sec
PW1	3.13 usec
IRNUC	1H
CTEMP	22.0 c
SLVNT	CDCL3
EXREF	77.00 ppm
BF	1.20 Hz
RGAIN	54

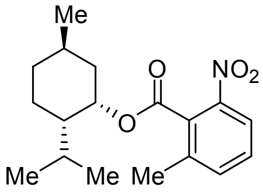

47

150423

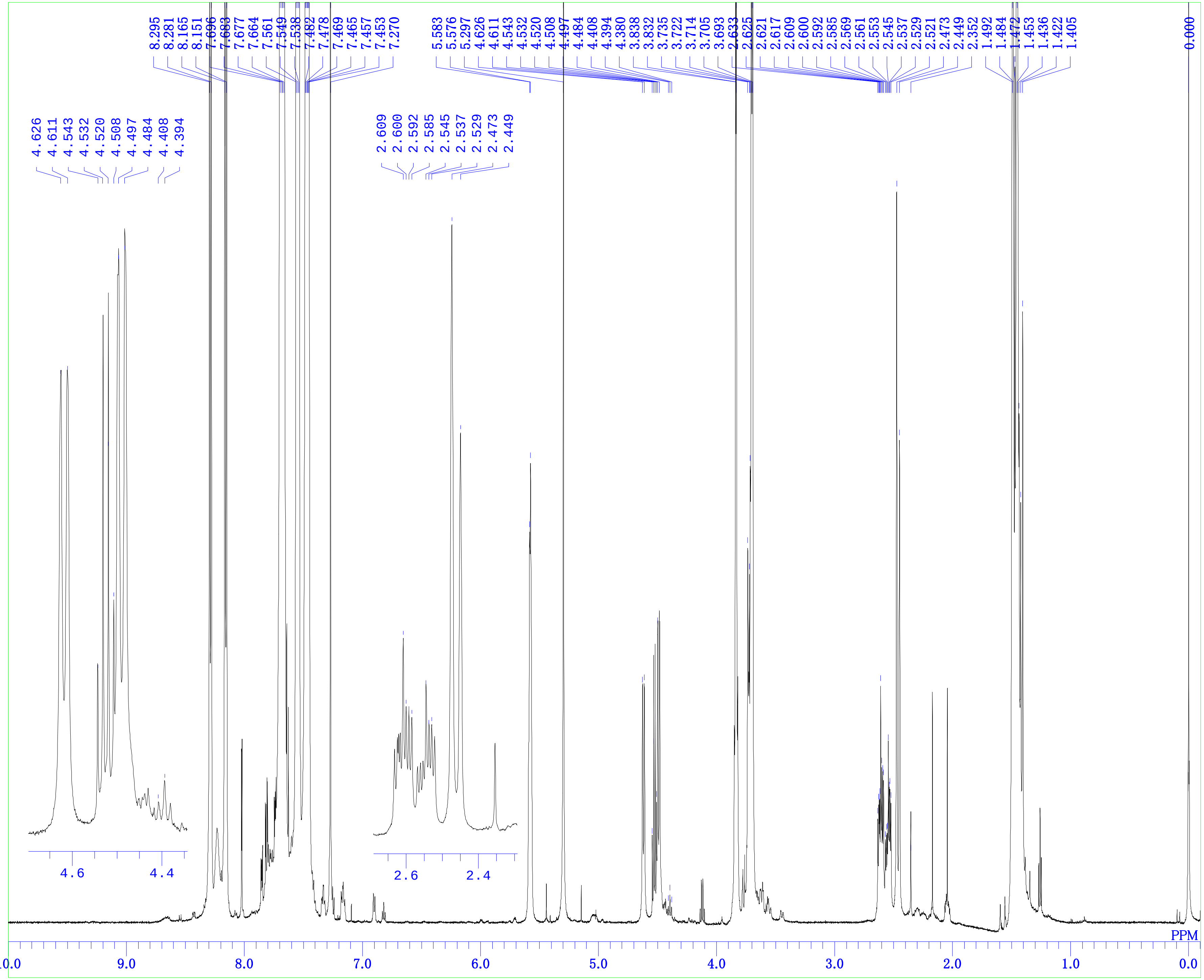

DFILE	d1561-crude-diastereo-1.jdf
COMNT	150423
DATIM	2015-04-23 15:29:37
OBNUC	1H
EXMOD	single_pulse.ex2
OBFRQ	600.17 MHz
OBSET	5.30 KHz
OBFIN	5.47 Hz
POINT	32768
FREQU	11261.26 Hz
SCANS	32
ACQTM	2.9098 sec
PD	2.0000 sec
PW1	7.30 usec
IRNUC	1H
CTEMP	21.4 c
SLVNT	CDCL3
EXREF	0.00 ppm
BF	0.12 Hz
RGAIN	38

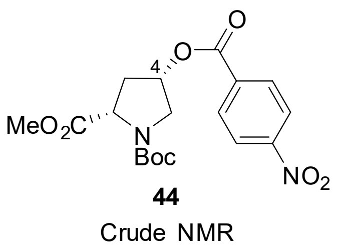

44

Crude NMR

151211

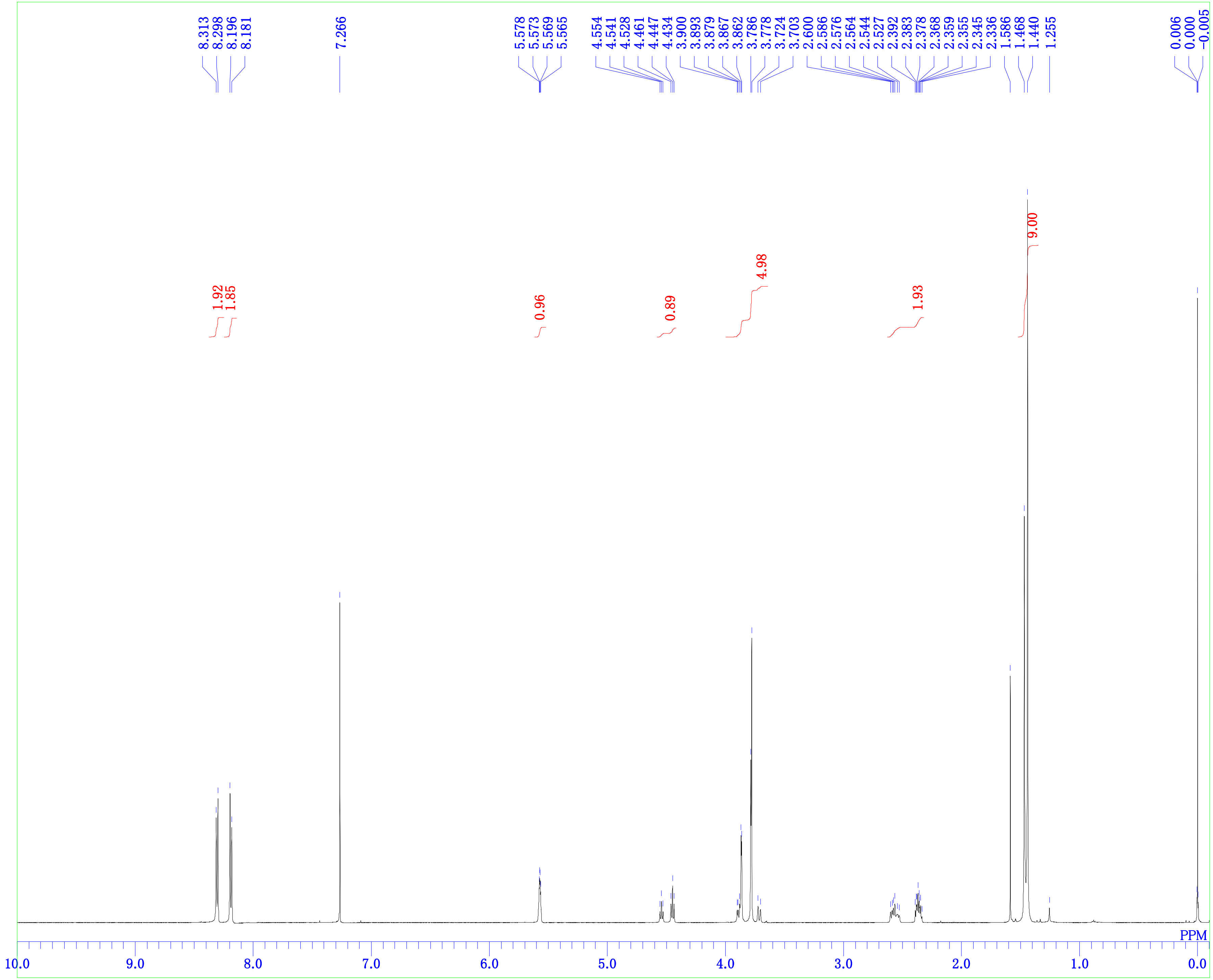

DFILE	d1564-a-1.als
COMNT	151211
DATIM	2015-12-11 13:35:47
OBNUC	1H
EXMOD	single_pulse.ex2
OBFRQ	600.17 MHz
OBSET	5.30 KHz
OBFIN	5.47 Hz
POINT	26214
FREQU	9008.87 Hz
SCANS	32
ACQTM	2.9098 sec
PD	2.0000 sec
PW1	7.30 usec
IRNUC	1H
CTEMP	20.3 c
SLVNT	CDCL3
EXREF	0.00 ppm
BF	0.12 Hz
RGAIN	46

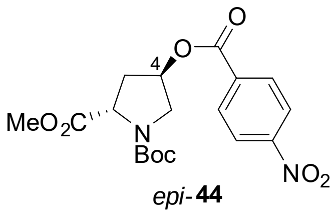

epi-**44**

150402

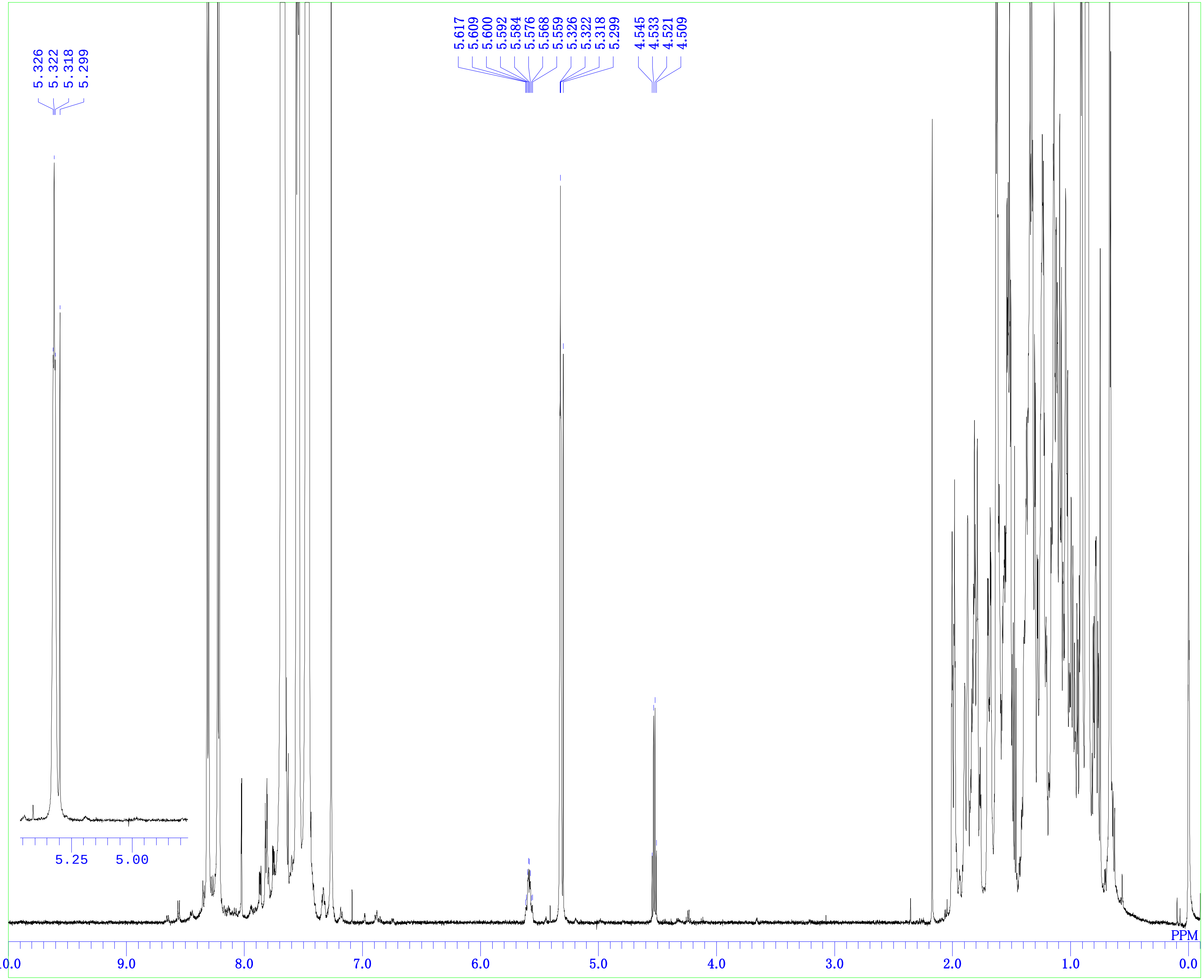

DFILE	d1534-crude-diastereo-1.als
COMNT	150402
DATIM	2015-04-02 10:17:19
OBNUC	1H
EXMOD	single_pulse.ex2
OBFRQ	600.17 MHz
OBSET	5.30 KHz
OBFIN	5.47 Hz
POINT	26214
FREQU	9008.87 Hz
SCANS	32
ACQTM	2.9098 sec
PD	2.0000 sec
PW1	7.30 usec
IRNUC	1H
CTEMP	20.7 c
SLVNT	CDCL3
EXREF	0.00 ppm
BF	0.12 Hz
RGAIN	40

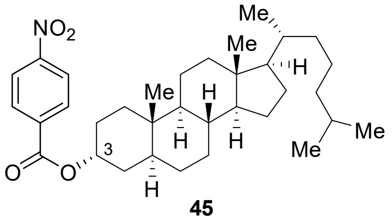

45

Crude NMR

151204

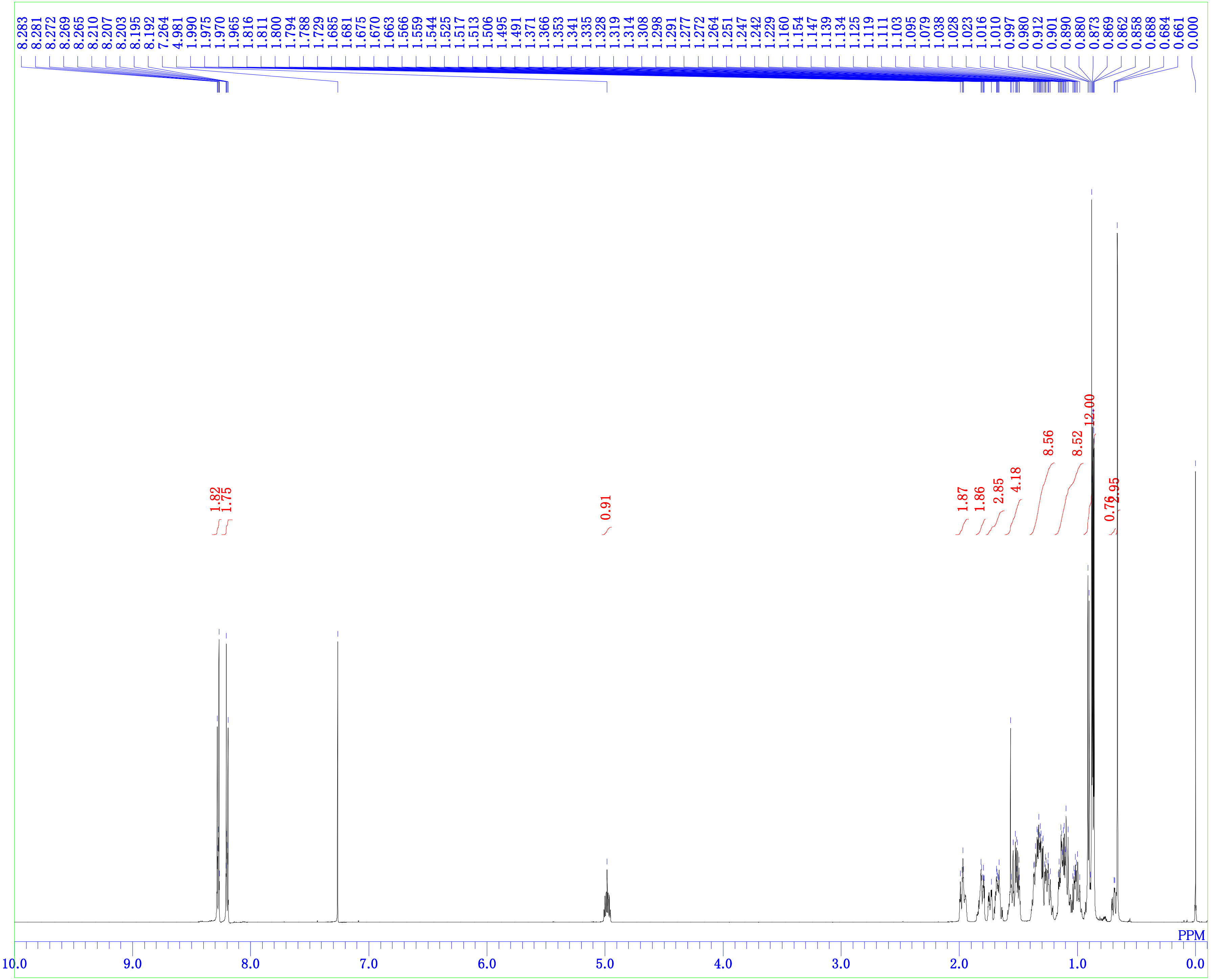

DFILE	d1550-151204.jdf
COMNT	151204
DATIM	2015-12-04 15:45:47
OBNUC	1H
EXMOD	single_pulse.ex2
OBFRQ	600.17 MHz
OBSET	5.30 KHz
OBFIN	5.47 Hz
POINT	32768
FREQU	11261.26 Hz
SCANS	32
ACQTM	2.9098 sec
PD	2.0000 sec
PW1	6.90 usec
IRNUC	1H
CTEMP	19.5 c
SLVNT	CDCL3
EXREF	0.00 ppm
BF	0.12 Hz
RGAIN	38

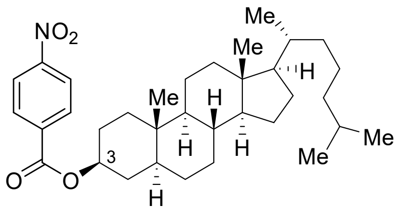

epi-**45**

150511

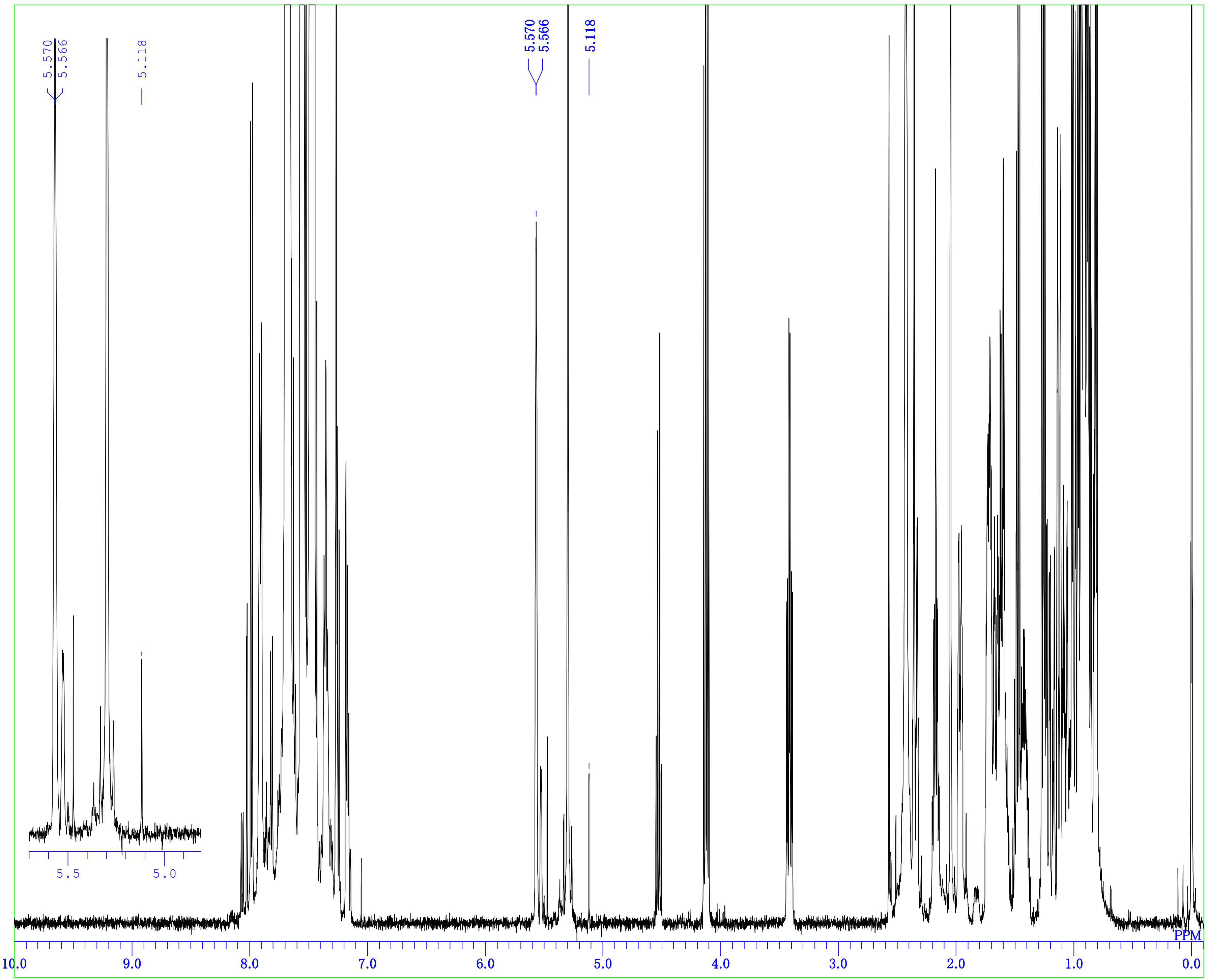

DFILE d1580-crude-1.als
COMNT 150511
DATIM 2015-05-11 13:53:49
OBNUC 1H
EXMOD single_pulse.ex2
OBFRQ 500.16 MHz
OBSET 2.41 KHz
OBFIN 6.01 Hz
POINT 13107
FREQU 7507.39 Hz
SCANS 8
ACQTM 1.7459 sec
PD 2.0000 sec
PW1 5.80 usec
IRNUC 1H
CTEMP 20.4 c
SLVNT CDCL3
EXREF 0.00 ppm
BF 0.12 Hz
RGAIN 42

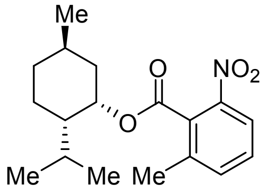

47

Crude NMR

151204

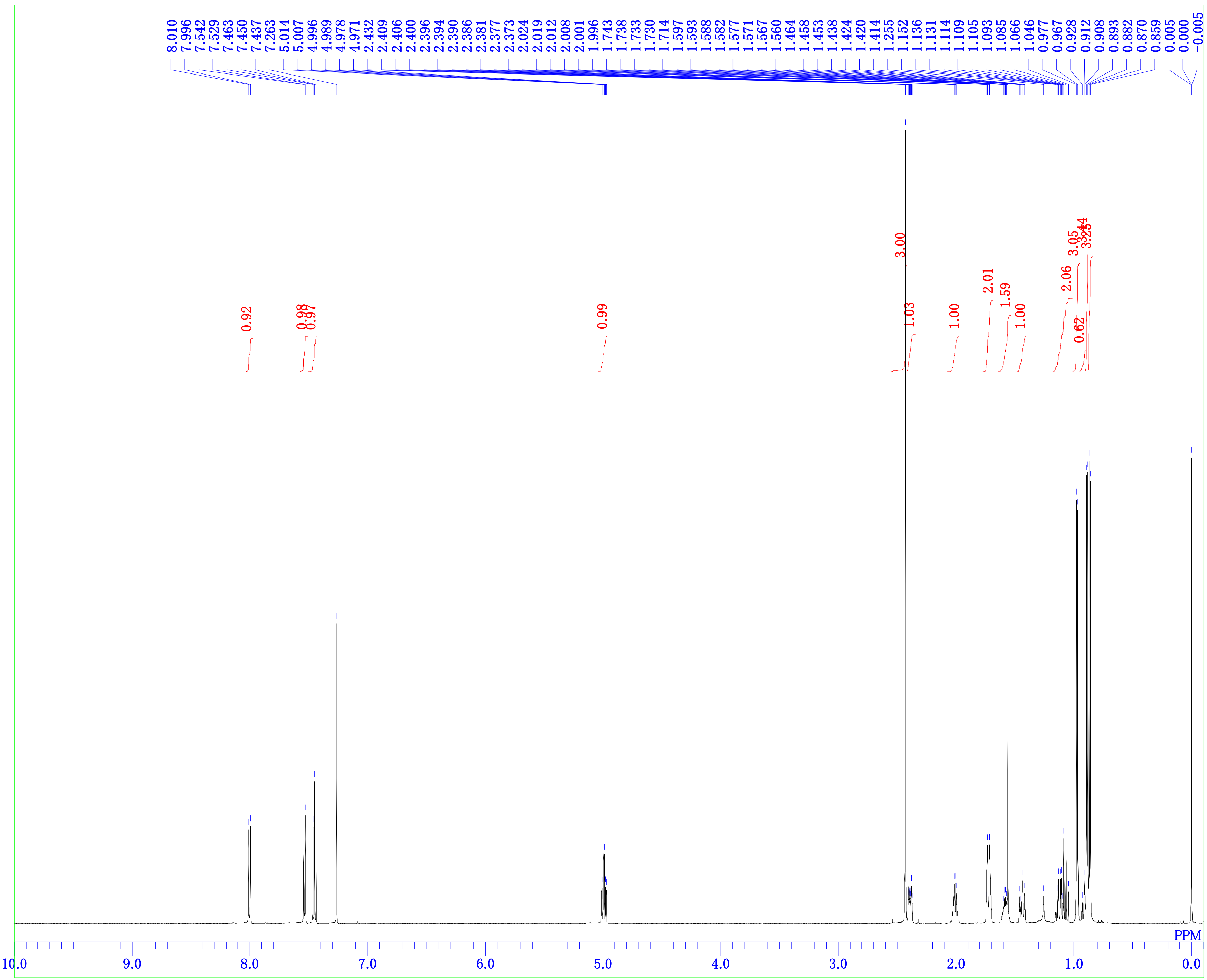

DFILE	d1656-151204.jdf
COMNT	151204
DATIM	2015-12-04 16:02:53
OBNUC	1H
EXMOD	single_pulse.ex2
OBFRQ	600.17 MHz
OBSET	5.30 KHz
OBFIN	5.47 Hz
POINT	32768
FREQU	11261.26 Hz
SCANS	32
ACQTM	2.9098 sec
PD	2.0000 sec
PW1	6.90 usec
IRNUC	1H
CTEMP	19.7 c
SLVNT	CDCL3
EXREF	0.00 ppm
BF	0.12 Hz
RGAIN	42

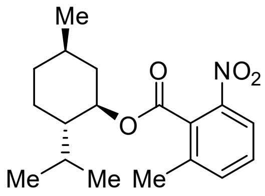

epi-**47**

5-racemic

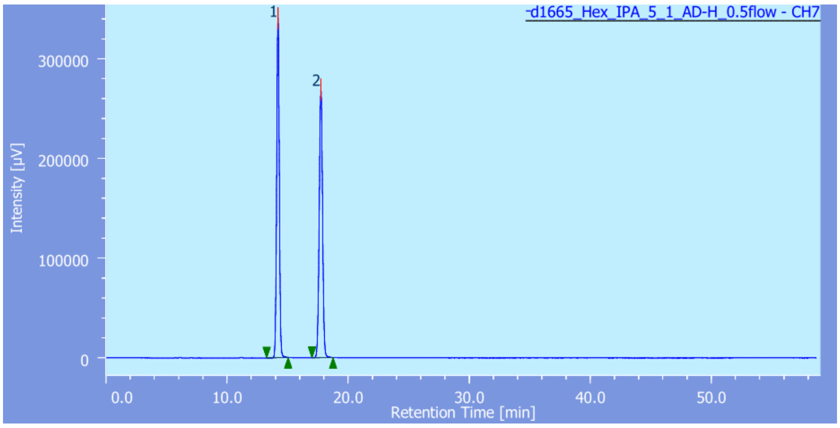

5-retention

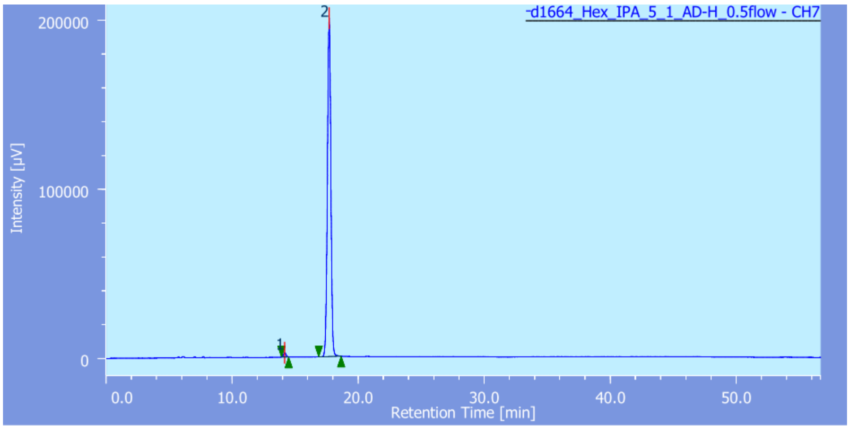

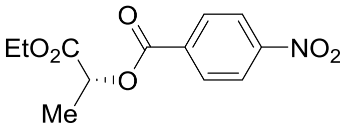

5

5-sample

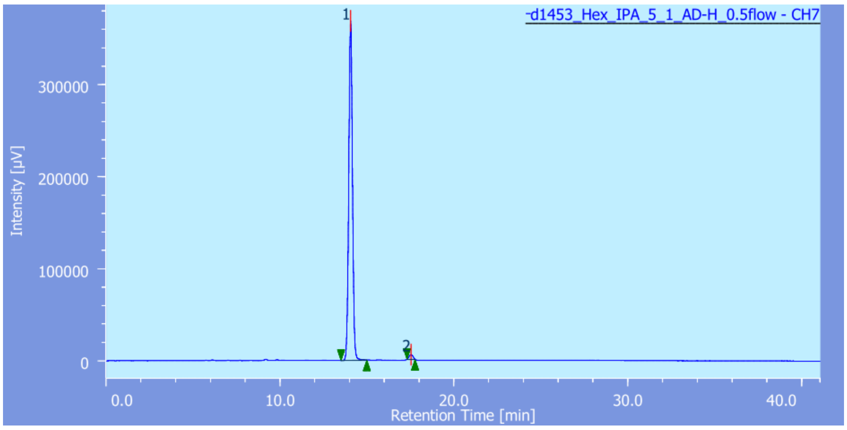

racemic mixture	tR (min)	area	area%
peak1	14.200	5087252	49.748
peak2	17.750	5138742	50.252
retention	tR (min)	area	area%
peak1	14.167	34569	0.904
peak2	17.700	3788748	99.096
sample	tR (min)	area	area%
peak1	14.058	5475109	98.611
peak2	17.533	77132	1.389

34-racemic

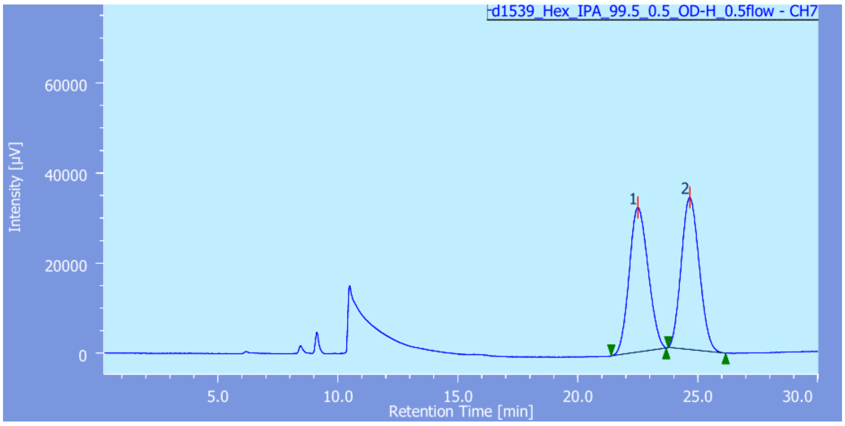

34-retention

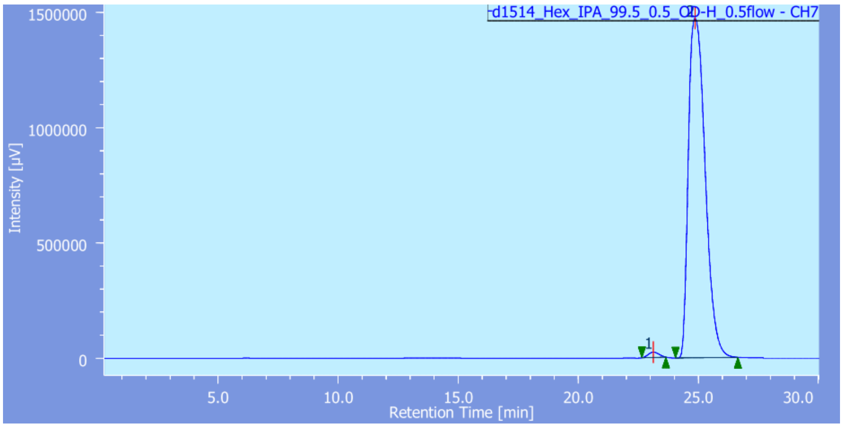

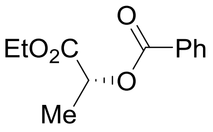

34

34-sample

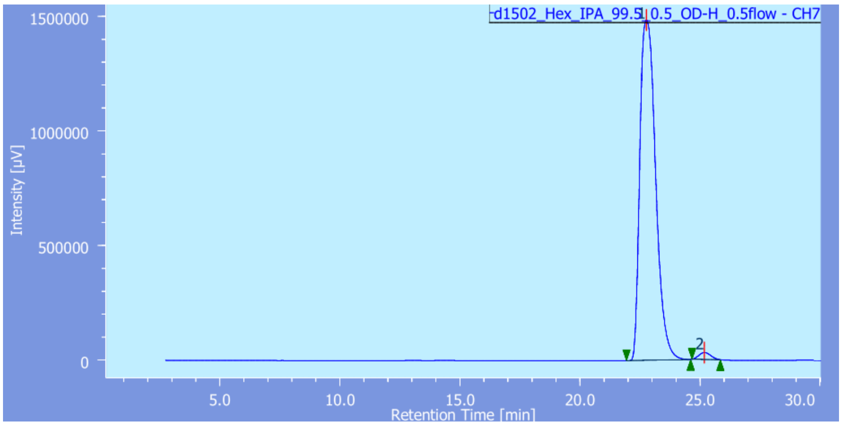

racemic mixture	tR (min)	area	area%
peak1	22.500	1762124	50.057
peak2	24.658	1758100	49.943
retention	tR (min)	area	area%
peak1	23.125	706735	0.980
peak2	24.858	71439775	99.020
sample	tR (min)	area	area%
peak1	22.758	67663135	98.502
peak2	25.175	1029277	1.498

35-racemic

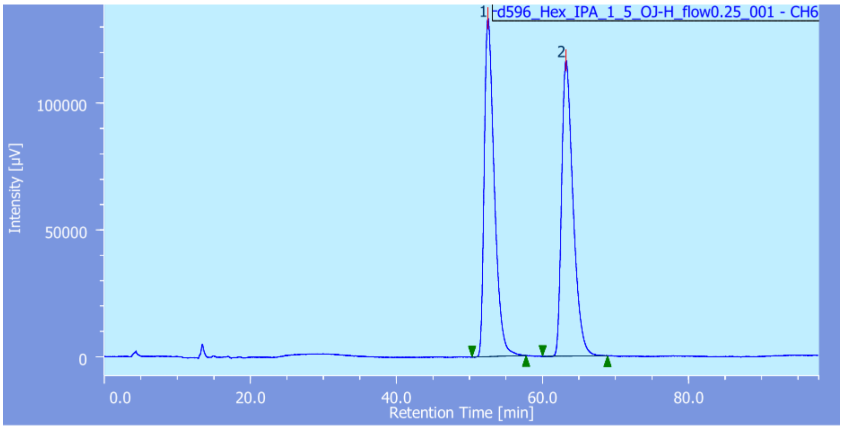

35-retention

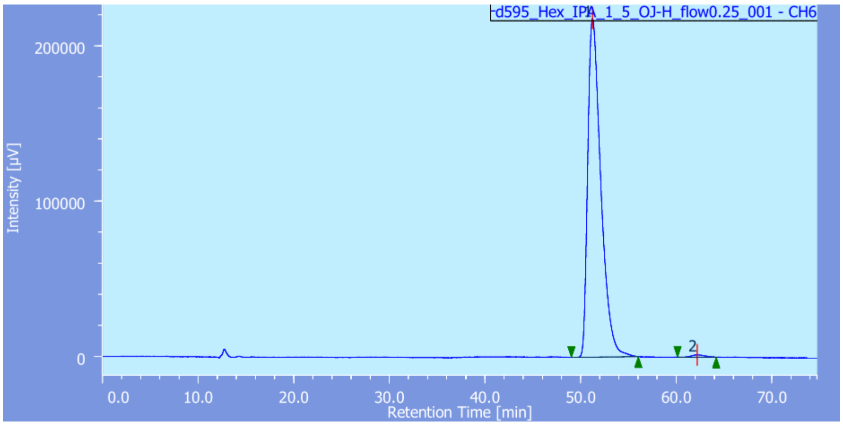

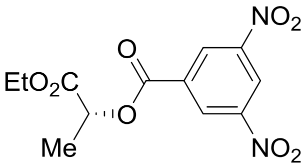

35

35-sample

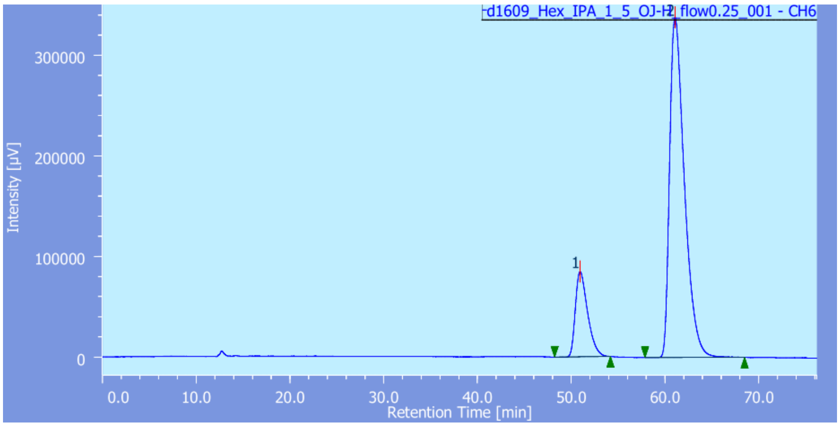

racemic mixture	tR (min)	area	area%
peak1	52.542	12747201	50.116
peak2	63.217	12688155	49.884
retention	tR (min)	area	area%
peak1	51.242	20707713	99.315
peak2	62.192	142775	0.685
sample	tR (min)	area	area%
peak1	50.950	7664177	17.167
peak2	61.067	36980193	82.833

36-racemic

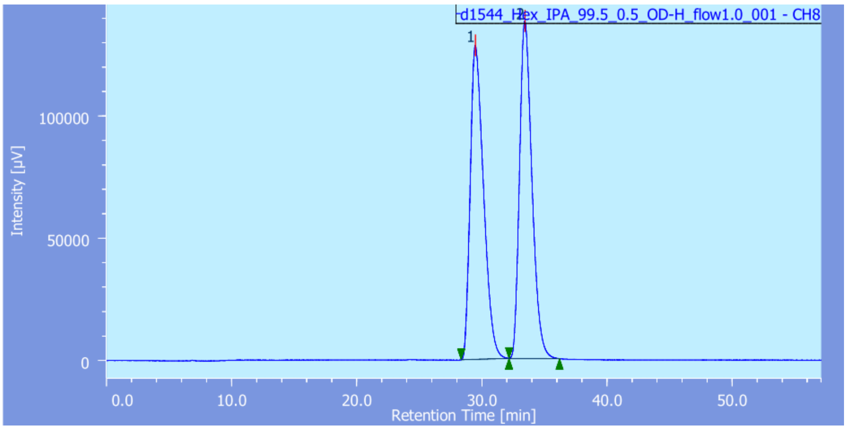

36-retention

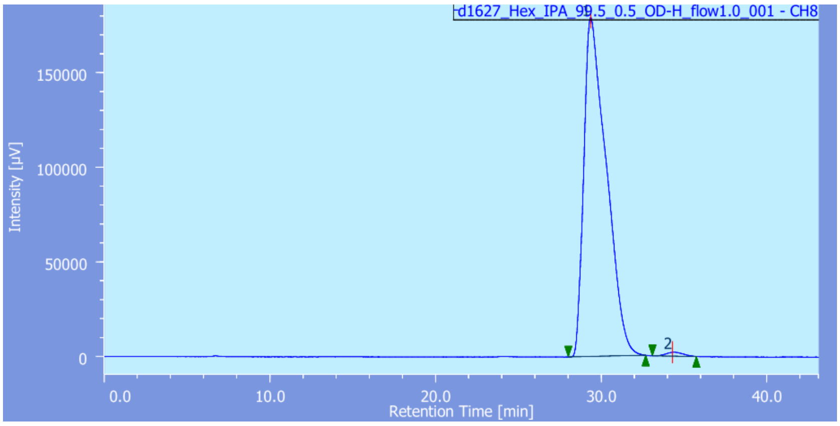

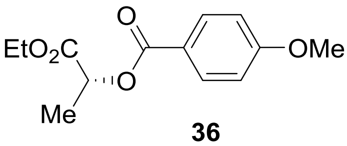

36

36-sample

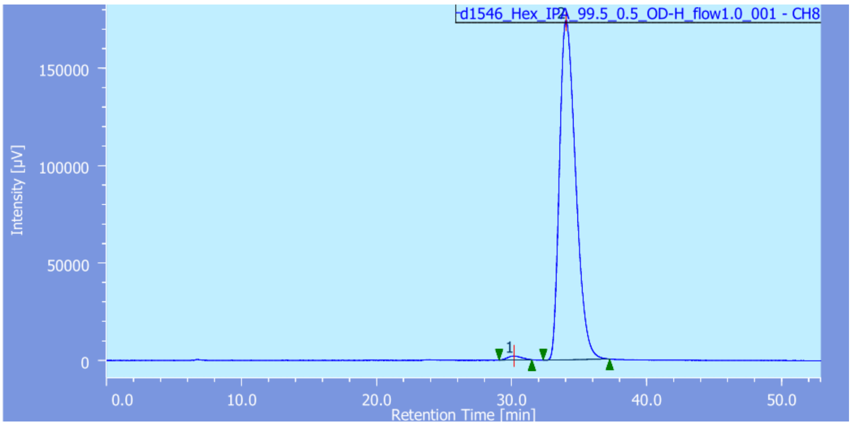

racemic mixture	tR (min)	area	area%
peak1	29.475	9510076	49.992
peak2	33.425	9513156	50.008
retention	tR (min)	area	area%
peak1	29.358	16911707	99.121
peak2	34.308	149995	0.879
sample	tR (min)	area	area%
peak1	30.183	146556	1.001
peak2	34.025	14495151	98.999

37-racemic

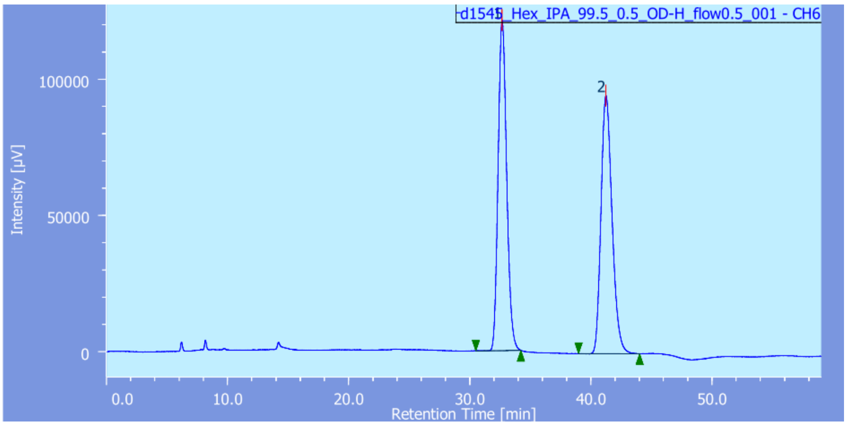

37-retention

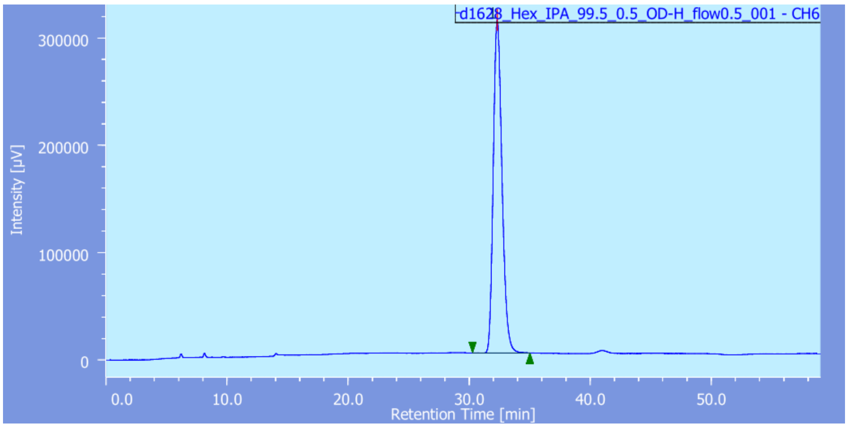

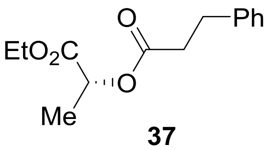

37

37-sample

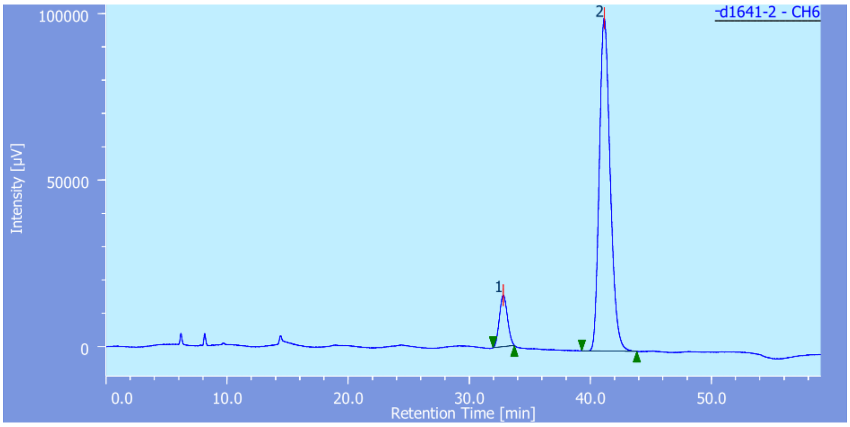

racemic mixture	tR (min)	area	area%
peak1	32.658	5946601	49.754
peak2	41.233	6005373	50.246
retention	tR (min)	area	area%
peak1	32.308	15520584	100.000
peak2			
sample	tR (min)	area	area%
peak1	32.800	713411	10.236
peak2	41.133	6255903	89.764

38-racemic

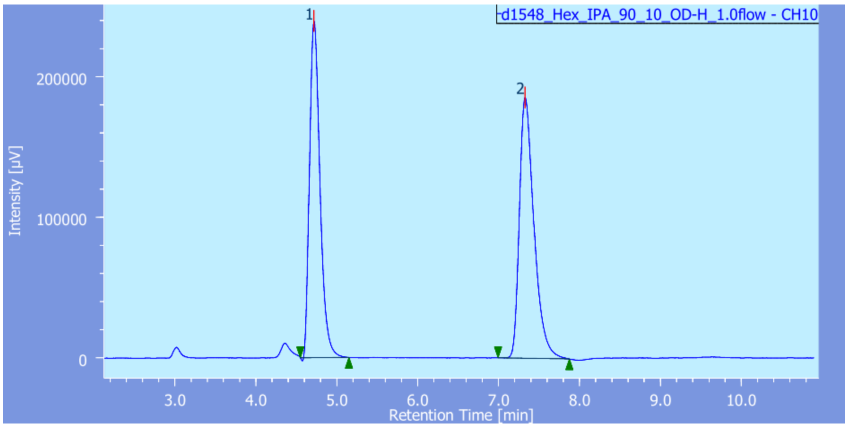

38-sample

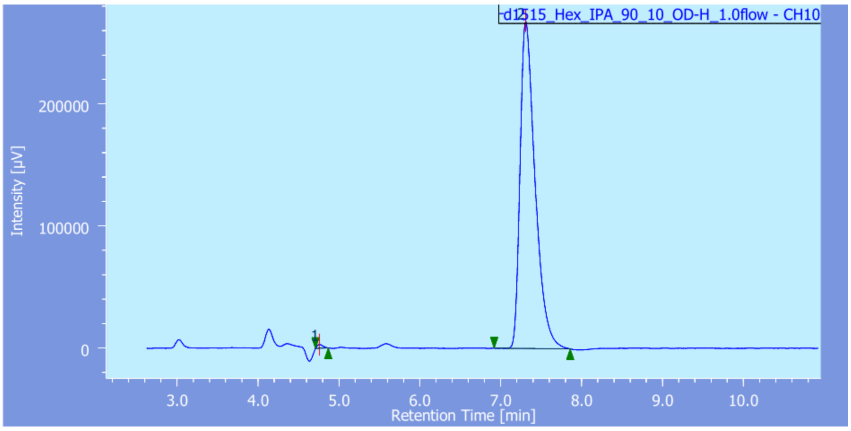

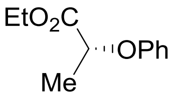

38

racemic mixture	tR (min)	area	area%
peak1	4.717	2147247	48.058
peak2	7.325	2320769	51.942
sample	tR (min)	area	area%
peak1	4.758	15534	0.448
peak2	7.308	3450655	99.552

39-racemic

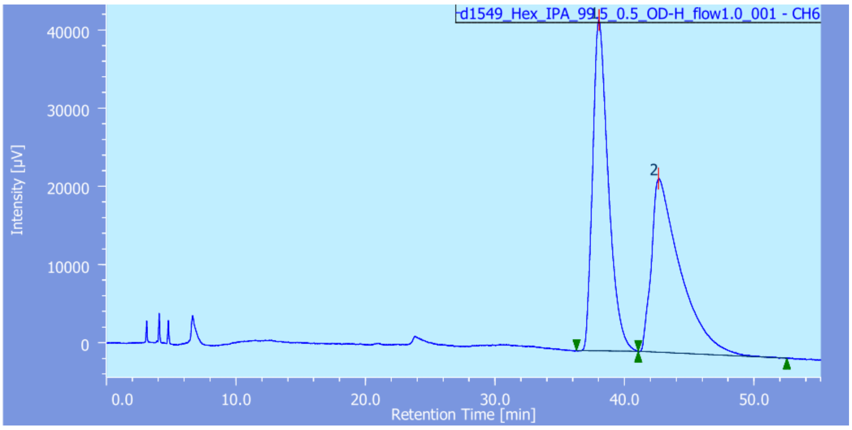

39-sample

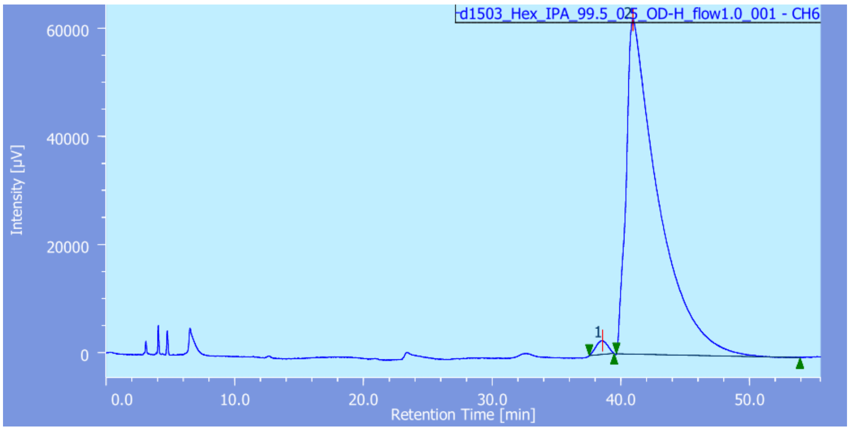

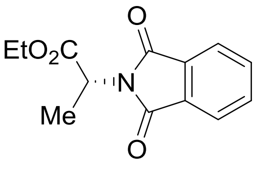

39

racemic mixture	tR (min)	area	area%
peak1	38.008	3546595	50.756
peak2	42.633	3440934	49.244
sample	tR (min)	area	area%
peak1	38.583	161011	1.479
peak2	40.925	10727198	98.521

40-racemic

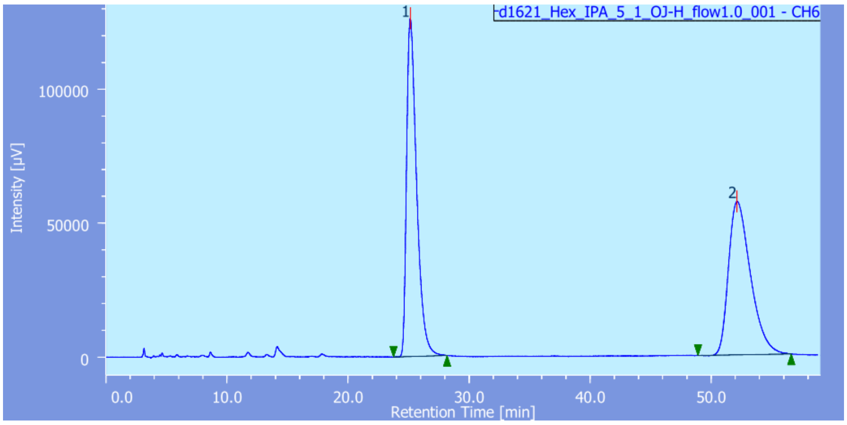

40-retention

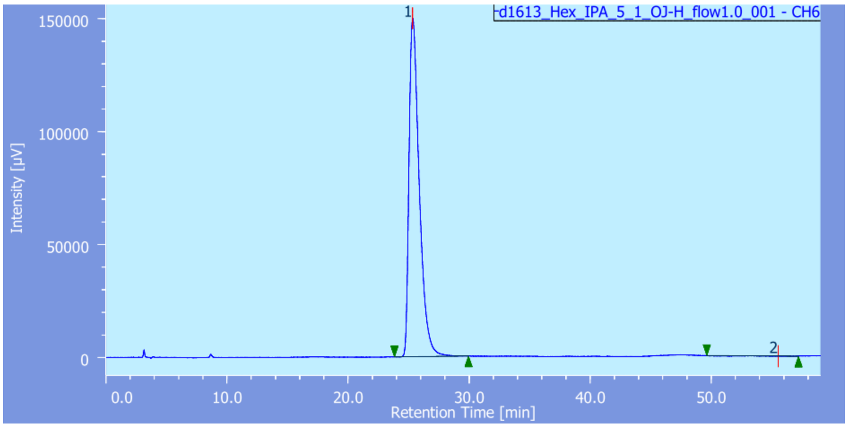

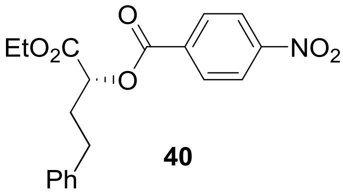

40

40-sample

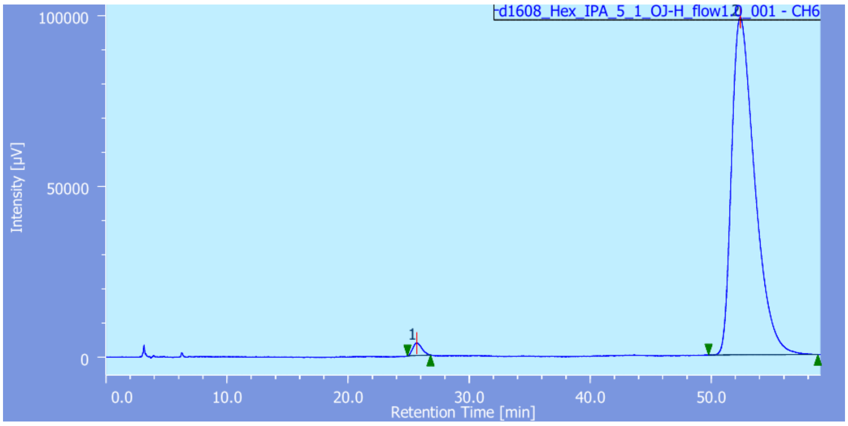

racemic mixture	tR (min)	area	area%
peak1	25.133	7263509	50.114
peak2	52.133	7230515	49.886
retention	tR (min)	area	area%
peak1	25.333	8802532	99.688
peak2	55.517	27550	0.312
sample	tR (min)	area	area%
peak1	25.683	191068	1.438
peak2	52.392	13093028	98.562

41-racemic

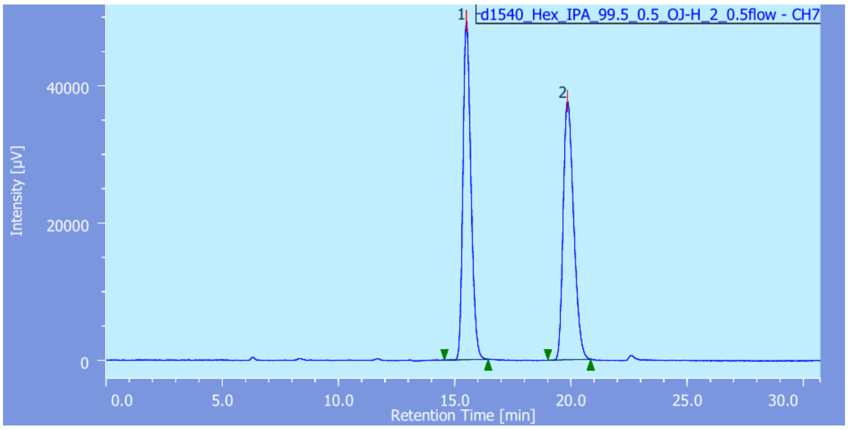

41-retention

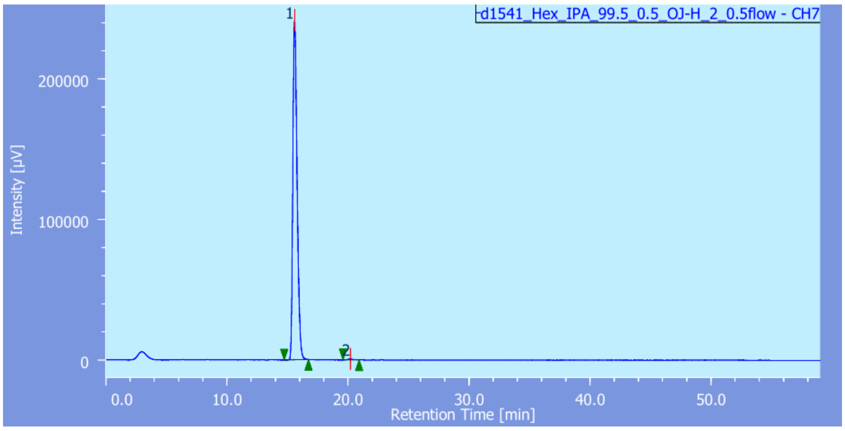

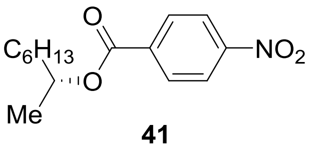

41

41-sample

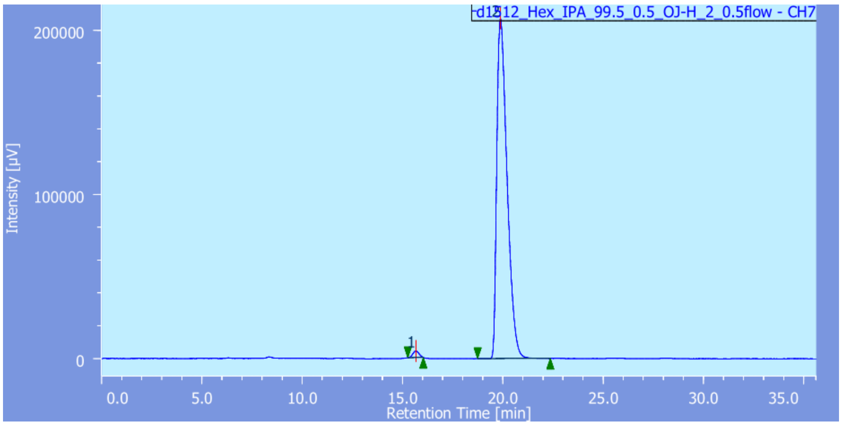

racemic mixture	tR (min)	area	area%
peak1	15.500	1180115	49.979
peak2	19.850	1181109	50.021
retention	tR (min)	area	area%
peak1	15.583	5979359	99.684
peak2	20.200	18955	0.316
sample	tR (min)	area	area%
peak1	15.658	86560	1.158
peak2	19.875	7388866	98.842

42-racemic

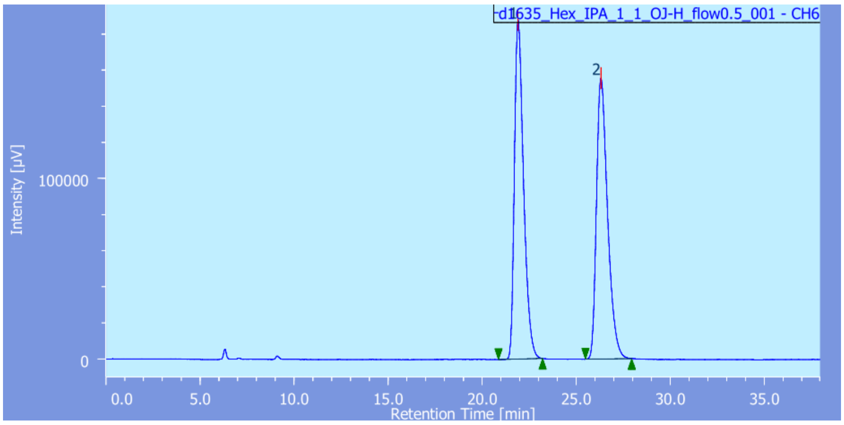

42-retention

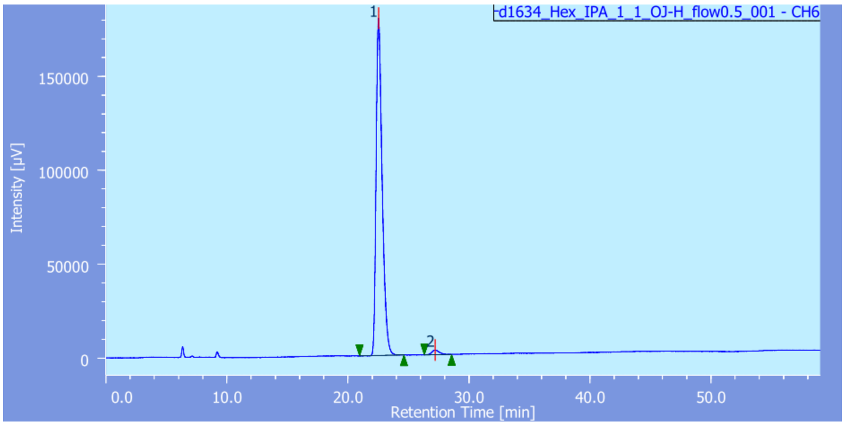

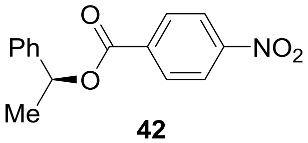

42

42-sample

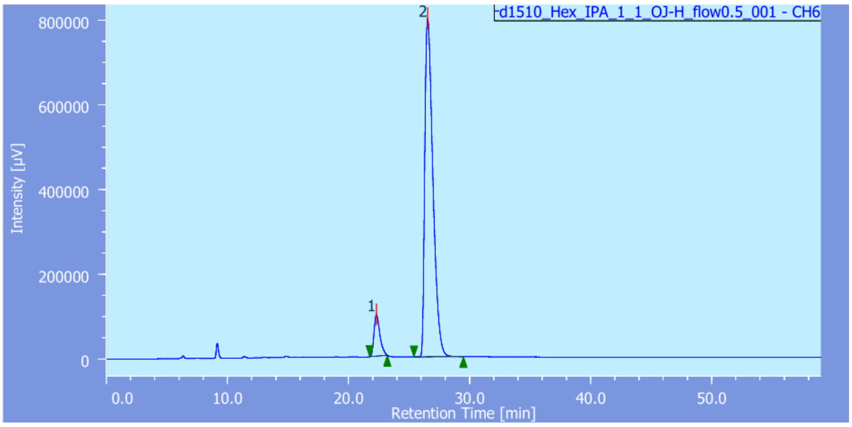

racemic mixture	tR (min)	area	area%
peak1	21.900	6358564	49.968
peak2	26.308	6366649	50.032
retention	tR (min)	area	area%
peak1	22.517	6321087	98.314
peak2	27.183	108430	1.686
sample	tR (min)	area	area%
peak1	22.292	3334306	8.216
peak2	26.533	37249530	91.784

43-racemic

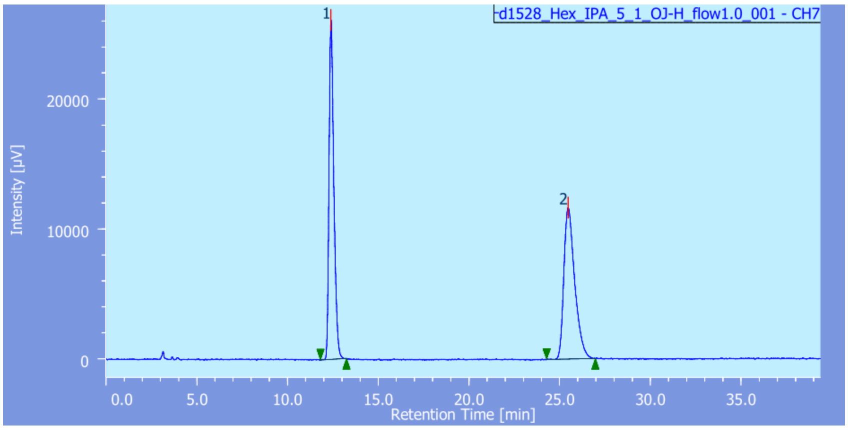

43-retention

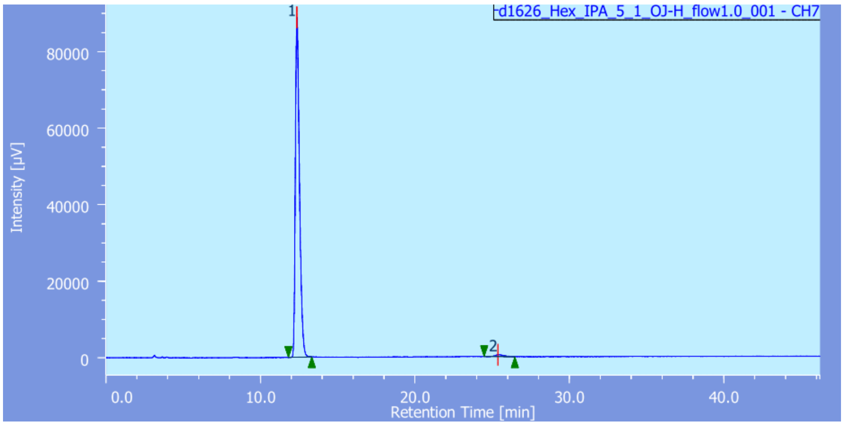

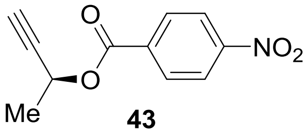

43

43-sample

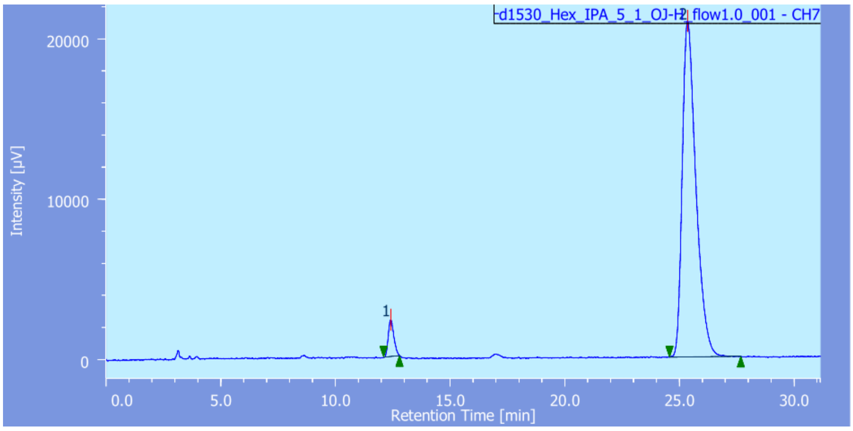

racemic mixture	tR (min)	area	area%
peak1	12.400	481719	49.988
peak2	25.458	481944	50.012
retention	tR (min)	area	area%
peak1	12.358	1659963	98.746
peak2	25.358	21073	1.254
sample	tR (min)	area	area%
peak1	12.408	38783	4.287
peak2	25.350	865888	95.713

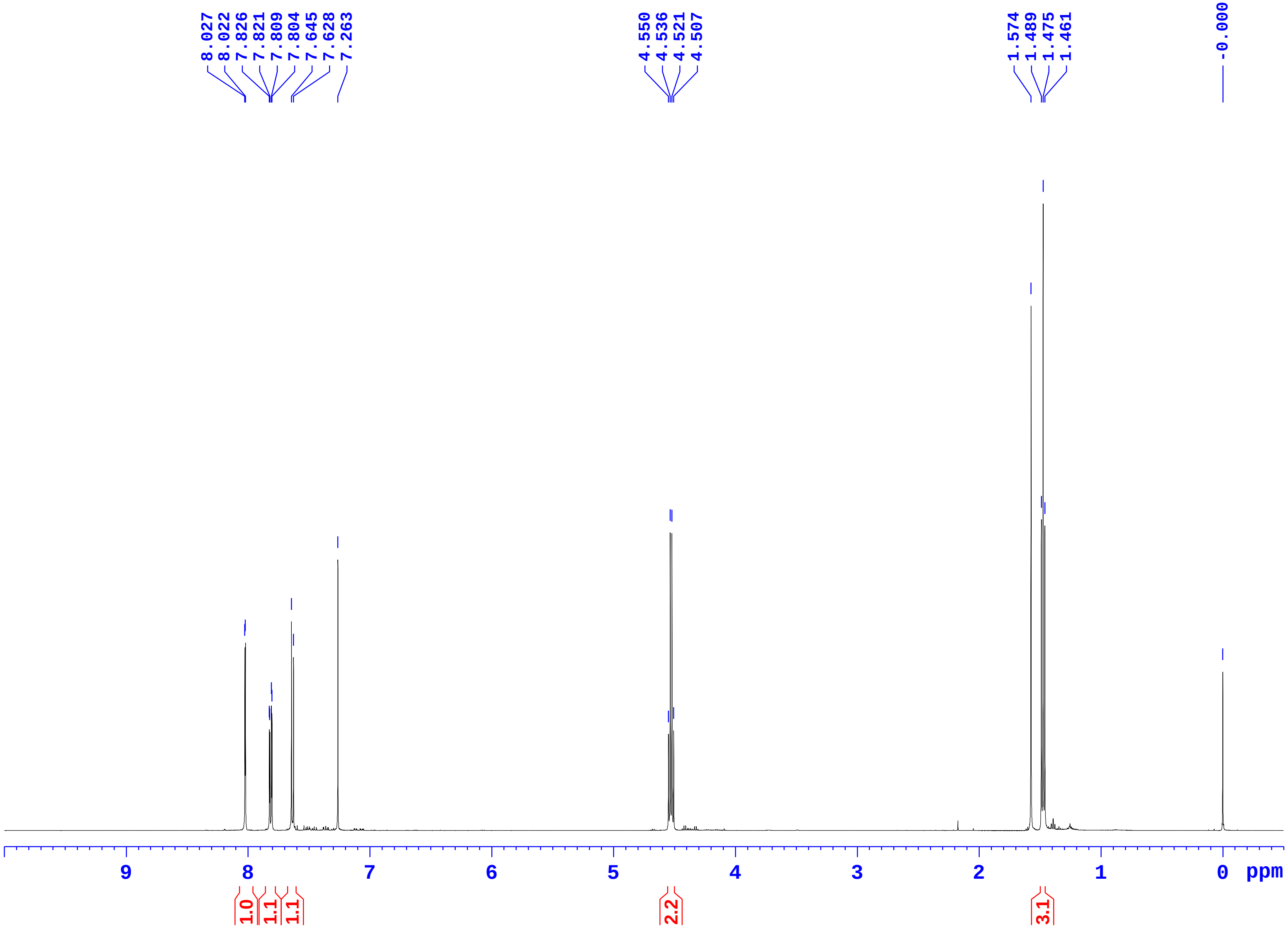

Current Data Parameters
NAME MGS-568
EXPNO 21
PROCNO 1

F2 - Acquisition Parameters
Date_ 20150617
Time 10.16
INSTRUM spect
PROBHD 5 mm PABBO BB-
PULPROG zg30
TD 65536
SOLVENT CDCl3
NS 16
DS 2
SWH 10330.578 Hz
FIDRES 0.157632 Hz
AQ 3.1719923 sec
RG 161
DW 48.400 usec
DE 6.50 usec
TE 296.0 K
D1 1.00000000 sec

======== CHANNEL f1 ========
NUC1 1H
P1 8.90 usec
PLW1 26.00000000 W
SFO1 500.1330885 MHz

F2 - Processing parameters
SI 65536
SF 500.1300121 MHz
WDW EM
SSB 0
LB 0.30 Hz
GB 0
PC 1.00

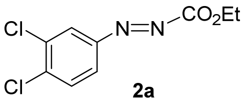

2a

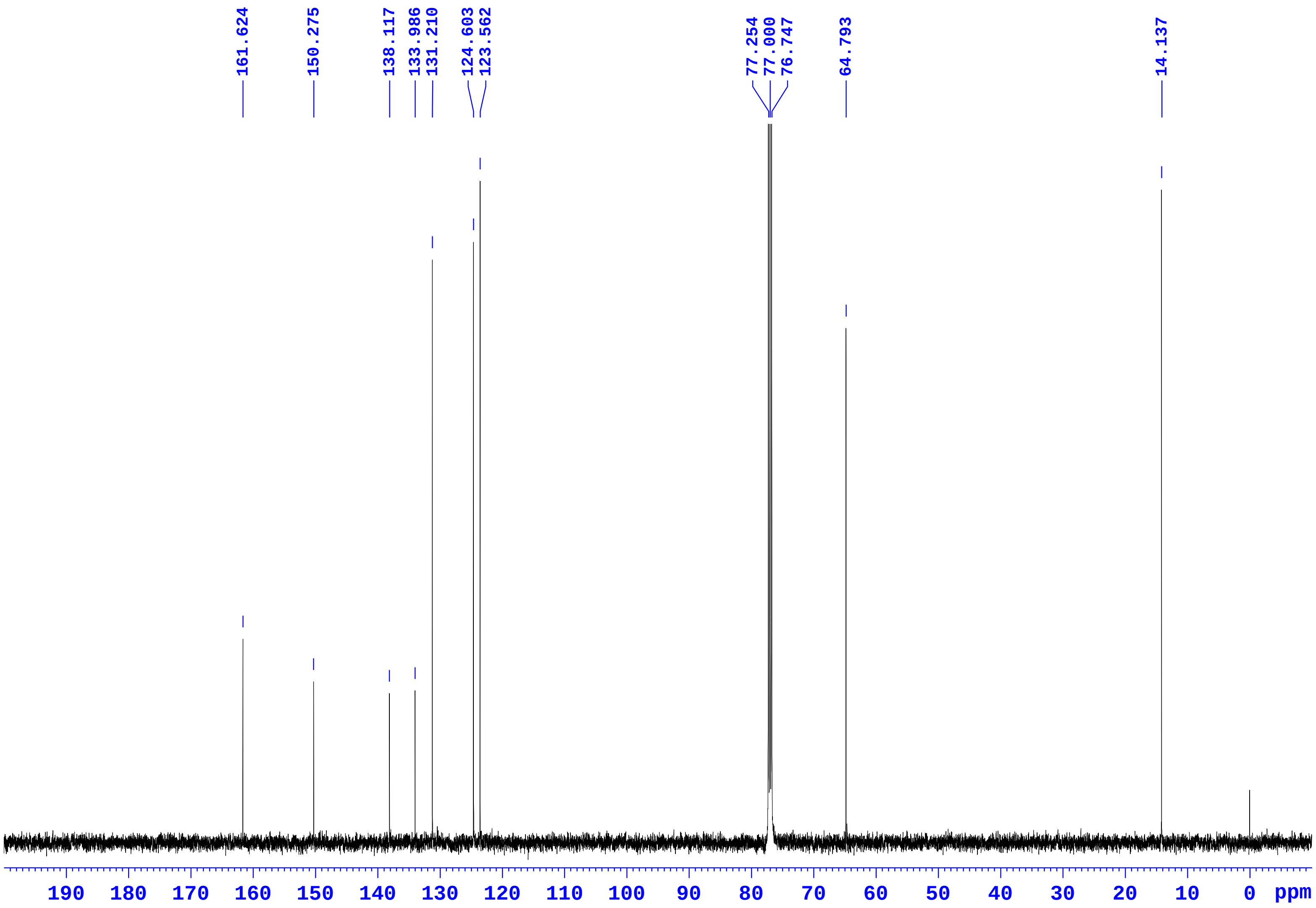


```
Current Data Parameters
NAME              MGS-568
EXPNO                  22
PROCNO                  1

F2 - Acquisition Parameters
Date_            20150617
Time                12.11
INSTRUM             spect
PROBHD    5 mm PABBO BB-
PULPROG           zgpg30
TD                 65536
SOLVENT            CDCl3
NS                  3072
DS                     4
SWH            29761.904 Hz
FIDRES          0.454131 Hz
AQ             1.1010548 sec
RG                  2050
DW                16.800 usec
DE                  6.50 usec
TE                 296.0 K
D1            1.00000000 sec
D11           0.03000000 sec

======== CHANNEL f1 ========
NUC1                 13C
P1                  9.00 usec
PLW1        122.00000000 W
SFO1         125.7703637 MHz

======== CHANNEL f2 ========
CPDPRG2          waltz16
NUC2                  1H
PCPD2              80.00 usec
PLW2         26.00000000 W
PLW12         0.32179001 W
PLW13         0.20595001 W
SFO2         500.1320005 MHz

F2 - Processing parameters
SI                 32768
SF           125.7577925 MHz
WDW                   EM
SSB        0
LB                  1.00 Hz
GB         0
PC                  1.40
```

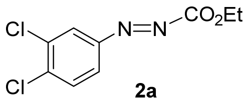

2a

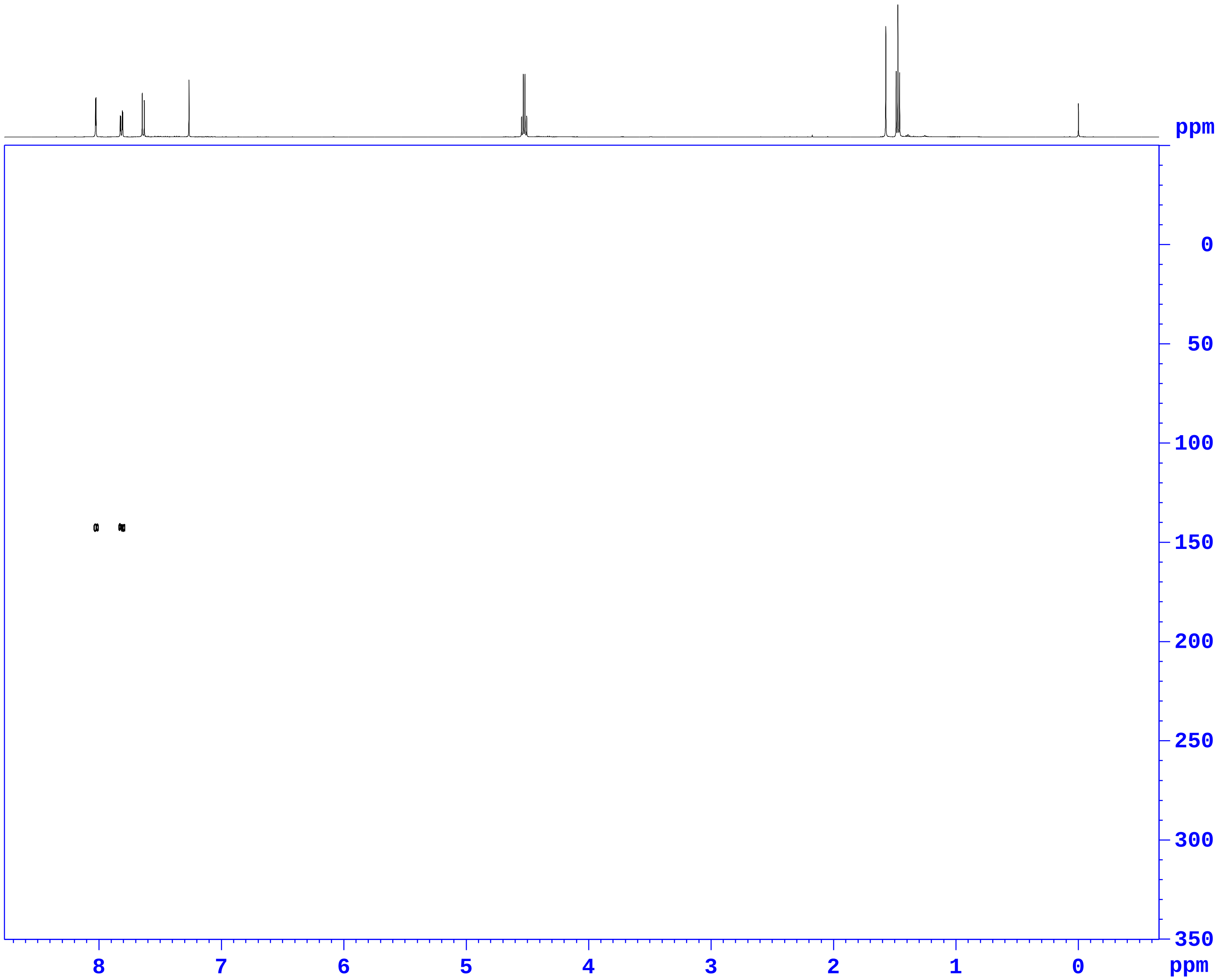


```
Current Data Parameters
NAME               MGS-568
EXPNO                   14
PROCNO                   1

F2 - Acquisition Parameters
Date_             20150615
Time                 21.52
INSTRUM              spect
PROBHD    5 mm PABBO BB-
PULPROG        hmbcgpndqf
TD                    2048
SOLVENT              CDCl3
NS                      64
DS                      16
SWH               4716.981 Hz
FIDRES            2.303213 Hz
AQ               0.2171380 sec
RG                    2050
DW                 106.000 usec
DE                    6.50 usec
TE                   296.0 K
CNST13           5.0000000
D0              0.00000300 sec
D1              1.94019794 sec
D6              0.10000000 sec
D16             0.00020000 sec
IN0             0.00002465 sec

======== CHANNEL f1 ========
NUC1                    1H
P1                    8.90 usec
P2                   17.80 usec
PLW1           26.00000000 W
SFO1           500.1320390 MHz

======== CHANNEL f2 ========
NUC2                   15N
P3                   14.40 usec
PLW2          206.00000000 W
SFO2            50.6853342 MHz

====== GRADIENT CHANNEL =====
GPNAM1          SMSQ10.100
GPNAM2          SMSQ10.100
GPNAM3          SMSQ10.100
GPZ1                 70.00 %
GPZ2                 30.00 %
GPZ3                 50.10 %
P16                1000.00 usec

F1 - Acquisition parameters
TD                     128
SFO1              50.68533 MHz
FIDRES          158.391663 Hz
SW                 400.000 ppm
FnMODE                  QF

F2 - Processing parameters
SI                    2048
SF             500.1300102 MHz
WDW                   SINE
SSB      0
LB       0 Hz
GB       0
PC                    1.40

F1 - Processing parameters
SI                    1024
MC2                     QF
SF              50.6777330 MHz
WDW                 States
SSB      0
LB       0 Hz
GB       0
```

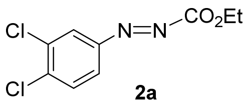

2a

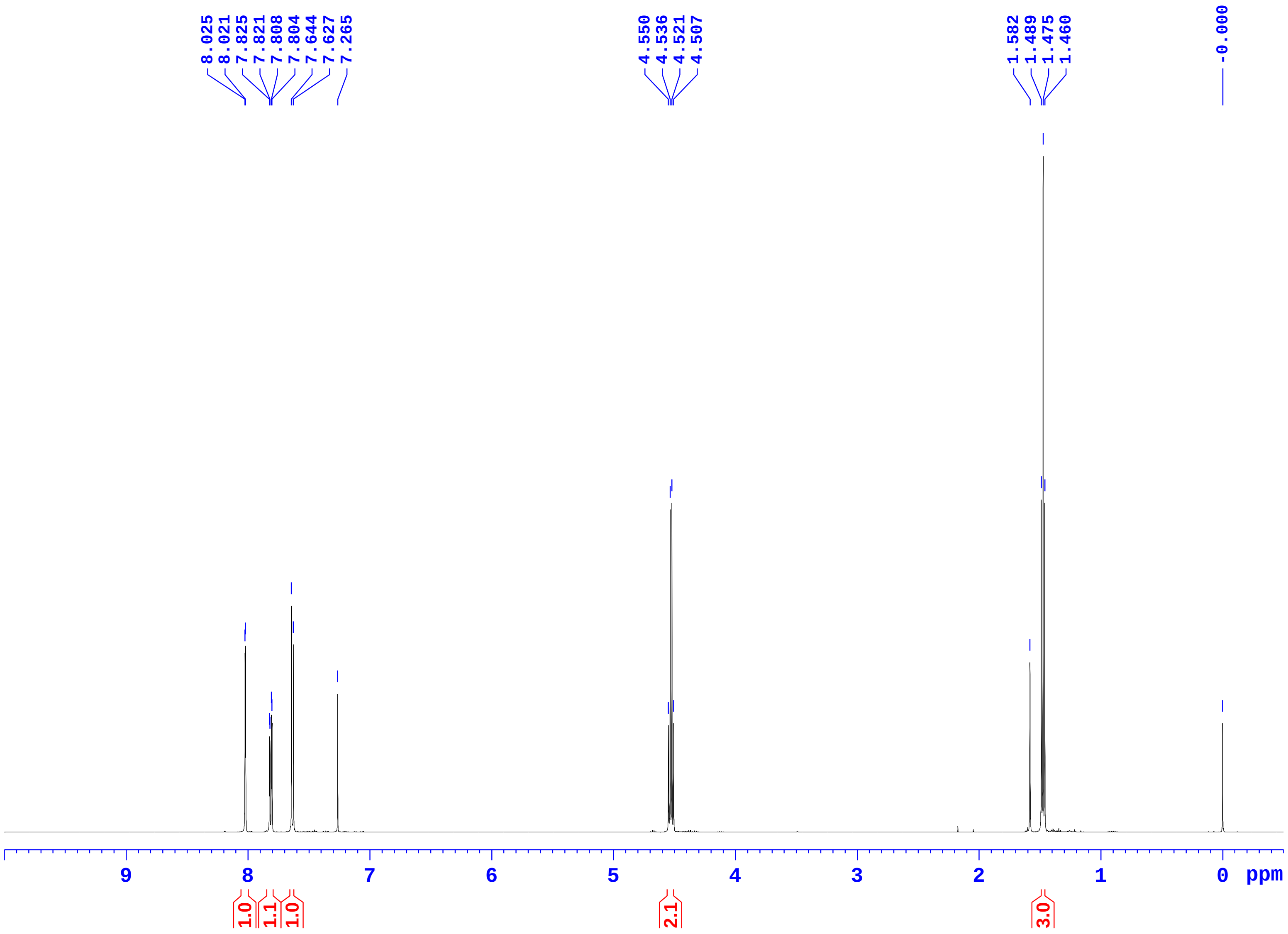


```
Current Data Parameters
NAME              mgs-576
EXPNO                   5
PROCNO                  1

F2 - Acquisition Parameters
Date_            20150607
Time                20.27
INSTRUM             spect
PROBHD   5 mm PABBO BB-
PULPROG              zg30
TD                  65536
SOLVENT             CDCl3
NS                     16
DS                      2
SWH            10330.578 Hz
FIDRES          0.157632 Hz
AQ             3.1719923 sec
RG                    144
DW                 48.400 usec
DE                   6.50 usec
TE                  296.0 K
D1             1.00000000 sec

======== CHANNEL f1 ========
NUC1                   1H
P1                   8.90 usec
PLW1          26.00000000 W
SFO1          500.1330885 MHz

F2 - Processing parameters
SI                  65536
SF            500.1300113 MHz
WDW                    EM
SSB      0
LB                   0.30 Hz
GB       0
PC                   1.00
```

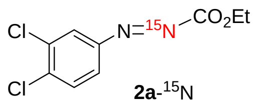

2a-^{15}N

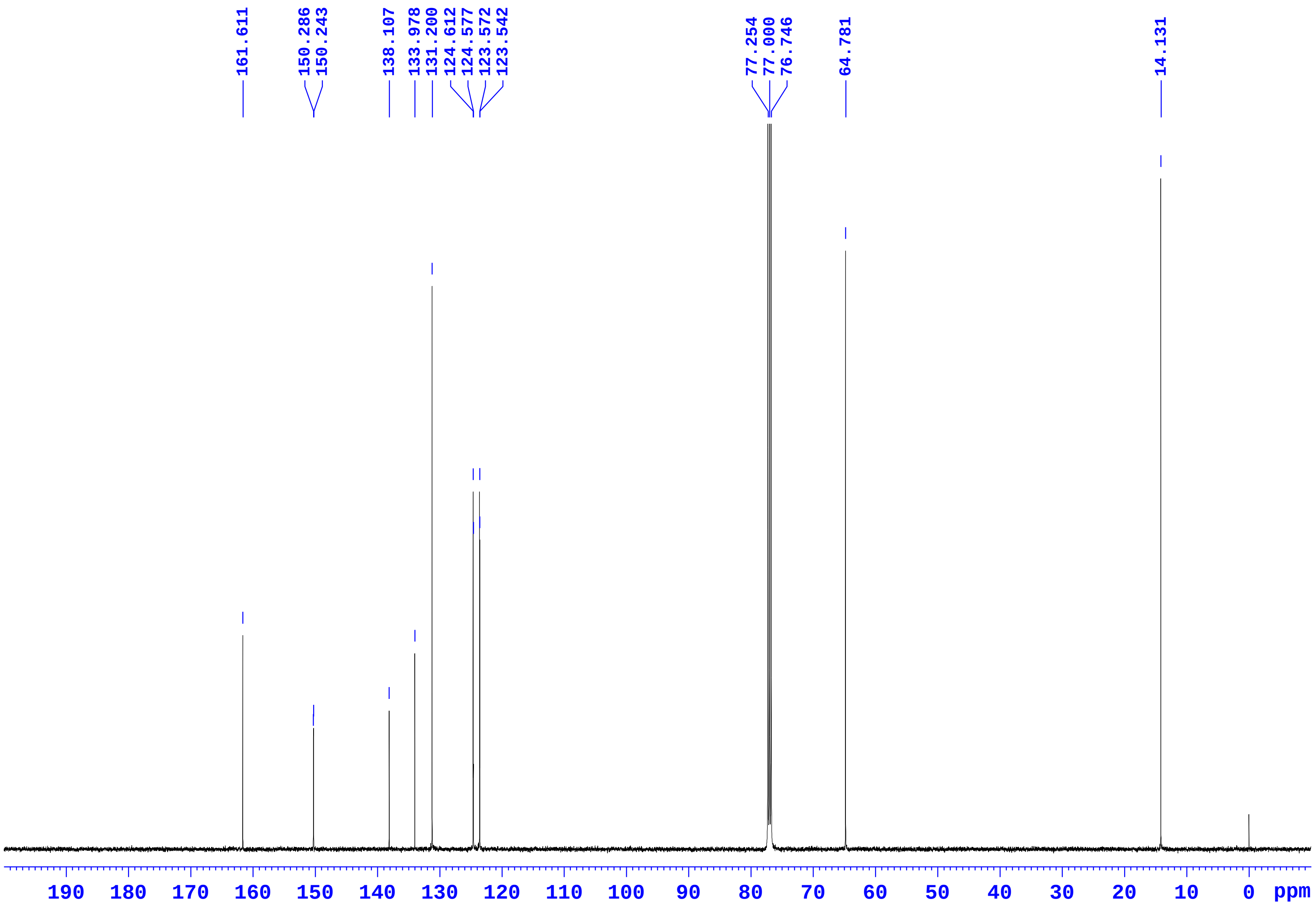


```
Current Data Parameters
NAME             mgs-576
EXPNO                  6
PROCNO                 1

F2 - Acquisition Parameters
Date_           20150608
Time                2.38
INSTRUM            spect
PROBHD   5 mm PABBO BB-
PULPROG          zgpg30
TD                 65536
SOLVENT            CDCl3
NS                 10240
DS                     4
SWH            29761.904 Hz
FIDRES          0.454131 Hz
AQ             1.1010548 sec
RG                  2050
DW                16.800 usec
DE                  6.50 usec
TE                 296.0 K
D1            1.00000000 sec
D11           0.03000000 sec

======== CHANNEL f1 ========
NUC1                 13C
P1                  9.00 usec
PLW1        122.00000000 W
SFO1         125.7703637 MHz

======== CHANNEL f2 ========
CPDPRG2          waltz16
NUC2                  1H
PCPD2              80.00 usec
PLW2         26.00000000 W
PLW12         0.32179001 W
PLW13         0.20595001 W
SFO2         500.1320005 MHz

F2 - Processing parameters
SI                 32768
SF           125.7577932 MHz
WDW                   EM
SSB      0
LB                  1.00 Hz
GB       0
PC                  1.40
```

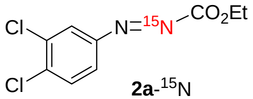

2a-^{15}N

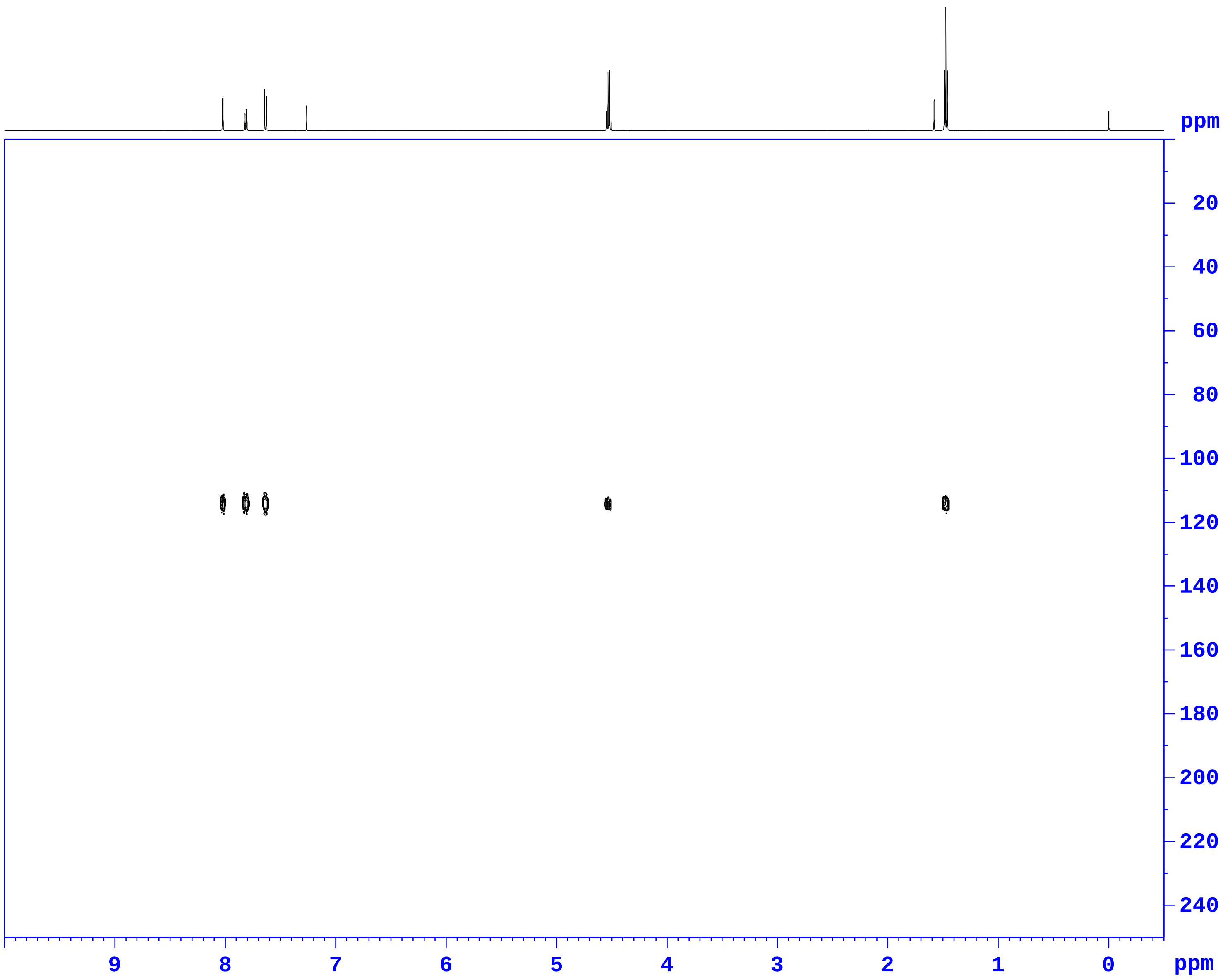

Current Data Parameters
NAME mgs-576
EXPNO 9
PROCNO 1

F2 - Acquisition Parameters
Date_ 20150608
Time 3.40
INSTRUM spect
PROBHD 5 mm PABBO BB-
PULPROG hmbcgpndqf
TD 2048
SOLVENT CDCl3
NS 8
DS 16
SWH 4672.897 Hz
FIDRES 2.281688 Hz
AQ 0.2191860 sec
RG 2050
DW 107.000 usec
DE 6.50 usec
TE 296.0 K
CNST13 5.0000000
D0 0.00000300 sec
D1 1.93815005 sec
D6 0.10000000 sec
D16 0.00020000 sec
IN0 0.00002465 sec

======== CHANNEL f1 ========
NUC1 1H
P1 8.90 usec
P2 17.80 usec
PLW1 26.00000000 W
SFO1 500.1320044 MHz

======== CHANNEL f2 ========
NUC2 15N
P3 14.40 usec
PLW2 206.00000000 W
SFO2 50.6777330 MHz

====== GRADIENT CHANNEL =====
GPNAM1 SMSQ10.100
GPNAM2 SMSQ10.100
GPNAM3 SMSQ10.100
GPZ1 70.00 %
GPZ2 30.00 %
GPZ3 50.10 %
P16 1000.00 usec

F1 - Acquisition parameters
TD 128
SFO1 50.67773 MHz
FIDRES 158.391663 Hz
SW 400.060 ppm
FnMODE QF

F2 - Processing parameters
SI 2048
SF 500.1300113 MHz
WDW SINE
SSB 0
LB 0 Hz
GB 0
PC 1.40

F1 - Processing parameters
SI 1024
MC2 QF
SF 50.6777330 MHz
WDW States
SSB 0
LB 0 Hz
GB 0

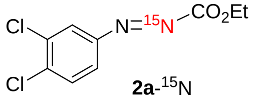

2a-^{15}N

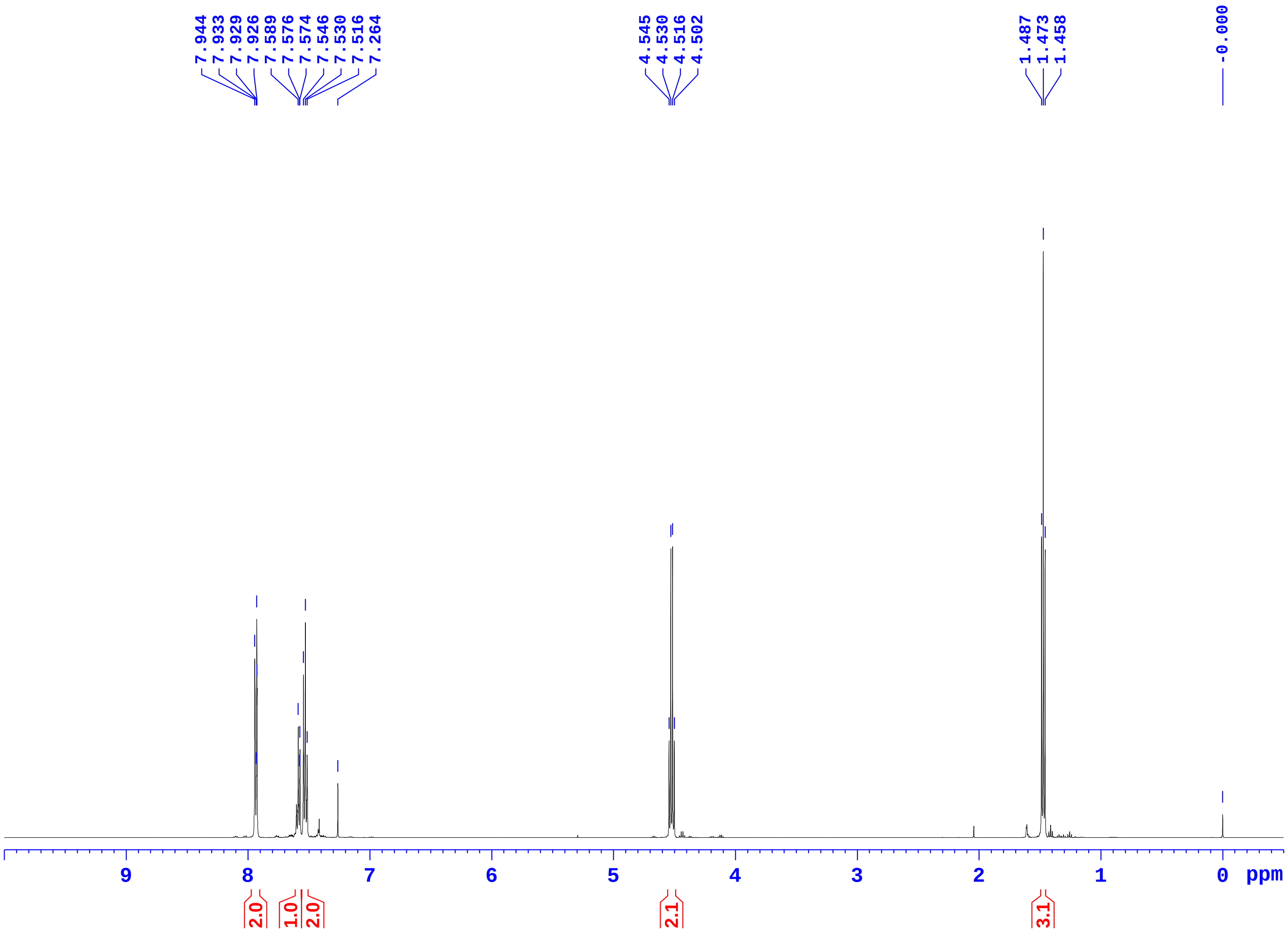


```
Current Data Parameters
NAME              MGS-661
EXPNO                   1
PROCNO                  1

F2 - Acquisition Parameters
Date_            20151102
Time                23.00
INSTRUM             spect
PROBHD   5 mm PABBO BB-
PULPROG              zg30
TD                  65536
SOLVENT             CDCl3
NS                     16
DS                      2
SWH             10330.578 Hz
FIDRES           0.157632 Hz
AQ              3.1719923 sec
RG                   80.6
DW                 48.400 usec
DE                   6.50 usec
TE                  296.0 K
D1             1.00000000 sec

======== CHANNEL f1 ========
NUC1                   1H
P1                   8.90 usec
PLW1          26.00000000 W
SFO1          500.1330885 MHz

F2 - Processing parameters
SI                  65536
SF            500.1300115 MHz
WDW                    EM
SSB      0
LB                   0.30 Hz
GB       0
PC                   1.00
```

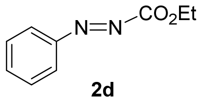

2d

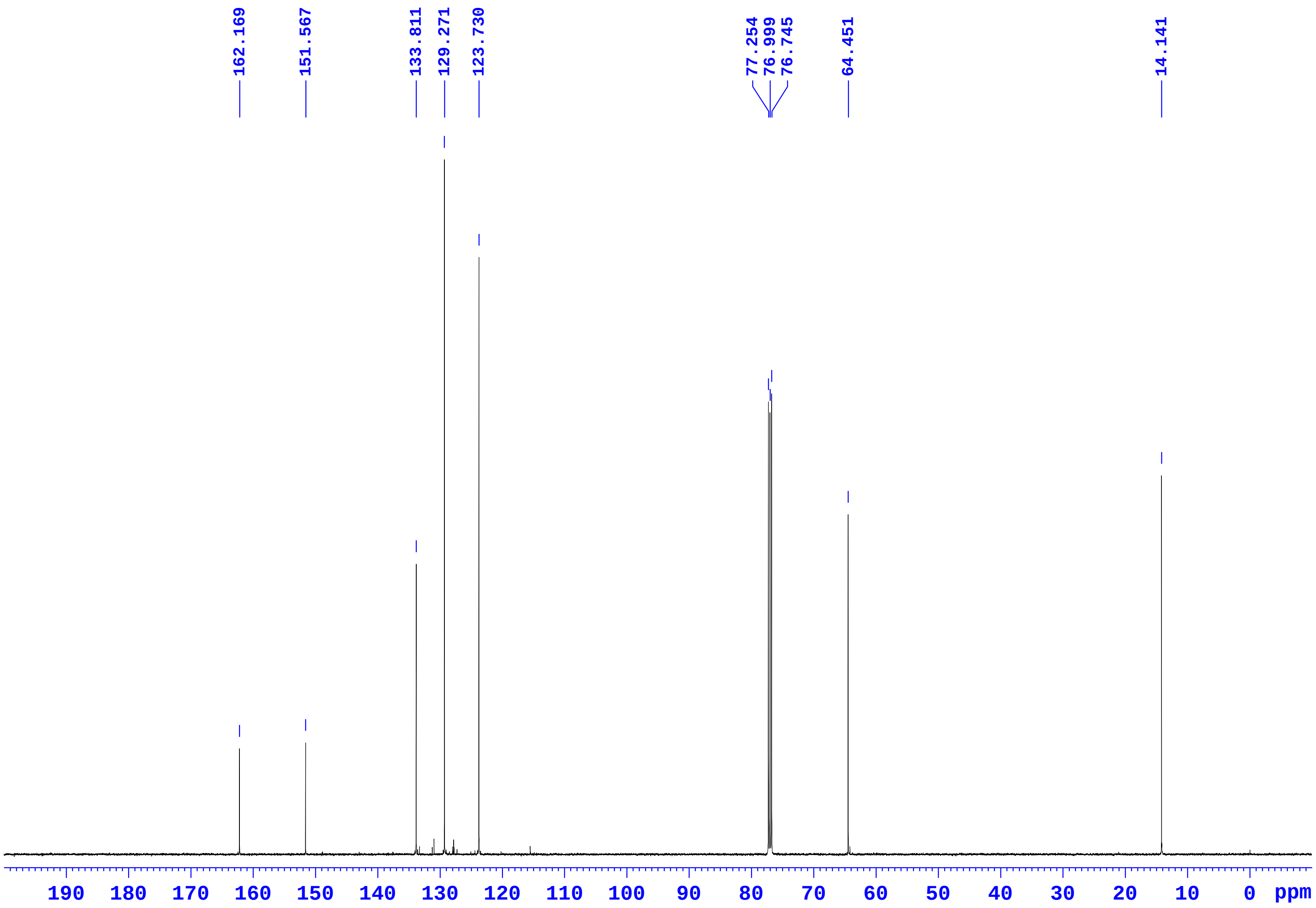


```
Current Data Parameters
NAME              MGS-661
EXPNO                   3
PROCNO                  1

F2 - Acquisition Parameters
Date_            20151103
Time                 0.58
INSTRUM             spect
PROBHD   5 mm PABBO BB-
PULPROG           zgpg30
TD                 65536
SOLVENT            CDCl3
NS                  3072
DS                     4
SWH            29761.904 Hz
FIDRES          0.454131 Hz
AQ             1.1010548 sec
RG                  2050
DW                16.800 usec
DE                  6.50 usec
TE                 296.0 K
D1            1.00000000 sec
D11           0.03000000 sec

======== CHANNEL f1 ========
NUC1                 13C
P1                  9.00 usec
PLW1        122.00000000 W
SFO1         125.7703637 MHz

======== CHANNEL f2 ========
CPDPRG2          waltz16
NUC2                  1H
PCPD2              80.00 usec
PLW2         26.00000000 W
PLW12         0.32179001 W
PLW13         0.20595001 W
SFO2         500.1320005 MHz

F2 - Processing parameters
SI                 32768
SF           125.7577957 MHz
WDW                   EM
SSB      0
LB                  1.00 Hz
GB       0
PC                  1.40
```

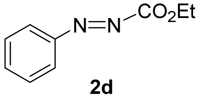

2d

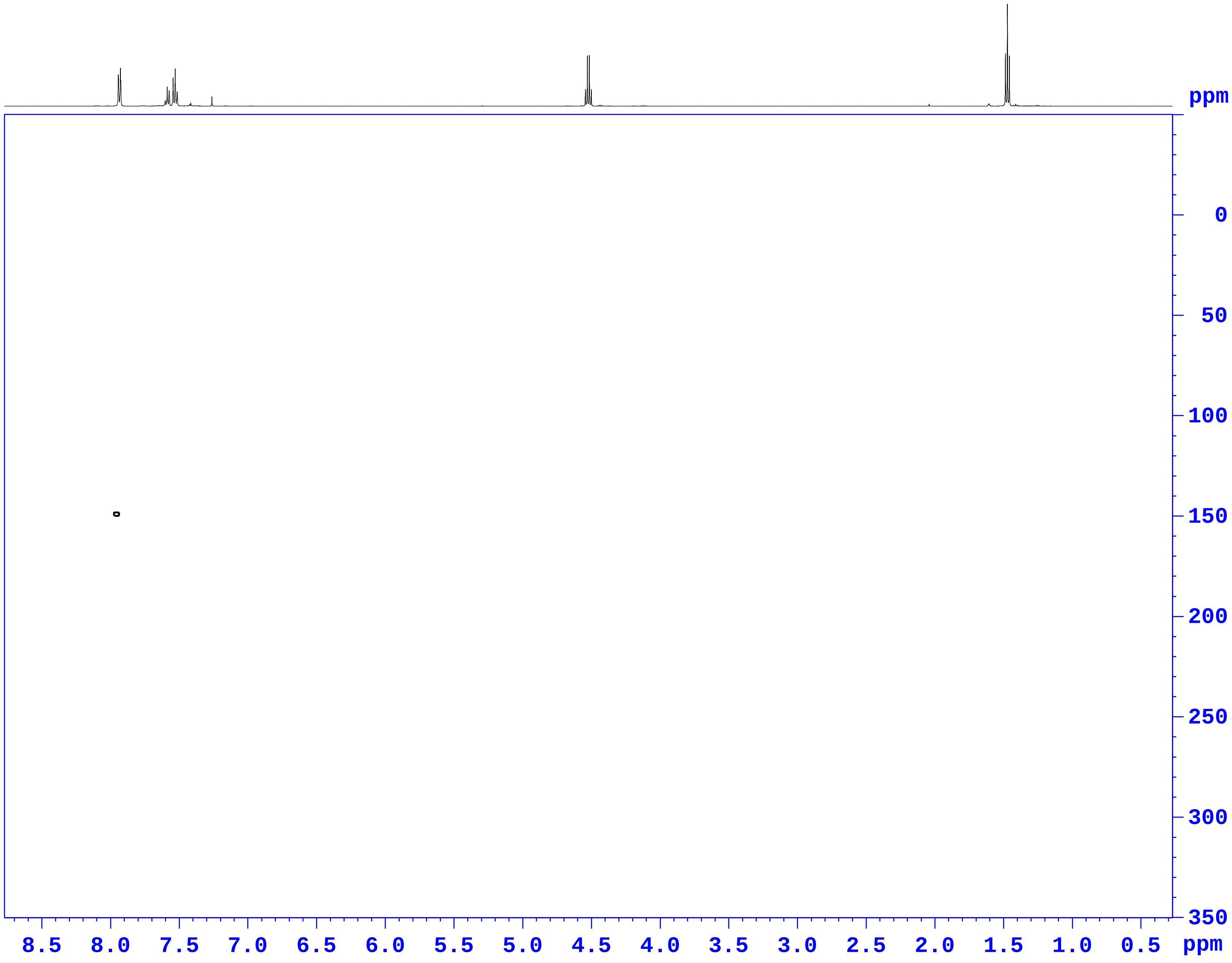


```
Current Data Parameters
NAME              MGS-472
EXPNO                  23
PROCNO                  1

F2 - Acquisition Parameters
Date_            20150222
Time                22.31
INSTRUM             spect
PROBHD    5 mm PABBO BB-
PULPROG        hmbcgpndqf
TD                   2048
SOLVENT             CDCl3
NS                      8
DS                     16
SWH              4464.286 Hz
FIDRES           2.179827 Hz
AQ              0.2294260 sec
RG                   2050
DW                112.000 usec
DE                   6.50 usec
TE                  296.0 K
CNST13          5.0000000
D0             0.00000300 sec
D1             1.92790997 sec
D6             0.10000000 sec
D16            0.00020000 sec
IN0            0.00002465 sec

======== CHANNEL f1 ========
NUC1                   1H
P1                   8.90 usec
P2                  17.80 usec
PLW1          26.00000000 W
SFO1          500.1323675 MHz

======== CHANNEL f2 ========
NUC2                  15N
P3                  14.40 usec
PLW2         206.00000000 W
SFO2           50.6853342 MHz

====== GRADIENT CHANNEL =====
GPNAM1         SMSQ10.100
GPNAM2         SMSQ10.100
GPNAM3         SMSQ10.100
GPZ1                70.00 %
GPZ2                30.00 %
GPZ3                50.10 %
P16               1000.00 usec

F1 - Acquisition parameters
TD                    256
SFO1             50.68533 MHz
FIDRES          79.195831 Hz
SW                400.000 ppm
FnMODE                 QF

F2 - Processing parameters
SI                   2048
SF            500.1300000 MHz
WDW                  SINE
SSB       0
LB        0 Hz
GB        0
PC                   1.40

F1 - Processing parameters
SI                   1024
MC2                    QF
SF             50.6777330 MHz
WDW          echo-antiecho
SSB       0
LB        0 Hz
GB        0
```

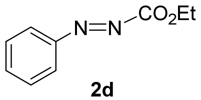

2d

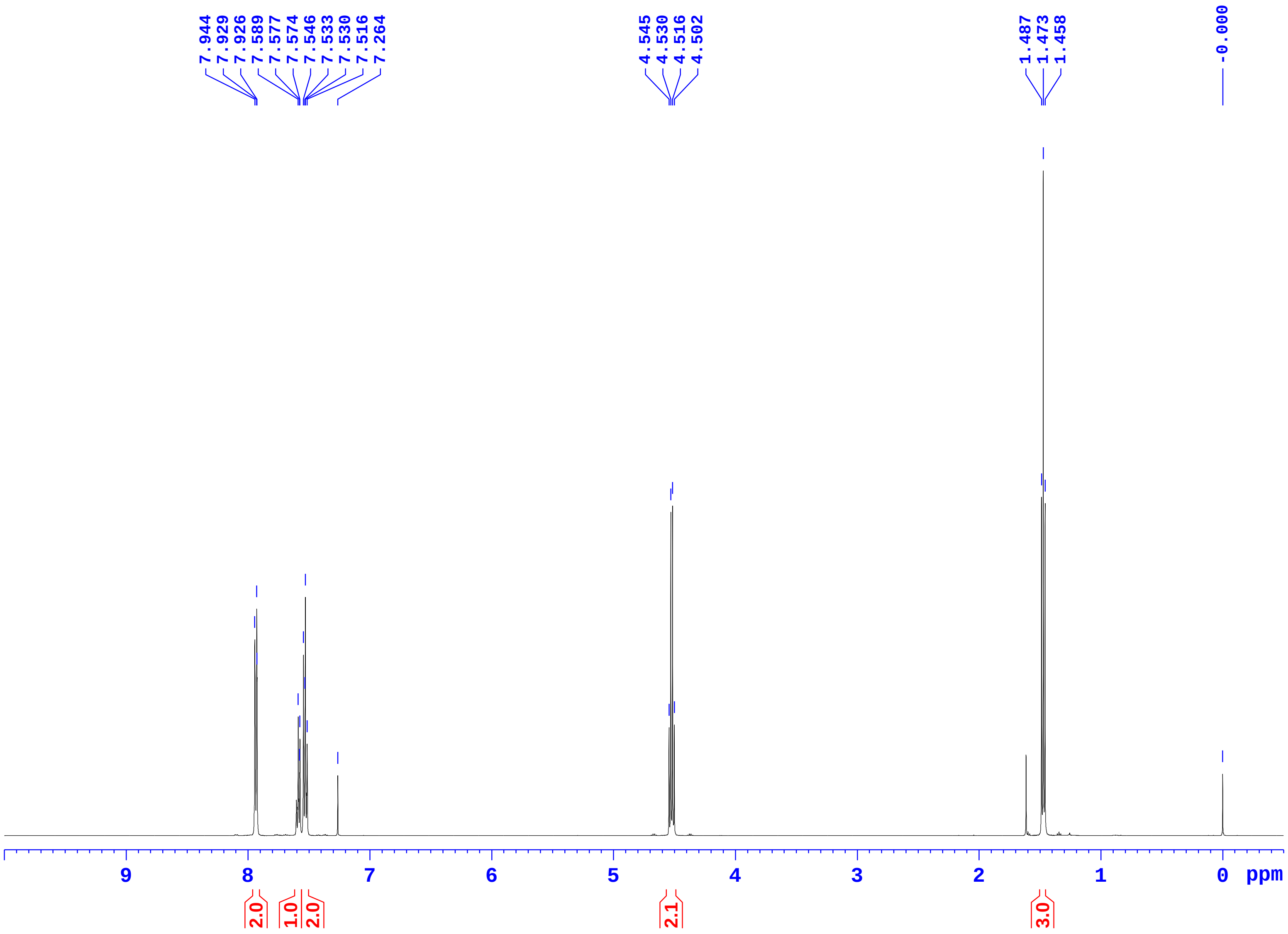

Current Data Parameters
NAME MGS-645
EXPNO 20
PROCNO 1

F2 - Acquisition Parameters
Date_ 20151014
Time 1.42
INSTRUM spect
PROBHD 5 mm PABBO BB-
PULPROG zg30
TD 65536
SOLVENT CDCl3
NS 16
DS 2
SWH 10330.578 Hz
FIDRES 0.157632 Hz
AQ 3.1719923 sec
RG 80.6
DW 48.400 usec
DE 6.50 usec
TE 296.3 K
D1 1.00000000 sec

======== CHANNEL f1 ========
NUC1 1H
P1 8.90 usec
PLW1 26.00000000 W
SFO1 500.1330885 MHz

F2 - Processing parameters
SI 65536
SF 500.1300116 MHz
WDW EM
SSB 0
LB 0.30 Hz
GB 0
PC 1.00

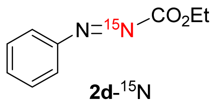

2d-15N

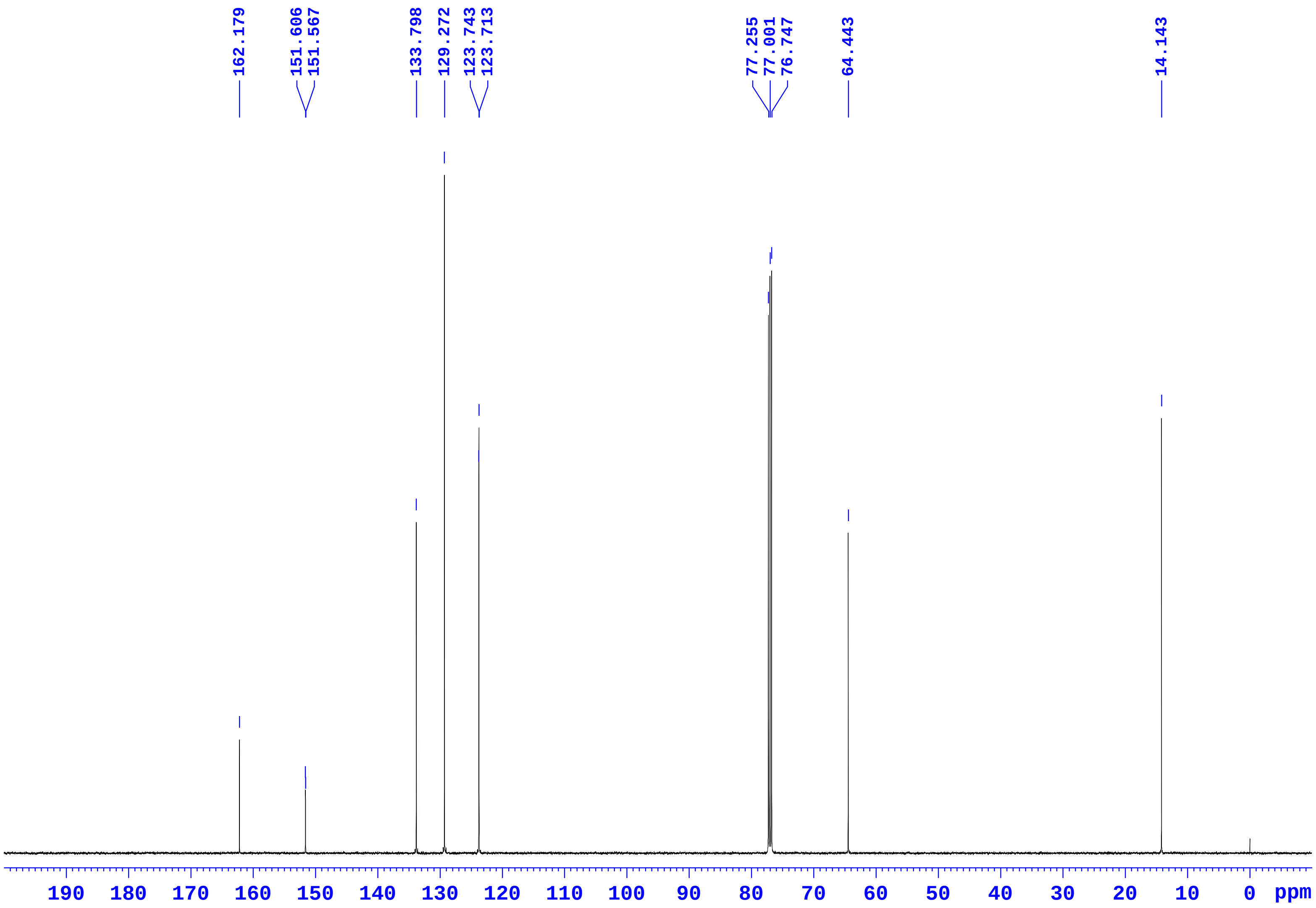


```
Current Data Parameters
NAME              MGS-645
EXPNO                  21
PROCNO                  1

F2 - Acquisition Parameters
Date_            20151014
Time                 4.11
INSTRUM             spect
PROBHD   5 mm PABBO BB-
PULPROG           zgpg30
TD                  65536
SOLVENT             CDCl3
NS                   4096
DS                      4
SWH             29761.904 Hz
FIDRES           0.454131 Hz
AQ              1.1010548 sec
RG                   2050
DW                 16.800 usec
DE                   6.50 usec
TE                  297.2 K
D1             1.00000000 sec
D11            0.03000000 sec

======== CHANNEL f1 ========
NUC1                  13C
P1                   9.00 usec
PLW1         122.00000000 W
SFO1          125.7703637 MHz

======== CHANNEL f2 ========
CPDPRG2           waltz16
NUC2                   1H
PCPD2               80.00 usec
PLW2          26.00000000 W
PLW12          0.32179001 W
PLW13          0.20595001 W
SFO2          500.1320005 MHz

F2 - Processing parameters
SI                  32768
SF            125.7577945 MHz
WDW                    EM
SSB       0
LB                   1.00 Hz
GB        0
PC                   1.40
```

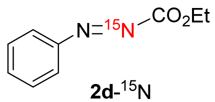

2d-^{15}N

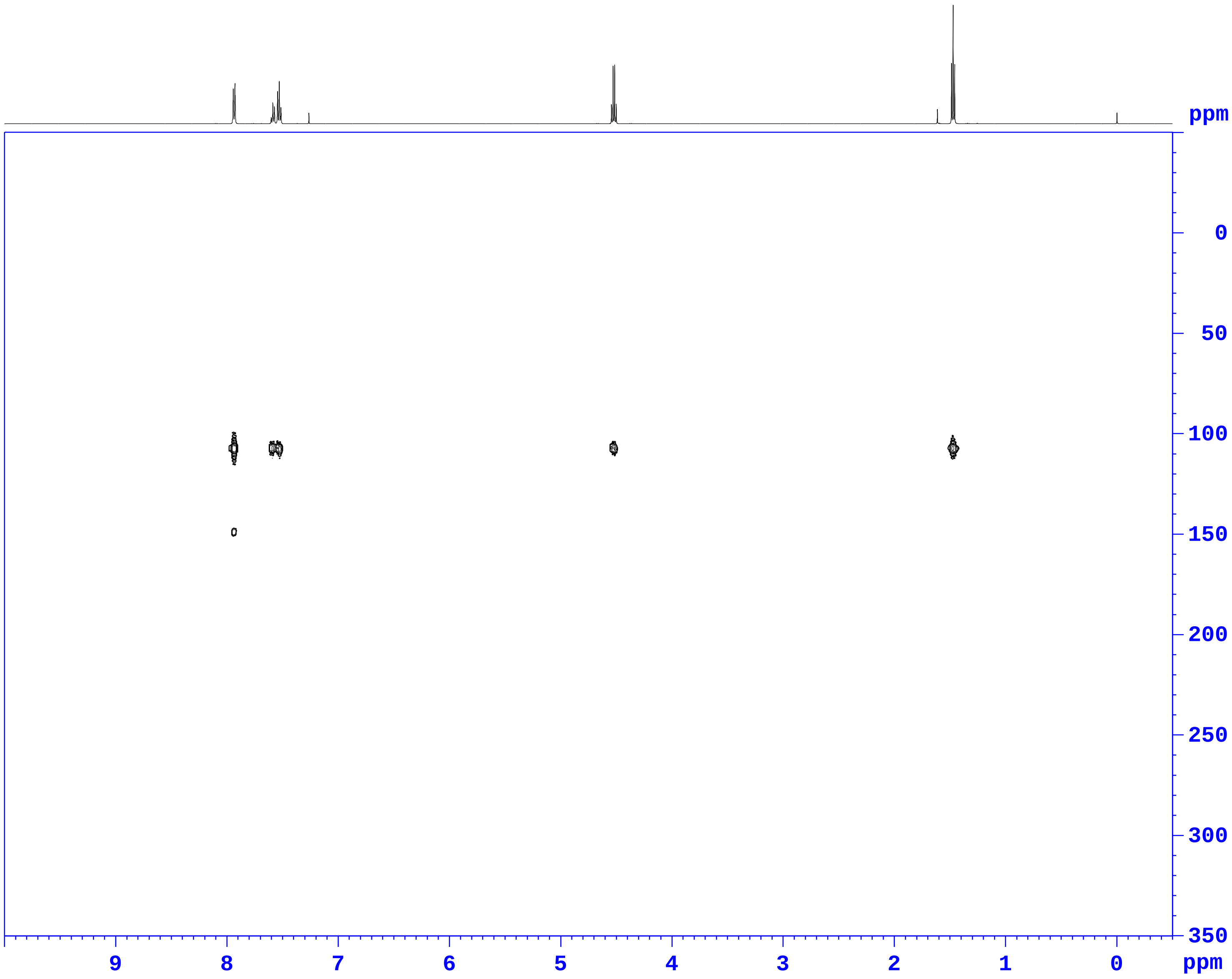


```
Current Data Parameters
NAME              MGS-645
EXPNO                  25
PROCNO                  1

F2 - Acquisition Parameters
Date_            20151014
Time                 5.54
INSTRUM             spect
PROBHD   5 mm PABBO BB-
PULPROG       hmbcgpndqf
TD                   2048
SOLVENT             CDCl3
NS                     32
DS                     16
SWH              4716.981 Hz
FIDRES           2.303213 Hz
AQ              0.2171380 sec
RG                   2050
DW                106.000 usec
DE                   6.50 usec
TE                  296.1 K
CNST13          5.0000000
D0             0.00000300 sec
D1             1.94019794 sec
D6             0.10000000 sec
D16            0.00020000 sec
IN0            0.00002465 sec

======== CHANNEL f1 ========
NUC1                   1H
P1                   8.90 usec
P2                  17.80 usec
PLW1          26.00000000 W
SFO1          500.1320253 MHz

======== CHANNEL f2 ========
NUC2                  15N
P3                  14.40 usec
PLW2         206.00000000 W
SFO2           50.6853342 MHz

====== GRADIENT CHANNEL =====
GPNAM1         SMSQ10.100
GPNAM2         SMSQ10.100
GPNAM3         SMSQ10.100
GPZ1                70.00 %
GPZ2                30.00 %
GPZ3                50.10 %
P16               1000.00 usec

F1 - Acquisition parameters
TD                    128
SFO1             50.68533 MHz
FIDRES         158.391663 Hz
SW                400.000 ppm
FnMODE                 QF

F2 - Processing parameters
SI                   2048
SF            500.1300116 MHz
WDW                  SINE
SSB      0
LB       0 Hz
GB       0
PC                   1.40

F1 - Processing parameters
SI                   1024
MC2                    QF
SF             50.6777330 MHz
WDW                States
SSB      0
LB       0 Hz
GB       0
```

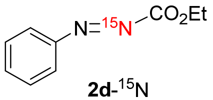

2d-^{15}N

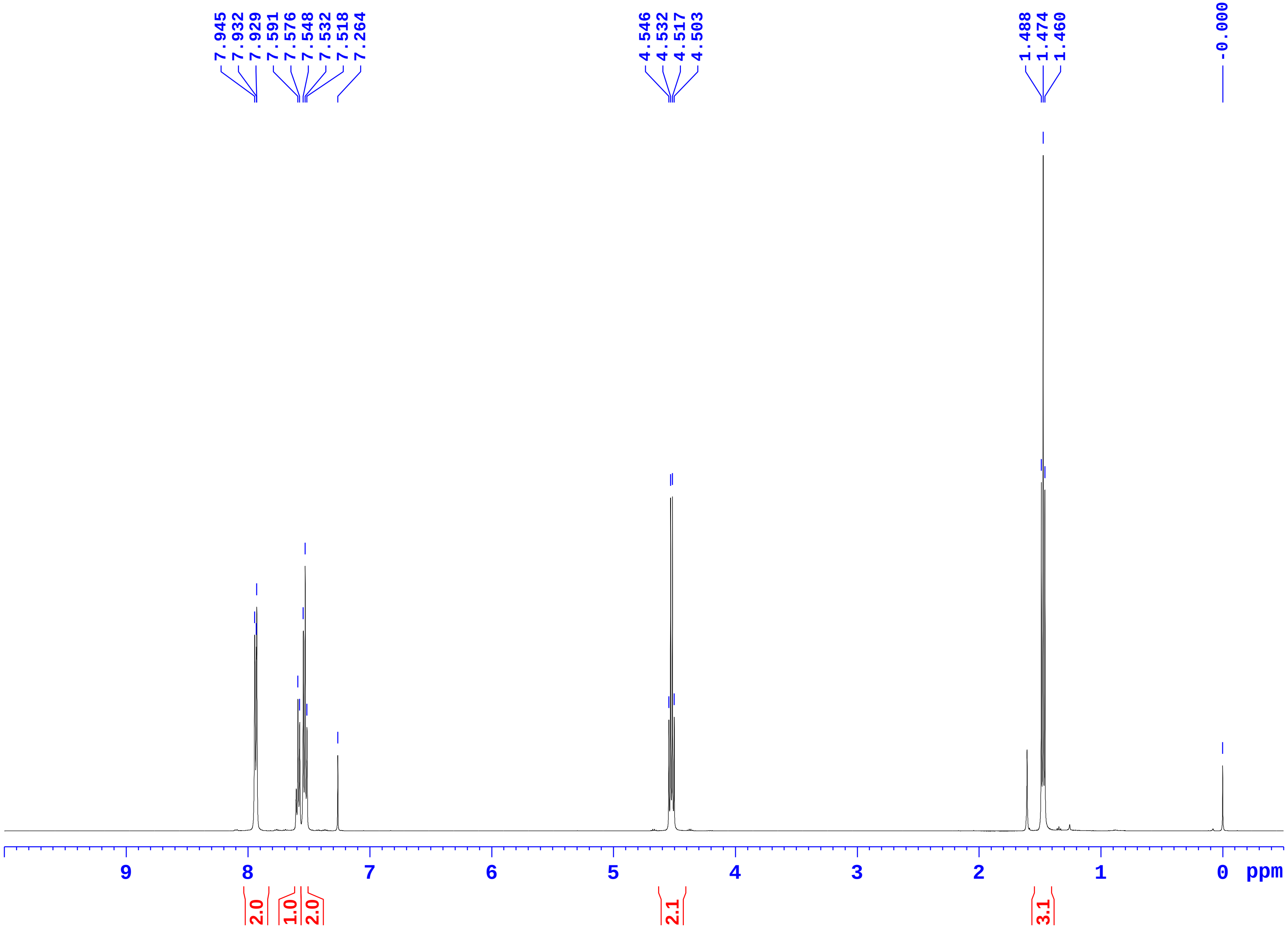


```
Current Data Parameters
NAME              MGS-646
EXPNO                   1
PROCNO                  1

F2 - Acquisition Parameters
Date_            20151017
Time                19.40
INSTRUM             spect
PROBHD   5 mm PABBO BB-
PULPROG              zg30
TD                  65536
SOLVENT             CDCl3
NS                     16
DS                      2
SWH             10330.578 Hz
FIDRES           0.157632 Hz
AQ              3.1719923 sec
RG                    101
DW                 48.400 usec
DE                   6.50 usec
TE                  296.0 K
D1             1.00000000 sec

======== CHANNEL f1 ========
NUC1                   1H
P1                   8.90 usec
PLW1          26.00000000 W
SFO1          500.1330885 MHz

F2 - Processing parameters
SI                  65536
SF            500.1300119 MHz
WDW                    EM
SSB      0
LB                   0.30 Hz
GB       0
PC                   1.00
```

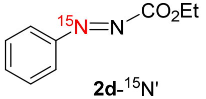

2d-^{15}N'

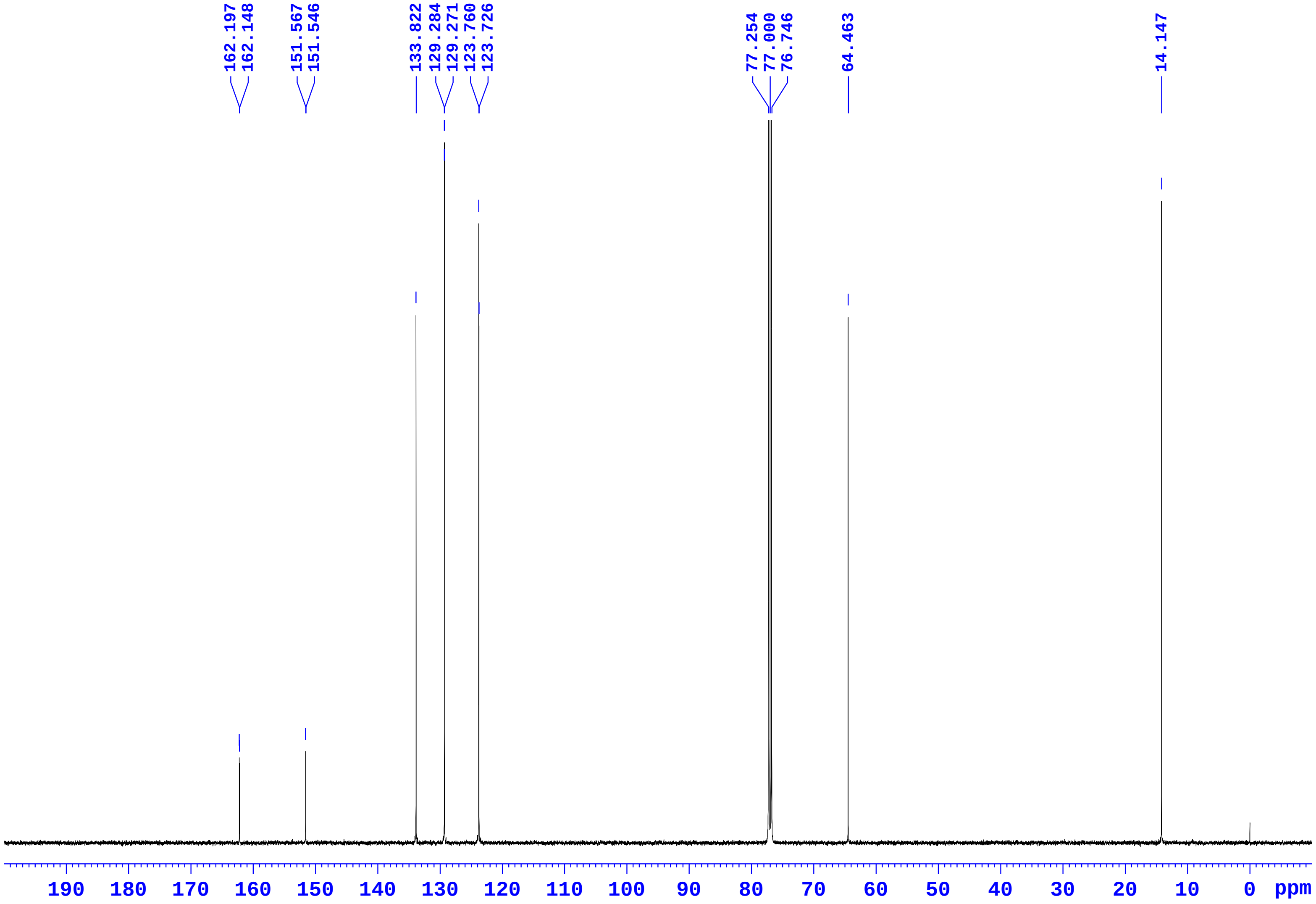


```
Current Data Parameters
NAME              MGS-646
EXPNO                   4
PROCNO                  1

F2 - Acquisition Parameters
Date_            20151017
Time                22.15
INSTRUM             spect
PROBHD    5 mm PABBO BB-
PULPROG           zgpg30
TD                 65536
SOLVENT            CDCl3
NS                  4096
DS                     4
SWH            29761.904 Hz
FIDRES          0.454131 Hz
AQ             1.1010548 sec
RG                  2050
DW                16.800 usec
DE                  6.50 usec
TE                 296.0 K
D1            1.00000000 sec
D11           0.03000000 sec

======== CHANNEL f1 ========
NUC1                 13C
P1                  9.00 usec
PLW1        122.00000000 W
SFO1         125.7703637 MHz

======== CHANNEL f2 ========
CPDPRG2          waltz16
NUC2                  1H
PCPD2              80.00 usec
PLW2         26.00000000 W
PLW12         0.32179001 W
PLW13         0.20595001 W
SFO2         500.1320005 MHz

F2 - Processing parameters
SI                 32768
SF           125.7577949 MHz
WDW                   EM
SSB       0
LB                  1.00 Hz
GB        0
PC                  1.40
```

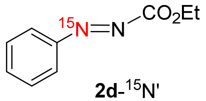

2d-^{15}N'

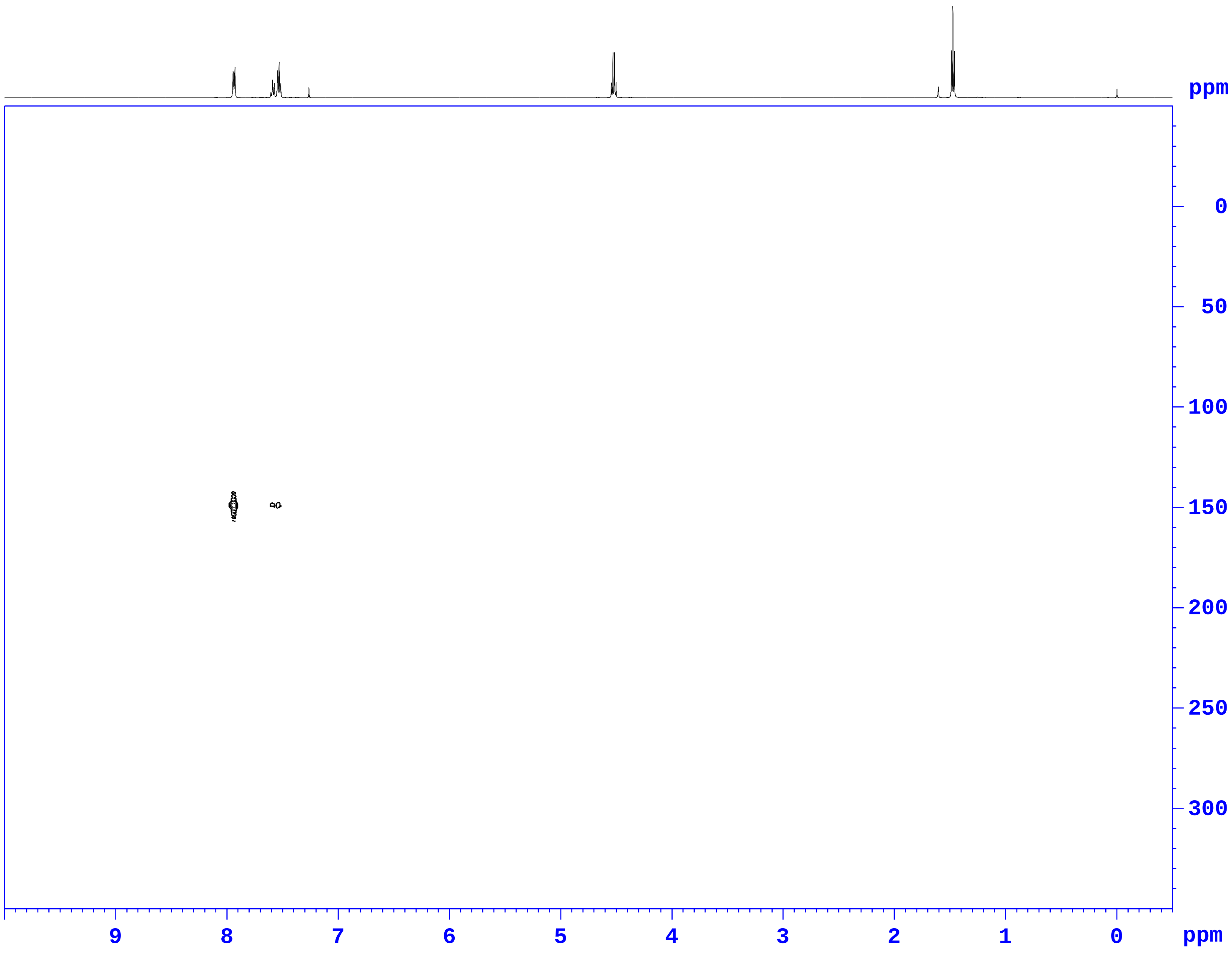


```
Current Data Parameters
NAME               MGS-646
EXPNO                    7
PROCNO                   1

F2 - Acquisition Parameters
Date_             20151017
Time                 23.33
INSTRUM              spect
PROBHD   5 mm PABBO BB-
PULPROG        hmbcgpndqf
TD                    2048
SOLVENT              CDCl3
NS                      16
DS                      16
SWH               4761.905 Hz
FIDRES            2.325149 Hz
AQ               0.2150900 sec
RG                    2050
DW                 105.000 usec
DE                    6.50 usec
TE                   296.0 K
CNST13           5.0000000
D0              0.00000300 sec
D1              1.94224596 sec
D6              0.10000000 sec
D16             0.00020000 sec
IN0             0.00002465 sec

======== CHANNEL f1 ========
NUC1                    1H
P1                    8.90 usec
P2                   17.80 usec
PLW1           26.00000000 W
SFO1           500.1320412 MHz

======== CHANNEL f2 ========
NUC2                   15N
P3                   14.40 usec
PLW2          206.00000000 W
SFO2            50.6853342 MHz

====== GRADIENT CHANNEL =====
GPNAM1          SMSQ10.100
GPNAM2          SMSQ10.100
GPNAM3          SMSQ10.100
GPZ1                 70.00 %
GPZ2                 30.00 %
GPZ3                 50.10 %
P16                1000.00 usec

F1 - Acquisition parameters
TD                     128
SFO1              50.68533 MHz
FIDRES          158.391663 Hz
SW                 400.000 ppm
FnMODE                  QF

F2 - Processing parameters
SI                    2048
SF             500.1300119 MHz
WDW                   SINE
SSB       0
LB        0 Hz
GB        0
PC                    1.40

F1 - Processing parameters
SI                    1024
MC2                     QF
SF              50.6777330 MHz
WDW           echo-antiecho
SSB       0
LB        0 Hz
GB        0
```

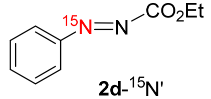

2d-^{15}N'

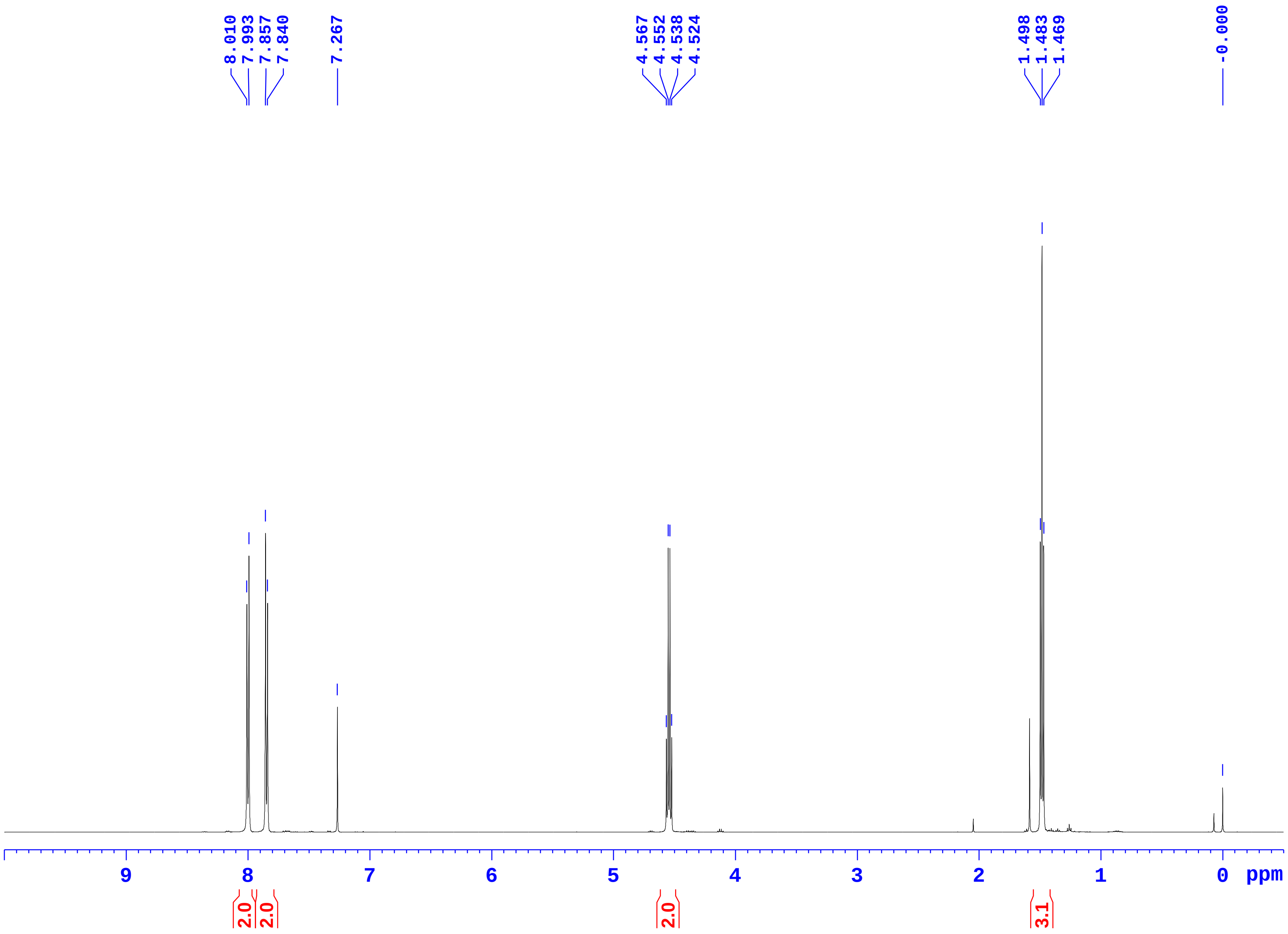


```
Current Data Parameters
NAME               MGS-650
EXPNO                    1
PROCNO                   1

F2 - Acquisition Parameters
Date_             20151022
Time                  0.17
INSTRUM              spect
PROBHD    5 mm PABBO BB-
PULPROG               zg30
TD                   65536
SOLVENT              CDCl3
NS                      16
DS                       2
SWH             10330.578 Hz
FIDRES           0.157632 Hz
AQ              3.1719923 sec
RG                     144
DW                  48.400 usec
DE                    6.50 usec
TE                   296.0 K
D1              1.00000000 sec

======== CHANNEL f1 ========
NUC1                    1H
P1                    8.90 usec
PLW1           26.00000000 W
SFO1           500.1330885 MHz

F2 - Processing parameters
SI                   65536
SF             500.1300102 MHz
WDW                     EM
SSB      0
LB                    0.30 Hz
GB       0
PC                    1.00
```

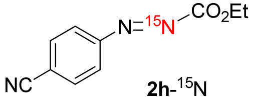

2h-^{15}N

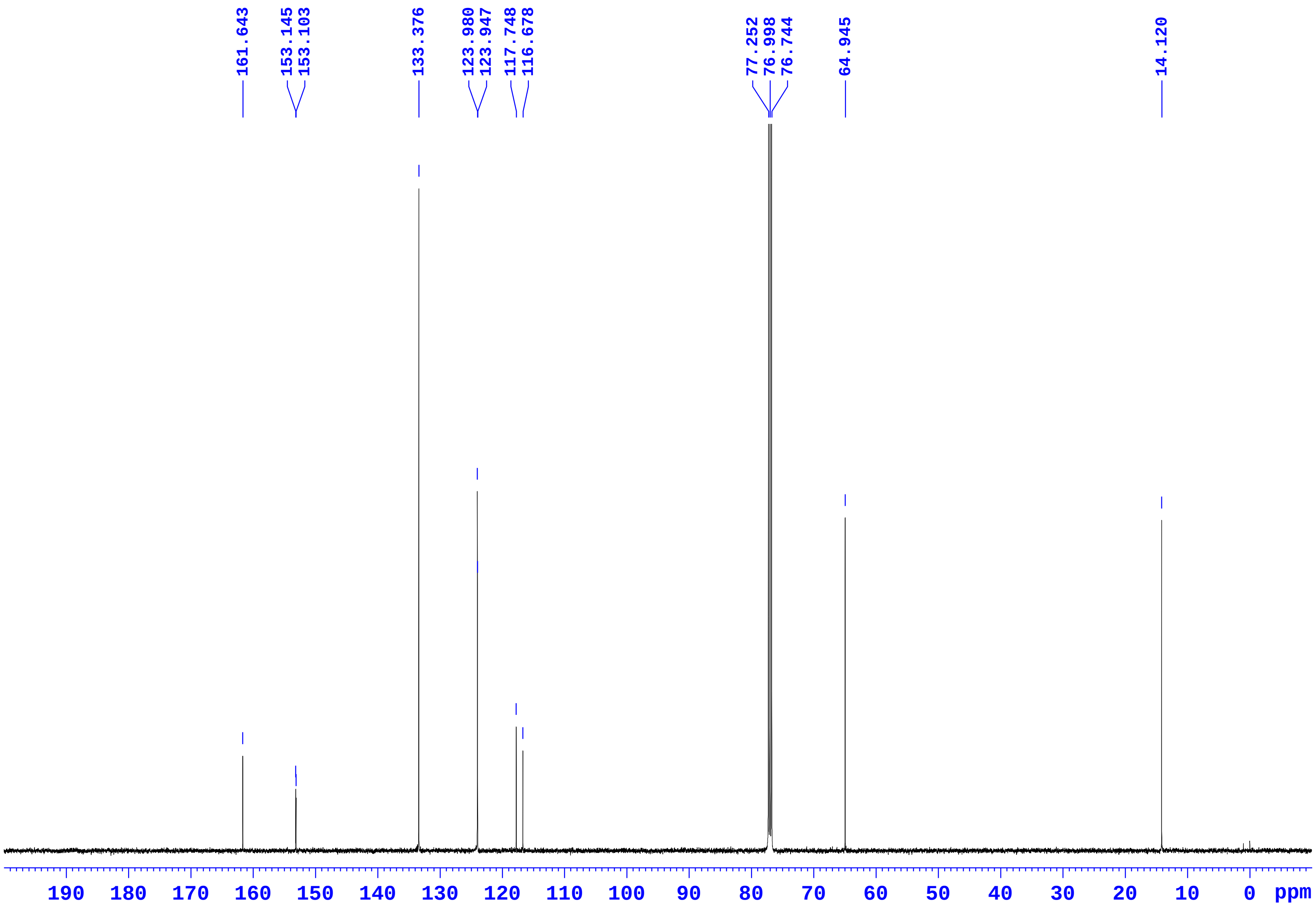


```
Current Data Parameters
NAME              MGS-650
EXPNO                   3
PROCNO                  1

F2 - Acquisition Parameters
Date_            20151022
Time                 2.15
INSTRUM             spect
PROBHD   5 mm PABBO BB-
PULPROG           zgpg30
TD                 65536
SOLVENT            CDCl3
NS                  3072
DS                     4
SWH            29761.904 Hz
FIDRES          0.454131 Hz
AQ             1.1010548 sec
RG                  2050
DW                16.800 usec
DE                  6.50 usec
TE                 296.0 K
D1            1.00000000 sec
D11           0.03000000 sec

======== CHANNEL f1 ========
NUC1                 13C
P1                  9.00 usec
PLW1        122.00000000 W
SFO1         125.7703637 MHz

======== CHANNEL f2 ========
CPDPRG2          waltz16
NUC2                  1H
PCPD2              80.00 usec
PLW2         26.00000000 W
PLW12         0.32179001 W
PLW13         0.20595001 W
SFO2         500.1320005 MHz

F2 - Processing parameters
SI                 32768
SF           125.7577945 MHz
WDW                   EM
SSB      0
LB                  1.00 Hz
GB       0
PC                  1.40
```

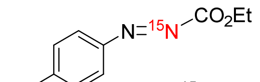

2h-^{15}N

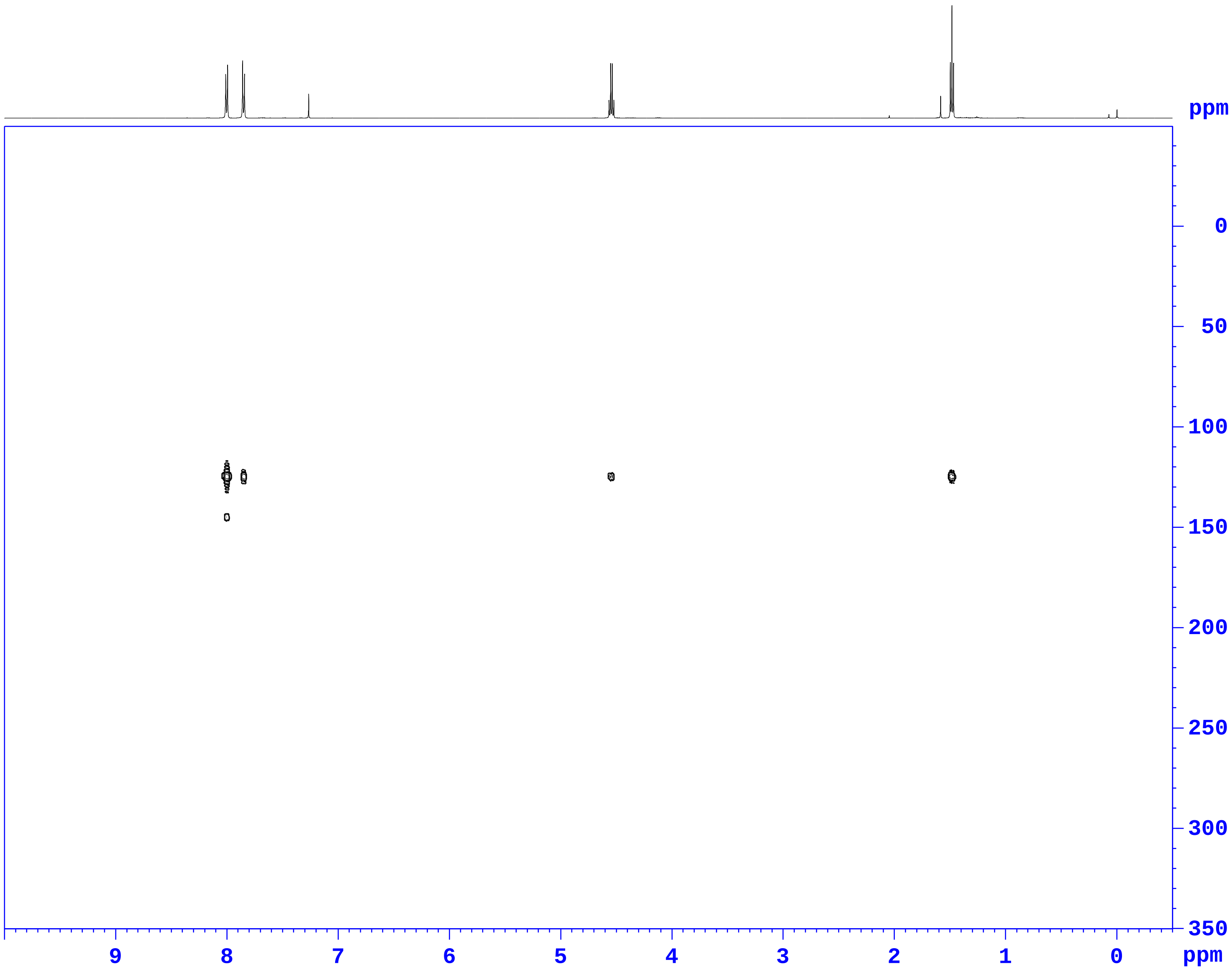


```
Current Data Parameters
NAME              MGS-650
EXPNO                   6
PROCNO                  1

F2 - Acquisition Parameters
Date_            20151022
Time                 3.04
INSTRUM             spect
PROBHD    5 mm PABBO BB-
PULPROG       hmbcgpndqf
TD                   2048
SOLVENT             CDCl3
NS                     16
DS                     16
SWH              4761.905 Hz
FIDRES           2.325149 Hz
AQ              0.2150900 sec
RG                   2050
DW                105.000 usec
DE                   6.50 usec
TE                  296.0 K
CNST13          5.0000000
D0             0.00000300 sec
D1             1.94224596 sec
D6             0.10000000 sec
D16            0.00020000 sec
IN0            0.00002465 sec

======== CHANNEL f1 ========
NUC1                   1H
P1                   8.90 usec
P2                  17.80 usec
PLW1          26.00000000 W
SFO1          500.1320605 MHz

======== CHANNEL f2 ========
NUC2                  15N
P3                  14.40 usec
PLW2         206.00000000 W
SFO2           50.6853342 MHz

====== GRADIENT CHANNEL =====
GPNAM1         SMSQ10.100
GPNAM2         SMSQ10.100
GPNAM3         SMSQ10.100
GPZ1                70.00 %
GPZ2                30.00 %
GPZ3                50.10 %
P16               1000.00 usec

F1 - Acquisition parameters
TD                    128
SFO1             50.68533 MHz
FIDRES         158.391663 Hz
SW                400.000 ppm
FnMODE                 QF

F2 - Processing parameters
SI                   2048
SF            500.1300102 MHz
WDW                  SINE
SSB      0
LB       0 Hz
GB       0
PC                   1.40

F1 - Processing parameters
SI                   1024
MC2                    QF
SF             50.6777330 MHz
WDW          echo-antiecho
SSB      0
LB       0 Hz
GB       0
```

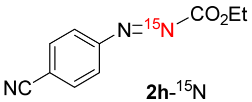

2h-^{15}N

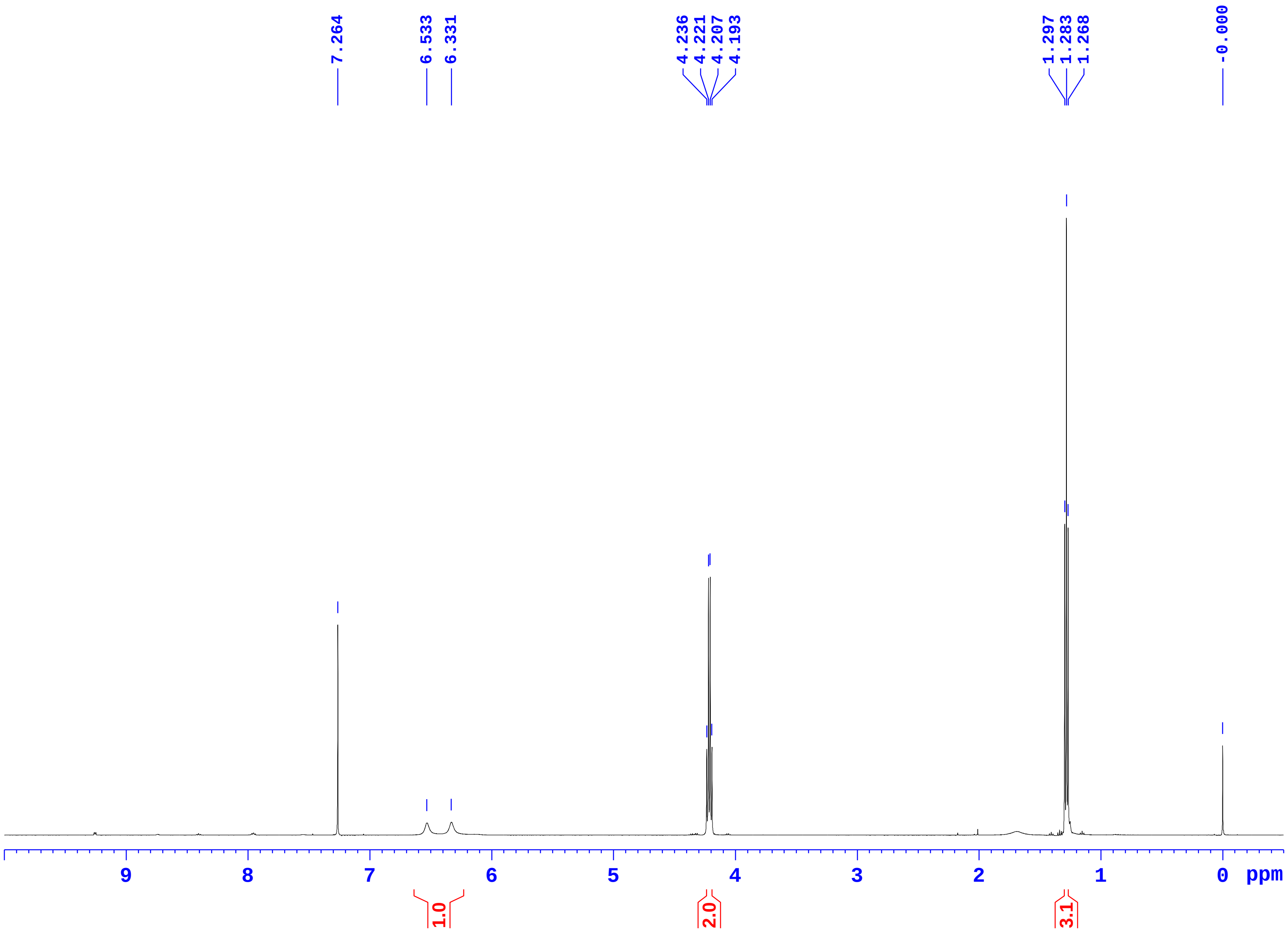


```
Current Data Parameters
NAME              MGS-657
EXPNO                   1
PROCNO                  1

F2 - Acquisition Parameters
Date_            20151028
Time                 6.00
INSTRUM             spect
PROBHD   5 mm PABBO BB-
PULPROG              zg30
TD                  65536
SOLVENT             CDCl3
NS                     16
DS                      2
SWH             10330.578 Hz
FIDRES           0.157632 Hz
AQ              3.1719923 sec
RG                    161
DW                 48.400 usec
DE                   6.50 usec
TE                  296.0 K
D1             1.00000000 sec

======== CHANNEL f1 ========
NUC1                   1H
P1                   8.90 usec
PLW1          26.00000000 W
SFO1          500.1330885 MHz

F2 - Processing parameters
SI                  65536
SF            500.1300115 MHz
WDW                    EM
SSB       0
LB                   0.30 Hz
GB        0
PC                   1.00
```

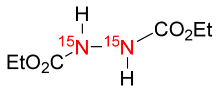

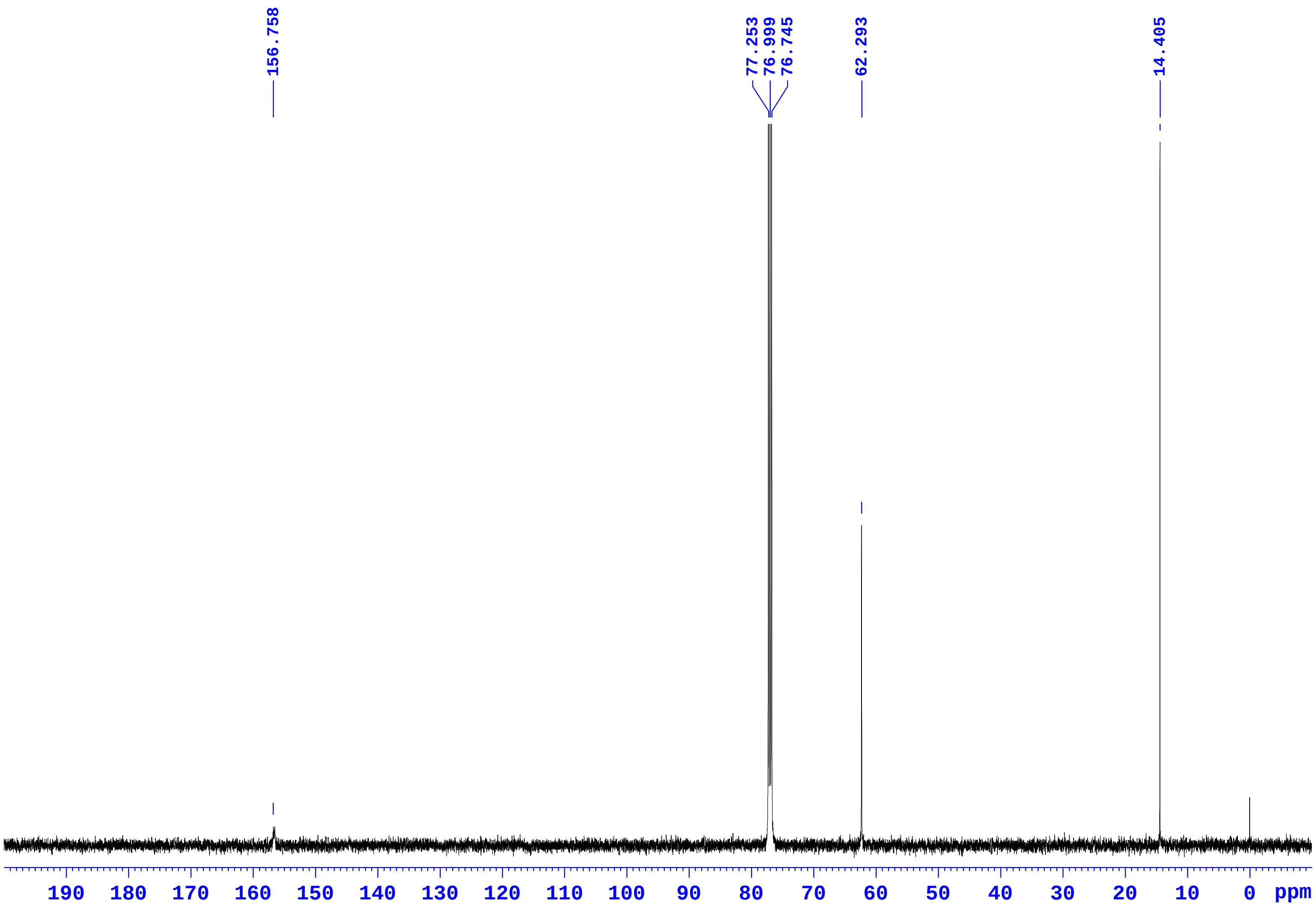

Current Data Parameters
NAME MGS-657
EXPNO 30
PROCNO 1

F2 - Acquisition Parameters
Date_ 20151102
Time 2.32
INSTRUM spect
PROBHD 5 mm PABBO BB-
PULPROG zgpg30
TD 65536
SOLVENT CDCl3
NS 10240
DS 4
SWH 29761.904 Hz
FIDRES 0.454131 Hz
AQ 1.1010548 sec
RG 2050
DW 16.800 usec
DE 6.50 usec
TE 296.0 K
D1 1.00000000 sec
D11 0.03000000 sec

======== CHANNEL f1 ========
NUC1 13C
P1 9.00 usec
PLW1 122.00000000 W
SFO1 125.7703637 MHz

======== CHANNEL f2 ========
CPDPRG2 waltz16
NUC2 1H
PCPD2 80.00 usec
PLW2 26.00000000 W
PLW12 0.32179001 W
PLW13 0.20595001 W
SFO2 500.1320005 MHz

F2 - Processing parameters
SI 32768
SF 125.7577920 MHz
WDW EM
SSB 0
LB 1.00 Hz
GB 0
PC 1.40

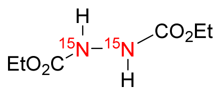

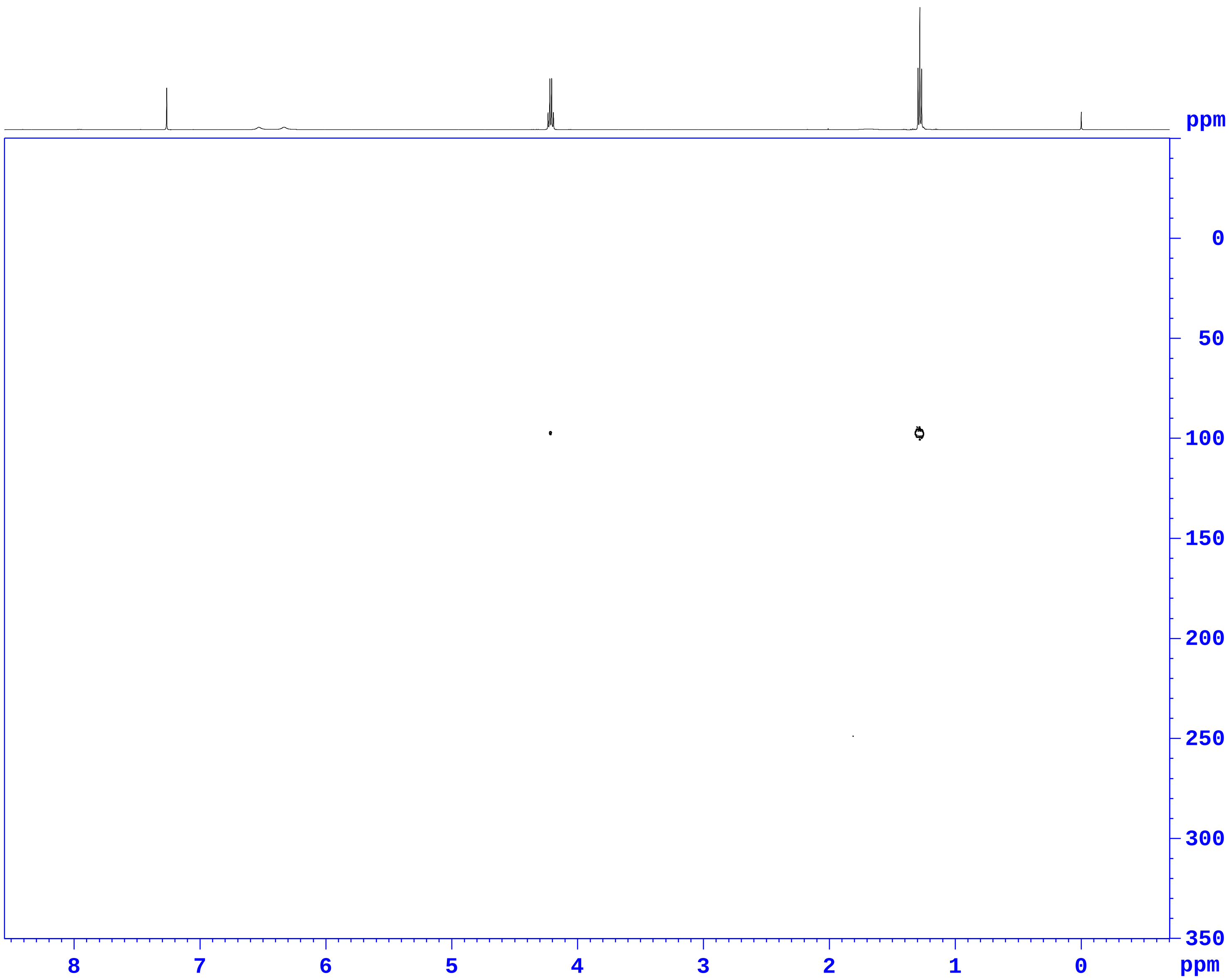

Current Data Parameters
NAME MGS-657
EXPNO 3
PROCNO 1

F2 - Acquisition Parameters
Date_ 20151028
Time 7.18
INSTRUM spect
PROBHD 5 mm PABBO BB-
PULPROG hmbcgpndqf
TD 2048
SOLVENT CDCl3
NS 8
DS 16
SWH 4629.629 Hz
FIDRES 2.260561 Hz
AQ 0.2212340 sec
RG 2050
DW 108.000 usec
DE 6.50 usec
TE 296.0 K
CNST13 5.0000000
D0 0.00000300 sec
D1 1.93610203 sec
D6 0.10000000 sec
D16 0.00020000 sec
IN0 0.00002465 sec

======== CHANNEL f1 ========
NUC1 1H
P1 8.90 usec
P2 17.80 usec
PLW1 26.00000000 W
SFO1 500.1319747 MHz

======== CHANNEL f2 ========
NUC2 15N
P3 14.40 usec
PLW2 206.00000000 W
SFO2 50.6853342 MHz

====== GRADIENT CHANNEL =====
GPNAM1 SMSQ10.100
GPNAM2 SMSQ10.100
GPNAM3 SMSQ10.100
GPZ1 70.00 %
GPZ2 30.00 %
GPZ3 50.10 %
P16 1000.00 usec

F1 - Acquisition parameters
TD 256
SFO1 50.68533 MHz
FIDRES 79.195831 Hz
SW 400.000 ppm
FnMODE QF

F2 - Processing parameters
SI 2048
SF 500.1300115 MHz
WDW SINE
SSB 0
LB 0 Hz
GB 0
PC 1.40

F1 - Processing parameters
SI 1024
MC2 QF
SF 50.6777330 MHz
WDW echo-antiecho
SSB 0
LB 0 Hz
GB 0

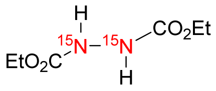

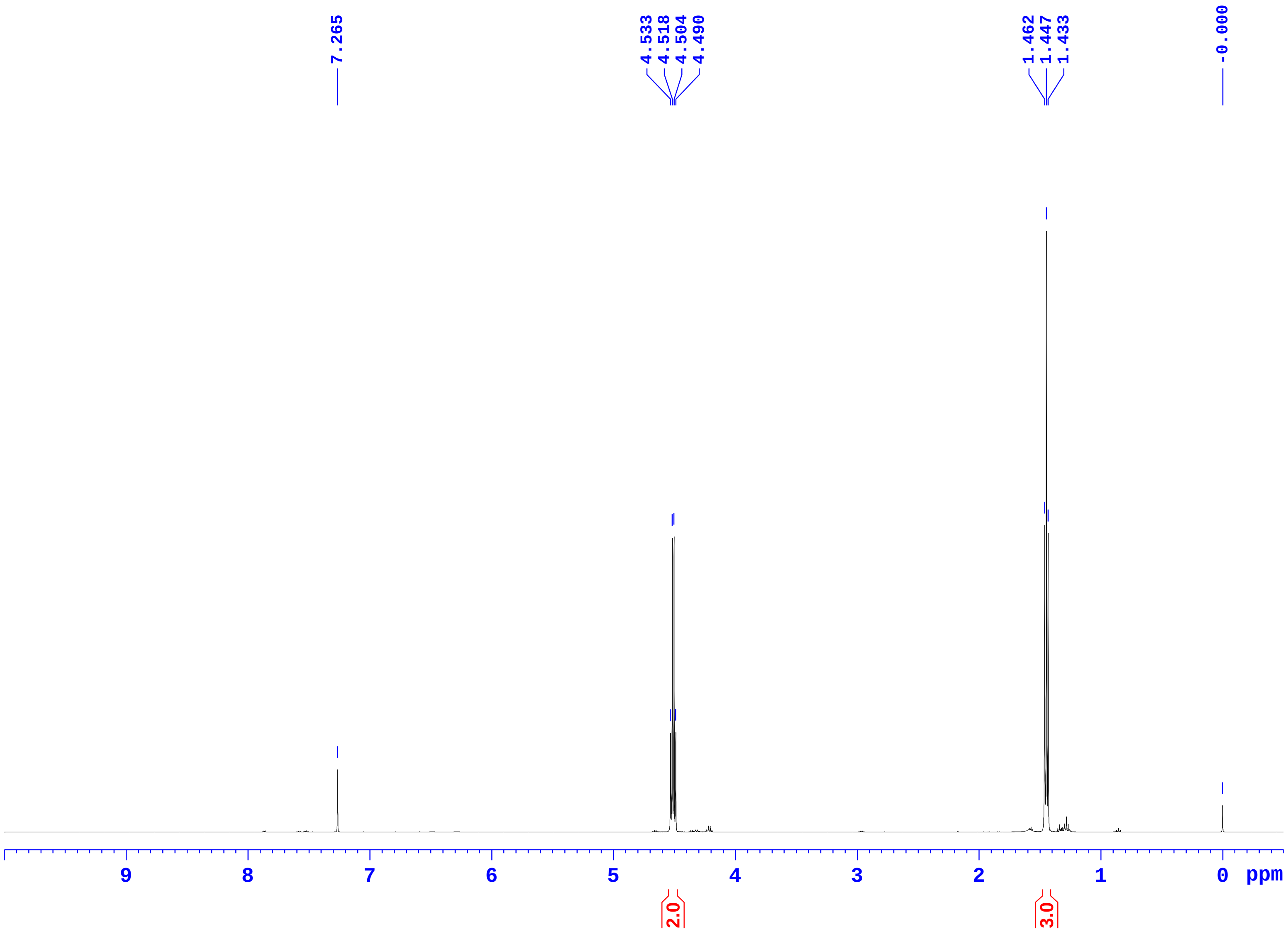


```
Current Data Parameters
NAME              MGS-658
EXPNO                   1
PROCNO                  1

F2 - Acquisition Parameters
Date_            20151028
Time                 3.21
INSTRUM             spect
PROBHD    5 mm PABBO BB-
PULPROG              zg30
TD                  65536
SOLVENT             CDCl3
NS                     16
DS                      2
SWH            10330.578 Hz
FIDRES          0.157632 Hz
AQ             3.1719923 sec
RG                    128
DW                 48.400 usec
DE                   6.50 usec
TE                  296.0 K
D1            1.00000000 sec

======== CHANNEL f1 ========
NUC1                   1H
P1                   8.90 usec
PLW1          26.00000000 W
SFO1          500.1330885 MHz

F2 - Processing parameters
SI                  65536
SF            500.1300108 MHz
WDW                    EM
SSB       0
LB                   0.30 Hz
GB        0
PC                   1.00
```

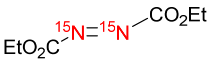

di-^{15}N-DEAD

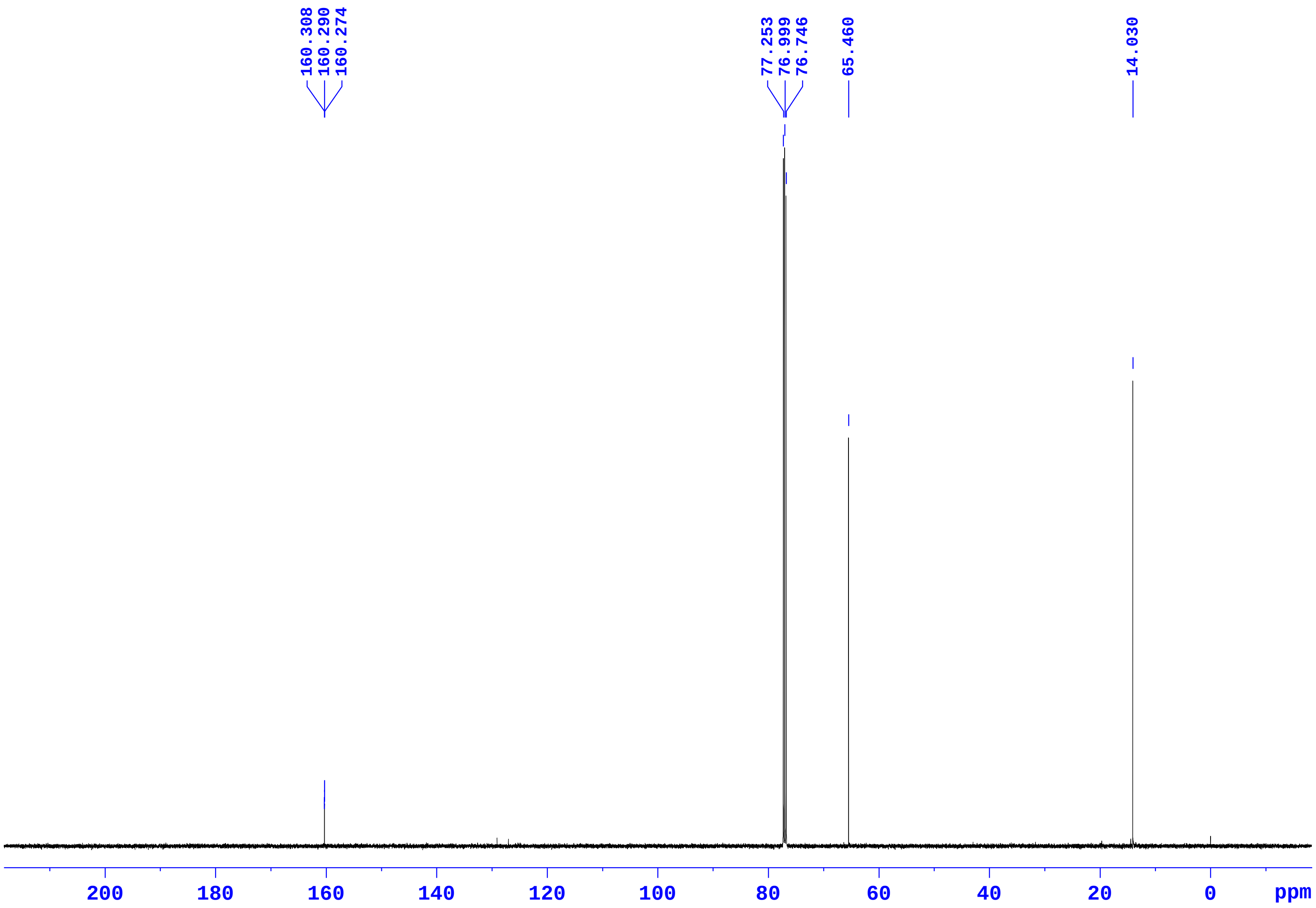


```
Current Data Parameters
NAME              MGS-658
EXPNO                   2
PROCNO                  1

F2 - Acquisition Parameters
Date_            20151028
Time                 4.37
INSTRUM             spect
PROBHD    5 mm PABBO BB-
PULPROG            zgpg30
TD                  65536
SOLVENT             CDCl3
NS                   2048
DS                      4
SWH             29761.904 Hz
FIDRES           0.454131 Hz
AQ              1.1010548 sec
RG                   2050
DW                 16.800 usec
DE                   6.50 usec
TE                  296.0 K
D1             1.00000000 sec
D11            0.03000000 sec

======== CHANNEL f1 ========
NUC1                  13C
P1                   9.00 usec
PLW1         122.00000000 W
SFO1          125.7703637 MHz

======== CHANNEL f2 ========
CPDPRG2           waltz16
NUC2                   1H
PCPD2               80.00 usec
PLW2          26.00000000 W
PLW12          0.32179001 W
PLW13          0.20595001 W
SFO2          500.1320005 MHz

F2 - Processing parameters
SI                  32768
SF            125.7577934 MHz
WDW                    EM
SSB      0
LB                   0.30 Hz
GB       0
PC                   1.40
```

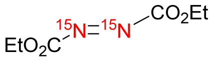

di-^{15}N-DEAD

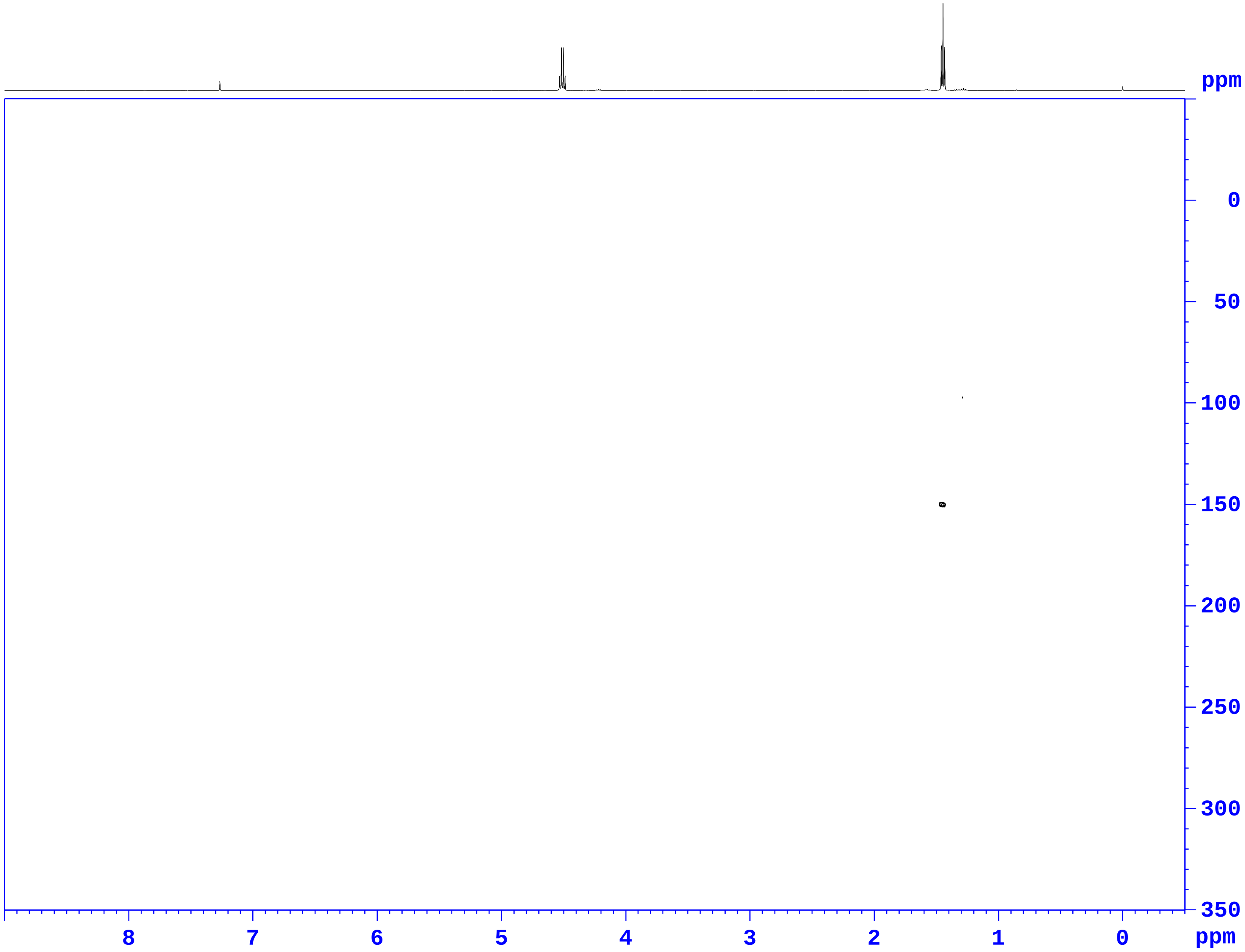


```
Current Data Parameters
NAME              MGS-658
EXPNO                   3
PROCNO                  1

F2 - Acquisition Parameters
Date_            20151028
Time                 4.39
INSTRUM             spect
PROBHD    5 mm PABBO BB-
PULPROG       hmbcgpndqf
TD                   2048
SOLVENT             CDCl3
NS                      8
DS                     16
SWH              2415.459 Hz
FIDRES           1.179423 Hz
AQ              0.4239860 sec
RG                   2050
DW                207.000 usec
DE                   6.50 usec
TE                  296.0 K
CNST13          5.0000000
D0             0.00000300 sec
D1             1.73335004 sec
D6             0.10000000 sec
D16            0.00020000 sec
IN0            0.00002465 sec

======== CHANNEL f1 ========
NUC1                   1H
P1                   8.90 usec
P2                  17.80 usec
PLW1          26.00000000 W
SFO1          500.1312404 MHz

======== CHANNEL f2 ========
NUC2                  15N
P3                  14.40 usec
PLW2         206.00000000 W
SFO2           50.6853342 MHz

====== GRADIENT CHANNEL =====
GPNAM1         SMSQ10.100
GPNAM2         SMSQ10.100
GPNAM3         SMSQ10.100
GPZ1                70.00 %
GPZ2                30.00 %
GPZ3                50.10 %
P16               1000.00 usec

F1 - Acquisition parameters
TD                    256
SFO1             50.68533 MHz
FIDRES          79.195831 Hz
SW                400.000 ppm
FnMODE                 QF

F2 - Processing parameters
SI                   2048
SF            500.1300108 MHz
WDW                  SINE
SSB       0
LB        0 Hz
GB        0
PC                   1.40

F1 - Processing parameters
SI                   1024
MC2                    QF
SF             50.6777330 MHz
WDW          echo-antiecho
SSB       0
LB        0 Hz
GB        0
```

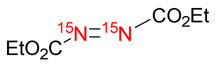

di-^{15}N-DEAD

Reaction mixture:

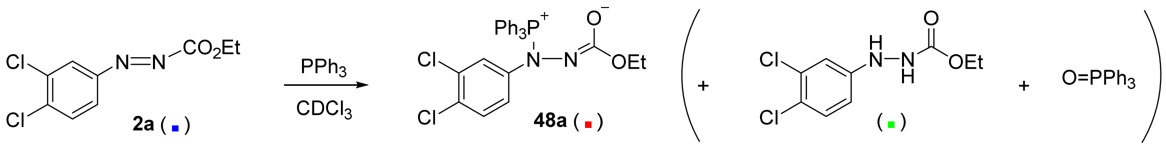

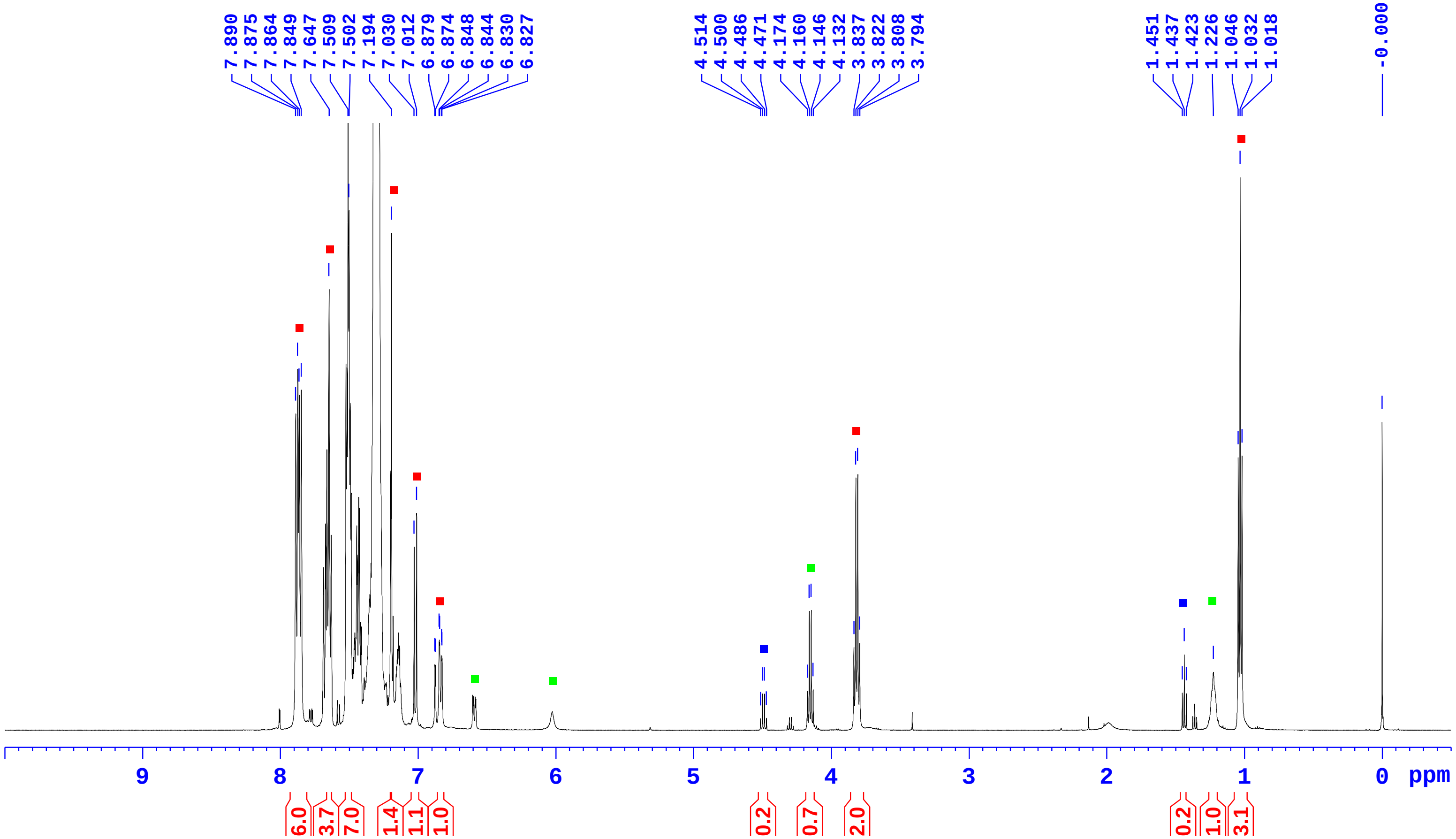


```
Current Data Parameters
NAME              mgs-583
EXPNO                   1
PROCNO                  1

F2 - Acquisition Parameters
Date_            20150613
Time                13.07
INSTRUM             spect
PROBHD   5 mm PABBO BB-
PULPROG              zg30
TD                  65536
SOLVENT             CDCl3
NS                     16
DS                      2
SWH            10330.578 Hz
FIDRES          0.157632 Hz
AQ             3.1719923 sec
RG                     32
DW                 48.400 usec
DE                   6.50 usec
TE                  296.0 K
D1             1.00000000 sec

======== CHANNEL f1 ========
NUC1                   1H
P1                   8.90 usec
PLW1          26.00000000 W
SFO1          500.1330885 MHz

F2 - Processing parameters
SI                  65536
SF            500.1300468 MHz
WDW                    EM
SSB      0
LB                   0.30 Hz
GB       0
PC                   1.00
```

Reaction mixture:

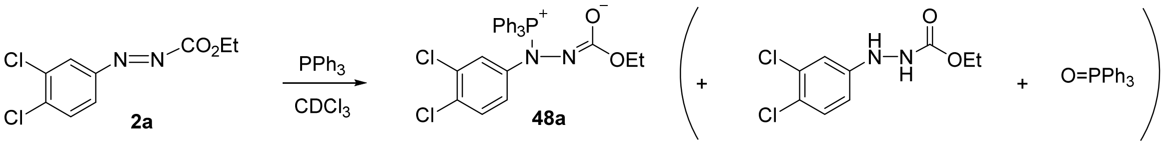

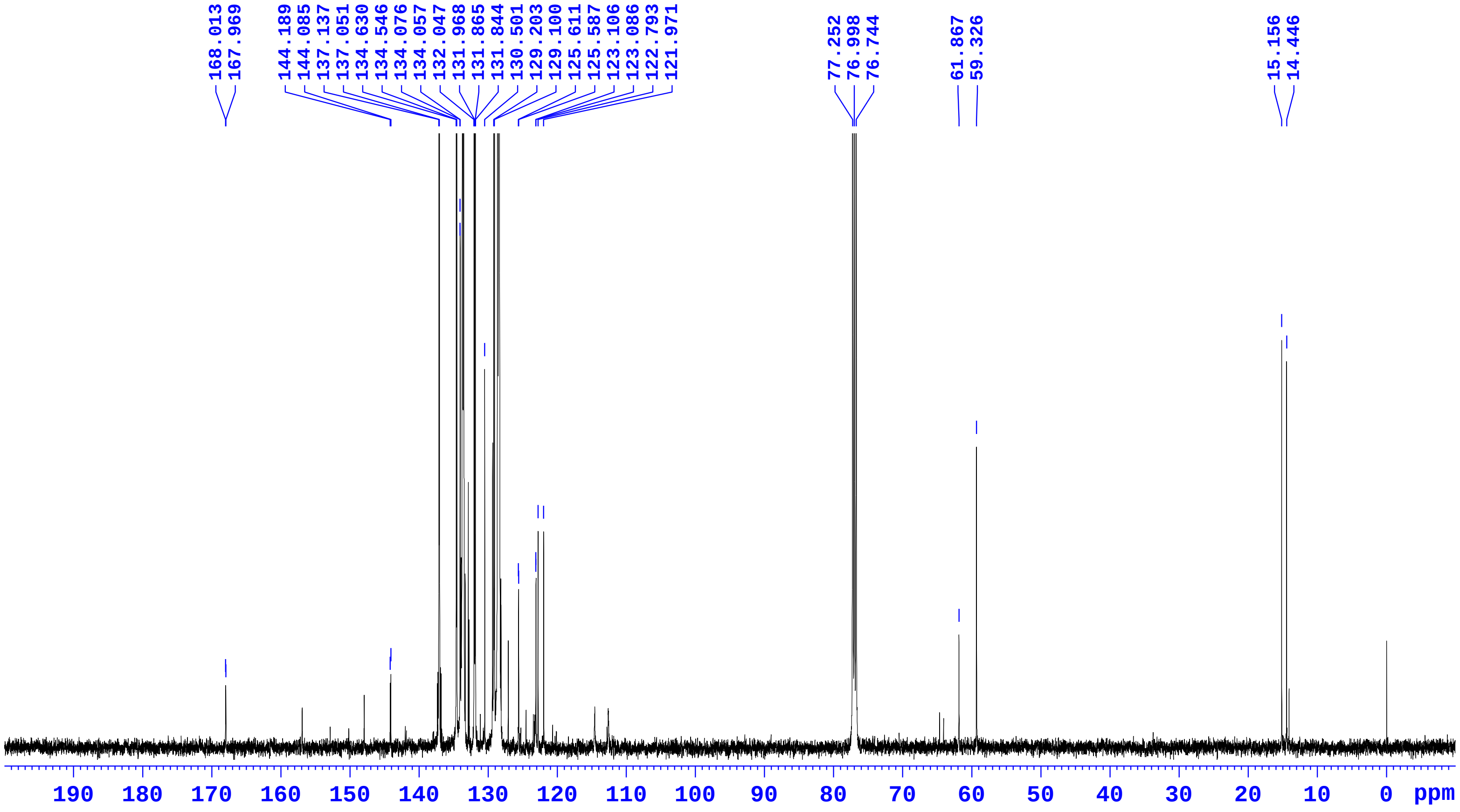


```
Current Data Parameters
NAME               mgs-583
EXPNO                   10
PROCNO                   1

F2 - Acquisition Parameters
Date_             20150614
Time                 10.46
INSTRUM              spect
PROBHD   5 mm PABBO BB-
PULPROG            zgpg30
TD                   65536
SOLVENT              CDCl3
NS                    8192
DS                       4
SWH              29761.904 Hz
FIDRES            0.454131 Hz
AQ               1.1010548 sec
RG                    2050
DW                  16.800 usec
DE                    6.50 usec
TE                   296.0 K
D1              1.00000000 sec
D11             0.03000000 sec

======== CHANNEL f1 ========
NUC1                   13C
P1                    9.00 usec
PLW1          122.00000000 W
SFO1           125.7703637 MHz

======== CHANNEL f2 ========
CPDPRG2            waltz16
NUC2                    1H
PCPD2                80.00 usec
PLW2           26.00000000 W
PLW12           0.32179001 W
PLW13           0.20595001 W
SFO2           500.1320005 MHz

F2 - Processing parameters
SI                   32768
SF             125.7578100 MHz
WDW                     EM
SSB      0
LB                    1.00 Hz
GB       0
PC                    1.40
```

Reaction mixture:

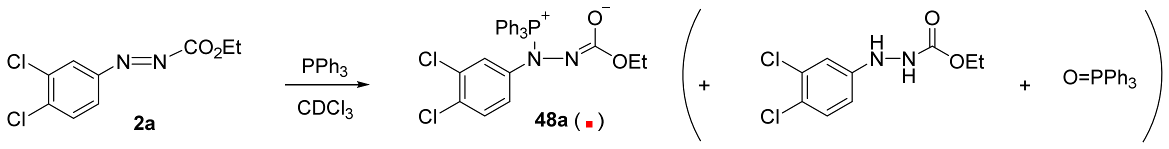

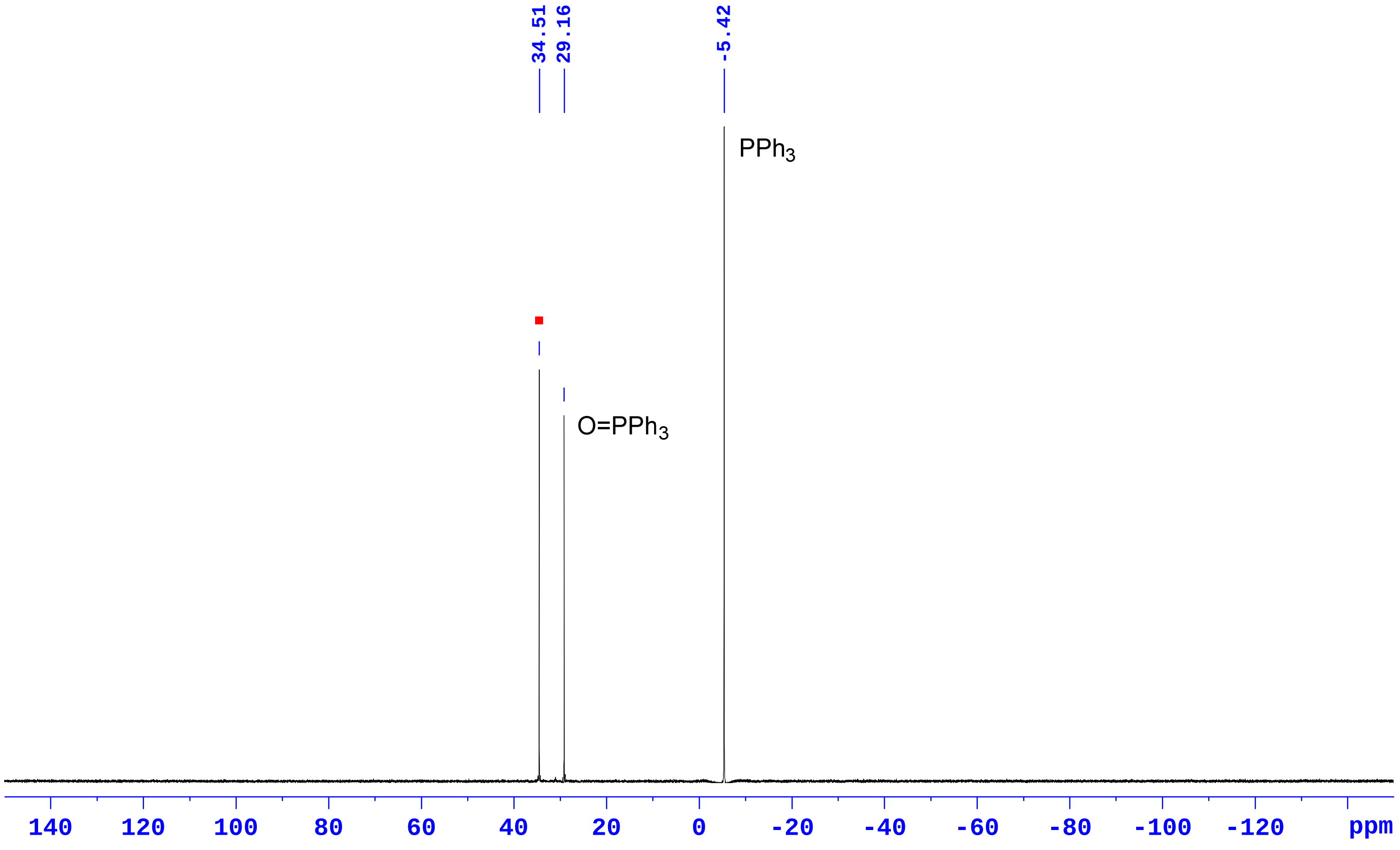


```
Current Data Parameters
NAME             mgs-583
EXPNO                  3
PROCNO                 1

F2 - Acquisition Parameters
Date_           20150613
Time               13.15
INSTRUM            spect
PROBHD    5 mm PABBO BB-
PULPROG          zgpg30
TD                 65536
SOLVENT            CDCl3
NS                    64
DS                     4
SWH            81521.742 Hz
FIDRES          1.243923 Hz
AQ             0.4020041 sec
RG                  2050
DW                 6.133 usec
DE                  6.50 usec
TE                 296.0 K
D1            2.00000000 sec
D11           0.03000000 sec

======== CHANNEL f1 ========
NUC1                 31P
P1                 10.00 usec
PLW1        100.00000000 W
SFO1         202.4462121 MHz

======== CHANNEL f2 ========
CPDPRG2          waltz16
NUC2                  1H
PCPD2              80.00 usec
PLW2         26.00000000 W
PLW12         0.32179001 W
PLW13         0.20595001 W
SFO2         500.1320005 MHz

F2 - Processing parameters
SI                 32768
SF           202.4563350 MHz
WDW                   EM
SSB      0
LB                  1.00 Hz
GB       0
PC                  1.40
```

Reaction mixture:

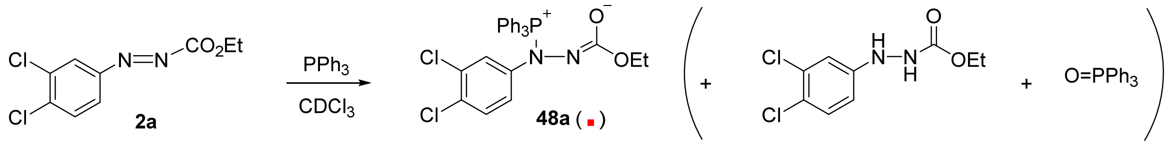

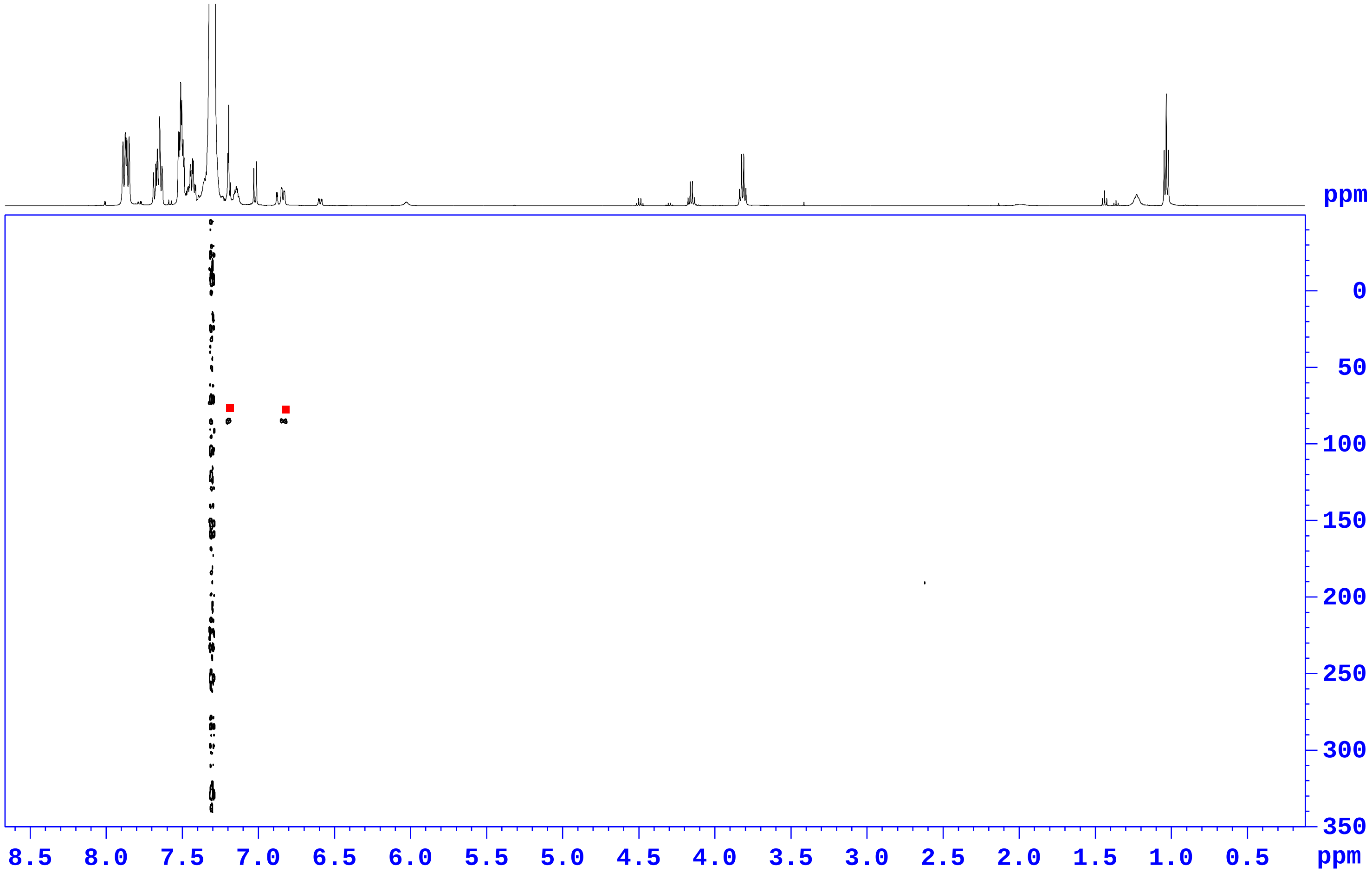


```
Current Data Parameters
NAME              mgs-583
EXPNO                   5
PROCNO                  1

F2 - Acquisition Parameters
Date_            20150613
Time                13.43
INSTRUM             spect
PROBHD    5 mm PABBO BB-
PULPROG       hmbcgpndqf
TD                   2048
SOLVENT             CDCl3
NS                    128
DS                     16
SWH              4273.504 Hz
FIDRES           2.086672 Hz
AQ              0.2396660 sec
RG                   2050
DW                117.000 usec
DE                   6.50 usec
TE                  296.0 K
CNST13          5.0000000
D0             0.00000300 sec
D1             1.91767001 sec
D6             0.10000000 sec
D16            0.00020000 sec
IN0            0.00002465 sec

======== CHANNEL f1 ========
NUC1                   1H
P1                   8.90 usec
P2                  17.80 usec
PLW1          26.00000000 W
SFO1          500.1322440 MHz

======== CHANNEL f2 ========
NUC2                  15N
P3                  14.40 usec
PLW2         206.00000000 W
SFO2           50.6853342 MHz

====== GRADIENT CHANNEL =====
GPNAM1         SMSQ10.100
GPNAM2         SMSQ10.100
GPNAM3         SMSQ10.100
GPZ1                70.00 %
GPZ2                30.00 %
GPZ3                50.10 %
P16               1000.00 usec

F1 - Acquisition parameters
TD                    128
SFO1             50.68533 MHz
FIDRES         158.391663 Hz
SW                400.000 ppm
FnMODE                 QF

F2 - Processing parameters
SI                   2048
SF            500.1300468 MHz
WDW                  SINE
SSB      0
LB       0 Hz
GB       0
PC                   1.40

F1 - Processing parameters
SI                   1024
MC2                    QF
SF             50.6777330 MHz
WDW                States
SSB      0
LB       0 Hz
GB       0
```

Reaction mixture:

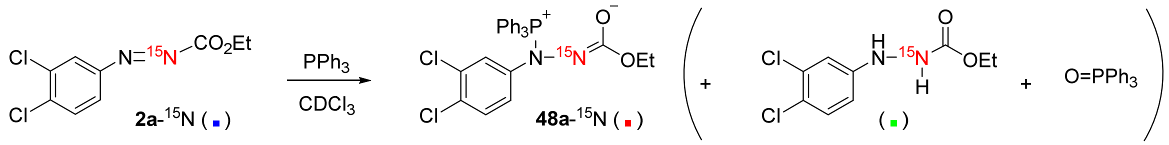

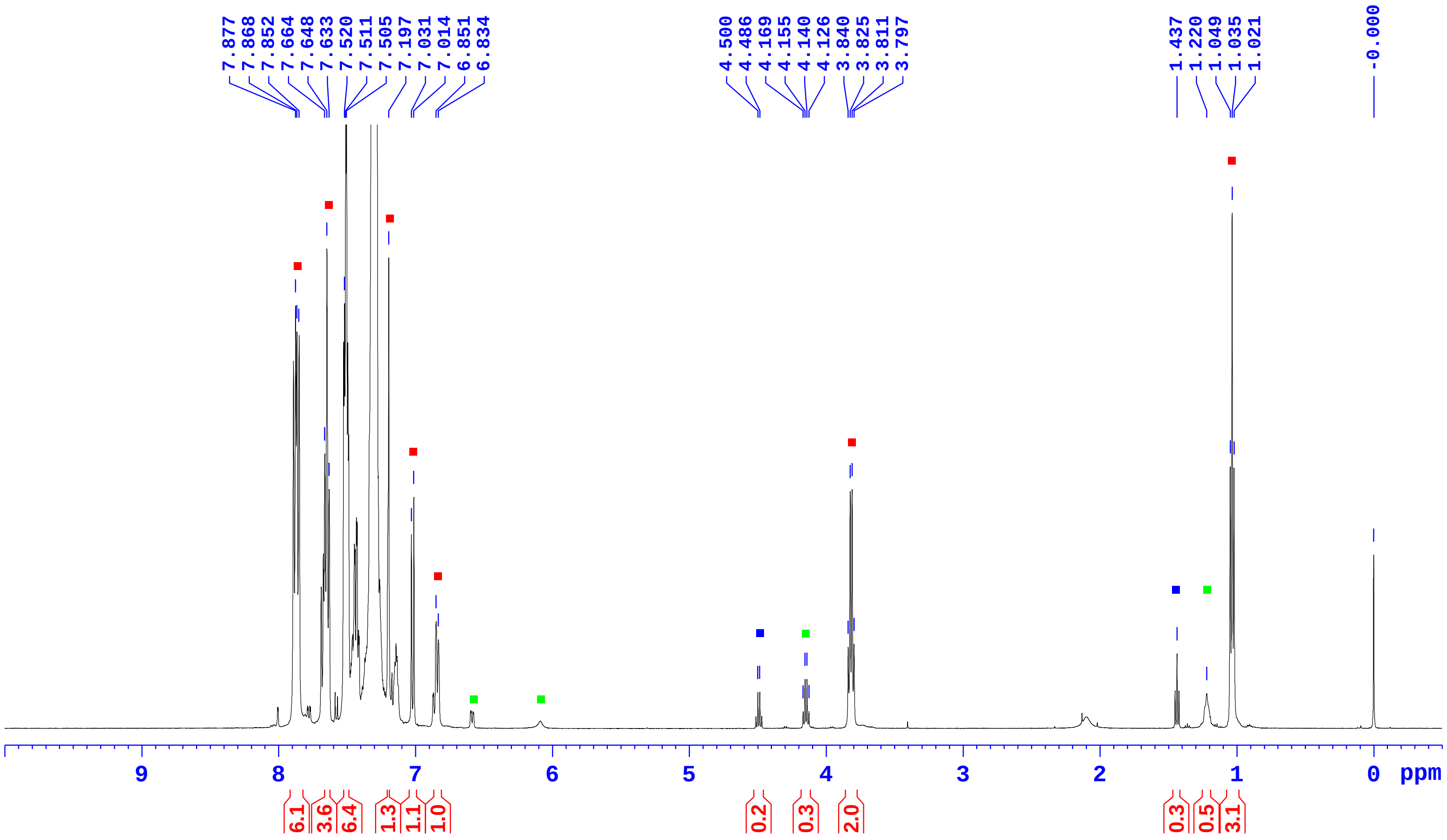


```
Current Data Parameters
NAME              mgs-576
EXPNO                  10
PROCNO                  1

F2 - Acquisition Parameters
Date_            20150606
Time                13.24
INSTRUM             spect
PROBHD   5 mm PABBO BB-
PULPROG              zg30
TD                  65536
SOLVENT             CDCl3
NS                     16
DS                      2
SWH             10330.578 Hz
FIDRES           0.157632 Hz
AQ              3.1719923 sec
RG                     32
DW                 48.400 usec
DE                   6.50 usec
TE                  296.0 K
D1             1.00000000 sec

======== CHANNEL f1 ========
NUC1                   1H
P1                   8.90 usec
PLW1          26.00000000 W
SFO1          500.1330885 MHz

F2 - Processing parameters
SI                  65536
SF            500.1300453 MHz
WDW                    EM
SSB      0
LB                   0.30 Hz
GB       0
PC                   1.00
```

Reaction mixture:

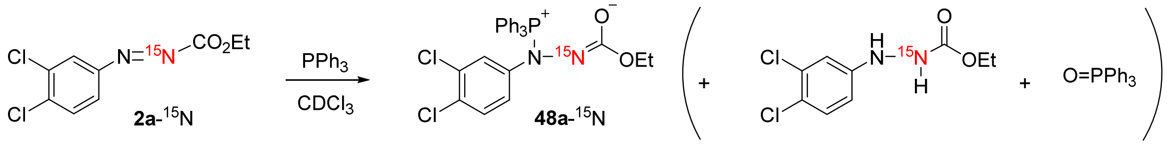

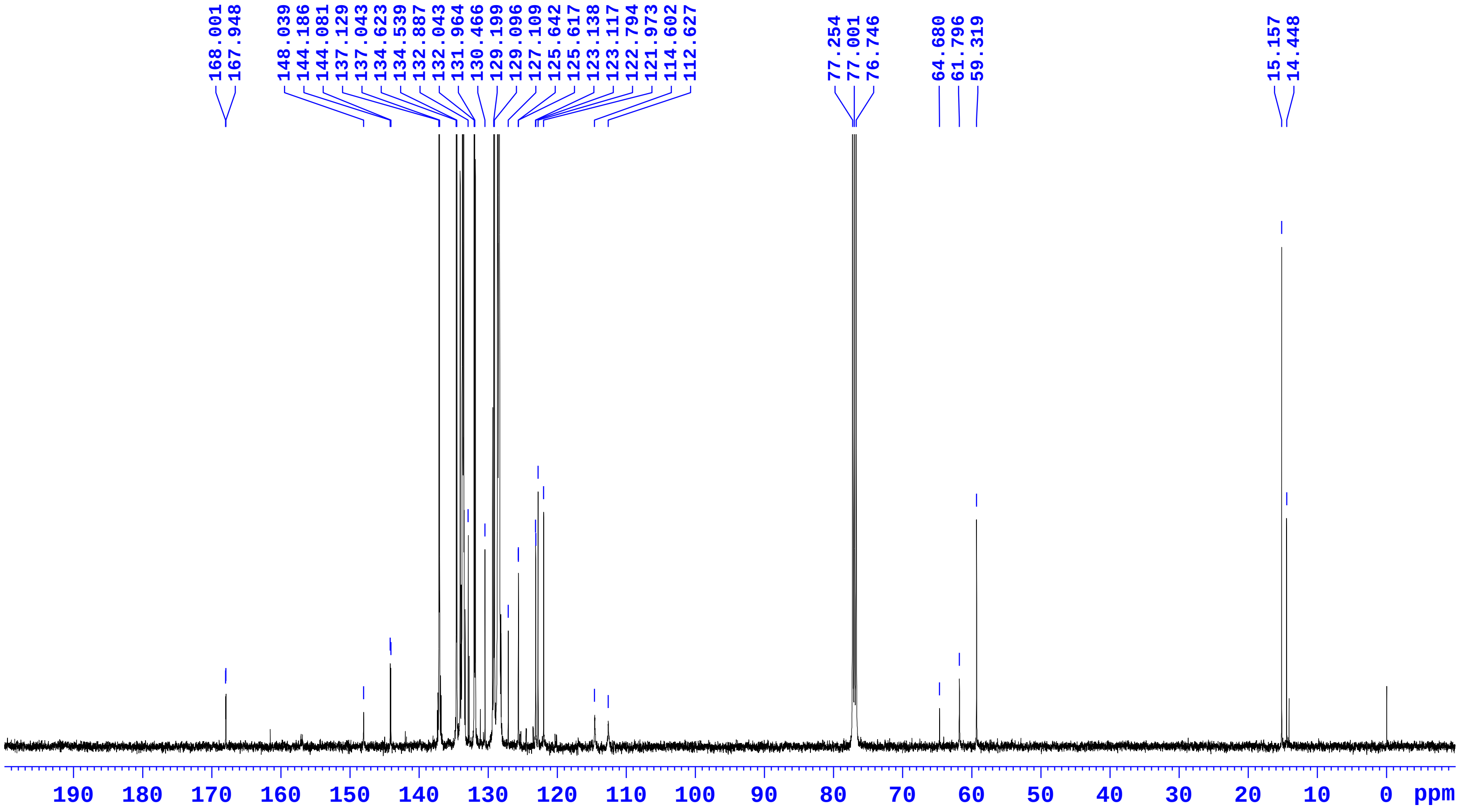


```
Current Data Parameters
NAME             mgs-576
EXPNO                 22
PROCNO                 1

F2 - Acquisition Parameters
Date_           20150606
Time               20.16
INSTRUM            spect
PROBHD   5 mm PABBO BB-
PULPROG          zgpg30
TD                65536
SOLVENT            CDCl3
NS                11264
DS                     4
SWH            29761.904 Hz
FIDRES          0.454131 Hz
AQ             1.1010548 sec
RG                  2050
DW                16.800 usec
DE                  6.50 usec
TE                 296.0 K
D1            1.00000000 sec
D11           0.03000000 sec

======== CHANNEL f1 ========
NUC1                 13C
P1                  9.00 usec
PLW1        122.00000000 W
SFO1         125.7703637 MHz

======== CHANNEL f2 ========
CPDPRG2          waltz16
NUC2                  1H
PCPD2              80.00 usec
PLW2         26.00000000 W
PLW12         0.32179001 W
PLW13         0.20595001 W
SFO2         500.1320005 MHz

F2 - Processing parameters
SI                 32768
SF           125.7578104 MHz
WDW                   EM
SSB      0
LB                  1.00 Hz
GB       0
PC                  1.40
```

Reaction mixture:

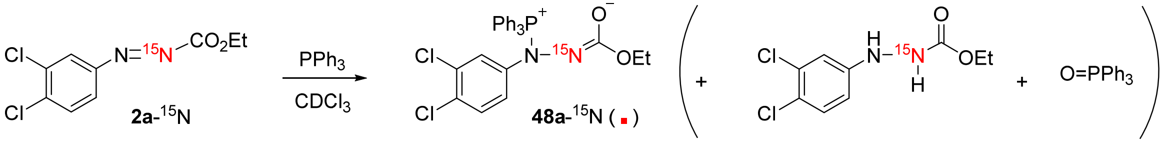

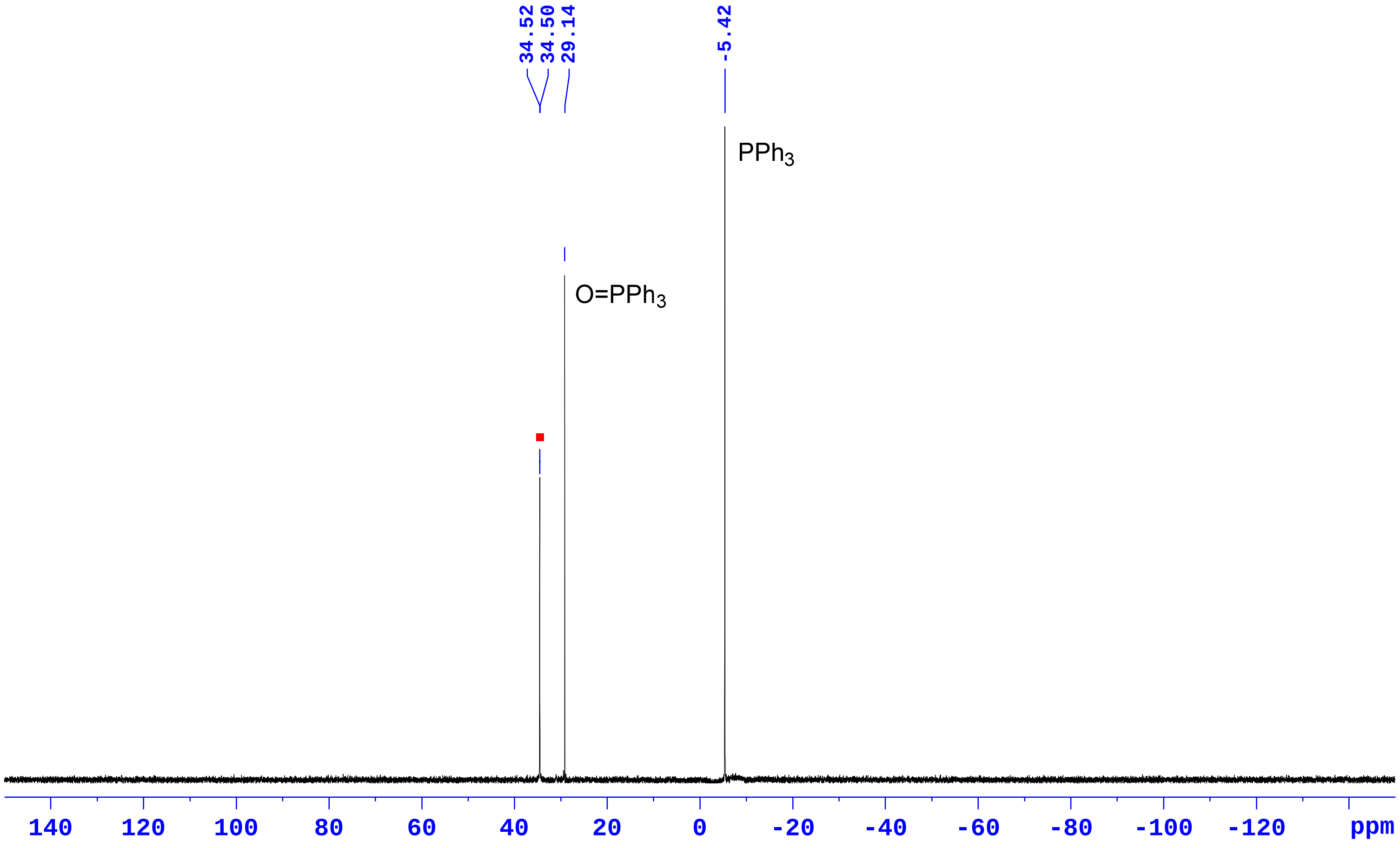


```
Current Data Parameters
NAME             mgs-576
EXPNO                 12
PROCNO                 1

F2 - Acquisition Parameters
Date_           20150606
Time               13.32
INSTRUM            spect
PROBHD   5 mm PABBO BB-
PULPROG          zgpg30
TD                 65536
SOLVENT            CDCl3
NS                    25
DS                     4
SWH            81521.742 Hz
FIDRES          1.243923 Hz
AQ             0.4020041 sec
RG                  2050
DW                 6.133 usec
DE                  6.50 usec
TE                 296.1 K
D1            2.00000000 sec
D11           0.03000000 sec

======== CHANNEL f1 ========
NUC1                 31P
P1                 10.00 usec
PLW1        100.00000000 W
SFO1         202.4462121 MHz

======== CHANNEL f2 ========
CPDPRG2          waltz16
NUC2                  1H
PCPD2              80.00 usec
PLW2         26.00000000 W
PLW12         0.32179001 W
PLW13         0.20595001 W
SFO2         500.1320005 MHz

F2 - Processing parameters
SI                 32768
SF           202.4563350 MHz
WDW                   EM
SSB      0
LB                  0.25 Hz
GB       0
PC                  1.40
```

Reaction mixture:

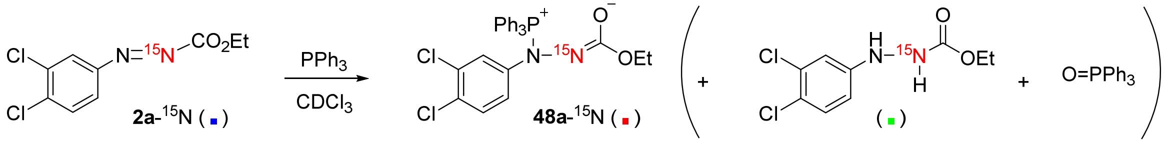

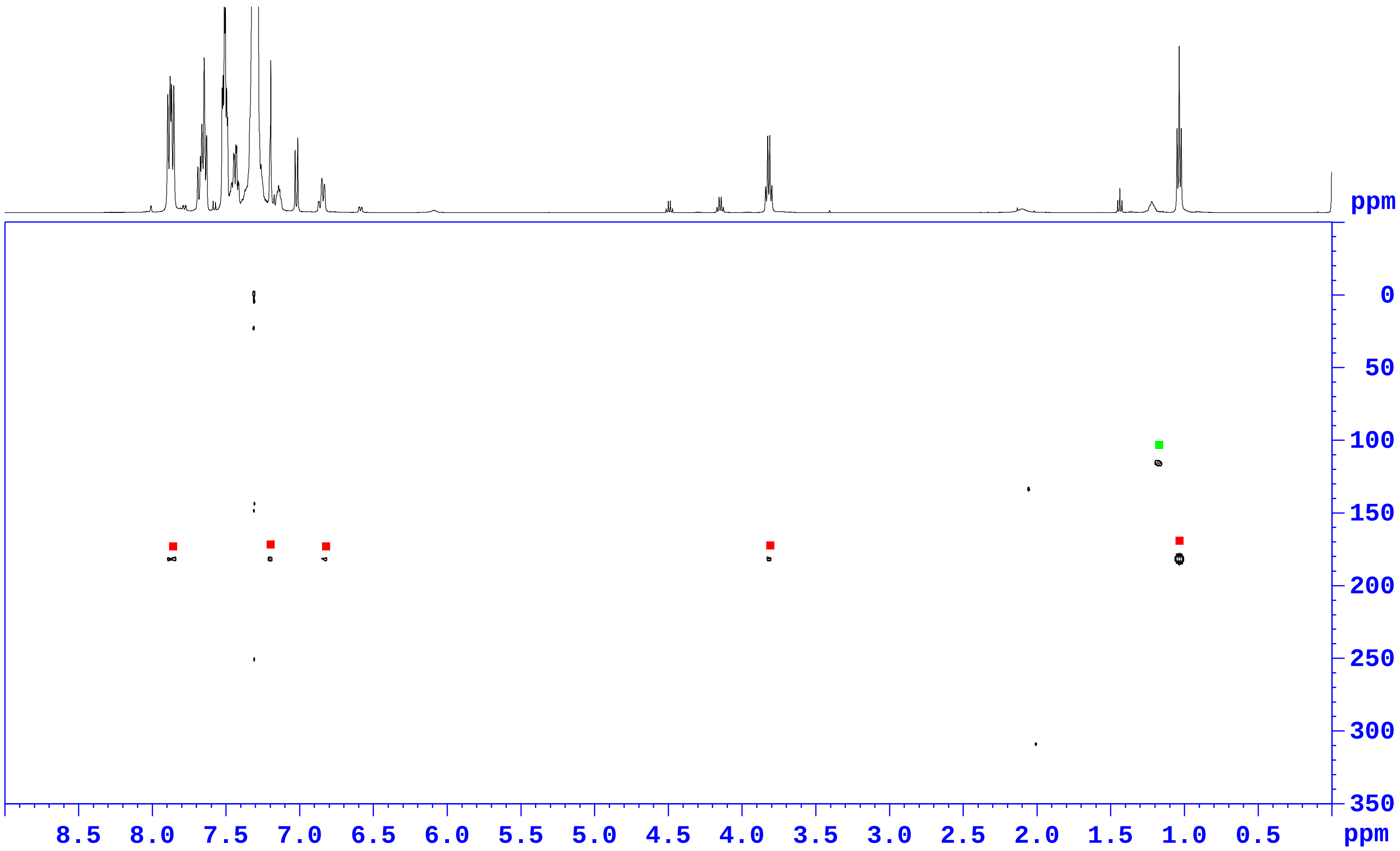


```
Current Data Parameters
NAME                mgs-576
EXPNO                    17
PROCNO                    1

F2 - Acquisition Parameters
Date_              20150606
Time                  14.13
INSTRUM               spect
PROBHD   5 mm PABBO BB-
PULPROG          hmbcgpndqf
TD                     2048
SOLVENT               CDCl3
NS                       32
DS                       16
SWH                4310.345 Hz
FIDRES             2.104661 Hz
AQ                0.2376180 sec
RG                     2050
DW                  116.000 usec
DE                     6.50 usec
TE                    296.0 K
CNST13            5.0000000
D0               0.00000300 sec
D1               1.91971803 sec
D6               0.10000000 sec
D16              0.00020000 sec
IN0              0.00002465 sec

======== CHANNEL f1 ========
NUC1                     1H
P1                     8.90 usec
P2                    17.80 usec
PLW1            26.00000000 W
SFO1            500.1323408 MHz

======== CHANNEL f2 ========
NUC2                    15N
P3                    14.40 usec
PLW2           206.00000000 W
SFO2             50.6853347 MHz

====== GRADIENT CHANNEL =====
GPNAM1           SMSQ10.100
GPNAM2           SMSQ10.100
GPNAM3           SMSQ10.100
GPZ1                  70.00 %
GPZ2                  30.00 %
GPZ3                  50.10 %
P16                 1000.00 usec

F1 - Acquisition parameters
TD                      128
SFO1               50.68533 MHz
FIDRES           158.391663 Hz
SW                  400.000 ppm
FnMODE                   QF

F2 - Processing parameters
SI                     2048
SF              500.1300456 MHz
WDW                    SINE
SSB      0
LB       0 Hz
GB       0
PC                     1.40

F1 - Processing parameters
SI                     1024
MC2                      QF
SF               50.6777330 MHz
WDW                  States
SSB      0
LB       0 Hz
GB       0
```

Reaction mixture:

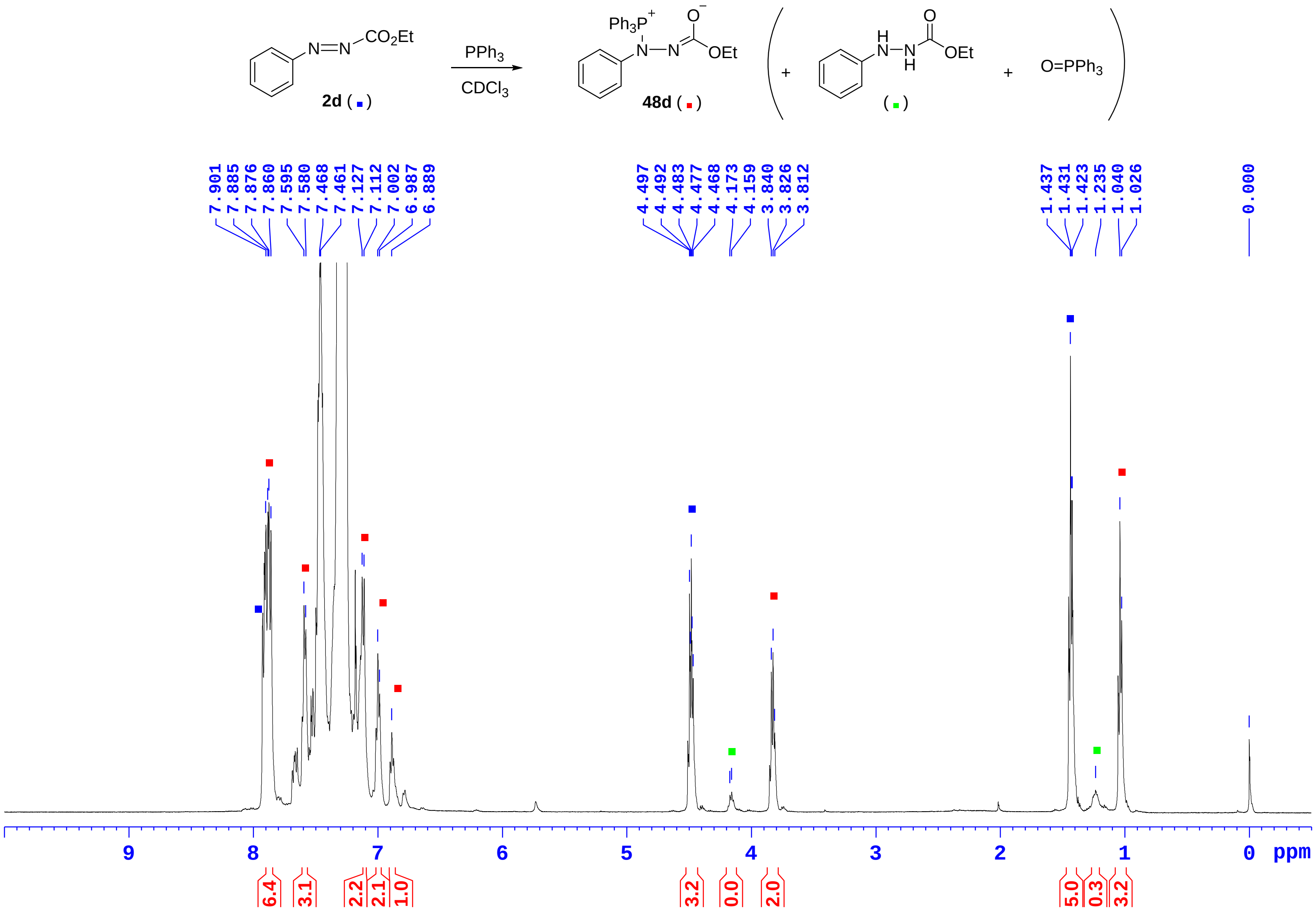


```
Current Data Parameters
NAME              MGS-661
EXPNO                  20
PROCNO                  1

F2 - Acquisition Parameters
Date_            20151103
Time                 9.06
INSTRUM             spect
PROBHD   5 mm PABBO BB-
PULPROG              zg30
TD                  65536
SOLVENT             CDCl3
NS                     16
DS                      2
SWH             10330.578 Hz
FIDRES           0.157632 Hz
AQ              3.1719923 sec
RG                     32
DW                 48.400 usec
DE                   6.50 usec
TE                  296.0 K
D1             1.00000000 sec

======== CHANNEL f1 ========
NUC1                   1H
P1                   8.90 usec
PLW1          26.00000000 W
SFO1          500.1330885 MHz

F2 - Processing parameters
SI                  65536
SF            500.1300539 MHz
WDW                    EM
SSB      0
LB                   0.30 Hz
GB       0
PC                   1.00
```

Reaction mixture:

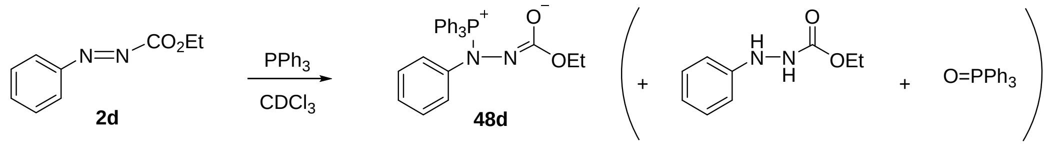

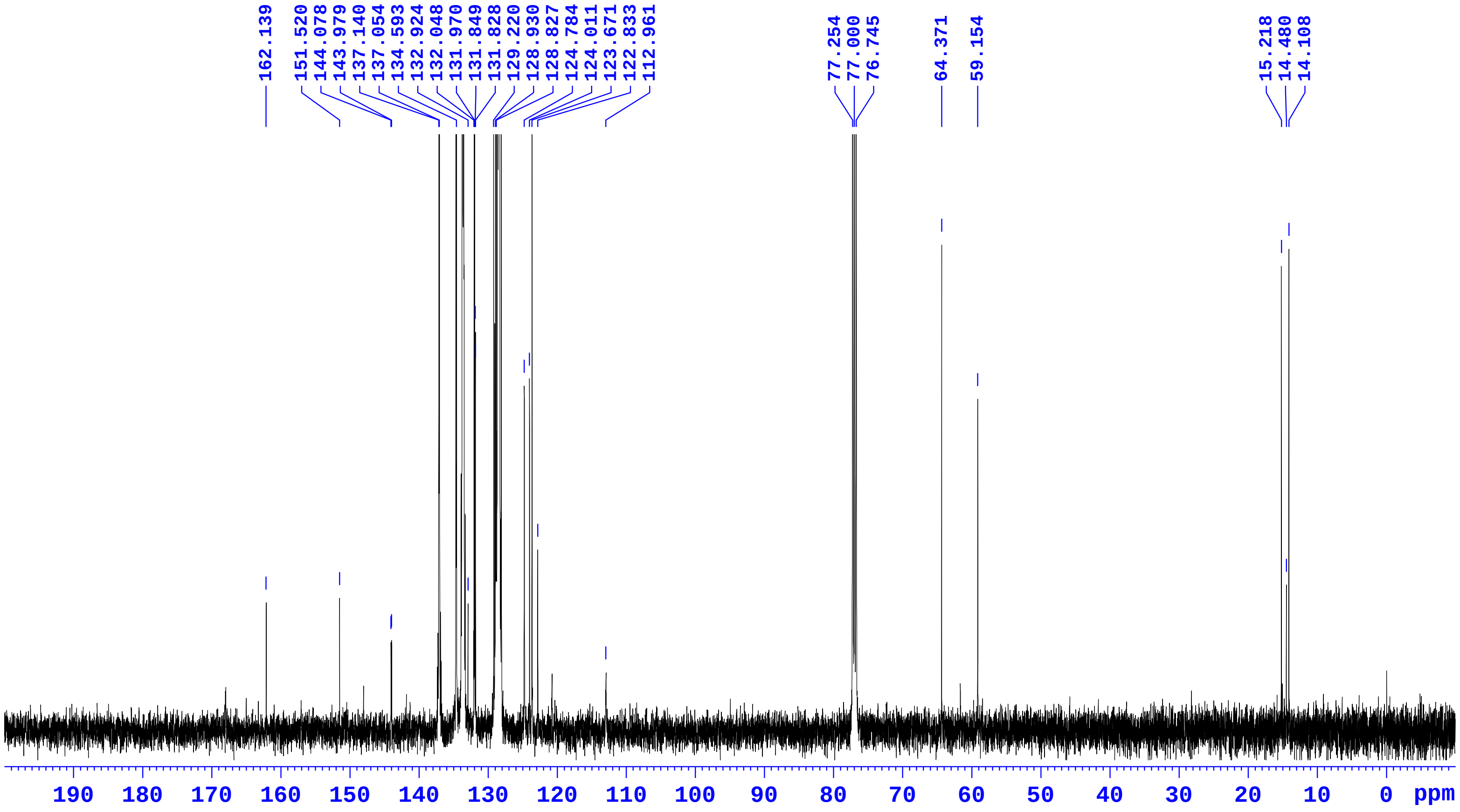


```
Current Data Parameters
NAME              MGS-661
EXPNO                  25
PROCNO                  1

F2 - Acquisition Parameters
Date_            20151103
Time                12.14
INSTRUM             spect
PROBHD    5 mm PABBO BB-
PULPROG            zgpg30
TD                  65536
SOLVENT             CDCl3
NS                   2048
DS                      4
SWH             29761.904 Hz
FIDRES           0.454131 Hz
AQ              1.1010548 sec
RG                   2050
DW                 16.800 usec
DE                   6.50 usec
TE                  296.0 K
D1             1.00000000 sec
D11            0.03000000 sec

======== CHANNEL f1 ========
NUC1                  13C
P1                   9.00 usec
PLW1         122.00000000 W
SFO1          125.7703637 MHz

======== CHANNEL f2 ========
CPDPRG2           waltz16
NUC2                   1H
PCPD2               80.00 usec
PLW2          26.00000000 W
PLW12          0.32179001 W
PLW13          0.20595001 W
SFO2          500.1320005 MHz

F2 - Processing parameters
SI                  32768
SF            125.7578114 MHz
WDW                    EM
SSB      0
LB                   1.00 Hz
GB       0
PC                   1.40
```

Reaction mixture:

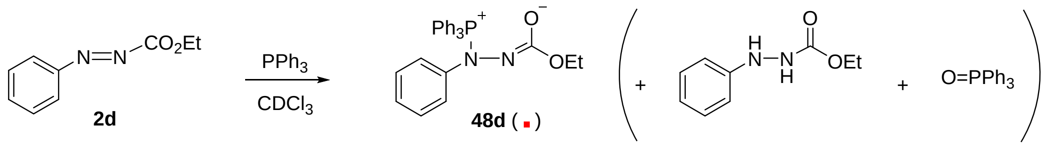

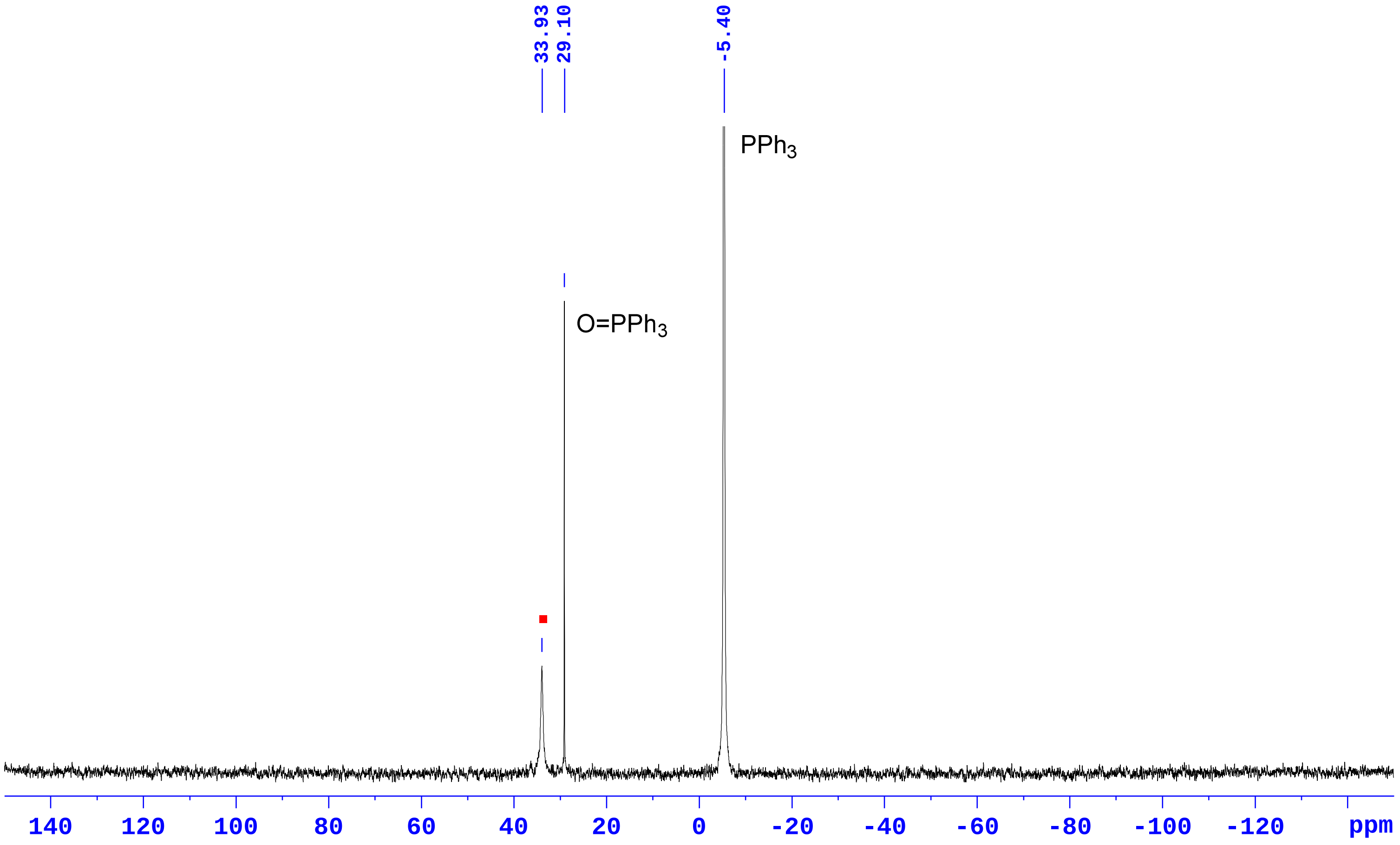


```
Current Data Parameters
NAME              MGS-472
EXPNO                   5
PROCNO                  1

F2 - Acquisition Parameters
Date_            20150217
Time                18.38
INSTRUM             spect
PROBHD   5 mm PABBO BB-
PULPROG           zgpg30
TD                 65536
SOLVENT            CDCl3
NS                    16
DS                     4
SWH            81521.742 Hz
FIDRES          1.243923 Hz
AQ             0.4020041 sec
RG                  1440
DW                 6.133 usec
DE                  6.50 usec
TE                 296.1 K
D1            2.00000000 sec
D11           0.03000000 sec

======== CHANNEL f1 ========
NUC1                 31P
P1                 10.00 usec
PLW1        100.00000000 W
SFO1         202.4462121 MHz

======== CHANNEL f2 ========
CPDPRG2          waltz16
NUC2                  1H
PCPD2              80.00 usec
PLW2         26.00000000 W
PLW12         0.32179001 W
PLW13         0.20595001 W
SFO2         500.1320005 MHz

F2 - Processing parameters
SI                 32768
SF           202.4563350 MHz
WDW                   EM
SSB      0
LB                 10.00 Hz
GB       0
PC                  1.40
```

Reaction mixture:

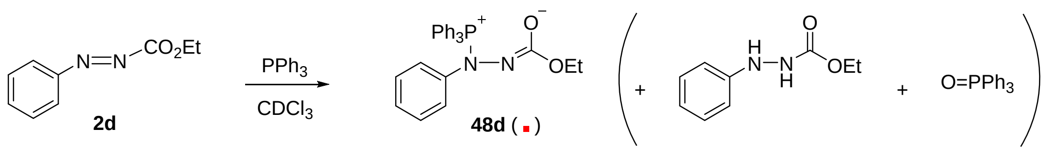

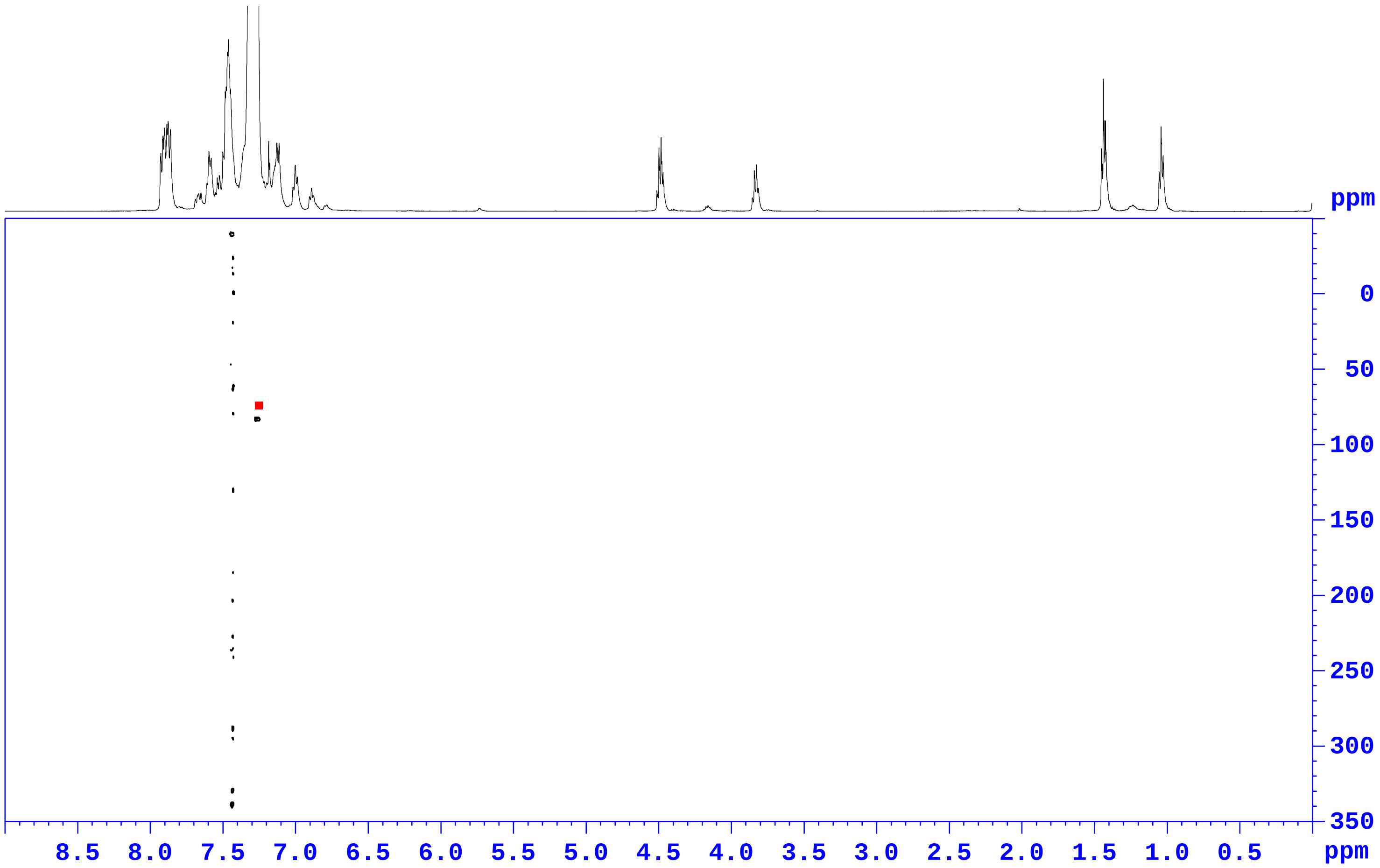


```
Current Data Parameters
NAME              MGS-472
EXPNO                   6
PROCNO                  1

F2 - Acquisition Parameters
Date_            20150217
Time                18.51
INSTRUM             spect
PROBHD   5 mm PABBO BB-
PULPROG        hmbcgpndqf
TD                   2048
SOLVENT             CDCl3
NS                    128
DS                     16
SWH              4273.504 Hz
FIDRES           2.086672 Hz
AQ              0.2396660 sec
RG                   2050
DW                117.000 usec
DE                   6.50 usec
TE                  296.0 K
CNST13          5.0000000
D0             0.00000300 sec
D1             1.91767001 sec
D6             0.10000000 sec
D16            0.00020000 sec
IN0            0.00002465 sec

======== CHANNEL f1 ========
NUC1                   1H
P1                   8.90 usec
P2                  17.80 usec
PLW1          26.00000000 W
SFO1          500.1323611 MHz

======== CHANNEL f2 ========
NUC2                  15N
P3                  14.40 usec
PLW2         206.00000000 W
SFO2           50.6853342 MHz

====== GRADIENT CHANNEL =====
GPNAM1         SMSQ10.100
GPNAM2         SMSQ10.100
GPNAM3         SMSQ10.100
GPZ1                70.00 %
GPZ2                30.00 %
GPZ3                50.10 %
P16               1000.00 usec

F1 - Acquisition parameters
TD                    128
SFO1             50.68533 MHz
FIDRES         158.391663 Hz
SW                400.000 ppm
FnMODE                 QF

F2 - Processing parameters
SI                   2048
SF            500.1300000 MHz
WDW                  SINE
SSB      0
LB       0 Hz
GB       0
PC                   1.40

F1 - Processing parameters
SI                   1024
MC2                    QF
SF             50.6777330 MHz
WDW          echo-antiecho
SSB      0
LB       0 Hz
GB       0
```

Reaction mixture:

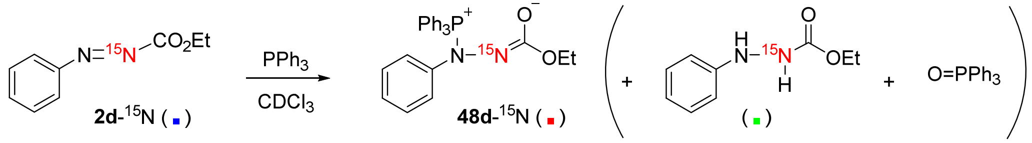

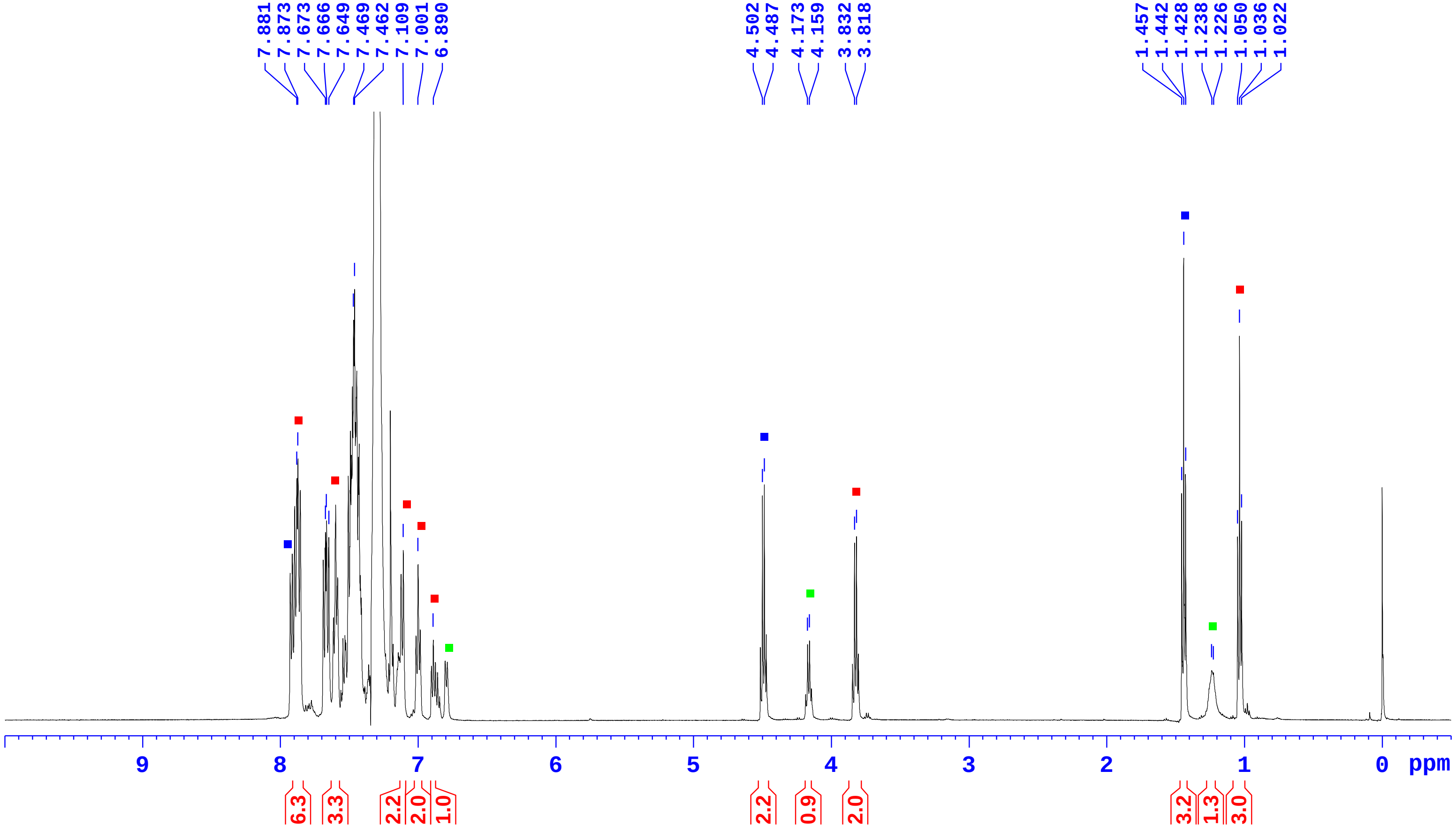


```
Current Data Parameters
NAME              MGS-645
EXPNO                  10
PROCNO                  1

F2 - Acquisition Parameters
Date_            20151014
Time                 8.57
INSTRUM             spect
PROBHD   5 mm PABBO BB-
PULPROG              zg30
TD                  65536
SOLVENT             CDCl3
NS                     16
DS                      2
SWH             10330.578 Hz
FIDRES           0.157632 Hz
AQ              3.1719923 sec
RG                     32
DW                 48.400 usec
DE                   6.50 usec
TE                  297.2 K
D1             1.00000000 sec

======== CHANNEL f1 ========
NUC1                   1H
P1                   8.90 usec
PLW1          26.00000000 W
SFO1          500.1330885 MHz

F2 - Processing parameters
SI                  65536
SF            500.1300430 MHz
WDW                    EM
SSB      0
LB                   0.30 Hz
GB       0
PC                   1.00
```

Reaction mixture:

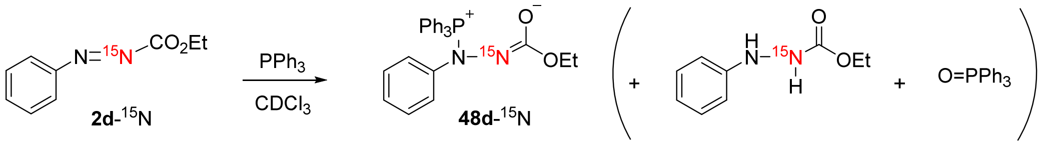

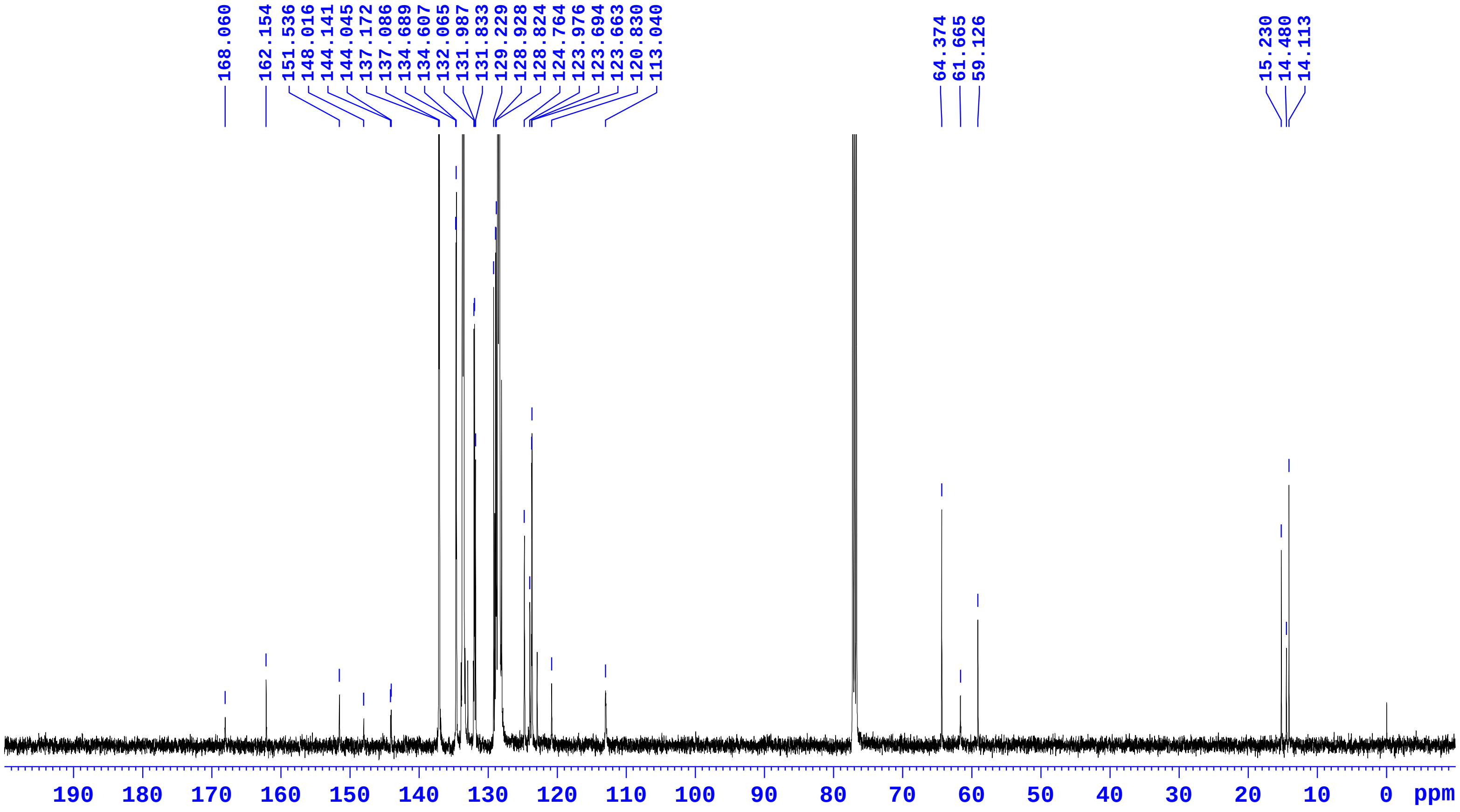


```
Current Data Parameters
NAME              MGS-645
EXPNO                   8
PROCNO                  1

F2 - Acquisition Parameters
Date_            20151013
Time                23.39
INSTRUM             spect
PROBHD   5 mm PABBO BB-
PULPROG           zgpg30
TD                  65536
SOLVENT             CDCl3
NS                   5120
DS                      4
SWH             29761.904 Hz
FIDRES           0.454131 Hz
AQ              1.1010548 sec
RG                   2050
DW                 16.800 usec
DE                   6.50 usec
TE                  297.5 K
D1             1.00000000 sec
D11            0.03000000 sec

======== CHANNEL f1 ========
NUC1                  13C
P1                   9.00 usec
PLW1         122.00000000 W
SFO1          125.7703637 MHz

======== CHANNEL f2 ========
CPDPRG2           waltz16
NUC2                   1H
PCPD2               80.00 usec
PLW2          26.00000000 W
PLW12          0.32179001 W
PLW13          0.20595001 W
SFO2          500.1320005 MHz

F2 - Processing parameters
SI                  32768
SF            125.7578067 MHz
WDW                    EM
SSB      0
LB                   1.00 Hz
GB       0
PC                   1.40
```

Reaction mixture:

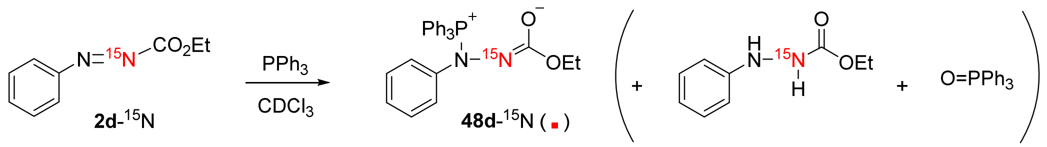

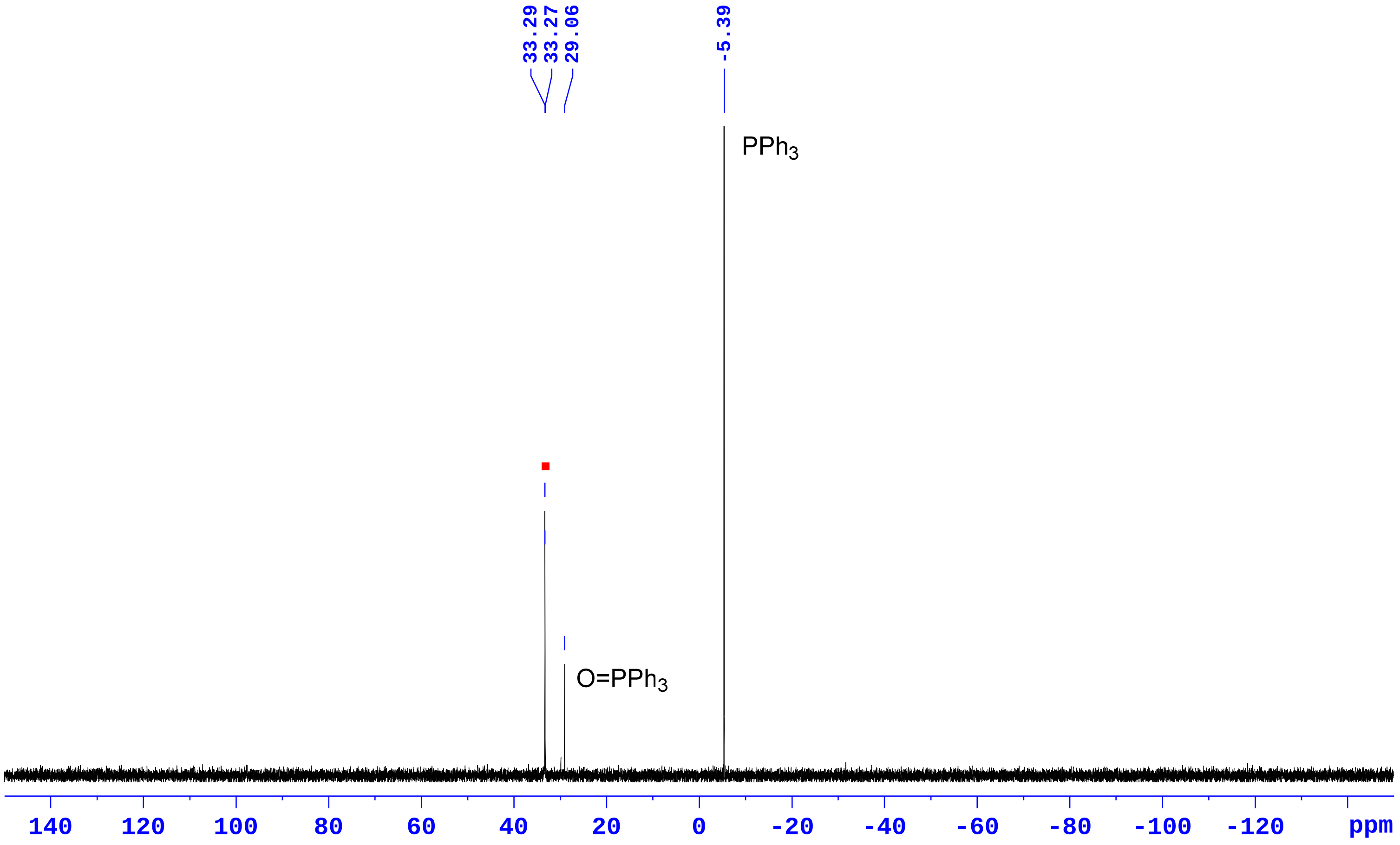


```
Current Data Parameters
NAME              MGS-645
EXPNO                   3
PROCNO                  1

F2 - Acquisition Parameters
Date_            20151013
Time                17.14
INSTRUM             spect
PROBHD     5 mm PABBO BB-
PULPROG           zgpg30
TD                  65536
SOLVENT             CDCl3
NS                     16
DS                      4
SWH             81521.742 Hz
FIDRES           1.243923 Hz
AQ              0.4020041 sec
RG                   2050
DW                  6.133 usec
DE                   6.50 usec
TE                  297.8 K
D1             2.00000000 sec
D11            0.03000000 sec

======== CHANNEL f1 ========
NUC1                  31P
P1                  10.00 usec
PLW1         100.00000000 W
SFO1          202.4462121 MHz

======== CHANNEL f2 ========
CPDPRG2           waltz16
NUC2                   1H
PCPD2               80.00 usec
PLW2          26.00000000 W
PLW12          0.32179001 W
PLW13          0.20595001 W
SFO2          500.1320005 MHz

F2 - Processing parameters
SI                  32768
SF            202.4563350 MHz
WDW                    no
SSB        0
LB         0 Hz
GB         0
PC                   1.40
```

Reaction mixture:

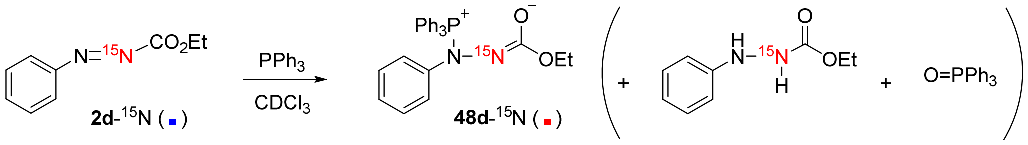

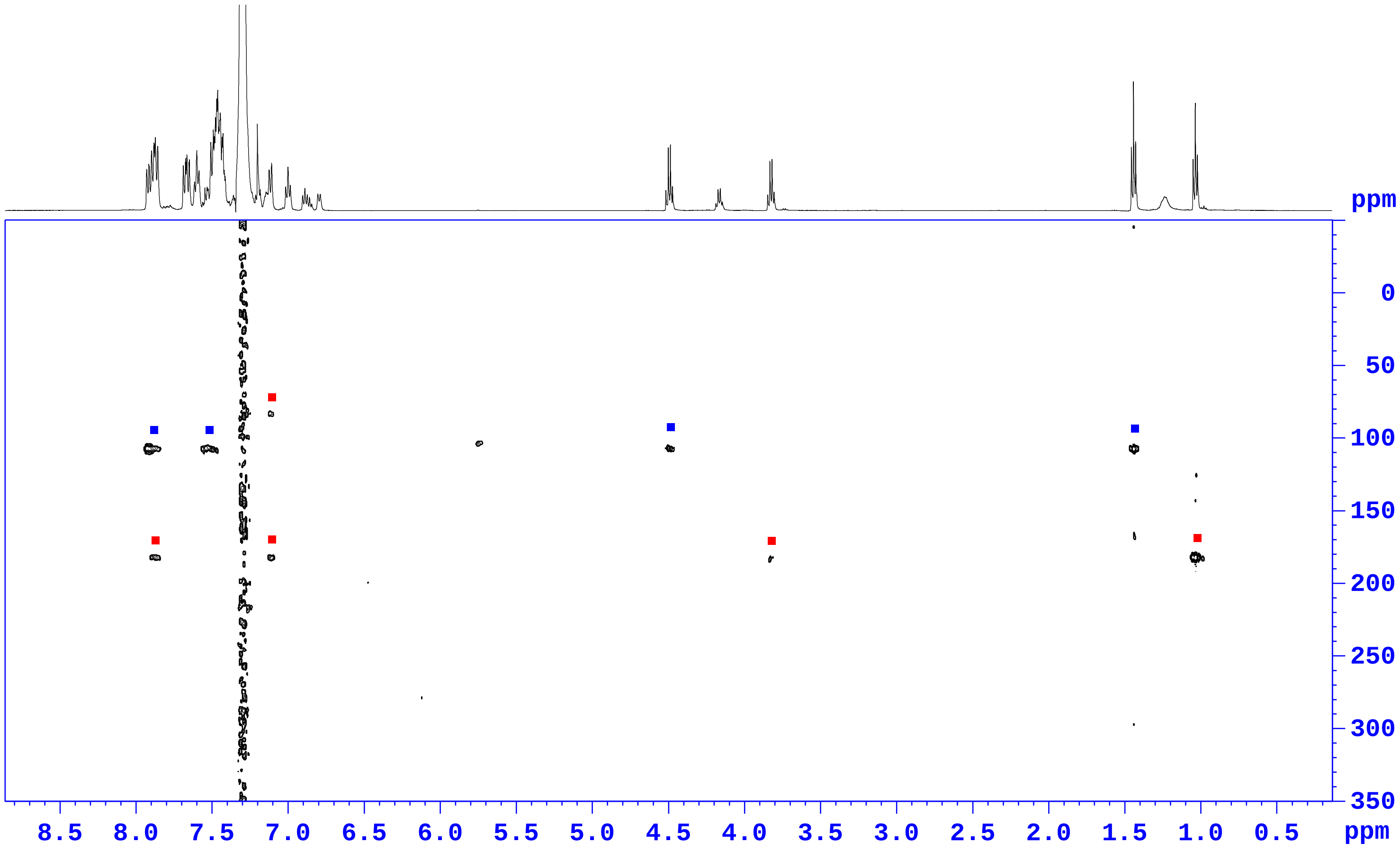


```
Current Data Parameters
NAME              MGS-645
EXPNO                   6
PROCNO                  1

F2 - Acquisition Parameters
Date_            20151013
Time                17.30
INSTRUM             spect
PROBHD    5 mm PABBO BB-
PULPROG        hmbcgpndqf
TD                   2048
SOLVENT             CDCl3
NS                     32
DS                     16
SWH              4424.779 Hz
FIDRES           2.160537 Hz
AQ              0.2314740 sec
RG                   2050
DW                113.000 usec
DE                   6.50 usec
TE                  297.5 K
CNST13          5.0000000
D0             0.00000300 sec
D1             1.92586195 sec
D6             0.10000000 sec
D16            0.00020000 sec
IN0            0.00002465 sec

======== CHANNEL f1 ========
NUC1                   1H
P1                   8.90 usec
P2                  17.80 usec
PLW1          26.00000000 W
SFO1          500.1323232 MHz

======== CHANNEL f2 ========
NUC2                  15N
P3                  14.40 usec
PLW2         206.00000000 W
SFO2           50.6853342 MHz

====== GRADIENT CHANNEL =====
GPNAM1         SMSQ10.100
GPNAM2         SMSQ10.100
GPNAM3         SMSQ10.100
GPZ1                70.00 %
GPZ2                30.00 %
GPZ3                50.10 %
P16               1000.00 usec

F1 - Acquisition parameters
TD                    128
SFO1             50.68533 MHz
FIDRES         158.391663 Hz
SW                400.000 ppm
FnMODE                 QF

F2 - Processing parameters
SI                   2048
SF            500.1300434 MHz
WDW                  SINE
SSB       0
LB        0 Hz
GB        0
PC                   1.40

F1 - Processing parameters
SI                   1024
MC2                    QF
SF             50.6777330 MHz
WDW                States
SSB       0
LB        0 Hz
GB        0
```

Reaction mixture:

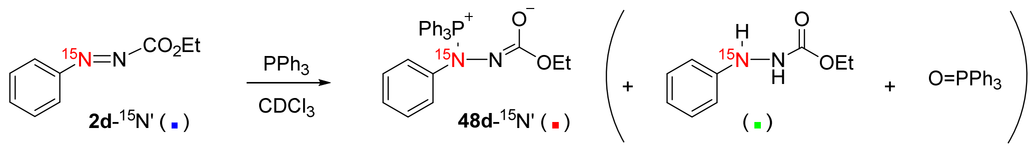

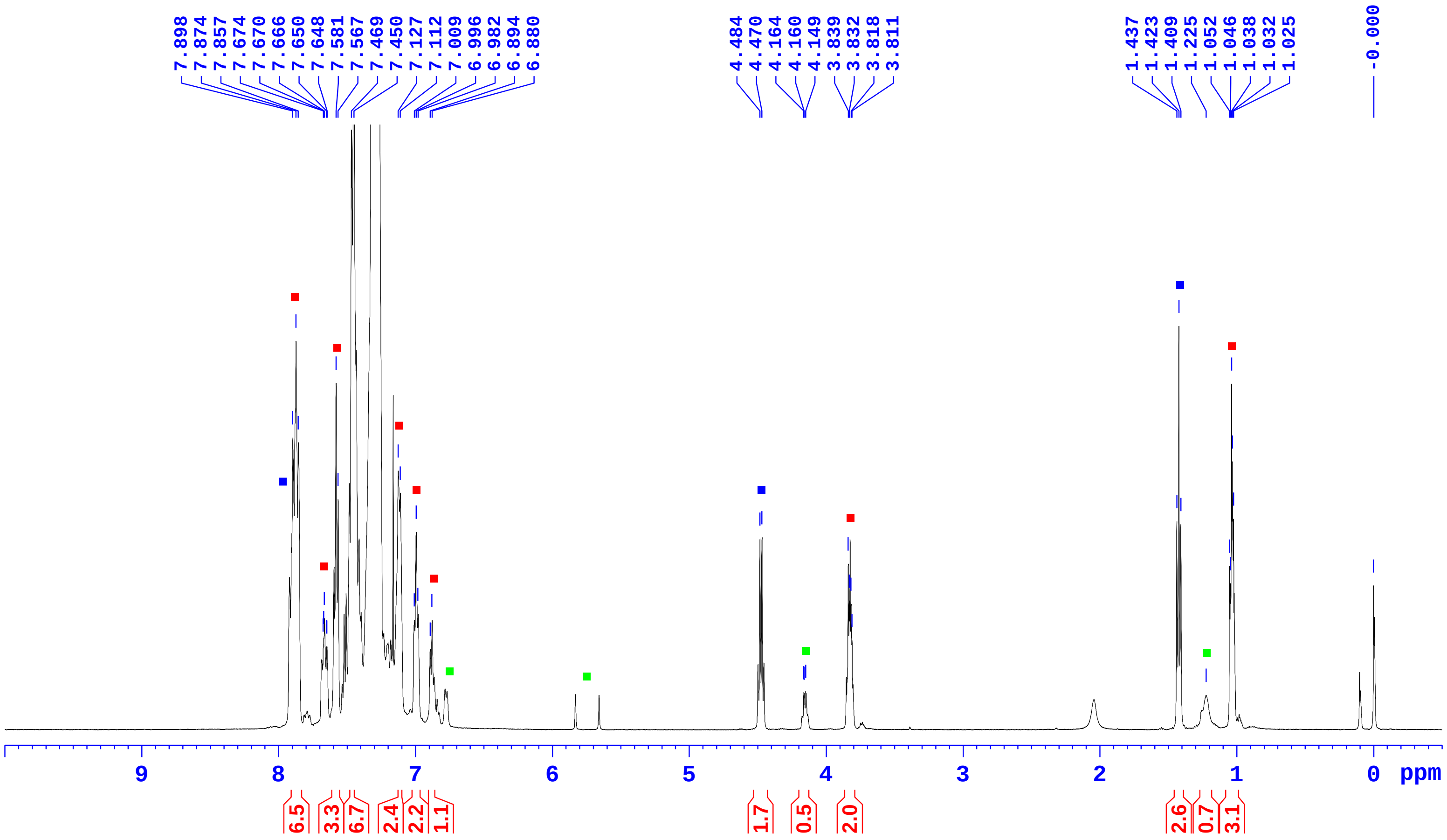


```
Current Data Parameters
NAME             MGS-614
EXPNO                  1
PROCNO                 1

F2 - Acquisition Parameters
Date_           20150730
Time               16.24
INSTRUM            spect
PROBHD   5 mm PABBO BB-
PULPROG             zg30
TD                 65536
SOLVENT            CDCl3
NS                    16
DS                     2
SWH            10330.578 Hz
FIDRES          0.157632 Hz
AQ             3.1719923 sec
RG                  20.2
DW                48.400 usec
DE                  6.50 usec
TE                 296.0 K
D1            1.00000000 sec

======== CHANNEL f1 ========
NUC1                  1H
P1                  8.90 usec
PLW1         26.00000000 W
SFO1         500.1330885 MHz

F2 - Processing parameters
SI                 65536
SF           500.1300618 MHz
WDW                   EM
SSB      0
LB                  0.30 Hz
GB       0
PC                  1.00
```

Reaction mixture:

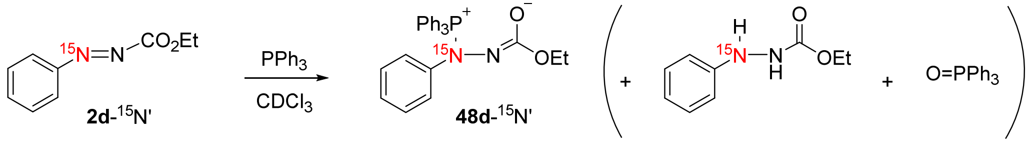

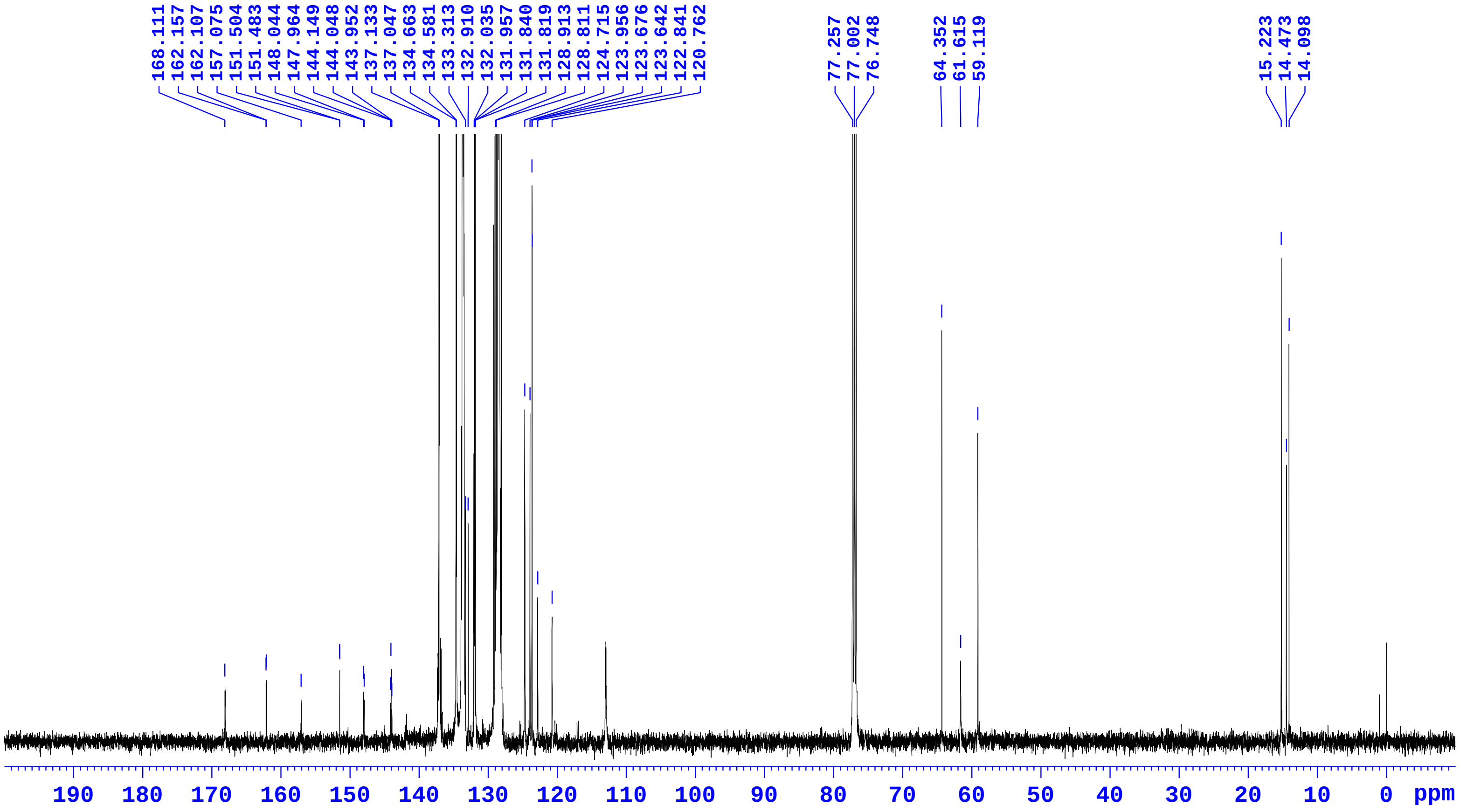


```
Current Data Parameters
NAME              MGS-614
EXPNO                  10
PROCNO                  1

F2 - Acquisition Parameters
Date_            20150731
Time                 5.35
INSTRUM             spect
PROBHD   5 mm PABBO BB-
PULPROG           zgpg30
TD                  65536
SOLVENT             CDCl3
NS                  12288
DS                      4
SWH             29761.904 Hz
FIDRES           0.454131 Hz
AQ              1.1010548 sec
RG                   2050
DW                 16.800 usec
DE                   6.50 usec
TE                  296.0 K
D1             1.00000000 sec
D11            0.03000000 sec

======== CHANNEL f1 ========
NUC1                  13C
P1                   9.00 usec
PLW1         122.00000000 W
SFO1          125.7703637 MHz

======== CHANNEL f2 ========
CPDPRG2           waltz16
NUC2                   1H
PCPD2               80.00 usec
PLW2          26.00000000 W
PLW12          0.32179001 W
PLW13          0.20595001 W
SFO2          500.1320005 MHz

F2 - Processing parameters
SI                  32768
SF            125.7578145 MHz
WDW                    EM
SSB      0
LB                   1.00 Hz
GB       0
PC                   1.40
```

Reaction mixture:

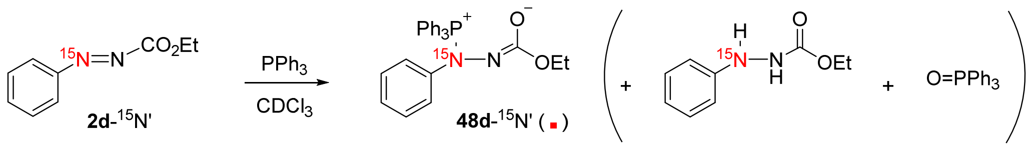

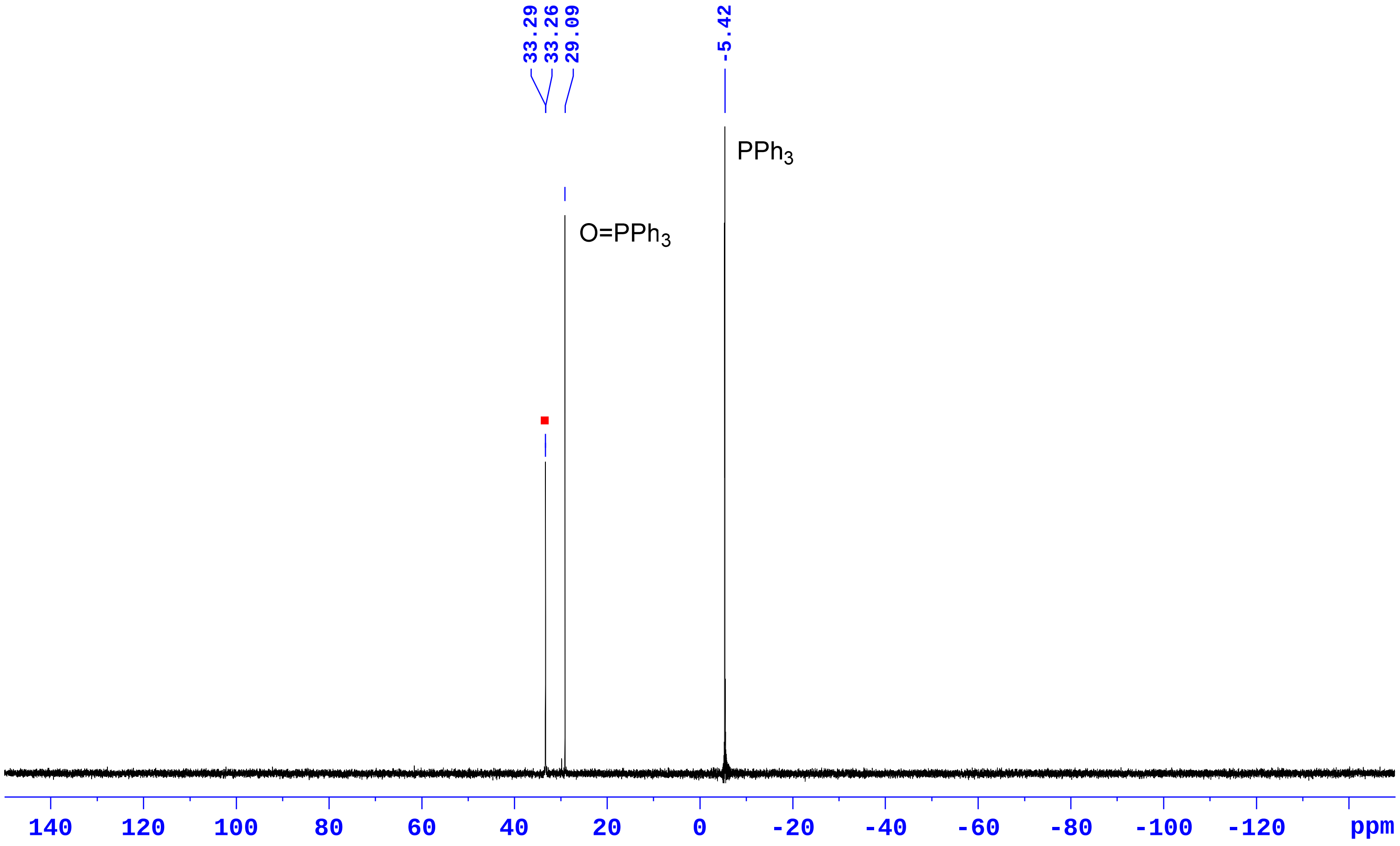


```
Current Data Parameters
NAME              MGS-614
EXPNO                   7
PROCNO                  1

F2 - Acquisition Parameters
Date_            20150730
Time                21.47
INSTRUM             spect
PROBHD    5 mm PABBO BB-
PULPROG           zgpg30
TD                  65536
SOLVENT             CDCl3
NS                     16
DS                      4
SWH            81521.742 Hz
FIDRES          1.243923 Hz
AQ             0.4020041 sec
RG                   1820
DW                  6.133 usec
DE                   6.50 usec
TE                  296.1 K
D1             2.00000000 sec
D11            0.03000000 sec

======== CHANNEL f1 ========
NUC1                  31P
P1                  10.00 usec
PLW1         100.00000000 W
SFO1          202.4462121 MHz

======== CHANNEL f2 ========
CPDPRG2           waltz16
NUC2                   1H
PCPD2               80.00 usec
PLW2          26.00000000 W
PLW12          0.32179001 W
PLW13          0.20595001 W
SFO2          500.1320005 MHz

F2 - Processing parameters
SI                  65536
SF            202.4563350 MHz
WDW                    EM
SSB       0
LB                   1.00 Hz
GB        0
PC                   1.40
```

Reaction mixture:

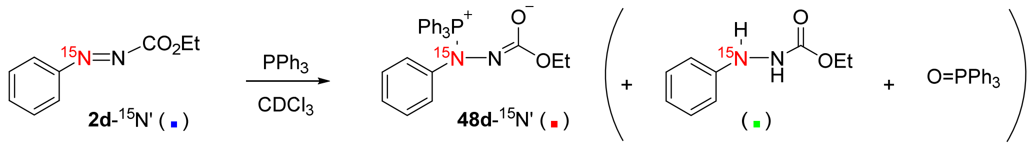

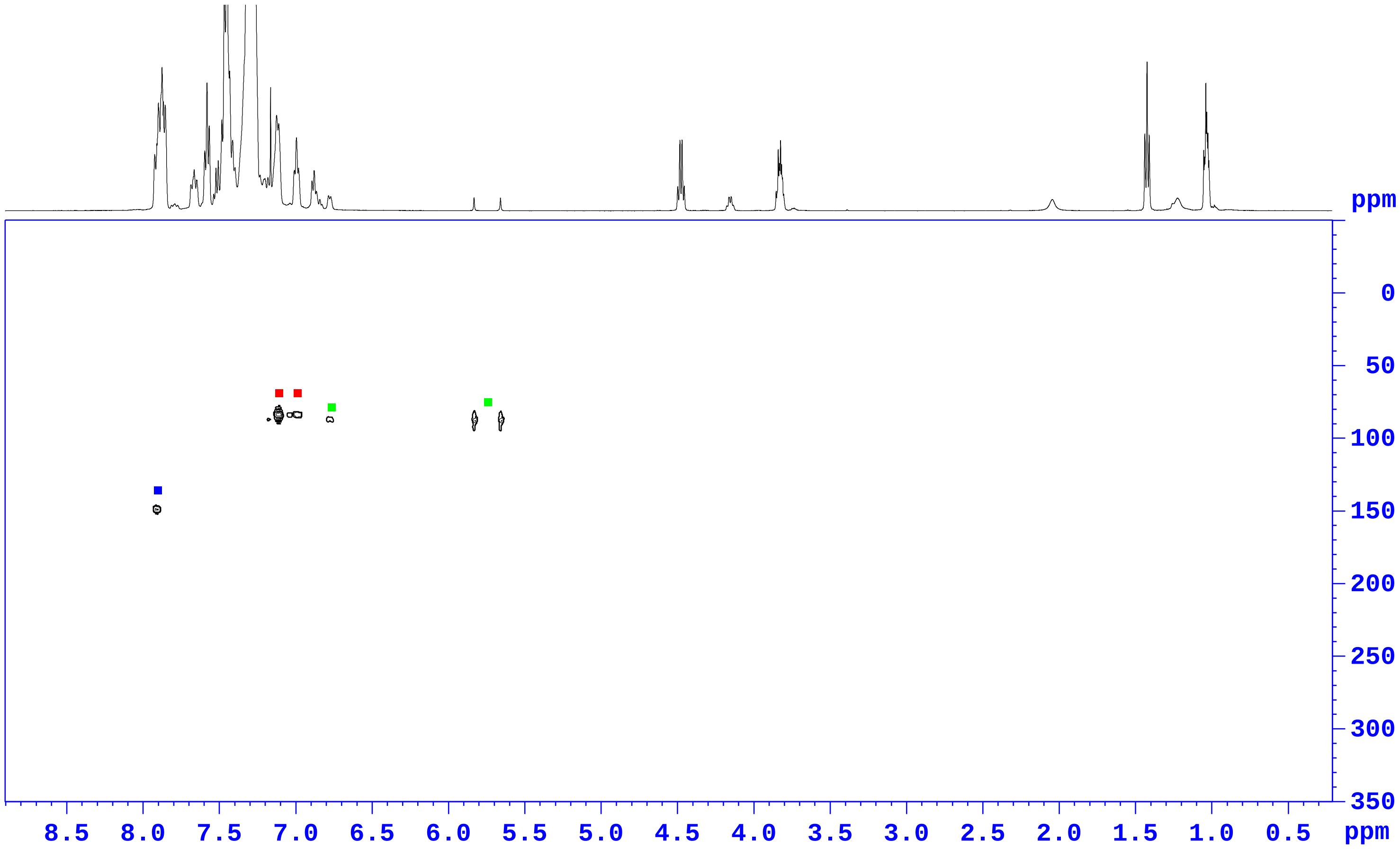


```
Current Data Parameters
NAME              MGS-614
EXPNO                   4
PROCNO                  1

F2 - Acquisition Parameters
Date_            20150730
Time                16.35
INSTRUM             spect
PROBHD    5 mm PABBO BB-
PULPROG        hmbcgpndqf
TD                   2048
SOLVENT             CDCl3
NS                     64
DS                     16
SWH              4347.826 Hz
FIDRES           2.122962 Hz
AQ              0.2355700 sec
RG                   2050
DW                115.000 usec
DE                   6.50 usec
TE                  296.0 K
CNST13          5.0000000
D0             0.00000300 sec
D1             1.92176604 sec
D6             0.10000000 sec
D16            0.00020000 sec
IN0            0.00002465 sec

======== CHANNEL f1 ========
NUC1                   1H
P1                   8.90 usec
P2                  17.80 usec
PLW1          26.00000000 W
SFO1          500.1323407 MHz

======== CHANNEL f2 ========
NUC2                  15N
P3                  14.40 usec
PLW2         206.00000000 W
SFO2           50.6853342 MHz

====== GRADIENT CHANNEL =====
GPNAM1         SMSQ10.100
GPNAM2         SMSQ10.100
GPNAM3         SMSQ10.100
GPZ1                70.00 %
GPZ2                30.00 %
GPZ3                50.10 %
P16               1000.00 usec

F1 - Acquisition parameters
TD                    128
SFO1             50.68533 MHz
FIDRES         158.391663 Hz
SW                400.000 ppm
FnMODE                 QF

F2 - Processing parameters
SI                   2048
SF            500.1300618 MHz
WDW                  SINE
SSB       0
LB        0 Hz
GB        0
PC                   1.40

F1 - Processing parameters
SI                   1024
MC2                    QF
SF             50.6777330 MHz
WDW          echo-antiecho
SSB       0
LB        0 Hz
GB        0
```

Reaction mixture:

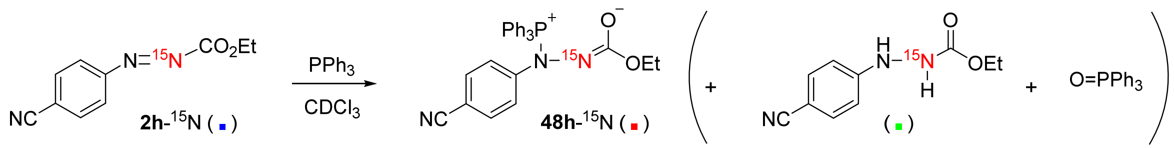

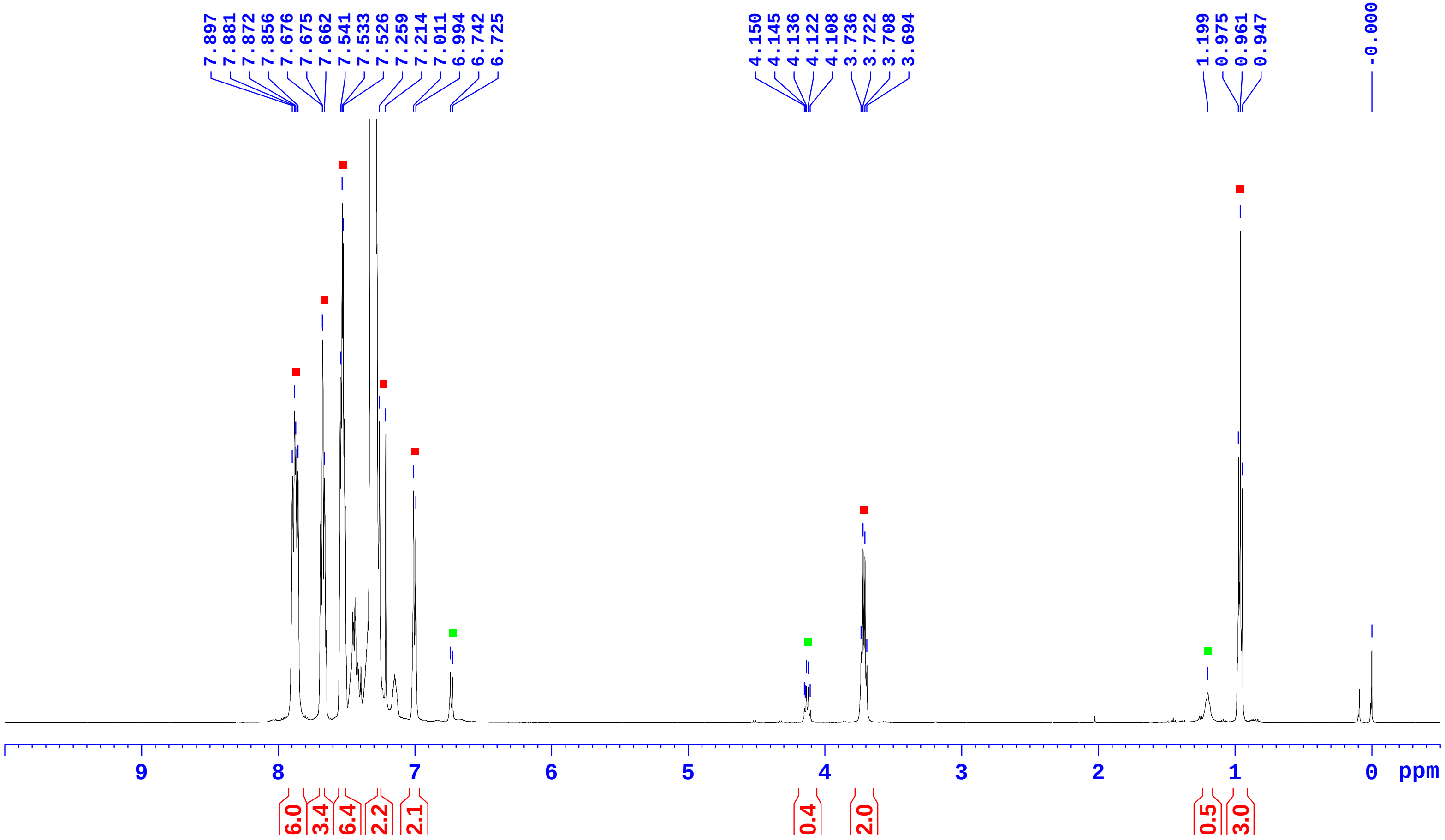


```
Current Data Parameters
NAME             MGS-651
EXPNO                  1
PROCNO                 1

F2 - Acquisition Parameters
Date_           20151021
Time               17.01
INSTRUM            spect
PROBHD   5 mm PABBO BB-
PULPROG             zg30
TD                 65536
SOLVENT            CDCl3
NS                    16
DS                     2
SWH          10330.578 Hz
FIDRES        0.157632 Hz
AQ           3.1719923 sec
RG                    32
DW              48.400 usec
DE                6.50 usec
TE               296.0 K
D1          1.00000000 sec

======== CHANNEL f1 ========
NUC1                  1H
P1                  8.90 usec
PLW1         26.00000000 W
SFO1         500.1330885 MHz

F2 - Processing parameters
SI                 65536
SF           500.1300365 MHz
WDW                   EM
SSB      0
LB                  0.30 Hz
GB       0
PC                  1.00
```

Reaction mixture:

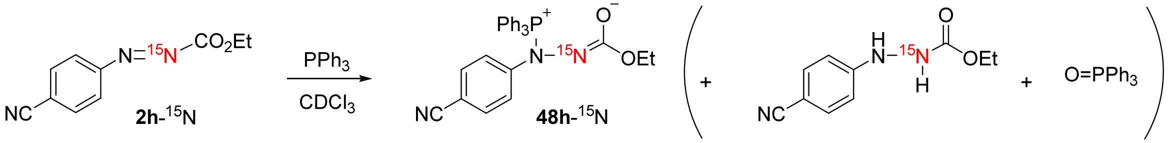

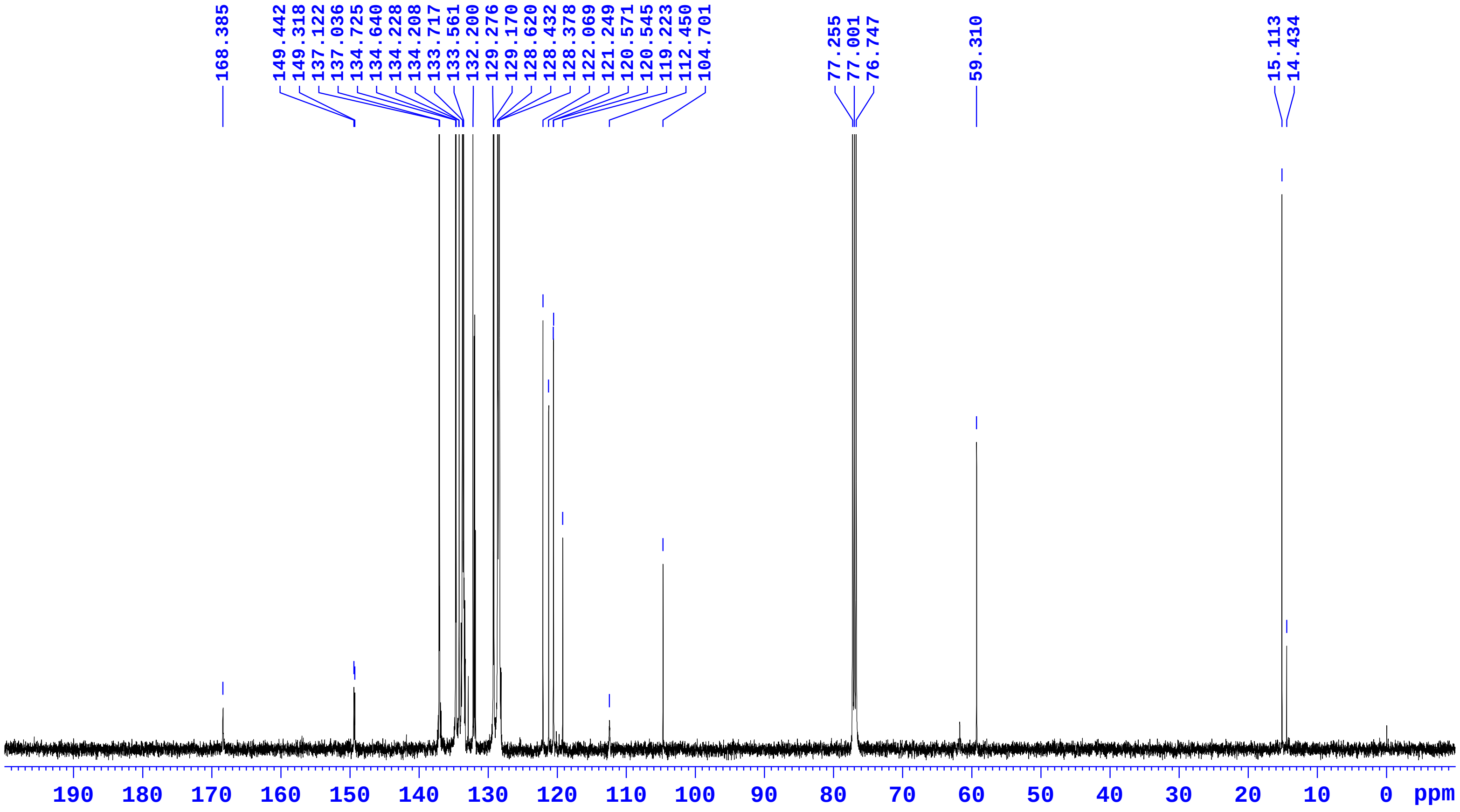


```
Current Data Parameters
NAME                MGS-651
EXPNO                     7
PROCNO                    1

F2 - Acquisition Parameters
Date_              20151021
Time                  20.38
INSTRUM               spect
PROBHD   5 mm PABBO BB-
PULPROG             zgpg30
TD                    65536
SOLVENT               CDCl3
NS                     4096
DS                        4
SWH               29761.904 Hz
FIDRES             0.454131 Hz
AQ                1.1010548 sec
RG                     2050
DW                   16.800 usec
DE                     6.50 usec
TE                    296.0 K
D1               1.00000000 sec
D11              0.03000000 sec

======== CHANNEL f1 ========
NUC1                    13C
P1                     9.00 usec
PLW1           122.00000000 W
SFO1            125.7703637 MHz

======== CHANNEL f2 ========
CPDPRG2             waltz16
NUC2                     1H
PCPD2                 80.00 usec
PLW2            26.00000000 W
PLW12            0.32179001 W
PLW13            0.20595001 W
SFO2            500.1320005 MHz

F2 - Processing parameters
SI                    32768
SF              125.7578087 MHz
WDW                      EM
SSB      0
LB                     1.00 Hz
GB       0
PC                     1.40
```

Reaction mixture:

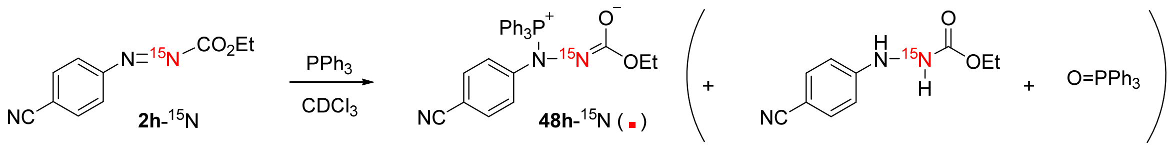

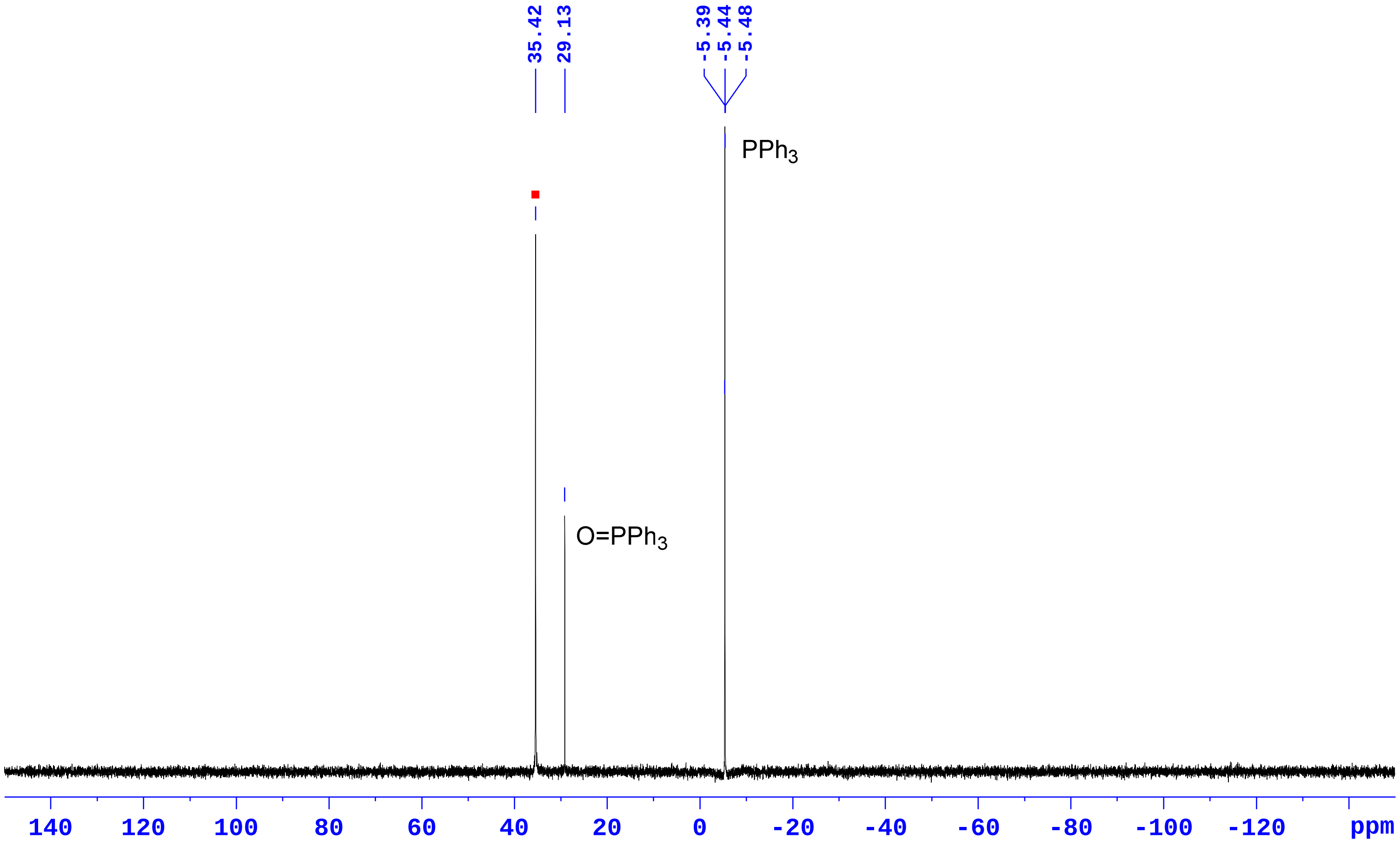


```
Current Data Parameters
NAME             MGS-651
EXPNO                  3
PROCNO                 1

F2 - Acquisition Parameters
Date_           20151021
Time               17.06
INSTRUM            spect
PROBHD   5 mm PABBO BB-
PULPROG          zgpg30
TD                 65536
SOLVENT            CDCl3
NS                    16
DS                     4
SWH            81521.742 Hz
FIDRES          1.243923 Hz
AQ             0.4020041 sec
RG                  2050
DW                 6.133 usec
DE                  6.50 usec
TE                 296.1 K
D1            2.00000000 sec
D11           0.03000000 sec

======== CHANNEL f1 ========
NUC1                 31P
P1                 10.00 usec
PLW1        100.00000000 W
SFO1         202.4462121 MHz

======== CHANNEL f2 ========
CPDPRG2          waltz16
NUC2                  1H
PCPD2              80.00 usec
PLW2         26.00000000 W
PLW12         0.32179001 W
PLW13         0.20595001 W
SFO2         500.1320005 MHz

F2 - Processing parameters
SI                 32768
SF           202.4563350 MHz
WDW                   EM
SSB      0
LB                  1.00 Hz
GB       0
PC                  1.40
```

Reaction mixture:

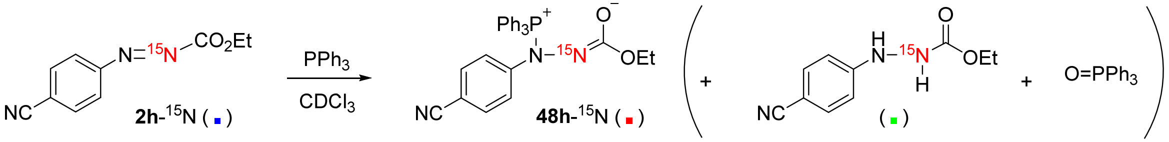

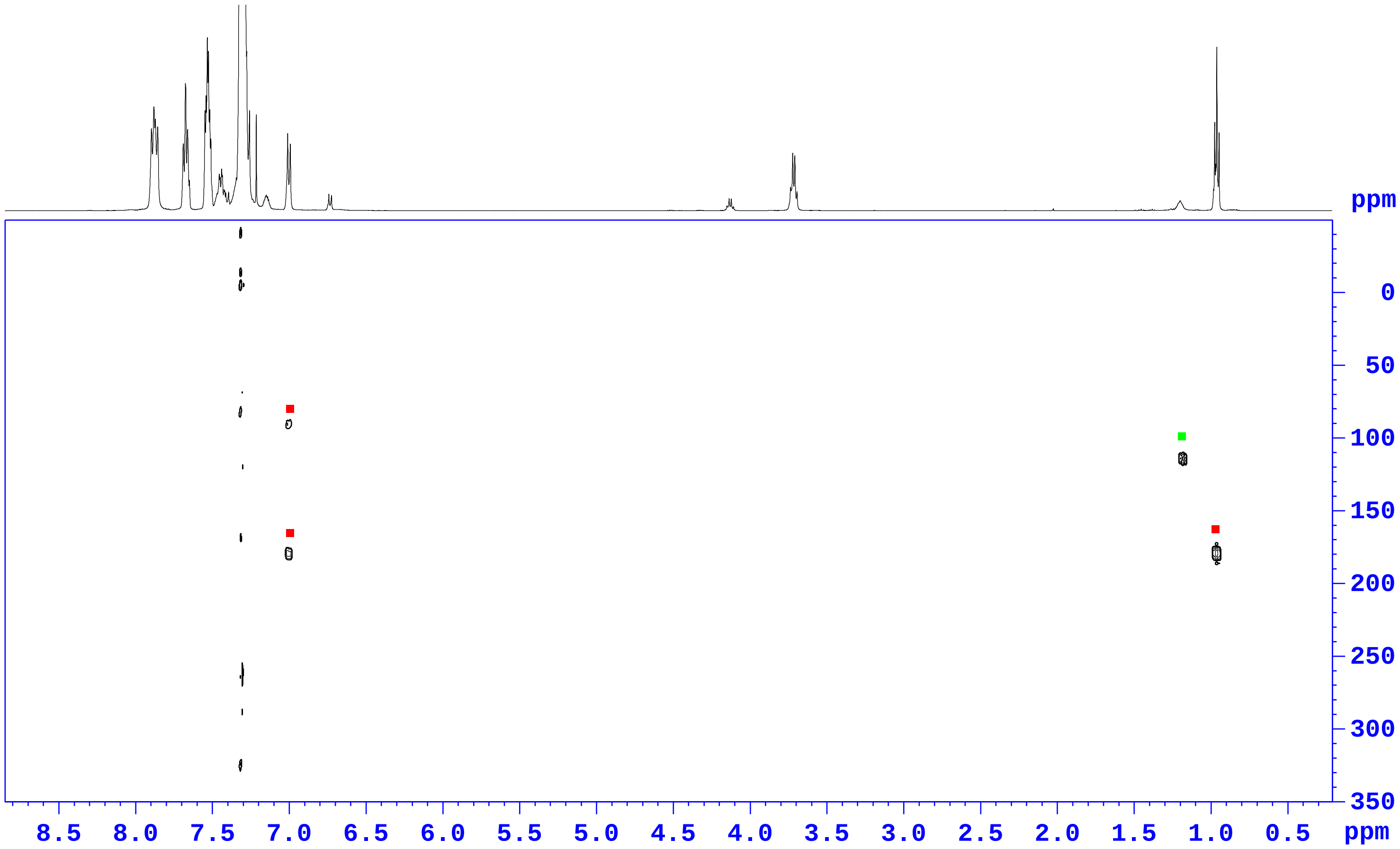


```
Current Data Parameters
NAME                MGS-651
EXPNO                     4
PROCNO                    1

F2 - Acquisition Parameters
Date_              20151021
Time                  17.09
INSTRUM               spect
PROBHD   5 mm PABBO BB-
PULPROG          hmbcgpndqf
TD                     2048
SOLVENT               CDCl3
NS                       16
DS                       16
SWH                4385.965 Hz
FIDRES             2.141584 Hz
AQ                0.2335220 sec
RG                     2050
DW                  114.000 usec
DE                     6.50 usec
TE                    296.0 K
CNST13            5.0000000
D0               0.00000300 sec
D1               1.92381406 sec
D6               0.10000000 sec
D16              0.00020000 sec
IN0              0.00002465 sec

======== CHANNEL f1 ========
NUC1                     1H
P1                     8.90 usec
P2                    17.80 usec
PLW1            26.00000000 W
SFO1            500.1322693 MHz

======== CHANNEL f2 ========
NUC2                    15N
P3                    14.40 usec
PLW2           206.00000000 W
SFO2             50.6853342 MHz

====== GRADIENT CHANNEL =====
GPNAM1           SMSQ10.100
GPNAM2           SMSQ10.100
GPNAM3           SMSQ10.100
GPZ1                  70.00 %
GPZ2                  30.00 %
GPZ3                  50.10 %
P16                 1000.00 usec

F1 - Acquisition parameters
TD                       57
SFO1               50.68533 MHz
FIDRES           355.686554 Hz
SW                  400.000 ppm
FnMODE                   QF

F2 - Processing parameters
SI                     2048
SF              500.1300365 MHz
WDW                    SINE
SSB      0
LB       0 Hz
GB       0
PC                     1.40

F1 - Processing parameters
SI                     1024
MC2                      QF
SF               50.6777330 MHz
WDW                  States
SSB      0
LB       0 Hz
GB       0
```

Reaction mixture:

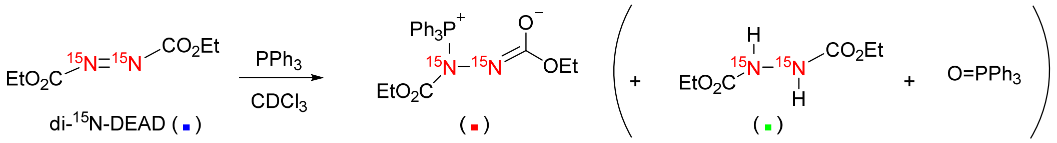

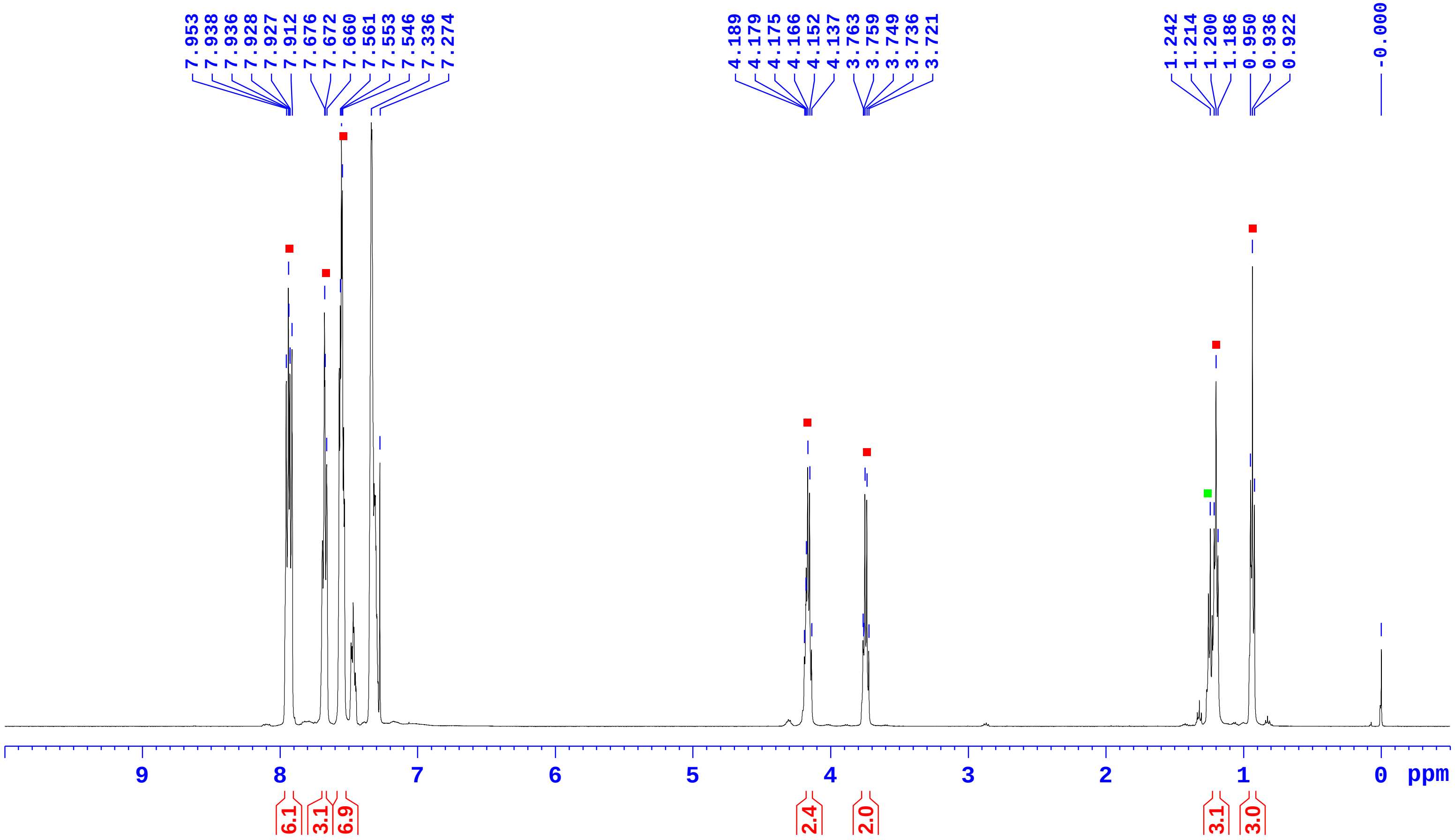


```
Current Data Parameters
NAME              MGS-659
EXPNO                   1
PROCNO                  1

F2 - Acquisition Parameters
Date_            20151027
Time                17.21
INSTRUM             spect
PROBHD   5 mm PABBO BB-
PULPROG              zg30
TD                  65536
SOLVENT             CDCl3
NS                     16
DS                      2
SWH             10330.578 Hz
FIDRES           0.157632 Hz
AQ              3.1719923 sec
RG                   80.6
DW                 48.400 usec
DE                   6.50 usec
TE                  296.0 K
D1             1.00000000 sec

======== CHANNEL f1 ========
NUC1                   1H
P1                   8.90 usec
PLW1          26.00000000 W
SFO1          500.1330885 MHz

F2 - Processing parameters
SI                  65536
SF            500.1300069 MHz
WDW                    EM
SSB      0
LB                   0.30 Hz
GB       0
PC                   1.00
```

Reaction mixture:

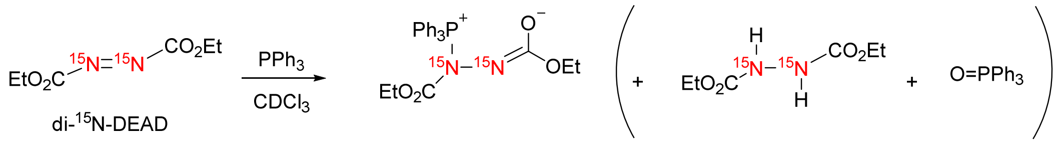

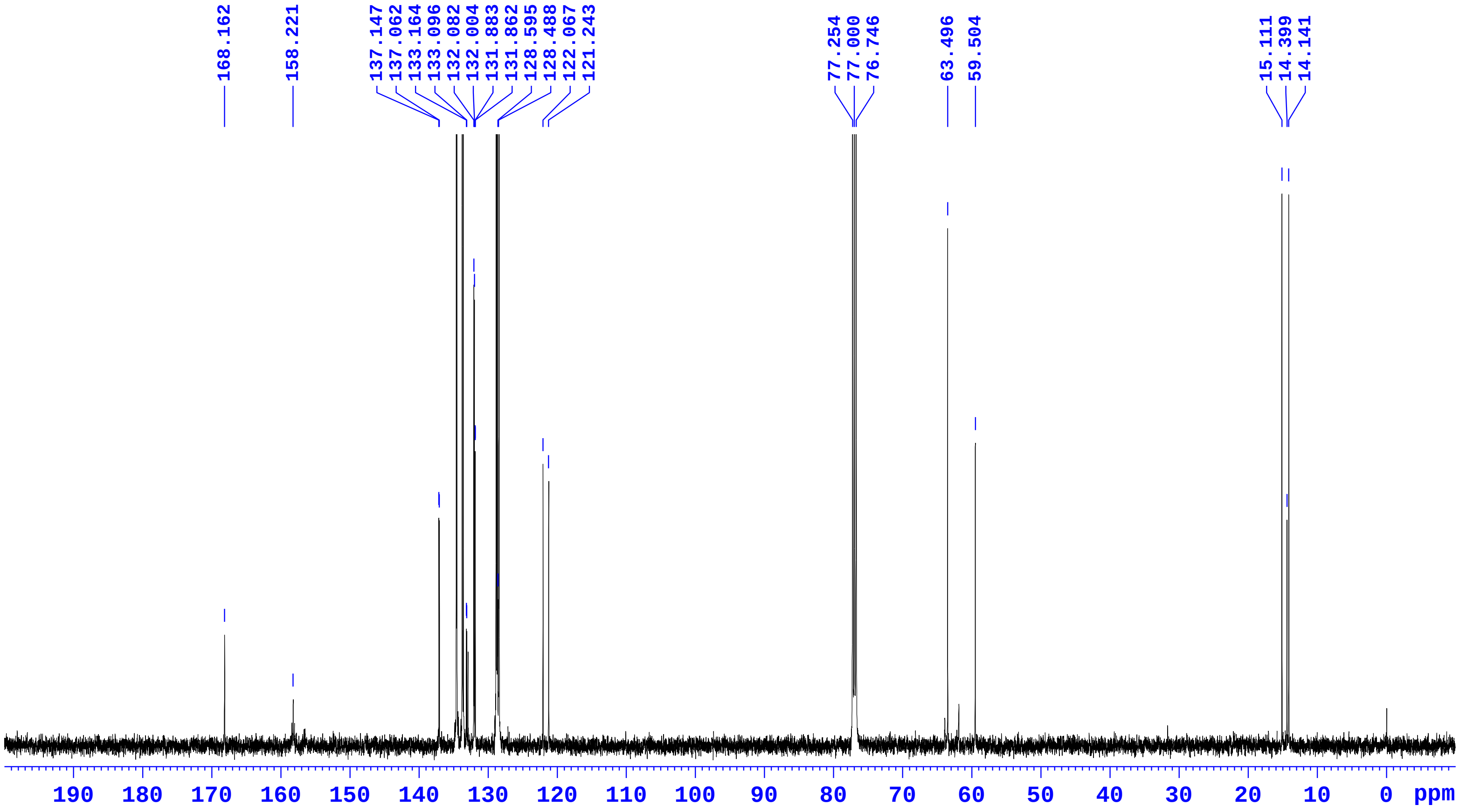


```
Current Data Parameters
NAME               MGS-659
EXPNO                   11
PROCNO                   1

F2 - Acquisition Parameters
Date_             20151027
Time                 20.22
INSTRUM              spect
PROBHD   5 mm PABBO BB-
PULPROG             zgpg30
TD                   65536
SOLVENT              CDCl3
NS                    3072
DS                       4
SWH              29761.904 Hz
FIDRES            0.454131 Hz
AQ               1.1010548 sec
RG                    2050
DW                  16.800 usec
DE                    6.50 usec
TE                   296.0 K
D1              1.00000000 sec
D11             0.03000000 sec

======== CHANNEL f1 ========
NUC1                   13C
P1                    9.00 usec
PLW1          122.00000000 W
SFO1           125.7703637 MHz

======== CHANNEL f2 ========
CPDPRG2             waltz16
NUC2                     1H
PCPD2                 80.00 usec
PLW2            26.00000000 W
PLW12            0.32179001 W
PLW13            0.20595001 W
SFO2            500.1320005 MHz

F2 - Processing parameters
SI                    32768
SF              125.7577970 MHz
WDW                      EM
SSB      0
LB                     1.00 Hz
GB       0
PC                     1.40
```

Reaction mixture:

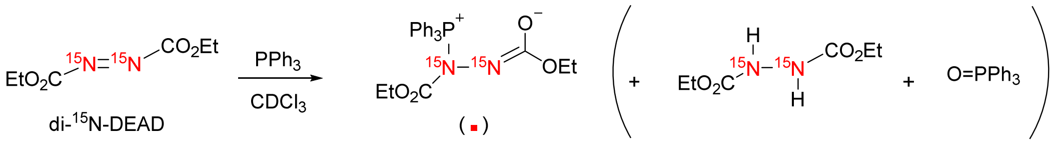

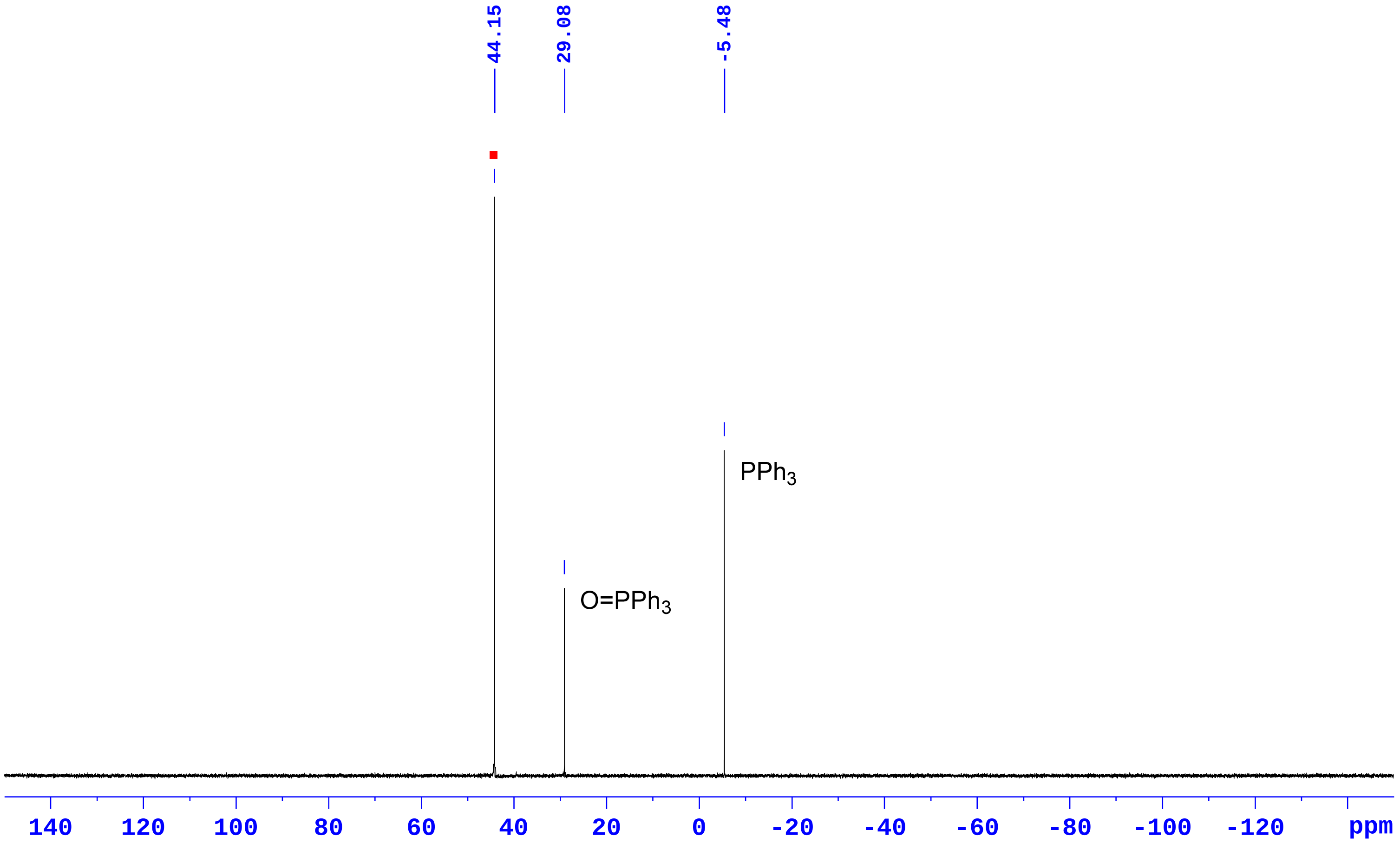


```
Current Data Parameters
NAME              MGS-659
EXPNO                   4
PROCNO                  1

F2 - Acquisition Parameters
Date_            20151027
Time                17.32
INSTRUM             spect
PROBHD    5 mm PABBO BB-
PULPROG           zgpg30
TD                  65536
SOLVENT             CDCl3
NS                     32
DS                      4
SWH           81521.742 Hz
FIDRES         1.243923 Hz
AQ            0.4020041 sec
RG                   2050
DW                  6.133 usec
DE                   6.50 usec
TE                  296.0 K
D1             2.00000000 sec
D11            0.03000000 sec

======== CHANNEL f1 ========
NUC1                  31P
P1                  10.00 usec
PLW1         100.00000000 W
SFO1          202.4462121 MHz

======== CHANNEL f2 ========
CPDPRG2           waltz16
NUC2                   1H
PCPD2               80.00 usec
PLW2          26.00000000 W
PLW12          0.32179001 W
PLW13          0.20595001 W
SFO2          500.1320005 MHz

F2 - Processing parameters
SI                  32768
SF            202.4563350 MHz
WDW                    EM
SSB       0
LB                   1.00 Hz
GB        0
PC                   1.40
```

Reaction mixture:

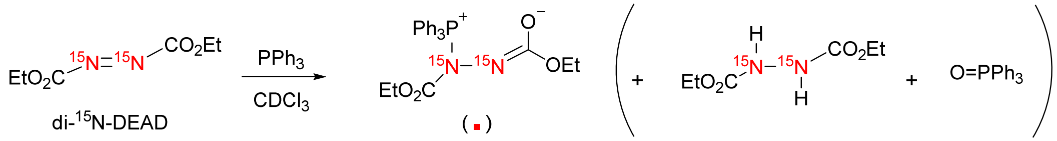

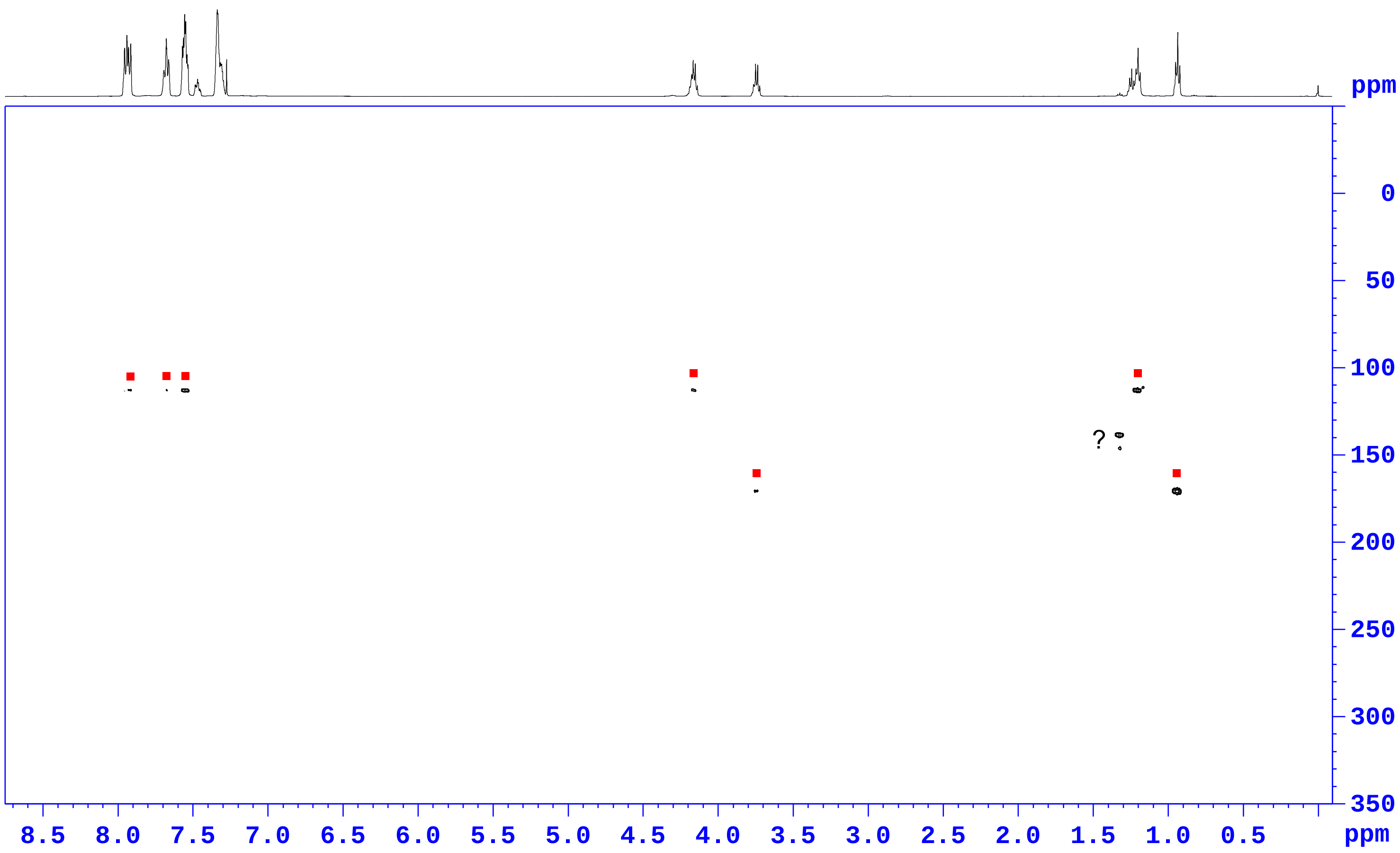


```
Current Data Parameters
NAME               MGS-659
EXPNO                   15
PROCNO                   1

F2 - Acquisition Parameters
Date_             20151028
Time                  0.43
INSTRUM              spect
PROBHD   5 mm PABBO BB-
PULPROG         hmbcgpndqf
TD                    2048
SOLVENT              CDCl3
NS                      16
DS                      16
SWH               4424.779 Hz
FIDRES            2.160537 Hz
AQ               0.2314740 sec
RG                    2050
DW                 113.000 usec
DE                    6.50 usec
TE                   296.0 K
CNST13           5.0000000
D0              0.00000300 sec
D1              1.92586195 sec
D6              0.10000000 sec
D16             0.00020000 sec
IN0             0.00002465 sec

======== CHANNEL f1 ========
NUC1                    1H
P1                    8.90 usec
P2                   17.80 usec
PLW1           26.00000000 W
SFO1           500.1321725 MHz

======== CHANNEL f2 ========
NUC2                   15N
P3                   14.40 usec
PLW2          206.00000000 W
SFO2            50.6853342 MHz

====== GRADIENT CHANNEL =====
GPNAM1         SMSQ10.100
GPNAM2         SMSQ10.100
GPNAM3         SMSQ10.100
GPZ1                70.00 %
GPZ2                30.00 %
GPZ3                50.10 %
P16               1000.00 usec

F1 - Acquisition parameters
TD                     256
SFO1              50.68533 MHz
FIDRES           79.195831 Hz
SW                 400.000 ppm
FnMODE                  QF

F2 - Processing parameters
SI                    2048
SF             500.1300073 MHz
WDW                   SINE
SSB       0
LB        0 Hz
GB        0
PC                    1.40

F1 - Processing parameters
SI                    1024
MC2                     QF
SF              50.6777330 MHz
WDW           echo-antiecho
SSB       0
LB        0 Hz
GB        0
```

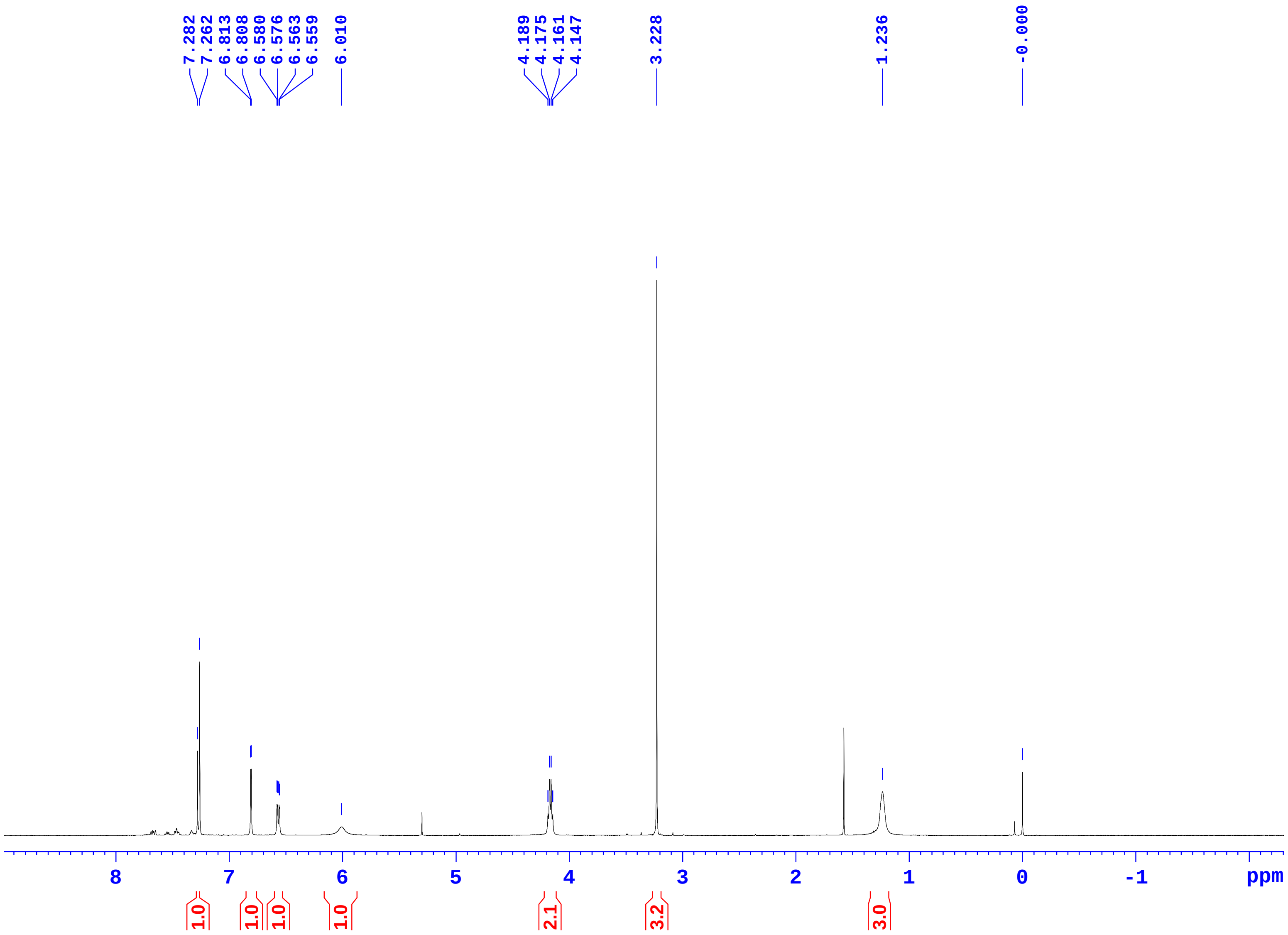


```
Current Data Parameters
NAME              mgs-634
EXPNO                  50
PROCNO                  1

F2 - Acquisition Parameters
Date_            20151028
Time                18.31
INSTRUM             spect
PROBHD   5 mm PABBO BB-
PULPROG              zg30
TD                  65536
SOLVENT             CDCl3
NS                     16
DS                      2
SWH           10330.578 Hz
FIDRES         0.157632 Hz
AQ            3.1719923 sec
RG                    161
DW               48.400 usec
DE                 6.50 usec
TE                296.0 K
D1           1.00000000 sec

======== CHANNEL f1 ========
NUC1                   1H
P1                 8.90 usec
PLW1        26.00000000 W
SFO1        500.1330885 MHz

F2 - Processing parameters
SI                  65536
SF          500.1300126 MHz
WDW                    EM
SSB      0
LB                   0.30 Hz
GB       0
PC                   1.00
```

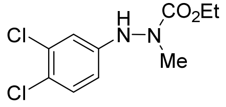

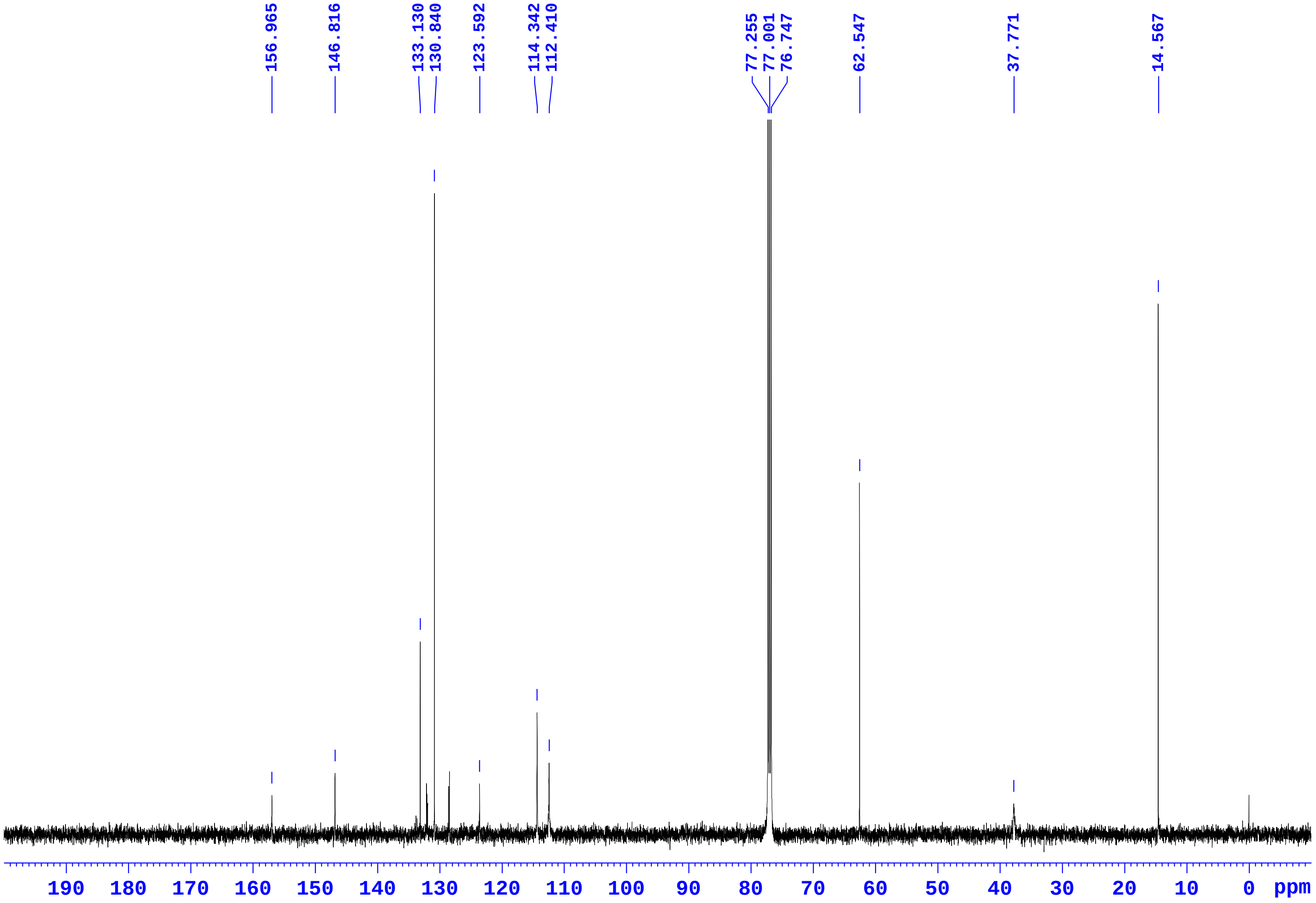


```
Current Data Parameters
NAME              mgs-634
EXPNO                  52
PROCNO                  1

F2 - Acquisition Parameters
Date_            20151028
Time                21.20
INSTRUM             spect
PROBHD    5 mm PABBO BB-
PULPROG           zgpg30
TD                  65536
SOLVENT             CDCl3
NS                   4096
DS                      4
SWH            29761.904 Hz
FIDRES          0.454131 Hz
AQ             1.1010548 sec
RG                   2050
DW                 16.800 usec
DE                   6.50 usec
TE                  296.0 K
D1             1.00000000 sec
D11            0.03000000 sec

======== CHANNEL f1 ========
NUC1                  13C
P1                   9.00 usec
PLW1         122.00000000 W
SFO1          125.7703637 MHz

======== CHANNEL f2 ========
CPDPRG2           waltz16
NUC2                   1H
PCPD2               80.00 usec
PLW2          26.00000000 W
PLW12          0.32179001 W
PLW13          0.20595001 W
SFO2          500.1320005 MHz

F2 - Processing parameters
SI                  32768
SF            125.7577928 MHz
WDW                    EM
SSB       0
LB                   1.00 Hz
GB        0
PC                   1.40
```

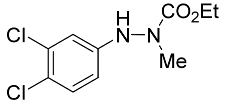

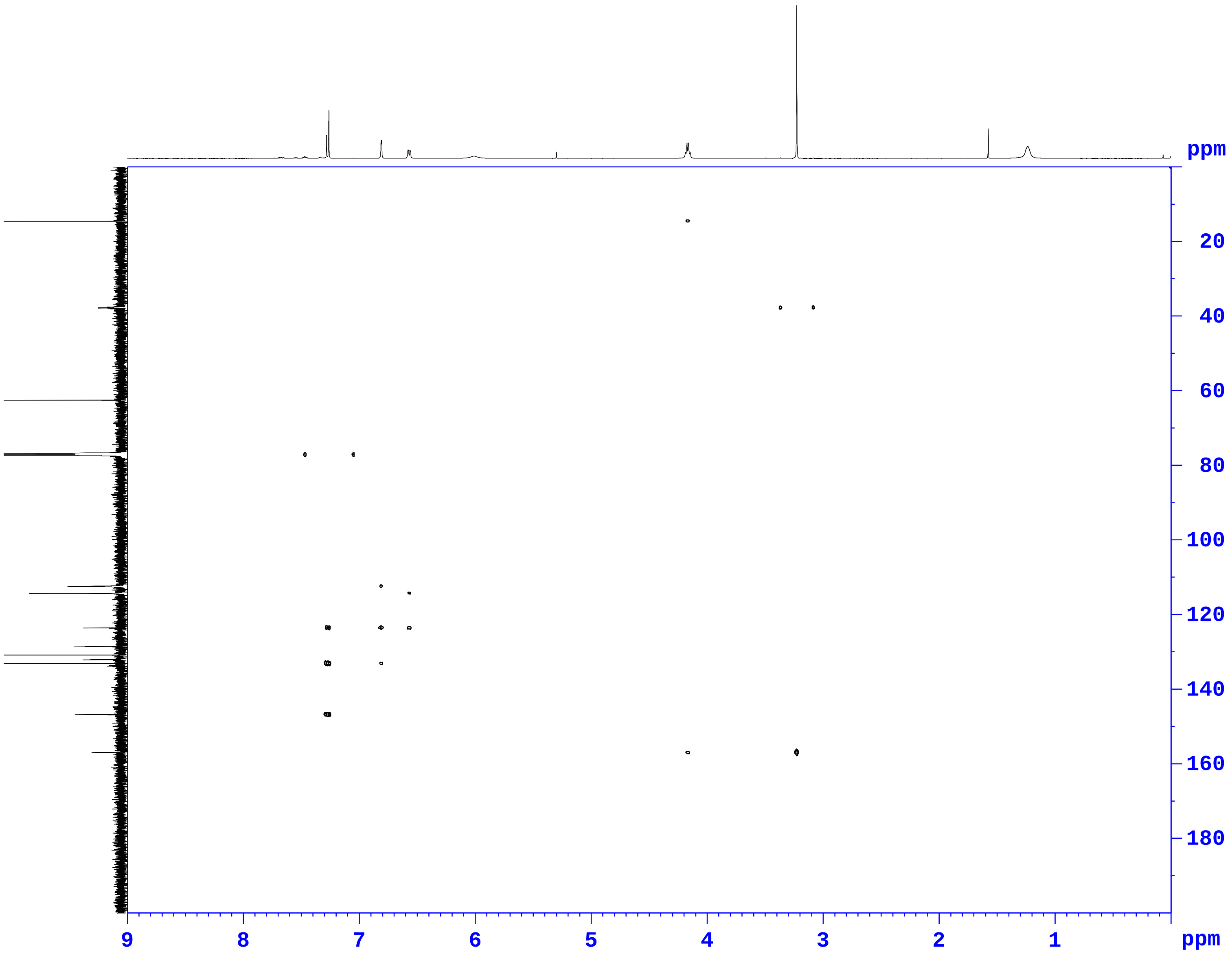


```
Current Data Parameters
NAME              mgs-634
EXPNO                  54
PROCNO                  1

F2 - Acquisition Parameters
Date_            20151028
Time                21.51
INSTRUM             spect
PROBHD   5 mm PABBO BB-
PULPROG    hmbcgplpndqf
TD                   2048
SOLVENT             CDCl3
NS                     16
DS                     16
SWH              4424.779 Hz
FIDRES           2.160537 Hz
AQ              0.2314740 sec
RG                   2050
DW                113.000 usec
DE                   6.50 usec
TE                  296.0 K
CNST2         145.0000000
CNST13         10.0000000
D0             0.00000300 sec
D1             1.42586195 sec
D2             0.00344828 sec
D6             0.05000000 sec
D16            0.00020000 sec
IN0            0.00001790 sec

======== CHANNEL f1 ========
NUC1                   1H
P1                   8.90 usec
P2                  17.80 usec
PLW1          26.00000000 W
SFO1          500.1320274 MHz

======== CHANNEL f2 ========
NUC2                  13C
P3                   9.00 usec
PLW2         122.00000000 W
SFO2          125.7703437 MHz

====== GRADIENT CHANNEL =====
GPNAM1         SMSQ10.100
GPNAM2         SMSQ10.100
GPNAM3         SMSQ10.100
GPZ1                50.00 %
GPZ2                30.00 %
GPZ3                40.10 %
P16               1000.00 usec

F1 - Acquisition parameters
TD                    256
SFO1             125.7703 MHz
FIDRES         109.113174 Hz
SW                222.095 ppm
FnMODE                 QF

F2 - Processing parameters
SI                   2048
SF            500.1300126 MHz
WDW                  SINE
SSB        0
LB         0 Hz
GB         0
PC                   1.40

F1 - Processing parameters
SI                   1024
MC2                    QF
SF            125.7577890 MHz
WDW                States
SSB        0
LB         0 Hz
GB         0
```

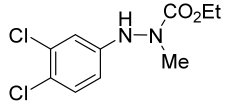

Reaction mixture:

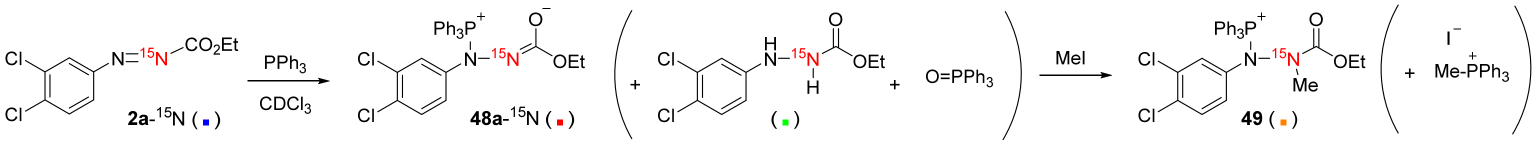

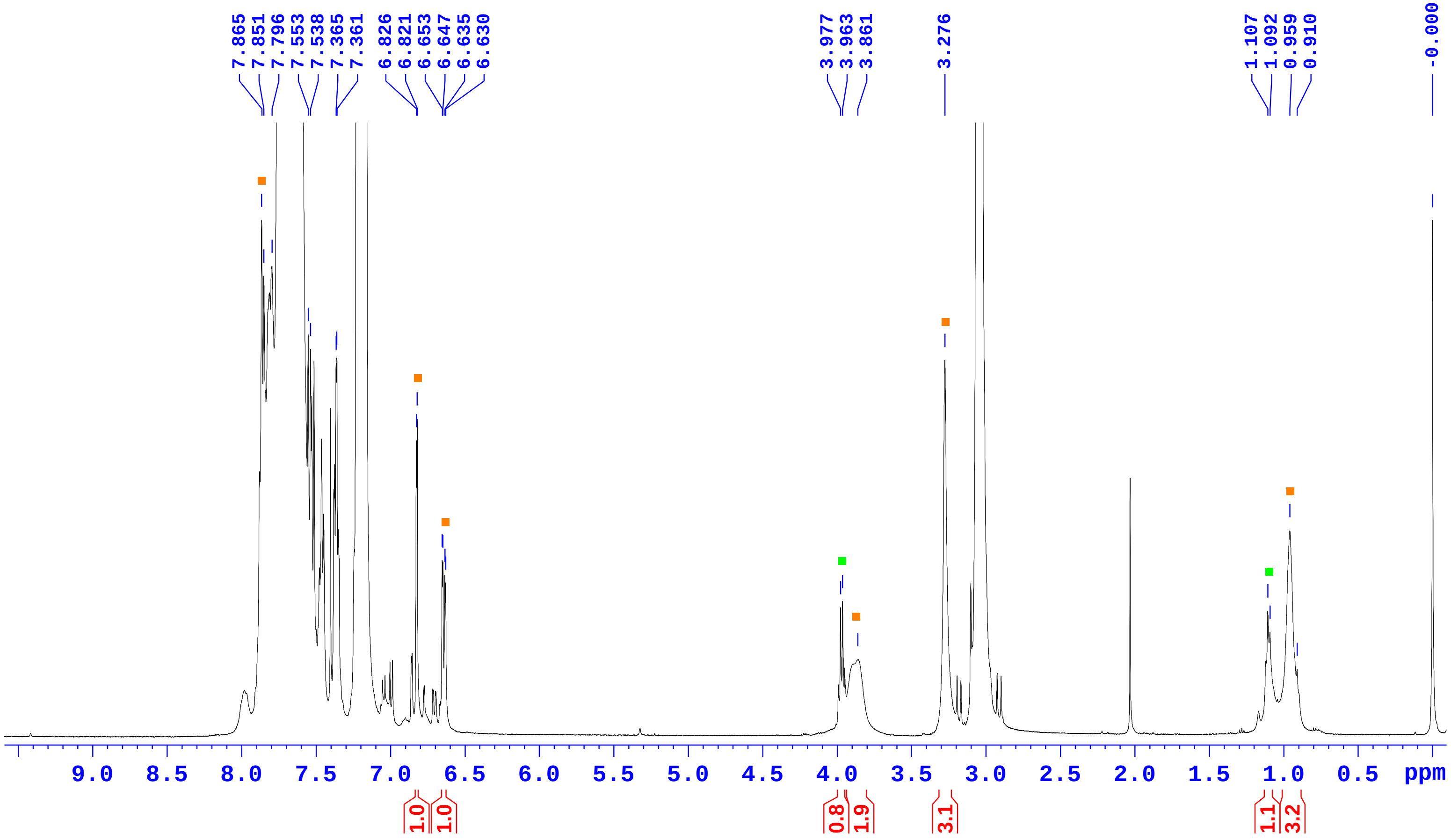


```
Current Data Parameters
NAME              MGS-620
EXPNO                   6
PROCNO                  1

F2 - Acquisition Parameters
Date_            20150808
Time                16.09
INSTRUM             spect
PROBHD   5 mm PABBO BB-
PULPROG              zg30
TD                  65536
SOLVENT             CDCl3
NS                     16
DS                      2
SWH             10330.578 Hz
FIDRES           0.157632 Hz
AQ              3.1719923 sec
RG                     32
DW                 48.400 usec
DE                   6.50 usec
TE                  299.5 K
D1             1.00000000 sec

======== CHANNEL f1 ========
NUC1                   1H
P1                   8.90 usec
PLW1          26.00000000 W
SFO1          500.1330885 MHz

F2 - Processing parameters
SI                  65536
SF            500.1299483 MHz
WDW                    EM
SSB      0
LB                   0.30 Hz
GB       0
PC                   1.00
```

Reaction mixture:

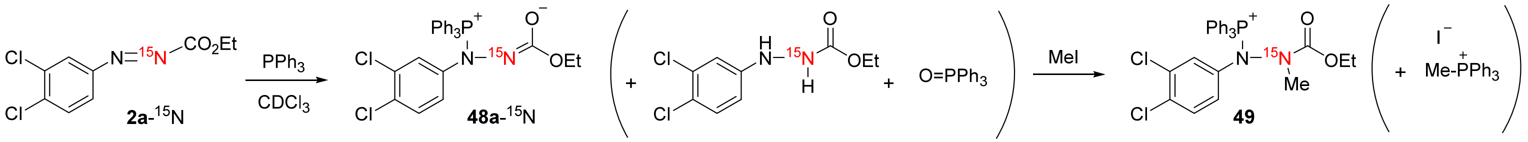

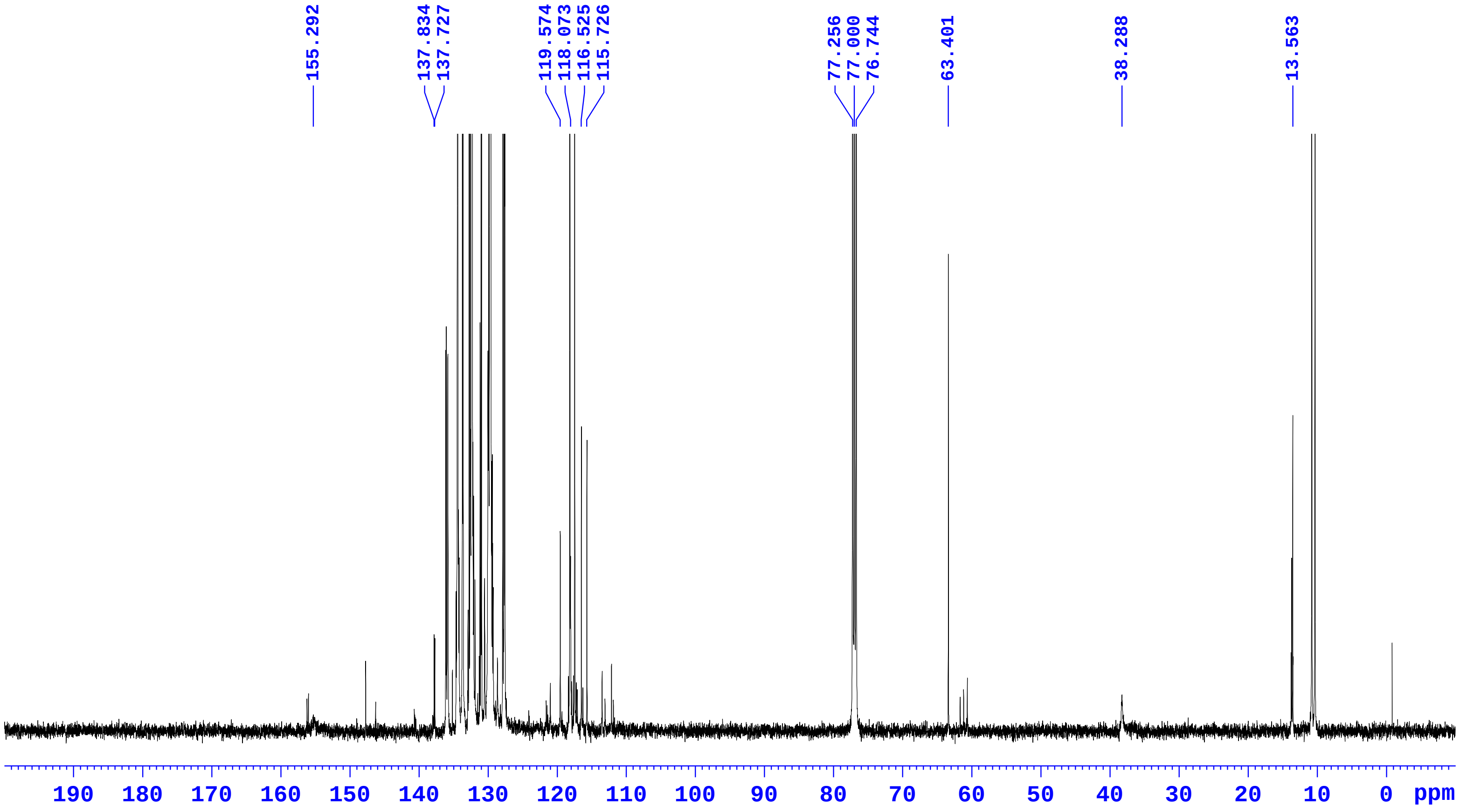


```
Current Data Parameters
NAME              MGS-620
EXPNO                  13
PROCNO                  1

F2 - Acquisition Parameters
Date_            20150809
Time                14.55
INSTRUM             spect
PROBHD   5 mm PABBO BB-
PULPROG           zgpg30
TD                 65536
SOLVENT            CDCl3
NS                  8192
DS                     4
SWH            29761.904 Hz
FIDRES          0.454131 Hz
AQ             1.1010548 sec
RG                  2050
DW                16.800 usec
DE                  6.50 usec
TE                 300.5 K
D1            1.00000000 sec
D11           0.03000000 sec

======== CHANNEL f1 ========
NUC1                 13C
P1                  9.00 usec
PLW1        122.00000000 W
SFO1         125.7703637 MHz

======== CHANNEL f2 ========
CPDPRG2          waltz16
NUC2                  1H
PCPD2              80.00 usec
PLW2         26.00000000 W
PLW12         0.32179001 W
PLW13         0.20595001 W
SFO2         500.1320005 MHz

F2 - Processing parameters
SI                 32768
SF           125.7578844 MHz
WDW                   EM
SSB      0
LB                  1.00 Hz
GB       0
PC                  1.40
```

Reaction mixture:

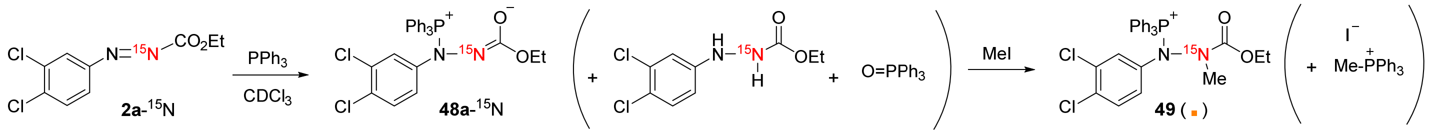

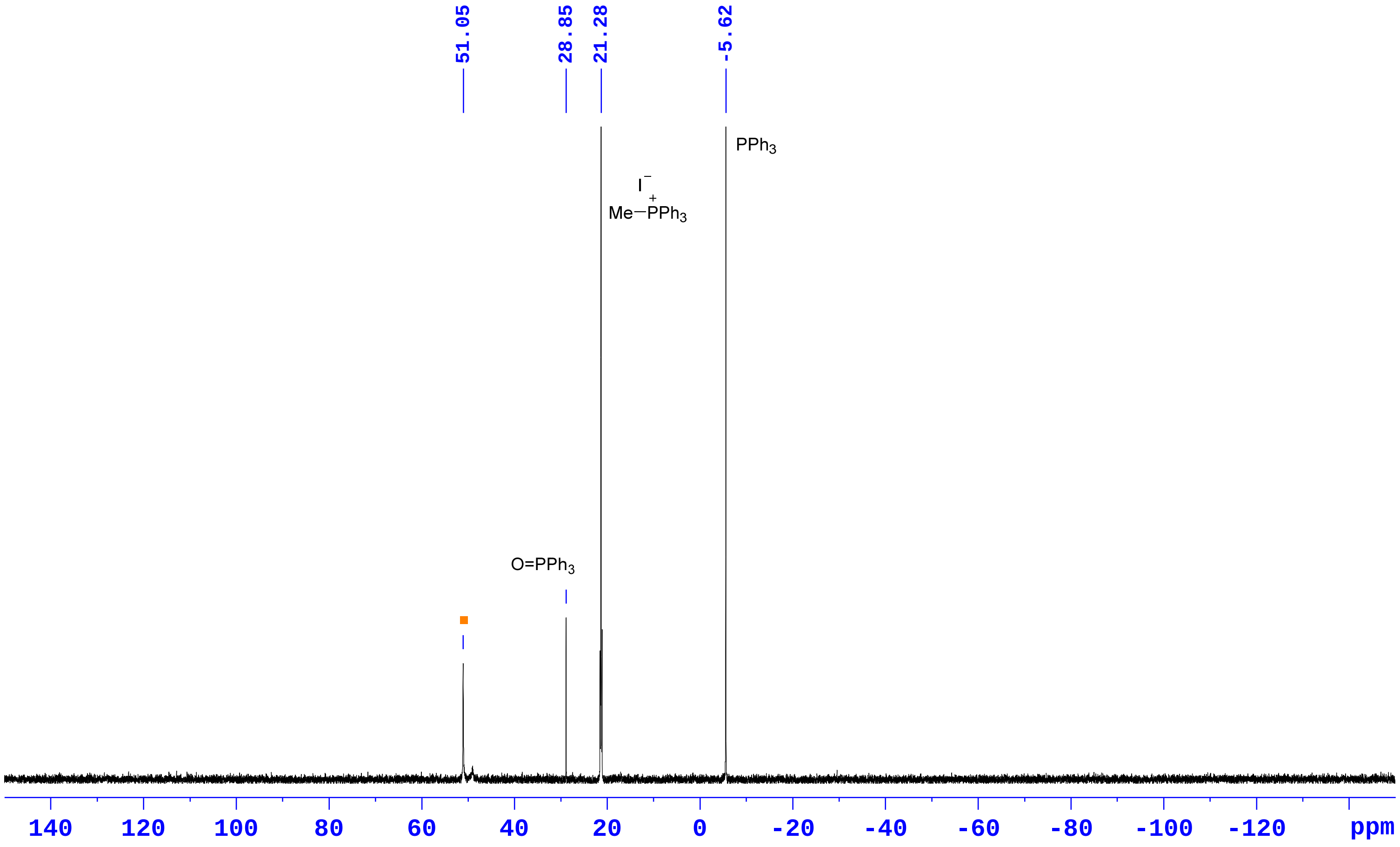


```
Current Data Parameters
NAME              MGS-620
EXPNO                   2
PROCNO                  1

F2 - Acquisition Parameters
Date_            20150808
Time                14.39
INSTRUM             spect
PROBHD   5 mm PABBO BB-
PULPROG           zgpg30
TD                 65536
SOLVENT            CDCl3
NS                    16
DS                     4
SWH           81521.742 Hz
FIDRES         1.243923 Hz
AQ            0.4020041 sec
RG                  2050
DW                 6.133 usec
DE                  6.50 usec
TE                 299.5 K
D1            2.00000000 sec
D11           0.03000000 sec

======== CHANNEL f1 ========
NUC1                 31P
P1                 10.00 usec
PLW1        100.00000000 W
SFO1         202.4462121 MHz

======== CHANNEL f2 ========
CPDPRG2          waltz16
NUC2                  1H
PCPD2              80.00 usec
PLW2         26.00000000 W
PLW12         0.32179001 W
PLW13         0.20595001 W
SFO2         500.1320005 MHz

F2 - Processing parameters
SI                 32768
SF           202.4563350 MHz
WDW                   EM
SSB      0
LB                  1.00 Hz
GB       0
PC                  1.40
```

Reaction mixture:

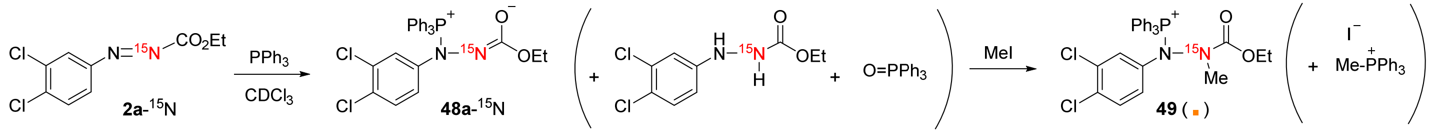

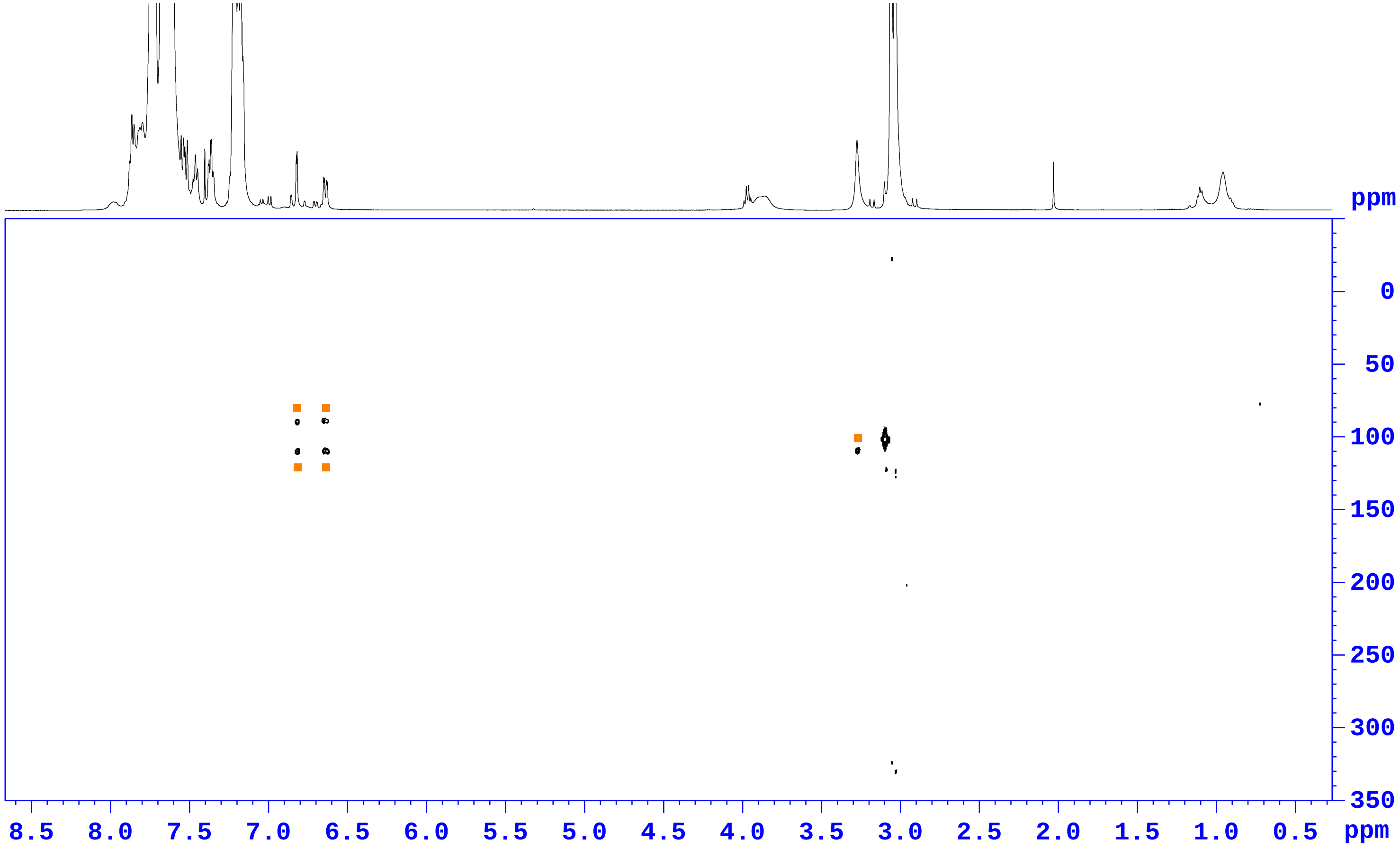


```
Current Data Parameters
NAME              MGS-620
EXPNO                  10
PROCNO                  1

F2 - Acquisition Parameters
Date_            20150808
Time                16.40
INSTRUM             spect
PROBHD    5 mm PABBO BB-
PULPROG       hmbcgpndqf
TD                   2048
SOLVENT             CDCl3
NS                      8
DS                     16
SWH              4201.681 Hz
FIDRES           2.051602 Hz
AQ              0.2437620 sec
RG                   2050
DW                119.000 usec
DE                   6.50 usec
TE                  299.5 K
CNST13          5.0000000
D0             0.00000300 sec
D1             1.91357398 sec
D6             0.10000000 sec
D16            0.00020000 sec
IN0            0.00002465 sec

======== CHANNEL f1 ========
NUC1                   1H
P1                   8.90 usec
P2                  17.80 usec
PLW1          26.00000000 W
SFO1          500.1321846 MHz

======== CHANNEL f2 ========
NUC2                  15N
P3                  14.40 usec
PLW2         206.00000000 W
SFO2           50.6853342 MHz

====== GRADIENT CHANNEL =====
GPNAM1         SMSQ10.100
GPNAM2         SMSQ10.100
GPNAM3         SMSQ10.100
GPZ1                70.00 %
GPZ2                30.00 %
GPZ3                50.10 %
P16               1000.00 usec

F1 - Acquisition parameters
TD                    128
SFO1             50.68533 MHz
FIDRES         158.391663 Hz
SW                400.000 ppm
FnMODE                 QF

F2 - Processing parameters
SI                   2048
SF            500.1299502 MHz
WDW                  SINE
SSB      0
LB       0 Hz
GB       0
PC                   1.40

F1 - Processing parameters
SI                   1024
MC2                    QF
SF             50.6777330 MHz
WDW                States
SSB      0
LB       0 Hz
GB       0
```

Reaction mixture:

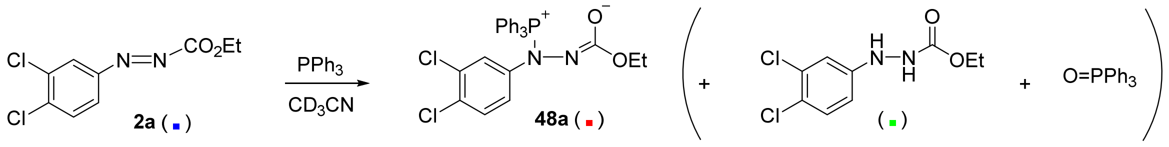

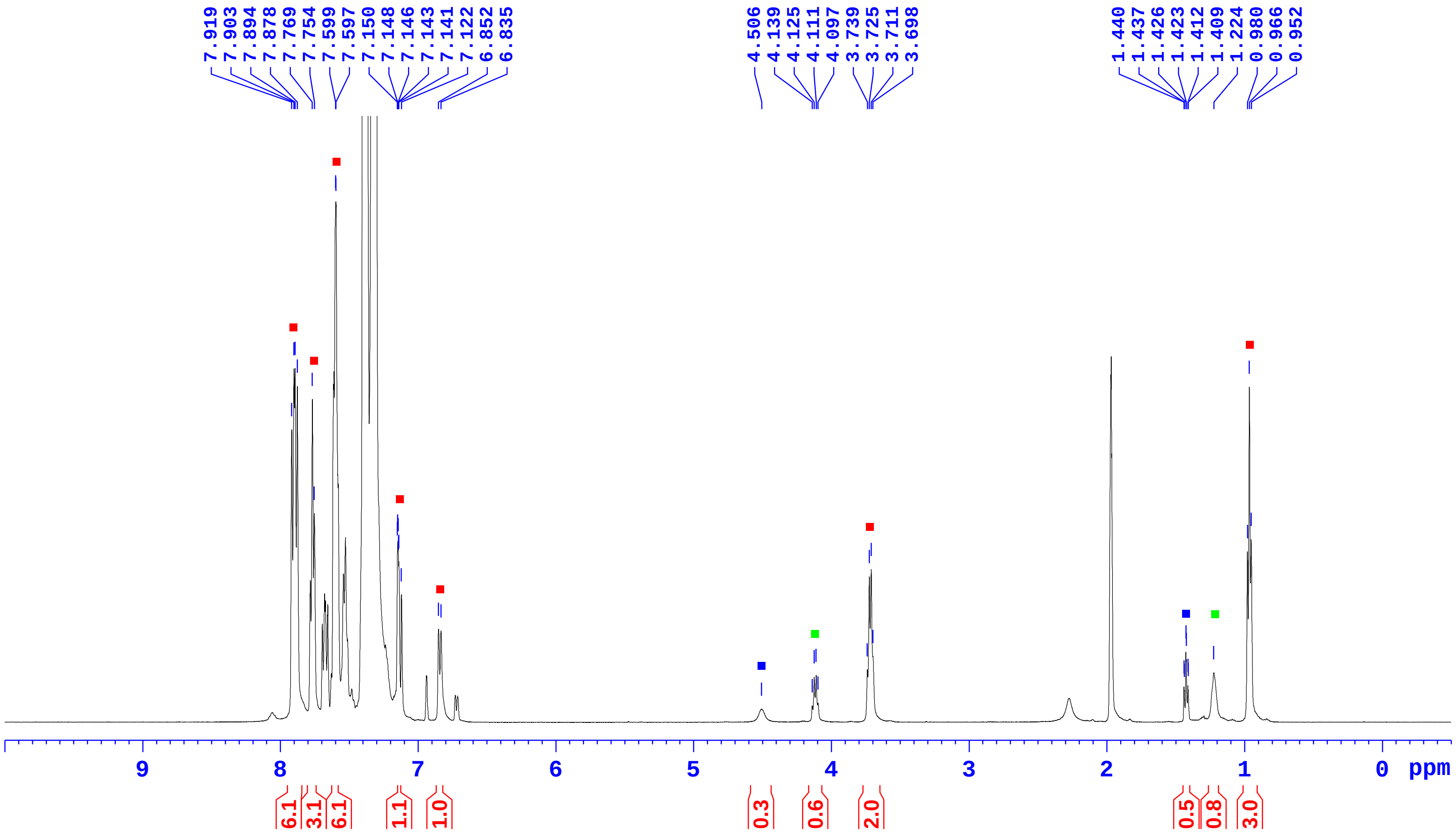

Current Data Parameters
NAME MGS-643
EXPNO 10
PROCNO 1

F2 - Acquisition Parameters
Date_ 20151006
Time 12.07
INSTRUM spect
PROBHD 5 mm PABBO BB-
PULPROG zg30
TD 65536
SOLVENT CD3CN
NS 16
DS 2
SWH 10330.578 Hz
FIDRES 0.157632 Hz
AQ 3.1719923 sec
RG 32
DW 48.400 usec
DE 6.50 usec
TE 296.7 K
D1 1.00000000 sec

======== CHANNEL f1 ========
NUC1 1H
P1 8.90 usec
PLW1 26.00000000 W
SFO1 500.1330885 MHz

F2 - Processing parameters
SI 65536
SF 500.1300000 MHz
WDW EM
SSB 0
LB 0.30 Hz
GB 0
PC 1.00

Reaction mixture:

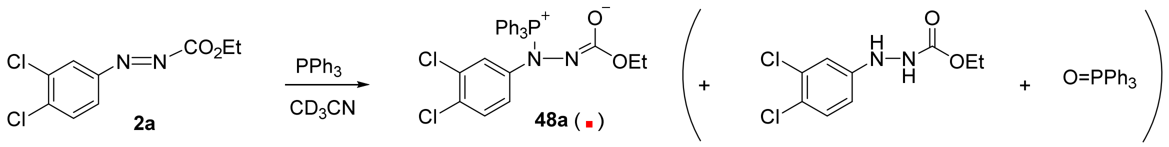

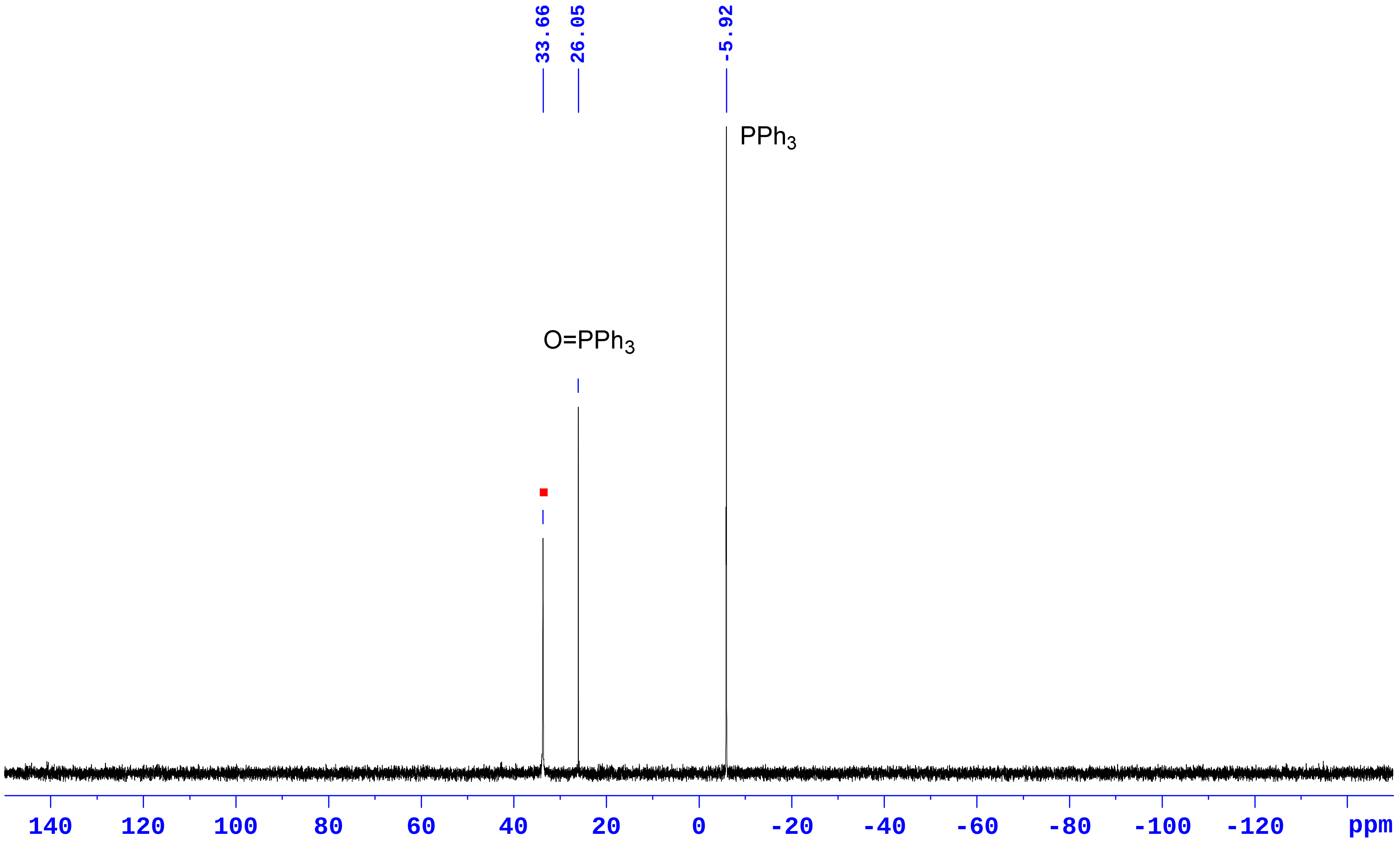


```
Current Data Parameters
NAME              MGS-643
EXPNO                  12
PROCNO                  1

F2 - Acquisition Parameters
Date_            20151006
Time                12.12
INSTRUM             spect
PROBHD   5 mm PABBO BB-
PULPROG           zgpg30
TD                 65536
SOLVENT            CD3CN
NS                    16
DS                     4
SWH           81521.742 Hz
FIDRES         1.243923 Hz
AQ            0.4020041 sec
RG                  2050
DW                 6.133 usec
DE                  6.50 usec
TE                 296.9 K
D1            2.00000000 sec
D11           0.03000000 sec

======== CHANNEL f1 ========
NUC1                 31P
P1                 10.00 usec
PLW1        100.00000000 W
SFO1         202.4462121 MHz

======== CHANNEL f2 ========
CPDPRG2          waltz16
NUC2                  1H
PCPD2              80.00 usec
PLW2         26.00000000 W
PLW12         0.32179001 W
PLW13         0.20595001 W
SFO2         500.1320005 MHz

F2 - Processing parameters
SI                 32768
SF           202.4563350 MHz
WDW                   EM
SSB      0
LB                  1.00 Hz
GB       0
PC                  1.40
```

Reaction mixture:

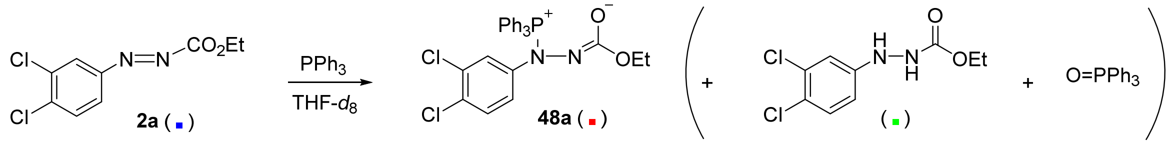

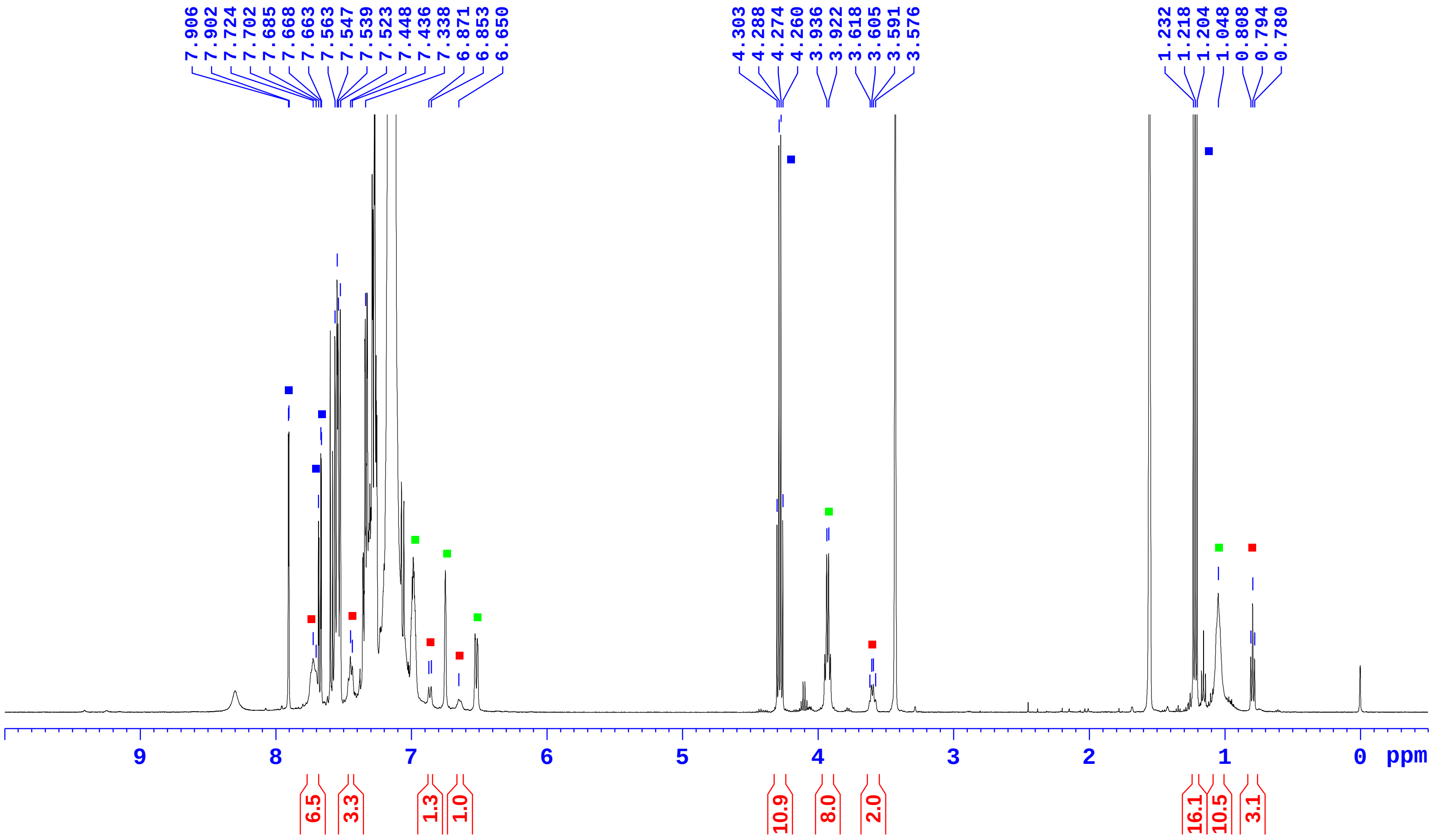


```
Current Data Parameters
NAME                MGS-625
EXPNO                     1
PROCNO                    1

F2 - Acquisition Parameters
Date_              20150910
Time                  17.13
INSTRUM               spect
PROBHD   5 mm PABBO BB-
PULPROG                zg30
TD                    65536
SOLVENT                 THF
NS                       16
DS                        2
SWH           10330.578 Hz
FIDRES         0.157632 Hz
AQ            3.1719923 sec
RG                       18
DW               48.400 usec
DE                 6.50 usec
TE                296.0 K
D1           1.00000000 sec

======== CHANNEL f1 ========
NUC1                     1H
P1                 8.90 usec
PLW1        26.00000000 W
SFO1        500.1330885 MHz

F2 - Processing parameters
SI                    65536
SF          500.1301070 MHz
WDW                      EM
SSB      0
LB                  0.30 Hz
GB       0
PC                     1.00
```

Reaction mixture:

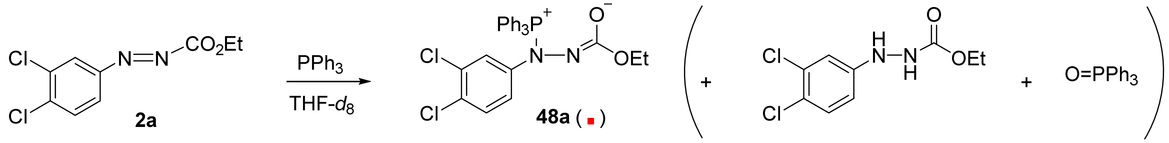

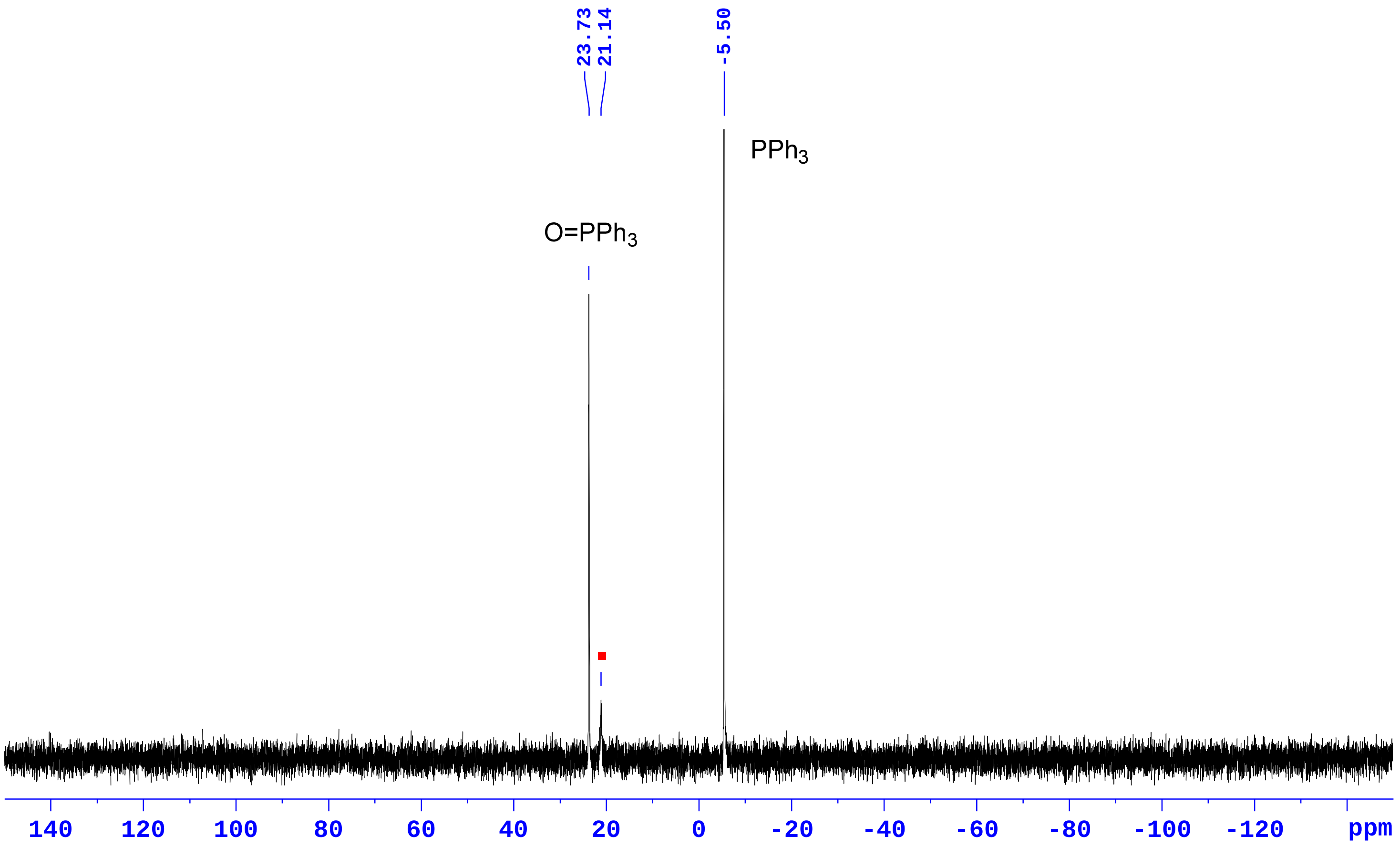


```
Current Data Parameters
NAME               MGS-625
EXPNO                    2
PROCNO                   1

F2 - Acquisition Parameters
Date_             20150910
Time                 17.15
INSTRUM              spect
PROBHD   5 mm PABBO BB-
PULPROG               zg30
TD                   65536
SOLVENT                THF
NS                      32
DS                       4
SWH             81521.742 Hz
FIDRES           1.243923 Hz
AQ              0.4020041 sec
RG                     203
DW                   6.133 usec
DE                    6.50 usec
TE                   296.0 K
D1              2.00000000 sec

======== CHANNEL f1 ========
NUC1                   31P
P1                   10.00 usec
PLW1       100.00000000 W
SFO1           202.4462121 MHz

F2 - Processing parameters
SI                   32768
SF             202.4563370 MHz
WDW                     EM
SSB      0
LB                    1.00 Hz
GB       0
PC                    1.40
```

Reaction mixture:

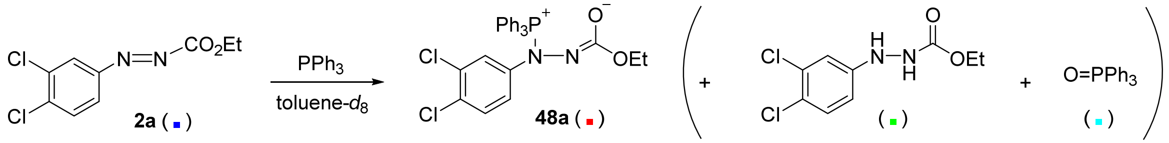

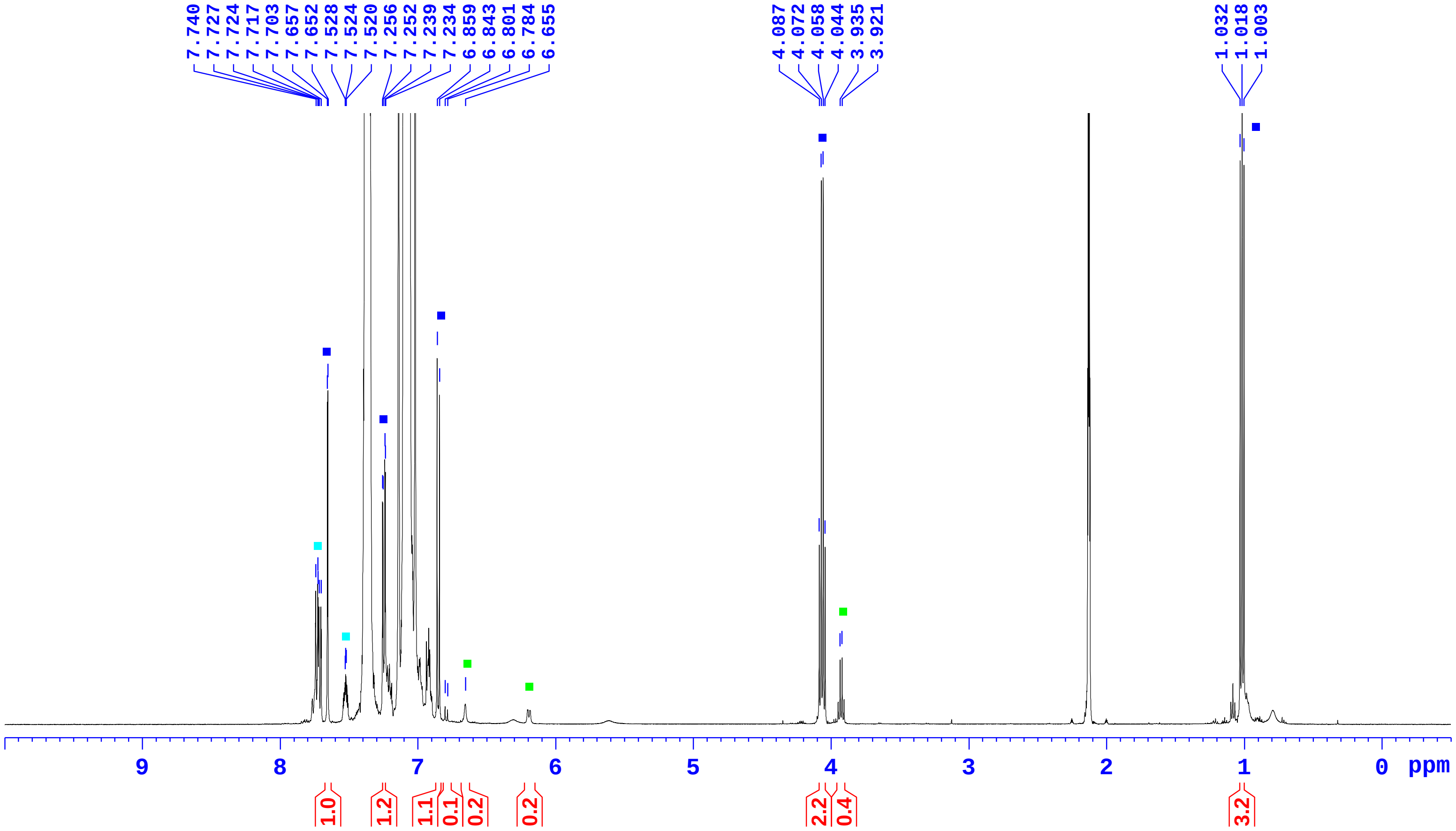


```
Current Data Parameters
NAME               MGS-652
EXPNO                   10
PROCNO                   1

F2 - Acquisition Parameters
Date_             20151022
Time                 10.48
INSTRUM              spect
PROBHD   5 mm PABBO BB-
PULPROG               zg30
TD                   65536
SOLVENT                Tol
NS                      16
DS                       2
SWH             10330.578 Hz
FIDRES           0.157632 Hz
AQ              3.1719923 sec
RG                      32
DW                  48.400 usec
DE                    6.50 usec
TE                   296.0 K
D1              1.00000000 sec

======== CHANNEL f1 ========
NUC1                    1H
P1                    8.90 usec
PLW1           26.00000000 W
SFO1           500.1330885 MHz

F2 - Processing parameters
SI                   65536
SF             500.1300000 MHz
WDW                     EM
SSB      0
LB                    0.30 Hz
GB       0
PC                    1.00
```

Reaction mixture:

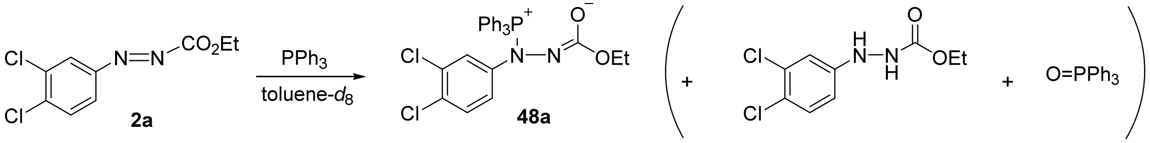

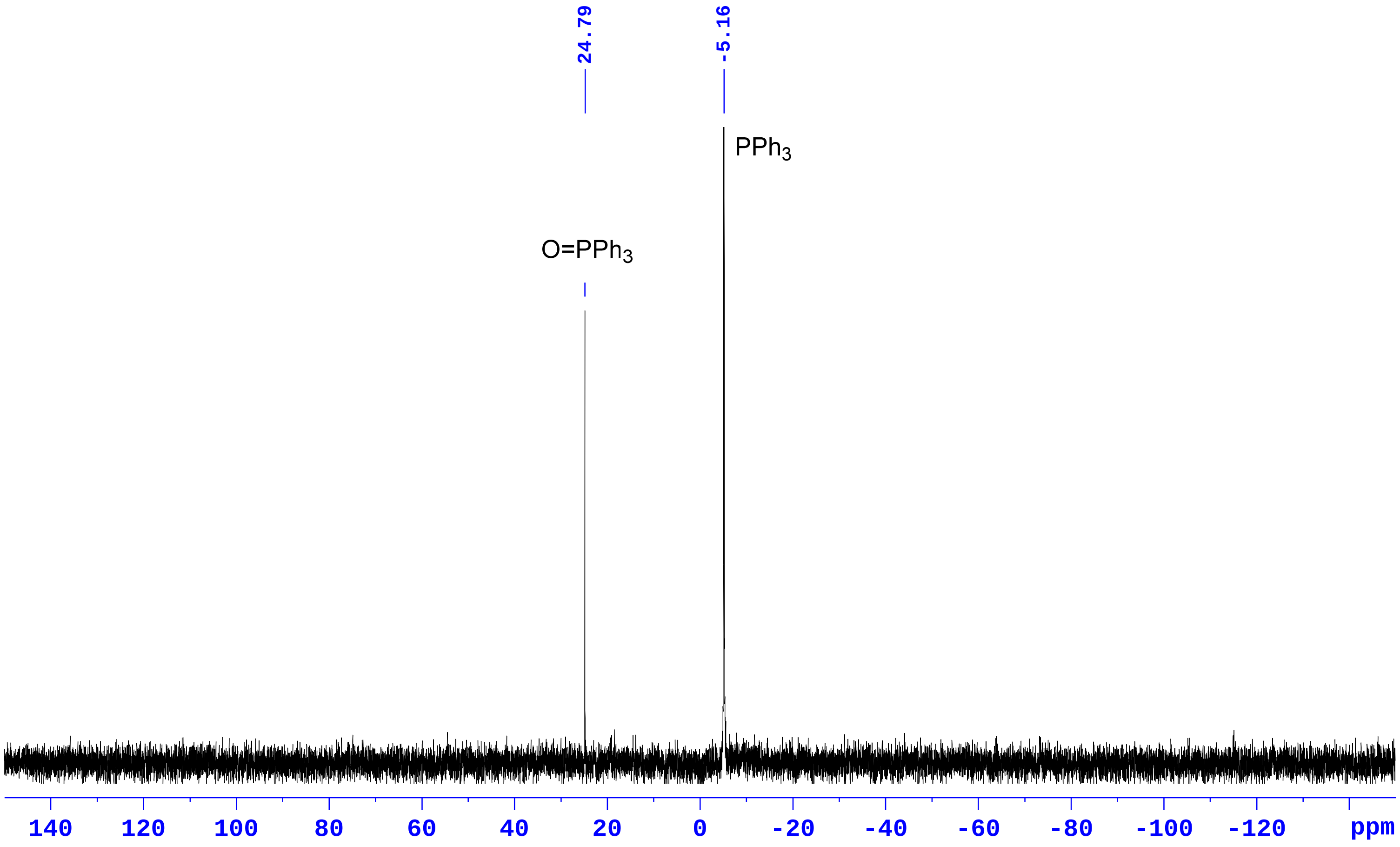


```
Current Data Parameters
NAME              MGS-662
EXPNO                   3
PROCNO                  1

F2 - Acquisition Parameters
Date_            20151117
Time                10.18
INSTRUM             spect
PROBHD    5 mm Multinucl
PULPROG           zgpg30
TD                  65536
SOLVENT               Tol
NS                     32
DS                      4
SWH           48661.801 Hz
FIDRES         0.742520 Hz
AQ            0.6734324 sec
RG              10321.3
DW               10.275 usec
DE                 6.00 usec
TE                302.0 K
D1           2.00000000 sec
d11          0.03000000 sec
DELTA        1.89999998 sec
TD0                     1

======== CHANNEL f1 ========
NUC1                  31P
P1                  10.60 usec
PL1                 -4.00 dB
SFO1         121.4887762 MHz

======== CHANNEL f2 ========
CPDPRG2           waltz16
NUC2                   1H
PCPD2               75.00 usec
PL2         0 dB
PL12                19.10 dB
PL13               120.00 dB
SFO2         300.1312005 MHz

F2 - Processing parameters
SI                  32768
SF           121.4948510 MHz
WDW                    EM
SSB         0
LB                   1.00 Hz
GB          0
PC                   1.40
```

151112

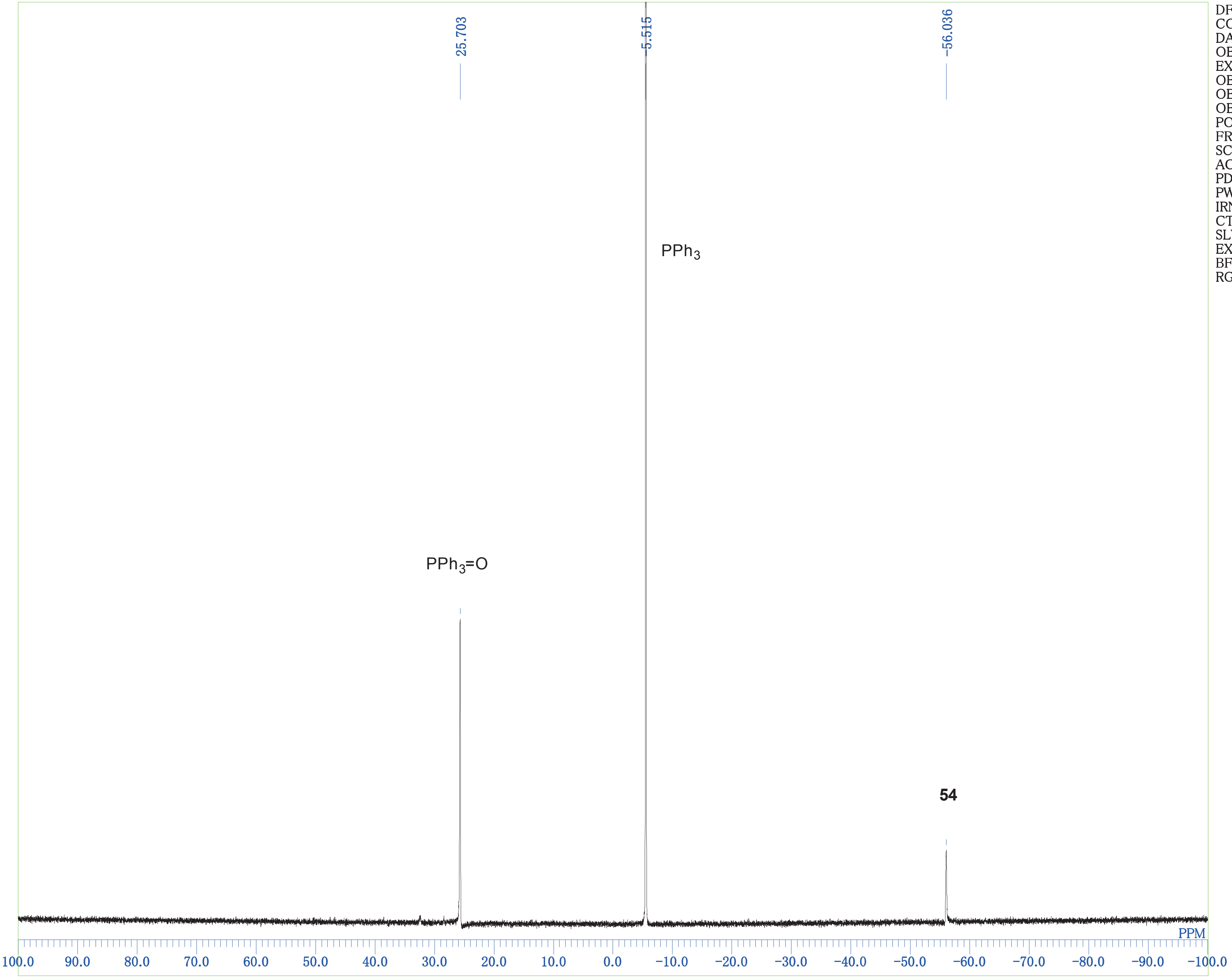

DFILE	54-thfd8.als
COMNT	151112
DATIM	2015-11-12 10:47:05
OBNUC	31P
EXMOD	single_pulse_dec
OBFRQ	242.95 MHz
OBSET	4.04 KHz
OBFIN	1.25 Hz
POINT	26214
FREQU	49018.86 Hz
SCANS	32
ACQTM	0.5348 sec
PD	3.0000 sec
PW1	6.75 usec
IRNUC	1H
CTEMP	25.0 c
SLVNT	C4D8O
EXREF	-5.90 ppm
BF	1.20 Hz
RGAIN	46

$(n\mathrm{BuO})_2\mathrm{PPh}_3$

54

in THF-d_8

151115

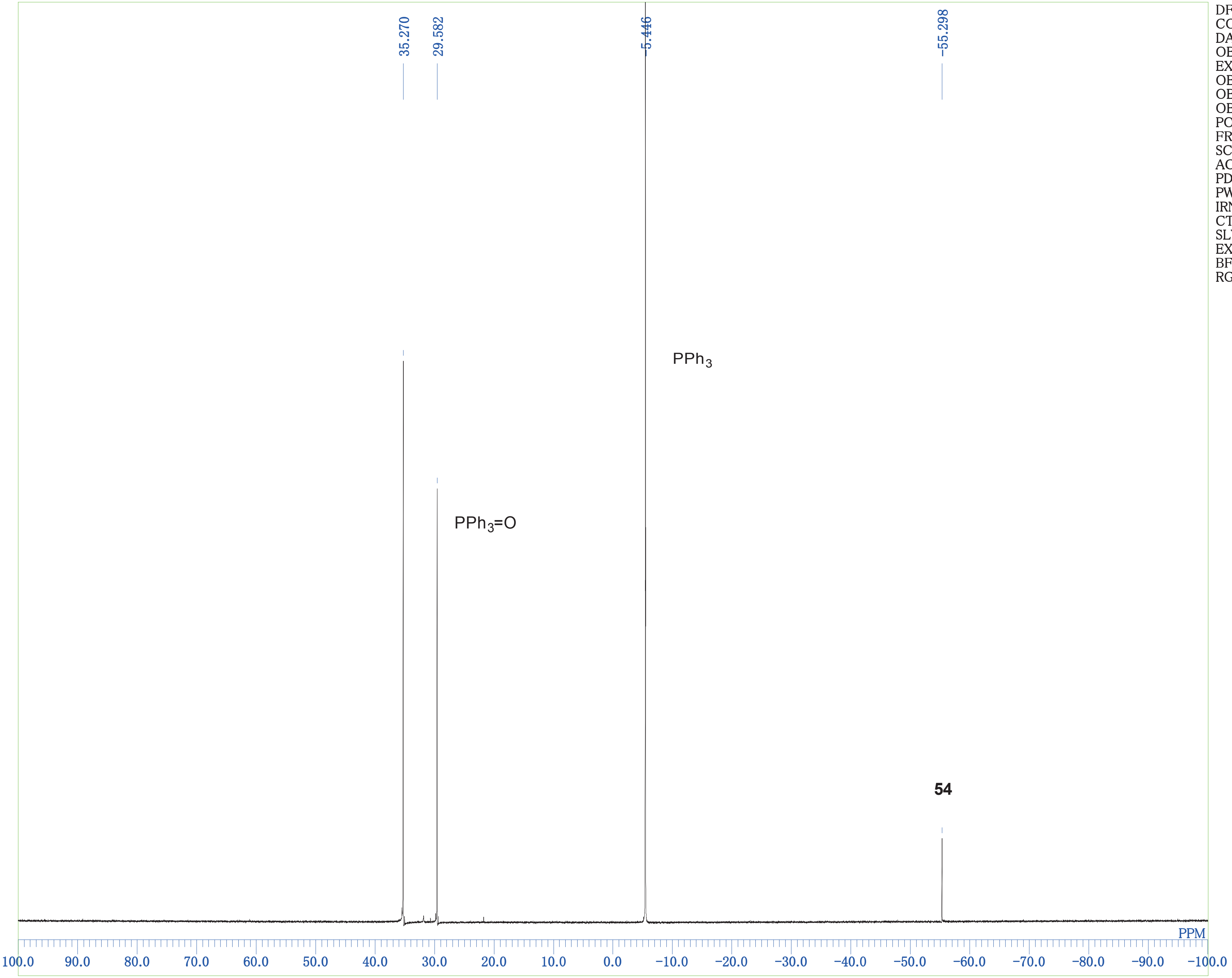

DFILE	54−cdcl3.als
COMNT	151115
DATIM	2015−11−15 13:08:19
OBNUC	31P
EXMOD	single_pulse_dec
OBFRQ	242.95 MHz
OBSET	4.04 KHz
OBFIN	1.25 Hz
POINT	26214
FREQU	49018.86 Hz
SCANS	32
ACQTM	0.5348 sec
PD	3.0000 sec
PW1	6.75 usec
IRNUC	1H
CTEMP	25.0 c
SLVNT	CDCL3
EXREF	−5.90 ppm
BF	1.20 Hz
RGAIN	46

$(n\text{BuO})_2\text{PPh}_3$

54

in $CDCl_3$

151115

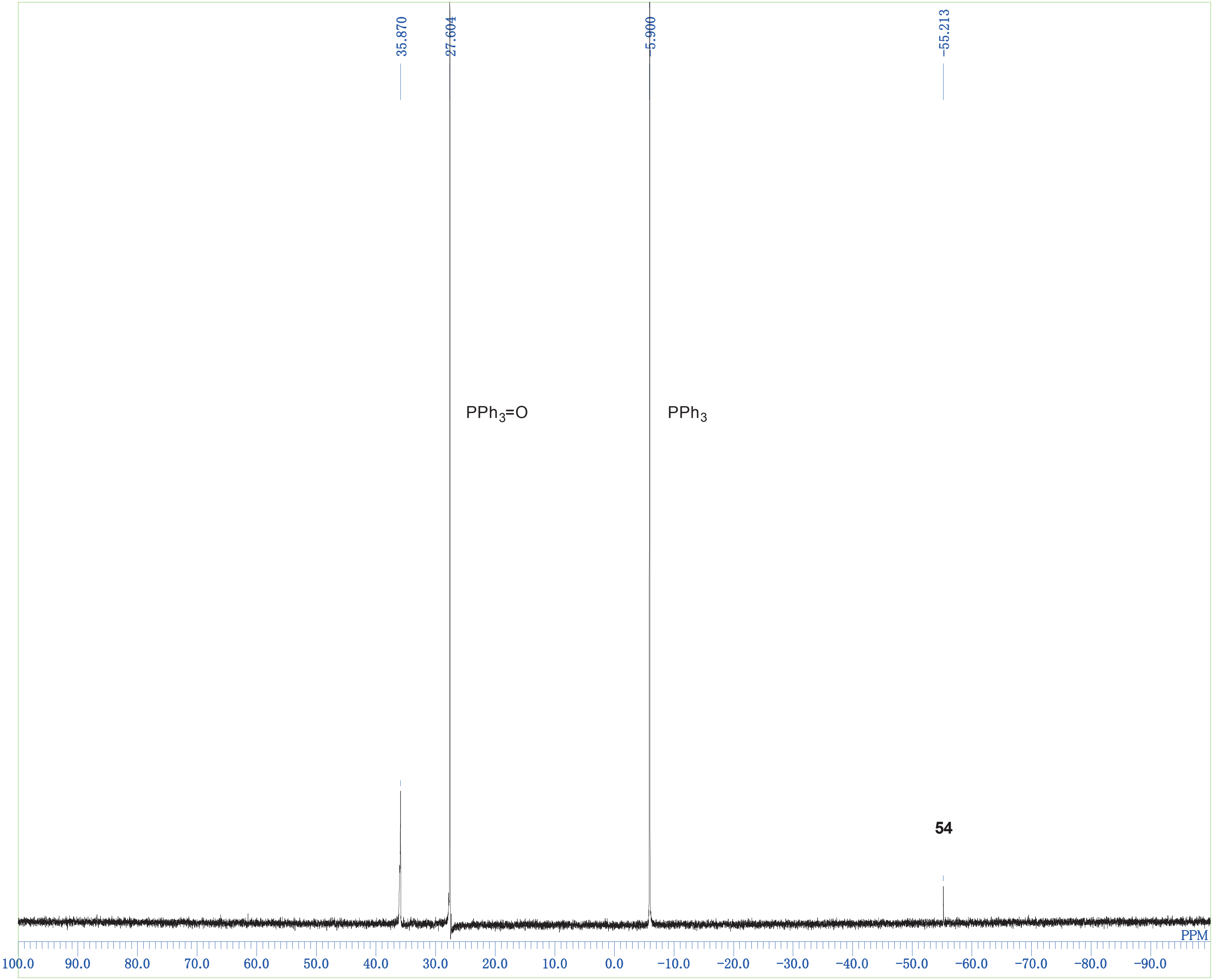

DFILE	54-cd3cn.als
COMNT	151115
DATIM	2015-11-15 12:16:52
OBNUC	31P
EXMOD	single_pulse_dec
OBFRQ	242.95 MHz
OBSET	4.04 KHz
OBFIN	1.25 Hz
POINT	26214
FREQU	49018.86 Hz
SCANS	32
ACQTM	0.5348 sec
PD	3.0000 sec
PW1	6.75 usec
IRNUC	1H
CTEMP	25.0 c
SLVNT	CD3CN
EXREF	-5.90 ppm
BF	1.20 Hz
RGAIN	50

$(n\text{BuO})_2PPh_3$

54

in CD_3CN

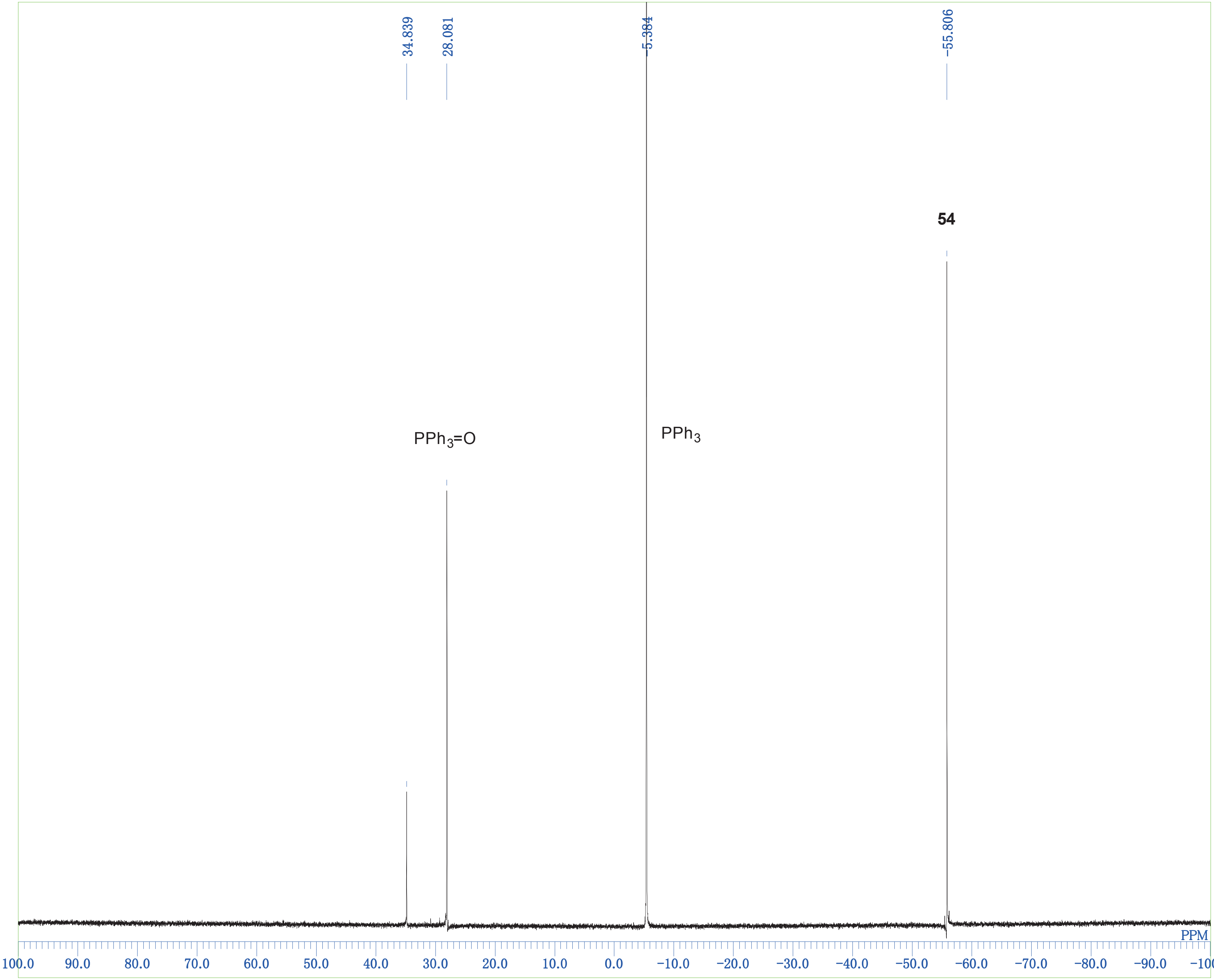

DFILE	54-toluened8.als
COMNT	151114
DATIM	2015-11-14 14:55:41
OBNUC	31P
EXMOD	single_pulse_dec
OBFRQ	242.95 MHz
OBSET	4.04 KHz
OBFIN	1.25 Hz
POINT	26214
FREQU	49018.86 Hz
SCANS	32
ACQTM	0.5348 sec
PD	3.0000 sec
PW1	6.75 usec
IRNUC	1H
CTEMP	25.0 c
SLVNT	C6D5CD3
EXREF	-5.90 ppm
BF	1.20 Hz
RGAIN	46

$(n\text{BuO})_2\text{PPh}_3$

54

in toluene-d_8

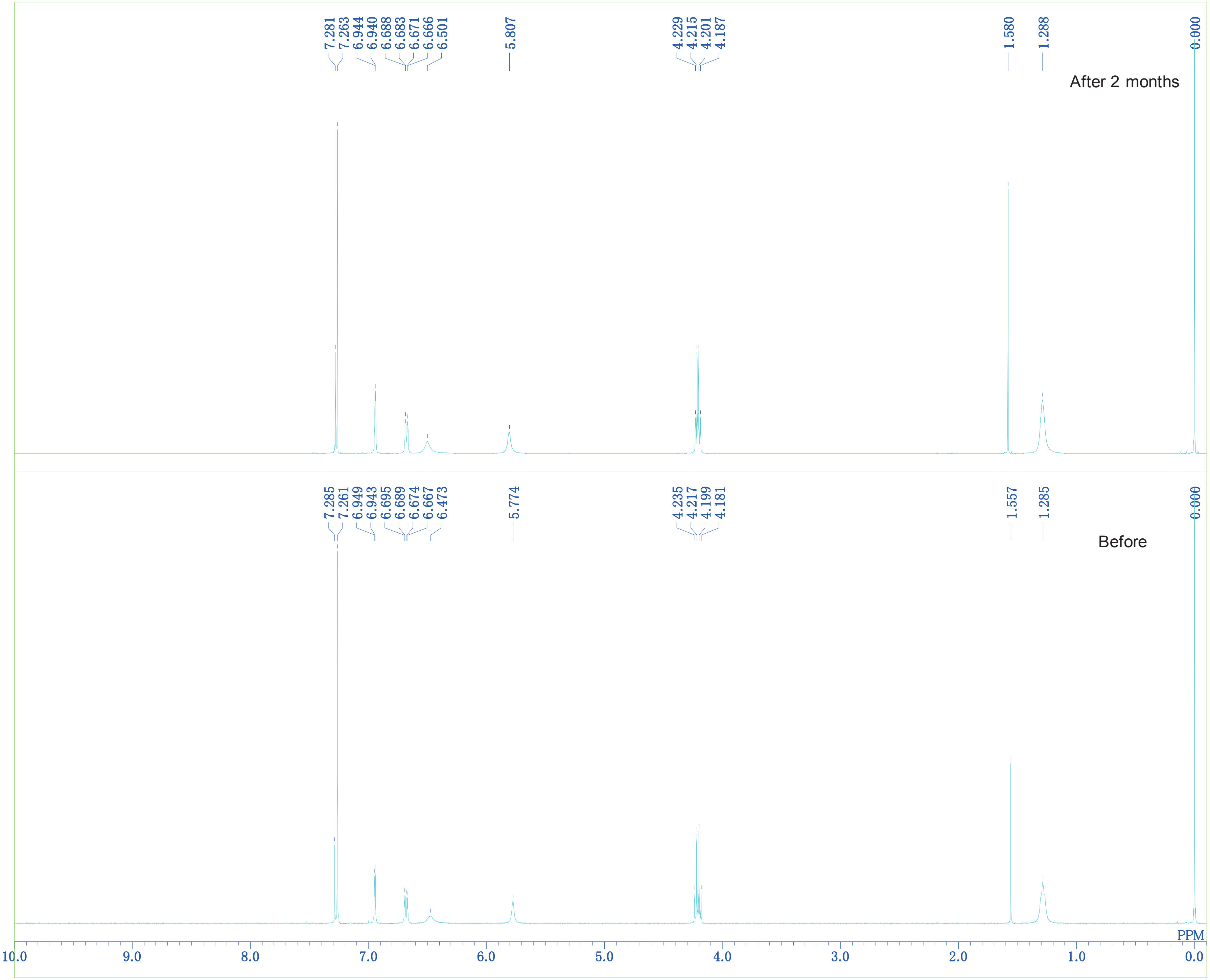

DFILE	1a-beforeafter.als
COMNT	141222
DATIM	2014-12-22 19:46:55
OBNUC	1H
EXMOD	single_pulse.ex2
OBFRQ	399.78 MHz
OBSET	4.19 KHz
OBFIN	7.29 Hz
POINT	13107
FREQU	6002.31 Hz
SCANS	8
ACQTM	2.1837 sec
PD	2.0000 sec
PW1	5.00 usec
IRNUC	1H
CTEMP	20.4 c
SLVNT	CDCL3
EXREF	0.00 ppm
BF	0.12 Hz
RGAIN	50

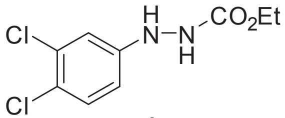

1a

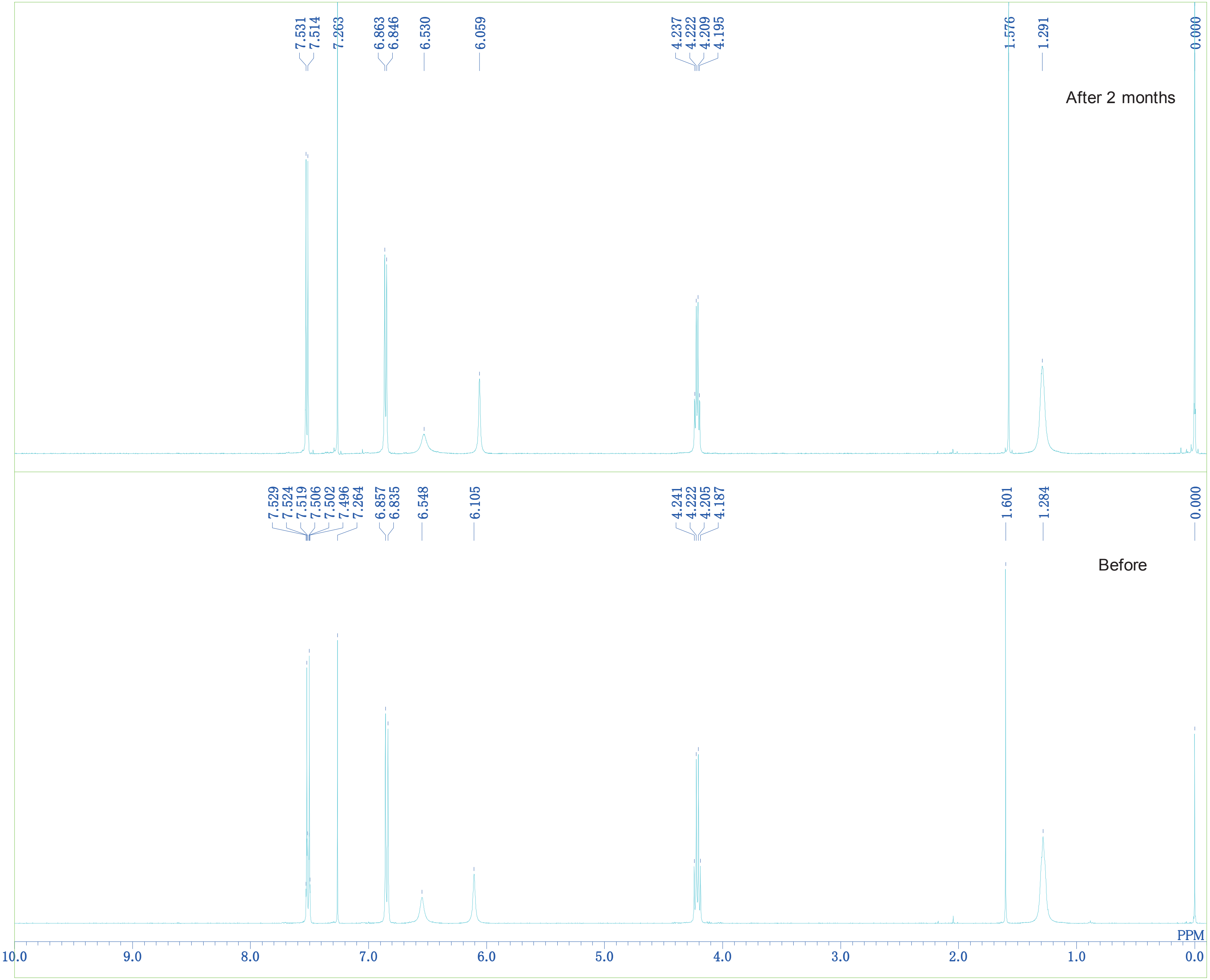

DFILE	1h-beforeafter.als
COMNT	150501
DATIM	2015-05-01 14:11:40
OBNUC	1H
EXMOD	single_pulse.ex2
OBFRQ	399.78 MHz
OBSET	4.19 KHz
OBFIN	7.29 Hz
POINT	13107
FREQU	6002.31 Hz
SCANS	8
ACQTM	2.1837 sec
PD	2.0000 sec
PW1	4.70 usec
IRNUC	1H
CTEMP	22.9 c
SLVNT	CDCL3
EXREF	0.00 ppm
BF	0.12 Hz
RGAIN	48

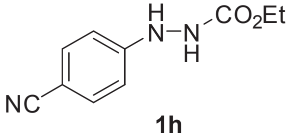

1h

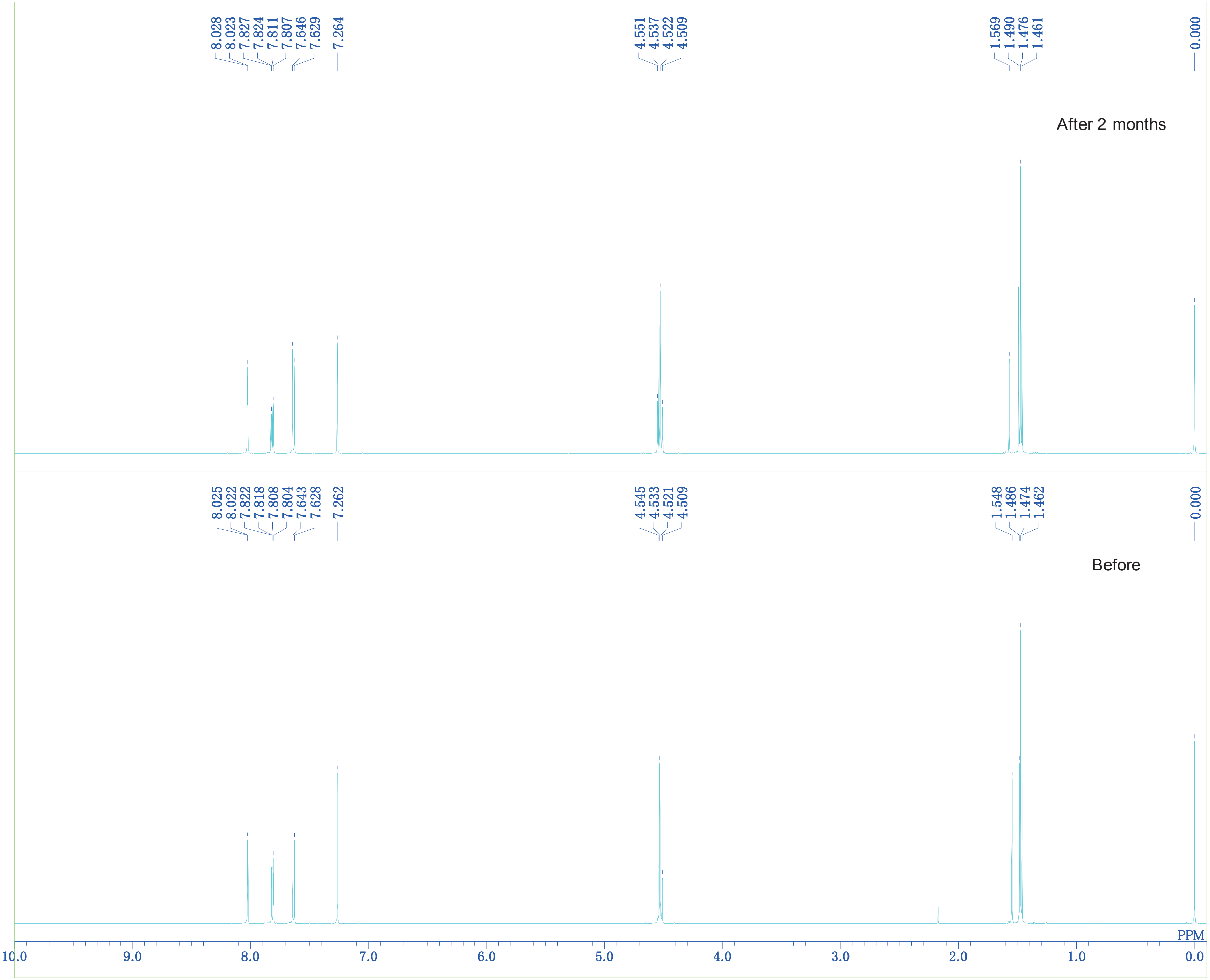

DFILE	2a–beforeafter.als
COMNT	150524
DATIM	2015–05–24 13:27:23
OBNUC	1H
EXMOD	single_pulse.ex2
OBFRQ	600.17 MHz
OBSET	5.30 KHz
OBFIN	5.47 Hz
POINT	26214
FREQU	9008.87 Hz
SCANS	8
ACQTM	2.9098 sec
PD	2.0000 sec
PW1	7.30 usec
IRNUC	1H
CTEMP	22.9 c
SLVNT	CDCL3
EXREF	0.00 ppm
BF	0.12 Hz
RGAIN	54

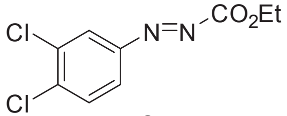

2a

DFILE	2h−beforeafter.als
COMNT	150520
DATIM	2015−05−20 15:22:12
OBNUC	1H
EXMOD	single_pulse.ex2
OBFRQ	500.16 MHz
OBSET	2.41 KHz
OBFIN	6.01 Hz
POINT	13107
FREQU	7507.39 Hz
SCANS	8
ACQTM	1.7459 sec
PD	2.0000 sec
PW1	5.80 usec
IRNUC	1H
CTEMP	20.0 c
SLVNT	CDCL3
EXREF	0.00 ppm
BF	0.12 Hz
RGAIN	48

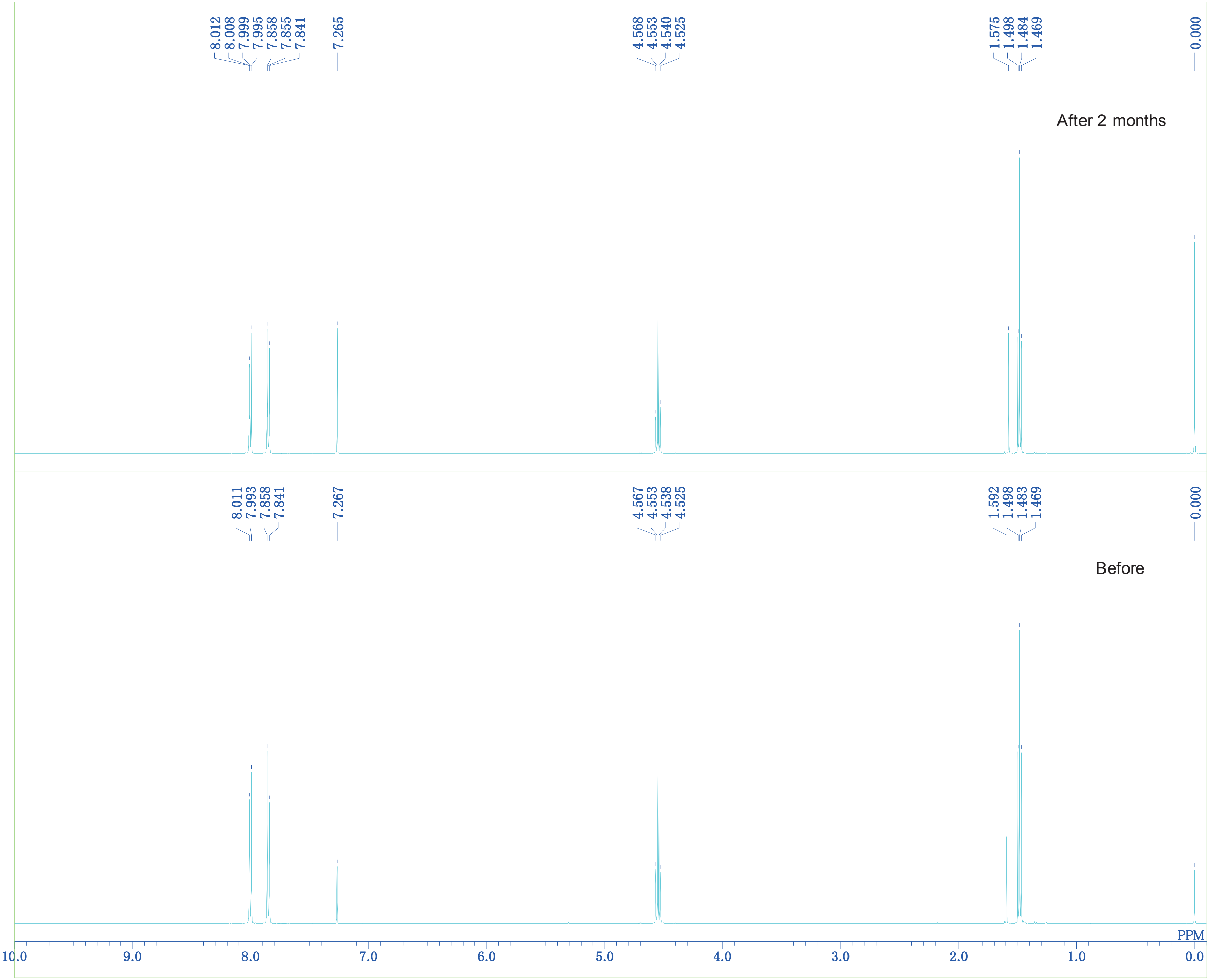

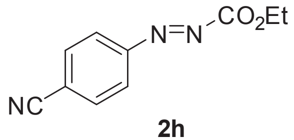

2h

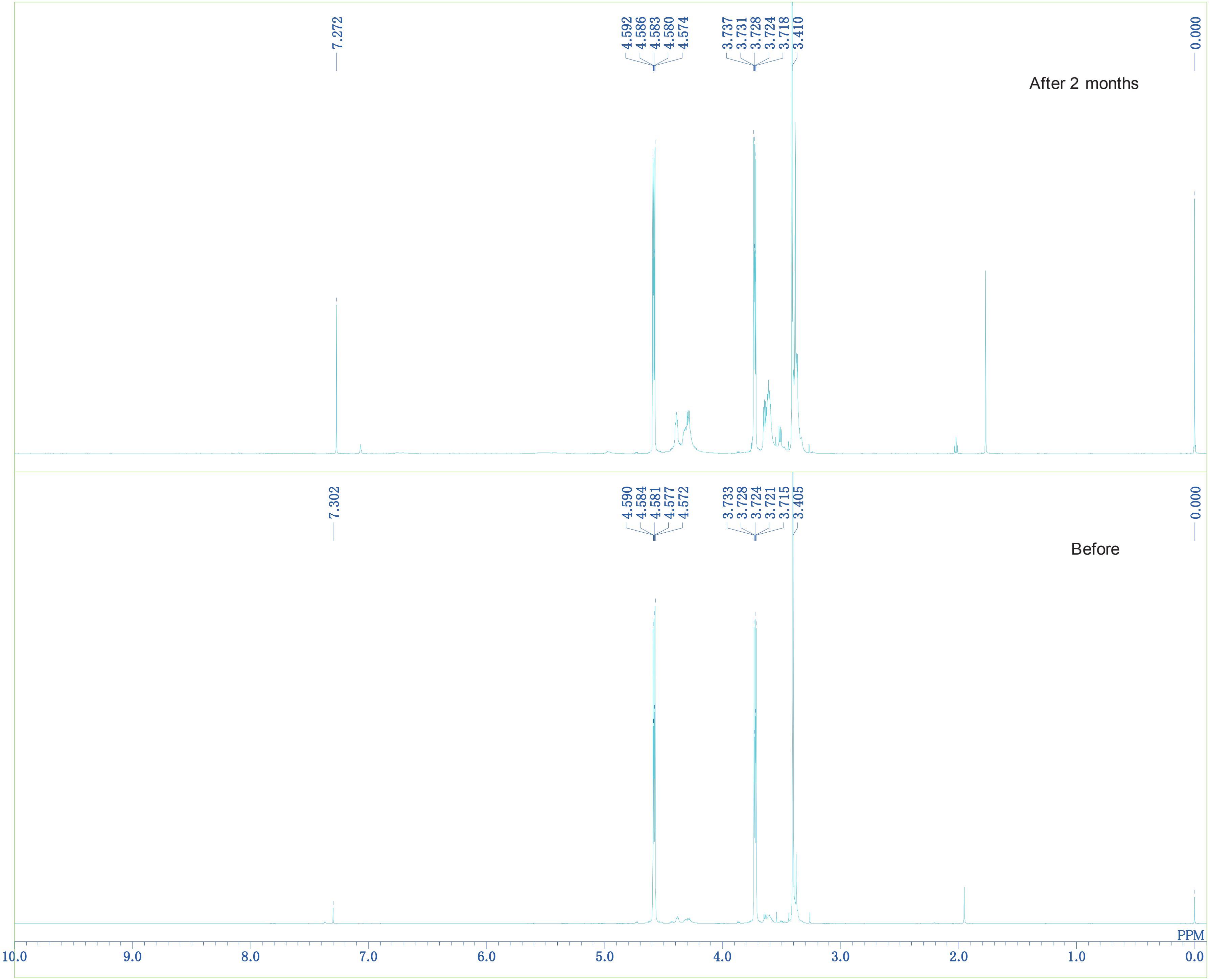

DFILE	DMEAD-beforeafter.als
COMNT	151105
DATIM	2015-11-05 16:06:46
OBNUC	1H
EXMOD	single_pulse.ex2
OBFRQ	500.16 MHz
OBSET	2.41 KHz
OBFIN	6.01 Hz
POINT	13107
FREQU	7507.39 Hz
SCANS	8
ACQTM	1.7459 sec
PD	2.0000 sec
PW1	5.80 usec
IRNUC	1H
CTEMP	19.6 c
SLVNT	CDCL3
EXREF	0.00 ppm
BF	0.12 Hz
RGAIN	30

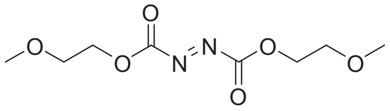

DMEAD
